# Identification of Novel Multi-Omics Expression Landscapes and Meta-Analysis of Landscape-Based Competitive Endogenous RNA Networks in ALDH+ Lung Adenocarcinoma Stem Cells

**DOI:** 10.1155/2022/9545609

**Published:** 2022-08-31

**Authors:** Wei Yang, Yong Liang, Yuanyuan Zheng, Haitao Luo, Xiaofei Yang, Furong Li

**Affiliations:** ^1^Translational Medicine Collaborative Innovation Center, The Second Clinical Medical College (Shenzhen People's Hospital), Jinan University, Shenzhen 518020, China; ^2^The First Affiliated Hospital (Shenzhen People's Hospital), Southern University of Science and Technology, Shenzhen 518020, China; ^3^Guangdong Engineering Technology Research Center of Stem cell and Cell Therapy, Shenzhen 518020, China; ^4^Shenzhen Key Laboratory of Stem Cell Research and Clinical Transformation, Shenzhen 518020, China; ^5^Shenzhen Xbiome Biotech Co., Ltd, Shenzhen 518057, China

## Abstract

ALDH+ H1975 lung adenocarcinoma stem cells (LSCs) are a rare cell population identified in lung adenocarcinoma (LUAD). LSCs can self-renew, drive tumor initiation, growth, metastasis, and recurrence and are also the predominant cause of poor prognosis due to their intrinsic resistance to drugs and chemotherapy. Consequently, LSCs are a promising target for LUAD therapy. Noncoding RNAs (ncRNAs), including microRNAs (miRNAs), long noncoding RNAs (lncRNAs), and circular RNAs (circRNAs), exert many significant regulatory functions in the pathogenesis of human cancers, showing the necessity for a comprehensive understanding of the mechanisms that underlie lung carcinogenesis. Nonetheless, research on many known transcripts and messenger RNAs (mRNAs) has already generated new information. Unknown biomarkers in ncRNAs and systematic and comprehensive interrelation with unknown ncRNAs and mRNAs may provide further insights into the biology of LUAD. Herein, a set of novel ncRNAs that include miRNAs, lncRNAs, and circRNAs were identified, and differentially expressed patterns of ncRNAs and mRNAs in LSCs and ALDH-H1975 LUAD tumor cells (LTCs) were obtained using stringent bioinformatics pipelines. Through a meta-analysis of the identified landscapes, novel competitive endogenous RNA (ceRNA) networks were constructed to reveal the potential molecular mechanisms that regulate the hallmarks of LSCs and LTCs. This study presents a summary of novel ncRNAs and the fundamental roles of differentially expressed ncRNAs implicated in the activity of LSCs and LTCs. In addition, the study also provides a comprehensive resource for the future identification of diagnostic, therapeutic, and prognostic biomarkers in LUAD.

## 1. Introduction

Lung cancer is the most common respiratory tumor and is the leading cause of cancer deaths worldwide, with ~18% mortality rate and ~22.4% incident ratio among all cancer deaths [2, 3]. Non-small cell lung cancer (NSCLC) is the most common form of lung cancer, accounting for ~90% of all cases, and lung adenocarcinoma (LUAD) is its most common histological subtype [4], with a dismal prognosis and 15% of patients surviving 5 years after diagnosis [5].

Cancer stem cells (CSCs), a subset of cancer cells with stem-like characteristics, play a critical role in tumor heterogeneity and are involved in tumor initiation, growth, metastasis, recurrence, and drug resistance [6]. Increasing research has demonstrated that the leading cause of poor prognosis for LUAD [7] is the presence of LUAD stem cells [8], which could initiate and sustain primary and metastatic cancer relapse and growth [9]. Previous evidence [10] and our most recent studies [11, 12] showed that ALDH+ H1975 LUAD cells (LSCs) display strong CSC-like properties [13] and act as LSCs to influence the formation of a tumor niche, tumor initiation, and recurrence [7]. Our most recent study revealed that LSCs have the capacity for self-renewal and differentiation, which facilitates the generation of tumor cells and promotes tumor growth [11], recurrence, and metastasis. Therefore, LSCs are a promising target for interfering with the therapeutic mechanism of LUAD formation and LUAD therapy [7]. Elucidation of the differences and underlying molecular pathways that characterize LSCs [14] is important to identify and evaluate potential diagnostic, therapeutic, and prognostic biomarkers of LUAD [12].

LncRNAs, circRNAs, and miRNAs are three subtypes of non-coding RNAs, which account for a huge proportion of the human transcriptome [15]. The diverse types of these ncRNAs are marked by sequence length, spatial architecture, and regulatory functions in various pathophysiological processes in cancer. Separately, miRNAs [16] are the most studied single-stranded ncRNAs, with a length of ~20 nucleotides [17]. Through binding to specific 3′-untranslated regions (UTRs) of their target mRNAs or RNA, miRNAs could silence the expression of multiple targeted mRNAs and RNA simultaneously [18]. Regulatory modulation of one-to-many miRNAs influences multiple oncogenic and tumor repressive pathways [19, 20]; for example, overexpression of has-miR-17-92 in lung cancer enhances cell proliferation [21], while has-let-7 in lung cancer is associated with the overall survival of patients [22]. LncRNAs are over 200 nucleotides in length [23] and have the ability to regulate gene expression rather than having protein-encoding capability [24]. Extensive studies have shown that lncRNAs may influence cancer progression by regulating mRNAs and miRNAs [25]. CircRNAs are a novel subtype of non-coding RNAs. Although circRNAs function as lncRNAs [26], their uniquely circular structure provides them with greater stability compared with linear lncRNAs [27]. CircRNAs play a vital role in various types of cancer [28]. Furthermore, there is evidence that mRNA transcripts are regulated or repressed by lncRNAs/circRNAs and miRNAs, individually or in combination [29]. A novel mechanism in the regulatory network [30] is the identification of the interaction between mRNAs, lncRNAs/circRNAs, and miRNAs, which has been defined as the ceRNA network. More importantly, lncRNAs/circRNAs upregulate transcript expression by binding miRNA sites, for example, lncRNA HRCR acts as a miR-233 sponge to prevent cardiac hypertrophy [31], lncRNA CHRF functions as an endogenous sponge of miR-489 to limit miR-489 expression [32], and lncRNA APF works as a sponge of miR-188-3p to prevent decomposition of ATG7 [33].

Previous research focused on the differences between LUAD cells and the adjacent normal cells based on known transcripts and genes to explore the cause and treatment options of tumorigenesis, tumor metastasis, relapse, and drug resistance. However, identification of unknown ncRNA sets and the associated molecular mechanism between LSCs and LUAD tumor cells (LTCs) is necessary to provide insight into the biological properties of LUAD.

In this study, the identification of unknown biomarkers and a meta-analysis of unknown ncRNAs and mRNAs between LSCs and LTCs were conducted to facilitate in the elucidation of the molecular events associated with tumorigenesis, metastasis, relapse, and drug resistance from the perspective of LSCs.

## 2. Materials and Methods

### 2.1. RNA Isolation and miRNA Sequencing

Two ALDH + LSC and two ALDH- LTC samples were obtained from our previous study [11]. A total of ~3–5 × 10^6^ cells were used for the isolation of RNA. Total RNA of cellular samples was extracted using TRIzol™ reagent (Invitrogen, Carlsbad, CA, USA) based on the manufacturer's protocols. At least 3 *μ*g RNA per sample was used as a substrate for the subsequent analyses. Sequencing libraries were generated using the NEBNext® Multiplex Small RNA Library Prep Set for Illumina® (NEB, USA), according to the manufacturer's recommendations. Index codes were added to the attribute sequences of each sample. Briefly, the NEB 3′ sequence replication (SR) adaptor was directly and specifically linked to the 3′ end of miRNA, small interfering RNA (siRNA), and piwi-interacting (piRNA). After the 3′ ligation reaction, the SR random primer (RT) was hybridized to the excess of the 3′ SR adaptor, which was transformed from a single-stranded DNA adaptor into a double-stranded DNA molecule. This important step prevented adaptor-dimer formation. Thus, dsDNAs were not ligation-mediated substrates for T4 RNA Ligase 1 and therefore did not ligate to the 5′ SR adaptor in the subsequent ligation step. The 5′ end adapters were ligated to the 5′ ends of miRNA, siRNA, and piRNA. First-strand cDNA was then synthesized using M-MuLV reverse transcriptase (RNase H-). PCR amplification was performed using LongAmp® Taq 2X Master Mix, SR Primer for Illumina, and index primer (X).

The PCR products were purified on an 8% polyacrylamide gel (100 V, 80 min). DNA fragments corresponding to 140–160 bp (the length of small non-coding RNA plus the 3′ and 5′ adaptors) were recovered and dissolved in 8 *μ*L elution buffer. Library quality was evaluated on the Agilent Bioanalyzer 2100 system using DNA High Sensitivity Chips. Clustering of the index-coded samples was performed on a cBot Cluster Generation System using the TruSeq SR Cluster Kit v3-cBot-HS (Illumina) according to the manufacturer's instructions. After cluster generation, library preparations were sequenced on an Illumina Hiseq 2500/2000 platform, and 50-bp single-end reads were generated.

### 2.2. RNA Raw Data Quality Control and Reads Mapping Statistics

Two LSC samples and two LTC samples were obtained from our previous study [11]. Raw reads of miRNAs and lncRNAs/circRNAs/mRNAs from the Illumina Hiseq platform were conducted using ReSeqTools [34] to remove reads with poly-N (ratio of N greater than 10%)/adapter sequence and low-quality reads (reads with more than 50% bases having quality *Q* value ≤ 5). Raw reads of miRNAs were then processed to filter reads containing poly A/T/G/C regions. Clean reads from each miRNA sample were mapped to the human reference genome (version GRCh38) using Bowtie [35], with no mismatches. Clean reads of lncRNAs/circRNAs/mRNAs were aligned with the same human reference genome mentioned above using HISAT2 with “-rna-standness RF” and other default parameters [36]. The mapping results were sorted, analyzed, and indexed using ReSeqTools [34] and Samtools [37].

### 2.3. Identification of miRNAs

Based on the aligned small RNA reads, miRbase 20.0 [38] was used as a reference, and miRDeep2/srna-tools-cli [39] were used to identify potential miRNAs and to define the secondary structures. Based on RepeatMasker [40], the Rfam database [41] mapped reads originating from protein-coding genes, repeat sequences, rRNA, tRNA, snRNA, and snoRNA [42] were removed. Based on the characteristics of the hairpin structure of the miRNA precursor, the Dicer cleavage site, and the minimum information of the small RNA reads not annotated in the above steps, miREvo [43] and miRDeep2 [39] were integrated to predict novel miRNAs, and the base bias was counted at the first position and at all positions of all identified miRNAs. For alignment and annotation, the diversity RNA was counted with the following priority rule: known miRNA > rRNA > tRNA > snRNA > snoRNA > repeat > mRNA > novel miRNA, to ensure each unique small RNA to be matched to only one annotation. miRNA expression levels were estimated as transcripts per million (TPM) [44] using StringTie [45], whose equation is normalized to the expression level:

(1) $$\begin{array}{c} \text{TPMi}=\left(\text{Ni}/\text{Li}\right)*1,000,000/\text{sum }\left(\text{Ni}/\text{Li}+\cdots \cdots ..+\text{Nm}/\text{Lm}\right). \end{array}$$

In the above formula, *Ni* indicates the mapped reads in exon i. *Li* indicates the length of exon i. *Nm* indicates the mapped reads in exon m. *Lm* indicates the length of exon m.

### 2.4. Identification and Quantification of lncRNAs/circRNAs and mRNAs

**Figure 1:**
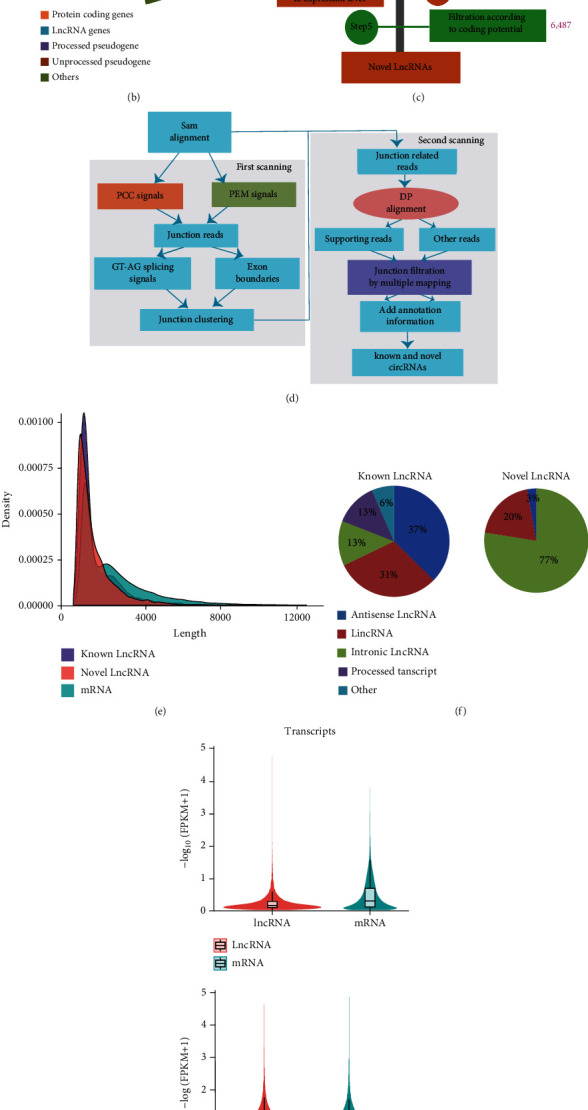
Distribution of novel and known lncRNAs/circRNAs and DE lncRNAs/circRNAs, mRNAs. (a) Sample correlation; (b) transcript classes; and (c) in silico pipeline used for the identification of novel lncRNAs. The purple number next to the bar is the remaining count of lncRNAs after filtering each step; (d) bioinformatics pipeline used for the identification of circRNAs; (e) FPKM density of all transcripts; (f) composition of known and novel lncRNAs; (g) violin plot of transcripts between lncRNA and mRNA and group LTCs and group LSCs; (h) counts of lncRNA and circRNA; (i) composition of circRNAs; and (j) Venn diagram showing predicted circRNAs in LTCs and LSCs, respectively.

The total mapped reads for each sample were assembled by StringTie [45] using a reference-based approach with an optional de novo assembly step, and a comprehensive annotation file with full-length transcripts and potential novel ncRNAs was generated. Transcripts with low confidence (exon numbers < 2), length  ≤ 200 nt, repeat annotations, and low expression (FPKM < 0.05) were removed by the filtering step in Figure 1(c). Quantification of known ncRNAs and mRNAs was performed by StringTie for each sample. Four tools, including CNCI (coding-noncoding index) [46], CPC (coding potential calculator) [47], Pfam-scan [48], and PhyloCSF (phylogenetic codon substitution frequency) [49] with default parameters, were used to predict the coding potential of transcripts, which were filtered and became candidate sets of novel lncRNAs. Cufflinks were used to calculate fragments per kilobase of exon model per million mapped fragments (FPKM) [50] of both the lncRNAs and the coding genes in each sample. FPKMs were defined as the total mapped exon fragments/(mapped reads (millions)∗exon length (KB).

The BAM files mentioned above were used to identify circRNAs. The detection and identification of circRNAs was based on our previous studies [11]. TMP was used to quantify the circle RNAs.

### 2.5. Differential Expression Analysis

After quantification of digital transcript expression, a differential expression analysis of lncRNAs and mRNAs between the LSC and LTC groups was performed using Cuffdiff [51], which was based on the negative binomial distribution model. mRNA gene transcripts with a *P* adjust < 0.05 and |log2 *foldchange*| ≥ 2 were assigned as differentially expressed (DE) genes.

DE analysis of miRNA and circRNAs between two conditions was performed using the DESeq2 R package [52]. *P* values were adjusted using the Benjamini and Hochberg method to control the false discovery rate [53]. Corrected *P* value < 0.05 and |log2 *foldchange*| ≥ 2 were established as the threshold for significant DE.

### 2.6. Kyoto Encyclopedia of Genes and Genomes Analysis and Gene Ontology Analysis on Differentially Expressed mRNAs

The Kyoto Encyclopedia of Genes and Genomes (KEGG) pathway analysis of DE mRNA was conducted using DAVID 6.8 [54] with default parameters to ascertain the potential functions of genes that participate in cancer progression of LSCs and LTCs, separately. Gene ontology (GO) analysis was performed based on Metascape 3.5 websites [55], which organized genes into hierarchical categories and uncovered the gene regulatory network using the database of the most recent biological processes. The *P* value was adjusted using the Benjamini and Hochberg method to control the false discovery rate and to identify significant KEGG pathways and GO terms.

### 2.7. Analysis of the CeRNA Network and Survival Analysis

LncRNA-mRNA co-location networks were predicted and performed based on the parameters of the upstream and downstream 100 kb distance of DE lncRNAs.

Prediction of miRNA target gene was performed on miRanda [56], PITA [57], and RNAhybrid [58], separately. The overlapping results of miRNAs and mRNAs pairs using the three software algorithms provided the final pairings. Concurrently, miRNA target prediction of lncRNAs/circRNAs was performed by miRanda.

The ceRNA networks were constructed using Cytoscape 3.9.0 [59], and the hub modules in the ceRNA networks were identified using MCODE 2.0.0 [60], a Cytoscape 3.9.0 plug-in, with default parameters. Each molecule of those hub modules was identified using the UALCAN website and the lnCAR website to obtain known hub molecules.

According to the location of the novel molecules and using the gene symbols of known molecules, survival analysis was performed using the UALCAN website [61] and the lnCAR website [62] with default parameters.

## 3. Results

### 3.1. Identification of Novel and Known miRNAs and Differentially Expressed miRNAs in LSCs and LTCs

**Figure 2:**
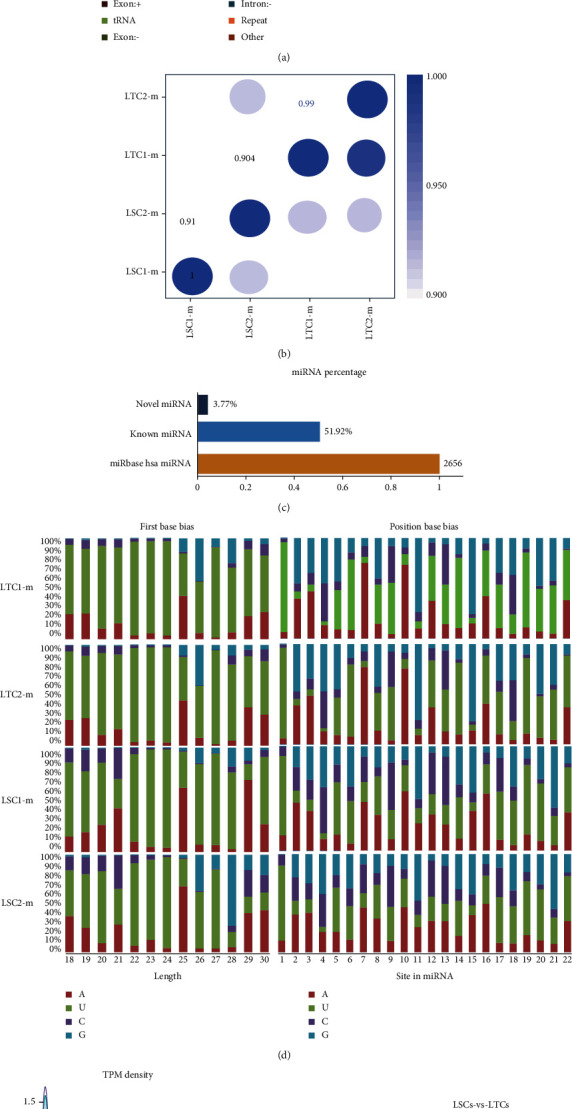
Distribution of novel and known miRNA and DE miRNAs. (a) small RNA (sRNA) category ratio; (b) sample correlation; (c) miRNA percentage from the miRbase database of known miRNAs; (d) first base bias and position base bias on known miRNA; (e) TMP density of all miRNAs; (f) Venn diagram on predicted miRNA in LSCs and LTCs; and (g) volcano plot of DE miRNAs.

Two LSC samples (ALDH+ H1975 LUAD tumor cells (LSC1-m) and ALDH+ H1975 LUAD tumor cells (LSC2-m)) and two LTC samples (ALDH-H1975 LUAD tumor cell (LTC1-m) and ALDH-H1975 LUAD tumor cells (LTC2-m)) were obtained as described in our previous study [11]. Raw miRNA data were obtained using the Illumina Hiseq 2500/2000 platform, and 50-bp single-end reads were generated. Sequencing reads of small RNA tags (~18–30 nt) extracted from raw reads were 16.62 Mb (LTC1-m), 17.93 Mb (LTC2-m), 15.58 Mb (LSC1-m), and 11.82 Mb (LSC2-m), respectively. The correlation between these four samples is shown in Figure 2(b). MiRNAs expression landscape analysis on sequencing reads was performed to dissect the distribution of ncRNAs, to predict novel miRNAs, also known as miRNAs and DE miRNAs. Of these, at least 91.61% of the total small RNA tags were perfectly mapped to the Hg38.94 genome in the four tumor samples. Interestingly, more than 55.74% of the small RNA tags in LTC2-m were mapped to known miRNAs, and only 0.01% of those small RNA tags were predicted to be novel miRNAs. In contrast, the percentage of known miRNAs in LSC2-m was only 32.5%, while the percentage of novel miRNAs was only 0.02%. The remaining small RNA tags were assigned to rRNA, tRNA, snoRNA, and exons (Supplementary Table S1.1). The most abundant class of small RNA tags was miRNAs, followed by other (Figure 2(a)). Overall, 100 new miRNAs and 1379 known miRNAs were obtained (Figure 2(c)). The base bias at the first position (based on 22 nt) and all the positions of the identified miRNAs (~18–30 nt) were similar in the new miRNAs and the known miRNAs (Figure 2(d); Figure S1). These predicted novel miRNAs and known miRNAs (Figure 2(e); Table S1.3) were used for the subsequent analysis. The overview of the study flow is shown in Figure 2 and Supplementary Figure S1.

A total of 954 common miRNAs were obtained in the two groups (LSCs contained LSC1-m/LSC2-m and the LTC group consisted of LTC1-m/LTC2-m). A total of 208 specific miRNAs were expressed in the LSC group, and 196 specific miRNAs were expressed in the LTC group (Figure 2(f)). Of these, 53 upregulated miRNAs and 27 downregulated miRNAs were identified (Figure 2(g)).

### 3.2. Identification of Novel and Known lncRNAs/circRNAs and DE lncRNAs/circRNAs, mRNA Identified in LSCs and LTCs

Raw data relative to lncRNAs/circRNAs/mRNAs were retrieved from our previous study [11]. Reads were sequenced using the Illumina Hiseq2500 platform as 125 bp pair-end reads. The correlation between the four samples evaluated is shown in Figure 1(a). Approximately 79.3 Mb pair-end reads were obtained, 93.45% of which could be matched to the Hg38.94 genome. Subsequently, the distribution of known transcripts was identified using HTseq software. Approximately 79.64% of the reads were in protein-coding genes, and 4.12% was known lncRNAs for a total of 3091 known lncRNAs (Figures 1(b) and 1(h)). Meanwhile, 188,945 new transcripts were assembled using HISAT2 and StringTie. Based on the assembled transcripts and known transcripts, a total of 6487 novel lncRNAs were identified using a specific bioinformatics pipeline A (Figures 1(c) and 1(h); Methods 2.4). The most abundant size class of known lncRNAs consisted of antisense lncRNAs (37%) and lincRNAs (31%), and the novel lncRNAs were intronic lncRNAs (77%) (Figure 1(f)). In addition, from these short reads, 681 known circRNAs and 173 novel circRNAs (Figure 1(h)) were identified using the in-silico pipeline B (Figure 1(d); Methods 2.4). Most of the circRNAs were located in exon regions (Figure 1(i)). Of these, 630 circRNAs were identified (Figure 1(j)) in two groups. A total of 155 circRNAs were only expressed in LTCs, while 68 circRNAs were only expressed in LSCs.

### 3.3. Differential Expression Analysis and Annotation Analysis

Quantitative analysis on mRNAs/lncRNAs was performed using Cufflinks software (Figures 1(e) and 1(g)), and Cuffdiff was used for DE analysis on lncRNAs, yielding 16 upregulated lncRNAs, 18 downregulated lncRNAs, 378 upregulated mRNAs, and 364 downregulated mRNAs (Padj < 0.05 and |log2 (*Fold* *Change*)| ≥ 2). Meanwhile, DE analysis was conducted on circRNAs using DESeq2 software, and 54 upregulated circRNAs and 60 downregulated circRNAs (Padj < 0.05 and |log2 (*Fold* *Change*)| ≥ 2) were obtained.

**Figure 3:**
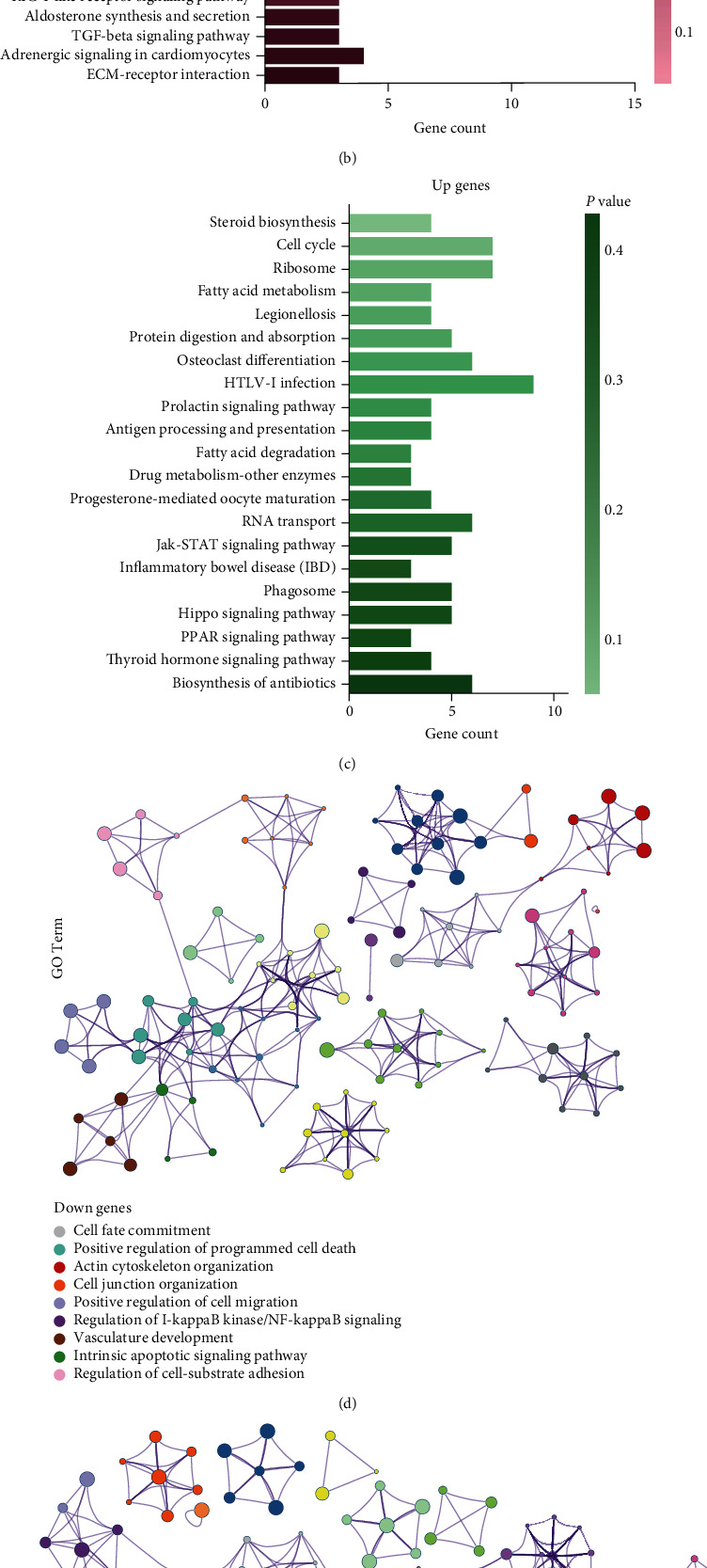
KEGG pathway analysis and GO cluster analysis. (a) Common pathway found in both upregulated gene sets and downregulated gene sets; (b) specific Kegg pathways in downregulated gene sets; (c) specific KEGG pathways in upregulated gene sets; (d) clusters of GO terms in downregulated gene sets; and (e) clusters of GO terms in downregulated gene sets.

KEGG analysis was performed to identify the potential molecular pathways that regulate LSCs and LTCs. Upregulated gene sets and downregulated gene sets were analyzed separately. The results of KEGG analysis identified common pathways (Figure 3(a)) in LTCs and LSCs, which showed the commonality between LSCs and LTCs, such as pathways in cancer, the MAPK signaling pathway, and the PI3K-Akt signaling pathway. KEGG analysis of upregulated gene sets (Figure 3(c)) included pathways, such as steroid biosynthesis, fatty acid metabolism, antigen processing and presentation, fatty acid degradation, inflammatory bowel disease (IBD), biosynthesis of antibiotics, HTLV-I infection [63], and drug metabolism-other enzymes, which may be related to immune evasion [64] and poor prognosis [63] in LUAD. Other pathways (Figure 3(b)), which were enriched by upregulated gene sets included cell cycle, osteoclast differentiation, progesterone-mediated oocyte maturation [65], and hippo signaling pathway, and these may be involved in cancer proliferation and cancer cell heterogeneity [66].

With screening criteria using the *P* value < 0.01, GO analysis was conducted on upregulated DE mRNAs and downregulated DE mRNAs, separately; then, hierarchical clustering on GO analysis was performed. Nine clusters of GO terms were found based on downregulated gene sets, and 12 clusters of GO terms were found based on upregulated gene sets (Figures 3(d) and 3(e)). The nine clusters included cell fate commitment, positive regulation of programmed cell death, intrinsic apoptotic signaling pathway, cell junction organization, actin cytoskeleton organization, regulation of cell-substrate adhesion [67], positive regulation of cell migration, and regulation of I-kappaB kinase/NF-kappaB signaling [68], which may play a vital role in cell migration, cell apoptosis, and cell communication as described in previous studies [69]. Clusters of GO terms based on upregulated gene sets were enriched in the mitotic cell cycle, DNA replication initiation, telomere maintenance, regulation of cell morphogenesis, homeostasis of the number of cells, cell morphogenesis involved in differentiation, cell junction organization, positive regulation of alpha-beta T cell activation, and steroid biosynthetic process, which may be linked to cancer growth, cancer proliferation, metastasis, and immune evasion.

### 3.4. Target Prediction and Integrative Analysis of Competitive Endogenous RNA (ceRNA) Networks

**Figure 4:**
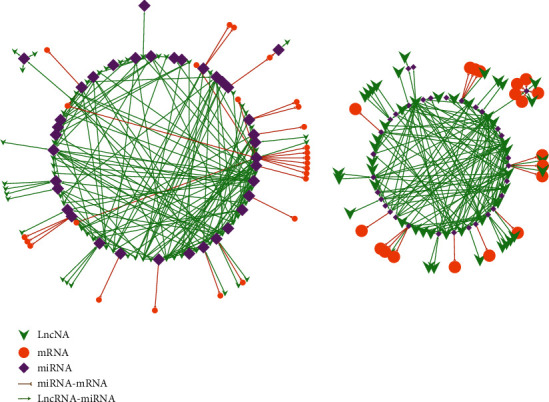
Constructed ceRNA networks. The left subnetwork consisted of upregulated miRNAs, downregulated mRNAs, and downregulated lncRNA/circRNAs; the right subnetwork consisted of downregulated miRNAs, upregulated mRNAs, and upregulated lncRNA/circRNAs. Green arrow, lncRNA/circRNA; orange circle, mRNA; purple rhombus, miRNAs; combination of brown line and semicircle, inhibition or degradation, respectively; light green line or arrow, promotion. A large or small shape (green arrow/orange circle/purple rhombus) indicates upregulation or downregulation, respectively.

Of the 742 DE mRNAs and 80 DE miRNAs, the target mRNAs of miRNAs were predicted using miRana, PITA, and RNAhybrid, separately. The overlapping results in the three software algorithms represented the final results. Of the 32 DE lncRNAs, 115 DE circRNAs, and 80 DE miRNAs, miRanda was used to search for the target lncRNAs/circRNAs of miRNAs. In addition, target mRNAs of lncRNAs were searched by scanning coding genes 10 k/100 k upstream and downstream of lncRNAs, and 12 lncRNA-mRNA pairs were obtained. Finally, ceRNA networks were constructed with 420 pairs, which consisted of 90 DE mRNAs, 80 DE miRNAs, and 32 DE lncRNAs/96 DE circRNAs (Table S5.1: ceRNA-pairs; Table S5.2: ceRNA-node-information). The 80 DE miRNAs included 78 known miRNAs and 2 novel miRNAs; the 32 DE lncRNAs included 8 known lncRNAs and 24 novel lncRNAs; and the 96 DE circRNAs included 73 known circRNAs and 32 novel circRNAs (Table S5.2: ceRNA-nodes-information). The 420 pairs included 363 pairs comprising known molecules. The remaining pairs mostly involved novel molecules. Based on the StarBase v3.0 [70] website, 280 pairs were identified, which contained most of the known ncRNAs and mRNAs that have been described previously. Interestingly, based on the ceRNA networks constructed using Cytoscape, we determined that the network could be divided into two subpopulations (Figure 4) yielding two subnetworks that satisfied the ceRNA theory. That is, the right subnetwork in Figure 4 included downregulated miRNAs, upregulated mRNAs, and lncRNA/circRNAs, while the subnetwork on the left one consisted of upregulated miRNAs, downregulated mRNAs, and lncRNA/circRNAs.

**Figure 5:**
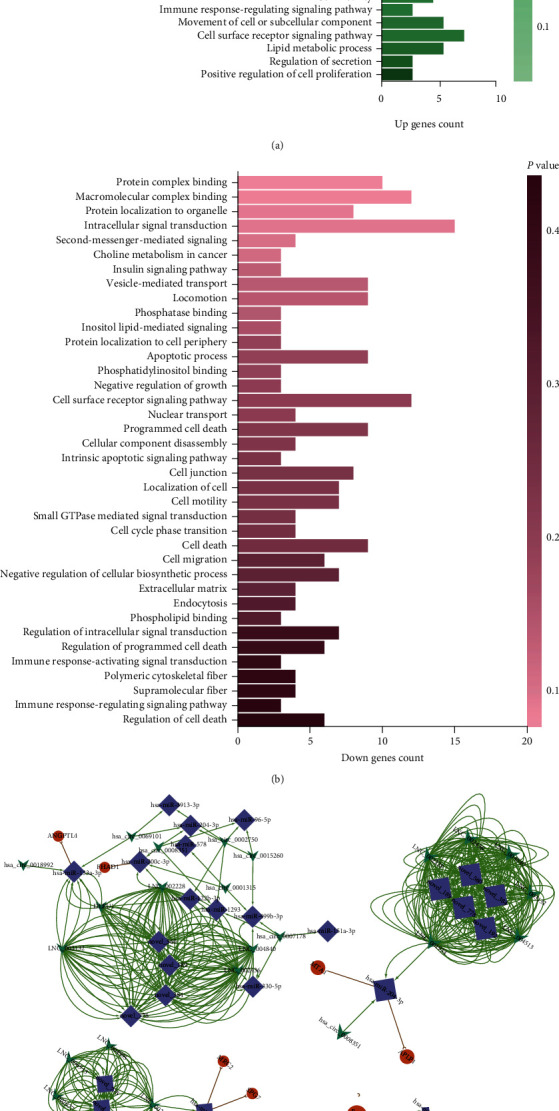
GO analysis and MCODE analysis. (a) GO analysis of upregulated gene sets comprising the ceRNA networks; (b) GO analysis of downregulated gene sets comprising the ceRNA networks; (c) Four main subnetworks; (d) Heatmap of 11 transcripts in the four subnetworks.

To reveal different pathways based on DE mRNAs in the ceRNA network of LSCs and LTs, GO analysis was performed on 33 upregulated genes and 54 downregulated genes. The results are shown in Figures 5(a) and 5(b). The GO terms of downregulated gene sets clustered in cell junction, localization of cell, cell motility, cell migration, locomotion, intracellular signal transduction, endocytosis, and extracellular matrix terms. These enriched GO terms were similar to those of upregulated gene sets, including cell adhesion, positive regulation of signaling transduction, vesicle-mediated transport, lysosome, regulation of secretion, cell migration, locomotion, localization of cell, and cell motility. The remaining GO terms in the upregulated gene sets were cell growth, cell differentiation, cell junction assembly, cell morphogenesis, and cell proliferation [71].

Next, the hub modules in ceRNA networks were identified using the Cytoscape plug-in: MCODE with default parameters. Four main hub modules were obtained as shown in Figure 5(c).

**Figure 6:**
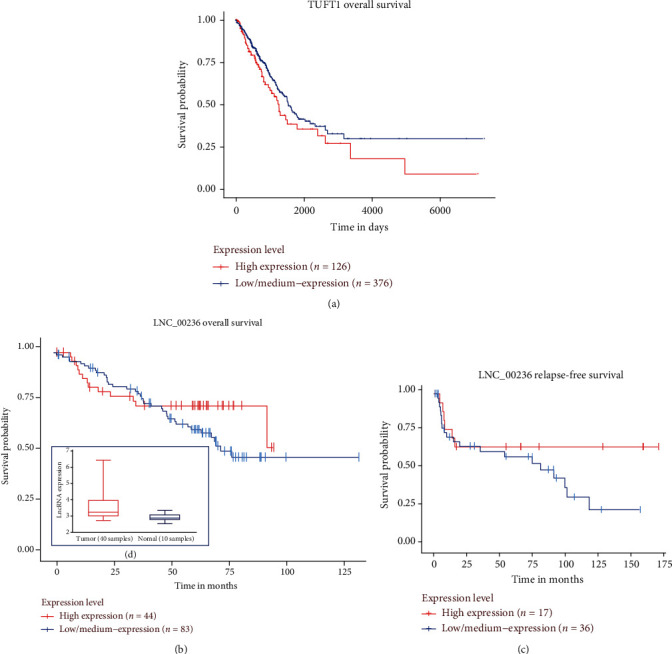
Survival analysis of patients with LUAD. Overall survival analysis stratifying patients by median expression of (a) TUFT1 and (b) LNC_00236; (c) relapse-free survival analysis of patients stratified by LNC_00236 expression; and (d) LNC_00236 expression atlas in tumor and normal tissue.

A clustering heat map was developed for all 11 mRNAs in the four modules (Figure 5(d)). Among these genes, two S100A8 transcripts and one S100A9 transcript were significantly upregulated and all were regulated by the same two novel lncRNAs (LNC_000305 and LNC_000605). We then listed all genes/RNAs in the four hub subnetworks based on the results of the Cytoscape plug-in MCODE. According to the location of the novel molecules and using the gene symbols of known molecules, a survival analysis was performed including all the molecules obtained in the four hub modules in lung adenocarcinoma patients using the UALCAN website and the lnCAR website. Then, based on the survival analysis from the UALCAN website and the lnCAR website, the survival curves of a novel lncRNA and the mRNAs, LNC_00236, and TUFT1, which belonged to the same submodule, were constructed (Figures 5(c) and 6(a)–6(c)). The rest of the molecules from the same submodule are unknown, according to the UALCAN website and the lnCAR website. The three curves revealed that expression of TUFT1 and LNC_00236 was associated with the overall survival of patients with LUAD. In addition, LNC_00236 was highly expressed in 40 patients with LUAD and may represent a potential prognostic biomarker. Furthermore, the remaining novel lncRNAs may represent a potential resource for future research into prognostic biomarkers (Figure 6(d)).

## 4. Conclusions

Our study identified novel miRNAs, lncRNAs, and circRNAs, which represent an essential and crucial resource in understanding the underlying biology of LUAD. Specifically, the study provided a detailed dissection of the constituents and functional properties of differentially expressed miRNAs, mRNAs, lncRNAs, and circRNAs between ALDH+ H1975 LSCs and ALDH- H1975 LTCs. Based on these data, an interaction network of differentially expressed miRNAs, mRNAs, and lncRNAs/circRNAs was constructed to investigate the potential novel regulatory mechanism. The findings of this meta-analysis provide comprehensive and systematic information on the underlying hallmarks distinguishing LSCs and LTCs and the pathogenesis of LUAD from the perspective of lung CSCs. This study also provides a rationale for further experimental validation of these regulatory mechanisms and their role in the prognosis of LUAD and as therapeutic biomarkers of LUAD.

## 5. Discussion

CSCs, as a rare cell population of cancers, display a broad spectrum of functional heterogeneity [12]. They exhibit similar properties as normal stem cells, such as self-renewal, asymmetric cell division, and evasion of apoptosis [14], which enable them to initiate cancers, promote cancer growth, metastasis, and relapse [6]. Experimental evidence from previous studies [10] and our most recent study [11] indicated that ALDH+ H1975 LSCs are the key CSCs that proliferate extensively and form new tumors, while the remaining cancer cells lack this ability. The activities of LSCs are diverse [15]. For example, exosomes from LSCs are associated with cell proliferation, migration, adhesion, and cell-cell communication [11]. Furthermore, several pathways are associated with cell cycle kinetics, DNA repair, and mRNA expression of multidrug-resistance genes in CSCs that promote oncogenic resistance to chemotherapy and drugs [72]. In addition, steroid biosynthesis of T cells induced from the microenvironment in CSCs and resident cancer cells may lead to immune evasion [64]. The above functions of LSCs may be broadly regulated by ncRNAs, such as miRNAs [20], lncRNAs, and circRNAs [26]. Furthermore, identification of known and unknown biomarkers of ncRNAs and their systematic and comprehensive interrelation with unknown ncRNAs and mRNAs may also be necessary to provide insights into the function of LSCs. Herein, we provide, for the first time, the ncRNA expression profile, including known and unknown miRNAs, lncRNAs, and circRNAs of LSCs and LTCs.

The gene expression profile of LSCs reflects the properties of LSCs that drive tumorigenesis. The regulatory mechanisms of mRNAs are associated with ncRNAs [26]. Therefore, a meta-analysis on ncRNAs and their relative regulated mRNAs is needed to enhance understanding of the underlying regulatory mechanisms of LSCs. In the current study, the detailed constituents and functional properties of LSCs were dissected from ceRNAs, GO enrichment, and KEGG pathway analyses. GO enrichment based on all DE mRNAs confirmed that (a) clusters of GO terms based on upregulated gene sets were closely related to processes such as DNA repair, the mitotic cell cycle, cell morphogenesis involved in differentiation, cell junction, positive regulation of alpha-beta T cell activation, and steroid biosynthesis. These pathways were highly correlated with the function of LSCs in oncogenic resistance to chemotherapy and drugs [72] and immune evasion [64]. In addition, (b) clusters of GO terms based on downregulated gene sets were closely related to the commitment of cell fate, the positive regulation of programmed cell death, and cell adhesion. These pathways may play a vital role in cell migration and cell apoptosis. Likewise, LSCs and LTCs are cancer cells. Thus, they have the traits of cancer cells and may share common cancer-related signaling pathways, which is comparable with our results in the KEGG pathway analyses based on all DE mRNAs.

In ceRNAs and relative GO enrichment analysis, two predominant properties were uncovered. First, the GO terms of downregulated gene sets are clustered in cell-cell communication, such as cell junction, localization of the cell, cell motility, intracellular signal transduction, endocytosis, and the extracellular matrix, which are mechanisms that may be linked to cell communication, the tumor microenvironment, exosomes, and cancer metastasis [73]. These GO terms were similar to our previous study, that is, communication between cells, such as LSCs-LTCs and LTCs-LTCs, and they may depend on exosomes secreted by neighboring cells, which ultimately lead to altered cell functions, such as cell migration and proliferation of cancer cells. Second, the GO terms of upregulated gene sets also clustered in fatty acid-binding, positive regulation of the defense response, Ras protein signal transduction, the defense response, and the innate immune response. These GO terms may influence the immune resistance of LSCs, such as immune evasion, and may eventually be associated with a poor prognosis, as suggested above. The remaining GO terms in the upregulated gene sets involved pathways such as cell growth, cell differentiation, cell junction assembly, cell morphogenesis, and cell proliferation, which are closely related to the stemness of LSCs [73]. The ncRNAs associated with these terms and pathways may play a vital role in regulating the relative expression of mRNAs. These findings will provide a guide for future experimental verification of key ncRNAs and the identification of efficient biomarkers for cancer therapy and diagnosis. The three mRNAs (two S100A8 transcripts and one S100A9 transcript) from MCODE analysis were associated with several regulatory functions, such as regulation of defense response, regulation of NF-kappaB transcription factor (TF) activity, and the Toll-like receptor signaling pathway. Furthermore, S100A9 was correlated with a poor prognosis in LUAD [11].

In summary, the novel ncRNA transcripts and the overall profile-based meta-analysis of our study provide a comprehensive description of the biological molecular composition and properties of LUAD CSCs. We have dissected what is known and unknown about LSCs to provide a therapeutic perspective on these findings.

## Data Availability

The data that support the findings of this study are openly available in the Sequence Read Archive of NCBI at https://dataview.ncbi.nlm.nih.gov/object/PRJNA675697, reference number PRJNA675697, that contains the sequencing reads of all replicates in the fastq format.

## Abbreviations

ncRNAs: Noncoding RNAs
CSCs: Cancer stem cells
miRNAs: Micro RNAs
lncRNAs: Long noncoding RNAs
circRNAs: Circular RNAs
mRNAs: Messenger RNAs
LSCs: Lung adenocarcinoma ALDH+ H1975 stem cells
LTCs: Lung adenocarcinoma ALDH- H1975 tumor cells
LUAD: Lung adenocarcinoma
ceRNA: Competitive endogenous RNA
NSCLC: Non-small cell lung cancer
UTR: Untranslated region
TMP: Transcripts per million
FPKM: Fragments per kilobase of exon model per million mapped fragments
PCC: Pair chiastic clipping
PEM: Pair end mapping
DP: Dynamic programming alignment
KEGG: Kyoto encyclopedia of gene and genome
GO: Gene ontology
Padj: Adjusted *P* value.

## Additional Points

*Note*. A preprint has previously been published [1].

## Supplementary Materials

Supplementary Materials The supporting information includes the following: Figure S1. First base bias and position base bias of novel miRNAs; Table S1. Detailed information of miRNAs; Table S2. Novel lncRNAs; Table S3. Novel miRNAs; Table S4. Novel circRNAs; Table S5. Information on ceRNA-based molecular interactions and pairs.
