# Supplementary figures and images for: Identification of Novel Multi-Omics Expression Landscapes and Meta-Analysis of Landscape-Based Competitive Endogenous RNA Networks in ALDH+ Lung Adenocarcinoma Stem Cells
Source: Biomed Res Int. 2022 Aug 31;2022:9545609. doi: 10.1155/2022/9545609 (PMC9453044; doi:10.1155/2022/9545609)

# Novel miRNA

First Base Bias

Position Base Bias

■ A ■ U ■ C ■ G

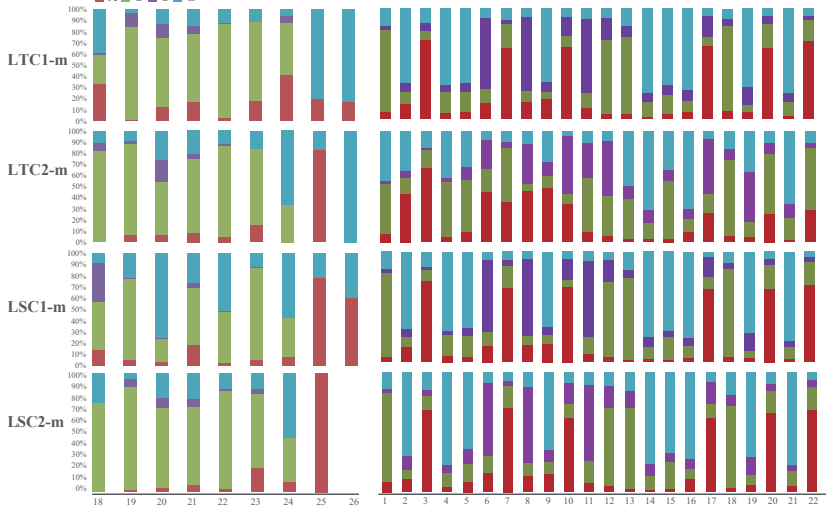

Supplement: Supplementary Materials — The supporting information includes the following: Figure S1. First base bias and position base bias of novel miRNAs; Table S1. Detailed information of miRNAs; Table S2. Novel lncRNAs; Table S3. Novel miRNAs; Table S4. Novel circRNAs; Table S5. Information on ceRNA-based molecular interactions and pairs. [file 9545609.f1.zip › Figure S1 novel miRNAs-base bias.pdf]
