# Supplementary material for: Identification of Novel Multi-Omics Expression Landscapes and Meta-Analysis of Landscape-Based Competitive Endogenous RNA Networks in ALDH+ Lung Adenocarcinoma Stem Cells
Source: Biomed Res Int. 2022 Aug 31;2022:9545609. doi: 10.1155/2022/9545609 (PMC9453044; doi:10.1155/2022/9545609)
Supplement: Supplementary Materials — The supporting information includes the following: Figure S1. First base bias and position base bias of novel miRNAs; Table S1. Detailed information of miRNAs; Table S2. Novel lncRNAs; Table S3. Novel miRNAs; Table S4. Novel circRNAs; Table S5. Information on ceRNA-based molecular interactions and pairs. [file 9545609.f1.zip › Table_S1_Detailed information of miRNAs.doc]

| **Table 1:Regions containing reads and percentage of those reads** | | | | | | | | |
| --- | --- | --- | --- | --- | --- | --- | --- | --- |
| **Types** | **SC1** | **SC1(percent)** | **SC2** | **SC2(percent)** | **TC1** | **TC1(percent)** | **TC2** | **TC2(percent)** |
| total | 14276843 | 100.00% | 10954687 | 100.00% | 15868627 | 100.00% | 17057766 | 100.00% |
| known_miRNA | 5119594 | 35.86% | 3560572 | 32.50% | 9270409 | 58.42% | 9508082 | 55.74% |
| rRNA | 168686 | 1.18% | 521365 | 4.76% | 68579 | 0.43% | 88369 | 0.52% |
| tRNA | 922088 | 6.46% | 550383 | 5.02% | 1285179 | 8.10% | 1719692 | 10.08% |
| snRNA | 5887 | 0.04% | 10168 | 0.09% | 6856 | 0.04% | 6472 | 0.04% |
| snoRNA | 621501 | 4.35% | 349569 | 3.19% | 169176 | 1.07% | 178064 | 1.04% |
| repeat | 557944 | 3.91% | 260643 | 2.38% | 427746 | 2.70% | 639696 | 3.75% |
| novel_miRNA | 1928 | 0.01% | 1722 | 0.02% | 1077 | 0.01% | 1130 | 0.01% |
| exon:+ | 649706 | 4.55% | 504667 | 4.61% | 315860 | 1.99% | 379499 | 2.22% |
| exon:- | 70938 | 0.50% | 85742 | 0.78% | 24358 | 0.15% | 33819 | 0.20% |
| intron:+ | 1602473 | 11.22% | 1307153 | 11.93% | 315370 | 1.99% | 423323 | 2.48% |
| intron:- | 70228 | 0.49% | 147203 | 1.34% | 43928 | 0.28% | 45893 | 0.27% |
| other | 4485870 | 31.42% | 3655500 | 33.37% | 3940089 | 24.83% | 4033727 | 23.65% |

| **Table 2:Pearson correlation of miRNA samples** | | | | |
| --- | --- | --- | --- | --- |
| **R^2** | **LSC1** | **LSC2** | **LTC1** | **LTC2** |
| LSC1 | 1 | 0.905 | 0.887 | 0.891 |
| LSC2 | 0.905 | 1 | 0.904 | 0.906 |
| LTC1 | 0.887 | 0.904 | 1 | 0.99 |
| LTC2 | 0.891 | 0.906 | 0.99 | 1 |

| **Table 3:Known/novel miRNAs** | | |
| --- | --- | --- |
| 2656 | 1 | **hsadatabse_miRNA** |
| 1379 | 0.519201807 | **known_miRNA** |
| 100 | 0.037650602 | **novel_miRNA** |
