# Supplementary material for: Identification of Novel Multi-Omics Expression Landscapes and Meta-Analysis of Landscape-Based Competitive Endogenous RNA Networks in ALDH+ Lung Adenocarcinoma Stem Cells
Source: Biomed Res Int. 2022 Aug 31;2022:9545609. doi: 10.1155/2022/9545609 (PMC9453044; doi:10.1155/2022/9545609)
Supplement: Supplementary Materials — The supporting information includes the following: Figure S1. First base bias and position base bias of novel miRNAs; Table S1. Detailed information of miRNAs; Table S2. Novel lncRNAs; Table S3. Novel miRNAs; Table S4. Novel circRNAs; Table S5. Information on ceRNA-based molecular interactions and pairs. [file 9545609.f1.zip › Table_S2_Novel_lncRNAs.doc]

| **Transcript_id** | **Gene_id** | **icial_Sym** | **Gene_type** | **LSC1_FPKM** | **LSC2_FPKM** | **LTC1_FPKM** | **LTC2_FPKM** |
| --- | --- | --- | --- | --- | --- | --- | --- |
| LNC_003902 | XLOC_083971 | -- | lincRNA | 16.644058 | 59.255447 | 14.640118 | 17.206306 |
| LNC_003903 | XLOC_083971 | -- | lincRNA | 29.443529 | 24.490482 | 8.214715 | 12.845423 |
| LNC_003900 | XLOC_083904 | -- | lincRNA | 0.569712 | 0.498291 | 0.483049 | 0.319883 |
| LNC_003901 | XLOC_083971 | -- | lincRNA | 0 | 12.073474 | 0 | 0 |
| LNC_003906 | XLOC_083971 | -- | lincRNA | 8.256744 | 2.892186 | 1.921601 | 4.670701 |
| LNC_003907 | XLOC_083971 | -- | lincRNA | 4.030378 | 24.159952 | 8.503959 | 8.737496 |
| LNC_003904 | XLOC_083971 | -- | lincRNA | 65.175713 | 68.040092 | 66.247879 | 74.270401 |
| LNC_003905 | XLOC_083971 | -- | lincRNA | 5.017535 | 3.54871 | 7.049674 | 4.319219 |
| LNC_003908 | XLOC_083973 | -- | lincRNA | 4.891936 | 4.633807 | 5.295711 | 3.877164 |
| LNC_003909 | XLOC_084004 | -- | lincRNA | 1.197827 | 3.347149 | 2.043422 | 2.293788 |
| LNC_000256 | XLOC_003649 | -- | intronic_lncRNA | 0.157334 | 0.247018 | 0.281184 | 0.594237 |
| LNC_000109 | XLOC_000943 | -- | intronic_lncRNA | 0 | 1.360977 | 0.090514 | 0.130407 |
| LNC_000108 | XLOC_000943 | -- | intronic_lncRNA | 0.672862 | 0.033658 | 0.766941 | 0.697389 |
| LNC_000103 | XLOC_000816 | -- | lincRNA | 0.031524 | 0.104182 | 0.475317 | 0.269686 |
| LNC_000102 | XLOC_000793 | -- | lincRNA | 0.083111 | 1.240688 | 0.098256 | 0.006038 |
| LNC_000101 | XLOC_000793 | -- | lincRNA | 0.709893 | 1.444773 | 0.546507 | 0.192996 |
| LNC_000100 | XLOC_000780 | -- | lincRNA | 0 | 0.534336 | 0 | 0 |
| LNC_000107 | XLOC_000902 | -- | intronic_lncRNA | 0.918649 | 0.732368 | 0.920879 | 1.599315 |
| LNC_000106 | XLOC_000890 | -- | intronic_lncRNA | 0.4166 | 0.252501 | 0.088571 | 0.455195 |
| LNC_000105 | XLOC_000855 | -- | lincRNA | 0 | 0.402829 | 0.648651 | 0.513813 |
| LNC_000104 | XLOC_000852 | -- | lincRNA | 0 | 0 | 0.410588 | 0.219308 |
| LNC_004275 | XLOC_092912 | -- | intronic_lncRNA | 0.429 | 0.167635 | 0.415479 | 0.296182 |
| LNC_004274 | XLOC_092908 | -- | intronic_lncRNA | 0.439429 | 0.087258 | 0.284039 | 0.410855 |
| LNC_000819 | XLOC_017259 | -- | intronic_lncRNA | 0.375555 | 0 | 0.495455 | 0.257717 |
| LNC_000818 | XLOC_017246 | -- | intronic_lncRNA | 0.095479 | 0.437402 | 0.36598 | 0.972322 |
| LNC_001432 | XLOC_031237 | -- | intronic_lncRNA | 0.698714 | 0.157607 | 0.606287 | 0.50764 |
| LNC_001433 | XLOC_031238 | -- | intronic_lncRNA | 0.238601 | 0.937695 | 0.239319 | 0.46773 |
| LNC_001434 | XLOC_031242 | -- | intronic_lncRNA | 0 | 0 | 0.910668 | 0 |
| LNC_001435 | XLOC_031242 | -- | intronic_lncRNA | 1.02096 | 0.811752 | 0.469154 | 0 |
| LNC_001436 | XLOC_031242 | -- | intronic_lncRNA | 0.147964 | 0.51725 | 0.527591 | 1.546538 |
| LNC_001437 | XLOC_031253 | -- | intronic_lncRNA | 0.787274 | 0.444026 | 0.349925 | 0.421194 |
| LNC_000811 | XLOC_017137 | -- | intronic_lncRNA | 0.653676 | 0.564196 | 1.040963 | 0.852058 |
| LNC_000810 | XLOC_017077 | -- | intronic_lncRNA | 0.359217 | 0.291737 | 0.71346 | 0.779576 |
| LNC_000813 | XLOC_017187 | -- | antisense_lncRNA | 0 | 0.954509 | 0.728342 | 0.249159 |
| LNC_000812 | XLOC_017170 | -- | intronic_lncRNA | 0.35493 | 0.261768 | 0.586095 | 0.832423 |
| LNC_000815 | XLOC_017200 | -- | intronic_lncRNA | 0.635793 | 0.22656 | 0.403415 | 0.66042 |
| LNC_000814 | XLOC_017199 | -- | intronic_lncRNA | 0.505814 | 0.063111 | 0.076667 | 0.195416 |
| LNC_000817 | XLOC_017246 | -- | intronic_lncRNA | 0.23817 | 0.467972 | 0.18338 | 0.547496 |
| LNC_000816 | XLOC_017215 | -- | intronic_lncRNA | 0.367067 | 0.520191 | 0.973612 | 0.934273 |
| LNC_006112 | XLOC_146072 | -- | intronic_lncRNA | 0 | 0 | 0 | 0.419314 |
| LNC_006113 | XLOC_146170 | -- | lincRNA | 0.134692 | 0 | 0.527516 | 0.108551 |
| LNC_006110 | XLOC_146038 | -- | intronic_lncRNA | 0.619521 | 0.628703 | 0.420828 | 0.592349 |
| LNC_004482 | XLOC_098423 | -- | intronic_lncRNA | 0.78909 | 0.13122 | 0 | 0 |
| LNC_004483 | XLOC_098425 | -- | intronic_lncRNA | 0.103169 | 0.869252 | 0.08964 | 0.078571 |
| LNC_004480 | XLOC_098380 | -- | intronic_lncRNA | 0.445615 | 0.190162 | 0.23768 | 0.59384 |
| LNC_004481 | XLOC_098400 | -- | intronic_lncRNA | 0.356068 | 0.353137 | 0.695263 | 0.626376 |
| LNC_004486 | XLOC_098432 | -- | intronic_lncRNA | 0.040524 | 0.578018 | 0.121274 | 0.783697 |
| LNC_004487 | XLOC_098438 | -- | intronic_lncRNA | 0.508033 | 0.380171 | 0.220609 | 0.735642 |
| LNC_004484 | XLOC_098426 | -- | intronic_lncRNA | 0.145688 | 0.817417 | 0 | 0 |
| LNC_004485 | XLOC_098432 | -- | intronic_lncRNA | 1.399605 | 0.965427 | 1.10308 | 1.085327 |
| LNC_006116 | XLOC_146177 | -- | intronic_lncRNA | 0.394115 | 0.953203 | 0.186466 | 0.787532 |
| LNC_004488 | XLOC_098439 | -- | intronic_lncRNA | 0 | 0 | 0.725425 | 0.261108 |
| LNC_004489 | XLOC_098451 | -- | intronic_lncRNA | 0.079966 | 0 | 0.452316 | 0.102316 |
| LNC_006117 | XLOC_146281 | -- | intronic_lncRNA | 1.087608 | 0.374547 | 1.354876 | 1.450531 |
| LNC_006035 | XLOC_143568 | -- | intronic_lncRNA | 0.587514 | 0.451542 | 0.602766 | 0.609136 |
| LNC_006114 | XLOC_146176 | -- | intronic_lncRNA | 0.568936 | 0.232239 | 0.783235 | 0.969988 |
| LNC_006034 | XLOC_143567 | -- | intronic_lncRNA | 0.645175 | 0.62485 | 0.795415 | 0.689374 |
| LNC_006115 | XLOC_146176 | -- | intronic_lncRNA | 0.187837 | 0.646117 | 0.350852 | 0.302922 |
| LNC_006037 | XLOC_143719 | -- | intronic_lncRNA | 0 | 0.869809 | 1.052423 | 0.905647 |
| LNC_006036 | XLOC_143695 | -- | intronic_lncRNA | 0.089711 | 0 | 0.079676 | 0.336739 |
| LNC_006031 | XLOC_143468 | -- | intronic_lncRNA | 0.319612 | 0.438134 | 0.628768 | 0.888764 |
| LNC_006030 | XLOC_143466 | -- | intronic_lncRNA | 1.319775 | 1.958736 | 0.916184 | 1.956391 |
| LNC_006033 | XLOC_143552 | -- | intronic_lncRNA | 0.886209 | 0.524742 | 0.666447 | 0.901274 |
| LNC_005035 | XLOC_114862 | -- | intronic_lncRNA | 0.517869 | 0.693117 | 0.407572 | 0.507172 |
| LNC_006032 | XLOC_143550 | -- | intronic_lncRNA | 0.918395 | 1.736449 | 0.412518 | 0.806666 |
| LNC_003289 | XLOC_075151 | -- | lincRNA | 3.584144 | 1.294781 | 0.541558 | 0.35679 |
| LNC_003288 | XLOC_075151 | -- | lincRNA | 0.770776 | 0.819297 | 0.344759 | 0.415489 |
| LNC_003281 | XLOC_075147 | -- | lincRNA | 1.759192 | 1.152405 | 0.095854 | 0.541253 |
| LNC_003280 | XLOC_075145 | -- | lincRNA | 0.79071 | 4.007394 | 1.407792 | 2.5967 |
| LNC_003283 | XLOC_075149 | -- | lincRNA | 1.532212 | 1.181583 | 1.694828 | 1.338266 |
| LNC_003282 | XLOC_075148 | -- | lincRNA | 2.910869 | 2.241162 | 0.23945 | 0.665901 |
| LNC_003285 | XLOC_075151 | -- | lincRNA | 3.897447 | 1.509984 | 0.640755 | 1.050936 |
| LNC_003284 | XLOC_075149 | -- | lincRNA | 5.717796 | 2.176246 | 1.308915 | 1.657011 |
| LNC_003287 | XLOC_075151 | -- | lincRNA | 3.53422 | 1.729966 | 0.246762 | 0.866869 |
| LNC_003286 | XLOC_075151 | -- | lincRNA | 1.533065 | 0.058047 | 0.255316 | 0.137942 |
| LNC_000741 | XLOC_015110 | -- | intronic_lncRNA | 0.640658 | 0.453277 | 0.656661 | 0.916121 |

| LNC_000740 | XLOC_015104 | -- | intronic_lncRNA | 0.475165 | 0.208012 | 0.216963 | 0.349821 |
| --- | --- | --- | --- | --- | --- | --- | --- |
| LNC_000743 | XLOC_015155 | -- | intronic_lncRNA | 0.551557 | 0 | 1.203589 | 0.525827 |
| LNC_000742 | XLOC_015138 | -- | intronic_lncRNA | 0 | 0 | 0 | 0.516079 |
| LNC_000745 | XLOC_015259 | -- | intronic_lncRNA | 0.456658 | 0.459513 | 0.627566 | 0.54022 |
| LNC_000744 | XLOC_015216 | -- | intronic_lncRNA | 0 | 0 | 0.689156 | 0.533409 |
| LNC_000747 | XLOC_015286 | -- | intronic_lncRNA | 0.299319 | 0.410484 | 0.116177 | 0.091368 |
| LNC_000746 | XLOC_015285 | -- | intronic_lncRNA | 0.675216 | 0.464013 | 0.522309 | 1.061866 |
| LNC_000749 | XLOC_015343 | -- | antisense_lncRNA | 0.548393 | 0 | 0 | 0 |
| LNC_000748 | XLOC_015342 | -- | intronic_lncRNA | 0.281128 | 0.166511 | 0.407265 | 0 |
| LNC_005999 | XLOC_142636 | -- | intronic_lncRNA | 0 | 0.502159 | 0 | 0 |
| LNC_005998 | XLOC_142633 | -- | intronic_lncRNA | 0.298757 | 0 | 0.524432 | 0 |
| LNC_002523 | XLOC_058479 | -- | intronic_lncRNA | 0.75373 | 1.334029 | 0.538482 | 0.754534 |
| LNC_002522 | XLOC_058466 | -- | intronic_lncRNA | 4.943318 | 3.05419 | 5.584066 | 3.710968 |
| LNC_002521 | XLOC_058466 | -- | intronic_lncRNA | 3.16588 | 3.064009 | 3.104634 | 3.55388 |
| LNC_002520 | XLOC_058462 | -- | intronic_lncRNA | 0.533515 | 0.409846 | 1.068145 | 0.940242 |
| LNC_002527 | XLOC_058541 | -- | intronic_lncRNA | 0.523125 | 0.238845 | 1.278606 | 0.269134 |
| LNC_002526 | XLOC_058539 | -- | intronic_lncRNA | 0.870557 | 0.148236 | 0.364576 | 0.622115 |
| LNC_002525 | XLOC_058502 | -- | intronic_lncRNA | 0.330769 | 0.310624 | 0.80631 | 0.377617 |
| LNC_002524 | XLOC_058480 | -- | intronic_lncRNA | 0.342132 | 0.338117 | 0.646511 | 0.619016 |
| LNC_002529 | XLOC_058593 | -- | intronic_lncRNA | 0.629636 | 0.28412 | 0.423863 | 0.826696 |
| LNC_002528 | XLOC_058562 | -- | intronic_lncRNA | 0.983154 | 1.200644 | 0.369507 | 0.563368 |
| LNC_003133 | XLOC_072257 | -- | intronic_lncRNA | 0.497625 | 0.506341 | 0.562061 | 0.871316 |
| LNC_003132 | XLOC_072248 | -- | intronic_lncRNA | 0 | 0 | 1.714566 | 5.187311 |
| LNC_003131 | XLOC_072248 | -- | intronic_lncRNA | 1.943875 | 2.536733 | 11.097886 | 8.430476 |
| LNC_003130 | XLOC_072243 | -- | intronic_lncRNA | 0.347596 | 0 | 0.26146 | 1.033687 |
| LNC_003137 | XLOC_072363 | -- | intronic_lncRNA | 0.522319 | 0.242099 | 0.498125 | 0.618318 |
| LNC_003136 | XLOC_072312 | -- | intronic_lncRNA | 0.971139 | 0.553693 | 0.464481 | 0.645604 |
| LNC_003135 | XLOC_072264 | -- | antisense_lncRNA | 0.917028 | 0.066191 | 0.19492 | 0.625142 |
| LNC_003134 | XLOC_072263 | -- | intronic_lncRNA | 0.126358 | 0 | 0.451249 | 0 |
| LNC_003139 | XLOC_072400 | -- | lincRNA | 0.821728 | 0.184474 | 0.061378 | 0.091693 |
| LNC_003138 | XLOC_072382 | -- | antisense_lncRNA | 0.400354 | 0.358377 | 0.277692 | 0.724378 |
| LNC_005199 | XLOC_118864 | -- | intronic_lncRNA | 0.152172 | 0 | 0.16696 | 0.859762 |
| LNC_005198 | XLOC_118850 | -- | intronic_lncRNA | 0.294434 | 0.461644 | 0.473791 | 0.407784 |
| LNC_005193 | XLOC_118716 | -- | intronic_lncRNA | 0.951264 | 0.311482 | 0.537999 | 0.547206 |
| LNC_005192 | XLOC_118666 | -- | intronic_lncRNA | 0.640547 | 0.079889 | 0 | 0.064688 |
| LNC_005191 | XLOC_118665 | -- | intronic_lncRNA | 0.590748 | 0.264062 | 0.281955 | 0.095454 |
| LNC_005190 | XLOC_118664 | -- | intronic_lncRNA | 0 | 0 | 0.491331 | 0 |
| LNC_005197 | XLOC_118846 | -- | intronic_lncRNA | 0.214897 | 0.320908 | 0.516308 | 0 |
| LNC_005196 | XLOC_118845 | -- | intronic_lncRNA | 0.125279 | 0.499994 | 0 | 0 |
| LNC_005195 | XLOC_118716 | -- | intronic_lncRNA | 0 | 0.637442 | 0 | 0 |
| LNC_005194 | XLOC_118716 | -- | intronic_lncRNA | 0.683443 | 0.405064 | 0.262598 | 0.820118 |
| LNC_002428 | XLOC_056132 | -- | intronic_lncRNA | 0.352281 | 0.064731 | 0.467791 | 0.491209 |
| LNC_002429 | XLOC_056209 | -- | intronic_lncRNA | 1.132384 | 1.193416 | 1.452141 | 0.943068 |
| LNC_002426 | XLOC_056089 | -- | intronic_lncRNA | 0.917836 | 0 | 0.37632 | 0 |
| LNC_002427 | XLOC_056091 | -- | intronic_lncRNA | 0.823234 | 0.199453 | 0 | 0 |
| LNC_002424 | XLOC_055973 | -- | intronic_lncRNA | 0.628361 | 0.470735 | 0.688691 | 0.59868 |
| LNC_002425 | XLOC_056069 | -- | intronic_lncRNA | 0.291665 | 0.210051 | 0.462316 | 0.350272 |
| LNC_002422 | XLOC_055938 | -- | lincRNA | 0.192429 | 0 | 1.042006 | 1.456398 |
| LNC_002423 | XLOC_055973 | -- | intronic_lncRNA | 0.862978 | 1.079684 | 0.815931 | 1.335272 |
| LNC_002420 | XLOC_055936 | -- | intronic_lncRNA | 3.856138 | 2.290694 | 4.834871 | 5.55436 |
| LNC_002421 | XLOC_055937 | -- | intronic_lncRNA | 0 | 0 | 0.281077 | 0.793744 |
| LNC_005429 | XLOC_125321 | -- | intronic_lncRNA | 0.396926 | 0.398897 | 0.834149 | 0.940439 |
| LNC_005428 | XLOC_125267 | -- | intronic_lncRNA | 0.556797 | 0 | 0 | 0 |
| LNC_005423 | XLOC_125164 | -- | intronic_lncRNA | 0.536889 | 0.611399 | 0.867464 | 0.781151 |
| LNC_005422 | XLOC_125145 | -- | intronic_lncRNA | 0.050076 | 0.016394 | 0.618161 | 0.063705 |
| LNC_005421 | XLOC_125142 | -- | intronic_lncRNA | 0.309008 | 0.134918 | 0.798745 | 0.688706 |
| LNC_005420 | XLOC_125101 | -- | intronic_lncRNA | 0.471876 | 0.212806 | 0.950535 | 0.786748 |
| LNC_005427 | XLOC_125259 | -- | intronic_lncRNA | 0.458063 | 0.484316 | 1.483741 | 0.371352 |
| LNC_005426 | XLOC_125223 | -- | intronic_lncRNA | 3.234786 | 4.094971 | 1.953296 | 4.058622 |
| LNC_005425 | XLOC_125203 | -- | intronic_lncRNA | 0.811565 | 0.525597 | 0.506078 | 0.607592 |
| LNC_005424 | XLOC_125172 | -- | intronic_lncRNA | 0.245655 | 0.195379 | 1.116961 | 0.605281 |
| LNC_004248 | XLOC_092122 | -- | intronic_lncRNA | 0.537177 | 0.479114 | 0.5563 | 1.11027 |
| LNC_004249 | XLOC_092152 | -- | lincRNA | 0.078305 | 0.113588 | 0.716526 | 0.175407 |
| LNC_004240 | XLOC_091856 | -- | intronic_lncRNA | 0 | 0.600225 | 0.682752 | 0.657019 |
| LNC_004241 | XLOC_091878 | -- | intronic_lncRNA | 0.358008 | 0.703022 | 0.662796 | 0.574201 |
| LNC_004242 | XLOC_091879 | -- | intronic_lncRNA | 0.255185 | 0.095808 | 0.544749 | 0.127429 |
| LNC_004243 | XLOC_091888 | -- | intronic_lncRNA | 0 | 0.536118 | 0.604132 | 0.198008 |
| LNC_004244 | XLOC_092001 | -- | intronic_lncRNA | 1.63888 | 0.37923 | 1.004453 | 1.502527 |
| LNC_004245 | XLOC_092055 | -- | intronic_lncRNA | 0.317011 | 0 | 0.167568 | 0.399081 |
| LNC_004246 | XLOC_092098 | -- | intronic_lncRNA | 0.681767 | 0.453964 | 0.682933 | 0.517552 |
| LNC_004247 | XLOC_092111 | -- | intronic_lncRNA | 0.343646 | 0.722423 | 0.210558 | 0.594508 |
| LNC_001199 | XLOC_025914 | -- | intronic_lncRNA | 0.754808 | 0.835657 | 0.223055 | 0.898771 |
| LNC_001198 | XLOC_025914 | -- | intronic_lncRNA | 0.412876 | 0.455599 | 1.582418 | 0.020419 |
| LNC_001197 | XLOC_025907 | -- | lincRNA | 0.360895 | 44.602333 | 10.579405 | 23.817921 |
| LNC_001196 | XLOC_025907 | -- | lincRNA | 76.224525 | 21.721077 | 128.586487 | 99.154205 |
| LNC_001195 | XLOC_025907 | -- | lincRNA | 52.892479 | 27.565294 | 0 | 0 |
| LNC_001194 | XLOC_025898 | -- | lincRNA | 0 | 2.324657 | 0 | 0 |

| LNC_001193 | XLOC_025883 | -- | intronic_lncRNA | 0.640988 | 1.196909 | 0.472418 | 0.987858 |
| --- | --- | --- | --- | --- | --- | --- | --- |
| LNC_001192 | XLOC_025855 | -- | intronic_lncRNA | 0 | 0.211721 | 0.410588 | 0.106326 |
| LNC_001191 | XLOC_025854 | -- | intronic_lncRNA | 1.128731 | 0 | 0.133119 | 0 |
| LNC_001190 | XLOC_025841 | -- | intronic_lncRNA | 0.557956 | 1.558895 | 1.398895 | 1.26877 |
| LNC_000113 | XLOC_000964 | -- | intronic_lncRNA | 0.20054 | 0.880603 | 0 | 0 |
| LNC_004107 | XLOC_088661 | -- | intronic_lncRNA | 0.566985 | 0.543732 | 0.288355 | 0.728408 |
| LNC_004106 | XLOC_088655 | -- | intronic_lncRNA | 0.094983 | 0.177786 | 0.460503 | 0.054129 |
| LNC_004105 | XLOC_088653 | -- | intronic_lncRNA | 4.714857 | 4.276285 | 4.417713 | 4.206174 |
| LNC_004104 | XLOC_088652 | -- | intronic_lncRNA | 0.091766 | 0.561613 | 0.041348 | 0.045199 |
| LNC_004103 | XLOC_088651 | -- | intronic_lncRNA | 0 | 0 | 0 | 0.438908 |
| LNC_004102 | XLOC_088649 | -- | intronic_lncRNA | 0 | 0.385491 | 0.36129 | 0.760425 |
| LNC_004101 | XLOC_088648 | -- | intronic_lncRNA | 0.57935 | 0.078775 | 0 | 0.471429 |
| LNC_004100 | XLOC_088647 | -- | intronic_lncRNA | 0 | 0.711864 | 0 | 0.361887 |
| LNC_004109 | XLOC_088664 | -- | intronic_lncRNA | 1.024988 | 0.558282 | 0.939537 | 0.702522 |
| LNC_004108 | XLOC_088662 | -- | intronic_lncRNA | 0.448862 | 0.676024 | 0.752534 | 0.345204 |
| LNC_001997 | XLOC_045167 | -- | intronic_lncRNA | 0.54321 | 0.402569 | 0.627487 | 0.529563 |
| LNC_001996 | XLOC_045166 | -- | intronic_lncRNA | 0.879543 | 0.358231 | 0.977801 | 0.460423 |
| LNC_001995 | XLOC_045150 | -- | intronic_lncRNA | 0.969406 | 0.420935 | 0.926408 | 1.592314 |
| LNC_001994 | XLOC_045107 | -- | intronic_lncRNA | 0.79235 | 0.841757 | 1.120172 | 1.380049 |
| LNC_001993 | XLOC_045094 | -- | lincRNA | 0.046917 | 0 | 0.31806 | 0.643359 |
| LNC_001992 | XLOC_045048 | -- | intronic_lncRNA | 0 | 0.185713 | 0.517446 | 0.811014 |
| LNC_001991 | XLOC_044980 | -- | intronic_lncRNA | 0.612792 | 0 | 0.104012 | 0 |
| LNC_001990 | XLOC_044878 | -- | intronic_lncRNA | 0.604827 | 0.755486 | 0.434235 | 0.339745 |
| LNC_001999 | XLOC_045181 | -- | intronic_lncRNA | 0 | 0 | 0.399755 | 0 |
| LNC_001998 | XLOC_045175 | -- | intronic_lncRNA | 0.926633 | 1.074933 | 0.795373 | 0.540908 |
| LNC_003739 | XLOC_080266 | -- | intronic_lncRNA | 0.458067 | 0.608367 | 0.486947 | 0.736094 |
| LNC_003738 | XLOC_080251 | -- | intronic_lncRNA | 0.506358 | 0.157811 | 0.7776 | 0.866245 |
| LNC_003731 | XLOC_080084 | -- | intronic_lncRNA | 1.492452 | 3.622739 | 0 | 0 |
| LNC_003730 | XLOC_080047 | -- | lincRNA | 2.515627 | 1.181298 | 0.364843 | 0.317805 |
| LNC_003733 | XLOC_080090 | -- | intronic_lncRNA | 0 | 0.208652 | 0.17725 | 0.430643 |
| LNC_003732 | XLOC_080086 | -- | lincRNA | 0.67352 | 0 | 0 | 0 |
| LNC_003735 | XLOC_080216 | -- | intronic_lncRNA | 0.541324 | 0.47181 | 0.631433 | 0.556117 |
| LNC_003734 | XLOC_080141 | -- | lincRNA | 0 | 0.211134 | 0.5133 | 0.301046 |
| LNC_003737 | XLOC_080226 | -- | intronic_lncRNA | 0.222309 | 0.552719 | 0.405156 | 0.48769 |
| LNC_003736 | XLOC_080216 | -- | intronic_lncRNA | 0.095109 | 0.158426 | 0.012844 | 0.351163 |
| LNC_003454 | XLOC_075227 | -- | lincRNA | 1.158936 | 0.053358 | 0 | 0.132619 |
| LNC_003455 | XLOC_075229 | -- | lincRNA | 2.469739 | 1.661565 | 0.554801 | 0.98686 |
| LNC_003456 | XLOC_075230 | -- | lincRNA | 1.278038 | 1.469597 | 0.39206 | 0.704828 |
| LNC_003457 | XLOC_075230 | -- | lincRNA | 2.247774 | 0.035531 | 0.064421 | 0.038193 |
| LNC_003450 | XLOC_075227 | -- | lincRNA | 0.482137 | 1.233034 | 0.185501 | 0.073102 |
| LNC_003451 | XLOC_075227 | -- | lincRNA | 1.11583 | 0.232594 | 0.006675 | 0.639126 |
| LNC_003452 | XLOC_075227 | -- | lincRNA | 0.958403 | 1.085603 | 0.291098 | 1.365721 |
| LNC_003453 | XLOC_075227 | -- | lincRNA | 1.66046 | 0.71434 | 0.578396 | 0.410673 |
| LNC_003458 | XLOC_075231 | -- | lincRNA | 4.915192 | 1.841248 | 2.229563 | 2.160262 |
| LNC_003459 | XLOC_075232 | -- | lincRNA | 1.44712 | 1.062011 | 0.227823 | 0.357815 |
| LNC_005676 | XLOC_132516 | -- | intronic_lncRNA | 0.255585 | 0.059055 | 0.114665 | 0.256998 |
| LNC_005677 | XLOC_132533 | -- | intronic_lncRNA | 0.903178 | 0.809194 | 0.412172 | 0.634746 |
| LNC_005674 | XLOC_132509 | -- | intronic_lncRNA | 0.835233 | 0 | 0 | 0 |
| LNC_005675 | XLOC_132513 | -- | intronic_lncRNA | 0.182175 | 0.243452 | 0.474282 | 0.368607 |
| LNC_005672 | XLOC_132460 | -- | intronic_lncRNA | 0.884885 | 0.120094 | 0.315507 | 0.058804 |
| LNC_005673 | XLOC_132480 | -- | lincRNA | 0.107896 | 0.419978 | 0.352221 | 0.598382 |
| LNC_005670 | XLOC_132431 | -- | intronic_lncRNA | 0.521236 | 0.14529 | 0.752611 | 0.709628 |
| LNC_005671 | XLOC_132431 | -- | intronic_lncRNA | 0 | 0.133484 | 0 | 0 |
| LNC_005678 | XLOC_132533 | -- | intronic_lncRNA | 0.510994 | 0.044458 | 0.047239 | 0.027756 |
| LNC_005679 | XLOC_132533 | -- | intronic_lncRNA | 1.062808 | 0 | 0.641976 | 0.358714 |
| LNC_005372 | XLOC_123539 | -- | intronic_lncRNA | 0.815387 | 0.649057 | 0.031166 | 0.375414 |
| LNC_001609 | XLOC_035074 | -- | intronic_lncRNA | 0.460011 | 0.413209 | 0.280729 | 0.105812 |
| LNC_001608 | XLOC_035057 | -- | lincRNA | 0.570814 | 0.147765 | 0.396485 | 0.349329 |
| LNC_001607 | XLOC_035049 | -- | intronic_lncRNA | 1.671925 | 1.297646 | 2.050144 | 1.859547 |
| LNC_001606 | XLOC_035043 | -- | intronic_lncRNA | 0.648286 | 0.215611 | 0 | 0.087292 |
| LNC_001605 | XLOC_034900 | -- | intronic_lncRNA | 0.713491 | 0.714301 | 0.401798 | 0.580822 |
| LNC_001604 | XLOC_034897 | -- | intronic_lncRNA | 0.200971 | 0.002074 | 0.36005 | 0.94145 |
| LNC_001603 | XLOC_034897 | -- | intronic_lncRNA | 0.952019 | 0.379815 | 0.378886 | 0.336952 |
| LNC_001602 | XLOC_034870 | -- | intronic_lncRNA | 0.679962 | 0.250737 | 0.459723 | 0.718565 |
| LNC_001601 | XLOC_034772 | -- | intronic_lncRNA | 0.638787 | 0.152579 | 0.122435 | 0.164381 |
| LNC_001600 | XLOC_034770 | -- | intronic_lncRNA | 0.067612 | 0.203506 | 0.845483 | 0.253601 |
| LNC_005139 | XLOC_117363 | -- | intronic_lncRNA | 0.867535 | 0.871656 | 0.602509 | 0.774488 |
| LNC_000458 | XLOC_008145 | -- | intronic_lncRNA | 0.249779 | 0.116221 | 0.474302 | 0.397298 |
| LNC_005078 | XLOC_115893 | -- | lincRNA | 1.708578 | 1.171342 | 0 | 0.040243 |
| LNC_005079 | XLOC_115946 | -- | intronic_lncRNA | 0.382598 | 0.164072 | 0.791713 | 0.550007 |
| LNC_000554 | XLOC_010310 | -- | intronic_lncRNA | 0.170023 | 0.072977 | 0.557281 | 0.37125 |
| LNC_000555 | XLOC_010349 | -- | intronic_lncRNA | 0.519802 | 0 | 0 | 0.34449 |
| LNC_000556 | XLOC_010449 | -- | intronic_lncRNA | 0.548393 | 0.225249 | 0 | 0.326379 |
| LNC_000557 | XLOC_010457 | -- | intronic_lncRNA | 0.851811 | 0.407437 | 0.281716 | 0.520098 |
| LNC_000550 | XLOC_010192 | -- | intronic_lncRNA | 0.116871 | 0 | 0 | 0.489348 |
| LNC_005071 | XLOC_115731 | -- | intronic_lncRNA | 0.453342 | 0.135339 | 1.253439 | 0.752898 |
| LNC_000552 | XLOC_010240 | -- | antisense_lncRNA | 0 | 0 | 0 | 0.578101 |

| LNC_000553 | XLOC_010286 | -- | intronic_lncRNA | 0.328534 | 0.167386 | 0.101669 | 0.576253 |
| --- | --- | --- | --- | --- | --- | --- | --- |
| LNC_000451 | XLOC_007936 | -- | intronic_lncRNA | 0.490437 | 0.23727 | 0.313219 | 0.563612 |
| LNC_000450 | XLOC_007921 | -- | antisense_lncRNA | 0.377269 | 0.505437 | 0.057542 | 0 |
| LNC_005133 | XLOC_117255 | -- | intronic_lncRNA | 0.445715 | 0.491817 | 0.266126 | 0.456771 |
| LNC_005132 | XLOC_117252 | -- | intronic_lncRNA | 0.33231 | 0.117085 | 0.806211 | 0.461964 |
| LNC_000455 | XLOC_008029 | -- | intronic_lncRNA | 0.295074 | 0.353725 | 0.55078 | 0 |
| LNC_000454 | XLOC_008010 | -- | lincRNA | 0.205568 | 0 | 0.244001 | 0.699542 |
| LNC_003924 | XLOC_084604 | -- | intronic_lncRNA | 0.462634 | 0.224804 | 0 | 0.525454 |
| LNC_003925 | XLOC_084607 | -- | intronic_lncRNA | 0.077168 | 0 | 0 | 0.408652 |
| LNC_003926 | XLOC_084608 | -- | intronic_lncRNA | 0.192331 | 0.34004 | 0 | 0.594816 |
| LNC_003927 | XLOC_084609 | -- | intronic_lncRNA | 1.046333 | 0.451266 | 0.338532 | 0.411233 |
| LNC_003920 | XLOC_084510 | -- | intronic_lncRNA | 0.388178 | 0.193654 | 0.233419 | 0.235208 |
| LNC_005137 | XLOC_117294 | -- | intronic_lncRNA | 0.385839 | 0.246789 | 0.560851 | 0.908151 |
| LNC_003922 | XLOC_084557 | -- | intronic_lncRNA | 1.29328 | 0.405576 | 0.422597 | 0.425751 |
| LNC_003923 | XLOC_084565 | -- | intronic_lncRNA | 0.87677 | 0.246384 | 0.22552 | 0.242463 |
| LNC_005136 | XLOC_117290 | -- | intronic_lncRNA | 0.099379 | 0.139169 | 0.504637 | 0.109177 |
| LNC_003928 | XLOC_084637 | -- | intronic_lncRNA | 0 | 0 | 0 | 0.965771 |
| LNC_003929 | XLOC_084644 | -- | intronic_lncRNA | 0 | 1.071572 | 0.060986 | 0 |
| LNC_000169 | XLOC_002015 | -- | intronic_lncRNA | 1.239731 | 0.460483 | 0.666505 | 0.590311 |
| LNC_000168 | XLOC_001990 | -- | intronic_lncRNA | 0.75541 | 2.396082 | 1.085923 | 1.509274 |
| LNC_000165 | XLOC_001922 | -- | intronic_lncRNA | 0.969571 | 0.799856 | 0.700362 | 0.627528 |
| LNC_000164 | XLOC_001844 | -- | intronic_lncRNA | 0.349687 | 0.776267 | 0.9568 | 0.487698 |
| LNC_005447 | XLOC_125558 | -- | intronic_lncRNA | 1.155559 | 1.007744 | 1.44277 | 0 |
| LNC_000166 | XLOC_001927 | -- | intronic_lncRNA | 0.306332 | 0.131382 | 0.511923 | 0.67492 |
| LNC_000161 | XLOC_001795 | -- | lincRNA | 0.146049 | 0.589824 | 0 | 0 |
| LNC_000160 | XLOC_001753 | -- | intronic_lncRNA | 0.421288 | 0.602411 | 0.509593 | 0.368941 |
| LNC_000163 | XLOC_001815 | -- | intronic_lncRNA | 0 | 0 | 0.620203 | 0.300169 |
| LNC_000162 | XLOC_001796 | -- | intronic_lncRNA | 0.503068 | 0.837445 | 0.470131 | 0.473494 |
| LNC_001388 | XLOC_030484 | -- | intronic_lncRNA | 0 | 0.176472 | 0 | 0.536419 |
| LNC_001389 | XLOC_030486 | -- | intronic_lncRNA | 0.156684 | 0.529967 | 0 | 0.75909 |
| LNC_001382 | XLOC_030376 | -- | intronic_lncRNA | 0.496844 | 0.6399 | 0.689086 | 0.951953 |
| LNC_001383 | XLOC_030381 | -- | intronic_lncRNA | 0.269997 | 0.60865 | 1.109347 | 0.864389 |
| LNC_001380 | XLOC_030293 | -- | intronic_lncRNA | 0.826346 | 0.230246 | 0.244766 | 0.348463 |
| LNC_001381 | XLOC_030347 | -- | intronic_lncRNA | 0.848762 | 0.549789 | 0.459794 | 1.313627 |
| LNC_001386 | XLOC_030465 | -- | intronic_lncRNA | 0.936251 | 0.395949 | 1.131413 | 0.97358 |
| LNC_001387 | XLOC_030477 | -- | intronic_lncRNA | 0.103133 | 0.154731 | 0.532846 | 0.268271 |
| LNC_001384 | XLOC_030446 | -- | lincRNA | 0.129807 | 0 | 0.289946 | 0.434844 |
| LNC_001385 | XLOC_030458 | -- | intronic_lncRNA | 1.681184 | 1.316852 | 2.182713 | 1.881178 |
| LNC_004712 | XLOC_104899 | -- | intronic_lncRNA | 0.912499 | 0.687991 | 1.125475 | 1.099051 |
| LNC_004713 | XLOC_104994 | -- | intronic_lncRNA | 0.598609 | 0 | 0.237816 | 0.16031 |
| LNC_004710 | XLOC_104882 | -- | intronic_lncRNA | 0 | 0 | 0.429883 | 0 |
| LNC_004711 | XLOC_104896 | -- | intronic_lncRNA | 0.514582 | 0.440501 | 0.464179 | 0.308701 |
| LNC_004716 | XLOC_105185 | -- | intronic_lncRNA | 0.202319 | 0.421543 | 0.335628 | 0.540743 |
| LNC_004717 | XLOC_105188 | -- | intronic_lncRNA | 0.473341 | 0.082856 | 0.414689 | 0.672021 |
| LNC_004714 | XLOC_104995 | -- | intronic_lncRNA | 0.285883 | 0.41899 | 1.232033 | 0.189906 |
| LNC_004715 | XLOC_105018 | -- | intronic_lncRNA | 0.477402 | 0.674048 | 0.438794 | 0.735545 |
| LNC_004718 | XLOC_105191 | -- | intronic_lncRNA | 0.166279 | 0.087292 | 0.497943 | 0.142075 |
| LNC_004719 | XLOC_105211 | -- | intronic_lncRNA | 0.088426 | 0 | 0.214116 | 0.503434 |
| LNC_000833 | XLOC_017855 | -- | lincRNA | 0.865757 | 0.53582 | 0.340262 | 0.443811 |
| LNC_000832 | XLOC_017848 | -- | antisense_lncRNA | 0 | 0.091482 | 0.584795 | 0 |
| LNC_000831 | XLOC_017701 | -- | intronic_lncRNA | 0.511851 | 0.725839 | 0.092241 | 0.117702 |
| LNC_000830 | XLOC_017594 | -- | intronic_lncRNA | 0.588815 | 0.202537 | 1.141559 | 0.665657 |
| LNC_000837 | XLOC_017959 | -- | intronic_lncRNA | 0.734548 | 0.611846 | 1.416301 | 1.45016 |
| LNC_000836 | XLOC_017959 | -- | intronic_lncRNA | 0.211935 | 0.763979 | 0.469629 | 0.294655 |
| LNC_000835 | XLOC_017927 | -- | intronic_lncRNA | 0.065081 | 0.56346 | 0.045174 | 0.114644 |
| LNC_000834 | XLOC_017871 | -- | intronic_lncRNA | 3.411398 | 3.128907 | 1.983163 | 2.872392 |
| LNC_000839 | XLOC_017959 | -- | intronic_lncRNA | 3.835699 | 6.290742 | 5.224659 | 4.288972 |
| LNC_000838 | XLOC_017959 | -- | intronic_lncRNA | 0.961109 | 1.419675 | 0.713526 | 2.348424 |
| LNC_006408 | XLOC_153684 | -- | intronic_lncRNA | 0.212152 | 0.423353 | 0.171428 | 0.528364 |
| LNC_006409 | XLOC_153752 | -- | intronic_lncRNA | 0.319014 | 1.432726 | 0 | 0.699986 |
| LNC_002093 | XLOC_047600 | -- | intronic_lncRNA | 0.457203 | 0.474075 | 0.391427 | 0.4718 |
| LNC_002092 | XLOC_047590 | -- | intronic_lncRNA | 0.882022 | 1.032887 | 0.543764 | 0.384303 |
| LNC_002091 | XLOC_047543 | -- | intronic_lncRNA | 0.288181 | 1.284552 | 0.174381 | 0.324782 |
| LNC_002090 | XLOC_047523 | -- | intronic_lncRNA | 0 | 0 | 0 | 0.450689 |
| LNC_002097 | XLOC_047617 | -- | intronic_lncRNA | 0.629631 | 0.469902 | 0.444162 | 0.800696 |
| LNC_002096 | XLOC_047613 | -- | intronic_lncRNA | 0.66387 | 0.060416 | 0.509834 | 0.114799 |
| LNC_002095 | XLOC_047612 | -- | intronic_lncRNA | 0.460535 | 0.620867 | 0.037307 | 0.223703 |
| LNC_002094 | XLOC_047603 | -- | intronic_lncRNA | 0.90791 | 0.496124 | 0.284081 | 0.522902 |
| LNC_002099 | XLOC_047685 | -- | antisense_lncRNA | 0.874281 | 0.413544 | 0.51486 | 0.313248 |
| LNC_002098 | XLOC_047674 | -- | intronic_lncRNA | 27.618647 | 14.733362 | 27.94799 | 23.400581 |
| LNC_001430 | XLOC_031220 | -- | intronic_lncRNA | 0 | 0 | 0.631575 | 0.545829 |
| LNC_000769 | XLOC_016113 | -- | intronic_lncRNA | 0.52733 | 0.193796 | 0.334199 | 0.57339 |
| LNC_000768 | XLOC_016030 | -- | intronic_lncRNA | 0.842108 | 0.658958 | 1.029068 | 0.805243 |
| LNC_001431 | XLOC_031220 | -- | intronic_lncRNA | 0.995115 | 0.117751 | 0.304593 | 0 |
| LNC_000763 | XLOC_015838 | -- | intronic_lncRNA | 0.535574 | 0.645674 | 0.663472 | 0.358583 |
| LNC_000762 | XLOC_015834 | -- | intronic_lncRNA | 0.663103 | 0.407156 | 0.40755 | 0.6755 |
| LNC_000761 | XLOC_015802 | -- | lincRNA | 0 | 0 | 0.481673 | 0.532863 |

| LNC_000760 | XLOC_015792 | -- | lincRNA | 0.531673 | 0 | 0 | 0 |
| --- | --- | --- | --- | --- | --- | --- | --- |
| LNC_000767 | XLOC_016029 | -- | intronic_lncRNA | 0.573667 | 0.096445 | 0.217092 | 0.526426 |
| LNC_000766 | XLOC_016006 | -- | intronic_lncRNA | 0.02049 | 0 | 0.650276 | 0.801128 |
| LNC_000765 | XLOC_015999 | -- | intronic_lncRNA | 0.580587 | 0 | 0.398683 | 0.458177 |
| LNC_000764 | XLOC_015933 | -- | intronic_lncRNA | 0.455758 | 1.000074 | 0.425615 | 0.475344 |
| LNC_006353 | XLOC_152277 | -- | intronic_lncRNA | 0.102469 | 1.076763 | 0.877588 | 0.970704 |
| LNC_006354 | XLOC_152278 | -- | intronic_lncRNA | 0.251714 | 0.849317 | 0.33314 | 0.462502 |
| LNC_006355 | XLOC_152310 | -- | intronic_lncRNA | 1.456139 | 0.991145 | 0.937831 | 1.07269 |
| LNC_002545 | XLOC_058889 | -- | intronic_lncRNA | 0.89028 | 0.440415 | 0.425814 | 0.51974 |
| LNC_002544 | XLOC_058879 | -- | intronic_lncRNA | 0.335131 | 0.717441 | 0.999162 | 1.102317 |
| LNC_002547 | XLOC_058935 | -- | intronic_lncRNA | 1.04898 | 1.214478 | 0.957534 | 0.555703 |
| LNC_002546 | XLOC_058922 | -- | lincRNA | 0.363566 | 2.234752 | 0 | 0.049644 |
| LNC_002541 | XLOC_058876 | -- | intronic_lncRNA | 0.569021 | 0.044766 | 0.265497 | 0.18594 |
| LNC_002540 | XLOC_058875 | -- | intronic_lncRNA | 0.241512 | 0.500922 | 0.60664 | 0.461211 |
| LNC_002543 | XLOC_058879 | -- | intronic_lncRNA | 0.578025 | 0.301714 | 0.069108 | 0.347407 |
| LNC_002542 | XLOC_058876 | -- | intronic_lncRNA | 0 | 0.305208 | 0.891741 | 0.785347 |
| LNC_006357 | XLOC_152358 | -- | intronic_lncRNA | 5.767539 | 3.036106 | 1.22422 | 1.43424 |
| LNC_002549 | XLOC_058999 | -- | intronic_lncRNA | 0.344337 | 0.052272 | 0.176119 | 0.895894 |
| LNC_002548 | XLOC_058999 | -- | intronic_lncRNA | 0.644712 | 0.130043 | 0.430043 | 1.12712 |
| LNC_003155 | XLOC_072771 | -- | intronic_lncRNA | 0.628123 | 0.724992 | 0.512117 | 0.421364 |
| LNC_001438 | XLOC_031254 | -- | intronic_lncRNA | 1.297554 | 0.638296 | 0.861177 | 0.752146 |
| LNC_003157 | XLOC_072930 | -- | lincRNA | 0.430259 | 0.735078 | 0.493147 | 0.174835 |
| LNC_003156 | XLOC_072790 | -- | lincRNA | 0 | 0 | 0.536046 | 0.089624 |
| LNC_003151 | XLOC_072744 | -- | intronic_lncRNA | 4.620565 | 2.440144 | 3.46189 | 3.763918 |
| LNC_003150 | XLOC_072744 | -- | intronic_lncRNA | 0.760087 | 0.91707 | 0.216076 | 0.184507 |
| LNC_003153 | XLOC_072746 | -- | intronic_lncRNA | 0.458783 | 0.840325 | 0.117413 | 0.99393 |
| LNC_003152 | XLOC_072744 | -- | intronic_lncRNA | 0.598332 | 0.303925 | 1.16339 | 0.794433 |
| LNC_003159 | XLOC_072979 | -- | intronic_lncRNA | 0.551064 | 0.441207 | 0.69033 | 0.432046 |
| LNC_003158 | XLOC_072930 | -- | lincRNA | 0.076975 | 0.489111 | 0.215107 | 0.499416 |
| LNC_006429 | XLOC_154049 | -- | intronic_lncRNA | 0.500796 | 0.292251 | 0.723056 | 0.241205 |
| LNC_000499 | XLOC_008785 | -- | intronic_lncRNA | 1.978036 | 1.561462 | 2.269593 | 1.361672 |
| LNC_000498 | XLOC_008784 | -- | intronic_lncRNA | 0.714655 | 0.323609 | 0.277848 | 0.207005 |
| LNC_000495 | XLOC_008755 | -- | intronic_lncRNA | 0.630075 | 1.728865 | 1.289964 | 0.9184 |
| LNC_000494 | XLOC_008745 | -- | intronic_lncRNA | 0.790556 | 0.432526 | 0.660198 | 0.407867 |
| LNC_000497 | XLOC_008771 | -- | intronic_lncRNA | 0.520525 | 0.231688 | 0.76357 | 1.062873 |
| LNC_000496 | XLOC_008755 | -- | intronic_lncRNA | 1.891599 | 1.463727 | 1.483289 | 0.83808 |
| LNC_000491 | XLOC_008727 | -- | intronic_lncRNA | 0.88324 | 0.237084 | 0.133913 | 0.092215 |
| LNC_000490 | XLOC_008722 | -- | intronic_lncRNA | 1.130367 | 0.372651 | 0.787537 | 0 |
| LNC_000493 | XLOC_008733 | -- | intronic_lncRNA | 0.226664 | 0.457318 | 0.472867 | 0.168707 |
| LNC_000492 | XLOC_008732 | -- | intronic_lncRNA | 0 | 0.49152 | 0.161374 | 0.705774 |
| LNC_002400 | XLOC_055424 | -- | intronic_lncRNA | 0.257088 | 0.824313 | 0.344456 | 0 |
| LNC_002401 | XLOC_055425 | -- | intronic_lncRNA | 0.180682 | 0 | 0.182854 | 0.766612 |
| LNC_002402 | XLOC_055528 | -- | intronic_lncRNA | 0.27909 | 0.940115 | 1.423533 | 1.245648 |
| LNC_002403 | XLOC_055528 | -- | intronic_lncRNA | 0.720695 | 0 | 0 | 0 |
| LNC_002404 | XLOC_055565 | -- | intronic_lncRNA | 0 | 0.722148 | 0 | 0 |
| LNC_002405 | XLOC_055623 | -- | intronic_lncRNA | 0.264273 | 0.339068 | 0.201305 | 0.583137 |
| LNC_002406 | XLOC_055673 | -- | intronic_lncRNA | 1.49947 | 0.33405 | 0.789798 | 0.657294 |
| LNC_002407 | XLOC_055749 | -- | lincRNA | 1.411679 | 0.767015 | 2.214411 | 1.842102 |
| LNC_002408 | XLOC_055749 | -- | lincRNA | 0 | 0.274878 | 1.284718 | 0.697722 |
| LNC_002409 | XLOC_055763 | -- | intronic_lncRNA | 0.67304 | 0.591998 | 0.516299 | 0.315113 |
| LNC_001018 | XLOC_022110 | -- | intronic_lncRNA | 0.50379 | 0.365854 | 0.918114 | 0.78272 |
| LNC_001019 | XLOC_022119 | -- | intronic_lncRNA | 0.404338 | 0.775059 | 0.257569 | 0.340713 |
| LNC_001012 | XLOC_021939 | -- | intronic_lncRNA | 0.177666 | 0.515741 | 0.03531 | 0.387876 |
| LNC_001013 | XLOC_021953 | -- | intronic_lncRNA | 0.24914 | 0.339007 | 0 | 0.506043 |
| LNC_001010 | XLOC_021713 | -- | antisense_lncRNA | 0.975039 | 0 | 0.095775 | 0.212233 |
| LNC_001011 | XLOC_021921 | -- | intronic_lncRNA | 0 | 0.51555 | 0 | 0 |
| LNC_001016 | XLOC_022108 | -- | intronic_lncRNA | 0.484616 | 0 | 0.289117 | 0.26521 |
| LNC_001017 | XLOC_022109 | -- | intronic_lncRNA | 0.19775 | 0.231595 | 0.405562 | 0.220644 |
| LNC_001014 | XLOC_021953 | -- | intronic_lncRNA | 1.386334 | 0.339904 | 0.777514 | 0.211261 |
| LNC_001015 | XLOC_021959 | -- | intronic_lncRNA | 0.759136 | 0.595883 | 0.102186 | 0 |
| LNC_001171 | XLOC_025495 | -- | intronic_lncRNA | 0.279948 | 0.308176 | 0.282295 | 0.420566 |
| LNC_001170 | XLOC_025488 | -- | intronic_lncRNA | 0.685113 | 0.305332 | 0.914895 | 0.531704 |
| LNC_001173 | XLOC_025518 | -- | intronic_lncRNA | 0 | 0 | 0.112286 | 0.550888 |
| LNC_001172 | XLOC_025510 | -- | intronic_lncRNA | 0.407168 | 0.347916 | 0.473774 | 0.289616 |
| LNC_001175 | XLOC_025543 | -- | intronic_lncRNA | 0.500561 | 0.235787 | 0.214556 | 0.427122 |
| LNC_001174 | XLOC_025531 | -- | intronic_lncRNA | 0 | 0.418569 | 0 | 0 |
| LNC_001177 | XLOC_025645 | -- | intronic_lncRNA | 0.433469 | 0.568503 | 0.257715 | 0.742977 |
| LNC_001176 | XLOC_025643 | -- | intronic_lncRNA | 0.274613 | 0.142855 | 0.220476 | 0.694037 |
| LNC_001179 | XLOC_025651 | -- | intronic_lncRNA | 0.742692 | 0.531824 | 0.947689 | 0.912061 |
| LNC_001178 | XLOC_025646 | -- | intronic_lncRNA | 0.484571 | 0.639554 | 0.559393 | 0.722237 |
| LNC_004279 | XLOC_092959 | -- | lincRNA | 0.645029 | 0.341425 | 0.131359 | 0.200511 |
| LNC_004278 | XLOC_092939 | -- | intronic_lncRNA | 0.871054 | 0.544943 | 0.611867 | 0.486232 |
| LNC_003713 | XLOC_079543 | -- | intronic_lncRNA | 0.519932 | 0.135724 | 0.73156 | 0.08336 |
| LNC_003712 | XLOC_079525 | -- | intronic_lncRNA | 0.729101 | 0.165914 | 0.819992 | 0.664664 |
| LNC_003711 | XLOC_079449 | -- | intronic_lncRNA | 0.645745 | 0.545305 | 0.886334 | 0.983208 |
| LNC_003710 | XLOC_079390 | -- | intronic_lncRNA | 0.953398 | 0.915816 | 0.077269 | 0.688503 |
| LNC_003717 | XLOC_079729 | -- | intronic_lncRNA | 0 | 0 | 0.679689 | 0.528044 |

| LNC_003716 | XLOC_079701 | -- | intronic_lncRNA | 1.802749 | 0.289492 | 1.170139 | 0.927202 |
| --- | --- | --- | --- | --- | --- | --- | --- |
| LNC_003715 | XLOC_079697 | -- | intronic_lncRNA | 0.827887 | 0.30607 | 0.577867 | 0.45714 |
| LNC_003714 | XLOC_079548 | -- | intronic_lncRNA | 0.530193 | 0 | 0 | 0 |
| LNC_003719 | XLOC_079737 | -- | intronic_lncRNA | 0 | 0 | 0 | 0.615973 |
| LNC_003718 | XLOC_079737 | -- | intronic_lncRNA | 0.396081 | 0.426378 | 0.375695 | 0.518392 |
| LNC_005386 | XLOC_124077 | -- | intronic_lncRNA | 0 | 0 | 0.54777 | 0.198076 |
| LNC_005387 | XLOC_124078 | -- | intronic_lncRNA | 0.510135 | 0 | 0.195367 | 0.116369 |
| LNC_005384 | XLOC_124075 | -- | intronic_lncRNA | 0 | 0 | 0.456032 | 0 |
| LNC_005385 | XLOC_124076 | -- | intronic_lncRNA | 0 | 0 | 0.406678 | 0.05828 |
| LNC_005382 | XLOC_123915 | -- | lincRNA | 0 | 0 | 0 | 0.419314 |
| LNC_005383 | XLOC_124074 | -- | intronic_lncRNA | 0.551197 | 0 | 0.183173 | 0 |
| LNC_005380 | XLOC_123777 | -- | intronic_lncRNA | 0.243493 | 0 | 0 | 0.623405 |
| LNC_005381 | XLOC_123892 | -- | lincRNA | 0 | 0.254585 | 0.455761 | 0 |
| LNC_005388 | XLOC_124088 | -- | intronic_lncRNA | 0 | 0.211263 | 0.105474 | 0.500507 |
| LNC_005389 | XLOC_124307 | -- | lincRNA | 0.433961 | 0.781371 | 0.595936 | 0.522985 |
| LNC_003438 | XLOC_075218 | -- | lincRNA | 1.610053 | 0.285554 | 0.244804 | 0.216218 |
| LNC_003439 | XLOC_075219 | -- | lincRNA | 2.750622 | 0.560516 | 0.192292 | 1.021893 |
| LNC_003436 | XLOC_075216 | -- | lincRNA | 1.711124 | 0.597287 | 0.272205 | 0.215473 |
| LNC_003437 | XLOC_075217 | -- | lincRNA | 2.37776 | 1.207159 | 0.238478 | 0.354885 |
| LNC_003434 | XLOC_075214 | -- | lincRNA | 5.763287 | 1.834487 | 1.970322 | 1.630386 |
| LNC_003435 | XLOC_075215 | -- | lincRNA | 4.190822 | 1.559758 | 0.598119 | 0.948767 |
| LNC_003432 | XLOC_075212 | -- | lincRNA | 1.649526 | 1.064116 | 0.96531 | 0.233515 |
| LNC_003433 | XLOC_075213 | -- | lincRNA | 0.319634 | 0.854748 | 0.196107 | 0.492636 |
| LNC_003430 | XLOC_075210 | -- | lincRNA | 1.422214 | 0.813074 | 0.647355 | 0.459185 |
| LNC_003431 | XLOC_075211 | -- | lincRNA | 3.633933 | 2.136356 | 1.225635 | 1.028695 |
| LNC_005658 | XLOC_132076 | -- | intronic_lncRNA | 1.024578 | 0.24667 | 0.222096 | 0.689274 |
| LNC_005659 | XLOC_132204 | -- | intronic_lncRNA | 0.929219 | 0.620824 | 0.82628 | 0.951612 |
| LNC_005650 | XLOC_131609 | -- | intronic_lncRNA | 1.911425 | 0.548597 | 1.529621 | 0.880391 |
| LNC_005651 | XLOC_131631 | -- | intronic_lncRNA | 0 | 0 | 0 | 0.396314 |
| LNC_005652 | XLOC_131727 | -- | intronic_lncRNA | 0 | 0 | 0.299929 | 0.806886 |
| LNC_005653 | XLOC_131729 | -- | intronic_lncRNA | 0 | 0 | 0 | 0.538314 |
| LNC_005654 | XLOC_131729 | -- | intronic_lncRNA | 0.395854 | 0.074593 | 0.647805 | 0 |
| LNC_005655 | XLOC_131810 | -- | lincRNA | 0.134315 | 0.483328 | 0 | 0 |
| LNC_005656 | XLOC_132076 | -- | intronic_lncRNA | 0.405973 | 0.702753 | 0.347354 | 0.804154 |
| LNC_005657 | XLOC_132076 | -- | intronic_lncRNA | 0.626079 | 0.734015 | 1.287026 | 0.697587 |
| LNC_002390 | XLOC_055262 | -- | intronic_lncRNA | 0.593707 | 0.461537 | 0.500421 | 0.494233 |
| LNC_002391 | XLOC_055263 | -- | intronic_lncRNA | 0.1349 | 0.360285 | 0.605927 | 0.576423 |
| LNC_002392 | XLOC_055285 | -- | intronic_lncRNA | 0 | 0 | 0.424439 | 0.140041 |
| LNC_002393 | XLOC_055286 | -- | intronic_lncRNA | 0 | 0.473952 | 0.785473 | 0 |
| LNC_002394 | XLOC_055287 | -- | intronic_lncRNA | 0 | 0 | 0.402611 | 0.06709 |
| LNC_002395 | XLOC_055290 | -- | intronic_lncRNA | 0.532042 | 0.759002 | 0.593802 | 0.695804 |
| LNC_002396 | XLOC_055311 | -- | intronic_lncRNA | 0.393217 | 1.016406 | 0.43543 | 0.526525 |
| LNC_002397 | XLOC_055349 | -- | intronic_lncRNA | 0.220851 | 0.356371 | 0.670297 | 0.238264 |
| LNC_002398 | XLOC_055400 | -- | intronic_lncRNA | 2.301259 | 0.898081 | 1.325394 | 1.077886 |
| LNC_002399 | XLOC_055423 | -- | intronic_lncRNA | 0.708164 | 0.754253 | 0.671073 | 0.52503 |
| LNC_003380 | XLOC_075175 | -- | lincRNA | 0.875332 | 1.327992 | 0.407737 | 0.367657 |
| LNC_003381 | XLOC_075175 | -- | lincRNA | 2.126443 | 1.148439 | 0 | 0.836031 |
| LNC_003382 | XLOC_075175 | -- | lincRNA | 0.602659 | 0.718248 | 0.713609 | 0 |
| LNC_003383 | XLOC_075176 | -- | lincRNA | 1.451303 | 0.093213 | 0.134204 | 0.898115 |
| LNC_003384 | XLOC_075177 | -- | lincRNA | 2.215524 | 0 | 0 | 0 |
| LNC_003385 | XLOC_075177 | -- | lincRNA | 12.835317 | 8.398623 | 3.958045 | 5.49651 |
| LNC_003386 | XLOC_075180 | -- | lincRNA | 4.684531 | 2.11358 | 1.203278 | 1.667733 |
| LNC_003387 | XLOC_075181 | -- | lincRNA | 3.619956 | 2.727999 | 0.532044 | 1.178928 |
| LNC_003388 | XLOC_075182 | -- | lincRNA | 1.329871 | 1.697356 | 0.397417 | 0.115348 |
| LNC_003389 | XLOC_075183 | -- | lincRNA | 2.545296 | 1.178772 | 0.517611 | 0.387657 |
| LNC_006099 | XLOC_145929 | -- | intronic_lncRNA | 1.051641 | 0.611112 | 1.640247 | 1.740771 |
| LNC_006098 | XLOC_145929 | -- | intronic_lncRNA | 0.986149 | 0.505539 | 0.321079 | 0.454144 |
| LNC_006097 | XLOC_145837 | -- | intronic_lncRNA | 0.11138 | 0.224248 | 0.470678 | 0.438555 |
| LNC_006096 | XLOC_145835 | -- | antisense_lncRNA | 0 | 0 | 0.36241 | 0 |
| LNC_006095 | XLOC_145835 | -- | intronic_lncRNA | 0 | 0.489301 | 0 | 0 |
| LNC_006094 | XLOC_145835 | -- | intronic_lncRNA | 0.234173 | 0.165357 | 0.501476 | 0.151145 |
| LNC_006093 | XLOC_145811 | -- | intronic_lncRNA | 0.190911 | 0 | 0.411467 | 0.171218 |
| LNC_006092 | XLOC_145810 | -- | intronic_lncRNA | 0.3489 | 0.928111 | 0.774431 | 0.7203 |
| LNC_006091 | XLOC_145806 | -- | intronic_lncRNA | 0 | 0.38064 | 0.398838 | 0.4816 |
| LNC_006090 | XLOC_145741 | -- | intronic_lncRNA | 0.68623 | 0.834447 | 0.468986 | 0.567744 |
| LNC_004529 | XLOC_099327 | -- | intronic_lncRNA | 1.511434 | 1.224605 | 1.133368 | 0.91157 |
| LNC_004528 | XLOC_099323 | -- | intronic_lncRNA | 0 | 0 | 0.521903 | 0 |
| LNC_004521 | XLOC_099023 | -- | intronic_lncRNA | 0.339384 | 0 | 0.420077 | 0.461238 |
| LNC_004520 | XLOC_099019 | -- | intronic_lncRNA | 0.201516 | 0.193331 | 0.62524 | 0.179504 |
| LNC_004523 | XLOC_099064 | -- | intronic_lncRNA | 0.357102 | 0.17824 | 0.611849 | 0.462585 |
| LNC_004522 | XLOC_099023 | -- | intronic_lncRNA | 0.487091 | 0.09135 | 0.508853 | 0.057525 |
| LNC_004525 | XLOC_099136 | -- | intronic_lncRNA | 0.308104 | 0.257775 | 0.198347 | 0.846183 |
| LNC_004524 | XLOC_099122 | -- | intronic_lncRNA | 0.313563 | 0.359959 | 1.234133 | 0.905018 |
| LNC_004527 | XLOC_099263 | -- | intronic_lncRNA | 0.582025 | 0.829385 | 0.478427 | 0.638403 |
| LNC_004526 | XLOC_099137 | -- | intronic_lncRNA | 0.078404 | 0.338637 | 0.086519 | 0.485866 |
| LNC_005016 | XLOC_114561 | -- | intronic_lncRNA | 0.587807 | 0.25365 | 0.075154 | 0.540264 |
| LNC_000537 | XLOC_009994 | -- | intronic_lncRNA | 0.68538 | 0.944497 | 0.778477 | 0 |

| LNC_000534 | XLOC_009956 | -- | lincRNA | 0 | 0 | 0 | 0.529094 |
| --- | --- | --- | --- | --- | --- | --- | --- |
| LNC_000535 | XLOC_009963 | -- | intronic_lncRNA | 0.568903 | 0.638499 | 0.386609 | 0.686555 |
| LNC_000532 | XLOC_009902 | -- | intronic_lncRNA | 2.586421 | 1.479769 | 0.955044 | 0.46941 |
| LNC_000533 | XLOC_009917 | -- | intronic_lncRNA | 0.520617 | 0.736764 | 0.914704 | 0.682061 |
| LNC_000530 | XLOC_009901 | -- | intronic_lncRNA | 0.991852 | 0.29787 | 0.671588 | 0.997001 |
| LNC_000531 | XLOC_009902 | -- | intronic_lncRNA | 1.109073 | 0.359662 | 0.165978 | 0.078081 |
| LNC_000538 | XLOC_009994 | -- | intronic_lncRNA | 0 | 0 | 0.173155 | 0.849471 |
| LNC_000539 | XLOC_009994 | -- | intronic_lncRNA | 0.373232 | 0.303053 | 0.70901 | 0.42666 |
| LNC_003948 | XLOC_084972 | -- | intronic_lncRNA | 0.007213 | 0.705669 | 0.007999 | 0.542373 |
| LNC_003949 | XLOC_084972 | -- | intronic_lncRNA | 0.315522 | 0.534984 | 0.415205 | 0.073723 |
| LNC_003946 | XLOC_084971 | -- | intronic_lncRNA | 0.069682 | 0.449877 | 0.294528 | 0.287011 |
| LNC_003947 | XLOC_084971 | -- | intronic_lncRNA | 0.219385 | 0 | 0.081548 | 0.157042 |
| LNC_003944 | XLOC_084969 | -- | intronic_lncRNA | 0.52161 | 0.889587 | 0 | 0.388831 |
| LNC_003945 | XLOC_084970 | -- | intronic_lncRNA | 0.191934 | 0.481108 | 0.196122 | 0.204426 |
| LNC_003942 | XLOC_084915 | -- | antisense_lncRNA | 0.507033 | 0.488154 | 0.443512 | 0.662512 |
| LNC_003943 | XLOC_084956 | -- | lincRNA | 0.258894 | 0.568673 | 1.495699 | 0.741649 |
| LNC_003940 | XLOC_084863 | -- | intronic_lncRNA | 0.185117 | 0 | 0.36036 | 0.461551 |
| LNC_003941 | XLOC_084863 | -- | intronic_lncRNA | 0 | 0.631492 | 0.036237 | 0.035547 |
| LNC_000147 | XLOC_001496 | -- | intronic_lncRNA | 0.626437 | 0.433402 | 0.082471 | 0 |
| LNC_000146 | XLOC_001490 | -- | intronic_lncRNA | 0 | 0.196181 | 0.425079 | 0 |
| LNC_000145 | XLOC_001474 | -- | antisense_lncRNA | 0 | 0 | 0.487438 | 0.57552 |
| LNC_000144 | XLOC_001442 | -- | intronic_lncRNA | 0.61133 | 0.332253 | 0.25946 | 0.401647 |
| LNC_000143 | XLOC_001423 | -- | intronic_lncRNA | 0.199114 | 0.028783 | 0.919624 | 0.359901 |
| LNC_000142 | XLOC_001423 | -- | intronic_lncRNA | 0.619175 | 0.473539 | 1.527521 | 0.381814 |
| LNC_000141 | XLOC_001423 | -- | antisense_lncRNA | 0.387059 | 0.281348 | 0.532894 | 0.457936 |
| LNC_000140 | XLOC_001418 | -- | intronic_lncRNA | 0.220712 | 0.346789 | 0.145678 | 0.992763 |
| LNC_000149 | XLOC_001520 | -- | intronic_lncRNA | 1.053223 | 0.226628 | 0.728112 | 0.952559 |
| LNC_000148 | XLOC_001498 | -- | intronic_lncRNA | 0.192482 | 0.220936 | 0.770757 | 1.211747 |
| LNC_005993 | XLOC_142527 | -- | intronic_lncRNA | 0.214507 | 0.642372 | 0.348409 | 0.429869 |
| LNC_005992 | XLOC_142523 | -- | intronic_lncRNA | 0.044182 | 0.098696 | 5.233321 | 0.026816 |
| LNC_005991 | XLOC_142523 | -- | intronic_lncRNA | 3.135305 | 0.00419 | 2.730511 | 5.4501 |
| LNC_005990 | XLOC_142512 | -- | intronic_lncRNA | 0 | 0.499994 | 0 | 0 |
| LNC_005997 | XLOC_142632 | -- | intronic_lncRNA | 0.501896 | 0.253964 | 0.686702 | 0.465547 |
| LNC_005996 | XLOC_142545 | -- | intronic_lncRNA | 0.242376 | 0 | 0.469713 | 0.483268 |
| LNC_005995 | XLOC_142529 | -- | intronic_lncRNA | 0.222126 | 0.542237 | 0.423793 | 0.626689 |
| LNC_005994 | XLOC_142528 | -- | intronic_lncRNA | 0.20718 | 0.277356 | 0.22799 | 0.677353 |
| LNC_001812 | XLOC_040713 | -- | intronic_lncRNA | 0.239048 | 0.063043 | 0.696741 | 0.537014 |
| LNC_001813 | XLOC_040802 | -- | intronic_lncRNA | 1.182437 | 0.391244 | 0.704452 | 0.646861 |
| LNC_001810 | XLOC_040705 | -- | intronic_lncRNA | 0.258674 | 0 | 0.38895 | 0 |
| LNC_001811 | XLOC_040713 | -- | intronic_lncRNA | 0.250188 | 0.268501 | 0.333627 | 0.437739 |
| LNC_001816 | XLOC_040815 | -- | intronic_lncRNA | 0.991142 | 0.507467 | 0.742685 | 0.470167 |
| LNC_001817 | XLOC_040846 | -- | intronic_lncRNA | 0.486101 | 1.010037 | 0.355803 | 0.653775 |
| LNC_001814 | XLOC_040802 | -- | intronic_lncRNA | 0.437044 | 0.158175 | 0.709423 | 0.501176 |
| LNC_001815 | XLOC_040812 | -- | antisense_lncRNA | 0.87667 | 0.129112 | 0 | 0 |
| LNC_001818 | XLOC_040912 | -- | lincRNA | 0.127477 | 0.254383 | 0.076569 | 0.411614 |
| LNC_001819 | XLOC_040917 | -- | intronic_lncRNA | 0.375087 | 0.418588 | 0.538661 | 0 |
| LNC_004734 | XLOC_105744 | -- | intronic_lncRNA | 0.127421 | 0.499084 | 0.516615 | 0 |
| LNC_004735 | XLOC_105745 | -- | intronic_lncRNA | 0 | 0.10538 | 0.580192 | 0.543858 |
| LNC_004736 | XLOC_105756 | -- | intronic_lncRNA | 0 | 0.079683 | 0 | 0.42776 |
| LNC_004737 | XLOC_105757 | -- | intronic_lncRNA | 0 | 0 | 0.592075 | 0.024994 |
| LNC_004730 | XLOC_105519 | -- | intronic_lncRNA | 0.296528 | 0.306065 | 0.490369 | 0.123913 |
| LNC_004731 | XLOC_105613 | -- | lincRNA | 0.171014 | 0.075406 | 0.430283 | 0.478435 |
| LNC_004732 | XLOC_105661 | -- | intronic_lncRNA | 0.176215 | 0.058554 | 0.652115 | 0.938314 |
| LNC_004733 | XLOC_105691 | -- | intronic_lncRNA | 0.743725 | 0.823872 | 1.082386 | 0.951412 |
| LNC_004738 | XLOC_105777 | -- | lincRNA | 0.669892 | 0.377846 | 0.306002 | 0 |
| LNC_004739 | XLOC_105838 | -- | intronic_lncRNA | 0 | 0.356136 | 0.801275 | 0 |
| LNC_000855 | XLOC_018341 | -- | intronic_lncRNA | 0 | 0.349757 | 0.50945 | 0.40681 |
| LNC_000854 | XLOC_018323 | -- | intronic_lncRNA | 0.377013 | 0.793849 | 0.16086 | 0.309871 |
| LNC_000857 | XLOC_018355 | -- | intronic_lncRNA | 0.761999 | 0.564787 | 0.112193 | 0.212829 |
| LNC_000856 | XLOC_018354 | -- | intronic_lncRNA | 0.565115 | 0.308361 | 0.38963 | 0.566696 |
| LNC_000851 | XLOC_018306 | -- | intronic_lncRNA | 0.240677 | 0.203506 | 0.981452 | 0.700328 |
| LNC_000850 | XLOC_018293 | -- | intronic_lncRNA | 0.920539 | 0.908107 | 0.579362 | 0.567904 |
| LNC_000853 | XLOC_018315 | -- | intronic_lncRNA | 0.680874 | 0.211164 | 0.272796 | 0.555696 |
| LNC_000852 | XLOC_018313 | -- | intronic_lncRNA | 0.516121 | 0.134485 | 0.427012 | 0.212376 |
| LNC_000859 | XLOC_018403 | -- | intronic_lncRNA | 0.003129 | 0.259561 | 0.529782 | 0.7256 |
| LNC_000858 | XLOC_018403 | -- | intronic_lncRNA | 0.600269 | 0 | 2.342556 | 1.909606 |
| LNC_000259 | XLOC_003721 | -- | intronic_lncRNA | 0.732285 | 0.169007 | 0.278716 | 0.238352 |
| LNC_003787 | XLOC_081094 | -- | lincRNA | 0 | 0 | 0.603125 | 0 |
| LNC_006468 | XLOC_155648 | -- | intronic_lncRNA | 0.606339 | 0.261757 | 0.340207 | 0.282108 |
| LNC_006469 | XLOC_155649 | -- | intronic_lncRNA | 0 | 0.54205 | 0.451422 | 0.228232 |
| LNC_006466 | XLOC_155480 | -- | intronic_lncRNA | 1.145937 | 0.584667 | 1.219061 | 0.94479 |
| LNC_006467 | XLOC_155647 | -- | intronic_lncRNA | 0.12712 | 0.245358 | 0.730747 | 0.932782 |
| LNC_006464 | XLOC_155460 | -- | lincRNA | 0.135147 | 0.398035 | 0.579356 | 0.144209 |
| LNC_006465 | XLOC_155472 | -- | intronic_lncRNA | 0.242896 | 0.154305 | 0.691543 | 0.518718 |
| LNC_006462 | XLOC_155401 | -- | intronic_lncRNA | 0.074001 | 0.19492 | 0.216675 | 0.450328 |
| LNC_006463 | XLOC_155430 | -- | lincRNA | 0.545697 | 0.33077 | 0.262176 | 0.395081 |
| LNC_006460 | XLOC_155376 | -- | intronic_lncRNA | 2.931718 | 2.983088 | 3.690462 | 4.775902 |

| LNC_006461 | XLOC_155396 | -- | intronic_lncRNA | 1.898067 | 1.257481 | 2.336564 | 2.998318 |
| --- | --- | --- | --- | --- | --- | --- | --- |
| LNC_000709 | XLOC_014079 | -- | intronic_lncRNA | 0.621269 | 0.372615 | 0.835976 | 1.401025 |
| LNC_000708 | XLOC_014075 | -- | lincRNA | 0.748233 | 0.501704 | 0.17825 | 0.553666 |
| LNC_000705 | XLOC_014029 | -- | lincRNA | 0.488707 | 0.307523 | 0.675375 | 1.857503 |
| LNC_000704 | XLOC_014028 | -- | intronic_lncRNA | 0.657066 | 0.628289 | 0.36664 | 0.88524 |
| LNC_000707 | XLOC_014034 | -- | intronic_lncRNA | 0.536534 | 0.183542 | 0.926048 | 0.34083 |
| LNC_000706 | XLOC_014029 | -- | lincRNA | 0.974055 | 0.141449 | 0 | 0 |
| LNC_000701 | XLOC_013941 | -- | antisense_lncRNA | 0.524405 | 0.188264 | 0.081125 | 0 |
| LNC_000700 | XLOC_013940 | -- | intronic_lncRNA | 0.488715 | 0.615144 | 0 | 0.595727 |
| LNC_000703 | XLOC_014027 | -- | intronic_lncRNA | 0.856416 | 0.294603 | 0.227119 | 0.035829 |
| LNC_000702 | XLOC_014027 | -- | intronic_lncRNA | 0.117913 | 0.495835 | 0.297308 | 0.700106 |
| LNC_005373 | XLOC_123539 | -- | intronic_lncRNA | 1.622775 | 0.688015 | 1.146221 | 0.263604 |
| LNC_002569 | XLOC_059686 | -- | intronic_lncRNA | 0.692375 | 0 | 0.059015 | 0.482912 |
| LNC_002568 | XLOC_059676 | -- | intronic_lncRNA | 0.275069 | 0.069314 | 0.41584 | 0.176603 |
| LNC_002567 | XLOC_059565 | -- | intronic_lncRNA | 2.612791 | 2.349881 | 5.062421 | 5.746733 |
| LNC_002566 | XLOC_059478 | -- | intronic_lncRNA | 0.213565 | 0 | 0.298182 | 0.656816 |
| LNC_002565 | XLOC_059430 | -- | lincRNA | 0.593964 | 0.254837 | 0.201449 | 0.234024 |
| LNC_002564 | XLOC_059425 | -- | lincRNA | 0.60498 | 0.157148 | 0.40279 | 0.324688 |
| LNC_002563 | XLOC_059306 | -- | intronic_lncRNA | 1.449244 | 0.31296 | 0.603344 | 0.595891 |
| LNC_002562 | XLOC_059268 | -- | intronic_lncRNA | 1.027534 | 0.8651 | 0.974557 | 0.730944 |
| LNC_002561 | XLOC_059265 | -- | intronic_lncRNA | 0.159172 | 0 | 0.497731 | 0.181561 |
| LNC_002560 | XLOC_059228 | -- | intronic_lncRNA | 0.193117 | 0.316615 | 0.340197 | 0.93141 |
| LNC_005746 | XLOC_134772 | -- | intronic_lncRNA | 0.763575 | 0.414119 | 0.837935 | 0.789552 |
| LNC_000250 | XLOC_003540 | -- | intronic_lncRNA | 0.56827 | 0.362127 | 0.961266 | 0.767591 |
| LNC_003179 | XLOC_073647 | -- | intronic_lncRNA | 0.935264 | 0.451564 | 1.121178 | 0.451966 |
| LNC_003178 | XLOC_073647 | -- | intronic_lncRNA | 0.630338 | 0.476085 | 0.48283 | 0.385588 |
| LNC_003177 | XLOC_073642 | -- | intronic_lncRNA | 1.202446 | 0.656458 | 1.773005 | 2.078725 |
| LNC_005377 | XLOC_123545 | -- | intronic_lncRNA | 0.939017 | 1.601241 | 0.909544 | 0.584868 |
| LNC_003175 | XLOC_073538 | -- | intronic_lncRNA | 0.260128 | 0.125792 | 0.410229 | 0 |
| LNC_003174 | XLOC_073537 | -- | intronic_lncRNA | 0.902393 | 0.351678 | 0 | 0.102899 |
| LNC_003173 | XLOC_073500 | -- | intronic_lncRNA | 0.966664 | 0.302855 | 1.089628 | 1.105462 |
| LNC_003172 | XLOC_073482 | -- | antisense_lncRNA | 1.048506 | 0.748535 | 0.469922 | 0.135887 |
| LNC_003171 | XLOC_073476 | -- | lincRNA | 0.947446 | 0 | 0 | 0.638051 |
| LNC_003170 | XLOC_073449 | -- | intronic_lncRNA | 0.076336 | 1.306918 | 0.382816 | 0.352224 |
| LNC_005745 | XLOC_134675 | -- | intronic_lncRNA | 1.288465 | 0.685519 | 0.441845 | 0.380258 |
| LNC_000255 | XLOC_003570 | -- | intronic_lncRNA | 1.390517 | 2.07788 | 1.657065 | 1.900007 |
| LNC_005742 | XLOC_134634 | -- | intronic_lncRNA | 1.029569 | 0.416359 | 0.147245 | 0.22083 |
| LNC_000254 | XLOC_003569 | -- | intronic_lncRNA | 0.364279 | 0 | 0.149169 | 0.426938 |
| LNC_005743 | XLOC_134658 | -- | intronic_lncRNA | 1.555338 | 0.80315 | 0.776043 | 0.803033 |
| LNC_001302 | XLOC_028547 | -- | intronic_lncRNA | 0.461737 | 0.329276 | 0.624667 | 0.855046 |
| LNC_005740 | XLOC_134593 | -- | intronic_lncRNA | 0.997066 | 0 | 0.436821 | 0.479583 |
| LNC_001303 | XLOC_028552 | -- | intronic_lncRNA | 0.354185 | 0.445115 | 1.384958 | 0.995848 |
| LNC_005741 | XLOC_134594 | -- | intronic_lncRNA | 0.412267 | 0.645808 | 0.420113 | 0.515892 |
| LNC_001300 | XLOC_028529 | -- | intronic_lncRNA | 1.814407 | 1.058369 | 0.971198 | 1.248394 |
| LNC_001301 | XLOC_028536 | -- | intronic_lncRNA | 0.105027 | 0 | 0 | 0.862175 |
| LNC_001306 | XLOC_028577 | -- | intronic_lncRNA | 0.230172 | 0.71012 | 0.451647 | 0.091849 |
| LNC_001307 | XLOC_028580 | -- | intronic_lncRNA | 0.549343 | 0.547536 | 0.85714 | 1.935279 |
| LNC_006288 | XLOC_150773 | -- | lincRNA | 0.441185 | 1.454548 | 0.293291 | 0.47445 |
| LNC_006289 | XLOC_150781 | -- | intronic_lncRNA | 0.521945 | 0.521297 | 0.08294 | 0.351025 |
| LNC_001304 | XLOC_028557 | -- | antisense_lncRNA | 0.360471 | 0.682731 | 0.510324 | 0.600344 |
| LNC_006282 | XLOC_150613 | -- | intronic_lncRNA | 0.97117 | 0.626535 | 0.71654 | 0.674055 |
| LNC_006283 | XLOC_150646 | -- | intronic_lncRNA | 0.619595 | 0 | 0 | 0 |
| LNC_006280 | XLOC_150585 | -- | intronic_lncRNA | 0 | 0.01314 | 0.542984 | 0.807621 |
| LNC_001305 | XLOC_028576 | -- | intronic_lncRNA | 0.239884 | 0.332248 | 0.720838 | 0.52274 |
| LNC_006286 | XLOC_150766 | -- | intronic_lncRNA | 0.064273 | 0.191684 | 0.083065 | 0.536089 |
| LNC_006287 | XLOC_150771 | -- | lincRNA | 0.354928 | 0.535095 | 0.041285 | 0 |
| LNC_006284 | XLOC_150679 | -- | lincRNA | 0.357605 | 0.348021 | 0.431154 | 0.411321 |
| LNC_006285 | XLOC_150683 | -- | lincRNA | 0 | 0.574251 | 0 | 0 |
| LNC_006277 | XLOC_150540 | -- | intronic_lncRNA | 0.853313 | 0.839883 | 0.538995 | 0.381804 |
| LNC_006276 | XLOC_150540 | -- | intronic_lncRNA | 2.416068 | 0.224896 | 0.744077 | 0.437567 |
| LNC_006275 | XLOC_150540 | -- | intronic_lncRNA | 0.703798 | 1.646033 | 0.444767 | 0.050413 |
| LNC_006274 | XLOC_150540 | -- | intronic_lncRNA | 0.944116 | 0.419887 | 1.648508 | 1.146825 |
| LNC_006273 | XLOC_150540 | -- | intronic_lncRNA | 3.363881 | 2.361592 | 2.940029 | 2.033407 |
| LNC_006272 | XLOC_150497 | -- | intronic_lncRNA | 0 | 0.070645 | 0.651266 | 0.974729 |
| LNC_001038 | XLOC_022484 | -- | intronic_lncRNA | 0.533299 | 0.380834 | 0 | 0 |
| LNC_001039 | XLOC_022485 | -- | intronic_lncRNA | 0.512155 | 0.1269 | 0 | 0 |
| LNC_001034 | XLOC_022403 | -- | intronic_lncRNA | 0.503737 | 0.89568 | 1.157287 | 1.263167 |
| LNC_001035 | XLOC_022410 | -- | intronic_lncRNA | 1.164749 | 1.074272 | 1.216668 | 0.22577 |
| LNC_001036 | XLOC_022410 | -- | intronic_lncRNA | 0 | 0 | 0 | 1.354819 |
| LNC_001037 | XLOC_022422 | -- | lincRNA | 0.577665 | 0.327235 | 2.310003 | 1.701992 |
| LNC_001030 | XLOC_022398 | -- | intronic_lncRNA | 0.160058 | 0.76346 | 0.193581 | 0.133794 |
| LNC_001031 | XLOC_022399 | -- | intronic_lncRNA | 15.928288 | 23.350338 | 21.500902 | 23.900511 |
| LNC_001032 | XLOC_022401 | -- | intronic_lncRNA | 0.749643 | 0.805338 | 0.837402 | 0.203905 |
| LNC_001033 | XLOC_022403 | -- | intronic_lncRNA | 0.795439 | 0.027557 | 1.418698 | 0.460385 |
| LNC_004284 | XLOC_093338 | -- | intronic_lncRNA | 0.787537 | 0.411817 | 0.306841 | 0.877528 |
| LNC_004285 | XLOC_093355 | -- | intronic_lncRNA | 0.329401 | 0.165712 | 0.402611 | 0.201271 |
| LNC_001159 | XLOC_025224 | -- | lincRNA | 0.418222 | 0.545483 | 0.703417 | 0.529425 |

| LNC_001158 | XLOC_025187 | -- | lincRNA | 1.12245 | 0.287653 | 0.134773 | 0.119366 |
| --- | --- | --- | --- | --- | --- | --- | --- |
| LNC_004280 | XLOC_092993 | -- | intronic_lncRNA | 0.78909 | 0 | 0 | 0.192669 |
| LNC_004281 | XLOC_093016 | -- | intronic_lncRNA | 0.173432 | 0.300294 | 0.836011 | 0.222737 |
| LNC_004282 | XLOC_093094 | -- | intronic_lncRNA | 0.356829 | 0.071261 | 0.353663 | 0.808627 |
| LNC_004283 | XLOC_093262 | -- | intronic_lncRNA | 0.507682 | 0.367245 | 0.478601 | 0.289162 |
| LNC_001153 | XLOC_024962 | -- | intronic_lncRNA | 1.682447 | 0.298471 | 1.299945 | 0.888046 |
| LNC_001152 | XLOC_024954 | -- | intronic_lncRNA | 0.164673 | 0.058207 | 0.478584 | 0.461649 |
| LNC_001151 | XLOC_024953 | -- | intronic_lncRNA | 0.553819 | 0.281445 | 0.574221 | 0.114824 |
| LNC_001150 | XLOC_024933 | -- | intronic_lncRNA | 0.402962 | 0.295683 | 0.552403 | 0.588494 |
| LNC_004288 | XLOC_093368 | -- | intronic_lncRNA | 1.036851 | 0.123844 | 0.162182 | 0.05782 |
| LNC_004289 | XLOC_093368 | -- | intronic_lncRNA | 0.628714 | 0.095045 | 0.040514 | 0.026308 |
| LNC_001155 | XLOC_024968 | -- | intronic_lncRNA | 0 | 0 | 0.474103 | 0.491441 |
| LNC_001154 | XLOC_024962 | -- | intronic_lncRNA | 1.584827 | 1.205278 | 1.258987 | 1.584135 |
| LNC_003883 | XLOC_083406 | -- | intronic_lncRNA | 0.227053 | 0.364924 | 0.530862 | 0.199351 |
| LNC_003882 | XLOC_083405 | -- | intronic_lncRNA | 1.690872 | 0.343297 | 1.129816 | 0.50268 |
| LNC_003881 | XLOC_083401 | -- | intronic_lncRNA | 0 | 0.224804 | 0.546178 | 0.273042 |
| LNC_003880 | XLOC_083400 | -- | intronic_lncRNA | 0 | 0 | 0 | 0.404855 |
| LNC_003887 | XLOC_083452 | -- | intronic_lncRNA | 0.368725 | 1.22518 | 1.184659 | 0.159358 |
| LNC_003886 | XLOC_083450 | -- | intronic_lncRNA | 1.036357 | 1.143185 | 1.430474 | 1.365193 |
| LNC_003885 | XLOC_083446 | -- | intronic_lncRNA | 0.243943 | 0.226173 | 0.149043 | 0.451569 |
| LNC_003884 | XLOC_083444 | -- | antisense_lncRNA | 0.598989 | 0 | 0.213637 | 0.069165 |
| LNC_003889 | XLOC_083628 | -- | intronic_lncRNA | 0 | 0.656712 | 0.200311 | 0.214192 |
| LNC_003888 | XLOC_083546 | -- | intronic_lncRNA | 0.522867 | 0.070506 | 0.06053 | 0.121039 |
| LNC_000288 | XLOC_004229 | -- | intronic_lncRNA | 0.598759 | 1.297843 | 0.535079 | 1.02102 |
| LNC_003774 | XLOC_080684 | -- | intronic_lncRNA | 0.858602 | 0.340044 | 0 | 0 |
| LNC_003777 | XLOC_080806 | -- | intronic_lncRNA | 0 | 0 | 1.108168 | 0 |
| LNC_003776 | XLOC_080744 | -- | intronic_lncRNA | 0.818296 | 0.746742 | 0.507718 | 0.437439 |
| LNC_003771 | XLOC_080661 | -- | intronic_lncRNA | 0.678932 | 0 | 0 | 0.992372 |
| LNC_003770 | XLOC_080661 | -- | intronic_lncRNA | 0.869466 | 0.228568 | 0.56422 | 0.626713 |
| LNC_003773 | XLOC_080675 | -- | intronic_lncRNA | 0 | 0.000733 | 0.454121 | 0.04274 |
| LNC_003772 | XLOC_080671 | -- | intronic_lncRNA | 0.600513 | 1.038555 | 0.278852 | 0.840938 |
| LNC_000280 | XLOC_004027 | -- | lincRNA | 1.22106 | 2.80407 | 0.427426 | 0.99757 |
| LNC_000281 | XLOC_004072 | -- | antisense_lncRNA | 0.48894 | 0.089272 | 0.46399 | 0.936822 |
| LNC_000282 | XLOC_004090 | -- | intronic_lncRNA | 0.858173 | 0.735237 | 0.324074 | 0.555126 |
| LNC_000283 | XLOC_004113 | -- | lincRNA | 0 | 0 | 0.391427 | 0 |
| LNC_003779 | XLOC_080808 | -- | intronic_lncRNA | 0.295316 | 0.579312 | 0.337796 | 0.455651 |
| LNC_003778 | XLOC_080808 | -- | intronic_lncRNA | 0.839854 | 0.804733 | 0.740872 | 0.880374 |
| LNC_000286 | XLOC_004155 | -- | antisense_lncRNA | 0.867861 | 0 | 0 | 0 |
| LNC_000287 | XLOC_004201 | -- | lincRNA | 0.888658 | 1.568322 | 1.514965 | 1.797436 |
| LNC_002828 | XLOC_065419 | -- | lincRNA | 0.297157 | 0.664761 | 0.253926 | 0.206718 |
| LNC_002829 | XLOC_065419 | -- | lincRNA | 0.503976 | 0 | 0 | 0 |
| LNC_002822 | XLOC_065386 | -- | lincRNA | 0.057369 | 0.037381 | 0 | 0.83364 |
| LNC_002823 | XLOC_065387 | -- | lincRNA | 2.554782 | 2.087475 | 0.496822 | 0.898698 |
| LNC_002820 | XLOC_065382 | -- | lincRNA | 1.292842 | 0.340327 | 0.331483 | 0.402277 |
| LNC_002821 | XLOC_065386 | -- | lincRNA | 1.330899 | 0.22226 | 0 | 0 |
| LNC_002826 | XLOC_065395 | -- | lincRNA | 3.345642 | 3.31986 | 0.23367 | 1.365829 |
| LNC_002827 | XLOC_065408 | -- | lincRNA | 0.788284 | 0.023986 | 0 | 0.545767 |
| LNC_002824 | XLOC_065391 | -- | lincRNA | 0.961688 | 0.381389 | 0.070505 | 1.068298 |
| LNC_002825 | XLOC_065391 | -- | lincRNA | 0.90499 | 0.152242 | 0.354012 | 0.668117 |
| LNC_003410 | XLOC_075199 | -- | lincRNA | 1.469175 | 0.049337 | 0 | 0 |
| LNC_003411 | XLOC_075199 | -- | lincRNA | 1.536597 | 0.386219 | 0.186277 | 0.370524 |
| LNC_003412 | XLOC_075200 | -- | lincRNA | 8.883965 | 5.810302 | 2.856723 | 3.027343 |
| LNC_003413 | XLOC_075201 | -- | lincRNA | 2.789025 | 1.564315 | 0.55495 | 1.312793 |
| LNC_003414 | XLOC_075202 | -- | lincRNA | 1.991201 | 0.682284 | 0.735187 | 0.364597 |
| LNC_003415 | XLOC_075203 | -- | lincRNA | 3.067774 | 1.079975 | 0.479769 | 0.340505 |
| LNC_003416 | XLOC_075206 | -- | lincRNA | 0 | 1.666212 | 0 | 0 |
| LNC_003417 | XLOC_075206 | -- | lincRNA | 3.687798 | 2.347609 | 1.133426 | 0.457452 |
| LNC_003418 | XLOC_075206 | -- | lincRNA | 1.240704 | 0 | 0 | 0 |
| LNC_003419 | XLOC_075206 | -- | lincRNA | 2.551462 | 0.506958 | 0.633969 | 0.418337 |
| LNC_006071 | XLOC_145122 | -- | lincRNA | 0.186458 | 0.174571 | 0.653335 | 0.594484 |
| LNC_006070 | XLOC_145089 | -- | intronic_lncRNA | 0.854505 | 0.729361 | 0.794796 | 0.775589 |
| LNC_006073 | XLOC_145188 | -- | antisense_lncRNA | 2.085853 | 0.067145 | 0.312737 | 0.501307 |
| LNC_006072 | XLOC_145156 | -- | lincRNA | 0.612344 | 0.488608 | 0.134904 | 0.322893 |
| LNC_006075 | XLOC_145266 | -- | intronic_lncRNA | 0 | 0 | 0.167795 | 0.462899 |
| LNC_006074 | XLOC_145203 | -- | intronic_lncRNA | 0.669031 | 0.603636 | 0.217285 | 0.214945 |
| LNC_006077 | XLOC_145269 | -- | intronic_lncRNA | 0.994054 | 3.217918 | 1.66516 | 2.39651 |
| LNC_006076 | XLOC_145267 | -- | intronic_lncRNA | 0 | 0 | 0.284244 | 0.243934 |
| LNC_006079 | XLOC_145305 | -- | lincRNA | 0.422974 | 0.343785 | 0.974478 | 0.679547 |
| LNC_006078 | XLOC_145301 | -- | intronic_lncRNA | 0 | 0.487062 | 0.100797 | 0 |
| LNC_004503 | XLOC_098756 | -- | intronic_lncRNA | 1.369288 | 1.241891 | 1.748995 | 1.673581 |
| LNC_004502 | XLOC_098750 | -- | intronic_lncRNA | 0.104175 | 0 | 0.462978 | 0 |
| LNC_004501 | XLOC_098699 | -- | intronic_lncRNA | 2.297934 | 0.96949 | 0.139887 | 0.217636 |
| LNC_004500 | XLOC_098699 | -- | intronic_lncRNA | 11.005509 | 4.827232 | 1.366239 | 1.975689 |
| LNC_004507 | XLOC_098802 | -- | intronic_lncRNA | 0.347764 | 0.108141 | 0.281639 | 0.694526 |
| LNC_004506 | XLOC_098777 | -- | intronic_lncRNA | 0.269785 | 0 | 0.435766 | 0.777871 |
| LNC_004505 | XLOC_098774 | -- | intronic_lncRNA | 0.560649 | 0.510354 | 0.731633 | 1.035605 |
| LNC_004504 | XLOC_098772 | -- | intronic_lncRNA | 0.508422 | 0 | 0.684712 | 0.677292 |

| LNC_004509 | XLOC_098881 | -- | intronic_lncRNA | 0 | 0 | 0 | 0.500768 |
| --- | --- | --- | --- | --- | --- | --- | --- |
| LNC_004508 | XLOC_098830 | -- | intronic_lncRNA | 0.246193 | 0.127846 | 0.336525 | 0.361903 |
| LNC_005049 | XLOC_115258 | -- | lincRNA | 0.554127 | 0.067793 | 0 | 0 |
| LNC_005048 | XLOC_115257 | -- | lincRNA | 0.609751 | 0 | 0 | 0 |
| LNC_005041 | XLOC_115099 | -- | intronic_lncRNA | 0.585741 | 0.376907 | 0 | 0 |
| LNC_005040 | XLOC_115091 | -- | intronic_lncRNA | 0 | 0.154665 | 0.626284 | 0.124818 |
| LNC_005043 | XLOC_115100 | -- | intronic_lncRNA | 0 | 0.108988 | 0 | 0.740837 |
| LNC_005042 | XLOC_115100 | -- | intronic_lncRNA | 0.534637 | 0.237299 | 0.13484 | 0.033624 |
| LNC_005045 | XLOC_115110 | -- | lincRNA | 0 | 0.487389 | 0 | 0 |
| LNC_005044 | XLOC_115103 | -- | intronic_lncRNA | 19.619501 | 41.643669 | 29.484444 | 23.064459 |
| LNC_005047 | XLOC_115197 | -- | intronic_lncRNA | 0.104637 | 0.262598 | 0.399755 | 0.696787 |
| LNC_005046 | XLOC_115192 | -- | intronic_lncRNA | 0.927911 | 1.192163 | 1.789273 | 1.661189 |
| LNC_000518 | XLOC_009176 | -- | intronic_lncRNA | 0.179412 | 0.064444 | 0.721483 | 0.441125 |
| LNC_000519 | XLOC_009195 | -- | intronic_lncRNA | 0 | 0 | 0.651539 | 0.012991 |
| LNC_000510 | XLOC_009026 | -- | intronic_lncRNA | 0.111749 | 0.666317 | 0.42227 | 0.717471 |
| LNC_000511 | XLOC_009026 | -- | intronic_lncRNA | 2.265705 | 1.952379 | 1.470776 | 1.387984 |
| LNC_005032 | XLOC_114844 | -- | intronic_lncRNA | 0.544757 | 0.494522 | 0.583099 | 0.162638 |
| LNC_005033 | XLOC_114844 | -- | intronic_lncRNA | 0 | 0 | 0.013478 | 0.502292 |
| LNC_005034 | XLOC_114846 | -- | intronic_lncRNA | 0.161688 | 0.570368 | 0.099793 | 0.26359 |
| LNC_000515 | XLOC_009042 | -- | intronic_lncRNA | 4.033277 | 5.871003 | 1.740274 | 2.057355 |
| LNC_005036 | XLOC_114956 | -- | intronic_lncRNA | 2.036124 | 1.064876 | 3.159278 | 2.778287 |
| LNC_000517 | XLOC_009112 | -- | intronic_lncRNA | 0.542744 | 0.836678 | 0.962491 | 0.671298 |
| LNC_003960 | XLOC_085212 | -- | intronic_lncRNA | 0.274111 | 0.195974 | 0.80505 | 0.387999 |
| LNC_003961 | XLOC_085306 | -- | intronic_lncRNA | 0.733528 | 0.499395 | 0.806888 | 0.72542 |
| LNC_003962 | XLOC_085348 | -- | lincRNA | 0.153824 | 0 | 0 | 0.603909 |
| LNC_003963 | XLOC_085357 | -- | lincRNA | 0.15093 | 0.240263 | 0.435797 | 0.349321 |
| LNC_003964 | XLOC_085436 | -- | intronic_lncRNA | 0.391445 | 0 | 0.079076 | 0.395313 |
| LNC_003965 | XLOC_085571 | -- | intronic_lncRNA | 0.42746 | 0.292878 | 0.792504 | 0.461311 |
| LNC_003966 | XLOC_085572 | -- | intronic_lncRNA | 0.711965 | 0.110135 | 1.371231 | 0.896663 |
| LNC_003967 | XLOC_085573 | -- | intronic_lncRNA | 0 | 0.107009 | 0.179112 | 0.753787 |
| LNC_003968 | XLOC_085573 | -- | intronic_lncRNA | 0 | 0.506303 | 0 | 0 |
| LNC_003969 | XLOC_085615 | -- | intronic_lncRNA | 0 | 0.31075 | 0.653691 | 0 |
| LNC_002611 | XLOC_060327 | -- | intronic_lncRNA | 0.52572 | 0 | 0.234856 | 0.701009 |
| LNC_002610 | XLOC_060272 | -- | intronic_lncRNA | 0.912218 | 0.023138 | 0.823029 | 1.823012 |
| LNC_002613 | XLOC_060341 | -- | intronic_lncRNA | 0 | 0 | 0 | 0.666484 |
| LNC_002612 | XLOC_060341 | -- | intronic_lncRNA | 2.288511 | 0.944342 | 4.366127 | 2.746408 |
| LNC_002615 | XLOC_060369 | -- | intronic_lncRNA | 0.005947 | 0.148336 | 0.022664 | 0.500759 |
| LNC_002614 | XLOC_060368 | -- | intronic_lncRNA | 0.619895 | 0 | 0.51213 | 1.122626 |
| LNC_002617 | XLOC_060461 | -- | intronic_lncRNA | 0 | 0 | 0.398062 | 0.086559 |
| LNC_002616 | XLOC_060369 | -- | intronic_lncRNA | 0.238129 | 0 | 0.39496 | 0.400782 |
| LNC_002619 | XLOC_060518 | -- | intronic_lncRNA | 0.254718 | 0.108399 | 0.067601 | 0.361744 |
| LNC_002618 | XLOC_060518 | -- | intronic_lncRNA | 0.647151 | 0.38811 | 0.447455 | 0.386112 |
| LNC_004349 | XLOC_094664 | -- | intronic_lncRNA | 0.143677 | 0 | 0.508091 | 0.23159 |
| LNC_004348 | XLOC_094651 | -- | intronic_lncRNA | 0.653958 | 0 | 0.493438 | 0.135659 |
| LNC_004341 | XLOC_094396 | -- | intronic_lncRNA | 0.13176 | 0 | 0 | 1.096411 |
| LNC_004340 | XLOC_094357 | -- | lincRNA | 0.893075 | 0.047577 | 0 | 0 |
| LNC_004343 | XLOC_094466 | -- | intronic_lncRNA | 0.421577 | 0.315531 | 0.5672 | 0.746537 |
| LNC_004342 | XLOC_094465 | -- | intronic_lncRNA | 0 | 0.133746 | 0.520746 | 0.971891 |
| LNC_004345 | XLOC_094495 | -- | intronic_lncRNA | 0.172654 | 0.555484 | 0.379913 | 0.561105 |
| LNC_004344 | XLOC_094471 | -- | intronic_lncRNA | 1.540025 | 0.875077 | 0.997649 | 1.076421 |
| LNC_004347 | XLOC_094510 | -- | intronic_lncRNA | 0.538268 | 0.196861 | 1.202941 | 1.016583 |
| LNC_004346 | XLOC_094497 | -- | intronic_lncRNA | 2.001794 | 0.434791 | 0.310846 | 0.292566 |
| LNC_001834 | XLOC_041168 | -- | intronic_lncRNA | 0 | 0.088145 | 0 | 0.711347 |
| LNC_001835 | XLOC_041176 | -- | intronic_lncRNA | 0.234079 | 0.622812 | 0.567438 | 0.378227 |
| LNC_001836 | XLOC_041181 | -- | intronic_lncRNA | 1.056704 | 0.226206 | 0.293112 | 0 |
| LNC_001837 | XLOC_041182 | -- | intronic_lncRNA | 0.735591 | 0.513759 | 0.087709 | 0.434844 |
| LNC_001830 | XLOC_041134 | -- | antisense_lncRNA | 0.378917 | 0.779091 | 0.852897 | 1.278288 |
| LNC_001831 | XLOC_041145 | -- | intronic_lncRNA | 0.72662 | 0.50689 | 0 | 0.240523 |
| LNC_001832 | XLOC_041154 | -- | intronic_lncRNA | 0.281592 | 0.565473 | 0.169588 | 0.690622 |
| LNC_001833 | XLOC_041155 | -- | intronic_lncRNA | 0.347743 | 0.483489 | 0.391819 | 0.403774 |
| LNC_001838 | XLOC_041183 | -- | intronic_lncRNA | 1.393713 | 0.649309 | 0.098937 | 0.128902 |
| LNC_001839 | XLOC_041183 | -- | intronic_lncRNA | 0 | 1.561508 | 0.740265 | 0.000163 |
| LNC_004758 | XLOC_106418 | -- | intronic_lncRNA | 0.136454 | 0.679533 | 0.081998 | 0.048632 |
| LNC_004759 | XLOC_106475 | -- | intronic_lncRNA | 0.69415 | 0.365455 | 1.054375 | 1.124177 |
| LNC_004756 | XLOC_106416 | -- | intronic_lncRNA | 0.230673 | 0.219867 | 0.545824 | 0.162756 |
| LNC_004757 | XLOC_106417 | -- | intronic_lncRNA | 0.893557 | 0 | 0.191498 | 0.388951 |
| LNC_004754 | XLOC_106393 | -- | intronic_lncRNA | 1.049111 | 0.954791 | 0.422767 | 0.451708 |
| LNC_004755 | XLOC_106399 | -- | intronic_lncRNA | 0.614521 | 0.812464 | 0.318516 | 0.44403 |
| LNC_004752 | XLOC_106272 | -- | intronic_lncRNA | 0 | 0.132018 | 0.245721 | 0.634312 |
| LNC_004753 | XLOC_106355 | -- | intronic_lncRNA | 0.546191 | 0 | 0 | 0 |
| LNC_004750 | XLOC_106269 | -- | intronic_lncRNA | 0.585989 | 0.314467 | 0.483438 | 0.564286 |
| LNC_004751 | XLOC_106270 | -- | intronic_lncRNA | 0.064882 | 0 | 0.787759 | 0.177291 |
| LNC_006000 | XLOC_142652 | -- | intronic_lncRNA | 0.808928 | 0.807491 | 1.011684 | 1.727187 |
| LNC_006001 | XLOC_142653 | -- | intronic_lncRNA | 0.373317 | 0.356239 | 0.609991 | 0.178794 |
| LNC_006101 | XLOC_145984 | -- | intronic_lncRNA | 1.447336 | 1.705668 | 1.266813 | 1.702343 |
| LNC_006100 | XLOC_145971 | -- | lincRNA | 0.862004 | 0.118426 | 0.219806 | 0 |
| LNC_006103 | XLOC_146009 | -- | intronic_lncRNA | 0.325606 | 0.569303 | 0.504197 | 1.050033 |

| LNC_006102 | XLOC_145984 | -- | intronic_lncRNA | 10.620545 | 8.245876 | 6.293523 | 3.269788 |
| --- | --- | --- | --- | --- | --- | --- | --- |
| LNC_006105 | XLOC_146034 | -- | intronic_lncRNA | 0.792967 | 0.445514 | 0.59102 | 0.550393 |
| LNC_006104 | XLOC_146034 | -- | intronic_lncRNA | 0.086448 | 0 | 0.108976 | 0 |
| LNC_006008 | XLOC_142787 | -- | intronic_lncRNA | 0.271877 | 0 | 0.982482 | 0.498984 |
| LNC_006107 | XLOC_146036 | -- | intronic_lncRNA | 1.097142 | 0.119095 | 0.343684 | 0.419326 |
| LNC_006009 | XLOC_142814 | -- | intronic_lncRNA | 0.447169 | 0.277841 | 0.340861 | 0.757972 |
| LNC_006106 | XLOC_146035 | -- | intronic_lncRNA | 0.731874 | 0.423808 | 1.115039 | 0.749718 |
| LNC_006440 | XLOC_154376 | -- | intronic_lncRNA | 0.529933 | 0.70992 | 0.722703 | 1.355726 |
| LNC_006441 | XLOC_154376 | -- | intronic_lncRNA | 1.136583 | 0.640369 | 0.44997 | 0.050077 |
| LNC_006442 | XLOC_154954 | -- | intronic_lncRNA | 0.157067 | 0.665913 | 0.559968 | 0.41896 |
| LNC_006443 | XLOC_154957 | -- | intronic_lncRNA | 0.27649 | 0.14517 | 0.372561 | 0.474477 |
| LNC_006444 | XLOC_154979 | -- | intronic_lncRNA | 0 | 0 | 0 | 0.888086 |
| LNC_006445 | XLOC_154991 | -- | antisense_lncRNA | 3.164109 | 1.879562 | 0.740062 | 0.490763 |
| LNC_000879 | XLOC_019126 | -- | intronic_lncRNA | 0.464619 | 0.519422 | 0.515071 | 0.388504 |
| LNC_000878 | XLOC_019125 | -- | intronic_lncRNA | 0.290385 | 0.118205 | 0 | 0.567046 |
| LNC_000877 | XLOC_019025 | -- | lincRNA | 0.889266 | 0.14962 | 0 | 0 |
| LNC_000876 | XLOC_018939 | -- | lincRNA | 0 | 0.412347 | 0.575009 | 0.44386 |
| LNC_000875 | XLOC_018811 | -- | intronic_lncRNA | 0.12317 | 0.209272 | 0.112518 | 0.650036 |
| LNC_000874 | XLOC_018811 | -- | intronic_lncRNA | 0.223957 | 0.09059 | 0.60207 | 0 |
| LNC_000873 | XLOC_018747 | -- | intronic_lncRNA | 0.562337 | 0.285642 | 0.505562 | 0.219345 |
| LNC_000872 | XLOC_018709 | -- | lincRNA | 0.758212 | 0 | 0 | 0.225287 |
| LNC_000871 | XLOC_018691 | -- | intronic_lncRNA | 0.692055 | 0.697408 | 0.383055 | 0.57547 |
| LNC_000870 | XLOC_018614 | -- | intronic_lncRNA | 0.830418 | 0.370249 | 0.704377 | 1.129736 |
| LNC_000727 | XLOC_014815 | -- | intronic_lncRNA | 0.548384 | 0.757599 | 0.234329 | 0.930275 |
| LNC_000726 | XLOC_014685 | -- | intronic_lncRNA | 0.626397 | 0.626104 | 1.39969 | 0.707596 |
| LNC_000725 | XLOC_014590 | -- | intronic_lncRNA | 0.635082 | 0.414785 | 0.543357 | 0.041655 |
| LNC_000724 | XLOC_014590 | -- | intronic_lncRNA | 0.071847 | 0.867866 | 0.018567 | 0.486893 |
| LNC_000723 | XLOC_014524 | -- | intronic_lncRNA | 1.399859 | 0.946823 | 1.578204 | 0.87084 |
| LNC_000722 | XLOC_014502 | -- | intronic_lncRNA | 1.796034 | 1.262786 | 1.432899 | 1.657175 |
| LNC_000721 | XLOC_014434 | -- | intronic_lncRNA | 0.14264 | 0.644282 | 0.011945 | 0.018791 |
| LNC_000720 | XLOC_014429 | -- | intronic_lncRNA | 0.991558 | 0.717481 | 0.804252 | 1.071421 |
| LNC_005939 | XLOC_140307 | -- | intronic_lncRNA | 0.37129 | 0.748506 | 0.963413 | 0.750604 |
| LNC_005938 | XLOC_140285 | -- | intronic_lncRNA | 0.544696 | 0.790824 | 0.602793 | 0.58004 |
| LNC_000729 | XLOC_014835 | -- | lincRNA | 0.589458 | 0 | 0 | 0.051119 |
| LNC_000728 | XLOC_014816 | -- | intronic_lncRNA | 0.82205 | 0.379896 | 1.766903 | 0.749454 |
| LNC_002581 | XLOC_059881 | -- | intronic_lncRNA | 0.604147 | 0.230391 | 0.581494 | 0.789709 |
| LNC_002580 | XLOC_059836 | -- | intronic_lncRNA | 0.085452 | 0.208132 | 0.649507 | 0.496128 |
| LNC_002583 | XLOC_059887 | -- | intronic_lncRNA | 0.630018 | 0 | 0 | 0.144071 |
| LNC_002582 | XLOC_059881 | -- | intronic_lncRNA | 0.236941 | 0.349029 | 0.373085 | 0.896034 |
| LNC_002585 | XLOC_059936 | -- | intronic_lncRNA | 1.02496 | 0.631593 | 0.981669 | 0.615208 |
| LNC_002584 | XLOC_059888 | -- | intronic_lncRNA | 0 | 0 | 0.506712 | 0 |
| LNC_002587 | XLOC_059954 | -- | intronic_lncRNA | 0.021137 | 0 | 0.534703 | 0.075595 |
| LNC_002586 | XLOC_059940 | -- | intronic_lncRNA | 0.735624 | 0.37754 | 0.314168 | 0.47948 |
| LNC_002589 | XLOC_059955 | -- | intronic_lncRNA | 0.367831 | 0.133475 | 0.854782 | 0.14213 |
| LNC_002588 | XLOC_059954 | -- | intronic_lncRNA | 1.343141 | 0.973956 | 1.100945 | 1.73744 |
| LNC_001439 | XLOC_031291 | -- | antisense_lncRNA | 0.576533 | 0.207209 | 0 | 0.085137 |
| LNC_006421 | XLOC_153987 | -- | antisense_lncRNA | 0.322492 | 0.28425 | 0.411006 | 0.457114 |
| LNC_003191 | XLOC_073655 | -- | lincRNA | 63.421543 | 0 | 0.088579 | 1.305063 |
| LNC_003190 | XLOC_073655 | -- | lincRNA | 44.083336 | 0 | 0 | 0 |
| LNC_003193 | XLOC_073680 | -- | intronic_lncRNA | 0.23098 | 0.200523 | 2.173138 | 4.524982 |
| LNC_003192 | XLOC_073656 | -- | intronic_lncRNA | 0.119523 | 0 | 0.567501 | 0.307156 |
| LNC_003195 | XLOC_073719 | -- | lincRNA | 0.627305 | 0.065648 | 0.070362 | 0 |
| LNC_003194 | XLOC_073680 | -- | intronic_lncRNA | 0.465474 | 0.476806 | 0.9039 | 1.320779 |
| LNC_003197 | XLOC_073804 | -- | lincRNA | 0.672797 | 0 | 0 | 0 |
| LNC_003196 | XLOC_073779 | -- | intronic_lncRNA | 1.068502 | 0.906641 | 0.251347 | 0.655713 |
| LNC_003199 | XLOC_073851 | -- | intronic_lncRNA | 0.67831 | 0.14109 | 0.347421 | 0.363725 |
| LNC_003198 | XLOC_073847 | -- | intronic_lncRNA | 0.578404 | 0 | 0.221225 | 0 |
| LNC_000597 | XLOC_011287 | -- | intronic_lncRNA | 0 | 0.783158 | 0 | 0.048424 |
| LNC_001588 | XLOC_034441 | -- | intronic_lncRNA | 0 | 0.591761 | 0.543687 | 0 |
| LNC_001589 | XLOC_034442 | -- | intronic_lncRNA | 0.36489 | 0.157607 | 0.642239 | 0.382852 |
| LNC_001584 | XLOC_034420 | -- | intronic_lncRNA | 0.099844 | 0.522141 | 0.230824 | 0.321198 |
| LNC_001585 | XLOC_034425 | -- | lincRNA | 0.385537 | 0.26295 | 0.763993 | 0.499846 |
| LNC_001586 | XLOC_034438 | -- | intronic_lncRNA | 0.633639 | 0.194629 | 0.416091 | 0.275528 |
| LNC_001587 | XLOC_034440 | -- | intronic_lncRNA | 0 | 0.722256 | 0.104381 | 0.280598 |
| LNC_001580 | XLOC_034316 | -- | intronic_lncRNA | 0.673249 | 0.338078 | 0.801382 | 1.218966 |
| LNC_001581 | XLOC_034360 | -- | intronic_lncRNA | 0.592149 | 0.179124 | 0.304037 | 0.40584 |
| LNC_001582 | XLOC_034384 | -- | intronic_lncRNA | 5.811047 | 6.186833 | 8.408284 | 8.516163 |
| LNC_001583 | XLOC_034411 | -- | intronic_lncRNA | 0.956735 | 0.810393 | 1.014894 | 1.081843 |
| LNC_005458 | XLOC_125960 | -- | intronic_lncRNA | 0.424793 | 0.048914 | 0.191662 | 0.498668 |
| LNC_005459 | XLOC_126000 | -- | intronic_lncRNA | 0.667324 | 0.451821 | 0.654487 | 0.772327 |
| LNC_005456 | XLOC_125743 | -- | intronic_lncRNA | 1.576823 | 0.653402 | 1.574647 | 0.951487 |
| LNC_005457 | XLOC_125941 | -- | intronic_lncRNA | 0.114204 | 0 | 0.546614 | 0.276797 |
| LNC_005454 | XLOC_125708 | -- | intronic_lncRNA | 0.25936 | 0 | 0.560182 | 0.140126 |
| LNC_001056 | XLOC_022846 | -- | intronic_lncRNA | 0.540741 | 0.225703 | 0 | 0 |
| LNC_001057 | XLOC_022851 | -- | intronic_lncRNA | 0.57163 | 0.209224 | 0.552395 | 0.771528 |
| LNC_001054 | XLOC_022823 | -- | intronic_lncRNA | 0.230275 | 0.479165 | 0 | 0.429864 |
| LNC_001055 | XLOC_022832 | -- | intronic_lncRNA | 2.718387 | 1.710508 | 2.787535 | 2.553002 |

| LNC_001052 | XLOC_022769 | -- | intronic_lncRNA | 0.125233 | 0.332272 | 0.592747 | 0.829062 |
| --- | --- | --- | --- | --- | --- | --- | --- |
| LNC_001053 | XLOC_022805 | -- | intronic_lncRNA | 0.854469 | 1.038136 | 0.659692 | 1.014127 |
| LNC_001050 | XLOC_022726 | -- | lincRNA | 0.03781 | 0.054409 | 0.436454 | 0.116679 |
| LNC_001051 | XLOC_022758 | -- | intronic_lncRNA | 0.404139 | 0.552374 | 0.761978 | 0.349333 |
| LNC_005452 | XLOC_125706 | -- | intronic_lncRNA | 1.443231 | 0.73413 | 0.448653 | 1.035387 |
| LNC_001058 | XLOC_022892 | -- | intronic_lncRNA | 0 | 0.78026 | 0 | 0 |
| LNC_001059 | XLOC_022915 | -- | intronic_lncRNA | 0.299569 | 0.559902 | 0.644002 | 0.490341 |
| LNC_005450 | XLOC_125679 | -- | lincRNA | 0.096231 | 0.273886 | 0.614019 | 0.934567 |
| LNC_005451 | XLOC_125706 | -- | intronic_lncRNA | 2.200275 | 1.452699 | 1.763533 | 1.803999 |
| LNC_001139 | XLOC_024814 | -- | lincRNA | 0.729661 | 0 | 0 | 0 |
| LNC_001138 | XLOC_024809 | -- | intronic_lncRNA | 0.597388 | 0 | 0.301867 | 0.047701 |
| LNC_001135 | XLOC_024808 | -- | intronic_lncRNA | 1.315372 | 0.572524 | 1.10441 | 0.975004 |
| LNC_001134 | XLOC_024807 | -- | lincRNA | 0.552448 | 0.074958 | 0 | 0 |
| LNC_001137 | XLOC_024809 | -- | intronic_lncRNA | 0.305797 | 0.608105 | 0.036587 | 0.196214 |
| LNC_001136 | XLOC_024809 | -- | intronic_lncRNA | 0.006292 | 0.09777 | 0.097175 | 0.508957 |
| LNC_001131 | XLOC_024752 | -- | intronic_lncRNA | 0.420058 | 0.204861 | 0.408256 | 0.453187 |
| LNC_001130 | XLOC_024745 | -- | intronic_lncRNA | 0 | 0.520827 | 0.403575 | 0.318486 |
| LNC_001133 | XLOC_024790 | -- | lincRNA | 0 | 0 | 0.455433 | 0 |
| LNC_001132 | XLOC_024762 | -- | intronic_lncRNA | 0.700953 | 0.383346 | 0.46076 | 0.472914 |
| LNC_003759 | XLOC_080447 | -- | intronic_lncRNA | 0.587038 | 0.63403 | 0.53265 | 0.376953 |
| LNC_003758 | XLOC_080446 | -- | intronic_lncRNA | 0.116259 | 0.289997 | 0.352285 | 0.234816 |
| LNC_003757 | XLOC_080445 | -- | intronic_lncRNA | 0.122452 | 0.586773 | 0.301593 | 0.137394 |
| LNC_003756 | XLOC_080398 | -- | intronic_lncRNA | 0.082896 | 0.069474 | 0 | 0.464519 |
| LNC_003755 | XLOC_080390 | -- | intronic_lncRNA | 1.534497 | 0.207883 | 0.117396 | 0.336654 |
| LNC_003754 | XLOC_080366 | -- | intronic_lncRNA | 0.689429 | 0.257195 | 1.729727 | 1.480648 |
| LNC_003753 | XLOC_080353 | -- | lincRNA | 0 | 0 | 0.240535 | 0.850447 |
| LNC_003752 | XLOC_080352 | -- | lincRNA | 0 | 0 | 0.512414 | 0 |
| LNC_003751 | XLOC_080344 | -- | intronic_lncRNA | 0.875445 | 0.807075 | 0.832852 | 0.877178 |
| LNC_003750 | XLOC_080339 | -- | intronic_lncRNA | 0.048124 | 0.041375 | 0.835412 | 1.177931 |
| LNC_005342 | XLOC_122294 | -- | lincRNA | 0.659874 | 0.146073 | 0.763678 | 0.205992 |
| LNC_005343 | XLOC_122424 | -- | intronic_lncRNA | 0 | 0.328378 | 0.314869 | 0.690635 |
| LNC_000260 | XLOC_003746 | -- | intronic_lncRNA | 0.809966 | 0.13084 | 0 | 0.258798 |
| LNC_005341 | XLOC_122267 | -- | lincRNA | 0.382438 | 0.433015 | 0.306024 | 0.429537 |
| LNC_005346 | XLOC_122629 | -- | intronic_lncRNA | 1.079422 | 0 | 0.433385 | 0.311544 |
| LNC_005347 | XLOC_122674 | -- | intronic_lncRNA | 0.293408 | 0 | 0.900629 | 0 |
| LNC_005344 | XLOC_122427 | -- | intronic_lncRNA | 0.068823 | 0 | 0.360398 | 0.748303 |
| LNC_005345 | XLOC_122604 | -- | lincRNA | 0 | 0.513268 | 0 | 0 |
| LNC_005348 | XLOC_122751 | -- | intronic_lncRNA | 0.576175 | 0.137972 | 0.724227 | 0.103443 |
| LNC_005349 | XLOC_122788 | -- | intronic_lncRNA | 0.22716 | 0.194512 | 0.692154 | 0.369201 |
| LNC_002808 | XLOC_065252 | -- | intronic_lncRNA | 0.156617 | 0.617697 | 0.455174 | 0.251588 |
| LNC_002809 | XLOC_065292 | -- | intronic_lncRNA | 0.635898 | 0.144637 | 0 | 0 |
| LNC_002804 | XLOC_065224 | -- | intronic_lncRNA | 0 | 2.628566 | 1.57021 | 0 |
| LNC_002805 | XLOC_065230 | -- | intronic_lncRNA | 0.356344 | 0.073944 | 0.736379 | 0 |
| LNC_002806 | XLOC_065238 | -- | intronic_lncRNA | 0.116603 | 0.831454 | 0 | 0.147553 |
| LNC_002807 | XLOC_065248 | -- | lincRNA | 0 | 0 | 0.075312 | 0.717549 |
| LNC_002800 | XLOC_065196 | -- | lincRNA | 0.594214 | 0 | 0 | 0.122958 |
| LNC_002801 | XLOC_065218 | -- | intronic_lncRNA | 0 | 0.61921 | 0.143198 | 0.749851 |
| LNC_002802 | XLOC_065222 | -- | intronic_lncRNA | 29.994694 | 55.844879 | 47.035397 | 58.626537 |
| LNC_002803 | XLOC_065224 | -- | intronic_lncRNA | 1.274973 | 2.444998 | 0 | 2.690594 |
| LNC_005694 | XLOC_133265 | -- | lincRNA | 0 | 0.199196 | 0.490021 | 0 |
| LNC_005695 | XLOC_133282 | -- | intronic_lncRNA | 1.762443 | 2.098078 | 1.488916 | 0.637096 |
| LNC_005696 | XLOC_133305 | -- | intronic_lncRNA | 0.766816 | 0.489204 | 0.745259 | 1.078678 |
| LNC_005697 | XLOC_133306 | -- | intronic_lncRNA | 0.550614 | 0.531983 | 0.960371 | 0.66923 |
| LNC_005690 | XLOC_133176 | -- | intronic_lncRNA | 0.230909 | 0.095709 | 0.778357 | 0.294041 |
| LNC_005691 | XLOC_133176 | -- | intronic_lncRNA | 0.402016 | 0 | 0.233791 | 0.48129 |
| LNC_005692 | XLOC_133241 | -- | intronic_lncRNA | 0.491173 | 0.44601 | 0.785039 | 0 |
| LNC_005693 | XLOC_133242 | -- | intronic_lncRNA | 2.021763 | 0.425758 | 0.66908 | 0.512498 |
| LNC_005698 | XLOC_133386 | -- | intronic_lncRNA | 1.967293 | 1.906604 | 1.083258 | 1.696102 |
| LNC_005699 | XLOC_133386 | -- | intronic_lncRNA | 0.723748 | 1.047449 | 0.610418 | 1.207787 |
| LNC_006059 | XLOC_144390 | -- | intronic_lncRNA | 0.450656 | 0.327957 | 0.903616 | 1.0314 |
| LNC_006058 | XLOC_144343 | -- | intronic_lncRNA | 0.051103 | 0.558748 | 0.648077 | 0.336465 |
| LNC_006053 | XLOC_144267 | -- | lincRNA | 0.806337 | 0 | 0 | 0 |
| LNC_006052 | XLOC_144115 | -- | intronic_lncRNA | 0.276266 | 0.590414 | 0.46622 | 0.901319 |
| LNC_006051 | XLOC_144114 | -- | intronic_lncRNA | 0.233787 | 0.059335 | 0.706286 | 0.598419 |
| LNC_006050 | XLOC_144114 | -- | intronic_lncRNA | 0.143386 | 0.485825 | 0.052969 | 0.403342 |
| LNC_006057 | XLOC_144342 | -- | intronic_lncRNA | 0.4666 | 0.510346 | 0.412705 | 0.293801 |
| LNC_006056 | XLOC_144318 | -- | intronic_lncRNA | 0 | 0.046618 | 0.437337 | 0.636789 |
| LNC_006055 | XLOC_144302 | -- | intronic_lncRNA | 0 | 0 | 0.398062 | 0 |
| LNC_006054 | XLOC_144294 | -- | intronic_lncRNA | 0.452178 | 0.288524 | 0.753385 | 0.344273 |
| LNC_004565 | XLOC_100308 | -- | intronic_lncRNA | 0.517037 | 0 | 0 | 0 |
| LNC_004564 | XLOC_100300 | -- | intronic_lncRNA | 0.292288 | 0.538791 | 0.327687 | 0.312232 |
| LNC_004567 | XLOC_100351 | -- | intronic_lncRNA | 0.876862 | 0.832425 | 1.425652 | 1.12799 |
| LNC_004566 | XLOC_100313 | -- | intronic_lncRNA | 0.401254 | 0.053855 | 0.362819 | 0.557438 |
| LNC_004561 | XLOC_100293 | -- | intronic_lncRNA | 0.345194 | 0.075526 | 0.472571 | 0.589532 |
| LNC_004560 | XLOC_100290 | -- | intronic_lncRNA | 2.42028 | 0.950129 | 0.908734 | 1.026014 |
| LNC_004563 | XLOC_100294 | -- | intronic_lncRNA | 0.907558 | 0.882651 | 0.609772 | 0.794173 |
| LNC_004562 | XLOC_100293 | -- | intronic_lncRNA | 0 | 0.486287 | 0 | 0.106996 |

| LNC_004569 | XLOC_100368 | -- | intronic_lncRNA | 0.569167 | 0.066012 | 0.073508 | 0 |
| --- | --- | --- | --- | --- | --- | --- | --- |
| LNC_004568 | XLOC_100354 | -- | intronic_lncRNA | 0.129702 | 1.13654 | 0.122308 | 0.26727 |
| LNC_005586 | XLOC_130055 | -- | intronic_lncRNA | 0.851297 | 0.613503 | 0.787988 | 0.868893 |
| LNC_002248 | XLOC_051510 | -- | intronic_lncRNA | 0.027489 | 0.363557 | 0.097087 | 0.487507 |
| LNC_002249 | XLOC_051512 | -- | intronic_lncRNA | 0.107955 | 0.067017 | 0.510557 | 0 |
| LNC_002242 | XLOC_051422 | -- | intronic_lncRNA | 0.533112 | 0.292269 | 0.584874 | 0.543068 |
| LNC_002243 | XLOC_051423 | -- | intronic_lncRNA | 0.323369 | 0.734969 | 0.537663 | 0.709904 |
| LNC_002240 | XLOC_051373 | -- | antisense_lncRNA | 0.05623 | 0.098659 | 0 | 0.529487 |
| LNC_002241 | XLOC_051378 | -- | intronic_lncRNA | 0.233452 | 0.229047 | 0.471599 | 0 |
| LNC_002246 | XLOC_051491 | -- | intronic_lncRNA | 0.078111 | 0.156235 | 0.237435 | 0.509382 |
| LNC_002247 | XLOC_051509 | -- | intronic_lncRNA | 0.204347 | 0 | 0.801558 | 0.08436 |
| LNC_002244 | XLOC_051435 | -- | lincRNA | 0 | 0 | 0.615344 | 0 |
| LNC_002245 | XLOC_051436 | -- | intronic_lncRNA | 0.703251 | 0.501769 | 0.738455 | 0.840194 |
| LNC_003038 | XLOC_069892 | -- | lincRNA | 0.38484 | 0.161158 | 0.619866 | 0.728523 |
| LNC_003039 | XLOC_069940 | -- | intronic_lncRNA | 0.973845 | 0.544753 | 0.568091 | 0.786853 |
| LNC_003032 | XLOC_069474 | -- | intronic_lncRNA | 0.462052 | 0.646728 | 0.57729 | 0.65481 |
| LNC_003033 | XLOC_069490 | -- | intronic_lncRNA | 0.13112 | 0.610228 | 0.087518 | 0.070621 |
| LNC_003030 | XLOC_069367 | -- | intronic_lncRNA | 0.26303 | 0.524881 | 0.380446 | 0.179919 |
| LNC_003031 | XLOC_069474 | -- | intronic_lncRNA | 0.760824 | 0.599183 | 1.051027 | 0.755062 |
| LNC_003036 | XLOC_069679 | -- | lincRNA | 0.902239 | 0.178966 | 0 | 0.457515 |
| LNC_003037 | XLOC_069879 | -- | intronic_lncRNA | 0 | 0.468582 | 0.398062 | 0.270635 |
| LNC_003034 | XLOC_069537 | -- | intronic_lncRNA | 0.470305 | 0 | 0.286793 | 0.303969 |
| LNC_003035 | XLOC_069543 | -- | intronic_lncRNA | 0.991355 | 0.93224 | 0.570646 | 0.241986 |
| LNC_005108 | XLOC_116893 | -- | intronic_lncRNA | 1.199023 | 0.740989 | 0.942416 | 0.689324 |
| LNC_000469 | XLOC_008292 | -- | intronic_lncRNA | 0.042735 | 0.462376 | 0.662793 | 0.666891 |
| LNC_004987 | XLOC_113653 | -- | intronic_lncRNA | 1.633623 | 1.445185 | 1.556582 | 1.705088 |
| LNC_000464 | XLOC_008233 | -- | intronic_lncRNA | 0.394577 | 0.671126 | 0.557573 | 0.504894 |
| LNC_004985 | XLOC_113641 | -- | intronic_lncRNA | 0.270718 | 0.098374 | 0.219466 | 0.495742 |
| LNC_004984 | XLOC_113445 | -- | intronic_lncRNA | 0.980123 | 0 | 0 | 0 |
| LNC_004983 | XLOC_113438 | -- | intronic_lncRNA | 0.998923 | 0.32625 | 0.370604 | 0.225871 |
| LNC_004982 | XLOC_113437 | -- | intronic_lncRNA | 0.530695 | 0.369514 | 0.499534 | 0.775931 |
| LNC_004981 | XLOC_113434 | -- | intronic_lncRNA | 0.602776 | 0.182306 | 0.495077 | 0 |
| LNC_000465 | XLOC_008234 | -- | intronic_lncRNA | 0 | 0.164046 | 0.582697 | 0.300794 |
| LNC_005106 | XLOC_116867 | -- | intronic_lncRNA | 0 | 0.789075 | 0.154482 | 0.714957 |
| LNC_004989 | XLOC_113670 | -- | intronic_lncRNA | 0.811919 | 0.353893 | 0.328236 | 0.643339 |
| LNC_004988 | XLOC_113666 | -- | intronic_lncRNA | 0.174647 | 0.424171 | 0 | 0.412186 |
| LNC_005107 | XLOC_116869 | -- | intronic_lncRNA | 0.110618 | 0.796134 | 0.421147 | 0.578364 |
| LNC_005100 | XLOC_116773 | -- | intronic_lncRNA | 0.565767 | 0.211291 | 0.353924 | 0.10549 |
| LNC_005101 | XLOC_116774 | -- | intronic_lncRNA | 0.327336 | 0.306875 | 0.424623 | 0 |
| LNC_002639 | XLOC_061014 | -- | lincRNA | 0.497048 | 0.575151 | 0 | 0 |
| LNC_002638 | XLOC_061011 | -- | lincRNA | 0.545818 | 0 | 0 | 0 |
| LNC_000462 | XLOC_008230 | -- | intronic_lncRNA | 0.197409 | 0.132057 | 0.340575 | 0.513205 |
| LNC_002633 | XLOC_060956 | -- | intronic_lncRNA | 0.176909 | 0 | 0.196456 | 0.474025 |
| LNC_002632 | XLOC_060920 | -- | intronic_lncRNA | 0.585168 | 0.599584 | 0.601625 | 0.408355 |
| LNC_002631 | XLOC_060909 | -- | intronic_lncRNA | 0.054947 | 0 | 0.440012 | 0.099706 |
| LNC_005103 | XLOC_116782 | -- | intronic_lncRNA | 0 | 1.87065 | 2.543177 | 1.168929 |
| LNC_002637 | XLOC_061002 | -- | lincRNA | 0.540741 | 0 | 0 | 0 |
| LNC_002636 | XLOC_060972 | -- | intronic_lncRNA | 0.323047 | 0.304669 | 0.358592 | 0.494536 |
| LNC_002635 | XLOC_060971 | -- | intronic_lncRNA | 0.339189 | 0.217682 | 0.08964 | 0.521212 |
| LNC_002634 | XLOC_060967 | -- | intronic_lncRNA | 0.210115 | 0.421031 | 0.82897 | 0.366447 |
| LNC_004363 | XLOC_095074 | -- | lincRNA | 0.098172 | 0.135489 | 0.359374 | 0.463432 |
| LNC_004362 | XLOC_095034 | -- | lincRNA | 0.625958 | 0.133585 | 0.356544 | 0.372234 |
| LNC_004361 | XLOC_094997 | -- | intronic_lncRNA | 0.016898 | 0.265841 | 0.034679 | 0.040319 |
| LNC_004360 | XLOC_094997 | -- | intronic_lncRNA | 0.488546 | 0.102198 | 0.330618 | 0.574214 |
| LNC_004367 | XLOC_095246 | -- | intronic_lncRNA | 0.233296 | 0.354551 | 0.392736 | 0 |
| LNC_004366 | XLOC_095233 | -- | intronic_lncRNA | 0.690574 | 0 | 0.009803 | 0 |
| LNC_004365 | XLOC_095233 | -- | intronic_lncRNA | 0 | 0.555281 | 0.880956 | 0.821251 |
| LNC_004364 | XLOC_095206 | -- | intronic_lncRNA | 0.149077 | 0.358098 | 0.128893 | 0.850724 |
| LNC_004369 | XLOC_095263 | -- | lincRNA | 0 | 0 | 0.611988 | 0.102094 |
| LNC_004368 | XLOC_095247 | -- | intronic_lncRNA | 0.36219 | 0.370012 | 0.673979 | 0.662378 |
| LNC_001858 | XLOC_041279 | -- | intronic_lncRNA | 0.170169 | 0.102099 | 0.463663 | 0.384944 |
| LNC_001859 | XLOC_041280 | -- | intronic_lncRNA | 0 | 0.298904 | 0.494294 | 0.862861 |
| LNC_001856 | XLOC_041262 | -- | intronic_lncRNA | 0.434247 | 0.476707 | 0.240218 | 0.321666 |
| LNC_001857 | XLOC_041278 | -- | intronic_lncRNA | 0.580824 | 0.228903 | 0.572123 | 0.343024 |
| LNC_001854 | XLOC_041254 | -- | intronic_lncRNA | 0.813206 | 0.393351 | 0.77202 | 0.678039 |
| LNC_001855 | XLOC_041255 | -- | intronic_lncRNA | 0.888704 | 0.58234 | 0.961759 | 0.389459 |
| LNC_001852 | XLOC_041240 | -- | intronic_lncRNA | 0 | 1.762165 | 1.591376 | 1.777708 |
| LNC_001853 | XLOC_041248 | -- | intronic_lncRNA | 0 | 0 | 0.424439 | 0.051988 |
| LNC_001850 | XLOC_041231 | -- | antisense_lncRNA | 0.658585 | 0.566682 | 0 | 0 |
| LNC_001851 | XLOC_041233 | -- | intronic_lncRNA | 0.48835 | 0.356526 | 0.61979 | 0.689607 |
| LNC_004770 | XLOC_106582 | -- | lincRNA | 3.111651 | 1.173755 | 1.60147 | 1.734723 |
| LNC_004771 | XLOC_106582 | -- | lincRNA | 0.902211 | 0 | 0.921639 | 1.546672 |
| LNC_004772 | XLOC_106582 | -- | lincRNA | 0.414222 | 0.424834 | 0 | 1.057 |
| LNC_004773 | XLOC_106688 | -- | intronic_lncRNA | 2.24995 | 0.649684 | 0.720661 | 0.687892 |
| LNC_004774 | XLOC_106694 | -- | antisense_lncRNA | 0.230679 | 0.119668 | 0.13352 | 0.48743 |
| LNC_004775 | XLOC_106705 | -- | intronic_lncRNA | 0.784148 | 1.086476 | 0.73761 | 0.23809 |
| LNC_004776 | XLOC_106712 | -- | intronic_lncRNA | 0 | 0 | 0.684283 | 0 |

| LNC_004777 | XLOC_106749 | -- | intronic_lncRNA | 0.104026 | 0.39372 | 0.607399 | 0.888502 |
| --- | --- | --- | --- | --- | --- | --- | --- |
| LNC_004778 | XLOC_106750 | -- | intronic_lncRNA | 0.539032 | 0.172079 | 0.787233 | 0.453697 |
| LNC_004779 | XLOC_106783 | -- | intronic_lncRNA | 1.425665 | 0.685181 | 0.079549 | 0.224211 |
| LNC_000099 | XLOC_000756 | -- | lincRNA | 1.077804 | 0 | 0 | 0.352681 |
| LNC_005578 | XLOC_129560 | -- | intronic_lncRNA | 7.541958 | 1.883293 | 0.644821 | 0.550838 |
| LNC_000091 | XLOC_000619 | -- | lincRNA | 9.017922 | 20.034824 | 5.728082 | 16.778442 |
| LNC_005570 | XLOC_129297 | -- | intronic_lncRNA | 0.228217 | 0.227895 | 0.195889 | 0.640323 |
| LNC_000093 | XLOC_000621 | -- | lincRNA | 7.979078 | 10.517551 | 12.622391 | 20.427977 |
| LNC_005572 | XLOC_129373 | -- | intronic_lncRNA | 0.493351 | 0.367624 | 0.791538 | 0.63663 |
| LNC_000095 | XLOC_000638 | -- | lincRNA | 1.259475 | 0 | 0.29357 | 0.508279 |
| LNC_000094 | XLOC_000622 | -- | lincRNA | 0.079237 | 1.586327 | 21.063509 | 16.231821 |
| LNC_000097 | XLOC_000727 | -- | lincRNA | 1.140357 | 0.239726 | 0 | 0.982196 |
| LNC_000096 | XLOC_000686 | -- | lincRNA | 0.236934 | 1.215941 | 0.098318 | 0.223414 |
| LNC_002031 | XLOC_045486 | -- | intronic_lncRNA | 0.793642 | 0 | 0.198127 | 0.321666 |
| LNC_002030 | XLOC_045470 | -- | intronic_lncRNA | 0.705859 | 0.378543 | 0.041506 | 0 |
| LNC_002033 | XLOC_045535 | -- | intronic_lncRNA | 0.035684 | 0.502494 | 0.480381 | 0.746106 |
| LNC_002032 | XLOC_045487 | -- | intronic_lncRNA | 0.113049 | 0 | 0.166327 | 0.716966 |
| LNC_002035 | XLOC_045563 | -- | intronic_lncRNA | 1.006179 | 0.542725 | 0.387089 | 0.46171 |
| LNC_002034 | XLOC_045542 | -- | intronic_lncRNA | 0.935531 | 1.14451 | 0.511465 | 0.589377 |
| LNC_002037 | XLOC_045730 | -- | intronic_lncRNA | 0.535931 | 0.076719 | 0.36264 | 0.326514 |
| LNC_002036 | XLOC_045592 | -- | intronic_lncRNA | 0.984941 | 1.047042 | 0.813127 | 0.513026 |
| LNC_002039 | XLOC_045735 | -- | intronic_lncRNA | 0.045848 | 0.500858 | 0.065441 | 0.632665 |
| LNC_002038 | XLOC_045732 | -- | intronic_lncRNA | 0.270828 | 0 | 0 | 0 |
| LNC_003201 | XLOC_073933 | -- | lincRNA | 0.170092 | 0.269689 | 0.427412 | 0.604013 |
| LNC_003200 | XLOC_073895 | -- | intronic_lncRNA | 1.647006 | 0.766294 | 0.454056 | 0.707294 |
| LNC_003203 | XLOC_073956 | -- | lincRNA | 0.349192 | 0 | 0 | 0.524803 |
| LNC_003202 | XLOC_073936 | -- | intronic_lncRNA | 0 | 0.094645 | 0.646834 | 0.223075 |
| LNC_003205 | XLOC_074038 | -- | intronic_lncRNA | 0.054788 | 0.273142 | 0.198333 | 0.597183 |
| LNC_003204 | XLOC_073986 | -- | intronic_lncRNA | 0.557267 | 0.402176 | 0.778857 | 0.738564 |
| LNC_003207 | XLOC_074075 | -- | intronic_lncRNA | 1.351521 | 0.230085 | 0.900865 | 0.833448 |
| LNC_003206 | XLOC_074042 | -- | intronic_lncRNA | 0 | 0 | 0.18582 | 0.516079 |
| LNC_003209 | XLOC_074213 | -- | intronic_lncRNA | 1.490629 | 0.888625 | 0.8744 | 0.886103 |
| LNC_003208 | XLOC_074088 | -- | intronic_lncRNA | 0 | 0 | 0.637619 | 0 |
| LNC_000891 | XLOC_019317 | -- | intronic_lncRNA | 30.348351 | 15.495729 | 26.216883 | 23.951555 |
| LNC_000890 | XLOC_019317 | -- | antisense_lncRNA | 0.410146 | 0.087456 | 0.4298 | 6.924263 |
| LNC_000893 | XLOC_019351 | -- | intronic_lncRNA | 0.56101 | 0.652634 | 0.935289 | 0.666614 |
| LNC_000892 | XLOC_019321 | -- | intronic_lncRNA | 0.571943 | 0.602679 | 0.846657 | 1.080207 |
| LNC_000895 | XLOC_019453 | -- | intronic_lncRNA | 0.136807 | 0.314298 | 0.831806 | 0.893122 |
| LNC_000894 | XLOC_019383 | -- | intronic_lncRNA | 0 | 0 | 0.287778 | 0.435982 |
| LNC_000897 | XLOC_019498 | -- | intronic_lncRNA | 0.763808 | 0.389599 | 0.492169 | 0.494586 |
| LNC_000896 | XLOC_019497 | -- | intronic_lncRNA | 0.299641 | 0.50373 | 0.136743 | 0.409475 |
| LNC_000899 | XLOC_019513 | -- | intronic_lncRNA | 0 | 0.330795 | 0.178598 | 0.399098 |
| LNC_000898 | XLOC_019498 | -- | intronic_lncRNA | 0.582053 | 0.218851 | 0.518976 | 1.0353 |
| LNC_006347 | XLOC_152226 | -- | intronic_lncRNA | 0.588492 | 0.51709 | 0.7221 | 0.563443 |
| LNC_006346 | XLOC_152226 | -- | intronic_lncRNA | 2.800044 | 1.570014 | 1.254421 | 0.871414 |
| LNC_006345 | XLOC_152226 | -- | intronic_lncRNA | 0.361845 | 1.205759 | 0.363983 | 0.245208 |
| LNC_006344 | XLOC_152210 | -- | intronic_lncRNA | 0.077778 | 4.166675 | 10.055834 | 9.487664 |
| LNC_001423 | XLOC_030959 | -- | intronic_lncRNA | 0.441004 | 0.351319 | 0.508363 | 1.019929 |
| LNC_001422 | XLOC_030956 | -- | intronic_lncRNA | 0.505475 | 0.884279 | 0.408446 | 0.084398 |
| LNC_006341 | XLOC_152177 | -- | intronic_lncRNA | 0.22633 | 3.002201 | 0.128277 | 0.429884 |
| LNC_006340 | XLOC_152177 | -- | intronic_lncRNA | 0 | 0 | 1.990934 | 0 |
| LNC_006439 | XLOC_154376 | -- | intronic_lncRNA | 1.874996 | 0 | 0.408875 | 0.227514 |
| LNC_006438 | XLOC_154371 | -- | intronic_lncRNA | 0.89343 | 0.405668 | 0.87306 | 1.094018 |
| LNC_001429 | XLOC_031220 | -- | intronic_lncRNA | 0.000563 | 0.518362 | 0.284681 | 0.630535 |
| LNC_001428 | XLOC_031202 | -- | intronic_lncRNA | 0.506368 | 0.503758 | 0.117587 | 0.66541 |
| LNC_005919 | XLOC_139513 | -- | intronic_lncRNA | 0 | 0.65755 | 0 | 0 |
| LNC_005918 | XLOC_139513 | -- | intronic_lncRNA | 0 | 0.548531 | 0 | 0 |
| LNC_005913 | XLOC_139470 | -- | intronic_lncRNA | 0 | 0 | 0 | 0.57422 |
| LNC_005912 | XLOC_139470 | -- | intronic_lncRNA | 0.488484 | 0.822875 | 0.216568 | 0.268457 |
| LNC_005911 | XLOC_139453 | -- | intronic_lncRNA | 0.611153 | 0.592706 | 0.453629 | 0.433917 |
| LNC_005910 | XLOC_139452 | -- | intronic_lncRNA | 0.628531 | 0.232552 | 0.244524 | 0.299519 |
| LNC_005917 | XLOC_139499 | -- | intronic_lncRNA | 0.715304 | 0.394927 | 0.716785 | 0.687897 |
| LNC_005916 | XLOC_139479 | -- | intronic_lncRNA | 0 | 0.685031 | 0 | 0 |
| LNC_005915 | XLOC_139478 | -- | intronic_lncRNA | 1.283245 | 0 | 0 | 0 |
| LNC_005914 | XLOC_139478 | -- | intronic_lncRNA | 0.657386 | 0.617839 | 0.827552 | 1.023717 |
| LNC_001078 | XLOC_023255 | -- | intronic_lncRNA | 0 | 0 | 0.543617 | 0.006101 |
| LNC_001079 | XLOC_023299 | -- | intronic_lncRNA | 0.282046 | 0.710006 | 0.273486 | 1.056614 |
| LNC_001070 | XLOC_023082 | -- | antisense_lncRNA | 0.382023 | 0.974224 | 0.094004 | 0.00276 |
| LNC_001071 | XLOC_023082 | -- | antisense_lncRNA | 0.832841 | 0.265354 | 1.43274 | 1.366579 |
| LNC_001072 | XLOC_023083 | -- | intronic_lncRNA | 0 | 0 | 0.042322 | 1.098584 |
| LNC_001073 | XLOC_023086 | -- | intronic_lncRNA | 1.105578 | 0.452684 | 1.081452 | 1.078324 |
| LNC_001074 | XLOC_023111 | -- | intronic_lncRNA | 0 | 0 | 0.587801 | 0.162718 |
| LNC_001075 | XLOC_023204 | -- | lincRNA | 0.216363 | 0.70546 | 0.159064 | 0.300858 |
| LNC_001076 | XLOC_023204 | -- | lincRNA | 0.228378 | 1.092444 | 0.411057 | 0.543035 |
| LNC_001077 | XLOC_023255 | -- | intronic_lncRNA | 0.752113 | 0.613583 | 0.308255 | 0.979493 |
| LNC_001117 | XLOC_024258 | -- | intronic_lncRNA | 0.348136 | 0.274229 | 0.122518 | 0 |
| LNC_001116 | XLOC_024245 | -- | intronic_lncRNA | 0.531728 | 0.5651 | 0.647767 | 0.613434 |

| LNC_001115 | XLOC_024216 | -- | intronic_lncRNA | 0 | 0 | 0.303694 | 0.400469 |
| --- | --- | --- | --- | --- | --- | --- | --- |
| LNC_001114 | XLOC_024213 | -- | antisense_lncRNA | 0 | 0.406794 | 0.814267 | 0.641066 |
| LNC_001113 | XLOC_024174 | -- | intronic_lncRNA | 0 | 0 | 0.146022 | 0.629506 |
| LNC_001112 | XLOC_023949 | -- | intronic_lncRNA | 0.411507 | 0 | 0.111859 | 0.42069 |
| LNC_001111 | XLOC_023948 | -- | intronic_lncRNA | 0.784009 | 0.02103 | 0.071115 | 0.053327 |
| LNC_001110 | XLOC_023826 | -- | intronic_lncRNA | 0.277237 | 0.207635 | 0.934568 | 0.356614 |
| LNC_001119 | XLOC_024288 | -- | lincRNA | 1.071575 | 0 | 0.053376 | 0 |
| LNC_001118 | XLOC_024274 | -- | intronic_lncRNA | 0.750351 | 0.342187 | 0.702617 | 0.593437 |
| LNC_000309 | XLOC_004461 | -- | intronic_lncRNA | 4.362719 | 4.173087 | 3.715926 | 3.996912 |
| LNC_000308 | XLOC_004455 | -- | intronic_lncRNA | 2.183215 | 1.234129 | 2.353141 | 2.820564 |
| LNC_000301 | XLOC_004304 | -- | intronic_lncRNA | 0.698519 | 0.711007 | 0.971216 | 1.186263 |
| LNC_000300 | XLOC_004303 | -- | intronic_lncRNA | 0.841898 | 0.707334 | 0.86033 | 1.041663 |
| LNC_000303 | XLOC_004319 | -- | intronic_lncRNA | 0.341122 | 0.338407 | 0.087121 | 0.429538 |
| LNC_000302 | XLOC_004319 | -- | intronic_lncRNA | 0.239653 | 0.572834 | 0.06738 | 0.050365 |
| LNC_000305 | XLOC_004439 | -- | intronic_lncRNA | 158.188431 | 150.467224 | 0.41515 | 1.295522 |
| LNC_000304 | XLOC_004320 | -- | intronic_lncRNA | 0.938621 | 0.438119 | 0 | 0.490762 |
| LNC_000307 | XLOC_004452 | -- | intronic_lncRNA | 0.199002 | 0.447871 | 0.453391 | 0.332429 |
| LNC_000306 | XLOC_004449 | -- | intronic_lncRNA | 0.202333 | 0.272911 | 0.484852 | 0.193703 |
| LNC_002798 | XLOC_065149 | -- | intronic_lncRNA | 0.679084 | 0 | 0 | 0 |
| LNC_002799 | XLOC_065194 | -- | intronic_lncRNA | 1.676464 | 2.148196 | 1.686067 | 1.981391 |
| LNC_002794 | XLOC_065017 | -- | intronic_lncRNA | 1.281377 | 1.540323 | 1.000391 | 1.23938 |
| LNC_002795 | XLOC_065027 | -- | intronic_lncRNA | 0.822366 | 0.837949 | 0.462215 | 0.703492 |
| LNC_002796 | XLOC_065069 | -- | intronic_lncRNA | 0 | 0.139723 | 0.622833 | 0 |
| LNC_002797 | XLOC_065104 | -- | intronic_lncRNA | 0 | 0.84465 | 0 | 0 |
| LNC_002790 | XLOC_064888 | -- | intronic_lncRNA | 0.372714 | 0 | 0.005312 | 0 |
| LNC_002791 | XLOC_064917 | -- | intronic_lncRNA | 0.628797 | 0.126775 | 0.906743 | 0 |
| LNC_002792 | XLOC_064944 | -- | intronic_lncRNA | 0 | 0.200603 | 0.339708 | 0.847145 |
| LNC_002793 | XLOC_064953 | -- | intronic_lncRNA | 0.614045 | 0.806467 | 0.572807 | 0.334581 |
| LNC_005364 | XLOC_123271 | -- | intronic_lncRNA | 0.231963 | 0 | 0 | 0.589144 |
| LNC_000245 | XLOC_003406 | -- | intronic_lncRNA | 1.332674 | 1.688244 | 0.852834 | 0.853146 |
| LNC_005366 | XLOC_123278 | -- | intronic_lncRNA | 0.279347 | 0 | 0 | 0.359046 |
| LNC_005367 | XLOC_123286 | -- | intronic_lncRNA | 0.67211 | 0.25477 | 0.54491 | 0.108963 |
| LNC_000240 | XLOC_003336 | -- | intronic_lncRNA | 1.186649 | 0.71208 | 0.658547 | 0.38063 |
| LNC_005361 | XLOC_123204 | -- | intronic_lncRNA | 0.069707 | 0 | 0.042544 | 1.588377 |
| LNC_000242 | XLOC_003355 | -- | intronic_lncRNA | 0.854214 | 0 | 0 | 0 |
| LNC_000243 | XLOC_003358 | -- | intronic_lncRNA | 0 | 0.253459 | 0.592733 | 0.068954 |
| LNC_005368 | XLOC_123288 | -- | intronic_lncRNA | 0.257515 | 0 | 0.406678 | 0 |
| LNC_000249 | XLOC_003513 | -- | intronic_lncRNA | 0.839331 | 1.110936 | 1.291323 | 0.849754 |
| LNC_002866 | XLOC_066067 | -- | intronic_lncRNA | 0.514246 | 1.065091 | 1.188815 | 1.203219 |
| LNC_002867 | XLOC_066068 | -- | intronic_lncRNA | 0.190475 | 0.380553 | 0.912481 | 0.971614 |
| LNC_002864 | XLOC_066060 | -- | intronic_lncRNA | 0.481101 | 1.46692 | 0.299091 | 0.200697 |
| LNC_002865 | XLOC_066067 | -- | intronic_lncRNA | 0.736631 | 0.038337 | 0.095693 | 0.298629 |
| LNC_002862 | XLOC_065940 | -- | intronic_lncRNA | 0.276257 | 0.135445 | 0.158219 | 0.437957 |
| LNC_002863 | XLOC_065944 | -- | intronic_lncRNA | 0 | 0 | 0.347292 | 0.780694 |
| LNC_002860 | XLOC_065844 | -- | lincRNA | 0.474404 | 1.021945 | 0.461811 | 0.132816 |
| LNC_002861 | XLOC_065890 | -- | intronic_lncRNA | 0.959954 | 0.545201 | 0.47817 | 0.696802 |
| LNC_002868 | XLOC_066110 | -- | intronic_lncRNA | 0.575015 | 0 | 0.172579 | 0 |
| LNC_002869 | XLOC_066159 | -- | intronic_lncRNA | 0 | 0.611073 | 0 | 0 |
| LNC_004095 | XLOC_088611 | -- | intronic_lncRNA | 0.525171 | 0.199998 | 0.566895 | 0.161942 |
| LNC_004094 | XLOC_088610 | -- | intronic_lncRNA | 0.536031 | 0.597029 | 0.189618 | 0.622005 |
| LNC_004097 | XLOC_088627 | -- | intronic_lncRNA | 0.507682 | 0 | 0.186654 | 0.148066 |
| LNC_004096 | XLOC_088612 | -- | intronic_lncRNA | 0.221982 | 0.910319 | 0.81941 | 0.83045 |
| LNC_004091 | XLOC_088576 | -- | intronic_lncRNA | 1.037984 | 0.971567 | 0.962568 | 0.932665 |
| LNC_004090 | XLOC_088570 | -- | intronic_lncRNA | 0.304896 | 0 | 0.03166 | 0.681426 |
| LNC_004093 | XLOC_088608 | -- | intronic_lncRNA | 0 | 0 | 0.194252 | 0.48717 |
| LNC_004092 | XLOC_088578 | -- | intronic_lncRNA | 1.27118 | 1.716692 | 2.020663 | 1.191769 |
| LNC_004099 | XLOC_088646 | -- | intronic_lncRNA | 0 | 0 | 0.776988 | 0 |
| LNC_004098 | XLOC_088643 | -- | intronic_lncRNA | 0 | 0 | 0.665014 | 0.105401 |
| LNC_000938 | XLOC_020154 | -- | intronic_lncRNA | 0.576666 | 0.720033 | 0.491047 | 0.368531 |
| LNC_000939 | XLOC_020158 | -- | intronic_lncRNA | 0.388148 | 0.723622 | 0.264796 | 0.519245 |
| LNC_006039 | XLOC_143759 | -- | intronic_lncRNA | 0.211744 | 0.553441 | 0 | 0 |
| LNC_006038 | XLOC_143719 | -- | intronic_lncRNA | 0 | 0.459784 | 0.522381 | 0 |
| LNC_000932 | XLOC_020086 | -- | intronic_lncRNA | 0.575216 | 0.223137 | 0.209901 | 0.68054 |
| LNC_000933 | XLOC_020087 | -- | intronic_lncRNA | 0.552405 | 0.448165 | 0.798128 | 0.622468 |
| LNC_000930 | XLOC_020067 | -- | intronic_lncRNA | 0.722107 | 0 | 0 | 0.135639 |
| LNC_000931 | XLOC_020084 | -- | intronic_lncRNA | 0.344189 | 0.434995 | 0.535637 | 0.208554 |
| LNC_000936 | XLOC_020109 | -- | lincRNA | 0.007256 | 1.396445 | 0 | 0.454393 |
| LNC_000937 | XLOC_020153 | -- | intronic_lncRNA | 0 | 0.767353 | 0.16052 | 0.361441 |
| LNC_000934 | XLOC_020109 | -- | lincRNA | 0.401527 | 0.031894 | 0.657853 | 0 |
| LNC_000935 | XLOC_020109 | -- | lincRNA | 0.175283 | 0 | 0.645995 | 0.363461 |
| LNC_004549 | XLOC_099804 | -- | intronic_lncRNA | 0.107053 | 0 | 0.645603 | 0.345665 |
| LNC_004548 | XLOC_099778 | -- | intronic_lncRNA | 0 | 0 | 0.407265 | 0 |
| LNC_004547 | XLOC_099770 | -- | intronic_lncRNA | 8.813691 | 5.545488 | 4.430228 | 4.93615 |
| LNC_004546 | XLOC_099706 | -- | intronic_lncRNA | 0.913888 | 1.377141 | 0.58872 | 0.97187 |
| LNC_004545 | XLOC_099701 | -- | lincRNA | 0.131222 | 0 | 0.624146 | 0.102763 |
| LNC_004544 | XLOC_099675 | -- | intronic_lncRNA | 0.36497 | 0.25774 | 0.982875 | 0.516885 |
| LNC_004543 | XLOC_099489 | -- | lincRNA | 0 | 0.543744 | 0 | 0 |

| LNC_004542 | XLOC_099460 | -- | intronic_lncRNA | 0.018399 | 0.18805 | 0 | 0 |
| --- | --- | --- | --- | --- | --- | --- | --- |
| LNC_004541 | XLOC_099425 | -- | intronic_lncRNA | 0.390502 | 0.515579 | 0.803951 | 0.688198 |
| LNC_004540 | XLOC_099422 | -- | intronic_lncRNA | 0.565463 | 0 | 0 | 0.274104 |
| LNC_001687 | XLOC_036978 | -- | intronic_lncRNA | 0.023612 | 0.005859 | 0.370808 | 0 |
| LNC_001686 | XLOC_036910 | -- | intronic_lncRNA | 0 | 0.257556 | 0.633714 | 0 |
| LNC_001685 | XLOC_036849 | -- | lincRNA | 0.273462 | 0.150727 | 0.346151 | 0.442599 |
| LNC_001684 | XLOC_036789 | -- | intronic_lncRNA | 0.736506 | 0.886758 | 0.570565 | 0.327065 |
| LNC_001683 | XLOC_036767 | -- | lincRNA | 0 | 0.224749 | 0.519594 | 0.246955 |
| LNC_001682 | XLOC_036705 | -- | antisense_lncRNA | 0 | 0 | 0.412683 | 0.051663 |
| LNC_001681 | XLOC_036681 | -- | intronic_lncRNA | 0.523737 | 0.368619 | 1.708905 | 1.808747 |
| LNC_001680 | XLOC_036681 | -- | intronic_lncRNA | 0.654456 | 0.248793 | 0.743258 | 0.702796 |
| LNC_001689 | XLOC_036984 | -- | intronic_lncRNA | 2.802469 | 2.342465 | 2.364746 | 3.168066 |
| LNC_001688 | XLOC_036978 | -- | intronic_lncRNA | 0.447263 | 0.985843 | 0.222386 | 0.782249 |
| LNC_006248 | XLOC_150040 | -- | intronic_lncRNA | 0.185803 | 0.725204 | 0.323536 | 0.082572 |
| LNC_002268 | XLOC_052163 | -- | intronic_lncRNA | 0.795864 | 0.122347 | 0.00733 | 0.070805 |
| LNC_002269 | XLOC_052197 | -- | intronic_lncRNA | 0 | 0 | 0.443125 | 0.400959 |
| LNC_002264 | XLOC_052122 | -- | intronic_lncRNA | 0.306197 | 0.221971 | 0.485033 | 0.717139 |
| LNC_002265 | XLOC_052135 | -- | lincRNA | 0.339319 | 0 | 0.066784 | 0.556436 |
| LNC_002266 | XLOC_052149 | -- | intronic_lncRNA | 7.838272 | 3.799632 | 4.461174 | 4.953979 |
| LNC_002267 | XLOC_052151 | -- | intronic_lncRNA | 0.617586 | 0 | 0.884007 | 0.872827 |
| LNC_002260 | XLOC_052076 | -- | intronic_lncRNA | 0.932126 | 0.578812 | 0.382658 | 0.477602 |
| LNC_002261 | XLOC_052084 | -- | intronic_lncRNA | 0.19955 | 0.269407 | 0.463871 | 0.919865 |
| LNC_002262 | XLOC_052105 | -- | intronic_lncRNA | 0.709588 | 0.234822 | 0.428961 | 0.08628 |
| LNC_002263 | XLOC_052121 | -- | intronic_lncRNA | 0.791737 | 0.225506 | 0.547884 | 0.316005 |
| LNC_003018 | XLOC_069035 | -- | intronic_lncRNA | 0.092153 | 0.097342 | 0.484494 | 0 |
| LNC_003019 | XLOC_069063 | -- | intronic_lncRNA | 0.037604 | 0.101755 | 0.569118 | 0.743742 |
| LNC_003014 | XLOC_069009 | -- | intronic_lncRNA | 0.618132 | 0.141593 | 0.171572 | 0.272819 |
| LNC_003015 | XLOC_069009 | -- | intronic_lncRNA | 0.508514 | 0.078061 | 0.091698 | 0.110373 |
| LNC_003016 | XLOC_069028 | -- | lincRNA | 0.988025 | 0.24952 | 0.160586 | 0.76265 |
| LNC_003017 | XLOC_069034 | -- | intronic_lncRNA | 0.611613 | 0.520591 | 0.159766 | 0.164353 |
| LNC_003010 | XLOC_068900 | -- | intronic_lncRNA | 0.048334 | 0 | 0.80985 | 0 |
| LNC_003011 | XLOC_068939 | -- | lincRNA | 0 | 0 | 0.736501 | 0.081018 |
| LNC_003012 | XLOC_068976 | -- | lincRNA | 1.181079 | 0.307417 | 0 | 0.414869 |
| LNC_003013 | XLOC_068983 | -- | lincRNA | 1.355677 | 1.00217 | 0.785008 | 0.89392 |
| LNC_004842 | XLOC_108816 | -- | intronic_lncRNA | 0.978664 | 0.422805 | 0.600167 | 0.453359 |
| LNC_002659 | XLOC_061823 | -- | intronic_lncRNA | 0.498096 | 0.314347 | 0.08964 | 0.537746 |
| LNC_002658 | XLOC_061821 | -- | intronic_lncRNA | 0.380158 | 0.137929 | 0.33511 | 0.704541 |
| LNC_004843 | XLOC_108817 | -- | intronic_lncRNA | 0.515961 | 0.731604 | 0.891157 | 0.277546 |
| LNC_002655 | XLOC_061754 | -- | intronic_lncRNA | 0.89383 | 0.707053 | 0.89163 | 0.957638 |
| LNC_002654 | XLOC_061735 | -- | intronic_lncRNA | 0.269776 | 0.14285 | 0.456665 | 0.631829 |
| LNC_002657 | XLOC_061759 | -- | antisense_lncRNA | 0.485653 | 0.425748 | 0 | 0.189388 |
| LNC_002656 | XLOC_061756 | -- | intronic_lncRNA | 0 | 0.259374 | 0.165781 | 0.690635 |
| LNC_002651 | XLOC_061537 | -- | intronic_lncRNA | 0.066586 | 0.766238 | 0.437071 | 0.507288 |
| LNC_002650 | XLOC_061400 | -- | lincRNA | 0.490076 | 0.470425 | 0.073321 | 0.058487 |
| LNC_002653 | XLOC_061651 | -- | lincRNA | 0.656793 | 0 | 1.060126 | 0 |
| LNC_002652 | XLOC_061538 | -- | intronic_lncRNA | 0.496229 | 0.211721 | 0 | 0.057272 |
| LNC_004841 | XLOC_108800 | -- | intronic_lncRNA | 7.834839 | 7.431987 | 9.518504 | 10.208133 |
| LNC_004846 | XLOC_108867 | -- | intronic_lncRNA | 0.753295 | 0.528137 | 0.945743 | 0.660915 |
| LNC_004847 | XLOC_108868 | -- | intronic_lncRNA | 4.264443 | 1.103746 | 1.071295 | 1.736579 |
| LNC_004336 | XLOC_094228 | -- | lincRNA | 0 | 0 | 0.577088 | 0.173429 |
| LNC_004337 | XLOC_094264 | -- | intronic_lncRNA | 0.399298 | 0.636831 | 0.480645 | 0.700028 |
| LNC_004305 | XLOC_093647 | -- | intronic_lncRNA | 0.626621 | 0.726292 | 0.300872 | 0.775213 |
| LNC_004304 | XLOC_093627 | -- | intronic_lncRNA | 1.111415 | 0.724672 | 1.826584 | 1.025633 |
| LNC_004307 | XLOC_093676 | -- | intronic_lncRNA | 0.155171 | 0.39563 | 0.20841 | 0.197549 |
| LNC_004306 | XLOC_093669 | -- | intronic_lncRNA | 0 | 0 | 0.106581 | 0.497923 |
| LNC_004301 | XLOC_093569 | -- | intronic_lncRNA | 0.905528 | 0.610987 | 0.488782 | 0.619416 |
| LNC_004300 | XLOC_093539 | -- | intronic_lncRNA | 0 | 0 | 0.5017 | 0.25249 |
| LNC_004303 | XLOC_093625 | -- | intronic_lncRNA | 0.376191 | 0.708323 | 0.947592 | 0.716919 |
| LNC_004302 | XLOC_093576 | -- | intronic_lncRNA | 0.548459 | 0.240689 | 0.728063 | 0.522812 |
| LNC_004309 | XLOC_093735 | -- | intronic_lncRNA | 1.691771 | 1.936803 | 0.842524 | 1.511206 |
| LNC_004308 | XLOC_093677 | -- | intronic_lncRNA | 0.136427 | 0.562211 | 0.881516 | 0.07046 |
| LNC_001870 | XLOC_041631 | -- | antisense_lncRNA | 0.204969 | 0.207977 | 0.514612 | 0.161588 |
| LNC_001871 | XLOC_041710 | -- | intronic_lncRNA | 0.555065 | 0.520771 | 0.477448 | 0.45045 |
| LNC_001872 | XLOC_041750 | -- | intronic_lncRNA | 0.910882 | 0.523197 | 1.077664 | 0.659367 |
| LNC_001873 | XLOC_041776 | -- | intronic_lncRNA | 1.146776 | 0.812728 | 1.091701 | 1.182776 |
| LNC_001874 | XLOC_041799 | -- | intronic_lncRNA | 0.307283 | 0.781772 | 1.261879 | 1.398614 |
| LNC_001875 | XLOC_041799 | -- | intronic_lncRNA | 0.639207 | 0.158997 | 0.411597 | 0.31733 |
| LNC_001876 | XLOC_041808 | -- | intronic_lncRNA | 2.173511 | 0.3306 | 0.895579 | 0.272841 |
| LNC_001877 | XLOC_041810 | -- | intronic_lncRNA | 2.261267 | 1.81659 | 0.982953 | 1.372845 |
| LNC_001878 | XLOC_041851 | -- | intronic_lncRNA | 0.45532 | 1.428749 | 1.110818 | 1.024904 |
| LNC_001879 | XLOC_041853 | -- | intronic_lncRNA | 1.214752 | 0.814708 | 1.06282 | 0.636468 |
| LNC_001308 | XLOC_028594 | -- | intronic_lncRNA | 0.545064 | 0.272969 | 0.193696 | 0.121544 |
| LNC_001309 | XLOC_028597 | -- | intronic_lncRNA | 0 | 0.394019 | 1.092189 | 1.002461 |
| LNC_003658 | XLOC_078152 | -- | intronic_lncRNA | 0.713112 | 0.19361 | 0.40894 | 0.324047 |
| LNC_003659 | XLOC_078153 | -- | intronic_lncRNA | 0.277602 | 0.49152 | 0 | 0.386072 |
| LNC_003652 | XLOC_078123 | -- | intronic_lncRNA | 1.056712 | 0.490839 | 0.872622 | 0.5483 |
| LNC_003653 | XLOC_078137 | -- | intronic_lncRNA | 0.717081 | 0.220774 | 0.679036 | 1.334958 |

| LNC_003650 | XLOC_078100 | -- | intronic_lncRNA | 2.415269 | 0.432167 | 0.820319 | 0.835026 |
| --- | --- | --- | --- | --- | --- | --- | --- |
| LNC_003651 | XLOC_078110 | -- | intronic_lncRNA | 3.728601 | 1.222235 | 0.935468 | 0.812953 |
| LNC_003656 | XLOC_078150 | -- | intronic_lncRNA | 0.472576 | 0.154152 | 0.498521 | 0.386694 |
| LNC_003657 | XLOC_078151 | -- | intronic_lncRNA | 0 | 0 | 0.456143 | 0 |
| LNC_003654 | XLOC_078137 | -- | intronic_lncRNA | 0.397241 | 1.02643 | 0.283381 | 0.093381 |
| LNC_003655 | XLOC_078144 | -- | intronic_lncRNA | 0.163994 | 0.60416 | 0 | 0.3669 |
| LNC_005553 | XLOC_128747 | -- | lincRNA | 0.810259 | 0.089472 | 0 | 0.116089 |
| LNC_005552 | XLOC_128690 | -- | lincRNA | 0.446764 | 0.502469 | 0.492262 | 0.519036 |
| LNC_005551 | XLOC_128582 | -- | intronic_lncRNA | 0.625308 | 0.007384 | 0.519263 | 0.370235 |
| LNC_005550 | XLOC_128582 | -- | intronic_lncRNA | 0.417898 | 1.13896 | 0.671828 | 1.017675 |
| LNC_005557 | XLOC_128918 | -- | intronic_lncRNA | 0 | 0 | 0.481591 | 0 |
| LNC_005556 | XLOC_128888 | -- | lincRNA | 0.320777 | 0.243502 | 0.043485 | 0.437274 |
| LNC_005555 | XLOC_128875 | -- | lincRNA | 0.130256 | 0.493252 | 0.453396 | 0.479065 |
| LNC_005554 | XLOC_128828 | -- | intronic_lncRNA | 0.708257 | 0.698057 | 0.414961 | 0.714777 |
| LNC_005559 | XLOC_128944 | -- | intronic_lncRNA | 0 | 0.610563 | 0 | 0 |
| LNC_005558 | XLOC_128940 | -- | intronic_lncRNA | 1.345379 | 0 | 0 | 0 |
| LNC_002019 | XLOC_045406 | -- | intronic_lncRNA | 0.58721 | 0.468459 | 0.408025 | 0 |
| LNC_002018 | XLOC_045406 | -- | intronic_lncRNA | 0.627941 | 0.101692 | 0.65417 | 1.404797 |
| LNC_002013 | XLOC_045355 | -- | intronic_lncRNA | 0.081016 | 0.001309 | 0 | 0.303265 |
| LNC_002012 | XLOC_045355 | -- | intronic_lncRNA | 0.286923 | 0.360385 | 0.034335 | 0.0779 |
| LNC_002011 | XLOC_045329 | -- | intronic_lncRNA | 0.876216 | 0.760643 | 0.852428 | 1.167262 |
| LNC_002010 | XLOC_045328 | -- | intronic_lncRNA | 0.401229 | 0.364016 | 0.700354 | 0.692576 |
| LNC_002017 | XLOC_045390 | -- | intronic_lncRNA | 0.272217 | 0 | 0.458256 | 0 |
| LNC_002016 | XLOC_045384 | -- | intronic_lncRNA | 0.369092 | 0.510709 | 0.45948 | 0.894907 |
| LNC_002015 | XLOC_045356 | -- | intronic_lncRNA | 0.697467 | 0.693725 | 1.003758 | 0.944466 |
| LNC_002014 | XLOC_045355 | -- | intronic_lncRNA | 0.311284 | 0.256048 | 0.963527 | 0.373833 |
| LNC_003229 | XLOC_074920 | -- | intronic_lncRNA | 1.165272 | 0.041004 | 0.223392 | 0.360678 |
| LNC_003228 | XLOC_074861 | -- | lincRNA | 0 | 0 | 0.182143 | 0.62489 |
| LNC_003223 | XLOC_074786 | -- | intronic_lncRNA | 1.305294 | 0.299935 | 0.021167 | 0.528719 |
| LNC_003222 | XLOC_074777 | -- | intronic_lncRNA | 1.761492 | 1.833107 | 1.30619 | 1.114941 |
| LNC_003221 | XLOC_074750 | -- | intronic_lncRNA | 0.630457 | 0.877173 | 0.359376 | 0.275641 |
| LNC_003220 | XLOC_074739 | -- | intronic_lncRNA | 0.574621 | 0.63746 | 0.58221 | 0.608842 |
| LNC_003227 | XLOC_074838 | -- | intronic_lncRNA | 0.504269 | 0 | 1.091223 | 0.315633 |
| LNC_003226 | XLOC_074791 | -- | intronic_lncRNA | 0.378149 | 0.324019 | 0.304103 | 0.05189 |
| LNC_003225 | XLOC_074790 | -- | intronic_lncRNA | 1.552467 | 0.693809 | 0.284541 | 0.099734 |
| LNC_003224 | XLOC_074789 | -- | intronic_lncRNA | 0.599274 | 0.482331 | 0.258862 | 0.644332 |
| LNC_000228 | XLOC_003185 | -- | intronic_lncRNA | 0.244935 | 0.281039 | 0.285166 | 0.649122 |
| LNC_000229 | XLOC_003187 | -- | intronic_lncRNA | 0.536205 | 0.127331 | 0.254879 | 0.900256 |
| LNC_000226 | XLOC_003175 | -- | intronic_lncRNA | 0.580454 | 0 | 0.102111 | 0 |
| LNC_000227 | XLOC_003184 | -- | intronic_lncRNA | 0.150958 | 0 | 0.684048 | 0 |
| LNC_000224 | XLOC_003165 | -- | intronic_lncRNA | 0 | 0.483328 | 0 | 0.359401 |
| LNC_000225 | XLOC_003165 | -- | intronic_lncRNA | 0.772487 | 0.026334 | 0 | 0 |
| LNC_005302 | XLOC_121358 | -- | intronic_lncRNA | 0.180514 | 0.321622 | 0.462905 | 0.349737 |
| LNC_005303 | XLOC_121361 | -- | intronic_lncRNA | 12.364991 | 16.965611 | 12.743036 | 16.966463 |
| LNC_005300 | XLOC_121321 | -- | intronic_lncRNA | 0.601854 | 1.765443 | 0.943798 | 1.76341 |
| LNC_000221 | XLOC_003149 | -- | intronic_lncRNA | 0 | 0 | 0 | 0.394993 |
| LNC_001311 | XLOC_028627 | -- | intronic_lncRNA | 0 | 0.955448 | 0 | 0 |
| LNC_001310 | XLOC_028597 | -- | intronic_lncRNA | 1.818003 | 1.23877 | 1.52661 | 1.001685 |
| LNC_001313 | XLOC_028741 | -- | intronic_lncRNA | 0 | 0 | 0 | 0.897957 |
| LNC_001312 | XLOC_028634 | -- | intronic_lncRNA | 2.625161 | 1.085643 | 5.29156 | 4.879851 |
| LNC_001315 | XLOC_028755 | -- | intronic_lncRNA | 0.267058 | 0.765034 | 0.602786 | 0.620902 |
| LNC_001314 | XLOC_028743 | -- | lincRNA | 0 | 0 | 0.456601 | 0 |
| LNC_001317 | XLOC_028792 | -- | intronic_lncRNA | 0.387926 | 0.488396 | 0.336598 | 0.2612 |
| LNC_001316 | XLOC_028773 | -- | intronic_lncRNA | 0.36373 | 0.280672 | 0.514587 | 0.634812 |
| LNC_005979 | XLOC_142243 | -- | intronic_lncRNA | 0.117766 | 0 | 0.529751 | 0 |
| LNC_005978 | XLOC_141991 | -- | lincRNA | 0.217646 | 0.951238 | 0.077205 | 0.077224 |
| LNC_005975 | XLOC_141854 | -- | intronic_lncRNA | 0.89752 | 1.132578 | 0.670483 | 0 |
| LNC_005974 | XLOC_141796 | -- | intronic_lncRNA | 1.401012 | 1.425759 | 0.897185 | 0.431606 |
| LNC_005977 | XLOC_141882 | -- | intronic_lncRNA | 0 | 0 | 0 | 0.559085 |
| LNC_005976 | XLOC_141881 | -- | intronic_lncRNA | 0 | 0 | 0 | 0.602092 |
| LNC_005971 | XLOC_141412 | -- | intronic_lncRNA | 0 | 0 | 0.438984 | 0.432324 |
| LNC_005970 | XLOC_141401 | -- | lincRNA | 0.315067 | 0.867688 | 0.046916 | 0.356968 |
| LNC_005973 | XLOC_141762 | -- | intronic_lncRNA | 0.219162 | 0.369389 | 0.248086 | 0.156367 |
| LNC_005972 | XLOC_141762 | -- | intronic_lncRNA | 0.388097 | 0.24725 | 0.741634 | 0.626297 |
| LNC_001092 | XLOC_023539 | -- | intronic_lncRNA | 0 | 0 | 0.544069 | 0 |
| LNC_001093 | XLOC_023540 | -- | intronic_lncRNA | 0.211717 | 0.20564 | 0.524249 | 0 |
| LNC_001090 | XLOC_023537 | -- | intronic_lncRNA | 0.026942 | 0.554239 | 0.224474 | 0.303362 |
| LNC_001091 | XLOC_023538 | -- | intronic_lncRNA | 0 | 0.892297 | 0.483362 | 0 |
| LNC_001096 | XLOC_023552 | -- | intronic_lncRNA | 0.505475 | 0 | 0 | 0 |
| LNC_001097 | XLOC_023582 | -- | intronic_lncRNA | 0.795414 | 0.718961 | 0.89437 | 1.273707 |
| LNC_001094 | XLOC_023542 | -- | intronic_lncRNA | 0.362977 | 0.327317 | 1.419481 | 1.016402 |
| LNC_001095 | XLOC_023542 | -- | intronic_lncRNA | 0.508632 | 0 | 0 | 0 |
| LNC_001098 | XLOC_023583 | -- | intronic_lncRNA | 0.219633 | 0.292188 | 0.483581 | 0.466083 |
| LNC_001099 | XLOC_023584 | -- | lincRNA | 0.408732 | 0.322291 | 0.445012 | 0.672382 |
| LNC_003821 | XLOC_082128 | -- | intronic_lncRNA | 0 | 0.404171 | 0.599105 | 1.560122 |
| LNC_003820 | XLOC_082126 | -- | lincRNA | 0 | 0.105798 | 0.465543 | 0.676967 |
| LNC_003823 | XLOC_082131 | -- | intronic_lncRNA | 0.428428 | 0.147742 | 0.298058 | 0.341799 |

| LNC_003822 | XLOC_082129 | -- | intronic_lncRNA | 0.321435 | 0.487496 | 0.739001 | 0.584931 |
| --- | --- | --- | --- | --- | --- | --- | --- |
| LNC_003825 | XLOC_082133 | -- | intronic_lncRNA | 0.498047 | 0.390425 | 0.561827 | 0.491895 |
| LNC_003824 | XLOC_082132 | -- | intronic_lncRNA | 0.113566 | 0 | 0.043654 | 0.523753 |
| LNC_003827 | XLOC_082258 | -- | intronic_lncRNA | 0.36513 | 0.111752 | 0.449199 | 0.450931 |
| LNC_003826 | XLOC_082255 | -- | intronic_lncRNA | 1.07782 | 0.803715 | 0.48276 | 0.526853 |
| LNC_003829 | XLOC_082269 | -- | intronic_lncRNA | 2.475285 | 0.922748 | 1.692092 | 1.622171 |
| LNC_003828 | XLOC_082269 | -- | intronic_lncRNA | 2.098618 | 1.719544 | 0.76542 | 1.992643 |
| LNC_000323 | XLOC_004657 | -- | intronic_lncRNA | 0.056986 | 0 | 0 | 0.612215 |
| LNC_000322 | XLOC_004641 | -- | intronic_lncRNA | 0.61151 | 2.609734 | 0.446684 | 0.009865 |
| LNC_000321 | XLOC_004641 | -- | intronic_lncRNA | 0.458793 | 0.472649 | 0.205103 | 1.021626 |
| LNC_000320 | XLOC_004599 | -- | intronic_lncRNA | 0.386018 | 0.209469 | 0.307661 | 0.85127 |
| LNC_000327 | XLOC_004720 | -- | intronic_lncRNA | 0.880191 | 0.850219 | 0.692057 | 0.946885 |
| LNC_000326 | XLOC_004698 | -- | antisense_lncRNA | 0.90591 | 0.853841 | 0 | 0 |
| LNC_000325 | XLOC_004680 | -- | intronic_lncRNA | 0 | 0.568621 | 0 | 0 |
| LNC_000324 | XLOC_004657 | -- | intronic_lncRNA | 1.784633 | 0 | 0 | 0 |
| LNC_000329 | XLOC_004995 | -- | intronic_lncRNA | 0.552295 | 0.606414 | 0.130759 | 0.049578 |
| LNC_000328 | XLOC_004861 | -- | antisense_lncRNA | 0 | 0.452785 | 0.306002 | 0.685326 |
| LNC_003799 | XLOC_081520 | -- | intronic_lncRNA | 0.168248 | 0.503612 | 0.847205 | 0.426814 |
| LNC_003798 | XLOC_081514 | -- | intronic_lncRNA | 0.761925 | 0.120985 | 0 | 0.207487 |
| LNC_003793 | XLOC_081344 | -- | intronic_lncRNA | 1.949801 | 1.392359 | 1.174433 | 1.212588 |
| LNC_003792 | XLOC_081203 | -- | lincRNA | 0.514421 | 0 | 0 | 0.175939 |
| LNC_003791 | XLOC_081145 | -- | intronic_lncRNA | 0.809267 | 0.221831 | 0.827223 | 0.872242 |
| LNC_003790 | XLOC_081125 | -- | intronic_lncRNA | 0 | 0.47542 | 0.025826 | 0 |
| LNC_003797 | XLOC_081513 | -- | intronic_lncRNA | 0.185745 | 0.151044 | 0 | 0.466262 |
| LNC_003796 | XLOC_081425 | -- | antisense_lncRNA | 0.972359 | 1.242474 | 1.699206 | 1.108551 |
| LNC_003795 | XLOC_081388 | -- | antisense_lncRNA | 0.847044 | 0.181723 | 0.270162 | 0.351768 |
| LNC_003794 | XLOC_081377 | -- | intronic_lncRNA | 1.168468 | 0.560717 | 1.352627 | 1.594648 |
| LNC_005308 | XLOC_121458 | -- | antisense_lncRNA | 1.841358 | 1.5616 | 0.388216 | 0.345618 |
| LNC_005309 | XLOC_121471 | -- | intronic_lncRNA | 0 | 0 | 0.019946 | 0.514874 |
| LNC_005306 | XLOC_121420 | -- | intronic_lncRNA | 0 | 0 | 0.417932 | 0.563911 |
| LNC_005307 | XLOC_121420 | -- | intronic_lncRNA | 2.004244 | 1.483857 | 2.186585 | 1.914312 |
| LNC_005304 | XLOC_121361 | -- | intronic_lncRNA | 2.094032 | 2.512881 | 5.428298 | 5.013955 |
| LNC_005305 | XLOC_121387 | -- | intronic_lncRNA | 0 | 0.668195 | 0 | 0 |
| LNC_000222 | XLOC_003164 | -- | intronic_lncRNA | 0.03934 | 0.065439 | 0.080585 | 0.337911 |
| LNC_000223 | XLOC_003164 | -- | intronic_lncRNA | 0.564399 | 0.773858 | 0.321605 | 0.193501 |
| LNC_000220 | XLOC_003101 | -- | intronic_lncRNA | 0.246752 | 1.245798 | 1.073824 | 0.176325 |
| LNC_005301 | XLOC_121357 | -- | intronic_lncRNA | 0.598713 | 0.421418 | 0.540082 | 0.254563 |
| LNC_002848 | XLOC_065595 | -- | intronic_lncRNA | 0.357201 | 0.865664 | 1.50826 | 0.398646 |
| LNC_002849 | XLOC_065606 | -- | intronic_lncRNA | 0.082308 | 0 | 0.421898 | 0.131234 |
| LNC_002840 | XLOC_065462 | -- | lincRNA | 0 | 0 | 0.385016 | 0.555248 |
| LNC_002841 | XLOC_065462 | -- | lincRNA | 0.651882 | 0.419805 | 0.109607 | 0.273423 |
| LNC_002842 | XLOC_065480 | -- | lincRNA | 0.486228 | 0.144074 | 0 | 0.321601 |
| LNC_002843 | XLOC_065539 | -- | intronic_lncRNA | 0 | 0.483328 | 0 | 0.78011 |
| LNC_002844 | XLOC_065552 | -- | intronic_lncRNA | 0.333802 | 0.204889 | 0.464117 | 0.426275 |
| LNC_002845 | XLOC_065552 | -- | intronic_lncRNA | 0.488463 | 0.662352 | 0.106399 | 0.260825 |
| LNC_002846 | XLOC_065570 | -- | intronic_lncRNA | 0 | 0.432831 | 0.241238 | 0 |
| LNC_002847 | XLOC_065590 | -- | intronic_lncRNA | 0.455254 | 0 | 0.391819 | 0.234685 |
| LNC_006017 | XLOC_143333 | -- | intronic_lncRNA | 0 | 0 | 0 | 0.869688 |
| LNC_006016 | XLOC_143260 | -- | intronic_lncRNA | 0 | 0 | 0.65247 | 0.326179 |
| LNC_006015 | XLOC_143240 | -- | intronic_lncRNA | 0.522425 | 0.104066 | 0.124419 | 0.21684 |
| LNC_006014 | XLOC_143239 | -- | intronic_lncRNA | 0.459235 | 0.313601 | 0.603117 | 0.70761 |
| LNC_000918 | XLOC_019918 | -- | intronic_lncRNA | 1.063863 | 0.299973 | 0.747813 | 0.947613 |
| LNC_000919 | XLOC_019918 | -- | intronic_lncRNA | 0.844094 | 0.380836 | 1.355616 | 1.048483 |
| LNC_006011 | XLOC_142904 | -- | lincRNA | 0.830074 | 0.317833 | 0 | 0 |
| LNC_006010 | XLOC_142848 | -- | intronic_lncRNA | 0.191919 | 0.201957 | 0.176479 | 0.620013 |
| LNC_000914 | XLOC_019854 | -- | intronic_lncRNA | 0.383495 | 0.13239 | 0.363349 | 0.645098 |
| LNC_000915 | XLOC_019856 | -- | lincRNA | 1.273521 | 0.979544 | 0.049705 | 0.074296 |
| LNC_000916 | XLOC_019858 | -- | lincRNA | 0 | 0.333475 | 0 | 0.410159 |
| LNC_000917 | XLOC_019918 | -- | intronic_lncRNA | 1.077872 | 1.31459 | 1.523883 | 0.809095 |
| LNC_000910 | XLOC_019772 | -- | intronic_lncRNA | 0.382635 | 0.330706 | 0.539127 | 0.500557 |
| LNC_000911 | XLOC_019784 | -- | intronic_lncRNA | 0 | 0.745944 | 0 | 0 |
| LNC_000912 | XLOC_019789 | -- | intronic_lncRNA | 0 | 0.074341 | 0.199031 | 0.486575 |
| LNC_000913 | XLOC_019789 | -- | intronic_lncRNA | 0 | 0.06281 | 0 | 0.637048 |
| LNC_006243 | XLOC_149835 | -- | intronic_lncRNA | 1.09302 | 0.310008 | 0.199192 | 0.942213 |
| LNC_006457 | XLOC_155302 | -- | intronic_lncRNA | 0.086767 | 0.996686 | 0 | 0.430893 |
| LNC_006456 | XLOC_155287 | -- | lincRNA | 0 | 0.946569 | 0.094459 | 0.05962 |
| LNC_000558 | XLOC_010462 | -- | intronic_lncRNA | 0.666876 | 0.241664 | 0.195714 | 0.138933 |
| LNC_000559 | XLOC_010467 | -- | intronic_lncRNA | 0.630053 | 0 | 0.152665 | 0.203745 |
| LNC_005074 | XLOC_115819 | -- | intronic_lncRNA | 0.261197 | 0.201251 | 0.41824 | 0.743099 |
| LNC_005075 | XLOC_115833 | -- | lincRNA | 0 | 0 | 0 | 0.392997 |
| LNC_005076 | XLOC_115842 | -- | intronic_lncRNA | 0.329061 | 0.626237 | 0.579288 | 0.148932 |
| LNC_005077 | XLOC_115855 | -- | lincRNA | 0.749429 | 0.26284 | 0.146159 | 0.157948 |
| LNC_005070 | XLOC_115728 | -- | lincRNA | 0.176073 | 0 | 0.349948 | 0.607484 |
| LNC_000551 | XLOC_010240 | -- | intronic_lncRNA | 0.52959 | 0.410862 | 0.247188 | 0.158955 |
| LNC_005896 | XLOC_138896 | -- | intronic_lncRNA | 0.294672 | 0.092651 | 0.150068 | 0.525147 |
| LNC_005897 | XLOC_138898 | -- | intronic_lncRNA | 0.657918 | 0 | 0.267897 | 0.323207 |
| LNC_005894 | XLOC_138837 | -- | intronic_lncRNA | 0 | 0.485069 | 0 | 0.634637 |

| LNC_005895 | XLOC_138838 | -- | intronic_lncRNA | 0 | 0 | 0.258878 | 0.413304 |
| --- | --- | --- | --- | --- | --- | --- | --- |
| LNC_005892 | XLOC_138781 | -- | intronic_lncRNA | 0.422668 | 0.143275 | 0.410577 | 0.819351 |
| LNC_005072 | XLOC_115731 | -- | intronic_lncRNA | 0.791504 | 0 | 1.840064 | 1.032531 |
| LNC_005890 | XLOC_138780 | -- | intronic_lncRNA | 0 | 0.037968 | 0 | 0.638625 |
| LNC_005891 | XLOC_138780 | -- | intronic_lncRNA | 0.397659 | 0.273347 | 0.550524 | 0.016227 |
| LNC_005073 | XLOC_115792 | -- | lincRNA | 0 | 0 | 0.416826 | 0.268253 |
| LNC_005898 | XLOC_138901 | -- | intronic_lncRNA | 0.328273 | 0.09385 | 0.189993 | 0.437462 |
| LNC_005899 | XLOC_138902 | -- | intronic_lncRNA | 0.506644 | 0.321611 | 0.180968 | 0.238703 |
| LNC_002206 | XLOC_050297 | -- | intronic_lncRNA | 0.700683 | 0.587224 | 0.69183 | 0.617547 |
| LNC_002207 | XLOC_050433 | -- | lincRNA | 5.43501 | 3.603048 | 4.253985 | 5.108716 |
| LNC_002204 | XLOC_050285 | -- | lincRNA | 0.805577 | 0.749057 | 0.489469 | 0.77048 |
| LNC_002205 | XLOC_050285 | -- | lincRNA | 0.956806 | 0 | 0.419487 | 1.060436 |
| LNC_002202 | XLOC_050223 | -- | lincRNA | 0.531681 | 0.429271 | 0.833253 | 0.329851 |
| LNC_002203 | XLOC_050260 | -- | lincRNA | 0.101625 | 0.202795 | 0.410588 | 0.480392 |
| LNC_002200 | XLOC_050216 | -- | lincRNA | 0.238001 | 0.46913 | 0.6621 | 0.344535 |
| LNC_002201 | XLOC_050216 | -- | lincRNA | 0.40099 | 0.065535 | 0.117489 | 0.752096 |
| LNC_002208 | XLOC_050434 | -- | lincRNA | 0.070687 | 0.587414 | 0 | 0 |
| LNC_002209 | XLOC_050439 | -- | intronic_lncRNA | 1.195937 | 1.55204 | 1.596552 | 1.750591 |
| LNC_003076 | XLOC_070589 | -- | intronic_lncRNA | 0.142663 | 0.293667 | 0.416201 | 0.344198 |
| LNC_003077 | XLOC_070589 | -- | intronic_lncRNA | 0.22919 | 0.087311 | 0.033082 | 0.091654 |
| LNC_003074 | XLOC_070587 | -- | intronic_lncRNA | 0.466061 | 0.088653 | 0.253686 | 0.534456 |
| LNC_003075 | XLOC_070588 | -- | intronic_lncRNA | 0.648521 | 0.394553 | 0.222253 | 0.106492 |
| LNC_003072 | XLOC_070571 | -- | intronic_lncRNA | 0 | 0.745914 | 0.177585 | 0.177554 |
| LNC_003073 | XLOC_070586 | -- | intronic_lncRNA | 0.140979 | 0.096344 | 0.506905 | 0.098815 |
| LNC_003070 | XLOC_070560 | -- | intronic_lncRNA | 0.344231 | 1.415601 | 0.174356 | 0 |
| LNC_003071 | XLOC_070562 | -- | intronic_lncRNA | 0.875762 | 0.857133 | 0.15696 | 0 |
| LNC_005831 | XLOC_137464 | -- | intronic_lncRNA | 1.196863 | 0.474086 | 1.973003 | 2.084758 |
| LNC_003078 | XLOC_070604 | -- | intronic_lncRNA | 0.038526 | 0 | 0.002745 | 0.993014 |
| LNC_003079 | XLOC_070641 | -- | intronic_lncRNA | 0 | 0 | 0.400323 | 0.054257 |
| LNC_006452 | XLOC_155232 | -- | intronic_lncRNA | 0.538552 | 0.526796 | 0.075357 | 0.58608 |
| LNC_005098 | XLOC_116727 | -- | intronic_lncRNA | 0 | 0.178197 | 0.550173 | 0.472004 |
| LNC_005099 | XLOC_116727 | -- | intronic_lncRNA | 0 | 0.026389 | 0.090493 | 0.430704 |
| LNC_005096 | XLOC_116534 | -- | lincRNA | 0.524306 | 0 | 0.080173 | 0.106252 |
| LNC_005097 | XLOC_116677 | -- | intronic_lncRNA | 0.659096 | 0 | 0 | 0 |
| LNC_005094 | XLOC_116461 | -- | lincRNA | 0 | 0.255504 | 0.310383 | 0 |
| LNC_005095 | XLOC_116502 | -- | lincRNA | 0.185449 | 0 | 0.702889 | 0 |
| LNC_005092 | XLOC_116416 | -- | intronic_lncRNA | 5.791486 | 14.297927 | 10.467499 | 13.494008 |
| LNC_005093 | XLOC_116454 | -- | lincRNA | 0.641364 | 0.501302 | 1.06851 | 0.617479 |
| LNC_005090 | XLOC_116372 | -- | intronic_lncRNA | 1.189447 | 0.750357 | 0.570828 | 0.926249 |
| LNC_005091 | XLOC_116381 | -- | lincRNA | 0 | 0 | 0.575853 | 0.108294 |
| LNC_006451 | XLOC_155232 | -- | intronic_lncRNA | 0.257356 | 0.19678 | 0.968647 | 0.333089 |
| LNC_006450 | XLOC_155096 | -- | intronic_lncRNA | 0.262634 | 0.084569 | 0.564976 | 0.583975 |
| LNC_002677 | XLOC_062342 | -- | intronic_lncRNA | 0.595475 | 0.607707 | 0.805072 | 1.453022 |
| LNC_002676 | XLOC_062262 | -- | intronic_lncRNA | 0.541693 | 0.324393 | 0.588479 | 0.829532 |
| LNC_002675 | XLOC_062260 | -- | intronic_lncRNA | 0 | 0 | 1.158028 | 0.106255 |
| LNC_002674 | XLOC_062237 | -- | intronic_lncRNA | 0.278187 | 0.727264 | 0.485419 | 0.176173 |
| LNC_002673 | XLOC_062167 | -- | intronic_lncRNA | 0.531123 | 0.224152 | 0.566681 | 0.294938 |
| LNC_002672 | XLOC_062159 | -- | intronic_lncRNA | 0.343349 | 0.596704 | 0 | 0.349716 |
| LNC_002671 | XLOC_062011 | -- | intronic_lncRNA | 0.108045 | 0.206664 | 0.509455 | 0.436794 |
| LNC_002670 | XLOC_062010 | -- | intronic_lncRNA | 0.1543 | 0.87077 | 0.825171 | 0.662985 |
| LNC_002679 | XLOC_062426 | -- | intronic_lncRNA | 0.315896 | 0 | 0.142337 | 0.479594 |
| LNC_002678 | XLOC_062361 | -- | intronic_lncRNA | 0.170113 | 0.213299 | 0.441045 | 0 |
| LNC_004859 | XLOC_109497 | -- | intronic_lncRNA | 0.192847 | 0.26244 | 0.477152 | 0.524528 |
| LNC_004858 | XLOC_109496 | -- | intronic_lncRNA | 0 | 0 | 0 | 0.404773 |
| LNC_004329 | XLOC_093956 | -- | intronic_lncRNA | 0.611962 | 0.284995 | 0.591856 | 0.447707 |
| LNC_004328 | XLOC_093955 | -- | intronic_lncRNA | 0.187379 | 0.255934 | 0.337 | 0.547904 |
| LNC_004327 | XLOC_093938 | -- | lincRNA | 0 | 0.646382 | 0 | 0 |
| LNC_004326 | XLOC_093931 | -- | intronic_lncRNA | 0.602644 | 0.112861 | 0.145851 | 0.235574 |
| LNC_004853 | XLOC_109093 | -- | intronic_lncRNA | 0.576141 | 0.345737 | 0.91579 | 0.632695 |
| LNC_004324 | XLOC_093910 | -- | intronic_lncRNA | 0.200715 | 0.694945 | 0.462225 | 0.657853 |
| LNC_004855 | XLOC_109172 | -- | intronic_lncRNA | 0.229049 | 0.089519 | 0.94469 | 0.520142 |
| LNC_004322 | XLOC_093832 | -- | antisense_lncRNA | 0.98565 | 0.457782 | 0 | 0.096927 |
| LNC_004857 | XLOC_109492 | -- | intronic_lncRNA | 0.754705 | 0 | 0 | 0 |
| LNC_004856 | XLOC_109237 | -- | intronic_lncRNA | 0.030375 | 0.022882 | 0.530908 | 0.504753 |
| LNC_005806 | XLOC_136696 | -- | lincRNA | 0.361578 | 0.329168 | 0.106632 | 1.135077 |
| LNC_005801 | XLOC_136665 | -- | intronic_lncRNA | 0.1444 | 0.487342 | 0.498883 | 0.618094 |
| LNC_005800 | XLOC_136632 | -- | intronic_lncRNA | 0.46251 | 0.502661 | 0.417976 | 0 |
| LNC_005803 | XLOC_136678 | -- | intronic_lncRNA | 0 | 0.185649 | 0.406678 | 0.201948 |
| LNC_005802 | XLOC_136666 | -- | intronic_lncRNA | 0.641412 | 1.110181 | 0.840972 | 0.419329 |
| LNC_003921 | XLOC_084555 | -- | intronic_lncRNA | 0.95855 | 0.95277 | 0.311412 | 0.410993 |
| LNC_001324 | XLOC_028880 | -- | intronic_lncRNA | 1.139081 | 0.688986 | 0.938033 | 1.113738 |
| LNC_001325 | XLOC_028937 | -- | intronic_lncRNA | 0.543584 | 0.178193 | 0.64152 | 0.806446 |
| LNC_001326 | XLOC_028940 | -- | intronic_lncRNA | 0 | 0.554134 | 0.14959 | 0.218364 |
| LNC_001327 | XLOC_029047 | -- | intronic_lncRNA | 0 | 0 | 0.449916 | 0.341527 |
| LNC_001320 | XLOC_028827 | -- | intronic_lncRNA | 0.623374 | 0.20369 | 0.464676 | 0.12531 |
| LNC_001321 | XLOC_028831 | -- | intronic_lncRNA | 0.8786 | 1.129732 | 1.113327 | 0.915065 |
| LNC_001322 | XLOC_028833 | -- | intronic_lncRNA | 0 | 0 | 0.687384 | 0 |

| LNC_001323 | XLOC_028835 | -- | intronic_lncRNA | 0.289234 | 0.278472 | 0.513903 | 0.481486 |
| --- | --- | --- | --- | --- | --- | --- | --- |
| LNC_001328 | XLOC_029074 | -- | intronic_lncRNA | 3.414485 | 0.072551 | 2.1312 | 0.125584 |
| LNC_001329 | XLOC_029088 | -- | intronic_lncRNA | 0.8094 | 1.004832 | 0.267558 | 0.680471 |
| LNC_006430 | XLOC_154050 | -- | intronic_lncRNA | 0.465942 | 0.250127 | 0.539469 | 0.502524 |
| LNC_006433 | XLOC_154101 | -- | intronic_lncRNA | 0.291977 | 0.131821 | 0.437887 | 0.474413 |
| LNC_003678 | XLOC_078630 | -- | intronic_lncRNA | 0.576933 | 0.153641 | 0 | 0 |
| LNC_003679 | XLOC_078637 | -- | intronic_lncRNA | 0.783884 | 0 | 0.202814 | 0.269826 |
| LNC_003674 | XLOC_078569 | -- | intronic_lncRNA | 0.252732 | 0 | 0.203712 | 0.574068 |
| LNC_003675 | XLOC_078571 | -- | intronic_lncRNA | 0.704459 | 0.101753 | 0.725171 | 0.20696 |
| LNC_003676 | XLOC_078614 | -- | intronic_lncRNA | 1.19418 | 0.364864 | 1.202872 | 0.945316 |
| LNC_003677 | XLOC_078625 | -- | intronic_lncRNA | 0 | 0 | 0.391427 | 0 |
| LNC_003670 | XLOC_078522 | -- | lincRNA | 0.641041 | 0.959003 | 0 | 0 |
| LNC_003671 | XLOC_078546 | -- | intronic_lncRNA | 1.936188 | 1.265551 | 1.76315 | 1.328945 |
| LNC_003672 | XLOC_078564 | -- | intronic_lncRNA | 1.050247 | 1.44679 | 2.456112 | 2.516593 |
| LNC_003673 | XLOC_078569 | -- | intronic_lncRNA | 0.74918 | 0.835592 | 0.90942 | 0 |
| LNC_006432 | XLOC_154069 | -- | intronic_lncRNA | 0 | 0 | 0 | 0.30712 |
| LNC_006435 | XLOC_154176 | -- | intronic_lncRNA | 0.631359 | 0.709434 | 0.841989 | 0.839425 |
| LNC_006013 | XLOC_143236 | -- | intronic_lncRNA | 0.358702 | 0.193331 | 0.576403 | 0.818046 |
| LNC_006012 | XLOC_143131 | -- | lincRNA | 0.260838 | 0.29489 | 0.419443 | 0.167539 |
| LNC_005535 | XLOC_127950 | -- | intronic_lncRNA | 0.968546 | 0.489707 | 0.802723 | 0.731749 |
| LNC_005534 | XLOC_127823 | -- | intronic_lncRNA | 1.241026 | 0.436244 | 0.359289 | 0.44851 |
| LNC_005537 | XLOC_127974 | -- | intronic_lncRNA | 0.238809 | 0.116231 | 0.094445 | 0.470573 |
| LNC_005536 | XLOC_127974 | -- | intronic_lncRNA | 0.277124 | 0.20622 | 0.621306 | 0.703943 |
| LNC_005531 | XLOC_127796 | -- | intronic_lncRNA | 0.365636 | 0.024639 | 0.498579 | 0.508202 |
| LNC_005530 | XLOC_127753 | -- | intronic_lncRNA | 0.349954 | 0.573696 | 0.55923 | 0.47693 |
| LNC_005533 | XLOC_127804 | -- | lincRNA | 0.522748 | 0 | 0.124889 | 0 |
| LNC_005532 | XLOC_127797 | -- | intronic_lncRNA | 0.235176 | 0.174324 | 0.607632 | 0.522075 |
| LNC_005539 | XLOC_128044 | -- | intronic_lncRNA | 0.213264 | 0.425844 | 1.071378 | 0.70494 |
| LNC_005538 | XLOC_128017 | -- | lincRNA | 1.197069 | 0.578925 | 0.248757 | 0.304858 |
| LNC_002079 | XLOC_046928 | -- | lincRNA | 0.594084 | 0.76762 | 1.069192 | 0.33375 |
| LNC_002078 | XLOC_046920 | -- | lincRNA | 0.929302 | 0.444387 | 0.826372 | 0.899377 |
| LNC_002075 | XLOC_046710 | -- | lincRNA | 1.160852 | 0.721819 | 0.709648 | 0.974899 |
| LNC_002074 | XLOC_046705 | -- | intronic_lncRNA | 0.071354 | 0.142862 | 0.218011 | 1.22588 |
| LNC_002077 | XLOC_046870 | -- | intronic_lncRNA | 0.688335 | 0.162972 | 0.543668 | 0.944882 |
| LNC_002076 | XLOC_046730 | -- | intronic_lncRNA | 0.343285 | 0.572001 | 0.53567 | 0.091215 |
| LNC_002071 | XLOC_046529 | -- | antisense_lncRNA | 0.575541 | 0 | 0 | 0 |
| LNC_002070 | XLOC_046519 | -- | lincRNA | 1.206168 | 0.117854 | 0.051234 | 0.212324 |
| LNC_002073 | XLOC_046705 | -- | intronic_lncRNA | 0.500333 | 0.183649 | 1.23027 | 0.220699 |
| LNC_002072 | XLOC_046585 | -- | intronic_lncRNA | 0.578366 | 0.359633 | 0.960877 | 0.346235 |
| LNC_003249 | XLOC_075119 | -- | lincRNA | 2.568583 | 1.658849 | 1.513448 | 0.990602 |
| LNC_003248 | XLOC_075118 | -- | lincRNA | 1.038818 | 0.905383 | 0.240324 | 0.524298 |
| LNC_003245 | XLOC_075117 | -- | lincRNA | 2.937135 | 1.722885 | 0.937455 | 1.031678 |
| LNC_003244 | XLOC_075117 | -- | lincRNA | 2.73755 | 0.089485 | 0.013187 | 0.010858 |
| LNC_003247 | XLOC_075117 | -- | lincRNA | 2.276577 | 0.738426 | 0 | 0.014012 |
| LNC_003246 | XLOC_075117 | -- | lincRNA | 10.714087 | 5.03323 | 2.933508 | 3.524037 |
| LNC_003241 | XLOC_075117 | -- | lincRNA | 0.740868 | 0.702013 | 0.312019 | 0.227304 |
| LNC_003240 | XLOC_075117 | -- | lincRNA | 0.35175 | 0.885803 | 0 | 0.096277 |
| LNC_003243 | XLOC_075117 | -- | lincRNA | 1.366803 | 0.126663 | 0 | 0.023065 |
| LNC_003242 | XLOC_075117 | -- | lincRNA | 0.880739 | 0.481104 | 0.717167 | 0.553405 |
| LNC_006436 | XLOC_154282 | -- | lincRNA | 1.563462 | 0 | 0.099275 | 0.122218 |
| LNC_004668 | XLOC_103749 | -- | intronic_lncRNA | 14.514283 | 22.155359 | 9.49343 | 12.577703 |
| LNC_004669 | XLOC_103756 | -- | intronic_lncRNA | 0.809255 | 0 | 0.148666 | 0.35913 |
| LNC_004662 | XLOC_103611 | -- | intronic_lncRNA | 0.498878 | 0.318043 | 0.296162 | 0.066145 |
| LNC_004663 | XLOC_103612 | -- | intronic_lncRNA | 0.443577 | 0.564938 | 0.017019 | 0 |
| LNC_004660 | XLOC_103491 | -- | intronic_lncRNA | 0.278132 | 0.036918 | 0.554616 | 0.317869 |
| LNC_004661 | XLOC_103588 | -- | intronic_lncRNA | 0.203454 | 0.461923 | 0.30755 | 0 |
| LNC_004666 | XLOC_103749 | -- | intronic_lncRNA | 2.93836 | 13.438206 | 13.328416 | 3.665345 |
| LNC_004667 | XLOC_103749 | -- | intronic_lncRNA | 4.606136 | 3.301437 | 2.03825 | 5.580842 |
| LNC_004664 | XLOC_103612 | -- | intronic_lncRNA | 0 | 0 | 0.530026 | 0.783181 |
| LNC_004665 | XLOC_103698 | -- | lincRNA | 0.275356 | 0.504817 | 0.198868 | 0.287137 |
| LNC_001522 | XLOC_032783 | -- | intronic_lncRNA | 0.531968 | 0.479433 | 0.344112 | 0.231364 |
| LNC_001523 | XLOC_032809 | -- | intronic_lncRNA | 0.719975 | 0.980089 | 0.761821 | 0.61983 |
| LNC_001520 | XLOC_032770 | -- | intronic_lncRNA | 0 | 0.609237 | 0 | 0 |
| LNC_001521 | XLOC_032774 | -- | intronic_lncRNA | 0 | 0.681955 | 0.438516 | 0.537084 |
| LNC_001526 | XLOC_032956 | -- | intronic_lncRNA | 0.353616 | 0.35448 | 1.000559 | 0.496147 |
| LNC_001527 | XLOC_032957 | -- | intronic_lncRNA | 0.522561 | 0.207838 | 0.651023 | 0.130114 |
| LNC_001524 | XLOC_032821 | -- | intronic_lncRNA | 0.041021 | 0.091183 | 0.736462 | 0.099284 |
| LNC_001525 | XLOC_032866 | -- | intronic_lncRNA | 1.425197 | 0.753206 | 1.131195 | 1.209409 |
| LNC_001528 | XLOC_033253 | -- | intronic_lncRNA | 0.394941 | 0.212808 | 0.588309 | 0.609236 |
| LNC_001529 | XLOC_033260 | -- | intronic_lncRNA | 0.492529 | 0.118173 | 0.386642 | 1.033037 |
| LNC_005957 | XLOC_141079 | -- | lincRNA | 1.33797 | 1.401001 | 1.367045 | 1.27339 |
| LNC_005956 | XLOC_141041 | -- | intronic_lncRNA | 0.372297 | 0.009351 | 0 | 0.019102 |
| LNC_005955 | XLOC_140975 | -- | lincRNA | 0.747935 | 0 | 0 | 0 |
| LNC_005954 | XLOC_140923 | -- | intronic_lncRNA | 1.065261 | 0.355578 | 0.97309 | 0.433869 |
| LNC_005953 | XLOC_140921 | -- | antisense_lncRNA | 0.326571 | 0.08146 | 0.329854 | 0.065959 |
| LNC_005952 | XLOC_140670 | -- | intronic_lncRNA | 0.471978 | 0.464891 | 0.192574 | 0.327828 |
| LNC_005951 | XLOC_140588 | -- | intronic_lncRNA | 0.23962 | 0.13227 | 0.789703 | 0.519057 |

| LNC_005950 | XLOC_140572 | -- | intronic_lncRNA | 0.580127 | 0.171461 | 0.24017 | 0.291129 |
| --- | --- | --- | --- | --- | --- | --- | --- |
| LNC_005449 | XLOC_125676 | -- | intronic_lncRNA | 0 | 1.113719 | 0 | 0 |
| LNC_005959 | XLOC_141104 | -- | intronic_lncRNA | 0.603591 | 0.551812 | 0.041939 | 0.606903 |
| LNC_005448 | XLOC_125586 | -- | intronic_lncRNA | 2.728779 | 0.607713 | 2.525788 | 1.791848 |
| LNC_005445 | XLOC_125538 | -- | intronic_lncRNA | 2.180471 | 1.856283 | 1.1149 | 1.007334 |
| LNC_005444 | XLOC_125538 | -- | intronic_lncRNA | 0.000182 | 0.00354 | 0.051877 | 0.003276 |
| LNC_000167 | XLOC_001947 | -- | intronic_lncRNA | 0.211797 | 0.280014 | 0.308474 | 0.61474 |
| LNC_005446 | XLOC_125558 | -- | intronic_lncRNA | 0.006046 | 0.017002 | 0.121507 | 1.173917 |
| LNC_005441 | XLOC_125538 | -- | intronic_lncRNA | 1.491618 | 1.313889 | 1.466844 | 1.27673 |
| LNC_005440 | XLOC_125527 | -- | intronic_lncRNA | 0 | 1.130645 | 0.187617 | 0.647656 |
| LNC_005443 | XLOC_125538 | -- | intronic_lncRNA | 0.214844 | 0.551022 | 0.885962 | 0.949964 |
| LNC_004723 | XLOC_105246 | -- | lincRNA | 0 | 0 | 0 | 0.39136 |
| LNC_005442 | XLOC_125538 | -- | intronic_lncRNA | 2.063947 | 0.089305 | 0.745905 | 1.124594 |
| LNC_000693 | XLOC_013800 | -- | intronic_lncRNA | 5.050541 | 1.05509 | 2.978786 | 1.792073 |
| LNC_000692 | XLOC_013800 | -- | intronic_lncRNA | 6.030815 | 1.020738 | 2.678256 | 1.766402 |
| LNC_000691 | XLOC_013769 | -- | intronic_lncRNA | 0.607228 | 0.418609 | 1.154936 | 0.975775 |
| LNC_000690 | XLOC_013765 | -- | intronic_lncRNA | 1.181707 | 0.532679 | 0.706455 | 0.480712 |
| LNC_000697 | XLOC_013805 | -- | intronic_lncRNA | 0.00165 | 0.16669 | 0.316456 | 0.753695 |
| LNC_000696 | XLOC_013805 | -- | intronic_lncRNA | 0.557747 | 0 | 0.008538 | 0.05602 |
| LNC_000695 | XLOC_013804 | -- | intronic_lncRNA | 0.693671 | 0.305137 | 0.481857 | 0.392294 |
| LNC_000694 | XLOC_013803 | -- | intronic_lncRNA | 0 | 0.721877 | 0.823543 | 0.288353 |
| LNC_000699 | XLOC_013938 | -- | intronic_lncRNA | 0.378896 | 0 | 0.407595 | 0.357329 |
| LNC_000698 | XLOC_013937 | -- | intronic_lncRNA | 0.562545 | 0 | 0 | 0 |
| LNC_003809 | XLOC_081816 | -- | lincRNA | 1.483605 | 1.818673 | 0.667454 | 0.370155 |
| LNC_003808 | XLOC_081780 | -- | intronic_lncRNA | 0.795498 | 0.904351 | 0.127275 | 0.180698 |
| LNC_003803 | XLOC_081673 | -- | intronic_lncRNA | 0.197366 | 0.802385 | 0.148643 | 0.29406 |
| LNC_003802 | XLOC_081652 | -- | intronic_lncRNA | 0 | 0 | 0 | 0.411958 |
| LNC_003801 | XLOC_081548 | -- | intronic_lncRNA | 0.215568 | 0 | 0.312605 | 0.859751 |
| LNC_003800 | XLOC_081544 | -- | intronic_lncRNA | 0.008655 | 0.500727 | 0.579166 | 0.330569 |
| LNC_003807 | XLOC_081689 | -- | intronic_lncRNA | 0.336826 | 0.579078 | 0.718342 | 0.874732 |
| LNC_003806 | XLOC_081685 | -- | intronic_lncRNA | 0.648693 | 0.95257 | 0.413413 | 0.589201 |
| LNC_003805 | XLOC_081677 | -- | intronic_lncRNA | 0 | 0.616603 | 0.126086 | 0.287206 |
| LNC_003804 | XLOC_081676 | -- | intronic_lncRNA | 0.86868 | 0.602183 | 1.118614 | 0.723758 |
| LNC_000345 | XLOC_005409 | -- | intronic_lncRNA | 0.102137 | 0.523317 | 0.138673 | 0.202472 |
| LNC_000344 | XLOC_005407 | -- | intronic_lncRNA | 1.235185 | 0 | 1.211563 | 0.971143 |
| LNC_000347 | XLOC_005519 | -- | intronic_lncRNA | 0.484193 | 0.588704 | 0.004996 | 0 |
| LNC_000346 | XLOC_005519 | -- | intronic_lncRNA | 2.396031 | 0.651897 | 0.361581 | 0.292698 |
| LNC_000341 | XLOC_005355 | -- | intronic_lncRNA | 1.407952 | 0.433392 | 0.654938 | 0.773655 |
| LNC_000340 | XLOC_005152 | -- | intronic_lncRNA | 0.166499 | 0 | 0 | 0.703041 |
| LNC_000343 | XLOC_005407 | -- | intronic_lncRNA | 0.535712 | 0.059339 | 0.395508 | 0.41803 |
| LNC_000342 | XLOC_005379 | -- | intronic_lncRNA | 0.800683 | 0 | 0 | 0.129375 |
| LNC_000349 | XLOC_005576 | -- | lincRNA | 0.006327 | 0.02483 | 0.831452 | 0.579621 |
| LNC_000348 | XLOC_005563 | -- | lincRNA | 0.265567 | 0.352067 | 0.4047 | 0.255238 |
| LNC_005320 | XLOC_121702 | -- | intronic_lncRNA | 0.435074 | 0 | 0.393275 | 0.205777 |
| LNC_005321 | XLOC_121713 | -- | intronic_lncRNA | 0 | 0 | 0.212898 | 0.639273 |
| LNC_005322 | XLOC_121758 | -- | intronic_lncRNA | 0.57412 | 0.250619 | 0.115978 | 0.27057 |
| LNC_005323 | XLOC_121769 | -- | intronic_lncRNA | 0.193385 | 0 | 0.276302 | 0.549745 |
| LNC_005324 | XLOC_121777 | -- | intronic_lncRNA | 0.468547 | 0.117266 | 0.952359 | 0.493227 |
| LNC_005325 | XLOC_121842 | -- | antisense_lncRNA | 0 | 0 | 0.277663 | 0.807879 |
| LNC_005326 | XLOC_121847 | -- | intronic_lncRNA | 0.697868 | 0.290412 | 0.409862 | 0.806959 |
| LNC_005327 | XLOC_121848 | -- | intronic_lncRNA | 0.714877 | 0.403505 | 0.711686 | 0.260907 |
| LNC_005328 | XLOC_121854 | -- | intronic_lncRNA | 0.197165 | 0.634262 | 0.149417 | 0 |
| LNC_005329 | XLOC_121932 | -- | intronic_lncRNA | 0.253758 | 0.653585 | 0.586408 | 0.585134 |
| LNC_003426 | XLOC_075207 | -- | lincRNA | 1.036807 | 0.86761 | 0.199236 | 0.997543 |
| LNC_004051 | XLOC_087531 | -- | intronic_lncRNA | 0 | 0 | 0 | 0.410159 |
| LNC_004050 | XLOC_087515 | -- | intronic_lncRNA | 0 | 0 | 0.204297 | 0.529724 |
| LNC_004053 | XLOC_087543 | -- | lincRNA | 0 | 0.150865 | 0.060204 | 0.48717 |
| LNC_004052 | XLOC_087543 | -- | lincRNA | 0.156613 | 0.642441 | 0.256504 | 0.287459 |
| LNC_004055 | XLOC_087573 | -- | intronic_lncRNA | 0.562871 | 0.45127 | 0.430699 | 0.57378 |
| LNC_004054 | XLOC_087553 | -- | intronic_lncRNA | 0.569898 | 0 | 0.18578 | 0.249652 |
| LNC_004057 | XLOC_087683 | -- | lincRNA | 0.087778 | 0.13482 | 0.373325 | 0.427949 |
| LNC_004056 | XLOC_087665 | -- | intronic_lncRNA | 0.337963 | 0.562009 | 0.060686 | 0.499565 |
| LNC_004059 | XLOC_087716 | -- | intronic_lncRNA | 0.218095 | 0.520173 | 0.421267 | 0 |
| LNC_004058 | XLOC_087715 | -- | intronic_lncRNA | 0 | 0.150623 | 0.166762 | 0.695957 |
| LNC_000976 | XLOC_020917 | -- | intronic_lncRNA | 0 | 0.569706 | 0.517021 | 1.56566 |
| LNC_000977 | XLOC_020931 | -- | intronic_lncRNA | 0.549747 | 0.509728 | 0.711649 | 0.749506 |
| LNC_000974 | XLOC_020916 | -- | intronic_lncRNA | 1.184456 | 0.958287 | 0.570875 | 0.958981 |
| LNC_000975 | XLOC_020917 | -- | intronic_lncRNA | 0.22771 | 0.379324 | 1.964639 | 2.119451 |
| LNC_000972 | XLOC_020858 | -- | intronic_lncRNA | 0 | 0.242885 | 0.240211 | 0 |
| LNC_000973 | XLOC_020876 | -- | lincRNA | 0.690376 | 2.109191 | 0.188555 | 0.164357 |
| LNC_000970 | XLOC_020744 | -- | intronic_lncRNA | 0.26415 | 0 | 1.00366 | 0.408085 |
| LNC_000971 | XLOC_020771 | -- | intronic_lncRNA | 0.515982 | 0.166395 | 0.911982 | 0.943133 |
| LNC_000978 | XLOC_020990 | -- | intronic_lncRNA | 0 | 0 | 0.850607 | 0 |
| LNC_000979 | XLOC_020991 | -- | intronic_lncRNA | 0.198034 | 0.455254 | 0.32199 | 0.425397 |
| LNC_004589 | XLOC_101481 | -- | intronic_lncRNA | 0.483289 | 0.41778 | 0.659365 | 0.861833 |
| LNC_004588 | XLOC_101475 | -- | intronic_lncRNA | 0.372723 | 0.303193 | 0.500117 | 0.272393 |
| LNC_004583 | XLOC_100920 | -- | lincRNA | 1.59633 | 0.701731 | 0.446834 | 0.38343 |

| LNC_004582 | XLOC_100541 | -- | intronic_lncRNA | 0 | 0 | 0.618646 | 0.07159 |
| --- | --- | --- | --- | --- | --- | --- | --- |
| LNC_004581 | XLOC_100530 | -- | intronic_lncRNA | 0.915164 | 0.222826 | 0.326792 | 0.212739 |
| LNC_004580 | XLOC_100528 | -- | intronic_lncRNA | 0.479064 | 0.122878 | 0.455689 | 0.805961 |
| LNC_004587 | XLOC_101411 | -- | lincRNA | 0.811866 | 0.062556 | 0 | 0 |
| LNC_004586 | XLOC_101229 | -- | lincRNA | 1.30285 | 0.239766 | 0.11455 | 0.054238 |
| LNC_004585 | XLOC_101209 | -- | lincRNA | 0.725479 | 0.180556 | 0.074913 | 0.029505 |
| LNC_004584 | XLOC_100920 | -- | lincRNA | 0.987759 | 0 | 0.236104 | 0.48483 |
| LNC_005933 | XLOC_140159 | -- | intronic_lncRNA | 0.237586 | 0.127192 | 0 | 0.411958 |
| LNC_002196 | XLOC_050092 | -- | intronic_lncRNA | 0 | 0 | 0.544874 | 0.067524 |
| LNC_002197 | XLOC_050106 | -- | intronic_lncRNA | 0.712326 | 2.93166 | 0.545431 | 1.12147 |
| LNC_002194 | XLOC_050056 | -- | lincRNA | 0 | 0 | 0 | 0.527676 |
| LNC_002195 | XLOC_050059 | -- | intronic_lncRNA | 0.110842 | 0.311824 | 0.615172 | 0.554132 |
| LNC_002192 | XLOC_050030 | -- | intronic_lncRNA | 0.788228 | 0.992885 | 0.244597 | 0.385496 |
| LNC_002193 | XLOC_050030 | -- | intronic_lncRNA | 0.358713 | 0.182131 | 0.367803 | 0.175133 |
| LNC_002190 | XLOC_050011 | -- | intronic_lncRNA | 1.36955 | 1.551328 | 1.562012 | 1.187294 |
| LNC_002191 | XLOC_050029 | -- | intronic_lncRNA | 0.342041 | 0.524039 | 0.160682 | 0.439536 |
| LNC_002198 | XLOC_050108 | -- | intronic_lncRNA | 0 | 0.56038 | 0 | 0 |
| LNC_002199 | XLOC_050109 | -- | intronic_lncRNA | 0.244928 | 0.08146 | 0.489064 | 0.66839 |
| LNC_003586 | XLOC_075948 | -- | intronic_lncRNA | 0.280606 | 0.283725 | 0.663776 | 1.00484 |
| LNC_003587 | XLOC_075949 | -- | intronic_lncRNA | 0.910426 | 0.420766 | 0.319233 | 0.756812 |
| LNC_003584 | XLOC_075904 | -- | intronic_lncRNA | 0.325702 | 0 | 0.044096 | 0.554402 |
| LNC_003585 | XLOC_075905 | -- | intronic_lncRNA | 0.637497 | 1.200104 | 0.770387 | 0.951382 |
| LNC_003582 | XLOC_075883 | -- | intronic_lncRNA | 0.343448 | 0.170291 | 0.648236 | 0.800176 |
| LNC_003583 | XLOC_075891 | -- | intronic_lncRNA | 0.180056 | 0.488617 | 0.277249 | 0.331917 |
| LNC_003580 | XLOC_075791 | -- | lincRNA | 1.706881 | 0.868333 | 0.144233 | 0.213841 |
| LNC_003581 | XLOC_075882 | -- | intronic_lncRNA | 0.240905 | 0 | 0.719075 | 0.297146 |
| LNC_003588 | XLOC_075960 | -- | lincRNA | 0.499682 | 0 | 0 | 0 |
| LNC_003589 | XLOC_076023 | -- | intronic_lncRNA | 0.3971 | 0.135548 | 0.867467 | 0.927464 |
| LNC_002228 | XLOC_051072 | -- | intronic_lncRNA | 0 | 0 | 1.423592 | 0.569849 |
| LNC_002229 | XLOC_051073 | -- | intronic_lncRNA | 0 | 0.895742 | 0 | 0 |
| LNC_005934 | XLOC_140170 | -- | intronic_lncRNA | 0.672199 | 0.286416 | 0.309856 | 0.816348 |
| LNC_002220 | XLOC_050833 | -- | intronic_lncRNA | 0.479116 | 0.746192 | 0.921225 | 0.741444 |
| LNC_002221 | XLOC_050840 | -- | intronic_lncRNA | 0.149373 | 0.171185 | 0.945242 | 0.235058 |
| LNC_002222 | XLOC_050842 | -- | intronic_lncRNA | 1.183589 | 0.250677 | 0.787187 | 1.463686 |
| LNC_002223 | XLOC_050898 | -- | intronic_lncRNA | 0.202117 | 0.655468 | 0.810428 | 0.503755 |
| LNC_002224 | XLOC_050926 | -- | lincRNA | 0 | 0 | 0 | 0.479038 |
| LNC_002225 | XLOC_050948 | -- | intronic_lncRNA | 0.07046 | 0.50799 | 0.275491 | 0.137263 |
| LNC_002226 | XLOC_050957 | -- | intronic_lncRNA | 0.168492 | 0 | 0.883151 | 0.570364 |
| LNC_002227 | XLOC_051065 | -- | lincRNA | 0.557417 | 0.139212 | 0.144167 | 0.392738 |
| LNC_003058 | XLOC_070257 | -- | intronic_lncRNA | 0.180265 | 0 | 0.046843 | 0.557527 |
| LNC_003059 | XLOC_070282 | -- | intronic_lncRNA | 0.280315 | 0.612669 | 0 | 0 |
| LNC_003050 | XLOC_070205 | -- | intronic_lncRNA | 0 | 0 | 0 | 0.41745 |
| LNC_003051 | XLOC_070216 | -- | intronic_lncRNA | 0.862845 | 0.062141 | 0 | 0 |
| LNC_003052 | XLOC_070224 | -- | intronic_lncRNA | 1.074003 | 0.550025 | 0.494103 | 0.416983 |
| LNC_003053 | XLOC_070252 | -- | intronic_lncRNA | 0 | 0 | 0.415675 | 0 |
| LNC_003054 | XLOC_070253 | -- | intronic_lncRNA | 0.913988 | 0 | 0 | 0 |
| LNC_003055 | XLOC_070254 | -- | intronic_lncRNA | 0.960814 | 0 | 0 | 0 |
| LNC_003056 | XLOC_070255 | -- | intronic_lncRNA | 0.775062 | 0 | 0 | 0 |
| LNC_003057 | XLOC_070256 | -- | intronic_lncRNA | 0.550992 | 0.274878 | 0 | 0.082352 |
| LNC_000590 | XLOC_011012 | -- | intronic_lncRNA | 0.283812 | 0.26665 | 0.135378 | 0.54185 |
| LNC_000473 | XLOC_008377 | -- | intronic_lncRNA | 0.313527 | 0.151698 | 0.528888 | 0.515762 |
| LNC_000592 | XLOC_011062 | -- | lincRNA | 0.822512 | 0.216508 | 0.399668 | 0.54756 |
| LNC_000593 | XLOC_011171 | -- | lincRNA | 0.298418 | 0.501229 | 0.627637 | 0.434458 |
| LNC_000594 | XLOC_011179 | -- | intronic_lncRNA | 0.044738 | 0.508086 | 0.380244 | 0.232773 |
| LNC_000595 | XLOC_011281 | -- | intronic_lncRNA | 0.88664 | 0.448331 | 0.557453 | 0.164935 |
| LNC_000596 | XLOC_011281 | -- | intronic_lncRNA | 0.013517 | 0.158679 | 0 | 0.392369 |
| LNC_005112 | XLOC_116905 | -- | intronic_lncRNA | 0.182853 | 0.853155 | 0.712636 | 0.59506 |
| LNC_000598 | XLOC_011398 | -- | intronic_lncRNA | 0.587589 | 0.771303 | 0.716479 | 0.779586 |
| LNC_000599 | XLOC_011414 | -- | intronic_lncRNA | 0.145015 | 0.534626 | 0.341959 | 0.493641 |
| LNC_000471 | XLOC_008358 | -- | intronic_lncRNA | 0.722601 | 0.153652 | 0.181894 | 0 |
| LNC_000470 | XLOC_008311 | -- | intronic_lncRNA | 0.108807 | 0.293486 | 0.136184 | 0.446699 |
| LNC_005117 | XLOC_117027 | -- | intronic_lncRNA | 0.473054 | 0.517763 | 0.76625 | 0.716899 |
| LNC_005116 | XLOC_117022 | -- | antisense_lncRNA | 0 | 0 | 0.251207 | 0.585945 |
| LNC_005115 | XLOC_117007 | -- | intronic_lncRNA | 0.378551 | 0.086852 | 0.438439 | 0.635883 |
| LNC_005114 | XLOC_116940 | -- | intronic_lncRNA | 26.347559 | 32.099258 | 38.554493 | 39.27747 |
| LNC_000479 | XLOC_008408 | -- | intronic_lncRNA | 6.07178 | 15.269597 | 10.819878 | 10.934102 |
| LNC_005893 | XLOC_138833 | -- | intronic_lncRNA | 0.218797 | 0.569818 | 0 | 0.078728 |
| LNC_005118 | XLOC_117068 | -- | intronic_lncRNA | 2.53563 | 1.802108 | 1.706771 | 2.981951 |
| LNC_001759 | XLOC_038792 | -- | intronic_lncRNA | 1.155249 | 1.07603 | 0.873638 | 0.931632 |
| LNC_005362 | XLOC_123217 | -- | lincRNA | 1.085656 | 0 | 0.711477 | 0.545602 |
| LNC_001751 | XLOC_038723 | -- | intronic_lncRNA | 0.295016 | 0.224773 | 0.335882 | 0.821343 |
| LNC_001750 | XLOC_038711 | -- | intronic_lncRNA | 0 | 0 | 0.40425 | 0 |
| LNC_001753 | XLOC_038724 | -- | intronic_lncRNA | 0.111073 | 0.406822 | 0.288472 | 0.539385 |
| LNC_001752 | XLOC_038724 | -- | intronic_lncRNA | 0.151312 | 0 | 0.458074 | 0.340338 |
| LNC_001755 | XLOC_038784 | -- | intronic_lncRNA | 1.074709 | 0.776877 | 1.231705 | 2.026141 |
| LNC_001754 | XLOC_038747 | -- | lincRNA | 0.520411 | 0.150583 | 0.473435 | 0 |
| LNC_001757 | XLOC_038788 | -- | intronic_lncRNA | 0.159276 | 0 | 0.947171 | 0.450652 |

| LNC_001756 | XLOC_038784 | -- | intronic_lncRNA | 0 | 0 | 0.004542 | 0.446907 |
| --- | --- | --- | --- | --- | --- | --- | --- |
| LNC_004873 | XLOC_110520 | -- | lincRNA | 0.492982 | 0.667484 | 0.433974 | 0.903203 |
| LNC_004872 | XLOC_110430 | -- | intronic_lncRNA | 0.175746 | 0.302642 | 0.309813 | 0.662554 |
| LNC_004871 | XLOC_110134 | -- | intronic_lncRNA | 0.087151 | 0.347242 | 0.344832 | 0.73578 |
| LNC_004870 | XLOC_110131 | -- | intronic_lncRNA | 0.081414 | 0 | 0.394717 | 0.392456 |
| LNC_004877 | XLOC_110626 | -- | intronic_lncRNA | 0.904239 | 0.452622 | 0.828905 | 0.736677 |
| LNC_004876 | XLOC_110561 | -- | lincRNA | 0.941006 | 1.041916 | 0.999664 | 0.874378 |
| LNC_004875 | XLOC_110537 | -- | lincRNA | 0.687974 | 0.083679 | 0 | 0 |
| LNC_004874 | XLOC_110522 | -- | intronic_lncRNA | 0.636595 | 0.134129 | 0.05362 | 0 |
| LNC_004879 | XLOC_110662 | -- | intronic_lncRNA | 0.571376 | 0.554689 | 0.39452 | 1.158296 |
| LNC_004878 | XLOC_110662 | -- | intronic_lncRNA | 0.092309 | 0.107719 | 0.746722 | 0.305075 |
| LNC_001348 | XLOC_029378 | -- | antisense_lncRNA | 0.48668 | 0 | 0 | 0 |
| LNC_001349 | XLOC_029381 | -- | intronic_lncRNA | 1.062104 | 0.199093 | 0.178945 | 0.190838 |
| LNC_001346 | XLOC_029307 | -- | intronic_lncRNA | 0 | 0 | 0 | 0.426938 |
| LNC_001347 | XLOC_029378 | -- | intronic_lncRNA | 1.436626 | 0.539194 | 0.676579 | 0.757667 |
| LNC_001344 | XLOC_029240 | -- | lincRNA | 0.297438 | 0.694009 | 0.475066 | 0.458125 |
| LNC_001345 | XLOC_029246 | -- | intronic_lncRNA | 0.101271 | 0 | 0.16339 | 0.408814 |
| LNC_001342 | XLOC_029209 | -- | antisense_lncRNA | 0.819597 | 1.976186 | 0.105439 | 0.743002 |
| LNC_001343 | XLOC_029234 | -- | intronic_lncRNA | 1.021389 | 0.729765 | 2.488135 | 1.774418 |
| LNC_001340 | XLOC_029199 | -- | intronic_lncRNA | 0.478884 | 0.262074 | 0.189511 | 0.068124 |
| LNC_001341 | XLOC_029200 | -- | intronic_lncRNA | 0 | 0.115078 | 0.745576 | 0 |
| LNC_003616 | XLOC_077112 | -- | intronic_lncRNA | 0.208368 | 0 | 0.544594 | 0.318533 |
| LNC_003617 | XLOC_077116 | -- | intronic_lncRNA | 0.903044 | 0.159893 | 0.147009 | 0.175657 |
| LNC_003614 | XLOC_077033 | -- | intronic_lncRNA | 0.702237 | 0.077852 | 0.125627 | 0.456604 |
| LNC_003615 | XLOC_077034 | -- | intronic_lncRNA | 0.537459 | 0.224315 | 0.368686 | 0.139662 |
| LNC_003612 | XLOC_077018 | -- | intronic_lncRNA | 0.353214 | 0.582594 | 0.623226 | 0.616663 |
| LNC_003613 | XLOC_077031 | -- | intronic_lncRNA | 0.555984 | 0.837103 | 0.360754 | 0.403535 |
| LNC_003610 | XLOC_076963 | -- | lincRNA | 0 | 0.759816 | 0 | 0 |
| LNC_003611 | XLOC_077012 | -- | lincRNA | 0.438344 | 0 | 0.06461 | 0.18346 |
| LNC_003618 | XLOC_077123 | -- | intronic_lncRNA | 0.94061 | 0.492684 | 0.342427 | 0.389948 |
| LNC_003619 | XLOC_077132 | -- | intronic_lncRNA | 0.440157 | 0 | 0.326482 | 0.471653 |
| LNC_001968 | XLOC_044355 | -- | lincRNA | 0 | 0 | 0.460503 | 0 |
| LNC_001969 | XLOC_044376 | -- | intronic_lncRNA | 0.888201 | 1.290189 | 2.541526 | 1.320151 |
| LNC_001962 | XLOC_044137 | -- | intronic_lncRNA | 0.509379 | 0.627434 | 0.242581 | 0.647249 |
| LNC_001963 | XLOC_044149 | -- | intronic_lncRNA | 0 | 0.250624 | 0 | 0.500792 |
| LNC_001960 | XLOC_044135 | -- | intronic_lncRNA | 1.32809 | 0.823247 | 0 | 0 |
| LNC_001961 | XLOC_044135 | -- | intronic_lncRNA | 0.5279 | 0.651828 | 1.587911 | 1.893616 |
| LNC_001966 | XLOC_044315 | -- | intronic_lncRNA | 1.930024 | 2.10575 | 2.490558 | 2.713722 |
| LNC_001967 | XLOC_044339 | -- | lincRNA | 0.037812 | 0 | 0.214535 | 0.649434 |
| LNC_001964 | XLOC_044247 | -- | intronic_lncRNA | 0.713517 | 0.117225 | 0.51816 | 0.866124 |
| LNC_001965 | XLOC_044314 | -- | intronic_lncRNA | 0.953367 | 0 | 0 | 0 |
| LNC_005519 | XLOC_127524 | -- | intronic_lncRNA | 0.816943 | 0.428831 | 0.876189 | 0.871329 |
| LNC_005518 | XLOC_127463 | -- | intronic_lncRNA | 0.758993 | 1.218654 | 1.263801 | 2.074366 |
| LNC_005517 | XLOC_127460 | -- | intronic_lncRNA | 0.186933 | 0 | 0.543686 | 0.525759 |
| LNC_005516 | XLOC_127457 | -- | intronic_lncRNA | 0.17021 | 0.492805 | 0.140934 | 0.173119 |
| LNC_005515 | XLOC_127395 | -- | intronic_lncRNA | 0.790029 | 0.212528 | 0 | 0.484992 |
| LNC_005514 | XLOC_127379 | -- | lincRNA | 0.521766 | 0.414614 | 0.580381 | 1.120955 |
| LNC_005513 | XLOC_127374 | -- | intronic_lncRNA | 0.097002 | 0 | 0.223381 | 0.688385 |
| LNC_005512 | XLOC_127370 | -- | intronic_lncRNA | 0 | 0 | 0.442793 | 0.057744 |
| LNC_005511 | XLOC_127369 | -- | intronic_lncRNA | 0 | 0 | 0.655402 | 0.26202 |
| LNC_005510 | XLOC_127350 | -- | intronic_lncRNA | 0.106496 | 0.846065 | 1.369208 | 0.636558 |
| LNC_005830 | XLOC_137316 | -- | lincRNA | 0 | 0.112344 | 0.420638 | 0 |
| LNC_002057 | XLOC_046218 | -- | lincRNA | 0.296403 | 1.34507 | 0.082033 | 0 |
| LNC_002056 | XLOC_046213 | -- | intronic_lncRNA | 0.077037 | 0.649605 | 0.266217 | 0.386078 |
| LNC_002055 | XLOC_046213 | -- | intronic_lncRNA | 0.72756 | 0 | 0.286745 | 0 |
| LNC_002054 | XLOC_046213 | -- | intronic_lncRNA | 0.44534 | 0.143848 | 0.412959 | 0.466091 |
| LNC_002053 | XLOC_046144 | -- | intronic_lncRNA | 0.688839 | 0 | 1.038238 | 0.133952 |
| LNC_002052 | XLOC_046087 | -- | intronic_lncRNA | 0.918536 | 0 | 0 | 0 |
| LNC_002051 | XLOC_046087 | -- | intronic_lncRNA | 0.598715 | 0.698618 | 0.852336 | 1.006371 |
| LNC_002050 | XLOC_046033 | -- | intronic_lncRNA | 0.457437 | 0.155671 | 0.522409 | 1.003394 |
| LNC_002059 | XLOC_046240 | -- | intronic_lncRNA | 0.700546 | 2.725844 | 4.01182 | 4.837216 |
| LNC_002058 | XLOC_046237 | -- | intronic_lncRNA | 0.078248 | 0 | 0.579892 | 0 |
| LNC_003267 | XLOC_075141 | -- | lincRNA | 2.113922 | 0.054928 | 0.014445 | 0.037672 |
| LNC_003266 | XLOC_075141 | -- | lincRNA | 1.210464 | 0 | 0 | 0 |
| LNC_003265 | XLOC_075141 | -- | lincRNA | 3.325714 | 1.383802 | 1.332022 | 1.526943 |
| LNC_003264 | XLOC_075140 | -- | lincRNA | 5.267916 | 1.936325 | 0.956251 | 1.293201 |
| LNC_003263 | XLOC_075138 | -- | lincRNA | 1.465646 | 0 | 0.014302 | 0 |
| LNC_003262 | XLOC_075138 | -- | lincRNA | 2.575131 | 1.109572 | 0.653411 | 0.244231 |
| LNC_003261 | XLOC_075136 | -- | lincRNA | 1.610646 | 1.003048 | 0.22091 | 0.312015 |
| LNC_003260 | XLOC_075135 | -- | lincRNA | 2.556817 | 1.08904 | 0.917877 | 0.679509 |
| LNC_003269 | XLOC_075141 | -- | lincRNA | 3.564631 | 2.01412 | 1.147995 | 1.137401 |
| LNC_003268 | XLOC_075141 | -- | lincRNA | 0.109552 | 1.057489 | 0.030682 | 0.035069 |
| LNC_006404 | XLOC_153429 | -- | intronic_lncRNA | 0.317924 | 0.165198 | 0.385714 | 0.447829 |
| LNC_006405 | XLOC_153616 | -- | intronic_lncRNA | 0.762487 | 0.256634 | 0.410595 | 0.733771 |
| LNC_006406 | XLOC_153650 | -- | intronic_lncRNA | 1.144947 | 0.244155 | 0.86948 | 0.810962 |
| LNC_006407 | XLOC_153683 | -- | intronic_lncRNA | 0.608396 | 0 | 0 | 0 |
| LNC_006400 | XLOC_153404 | -- | intronic_lncRNA | 0 | 0 | 0.654788 | 0 |

| LNC_006401 | XLOC_153425 | -- | intronic_lncRNA | 0.434592 | 0.685184 | 1.188086 | 0.89198 |
| --- | --- | --- | --- | --- | --- | --- | --- |
| LNC_006402 | XLOC_153426 | -- | intronic_lncRNA | 0.376503 | 0.985831 | 0.57282 | 0.366033 |
| LNC_006403 | XLOC_153428 | -- | intronic_lncRNA | 0.488768 | 0 | 0.116619 | 0.483703 |
| LNC_004648 | XLOC_103289 | -- | intronic_lncRNA | 0.236941 | 0.23641 | 0.444185 | 0.535993 |
| LNC_004649 | XLOC_103299 | -- | intronic_lncRNA | 0.086088 | 0 | 0.20245 | 0.436867 |
| LNC_004644 | XLOC_103239 | -- | intronic_lncRNA | 0.532212 | 0 | 0.42398 | 0.548444 |
| LNC_004645 | XLOC_103244 | -- | intronic_lncRNA | 3.023877 | 2.05033 | 0.90879 | 0.924971 |
| LNC_004646 | XLOC_103244 | -- | intronic_lncRNA | 28.039276 | 6.157156 | 26.218817 | 23.552177 |
| LNC_004647 | XLOC_103256 | -- | intronic_lncRNA | 23.486652 | 6.993937 | 10.417532 | 10.249102 |
| LNC_004640 | XLOC_103133 | -- | intronic_lncRNA | 0.1355 | 0.515099 | 0.509981 | 0.432655 |
| LNC_004641 | XLOC_103207 | -- | intronic_lncRNA | 0.693523 | 0 | 0 | 0.241554 |
| LNC_004642 | XLOC_103207 | -- | intronic_lncRNA | 0 | 0.141305 | 0.316208 | 0.533113 |
| LNC_004643 | XLOC_103208 | -- | intronic_lncRNA | 0.159828 | 0.38334 | 0.643669 | 0.282301 |
| LNC_001504 | XLOC_032605 | -- | intronic_lncRNA | 1.394197 | 23.318531 | 0 | 3.189522 |
| LNC_001505 | XLOC_032607 | -- | intronic_lncRNA | 1.152115 | 1.635575 | 1.185409 | 1.042949 |
| LNC_001506 | XLOC_032613 | -- | intronic_lncRNA | 3.762857 | 3.23701 | 1.880693 | 1.926848 |
| LNC_001507 | XLOC_032672 | -- | intronic_lncRNA | 3.711486 | 5.088825 | 14.223354 | 13.553248 |
| LNC_001500 | XLOC_032605 | -- | intronic_lncRNA | 1.867612 | 8.992703 | 0.012331 | 2.250332 |
| LNC_001501 | XLOC_032605 | -- | intronic_lncRNA | 0 | 0 | 0 | 7.050861 |
| LNC_001502 | XLOC_032605 | -- | intronic_lncRNA | 5.50196 | 8.366436 | 8.450164 | 6.498074 |
| LNC_001503 | XLOC_032605 | -- | intronic_lncRNA | 1.123756 | 1.05805 | 6.463588 | 0 |
| LNC_001508 | XLOC_032684 | -- | intronic_lncRNA | 0 | 0.141776 | 0 | 0.587153 |
| LNC_001509 | XLOC_032695 | -- | intronic_lncRNA | 0.986843 | 0.616573 | 0.412297 | 0.303879 |
| LNC_005580 | XLOC_129826 | -- | intronic_lncRNA | 0.654778 | 0.185469 | 0.314795 | 0.474226 |
| LNC_000061 | XLOC_000611 | -- | lincRNA | 0.428063 | 3.267554 | 64.182304 | 43.651272 |
| LNC_005582 | XLOC_129902 | -- | intronic_lncRNA | 0.183986 | 0.219857 | 2.406485 | 2.302417 |
| LNC_005583 | XLOC_129992 | -- | lincRNA | 0.74339 | 0.226254 | 0.258391 | 0.09507 |
| LNC_005584 | XLOC_130014 | -- | intronic_lncRNA | 0.525356 | 0.451375 | 0.393065 | 0 |
| LNC_005585 | XLOC_130015 | -- | intronic_lncRNA | 0.213729 | 0.11717 | 0.554799 | 0.308344 |
| LNC_003869 | XLOC_083274 | -- | intronic_lncRNA | 0.073997 | 0.786264 | 0.092463 | 0.428955 |
| LNC_003868 | XLOC_083267 | -- | intronic_lncRNA | 0.163376 | 0.811363 | 0.384934 | 0.339137 |
| LNC_003865 | XLOC_083013 | -- | intronic_lncRNA | 0.271526 | 0.635789 | 1.88162 | 1.591787 |
| LNC_003864 | XLOC_082943 | -- | lincRNA | 32.870338 | 15.291305 | 18.236473 | 29.799101 |
| LNC_003867 | XLOC_083267 | -- | intronic_lncRNA | 1.376007 | 0.375683 | 0.860938 | 0.945823 |
| LNC_003866 | XLOC_083093 | -- | lincRNA | 0.97423 | 1.009102 | 0.561575 | 0.530658 |
| LNC_003861 | XLOC_082870 | -- | intronic_lncRNA | 0.347962 | 0.171768 | 0.387637 | 0.535274 |
| LNC_003860 | XLOC_082870 | -- | intronic_lncRNA | 0 | 0 | 0 | 0.517477 |
| LNC_003863 | XLOC_082919 | -- | lincRNA | 0.385817 | 0 | 0.465062 | 0 |
| LNC_003862 | XLOC_082911 | -- | lincRNA | 0.82741 | 0.319595 | 0 | 0.758171 |
| LNC_005588 | XLOC_130135 | -- | lincRNA | 0 | 0 | 0.479299 | 0 |
| LNC_000369 | XLOC_006160 | -- | intronic_lncRNA | 0.574265 | 0.488645 | 0 | 0 |
| LNC_000368 | XLOC_006144 | -- | intronic_lncRNA | 1.008609 | 0.585008 | 0.101669 | 0.130286 |
| LNC_000367 | XLOC_006144 | -- | intronic_lncRNA | 3.666099 | 7.048637 | 3.405778 | 4.105849 |
| LNC_000366 | XLOC_006141 | -- | intronic_lncRNA | 0.198052 | 0.24808 | 0.415675 | 0.326941 |
| LNC_000365 | XLOC_006097 | -- | intronic_lncRNA | 0.39189 | 0.53041 | 0.670314 | 0.38775 |
| LNC_000364 | XLOC_006038 | -- | intronic_lncRNA | 1.11928 | 0.561624 | 1.143916 | 0.908708 |
| LNC_000363 | XLOC_006035 | -- | intronic_lncRNA | 0.971824 | 0.854045 | 0.30265 | 0.482543 |
| LNC_000362 | XLOC_005973 | -- | intronic_lncRNA | 0.378304 | 0.675124 | 0 | 0 |
| LNC_000361 | XLOC_005954 | -- | intronic_lncRNA | 0.718185 | 0.144277 | 0 | 0 |
| LNC_000360 | XLOC_005868 | -- | intronic_lncRNA | 0.965682 | 0.098504 | 0.669905 | 0.876179 |
| LNC_002732 | XLOC_063510 | -- | intronic_lncRNA | 2.461349 | 3.8269 | 4.00181 | 3.571559 |
| LNC_002733 | XLOC_063521 | -- | intronic_lncRNA | 0 | 0.073242 | 0 | 0.553811 |
| LNC_002730 | XLOC_063471 | -- | intronic_lncRNA | 2.178022 | 2.144668 | 2.210112 | 1.75009 |
| LNC_002731 | XLOC_063509 | -- | intronic_lncRNA | 0.512155 | 0 | 0 | 0 |
| LNC_002736 | XLOC_063550 | -- | intronic_lncRNA | 0 | 0.788014 | 0 | 0 |
| LNC_002737 | XLOC_063552 | -- | intronic_lncRNA | 1.132259 | 0.164166 | 0.87295 | 0.15996 |
| LNC_002734 | XLOC_063522 | -- | lincRNA | 0.102789 | 0.207909 | 0.495068 | 0.365651 |
| LNC_002735 | XLOC_063541 | -- | intronic_lncRNA | 1.019491 | 0.22171 | 1.152855 | 0.822255 |
| LNC_002738 | XLOC_063588 | -- | intronic_lncRNA | 0.746845 | 0.58812 | 0.762172 | 1.277392 |
| LNC_002739 | XLOC_063614 | -- | intronic_lncRNA | 0.724193 | 0.812917 | 1.135736 | 1.452721 |
| LNC_002884 | XLOC_066315 | -- | intronic_lncRNA | 0.721671 | 0.961203 | 0.766278 | 1.37459 |
| LNC_002885 | XLOC_066326 | -- | lincRNA | 3.048154 | 14.400642 | 3.045584 | 2.111177 |
| LNC_002886 | XLOC_066339 | -- | intronic_lncRNA | 0.538675 | 0.003407 | 0.032662 | 0.130145 |
| LNC_002887 | XLOC_066407 | -- | intronic_lncRNA | 0 | 0 | 0.64788 | 0 |
| LNC_002880 | XLOC_066265 | -- | intronic_lncRNA | 0.352141 | 0.627906 | 0.27081 | 0.069389 |
| LNC_002881 | XLOC_066266 | -- | intronic_lncRNA | 0.500484 | 0.392645 | 0.445225 | 0 |
| LNC_002882 | XLOC_066281 | -- | intronic_lncRNA | 61.331097 | 62.956909 | 83.472885 | 87.728043 |
| LNC_002883 | XLOC_066285 | -- | intronic_lncRNA | 14.594058 | 41.734776 | 18.146673 | 20.340256 |
| LNC_002888 | XLOC_066475 | -- | intronic_lncRNA | 0.468091 | 3.584505 | 0.255279 | 0.565345 |
| LNC_002889 | XLOC_066485 | -- | intronic_lncRNA | 2.365167 | 4.126146 | 10.199398 | 4.086792 |
| LNC_001283 | XLOC_027834 | -- | intronic_lncRNA | 1.03805 | 1.216899 | 0.829689 | 1.428492 |
| LNC_001282 | XLOC_027699 | -- | intronic_lncRNA | 2.350804 | 1.672829 | 1.035352 | 2.969748 |
| LNC_001281 | XLOC_027699 | -- | intronic_lncRNA | 3.903072 | 1.215303 | 8.29676 | 1.533755 |
| LNC_001280 | XLOC_027699 | -- | intronic_lncRNA | 1.543427 | 4.68039 | 1.12998 | 4.172935 |
| LNC_001287 | XLOC_028223 | -- | lincRNA | 0.918464 | 0.245497 | 0.080155 | 0.153179 |
| LNC_001286 | XLOC_028157 | -- | lincRNA | 1.235475 | 0.636471 | 0.187653 | 0.207123 |
| LNC_001285 | XLOC_027894 | -- | intronic_lncRNA | 0.274391 | 0.520014 | 0.281545 | 0.252262 |

| LNC_001284 | XLOC_027876 | -- | intronic_lncRNA | 0 | 0.538595 | 0 | 0 |
| --- | --- | --- | --- | --- | --- | --- | --- |
| LNC_001289 | XLOC_028263 | -- | intronic_lncRNA | 0.06315 | 0.094513 | 0.517893 | 0.894622 |
| LNC_001288 | XLOC_028260 | -- | intronic_lncRNA | 18.879953 | 11.911383 | 15.639662 | 13.372662 |
| LNC_004079 | XLOC_088278 | -- | intronic_lncRNA | 0.503929 | 0 | 0.223029 | 0.675729 |
| LNC_004078 | XLOC_088274 | -- | intronic_lncRNA | 0.40775 | 0 | 0.510557 | 0.095968 |
| LNC_004073 | XLOC_088129 | -- | intronic_lncRNA | 0.46907 | 0.185458 | 0.425079 | 0.624759 |
| LNC_004072 | XLOC_088128 | -- | intronic_lncRNA | 0.418889 | 0.313251 | 1.108764 | 0.606743 |
| LNC_004071 | XLOC_088128 | -- | intronic_lncRNA | 0.552754 | 0.096388 | 0 | 0.224739 |
| LNC_004070 | XLOC_088125 | -- | intronic_lncRNA | 0.401057 | 0.621421 | 0.401493 | 0.704448 |
| LNC_004077 | XLOC_088255 | -- | intronic_lncRNA | 0.561839 | 0.369304 | 0.792346 | 0.364864 |
| LNC_004076 | XLOC_088255 | -- | intronic_lncRNA | 0.107246 | 0.176385 | 0.411909 | 0.130817 |
| LNC_004075 | XLOC_088254 | -- | intronic_lncRNA | 0.276381 | 0.197449 | 0.49139 | 0.335911 |
| LNC_004074 | XLOC_088162 | -- | intronic_lncRNA | 0.086998 | 0.304107 | 0.396063 | 0.53068 |
| LNC_000958 | XLOC_020460 | -- | intronic_lncRNA | 0.004584 | 0.015804 | 0.008183 | 0.403375 |
| LNC_000959 | XLOC_020492 | -- | intronic_lncRNA | 0 | 0 | 0.451647 | 0 |
| LNC_005708 | XLOC_133728 | -- | lincRNA | 1.013019 | 0.130338 | 0 | 0 |
| LNC_005709 | XLOC_133748 | -- | antisense_lncRNA | 0.609587 | 0.578318 | 0.317562 | 0.634906 |
| LNC_000950 | XLOC_020305 | -- | intronic_lncRNA | 0.343879 | 0.761474 | 0.635993 | 0.484025 |
| LNC_000951 | XLOC_020307 | -- | intronic_lncRNA | 0.107382 | 0.115078 | 0.806465 | 0.237301 |
| LNC_000952 | XLOC_020311 | -- | intronic_lncRNA | 1.07942 | 0.113069 | 0.156255 | 0.133144 |
| LNC_000953 | XLOC_020311 | -- | intronic_lncRNA | 1.695071 | 0.421462 | 0.397017 | 0.344986 |
| LNC_000954 | XLOC_020426 | -- | intronic_lncRNA | 0.189966 | 0.377817 | 0.337702 | 0.593435 |
| LNC_000955 | XLOC_020449 | -- | intronic_lncRNA | 0.572706 | 0 | 0 | 0.159628 |
| LNC_000956 | XLOC_020460 | -- | intronic_lncRNA | 0.906855 | 0.651611 | 1.075321 | 0.76345 |
| LNC_000957 | XLOC_020460 | -- | intronic_lncRNA | 0.43874 | 0.222645 | 0.773756 | 0.266901 |
| LNC_005852 | XLOC_137819 | -- | intronic_lncRNA | 0 | 0 | 0.500875 | 0.329426 |
| LNC_005853 | XLOC_137825 | -- | intronic_lncRNA | 0.165506 | 0.242388 | 0.622944 | 0.16208 |
| LNC_005850 | XLOC_137723 | -- | intronic_lncRNA | 0.709325 | 0.388779 | 0.83649 | 0.669554 |
| LNC_005851 | XLOC_137788 | -- | intronic_lncRNA | 0.535528 | 0.684613 | 0.433119 | 0.299777 |
| LNC_005856 | XLOC_137829 | -- | intronic_lncRNA | 0.430696 | 0.19054 | 0.065786 | 0.306062 |
| LNC_005857 | XLOC_137840 | -- | intronic_lncRNA | 0.41753 | 0.156693 | 0.305856 | 0.41798 |
| LNC_005854 | XLOC_137826 | -- | intronic_lncRNA | 0.286151 | 0.202576 | 0.935682 | 0.36659 |
| LNC_005855 | XLOC_137829 | -- | intronic_lncRNA | 3.085117 | 1.586938 | 3.067025 | 3.84095 |
| LNC_005858 | XLOC_137842 | -- | intronic_lncRNA | 0.902589 | 0.751013 | 0.547152 | 0.571343 |
| LNC_005859 | XLOC_137846 | -- | intronic_lncRNA | 0.948266 | 0 | 0 | 0 |
| LNC_004851 | XLOC_109076 | -- | intronic_lncRNA | 0.548393 | 0 | 0 | 0 |
| LNC_004850 | XLOC_108875 | -- | intronic_lncRNA | 2.391791 | 0.19679 | 0.297435 | 0.34564 |
| LNC_004325 | XLOC_093910 | -- | intronic_lncRNA | 0.585602 | 0.14783 | 0.042701 | 0.153777 |
| LNC_001773 | XLOC_039300 | -- | lincRNA | 0.150189 | 0.250028 | 0.288409 | 0.779829 |
| LNC_001772 | XLOC_039036 | -- | intronic_lncRNA | 0.434287 | 0.504342 | 0.672234 | 0 |
| LNC_001771 | XLOC_038950 | -- | intronic_lncRNA | 0.422699 | 0.686704 | 0.552915 | 0.144589 |
| LNC_001770 | XLOC_038944 | -- | intronic_lncRNA | 1.474605 | 1.714863 | 1.237312 | 1.202846 |
| LNC_001777 | XLOC_039449 | -- | intronic_lncRNA | 2.729462 | 0.934559 | 1.192018 | 1.672554 |
| LNC_001776 | XLOC_039333 | -- | intronic_lncRNA | 1.13364 | 0.521014 | 0.841747 | 0.874314 |
| LNC_001775 | XLOC_039318 | -- | intronic_lncRNA | 0.436561 | 0.278956 | 0.571427 | 1.340717 |
| LNC_001774 | XLOC_039317 | -- | intronic_lncRNA | 0.494034 | 0.172078 | 0.306941 | 0.134591 |
| LNC_004323 | XLOC_093833 | -- | intronic_lncRNA | 0.755613 | 0.945454 | 0.859103 | 0.037141 |
| LNC_001779 | XLOC_039462 | -- | intronic_lncRNA | 0.537673 | 0.400823 | 0.814483 | 1.404429 |
| LNC_001778 | XLOC_039451 | -- | intronic_lncRNA | 0.666853 | 0.276273 | 0.607072 | 0.8287 |
| LNC_004854 | XLOC_109132 | -- | intronic_lncRNA | 0.144109 | 0.1424 | 0.284241 | 0.652827 |
| LNC_004321 | XLOC_093818 | -- | lincRNA | 0.162943 | 0.066348 | 0.672466 | 0.480945 |
| LNC_004320 | XLOC_093816 | -- | intronic_lncRNA | 0.013868 | 0.590206 | 1.329675 | 0.027231 |
| LNC_004815 | XLOC_107546 | -- | intronic_lncRNA | 0.463485 | 0.671308 | 0.464549 | 0.518715 |
| LNC_004814 | XLOC_107438 | -- | intronic_lncRNA | 0.266526 | 0.3839 | 0.610627 | 0.943257 |
| LNC_004817 | XLOC_107706 | -- | lincRNA | 0.125879 | 0 | 0 | 0.434909 |
| LNC_004816 | XLOC_107566 | -- | intronic_lncRNA | 0.325638 | 0.563856 | 0.413418 | 0.184184 |
| LNC_004811 | XLOC_107397 | -- | intronic_lncRNA | 0.512159 | 0.327084 | 0.484598 | 0.774635 |
| LNC_004810 | XLOC_107396 | -- | intronic_lncRNA | 0.240927 | 0.178121 | 0.424772 | 0.592596 |
| LNC_004813 | XLOC_107438 | -- | intronic_lncRNA | 0.066555 | 0.041577 | 0.265617 | 0.819751 |
| LNC_004812 | XLOC_107422 | -- | lincRNA | 0.498206 | 0.216528 | 0.326946 | 0.413772 |
| LNC_004819 | XLOC_107729 | -- | intronic_lncRNA | 0.228783 | 0.284159 | 0.601341 | 0.215175 |
| LNC_004818 | XLOC_107725 | -- | intronic_lncRNA | 0.56565 | 0.115515 | 0.295964 | 0.312962 |
| LNC_001360 | XLOC_029894 | -- | lincRNA | 1.34308 | 0.367263 | 0.022991 | 0.074573 |
| LNC_001361 | XLOC_029973 | -- | intronic_lncRNA | 0.508102 | 0 | 0 | 0 |
| LNC_001362 | XLOC_029984 | -- | intronic_lncRNA | 0.267288 | 0.441374 | 0.059608 | 0.496848 |
| LNC_001363 | XLOC_029985 | -- | intronic_lncRNA | 0.402284 | 0.680844 | 0.09545 | 0.246507 |
| LNC_001364 | XLOC_030044 | -- | intronic_lncRNA | 0.694126 | 0.30646 | 0.16627 | 0.124681 |
| LNC_001365 | XLOC_030056 | -- | intronic_lncRNA | 3.114336 | 2.679907 | 3.710102 | 3.85884 |
| LNC_001366 | XLOC_030067 | -- | intronic_lncRNA | 0.454461 | 0.167651 | 0.469066 | 0.447869 |
| LNC_001367 | XLOC_030069 | -- | intronic_lncRNA | 0.817464 | 0.424093 | 0.281446 | 0.143175 |
| LNC_001368 | XLOC_030100 | -- | intronic_lncRNA | 0.671527 | 0.173132 | 0.273959 | 0.055439 |
| LNC_001369 | XLOC_030101 | -- | intronic_lncRNA | 0.560656 | 0.584832 | 0.20204 | 0.388841 |
| LNC_003638 | XLOC_077827 | -- | intronic_lncRNA | 0.39056 | 0.777103 | 0.505632 | 0.219666 |
| LNC_003639 | XLOC_077828 | -- | intronic_lncRNA | 0.79906 | 0 | 0.053866 | 0.215427 |
| LNC_003630 | XLOC_077489 | -- | intronic_lncRNA | 1.69276 | 1.519521 | 2.683862 | 2.477897 |
| LNC_003631 | XLOC_077501 | -- | lincRNA | 0.905679 | 0.386899 | 0.887123 | 0.458265 |
| LNC_003632 | XLOC_077505 | -- | intronic_lncRNA | 0.606668 | 0.284396 | 0.964281 | 0.760227 |

| LNC_003633 | XLOC_077623 | -- | intronic_lncRNA | 1.645581 | 1.520248 | 1.85673 | 1.661471 |
| --- | --- | --- | --- | --- | --- | --- | --- |
| LNC_003634 | XLOC_077822 | -- | intronic_lncRNA | 0.428678 | 0.233252 | 0.507223 | 0.808584 |
| LNC_003635 | XLOC_077822 | -- | intronic_lncRNA | 0.342517 | 0.275032 | 0.331597 | 0.399185 |
| LNC_003636 | XLOC_077823 | -- | antisense_lncRNA | 0.457991 | 0 | 0.226949 | 0.294903 |
| LNC_003637 | XLOC_077826 | -- | intronic_lncRNA | 0.514921 | 0.617014 | 0.289823 | 0.968241 |
| LNC_005289 | XLOC_121195 | -- | intronic_lncRNA | 0.568969 | 0.234646 | 0.22349 | 0.873888 |
| LNC_005288 | XLOC_121134 | -- | intronic_lncRNA | 0.818757 | 0.619998 | 0.39896 | 0.610268 |
| LNC_005287 | XLOC_121114 | -- | intronic_lncRNA | 0 | 0.03151 | 0.188837 | 0.820628 |
| LNC_005286 | XLOC_121113 | -- | intronic_lncRNA | 0.584704 | 0.796155 | 0.92369 | 0.431086 |
| LNC_005285 | XLOC_121109 | -- | lincRNA | 1.938557 | 0.753645 | 1.110689 | 1.320176 |
| LNC_005284 | XLOC_121027 | -- | intronic_lncRNA | 0 | 0 | 0.664483 | 0.270365 |
| LNC_005283 | XLOC_121027 | -- | intronic_lncRNA | 0.124286 | 0 | 0.20534 | 0.762488 |
| LNC_005282 | XLOC_121019 | -- | intronic_lncRNA | 0.362176 | 0.223217 | 0.50165 | 0.469533 |
| LNC_005281 | XLOC_120949 | -- | lincRNA | 0.386672 | 0.142361 | 0.541923 | 0 |
| LNC_005280 | XLOC_120925 | -- | antisense_lncRNA | 0 | 0 | 0.414757 | 0.082124 |
| LNC_002949 | XLOC_067564 | -- | lincRNA | 0.641777 | 0.494468 | 0.19807 | 0.100599 |
| LNC_002948 | XLOC_067551 | -- | intronic_lncRNA | 1.542839 | 0.797371 | 1.500988 | 1.315705 |
| LNC_002941 | XLOC_067470 | -- | intronic_lncRNA | 0.789741 | 0.302299 | 0.483235 | 0.569251 |
| LNC_002940 | XLOC_067425 | -- | intronic_lncRNA | 1.404891 | 0.224913 | 0.724325 | 0.78213 |
| LNC_002943 | XLOC_067543 | -- | intronic_lncRNA | 0.505152 | 0.403062 | 0.867969 | 0.661124 |
| LNC_002942 | XLOC_067541 | -- | lincRNA | 0 | 0 | 0.104764 | 0.517976 |
| LNC_002945 | XLOC_067546 | -- | intronic_lncRNA | 1.193488 | 0.157725 | 0.022424 | 0.444884 |
| LNC_002944 | XLOC_067543 | -- | intronic_lncRNA | 0 | 0.184316 | 0.469955 | 0.806639 |
| LNC_002947 | XLOC_067548 | -- | intronic_lncRNA | 1.020265 | 0.567337 | 0.406883 | 0.347544 |
| LNC_002946 | XLOC_067547 | -- | intronic_lncRNA | 0 | 0.527267 | 0.133797 | 0 |
| LNC_001948 | XLOC_043955 | -- | intronic_lncRNA | 0 | 0.075988 | 0.192696 | 0.845395 |
| LNC_001949 | XLOC_043956 | -- | intronic_lncRNA | 0.216632 | 0.116894 | 0.540316 | 0.638913 |
| LNC_001944 | XLOC_043708 | -- | intronic_lncRNA | 0.796191 | 0 | 0.166565 | 0.078654 |
| LNC_001945 | XLOC_043712 | -- | intronic_lncRNA | 0.178882 | 0.256033 | 0.599335 | 0.447698 |
| LNC_001946 | XLOC_043716 | -- | intronic_lncRNA | 1.617207 | 1.691573 | 0.964985 | 1.212978 |
| LNC_001947 | XLOC_043812 | -- | lincRNA | 0.713409 | 0.900433 | 0.67051 | 0.291741 |
| LNC_001940 | XLOC_043667 | -- | intronic_lncRNA | 1.116219 | 1.005019 | 0.496606 | 0.929354 |
| LNC_001941 | XLOC_043668 | -- | lincRNA | 0 | 0 | 0.413844 | 0 |
| LNC_001942 | XLOC_043682 | -- | lincRNA | 0.649141 | 0 | 0.288325 | 0.463231 |
| LNC_001943 | XLOC_043708 | -- | intronic_lncRNA | 0 | 0.35311 | 0 | 0.302859 |
| LNC_000011 | XLOC_000222 | -- | lincRNA | 0.374397 | 1.371086 | 0.181576 | 0.744208 |
| LNC_000010 | XLOC_000222 | -- | lincRNA | 0.804314 | 0.101971 | 0 | 0.237263 |
| LNC_000013 | XLOC_000258 | -- | lincRNA | 0.418952 | 1.137344 | 0.169266 | 0.326627 |
| LNC_000012 | XLOC_000249 | -- | lincRNA | 0 | 0 | 0.836798 | 0 |
| LNC_000015 | XLOC_000288 | -- | lincRNA | 15.765784 | 6.24247 | 5.41766 | 3.178347 |
| LNC_000014 | XLOC_000288 | -- | lincRNA | 42.69495 | 31.521236 | 28.592916 | 18.093128 |
| LNC_000017 | XLOC_000289 | -- | lincRNA | 65.15226 | 0.122756 | 0.104774 | 0.058762 |
| LNC_000016 | XLOC_000289 | -- | lincRNA | 22.540487 | 0 | 0 | 0 |
| LNC_000019 | XLOC_000289 | -- | lincRNA | 6.067104 | 14.217477 | 3.877717 | 2.445741 |
| LNC_000018 | XLOC_000289 | -- | lincRNA | 19.261473 | 0.245645 | 36.601227 | 18.010824 |
| LNC_000239 | XLOC_003335 | -- | intronic_lncRNA | 1.238805 | 0.457821 | 0.575972 | 0.562013 |
| LNC_000238 | XLOC_003261 | -- | antisense_lncRNA | 0.972527 | 0.488113 | 0.394085 | 0.329584 |
| LNC_000235 | XLOC_003256 | -- | intronic_lncRNA | 0.489856 | 0 | 0 | 0 |
| LNC_000234 | XLOC_003253 | -- | intronic_lncRNA | 0.130944 | 0.124119 | 0.348455 | 0.155765 |
| LNC_000237 | XLOC_003260 | -- | intronic_lncRNA | 1.191307 | 0.06561 | 0.076514 | 0 |
| LNC_000236 | XLOC_003259 | -- | intronic_lncRNA | 0.901314 | 0.057086 | 0 | 0 |
| LNC_000231 | XLOC_003211 | -- | intronic_lncRNA | 2.766363 | 1.00814 | 2.318878 | 2.712892 |
| LNC_000230 | XLOC_003202 | -- | intronic_lncRNA | 0.499915 | 0.217497 | 0.251263 | 0.588876 |
| LNC_004626 | XLOC_102945 | -- | intronic_lncRNA | 0.428244 | 0.503337 | 0.696577 | 0.796453 |
| LNC_004627 | XLOC_102956 | -- | lincRNA | 0.592086 | 0.361088 | 0 | 0 |
| LNC_004624 | XLOC_102840 | -- | intronic_lncRNA | 0.311857 | 0.366266 | 0.530271 | 0.542858 |
| LNC_004625 | XLOC_102867 | -- | intronic_lncRNA | 0 | 0.197277 | 0.153783 | 0.625111 |
| LNC_004622 | XLOC_102827 | -- | intronic_lncRNA | 0.214164 | 0.273531 | 0.735009 | 0.134283 |
| LNC_004623 | XLOC_102833 | -- | intronic_lncRNA | 0.104264 | 0 | 0.447345 | 0 |
| LNC_004620 | XLOC_102797 | -- | lincRNA | 0 | 0 | 0 | 0.50498 |
| LNC_004621 | XLOC_102815 | -- | intronic_lncRNA | 0.500928 | 0.411561 | 0.533468 | 0.799875 |
| LNC_000232 | XLOC_003246 | -- | intronic_lncRNA | 0 | 0.615103 | 0.498516 | 0.306811 |
| LNC_004628 | XLOC_102963 | -- | lincRNA | 0.6184 | 0 | 0 | 0 |
| LNC_004629 | XLOC_102991 | -- | intronic_lncRNA | 0.290228 | 0.128887 | 0.05219 | 0.417101 |
| LNC_001568 | XLOC_034095 | -- | intronic_lncRNA | 0.196103 | 0.267752 | 0.033183 | 0.543685 |
| LNC_001569 | XLOC_034096 | -- | intronic_lncRNA | 0.48401 | 0 | 0.578008 | 0.337222 |
| LNC_001566 | XLOC_034046 | -- | intronic_lncRNA | 0.567127 | 0 | 0 | 0 |
| LNC_001567 | XLOC_034095 | -- | intronic_lncRNA | 0.730488 | 0.552692 | 0.254239 | 0.195674 |
| LNC_001564 | XLOC_034039 | -- | intronic_lncRNA | 1.028321 | 0.134459 | 1.278252 | 0.572447 |
| LNC_001565 | XLOC_034046 | -- | intronic_lncRNA | 0.463214 | 1.093688 | 1.231955 | 0.853618 |
| LNC_001562 | XLOC_034025 | -- | intronic_lncRNA | 0.570671 | 0.88984 | 0.57182 | 0.258411 |
| LNC_001563 | XLOC_034031 | -- | intronic_lncRNA | 0.484414 | 0 | 0.097857 | 0.267429 |
| LNC_001560 | XLOC_034021 | -- | intronic_lncRNA | 3.821437 | 0.833348 | 0.844859 | 0.271412 |
| LNC_001561 | XLOC_034025 | -- | intronic_lncRNA | 0.707224 | 0.073551 | 0.007508 | 0.015254 |
| LNC_000805 | XLOC_017053 | -- | intronic_lncRNA | 0.355598 | 0.529657 | 0.423072 | 0.397711 |
| LNC_003847 | XLOC_082459 | -- | lincRNA | 0.262033 | 0.27856 | 0.589542 | 0.582722 |
| LNC_003846 | XLOC_082459 | -- | lincRNA | 0.189446 | 0.544792 | 0.296577 | 0.935255 |

| LNC_003845 | XLOC_082459 | -- | lincRNA | 0.264846 | 0 | 0.585836 | 0.641372 |
| --- | --- | --- | --- | --- | --- | --- | --- |
| LNC_003844 | XLOC_082459 | -- | lincRNA | 0.418463 | 1.006016 | 1.130222 | 1.895584 |
| LNC_003843 | XLOC_082442 | -- | intronic_lncRNA | 0.816458 | 0.894619 | 0.963255 | 0.93238 |
| LNC_003842 | XLOC_082410 | -- | intronic_lncRNA | 0.78363 | 0.175576 | 0.312077 | 0 |
| LNC_003841 | XLOC_082398 | -- | intronic_lncRNA | 0.991514 | 0.330941 | 0.746953 | 0.352942 |
| LNC_003840 | XLOC_082392 | -- | intronic_lncRNA | 0.374939 | 0.532301 | 0.428925 | 0.733722 |
| LNC_003849 | XLOC_082487 | -- | intronic_lncRNA | 0.309192 | 0.33984 | 0.291736 | 0.312171 |
| LNC_003848 | XLOC_082479 | -- | intronic_lncRNA | 0.952618 | 0.485339 | 0.062769 | 0.098218 |
| LNC_000381 | XLOC_006526 | -- | intronic_lncRNA | 1.860377 | 3.679679 | 4.32803 | 3.248023 |
| LNC_000380 | XLOC_006475 | -- | lincRNA | 1.186575 | 1.666538 | 0.405368 | 0.457597 |
| LNC_000383 | XLOC_006545 | -- | intronic_lncRNA | 0.587375 | 0.316153 | 0.736804 | 0 |
| LNC_000382 | XLOC_006526 | -- | intronic_lncRNA | 1.278531 | 0.762086 | 0.950399 | 1.635536 |
| LNC_000385 | XLOC_006583 | -- | intronic_lncRNA | 1.997555 | 1.221501 | 1.917233 | 2.379007 |
| LNC_000384 | XLOC_006574 | -- | intronic_lncRNA | 0.878031 | 0.189788 | 0.673261 | 0.610603 |
| LNC_000387 | XLOC_006598 | -- | lincRNA | 0 | 0 | 0.535308 | 0 |
| LNC_000386 | XLOC_006598 | -- | lincRNA | 0.042897 | 0.633969 | 0.041568 | 0.697464 |
| LNC_000389 | XLOC_006631 | -- | intronic_lncRNA | 0.6827 | 0.142676 | 0.480999 | 0.598413 |
| LNC_000388 | XLOC_006630 | -- | intronic_lncRNA | 0.807236 | 0.215707 | 0.350092 | 0.684758 |
| LNC_002714 | XLOC_063039 | -- | intronic_lncRNA | 0.07765 | 0.128199 | 0.497116 | 0.110237 |
| LNC_002715 | XLOC_063108 | -- | intronic_lncRNA | 0.043466 | 0.536321 | 0.121142 | 0.002695 |
| LNC_002716 | XLOC_063111 | -- | intronic_lncRNA | 0 | 0.046015 | 0 | 0.654498 |
| LNC_002717 | XLOC_063111 | -- | intronic_lncRNA | 0.119118 | 0.654262 | 0.254687 | 0.855958 |
| LNC_002710 | XLOC_062925 | -- | intronic_lncRNA | 0.179602 | 0.914339 | 0.173125 | 0.29215 |
| LNC_002711 | XLOC_062929 | -- | intronic_lncRNA | 0.25053 | 0.484905 | 0 | 0 |
| LNC_002712 | XLOC_063005 | -- | intronic_lncRNA | 1.357268 | 0.881874 | 1.469461 | 2.384751 |
| LNC_002713 | XLOC_063020 | -- | intronic_lncRNA | 0.712085 | 0 | 0 | 0 |
| LNC_002718 | XLOC_063115 | -- | intronic_lncRNA | 0.189993 | 0.723024 | 0 | 0.433059 |
| LNC_002719 | XLOC_063129 | -- | intronic_lncRNA | 0 | 0.319849 | 0.517202 | 0.213431 |
| LNC_004019 | XLOC_086758 | -- | intronic_lncRNA | 0.64526 | 0 | 1.13663 | 0.275443 |
| LNC_004018 | XLOC_086755 | -- | intronic_lncRNA | 0.176999 | 0 | 0 | 0.485826 |
| LNC_004015 | XLOC_086656 | -- | lincRNA | 0 | 0 | 0.19559 | 0.444447 |
| LNC_004014 | XLOC_086599 | -- | intronic_lncRNA | 0.404228 | 0.863186 | 0.881802 | 1.127024 |
| LNC_004017 | XLOC_086744 | -- | lincRNA | 0.197333 | 0.133485 | 0.127925 | 0.486205 |
| LNC_004016 | XLOC_086710 | -- | intronic_lncRNA | 0.775381 | 1.522733 | 1.220219 | 1.740436 |
| LNC_004011 | XLOC_086507 | -- | intronic_lncRNA | 0.514338 | 0.963047 | 0 | 0.07182 |
| LNC_004010 | XLOC_086458 | -- | lincRNA | 0.644952 | 0.618087 | 0.718255 | 0.709782 |
| LNC_004013 | XLOC_086554 | -- | intronic_lncRNA | 0.927813 | 0.159266 | 0.931543 | 0.494886 |
| LNC_004012 | XLOC_086521 | -- | intronic_lncRNA | 0.131932 | 0.405293 | 0.333919 | 0.222574 |
| LNC_005498 | XLOC_127041 | -- | intronic_lncRNA | 0 | 0 | 0 | 0.410159 |
| LNC_005499 | XLOC_127045 | -- | intronic_lncRNA | 0.587838 | 0.276278 | 0.222223 | 0.301038 |
| LNC_005492 | XLOC_126862 | -- | intronic_lncRNA | 0.729933 | 0.645883 | 0.273163 | 0.424977 |
| LNC_005493 | XLOC_126905 | -- | intronic_lncRNA | 0 | 0 | 0.399434 | 0 |
| LNC_005490 | XLOC_126860 | -- | intronic_lncRNA | 0.530128 | 0.223936 | 0.453391 | 0.199457 |
| LNC_005491 | XLOC_126861 | -- | intronic_lncRNA | 1.167667 | 0.164101 | 0.106601 | 0.622589 |
| LNC_005496 | XLOC_126936 | -- | intronic_lncRNA | 0.970469 | 0.616877 | 2.32253 | 2.004618 |
| LNC_005497 | XLOC_126937 | -- | antisense_lncRNA | 0.626856 | 0.292461 | 0.161006 | 0 |
| LNC_005494 | XLOC_126906 | -- | intronic_lncRNA | 0.290648 | 0 | 0 | 0.237212 |
| LNC_005495 | XLOC_126931 | -- | intronic_lncRNA | 0.664852 | 0.018227 | 0.217796 | 0 |
| LNC_005063 | XLOC_115441 | -- | intronic_lncRNA | 0.287028 | 0.367011 | 0.73282 | 1.097127 |
| LNC_005062 | XLOC_115441 | -- | intronic_lncRNA | 0.985902 | 0.010963 | 0.3084 | 0 |
| LNC_005061 | XLOC_115440 | -- | intronic_lncRNA | 6.242408 | 15.03978 | 17.404057 | 19.786448 |
| LNC_002152 | XLOC_048954 | -- | intronic_lncRNA | 1.214089 | 0.274297 | 0.528956 | 0.235226 |
| LNC_002153 | XLOC_048955 | -- | intronic_lncRNA | 0.328977 | 0.372844 | 1.064218 | 0.540996 |
| LNC_002150 | XLOC_048952 | -- | intronic_lncRNA | 0.488033 | 0.369321 | 0.799348 | 0.447438 |
| LNC_002151 | XLOC_048953 | -- | intronic_lncRNA | 0.482004 | 0.566207 | 0.275492 | 0.233815 |
| LNC_002156 | XLOC_048956 | -- | intronic_lncRNA | 0.709886 | 0.373632 | 0.568506 | 0.611918 |
| LNC_002157 | XLOC_048956 | -- | intronic_lncRNA | 1.124878 | 0.094925 | 0.319324 | 0.216356 |
| LNC_002154 | XLOC_048955 | -- | intronic_lncRNA | 0.888617 | 0.167471 | 0.095953 | 0.075231 |
| LNC_002155 | XLOC_048956 | -- | intronic_lncRNA | 0.2824 | 0.313109 | 0.324212 | 0.098161 |
| LNC_005067 | XLOC_115475 | -- | intronic_lncRNA | 0.605499 | 0.313027 | 0.735639 | 0.657164 |
| LNC_002158 | XLOC_048956 | -- | intronic_lncRNA | 0.734884 | 0.206897 | 0.141637 | 0.307298 |
| LNC_002159 | XLOC_048957 | -- | intronic_lncRNA | 0.881673 | 0.485814 | 0.679813 | 0.605749 |
| LNC_005066 | XLOC_115471 | -- | lincRNA | 0.592321 | 0 | 0 | 0.080971 |
| LNC_003542 | XLOC_075274 | -- | lincRNA | 0.65819 | 1.06634 | 0 | 0 |
| LNC_003543 | XLOC_075274 | -- | lincRNA | 0.355831 | 1.720137 | 1.344877 | 1.599225 |
| LNC_003540 | XLOC_075272 | -- | lincRNA | 4.030209 | 1.300751 | 0.739333 | 0.590763 |
| LNC_003541 | XLOC_075274 | -- | lincRNA | 1.521519 | 0.761003 | 0.893251 | 1.705796 |
| LNC_003546 | XLOC_075277 | -- | lincRNA | 5.236462 | 1.005335 | 0.506844 | 0.587331 |
| LNC_003547 | XLOC_075278 | -- | lincRNA | 2.331595 | 1.122117 | 0.765472 | 0.720005 |
| LNC_003544 | XLOC_075274 | -- | lincRNA | 0.727874 | 1.040148 | 0.052692 | 1.002586 |
| LNC_003545 | XLOC_075274 | -- | lincRNA | 1.401139 | 1.302714 | 0.376782 | 0.092225 |
| LNC_003548 | XLOC_075279 | -- | lincRNA | 9.13775 | 2.164484 | 0.954525 | 1.273497 |
| LNC_000544 | XLOC_010019 | -- | intronic_lncRNA | 0.061562 | 0.568621 | 0.544416 | 0.37987 |
| LNC_005728 | XLOC_134378 | -- | intronic_lncRNA | 0.628841 | 0.436216 | 0.347124 | 0.3201 |
| LNC_005729 | XLOC_134413 | -- | intronic_lncRNA | 0.206575 | 0.136782 | 0.251676 | 0 |
| LNC_005724 | XLOC_134303 | -- | lincRNA | 0 | 0 | 0 | 0.589186 |
| LNC_005725 | XLOC_134336 | -- | lincRNA | 0.592072 | 1.009901 | 0.205592 | 0.75188 |

| LNC_005726 | XLOC_134356 | -- | intronic_lncRNA | 0.258888 | 0.239997 | 0.67941 | 0.582127 |
| --- | --- | --- | --- | --- | --- | --- | --- |
| LNC_005727 | XLOC_134364 | -- | intronic_lncRNA | 0 | 0 | 0 | 0.426938 |
| LNC_005720 | XLOC_134182 | -- | intronic_lncRNA | 0 | 0.143348 | 0.137343 | 0.457731 |
| LNC_005069 | XLOC_115643 | -- | lincRNA | 0 | 0 | 0.163095 | 0.969159 |
| LNC_005722 | XLOC_134271 | -- | lincRNA | 0 | 0 | 0.428309 | 0 |
| LNC_005723 | XLOC_134287 | -- | intronic_lncRNA | 2.800907 | 1.499539 | 2.966404 | 3.182731 |
| LNC_005874 | XLOC_138241 | -- | intronic_lncRNA | 0.306591 | 0.078329 | 0.194227 | 0.396314 |
| LNC_005875 | XLOC_138258 | -- | intronic_lncRNA | 0.614457 | 0.123274 | 0.411393 | 0.252151 |
| LNC_005876 | XLOC_138335 | -- | intronic_lncRNA | 0 | 0 | 0.221809 | 0.446523 |
| LNC_005068 | XLOC_115592 | -- | lincRNA | 0 | 0 | 0 | 0.421194 |
| LNC_005870 | XLOC_138125 | -- | intronic_lncRNA | 0.699864 | 0.754186 | 0.701521 | 0.707499 |
| LNC_005871 | XLOC_138136 | -- | intronic_lncRNA | 1.019279 | 0.590403 | 0.585627 | 0.64183 |
| LNC_005872 | XLOC_138206 | -- | intronic_lncRNA | 0.649376 | 0 | 0.954942 | 0.095901 |
| LNC_005873 | XLOC_138235 | -- | intronic_lncRNA | 0.750305 | 0.368612 | 0.36334 | 0.816375 |
| LNC_005878 | XLOC_138435 | -- | lincRNA | 0.542068 | 0.467047 | 0.358928 | 0.04802 |
| LNC_005879 | XLOC_138498 | -- | intronic_lncRNA | 0.070611 | 0 | 0.691025 | 0.167877 |
| LNC_006343 | XLOC_152210 | -- | intronic_lncRNA | 5.585359 | 0.371058 | 0.452983 | 0.499331 |
| LNC_003094 | XLOC_070920 | -- | intronic_lncRNA | 0.177916 | 0.243285 | 0.218925 | 0.416833 |
| LNC_003095 | XLOC_070925 | -- | intronic_lncRNA | 0 | 0 | 0 | 0.593219 |
| LNC_003096 | XLOC_070928 | -- | intronic_lncRNA | 0 | 0.279546 | 0.979617 | 0.900563 |
| LNC_003097 | XLOC_070936 | -- | intronic_lncRNA | 16.165836 | 9.567388 | 5.467627 | 6.388909 |
| LNC_003090 | XLOC_070737 | -- | intronic_lncRNA | 0.569809 | 0.762165 | 0.613514 | 0.419567 |
| LNC_003091 | XLOC_070745 | -- | lincRNA | 0.54736 | 0 | 0.092227 | 0 |
| LNC_003092 | XLOC_070759 | -- | intronic_lncRNA | 1.162029 | 0.163339 | 0.321122 | 0.151209 |
| LNC_003093 | XLOC_070891 | -- | antisense_lncRNA | 0.34616 | 0.580825 | 0.268664 | 0.056201 |
| LNC_003098 | XLOC_070938 | -- | intronic_lncRNA | 0.556877 | 0 | 0.316574 | 0.514665 |
| LNC_003099 | XLOC_071316 | -- | lincRNA | 0 | 0.212515 | 0.64838 | 0 |
| LNC_006342 | XLOC_152177 | -- | intronic_lncRNA | 2.225625 | 0.51975 | 0.971949 | 2.757082 |
| LNC_000419 | XLOC_007389 | -- | intronic_lncRNA | 0 | 1.166437 | 1.4818 | 0.251287 |
| LNC_006383 | XLOC_153167 | -- | intronic_lncRNA | 0.194989 | 0.882538 | 0.188186 | 0.41883 |
| LNC_006382 | XLOC_153166 | -- | intronic_lncRNA | 0.410649 | 0.915496 | 0 | 0.535932 |
| LNC_006381 | XLOC_153165 | -- | intronic_lncRNA | 0.091121 | 0.442582 | 0.051845 | 0.343153 |
| LNC_006380 | XLOC_153102 | -- | intronic_lncRNA | 0.329124 | 0.059933 | 0.334391 | 0.399634 |
| LNC_006387 | XLOC_153230 | -- | intronic_lncRNA | 0.292 | 0.635514 | 0.722957 | 0.576506 |
| LNC_006386 | XLOC_153230 | -- | intronic_lncRNA | 0.644928 | 0.727752 | 0.65005 | 1.16999 |
| LNC_006385 | XLOC_153217 | -- | intronic_lncRNA | 0 | 0 | 0.092321 | 0.918682 |
| LNC_006384 | XLOC_153212 | -- | intronic_lncRNA | 0.17582 | 0.087771 | 0.822282 | 0.52573 |
| LNC_006389 | XLOC_153254 | -- | intronic_lncRNA | 0.484997 | 0.382293 | 0.751599 | 0.644454 |
| LNC_006388 | XLOC_153246 | -- | intronic_lncRNA | 0.945197 | 0.095952 | 0.178847 | 0.131726 |
| LNC_000418 | XLOC_007389 | -- | intronic_lncRNA | 0 | 0 | 0 | 1.026298 |
| LNC_001715 | XLOC_037660 | -- | antisense_lncRNA | 0 | 0.283985 | 0.582153 | 0.27225 |
| LNC_001714 | XLOC_037649 | -- | intronic_lncRNA | 0.144889 | 0.193138 | 0.602088 | 0.498194 |
| LNC_001717 | XLOC_037694 | -- | intronic_lncRNA | 0.346382 | 0.157411 | 0.184635 | 0.491046 |
| LNC_001716 | XLOC_037673 | -- | intronic_lncRNA | 0.420256 | 0.476441 | 0.188044 | 0.288968 |
| LNC_001711 | XLOC_037554 | -- | intronic_lncRNA | 0 | 0 | 0.172688 | 0.508768 |
| LNC_001710 | XLOC_037553 | -- | intronic_lncRNA | 0 | 0.240194 | 0.146753 | 0.618426 |
| LNC_001713 | XLOC_037593 | -- | intronic_lncRNA | 0.274514 | 0.13772 | 0.422305 | 0.199236 |
| LNC_001712 | XLOC_037558 | -- | intronic_lncRNA | 0 | 0.818172 | 0.248768 | 0.58888 |
| LNC_001719 | XLOC_037824 | -- | lincRNA | 0 | 0.248362 | 0.846329 | 0.105773 |
| LNC_001718 | XLOC_037702 | -- | lincRNA | 0.580127 | 0 | 0 | 0 |
| LNC_004389 | XLOC_095595 | -- | intronic_lncRNA | 0.238795 | 0.407992 | 0.660465 | 1.035358 |
| LNC_004388 | XLOC_095589 | -- | intronic_lncRNA | 0.153112 | 0 | 0.69433 | 0.296452 |
| LNC_004839 | XLOC_108785 | -- | lincRNA | 0.112955 | 0.158218 | 0.306889 | 0.707338 |
| LNC_004838 | XLOC_108785 | -- | lincRNA | 0 | 0.059535 | 0.308971 | 0.580412 |
| LNC_004837 | XLOC_108667 | -- | intronic_lncRNA | 1.022645 | 1.254303 | 0.65562 | 0.817858 |
| LNC_004384 | XLOC_095524 | -- | intronic_lncRNA | 0.427591 | 0.287093 | 0.531804 | 0.700731 |
| LNC_004387 | XLOC_095587 | -- | intronic_lncRNA | 1.115496 | 0.128438 | 0.487585 | 0.348861 |
| LNC_004386 | XLOC_095587 | -- | intronic_lncRNA | 0.214022 | 0.932656 | 0.28042 | 0.350454 |
| LNC_004833 | XLOC_108563 | -- | lincRNA | 4.550197 | 3.906734 | 1.126216 | 1.352944 |
| LNC_004380 | XLOC_095454 | -- | intronic_lncRNA | 0.460762 | 0.350524 | 0.539073 | 0.835487 |
| LNC_004383 | XLOC_095507 | -- | intronic_lncRNA | 0 | 0 | 0 | 0.39136 |
| LNC_004382 | XLOC_095465 | -- | intronic_lncRNA | 0 | 0 | 0.113138 | 0.506069 |
| LNC_005838 | XLOC_137486 | -- | intronic_lncRNA | 0.529537 | 0 | 0.408228 | 0.160833 |
| LNC_005839 | XLOC_137487 | -- | intronic_lncRNA | 0.859149 | 0 | 0 | 0.201693 |
| LNC_000448 | XLOC_007920 | -- | intronic_lncRNA | 0 | 0 | 0.488855 | 1.232231 |
| LNC_005129 | XLOC_117157 | -- | intronic_lncRNA | 1.625708 | 0.787065 | 1.242733 | 2.382055 |
| LNC_000442 | XLOC_007883 | -- | intronic_lncRNA | 0 | 0.078166 | 0 | 0.809724 |
| LNC_000443 | XLOC_007884 | -- | intronic_lncRNA | 0.285484 | 0.136306 | 0.469359 | 0.510034 |
| LNC_000440 | XLOC_007877 | -- | intronic_lncRNA | 0.076891 | 0.356742 | 0.618828 | 0.949203 |
| LNC_000441 | XLOC_007881 | -- | intronic_lncRNA | 0.395931 | 0.2116 | 0.328547 | 0.593083 |
| LNC_000446 | XLOC_007906 | -- | intronic_lncRNA | 0.252007 | 0.098901 | 0.204465 | 0.345663 |
| LNC_000447 | XLOC_007916 | -- | intronic_lncRNA | 0 | 0 | 0.298366 | 0.34093 |
| LNC_000444 | XLOC_007884 | -- | intronic_lncRNA | 0.558968 | 0.137762 | 0 | 0 |
| LNC_000445 | XLOC_007885 | -- | intronic_lncRNA | 0.661719 | 0.223903 | 0.451636 | 0.505608 |
| LNC_006349 | XLOC_152255 | -- | intronic_lncRNA | 0.992269 | 0.18256 | 0.108531 | 0.375559 |
| LNC_006264 | XLOC_150358 | -- | intronic_lncRNA | 0.633898 | 0.468737 | 0.44027 | 0.55706 |
| LNC_005261 | XLOC_120668 | -- | intronic_lncRNA | 0.686517 | 0.382587 | 0.628851 | 0.437936 |

| LNC_005260 | XLOC_120664 | -- | intronic_lncRNA | 0.146588 | 0.099524 | 0.485234 | 0.18758 |
| --- | --- | --- | --- | --- | --- | --- | --- |
| LNC_005263 | XLOC_120708 | -- | lincRNA | 0 | 0 | 0 | 0.577416 |
| LNC_005262 | XLOC_120703 | -- | intronic_lncRNA | 0 | 0 | 0.515359 | 0 |
| LNC_005265 | XLOC_120713 | -- | lincRNA | 0.049683 | 0 | 0.399457 | 0 |
| LNC_005264 | XLOC_120712 | -- | lincRNA | 0.858017 | 0 | 0.138057 | 0.14676 |
| LNC_005267 | XLOC_120728 | -- | intronic_lncRNA | 0.85765 | 0.500759 | 0.38501 | 0.172867 |
| LNC_005266 | XLOC_120724 | -- | intronic_lncRNA | 0.281884 | 0.641585 | 0.935269 | 0.652853 |
| LNC_005269 | XLOC_120773 | -- | intronic_lncRNA | 0.710345 | 0.437255 | 0.55511 | 0.43579 |
| LNC_005268 | XLOC_120749 | -- | intronic_lncRNA | 0.34968 | 0 | 0.430037 | 0 |
| LNC_006265 | XLOC_150361 | -- | intronic_lncRNA | 1.054417 | 0.430158 | 0.902558 | 0.830145 |
| LNC_002963 | XLOC_067878 | -- | intronic_lncRNA | 0.538814 | 0 | 0.066918 | 0.141305 |
| LNC_002962 | XLOC_067841 | -- | intronic_lncRNA | 0.170412 | 0.223837 | 0.181003 | 0.426666 |
| LNC_002961 | XLOC_067833 | -- | intronic_lncRNA | 3.808508 | 3.339122 | 3.042038 | 2.332814 |
| LNC_002960 | XLOC_067810 | -- | lincRNA | 0.258171 | 0.10861 | 0 | 0.409865 |
| LNC_002967 | XLOC_068036 | -- | intronic_lncRNA | 1.530444 | 0.262869 | 1.017044 | 1.313612 |
| LNC_002966 | XLOC_068031 | -- | intronic_lncRNA | 0.585861 | 0 | 0.460923 | 1.144984 |
| LNC_002965 | XLOC_068006 | -- | intronic_lncRNA | 0.971781 | 0.419179 | 0.442742 | 0.2516 |
| LNC_002964 | XLOC_067879 | -- | intronic_lncRNA | 0 | 0.653706 | 0 | 0 |
| LNC_002969 | XLOC_068037 | -- | intronic_lncRNA | 1.346445 | 0.241851 | 0.518144 | 0.848438 |
| LNC_002968 | XLOC_068036 | -- | intronic_lncRNA | 0 | 0.640397 | 0 | 0 |
| LNC_001926 | XLOC_043393 | -- | intronic_lncRNA | 0 | 0 | 0 | 3.612532 |
| LNC_001927 | XLOC_043393 | -- | intronic_lncRNA | 4.08849 | 4.644789 | 3.557986 | 1.312166 |
| LNC_001924 | XLOC_043388 | -- | intronic_lncRNA | 1.719734 | 0.806332 | 0.875779 | 0.819628 |
| LNC_001925 | XLOC_043392 | -- | intronic_lncRNA | 0.996575 | 0.904853 | 0.533353 | 0.592889 |
| LNC_001922 | XLOC_043354 | -- | lincRNA | 0.323738 | 0.397145 | 0.056785 | 0.359298 |
| LNC_001923 | XLOC_043381 | -- | antisense_lncRNA | 0.952772 | 0 | 0 | 0.026176 |
| LNC_001920 | XLOC_043266 | -- | intronic_lncRNA | 0.294554 | 0.104621 | 0.755216 | 0.208145 |
| LNC_001921 | XLOC_043267 | -- | intronic_lncRNA | 0.368492 | 0.091917 | 0.515369 | 0.589955 |
| LNC_001928 | XLOC_043398 | -- | intronic_lncRNA | 0.906343 | 0.07539 | 0.387806 | 0.277114 |
| LNC_001929 | XLOC_043408 | -- | intronic_lncRNA | 0.678179 | 1.14668 | 0.380334 | 0 |
| LNC_000039 | XLOC_000393 | -- | lincRNA | 3.139384 | 0 | 0 | 0 |
| LNC_000038 | XLOC_000393 | -- | lincRNA | 3.920228 | 4.036863 | 2.217334 | 3.231253 |
| LNC_000033 | XLOC_000360 | -- | lincRNA | 1.394051 | 0.751923 | 0.618744 | 0.823608 |
| LNC_000032 | XLOC_000360 | -- | lincRNA | 1.167855 | 1.041176 | 0.421499 | 0.339698 |
| LNC_000031 | XLOC_000346 | -- | lincRNA | 0.577252 | 1.139047 | 0.580302 | 0.423892 |
| LNC_000030 | XLOC_000324 | -- | lincRNA | 0.143637 | 0.587496 | 0.642642 | 0.369163 |
| LNC_000037 | XLOC_000392 | -- | lincRNA | 0.622523 | 2.449317 | 0.055522 | 0.108804 |
| LNC_000036 | XLOC_000390 | -- | lincRNA | 0.507856 | 0 | 0.643174 | 0 |
| LNC_000035 | XLOC_000390 | -- | lincRNA | 4.500123 | 3.010082 | 0.483438 | 1.008014 |
| LNC_000034 | XLOC_000388 | -- | lincRNA | 0.712895 | 0.352024 | 0.097857 | 0 |
| LNC_004608 | XLOC_102302 | -- | intronic_lncRNA | 1.061558 | 0.482407 | 0.837654 | 0.836764 |
| LNC_004609 | XLOC_102345 | -- | lincRNA | 0.886536 | 0.480111 | 0.185088 | 0.375459 |
| LNC_004600 | XLOC_101714 | -- | intronic_lncRNA | 1.121135 | 0.392772 | 1.425749 | 0.477053 |
| LNC_004601 | XLOC_101734 | -- | lincRNA | 0.130852 | 0.228859 | 0.52408 | 0.1132 |
| LNC_004602 | XLOC_101747 | -- | intronic_lncRNA | 0 | 0.703152 | 0.105154 | 0.252786 |
| LNC_004603 | XLOC_101786 | -- | intronic_lncRNA | 0.369893 | 0.144503 | 0.390424 | 0.570583 |
| LNC_004604 | XLOC_101845 | -- | lincRNA | 0 | 0.186355 | 0.262962 | 0.419891 |
| LNC_004605 | XLOC_101882 | -- | intronic_lncRNA | 7.037882 | 12.851228 | 6.600646 | 7.794996 |
| LNC_004606 | XLOC_101887 | -- | intronic_lncRNA | 0.587408 | 0 | 0.148034 | 0.018984 |
| LNC_004607 | XLOC_102254 | -- | antisense_lncRNA | 0 | 0 | 0.461099 | 0.075992 |
| LNC_001540 | XLOC_033728 | -- | intronic_lncRNA | 2.041496 | 1.932451 | 2.108917 | 1.777057 |
| LNC_001541 | XLOC_033754 | -- | intronic_lncRNA | 0.148139 | 0.147807 | 1.528521 | 1.027509 |
| LNC_001542 | XLOC_033825 | -- | intronic_lncRNA | 0.633072 | 0.110624 | 0.698336 | 0.040773 |
| LNC_001543 | XLOC_033874 | -- | intronic_lncRNA | 0.388339 | 0.533817 | 0.94076 | 0.710674 |
| LNC_001544 | XLOC_033874 | -- | intronic_lncRNA | 0.241234 | 0.208053 | 0.553959 | 0.56333 |
| LNC_001545 | XLOC_033880 | -- | intronic_lncRNA | 0.21594 | 0.819682 | 0.224831 | 0.070807 |
| LNC_001546 | XLOC_033881 | -- | intronic_lncRNA | 0.881702 | 0.679654 | 0.716379 | 1.057562 |
| LNC_001547 | XLOC_033882 | -- | intronic_lncRNA | 0.689445 | 0.481781 | 0.421164 | 0.102553 |
| LNC_001548 | XLOC_033883 | -- | intronic_lncRNA | 0.719306 | 0.521135 | 0.493992 | 0.805929 |
| LNC_001549 | XLOC_033883 | -- | intronic_lncRNA | 0 | 0.611958 | 0.166781 | 0.001036 |
| LNC_005470 | XLOC_126193 | -- | lincRNA | 0.065123 | 0 | 0.464536 | 0.637483 |
| LNC_005471 | XLOC_126219 | -- | intronic_lncRNA | 1.356598 | 0.095906 | 1.947582 | 1.566443 |
| LNC_005472 | XLOC_126222 | -- | intronic_lncRNA | 0 | 0.923891 | 0.321079 | 0.264754 |
| LNC_005473 | XLOC_126224 | -- | intronic_lncRNA | 0 | 0.270603 | 0.292201 | 0.58146 |
| LNC_005474 | XLOC_126229 | -- | intronic_lncRNA | 0.51877 | 0.443711 | 0.775206 | 0.652694 |
| LNC_005475 | XLOC_126244 | -- | intronic_lncRNA | 0.125917 | 0.230412 | 0.731838 | 0.620575 |
| LNC_005476 | XLOC_126253 | -- | intronic_lncRNA | 0 | 0 | 0.477872 | 0.825373 |
| LNC_005477 | XLOC_126253 | -- | intronic_lncRNA | 0.853219 | 0 | 0.223776 | 0.22076 |
| LNC_005478 | XLOC_126275 | -- | intronic_lncRNA | 0.140768 | 0.072628 | 0.57492 | 0.474408 |
| LNC_005479 | XLOC_126282 | -- | intronic_lncRNA | 2.717515 | 3.929048 | 3.577498 | 3.69312 |
| LNC_002369 | XLOC_054840 | -- | intronic_lncRNA | 0.330884 | 0 | 0.69393 | 0.176711 |
| LNC_002368 | XLOC_054800 | -- | intronic_lncRNA | 0.400156 | 0 | 0.877451 | 0 |
| LNC_005747 | XLOC_134809 | -- | intronic_lncRNA | 0.355798 | 0.060521 | 0.600983 | 0.668942 |
| LNC_002361 | XLOC_054618 | -- | intronic_lncRNA | 0.311995 | 0.252313 | 0.557286 | 0.572579 |
| LNC_002360 | XLOC_054615 | -- | intronic_lncRNA | 0.777669 | 0.677099 | 0.642712 | 0.45189 |
| LNC_002363 | XLOC_054645 | -- | intronic_lncRNA | 0.003884 | 0.756766 | 0.041567 | 0.003993 |
| LNC_002362 | XLOC_054619 | -- | intronic_lncRNA | 0.428338 | 0.813902 | 0.402867 | 0.339864 |

| LNC_002365 | XLOC_054709 | -- | lincRNA | 0.083481 | 0 | 0.466554 | 0.234546 |
| --- | --- | --- | --- | --- | --- | --- | --- |
| LNC_002364 | XLOC_054659 | -- | intronic_lncRNA | 0.444303 | 0.147276 | 0.284018 | 0.587657 |
| LNC_002367 | XLOC_054800 | -- | intronic_lncRNA | 0.48133 | 0.255343 | 0.372428 | 0.706958 |
| LNC_002366 | XLOC_054787 | -- | intronic_lncRNA | 0.664247 | 1.474363 | 1.035204 | 0.98827 |
| LNC_003379 | XLOC_075175 | -- | lincRNA | 1.451076 | 0.049146 | 0.060481 | 0.128595 |
| LNC_003378 | XLOC_075175 | -- | lincRNA | 0 | 1.187243 | 0.299608 | 0 |
| LNC_003371 | XLOC_075175 | -- | lincRNA | 0.084614 | 0.045798 | 0.058278 | 3.894406 |
| LNC_003370 | XLOC_075175 | -- | lincRNA | 0.077905 | 0.453219 | 0 | 0 |
| LNC_003373 | XLOC_075175 | -- | lincRNA | 4.40238 | 0.805388 | 0.919776 | 3.263227 |
| LNC_003372 | XLOC_075175 | -- | lincRNA | 1.473485 | 1.424481 | 0.391884 | 0.787598 |
| LNC_003375 | XLOC_075175 | -- | lincRNA | 2.14275 | 1.072843 | 0.803091 | 0.914059 |
| LNC_003374 | XLOC_075175 | -- | lincRNA | 2.763669 | 1.907655 | 0.542274 | 0.319836 |
| LNC_003377 | XLOC_075175 | -- | lincRNA | 5.447235 | 2.511343 | 1.283544 | 1.79321 |
| LNC_003376 | XLOC_075175 | -- | lincRNA | 0.83351 | 1.454505 | 0.288854 | 1.392366 |
| LNC_005744 | XLOC_134675 | -- | intronic_lncRNA | 2.662052 | 2.811301 | 3.108702 | 3.913618 |
| LNC_000631 | XLOC_011901 | -- | intronic_lncRNA | 0.318184 | 0.426466 | 0.023444 | 0.743207 |
| LNC_000630 | XLOC_011901 | -- | intronic_lncRNA | 0.282301 | 0.192191 | 0.432936 | 0.553197 |
| LNC_000633 | XLOC_011916 | -- | intronic_lncRNA | 2.033354 | 1.117577 | 1.21153 | 1.978828 |
| LNC_000632 | XLOC_011906 | -- | intronic_lncRNA | 1.57057 | 0 | 1.069511 | 0 |
| LNC_000635 | XLOC_012143 | -- | intronic_lncRNA | 0 | 0 | 1.513564 | 1.146011 |
| LNC_000634 | XLOC_011990 | -- | intronic_lncRNA | 0.379106 | 0.32404 | 0 | 0.408376 |
| LNC_000637 | XLOC_012190 | -- | intronic_lncRNA | 0.629108 | 0.403401 | 1.092269 | 0.354087 |
| LNC_000636 | XLOC_012143 | -- | intronic_lncRNA | 1.289972 | 0.950348 | 0.081467 | 0.102605 |
| LNC_000639 | XLOC_012232 | -- | intronic_lncRNA | 0 | 0.117473 | 0.430039 | 0 |
| LNC_000638 | XLOC_012194 | -- | intronic_lncRNA | 0.55298 | 0.426013 | 0.909748 | 0.755881 |
| LNC_002778 | XLOC_064464 | -- | intronic_lncRNA | 0 | 0 | 0.447345 | 0 |
| LNC_002779 | XLOC_064486 | -- | intronic_lncRNA | 1.116955 | 1.251578 | 0.953755 | 2.04392 |
| LNC_002776 | XLOC_064340 | -- | antisense_lncRNA | 0 | 0 | 0 | 0.646086 |
| LNC_002777 | XLOC_064421 | -- | lincRNA | 0.084452 | 0.099827 | 0.062277 | 0.685452 |
| LNC_002774 | XLOC_064233 | -- | intronic_lncRNA | 0.424408 | 0.143138 | 0.298481 | 0.719742 |
| LNC_002775 | XLOC_064322 | -- | intronic_lncRNA | 0.035098 | 0.103467 | 0.56253 | 0 |
| LNC_002772 | XLOC_064136 | -- | antisense_lncRNA | 0.111256 | 0 | 0.593989 | 0.123015 |
| LNC_002773 | XLOC_064141 | -- | intronic_lncRNA | 0 | 0 | 0.468308 | 0 |
| LNC_002770 | XLOC_064112 | -- | intronic_lncRNA | 0.507682 | 0 | 0 | 0 |
| LNC_002771 | XLOC_064134 | -- | intronic_lncRNA | 0 | 0 | 0.192994 | 0.425648 |
| LNC_004037 | XLOC_087202 | -- | intronic_lncRNA | 0.350719 | 0.462945 | 1.023431 | 0.744156 |
| LNC_004036 | XLOC_087175 | -- | intronic_lncRNA | 0 | 0.465225 | 0 | 0.209317 |
| LNC_004035 | XLOC_087174 | -- | intronic_lncRNA | 0.461308 | 0.48535 | 0.484441 | 0.193879 |
| LNC_004034 | XLOC_087151 | -- | intronic_lncRNA | 0.264859 | 0 | 0 | 0.506797 |
| LNC_004033 | XLOC_087149 | -- | intronic_lncRNA | 0.48644 | 0.235395 | 0 | 0 |
| LNC_004032 | XLOC_087141 | -- | intronic_lncRNA | 1.238187 | 0.826704 | 0.641028 | 0.362428 |
| LNC_004031 | XLOC_087117 | -- | intronic_lncRNA | 0.5468 | 0 | 0 | 0 |
| LNC_004030 | XLOC_087112 | -- | intronic_lncRNA | 0.522407 | 0.09477 | 0.66338 | 0.191843 |
| LNC_004039 | XLOC_087224 | -- | intronic_lncRNA | 0.174892 | 0.945319 | 0.382259 | 0.160775 |
| LNC_004038 | XLOC_087208 | -- | intronic_lncRNA | 0.978376 | 1.567292 | 0.723377 | 0.937013 |
| LNC_006431 | XLOC_154052 | -- | intronic_lncRNA | 1.969747 | 1.05057 | 1.305455 | 0.959075 |
| LNC_000198 | XLOC_002531 | -- | intronic_lncRNA | 0.532577 | 0.615461 | 0.375847 | 0.366679 |
| LNC_000199 | XLOC_002532 | -- | intronic_lncRNA | 0.561543 | 0.373523 | 0.369091 | 0.447214 |
| LNC_000194 | XLOC_002498 | -- | intronic_lncRNA | 0.676977 | 0.382244 | 0.624248 | 0.796553 |
| LNC_000195 | XLOC_002498 | -- | intronic_lncRNA | 0.307504 | 0 | 0.129546 | 0 |
| LNC_000196 | XLOC_002500 | -- | intronic_lncRNA | 0.17765 | 0 | 0.393401 | 0 |
| LNC_000197 | XLOC_002505 | -- | intronic_lncRNA | 1.645701 | 1.136202 | 2.097335 | 1.937953 |
| LNC_000190 | XLOC_002460 | -- | intronic_lncRNA | 0.033562 | 0.288752 | 0.499948 | 0.469632 |
| LNC_000191 | XLOC_002460 | -- | intronic_lncRNA | 0.841779 | 0 | 0.257447 | 0 |
| LNC_000192 | XLOC_002484 | -- | intronic_lncRNA | 1.017852 | 0.62821 | 0.753427 | 1.007916 |
| LNC_000193 | XLOC_002486 | -- | intronic_lncRNA | 1.31252 | 0.511759 | 1.370881 | 0.232337 |
| LNC_002174 | XLOC_049529 | -- | intronic_lncRNA | 0.402261 | 0.627973 | 0.965962 | 0.865897 |
| LNC_002175 | XLOC_049556 | -- | intronic_lncRNA | 0.500898 | 1.162911 | 1.009663 | 1.151173 |
| LNC_002176 | XLOC_049556 | -- | intronic_lncRNA | 1.121102 | 0 | 0.021016 | 0.063038 |
| LNC_002177 | XLOC_049602 | -- | intronic_lncRNA | 0.534933 | 0 | 0 | 0.151007 |
| LNC_002170 | XLOC_049481 | -- | intronic_lncRNA | 0.326983 | 0.578716 | 0.278798 | 0.039647 |
| LNC_002171 | XLOC_049481 | -- | intronic_lncRNA | 0.979764 | 0 | 0.475122 | 0.229659 |
| LNC_002172 | XLOC_049481 | -- | intronic_lncRNA | 0 | 0.320784 | 0.329517 | 0.431532 |
| LNC_002173 | XLOC_049489 | -- | lincRNA | 0.151316 | 0.060796 | 0.341371 | 0.378803 |
| LNC_002178 | XLOC_049611 | -- | intronic_lncRNA | 2.08258 | 1.476447 | 2.268537 | 3.178701 |
| LNC_002179 | XLOC_049614 | -- | intronic_lncRNA | 0.823195 | 0.706213 | 0.819255 | 0.385377 |
| LNC_003564 | XLOC_075432 | -- | intronic_lncRNA | 7.788976 | 8.690892 | 3.573555 | 4.61759 |
| LNC_003565 | XLOC_075463 | -- | intronic_lncRNA | 1.023262 | 1.528022 | 0.836652 | 0.686805 |
| LNC_003566 | XLOC_075474 | -- | intronic_lncRNA | 0.603662 | 0.456144 | 0.021094 | 0.404912 |
| LNC_003567 | XLOC_075520 | -- | lincRNA | 0 | 0.226714 | 0.214703 | 0.078578 |
| LNC_003560 | XLOC_075421 | -- | antisense_lncRNA | 0.293132 | 0.938977 | 0.720713 | 0.612654 |
| LNC_003561 | XLOC_075422 | -- | antisense_lncRNA | 0.892978 | 1.317068 | 0 | 0 |
| LNC_003562 | XLOC_075423 | -- | intronic_lncRNA | 0.007074 | 0.688632 | 0.06124 | 0.244919 |
| LNC_003563 | XLOC_075427 | -- | lincRNA | 2.880721 | 2.663575 | 1.073493 | 2.146672 |
| LNC_003568 | XLOC_075520 | -- | intronic_lncRNA | 0.562363 | 0.356369 | 0.293157 | 0.364213 |
| LNC_003569 | XLOC_075550 | -- | lincRNA | 0.201054 | 0.285123 | 0.70106 | 0.250383 |
| LNC_000994 | XLOC_021184 | -- | intronic_lncRNA | 1.068924 | 0.429779 | 1.237861 | 1.14466 |

| LNC_000995 | XLOC_021185 | -- | intronic_lncRNA | 0 | 0 | 0 | 0.519504 |
| --- | --- | --- | --- | --- | --- | --- | --- |
| LNC_000996 | XLOC_021223 | -- | intronic_lncRNA | 0.590942 | 0.756255 | 0.633104 | 0.880703 |
| LNC_000997 | XLOC_021226 | -- | intronic_lncRNA | 3.122406 | 1.435912 | 1.067552 | 2.655433 |
| LNC_000990 | XLOC_021094 | -- | intronic_lncRNA | 0.520612 | 0.74244 | 0.727328 | 0.62607 |
| LNC_000991 | XLOC_021102 | -- | intronic_lncRNA | 0.831292 | 0.331109 | 0.643565 | 0.932561 |
| LNC_000992 | XLOC_021118 | -- | intronic_lncRNA | 0 | 0.008214 | 0.149208 | 0.718071 |
| LNC_000993 | XLOC_021138 | -- | intronic_lncRNA | 0.637469 | 0 | 0 | 0.426938 |
| LNC_000998 | XLOC_021226 | -- | intronic_lncRNA | 0.199005 | 0.062703 | 0.837978 | 0.299606 |
| LNC_000999 | XLOC_021228 | -- | intronic_lncRNA | 1.412648 | 0.812418 | 0.898638 | 0.694581 |
| LNC_005748 | XLOC_134826 | -- | intronic_lncRNA | 1.033262 | 0.986708 | 0.750316 | 1.593017 |
| LNC_005749 | XLOC_134830 | -- | intronic_lncRNA | 0 | 0.411656 | 0.535794 | 0 |
| LNC_002288 | XLOC_052656 | -- | antisense_lncRNA | 0 | 0.483879 | 0.215099 | 0 |
| LNC_002289 | XLOC_052661 | -- | intronic_lncRNA | 0.281455 | 0.231599 | 0.557948 | 0 |
| LNC_002286 | XLOC_052637 | -- | lincRNA | 2.326003 | 0.487814 | 0 | 0 |
| LNC_002287 | XLOC_052656 | -- | intronic_lncRNA | 0 | 0.692475 | 0.923906 | 0.146303 |
| LNC_002284 | XLOC_052611 | -- | intronic_lncRNA | 0.249302 | 0 | 0.414452 | 0.36146 |
| LNC_002285 | XLOC_052611 | -- | intronic_lncRNA | 0.322345 | 0.317335 | 0.713005 | 0.156602 |
| LNC_002282 | XLOC_052610 | -- | intronic_lncRNA | 0.427367 | 0.109663 | 0.425811 | 0.109885 |
| LNC_002283 | XLOC_052610 | -- | intronic_lncRNA | 0.056001 | 0.359252 | 0.244053 | 0.846004 |
| LNC_002280 | XLOC_052448 | -- | intronic_lncRNA | 0 | 0.088726 | 0.465258 | 0 |
| LNC_002281 | XLOC_052537 | -- | intronic_lncRNA | 0.252551 | 0.24686 | 0 | 0.412455 |
| LNC_000406 | XLOC_007096 | -- | intronic_lncRNA | 0.363655 | 0.159887 | 0.593912 | 0.44463 |
| LNC_000407 | XLOC_007097 | -- | intronic_lncRNA | 0 | 0 | 0 | 0.561162 |
| LNC_000404 | XLOC_007092 | -- | intronic_lncRNA | 0.490765 | 1.03474 | 0.73232 | 0.490546 |
| LNC_005165 | XLOC_118017 | -- | intronic_lncRNA | 0.650694 | 0.729323 | 0.712282 | 0.579197 |
| LNC_000402 | XLOC_007037 | -- | intronic_lncRNA | 0.813966 | 0.793748 | 0.170284 | 0.111337 |
| LNC_000403 | XLOC_007060 | -- | intronic_lncRNA | 0.888941 | 0.609064 | 0.592616 | 1.008778 |
| LNC_001485 | XLOC_032367 | -- | intronic_lncRNA | 1.212316 | 0.8745 | 1.32498 | 1.025244 |
| LNC_001484 | XLOC_032359 | -- | intronic_lncRNA | 0.6167 | 1.061889 | 1.039554 | 0.752764 |
| LNC_001487 | XLOC_032370 | -- | intronic_lncRNA | 1.30548 | 0.519699 | 0.841766 | 0.328049 |
| LNC_001486 | XLOC_032369 | -- | intronic_lncRNA | 0.894138 | 0.425414 | 0.637779 | 0.344351 |
| LNC_001481 | XLOC_032340 | -- | intronic_lncRNA | 1.898545 | 0.399257 | 1.907049 | 1.504329 |
| LNC_001480 | XLOC_032340 | -- | intronic_lncRNA | 5.291741 | 2.97492 | 8.435323 | 7.836393 |
| LNC_001483 | XLOC_032358 | -- | intronic_lncRNA | 0.663511 | 0.497668 | 0.8099 | 1.170397 |
| LNC_001482 | XLOC_032357 | -- | intronic_lncRNA | 1.301255 | 0.945475 | 2.399294 | 2.485363 |
| LNC_000401 | XLOC_007037 | -- | intronic_lncRNA | 0.062196 | 0.05185 | 1.025015 | 0.99574 |
| LNC_001489 | XLOC_032426 | -- | intronic_lncRNA | 0.439161 | 0.465468 | 0.3579 | 0.594393 |
| LNC_001488 | XLOC_032371 | -- | intronic_lncRNA | 0.707193 | 0.499769 | 0.634061 | 0.711867 |
| LNC_006201 | XLOC_148634 | -- | intronic_lncRNA | 0.625284 | 0.269806 | 0.159859 | 0.479582 |
| LNC_005818 | XLOC_137244 | -- | intronic_lncRNA | 0.310854 | 0 | 0 | 0.936752 |
| LNC_005819 | XLOC_137248 | -- | intronic_lncRNA | 0.884844 | 0 | 0.353798 | 0.204976 |
| LNC_005816 | XLOC_137165 | -- | intronic_lncRNA | 0.505196 | 0.756753 | 0.733362 | 1.012254 |
| LNC_005817 | XLOC_137244 | -- | intronic_lncRNA | 0.09567 | 0.38457 | 0.764461 | 0.631176 |
| LNC_005814 | XLOC_137164 | -- | intronic_lncRNA | 0.42855 | 0.527427 | 0.492846 | 0.737585 |
| LNC_005815 | XLOC_137164 | -- | intronic_lncRNA | 0.165596 | 0.050768 | 0.262552 | 0.199278 |
| LNC_005812 | XLOC_137044 | -- | intronic_lncRNA | 0.490414 | 0.276223 | 0.603093 | 0.527531 |
| LNC_005813 | XLOC_137164 | -- | intronic_lncRNA | 0.214069 | 0.272273 | 0.317276 | 0.316411 |
| LNC_005810 | XLOC_137019 | -- | intronic_lncRNA | 0.098208 | 0.152009 | 0.652537 | 0.112508 |
| LNC_005811 | XLOC_137019 | -- | intronic_lncRNA | 0.251695 | 0.484941 | 0.265904 | 0.349833 |
| LNC_006204 | XLOC_148702 | -- | intronic_lncRNA | 0.216003 | 0.077987 | 0.654401 | 0.310129 |
| LNC_006205 | XLOC_148703 | -- | intronic_lncRNA | 0.364892 | 0.323514 | 0.472073 | 0.232063 |
| LNC_006109 | XLOC_146038 | -- | intronic_lncRNA | 0.781667 | 0 | 0.97747 | 0.54661 |
| LNC_006108 | XLOC_146037 | -- | intronic_lncRNA | 0.48986 | 0.444625 | 0.572056 | 0.469045 |
| LNC_000408 | XLOC_007097 | -- | intronic_lncRNA | 0.240114 | 0.702436 | 0 | 0 |
| LNC_001739 | XLOC_038419 | -- | intronic_lncRNA | 0.26216 | 0.208793 | 0.301769 | 0.705907 |
| LNC_001738 | XLOC_038418 | -- | intronic_lncRNA | 0.372545 | 0 | 0.610898 | 0.06364 |
| LNC_001737 | XLOC_038398 | -- | intronic_lncRNA | 0.735076 | 0.281011 | 0.223742 | 0.741792 |
| LNC_001736 | XLOC_038381 | -- | intronic_lncRNA | 0.167631 | 0.166883 | 0.451914 | 0.041624 |
| LNC_001735 | XLOC_038379 | -- | intronic_lncRNA | 1.065942 | 0.159191 | 1.34673 | 2.064328 |
| LNC_001734 | XLOC_038353 | -- | intronic_lncRNA | 0.733188 | 0.219471 | 0.559683 | 0.508158 |
| LNC_001733 | XLOC_038202 | -- | intronic_lncRNA | 0.555001 | 0.133975 | 0.499847 | 0.410181 |
| LNC_001732 | XLOC_038179 | -- | intronic_lncRNA | 0.465037 | 0.53621 | 0.72391 | 0.414381 |
| LNC_001731 | XLOC_038178 | -- | intronic_lncRNA | 0 | 0 | 0.441045 | 0 |
| LNC_001730 | XLOC_038165 | -- | intronic_lncRNA | 0.227118 | 0.112337 | 0.225084 | 0.880233 |
| LNC_006209 | XLOC_148798 | -- | intronic_lncRNA | 0.349062 | 0.217593 | 0.532483 | 0.300062 |
| LNC_006365 | XLOC_152518 | -- | intronic_lncRNA | 19.285839 | 17.649385 | 18.682121 | 17.720991 |
| LNC_006364 | XLOC_152492 | -- | intronic_lncRNA | 1.218286 | 2.052513 | 1.019679 | 1.139371 |
| LNC_000468 | XLOC_008288 | -- | intronic_lncRNA | 0 | 0 | 0.60706 | 0 |
| LNC_005109 | XLOC_116898 | -- | intronic_lncRNA | 0.914119 | 0.320938 | 1.614652 | 1.304996 |
| LNC_005104 | XLOC_116782 | -- | intronic_lncRNA | 3.944352 | 2.904713 | 5.465421 | 5.498012 |
| LNC_005105 | XLOC_116825 | -- | intronic_lncRNA | 0 | 0 | 1.302982 | 0.027578 |
| LNC_000466 | XLOC_008271 | -- | intronic_lncRNA | 0 | 0.188218 | 0.465572 | 0.070898 |
| LNC_000467 | XLOC_008273 | -- | intronic_lncRNA | 0.556264 | 0.370012 | 0.299657 | 0.361832 |
| LNC_000460 | XLOC_008157 | -- | intronic_lncRNA | 0.755029 | 0.504823 | 0.558639 | 0.640302 |
| LNC_000461 | XLOC_008215 | -- | lincRNA | 0.305756 | 0 | 1.232414 | 0.086931 |
| LNC_005102 | XLOC_116782 | -- | intronic_lncRNA | 1.495473 | 0.019083 | 0.641023 | 0.641177 |
| LNC_000463 | XLOC_008233 | -- | intronic_lncRNA | 0 | 0.289747 | 0.491485 | 0.075007 |

| LNC_006366 | XLOC_152552 | -- | intronic_lncRNA | 0.427864 | 0.1518 | 0.754631 | 0.20607 |
| --- | --- | --- | --- | --- | --- | --- | --- |
| LNC_005249 | XLOC_120305 | -- | intronic_lncRNA | 0.841312 | 0.445632 | 0.725875 | 0.429452 |
| LNC_005248 | XLOC_120303 | -- | intronic_lncRNA | 0.705851 | 0.454322 | 0.051711 | 0.030836 |
| LNC_005243 | XLOC_120009 | -- | lincRNA | 0.298648 | 0.160881 | 0.743602 | 0.478411 |
| LNC_005242 | XLOC_119908 | -- | intronic_lncRNA | 0.733615 | 0.370534 | 0.745931 | 0.806243 |
| LNC_005241 | XLOC_119889 | -- | intronic_lncRNA | 1.175496 | 0.621384 | 1.172979 | 0.982711 |
| LNC_005240 | XLOC_119887 | -- | intronic_lncRNA | 0.387272 | 0.728423 | 0.929466 | 0.378833 |
| LNC_005247 | XLOC_120302 | -- | intronic_lncRNA | 0.942257 | 0.463444 | 0 | 0 |
| LNC_005246 | XLOC_120301 | -- | intronic_lncRNA | 1.549014 | 0.332057 | 0.076935 | 0 |
| LNC_005245 | XLOC_120140 | -- | intronic_lncRNA | 0.576705 | 0.730565 | 0.466992 | 0.394946 |
| LNC_005244 | XLOC_120056 | -- | lincRNA | 0 | 0.589714 | 0 | 0 |
| LNC_006361 | XLOC_152449 | -- | intronic_lncRNA | 0 | 0 | 0.07293 | 0.675737 |
| LNC_002905 | XLOC_066952 | -- | intronic_lncRNA | 0.713607 | 0 | 1.071588 | 0.167655 |
| LNC_002904 | XLOC_066952 | -- | intronic_lncRNA | 0.447356 | 0.620663 | 1.037383 | 0.461453 |
| LNC_002907 | XLOC_067164 | -- | intronic_lncRNA | 0 | 0 | 0.553753 | 0.269881 |
| LNC_002906 | XLOC_066953 | -- | intronic_lncRNA | 0 | 0 | 0.164891 | 0.472657 |
| LNC_002901 | XLOC_066891 | -- | intronic_lncRNA | 1.148801 | 1.192409 | 0.803336 | 3.681943 |
| LNC_002900 | XLOC_066843 | -- | intronic_lncRNA | 1.416033 | 0.86677 | 0.736933 | 0.810993 |
| LNC_002903 | XLOC_066920 | -- | intronic_lncRNA | 0.618884 | 0.119157 | 0.252172 | 0.424024 |
| LNC_002902 | XLOC_066918 | -- | intronic_lncRNA | 0.686968 | 0.592145 | 0.522084 | 0.74168 |
| LNC_005958 | XLOC_141104 | -- | intronic_lncRNA | 0.128175 | 0.139379 | 0.644895 | 0.021404 |
| LNC_002909 | XLOC_067173 | -- | intronic_lncRNA | 0 | 0.291387 | 0.043822 | 0.479707 |
| LNC_002908 | XLOC_067165 | -- | intronic_lncRNA | 0.469924 | 0 | 0.224066 | 0.616076 |
| LNC_006360 | XLOC_152437 | -- | intronic_lncRNA | 0.186178 | 0.72965 | 0.101997 | 0 |
| LNC_000059 | XLOC_000611 | -- | lincRNA | 24.461981 | 13.02667 | 10.88481 | 10.26546 |
| LNC_000058 | XLOC_000611 | -- | lincRNA | 45.848473 | 2.857996 | 0 | 0 |
| LNC_000055 | XLOC_000565 | -- | lincRNA | 1.441636 | 2.122064 | 0.518014 | 1.56464 |
| LNC_000054 | XLOC_000533 | -- | lincRNA | 0.96624 | 0 | 0 | 0.530583 |
| LNC_000057 | XLOC_000611 | -- | lincRNA | 38.758774 | 19.546591 | 11.636852 | 3.220022 |
| LNC_000056 | XLOC_000586 | -- | lincRNA | 0 | 0 | 0 | 0.486195 |
| LNC_000051 | XLOC_000450 | -- | lincRNA | 1.3944 | 0.915561 | 0.483869 | 1.148433 |
| LNC_000050 | XLOC_000432 | -- | lincRNA | 0.239451 | 0.592611 | 0.481837 | 0.202953 |
| LNC_000053 | XLOC_000497 | -- | lincRNA | 0 | 0.5057 | 0.660469 | 0.077133 |
| LNC_000052 | XLOC_000496 | -- | lincRNA | 0.320896 | 0 | 0.200877 | 0.480525 |
| LNC_006363 | XLOC_152479 | -- | intronic_lncRNA | 0.309811 | 0.209567 | 0.408987 | 0.363251 |
| LNC_006362 | XLOC_152449 | -- | intronic_lncRNA | 0.483268 | 0.520981 | 0.772008 | 0.052473 |
| LNC_004198 | XLOC_090517 | -- | intronic_lncRNA | 0 | 0.280363 | 0 | 0 |
| LNC_004199 | XLOC_090520 | -- | intronic_lncRNA | 0 | 0 | 0 | 0.577416 |
| LNC_004190 | XLOC_090370 | -- | intronic_lncRNA | 0.10868 | 0 | 0.843624 | 0.035814 |
| LNC_004191 | XLOC_090419 | -- | intronic_lncRNA | 0.471612 | 0.095246 | 0.344303 | 0.688742 |
| LNC_004192 | XLOC_090420 | -- | intronic_lncRNA | 0.162736 | 0.557686 | 0.123587 | 0.150523 |
| LNC_004193 | XLOC_090421 | -- | intronic_lncRNA | 0.373824 | 0.215939 | 0.450582 | 0.604028 |
| LNC_004194 | XLOC_090428 | -- | intronic_lncRNA | 0.143068 | 0.063044 | 0.248152 | 0.430436 |
| LNC_004195 | XLOC_090429 | -- | intronic_lncRNA | 0.094229 | 0.123824 | 1.073667 | 0.140101 |
| LNC_004196 | XLOC_090434 | -- | intronic_lncRNA | 0.486868 | 0.095797 | 0.470266 | 0.63088 |
| LNC_004197 | XLOC_090517 | -- | intronic_lncRNA | 0.863132 | 0.102022 | 0.332457 | 0.025804 |
| LNC_001908 | XLOC_043074 | -- | lincRNA | 0.413357 | 0 | 0.758584 | 0.607052 |
| LNC_001909 | XLOC_043080 | -- | antisense_lncRNA | 0.496834 | 0 | 0.144346 | 0 |
| LNC_001900 | XLOC_042925 | -- | intronic_lncRNA | 0.696504 | 0.674332 | 0.361565 | 1.05863 |
| LNC_001901 | XLOC_042941 | -- | intronic_lncRNA | 1.336356 | 0.201638 | 6.707844 | 12.189168 |
| LNC_001902 | XLOC_042985 | -- | intronic_lncRNA | 0.662976 | 1.538945 | 0.704023 | 0.164145 |
| LNC_001903 | XLOC_043013 | -- | intronic_lncRNA | 0.830423 | 0 | 0 | 0 |
| LNC_001904 | XLOC_043056 | -- | intronic_lncRNA | 1.300716 | 0.635122 | 1.287703 | 1.571074 |
| LNC_001905 | XLOC_043060 | -- | intronic_lncRNA | 0 | 0.517851 | 0.257917 | 0.097141 |
| LNC_001906 | XLOC_043066 | -- | intronic_lncRNA | 0 | 0.127065 | 0.474168 | 0.44545 |
| LNC_001907 | XLOC_043067 | -- | intronic_lncRNA | 0.941077 | 0.21229 | 0.119613 | 0.175454 |
| LNC_006249 | XLOC_150066 | -- | lincRNA | 0 | 0.532104 | 0 | 0 |
| LNC_006434 | XLOC_154101 | -- | intronic_lncRNA | 1.062273 | 1.038291 | 1.257276 | 1.814721 |
| LNC_006244 | XLOC_149865 | -- | intronic_lncRNA | 0.309079 | 0.290696 | 0.286608 | 1.186421 |
| LNC_006242 | XLOC_149666 | -- | intronic_lncRNA | 0.464017 | 0 | 0.015638 | 0.023533 |
| LNC_002343 | XLOC_054095 | -- | intronic_lncRNA | 0.679759 | 1.314096 | 1.878424 | 1.672018 |
| LNC_002342 | XLOC_054062 | -- | intronic_lncRNA | 0 | 0.51555 | 0 | 0 |
| LNC_002341 | XLOC_054009 | -- | intronic_lncRNA | 0 | 0 | 0.346834 | 0.198157 |
| LNC_002340 | XLOC_054008 | -- | intronic_lncRNA | 0 | 0 | 0.484917 | 0.541321 |
| LNC_002347 | XLOC_054148 | -- | intronic_lncRNA | 0.323126 | 0.395845 | 0.8567 | 0.611342 |
| LNC_002346 | XLOC_054147 | -- | intronic_lncRNA | 0.282397 | 0.29726 | 0.404292 | 0.473122 |
| LNC_002345 | XLOC_054133 | -- | intronic_lncRNA | 0 | 0 | 0 | 0.632501 |
| LNC_002344 | XLOC_054095 | -- | intronic_lncRNA | 0 | 0.779189 | 0 | 0 |
| LNC_002349 | XLOC_054168 | -- | intronic_lncRNA | 0.664858 | 0.389693 | 0.411259 | 0.498786 |
| LNC_002348 | XLOC_054167 | -- | intronic_lncRNA | 1.74659 | 0.694152 | 0.670535 | 0.549456 |
| LNC_003353 | XLOC_075175 | -- | lincRNA | 0.412631 | 0 | 2.916961 | 0 |
| LNC_003352 | XLOC_075175 | -- | lincRNA | 2.765682 | 0.432997 | 0.457874 | 0.436037 |
| LNC_003351 | XLOC_075175 | -- | lincRNA | 2.855056 | 0.708301 | 0.267558 | 0.541683 |
| LNC_003350 | XLOC_075175 | -- | lincRNA | 9.709511 | 0 | 0 | 0 |
| LNC_003357 | XLOC_075175 | -- | lincRNA | 1.849475 | 1.657078 | 1.011555 | 0.926144 |
| LNC_003356 | XLOC_075175 | -- | lincRNA | 1.089044 | 0 | 0 | 0.008846 |
| LNC_003355 | XLOC_075175 | -- | lincRNA | 2.124769 | 0.43414 | 0.611989 | 0.570671 |

| LNC_003354 | XLOC_075175 | -- | lincRNA | 1.837908 | 1.350992 | 0.77093 | 1.043651 |
| --- | --- | --- | --- | --- | --- | --- | --- |
| LNC_003359 | XLOC_075175 | -- | lincRNA | 0.5457 | 0.294225 | 0 | 0.004635 |
| LNC_003358 | XLOC_075175 | -- | lincRNA | 3.743755 | 0.380844 | 0.841825 | 1.142401 |
| LNC_000619 | XLOC_011683 | -- | intronic_lncRNA | 2.156857 | 3.037247 | 2.436241 | 2.805326 |
| LNC_000618 | XLOC_011682 | -- | antisense_lncRNA | 0 | 0.301054 | 0.725455 | 0.846039 |
| LNC_000613 | XLOC_011643 | -- | intronic_lncRNA | 0.591006 | 0.583156 | 0.096056 | 0.187917 |
| LNC_000612 | XLOC_011638 | -- | intronic_lncRNA | 2.515532 | 3.897151 | 5.274634 | 7.045622 |
| LNC_000611 | XLOC_011637 | -- | intronic_lncRNA | 0.991143 | 0.36038 | 0.452602 | 0.417003 |
| LNC_000610 | XLOC_011637 | -- | intronic_lncRNA | 0.776098 | 3.425328 | 0.407168 | 0.376116 |
| LNC_000617 | XLOC_011682 | -- | intronic_lncRNA | 0.719802 | 0.351193 | 0.755948 | 0.444613 |
| LNC_000616 | XLOC_011679 | -- | intronic_lncRNA | 0.01197 | 0.042299 | 0.883889 | 0.051594 |
| LNC_000615 | XLOC_011679 | -- | intronic_lncRNA | 1.923184 | 2.237936 | 1.320648 | 2.398172 |
| LNC_000614 | XLOC_011649 | -- | lincRNA | 0.095608 | 0.667596 | 0.038628 | 0 |
| LNC_005597 | XLOC_130370 | -- | intronic_lncRNA | 0.248125 | 0.622202 | 0.161242 | 0.105624 |
| LNC_005596 | XLOC_130314 | -- | intronic_lncRNA | 0.495209 | 0.424097 | 0.602401 | 0.506962 |
| LNC_005595 | XLOC_130303 | -- | intronic_lncRNA | 0.122982 | 0.040902 | 0.46375 | 0.066239 |
| LNC_005594 | XLOC_130280 | -- | intronic_lncRNA | 0.267033 | 0.00899 | 0.474583 | 0.098324 |
| LNC_006437 | XLOC_154335 | -- | antisense_lncRNA | 0.764961 | 0.6395 | 0.167879 | 0.18657 |
| LNC_005593 | XLOC_130280 | -- | intronic_lncRNA | 1.255651 | 0.685702 | 0.537495 | 0.989874 |
| LNC_005592 | XLOC_130253 | -- | intronic_lncRNA | 0.631671 | 0.182507 | 0.276153 | 0.303969 |
| LNC_005591 | XLOC_130252 | -- | intronic_lncRNA | 0 | 0.111688 | 0.466602 | 0 |
| LNC_005590 | XLOC_130252 | -- | intronic_lncRNA | 0.374377 | 0.98603 | 0.257848 | 0.257445 |
| LNC_002750 | XLOC_063891 | -- | lincRNA | 0.220033 | 1.01951 | 0 | 0 |
| LNC_002751 | XLOC_063896 | -- | intronic_lncRNA | 0.537871 | 0.13628 | 0.298239 | 0.254051 |
| LNC_002752 | XLOC_063897 | -- | intronic_lncRNA | 0.31227 | 0 | 0 | 0.895755 |
| LNC_002753 | XLOC_063922 | -- | lincRNA | 0.215777 | 2.295759 | 0.316646 | 0.514464 |
| LNC_002754 | XLOC_063922 | -- | lincRNA | 0.707932 | 4.5199 | 0.910027 | 0.353231 |
| LNC_002755 | XLOC_063927 | -- | intronic_lncRNA | 0.553246 | 0.183558 | 0 | 0 |
| LNC_002756 | XLOC_063948 | -- | intronic_lncRNA | 0.092859 | 0.100615 | 0.639289 | 0.170431 |
| LNC_002757 | XLOC_063963 | -- | intronic_lncRNA | 1.128277 | 1.294903 | 1.216708 | 0.76917 |
| LNC_002758 | XLOC_063992 | -- | intronic_lncRNA | 0.05624 | 0.539852 | 0.25442 | 0.32789 |
| LNC_002759 | XLOC_063992 | -- | intronic_lncRNA | 0.442259 | 0 | 0.624776 | 0.029453 |
| LNC_005599 | XLOC_130461 | -- | intronic_lncRNA | 0.577031 | 0.485962 | 0.147708 | 0.393821 |
| LNC_005598 | XLOC_130408 | -- | intronic_lncRNA | 1.735181 | 1.242913 | 2.85014 | 2.038731 |
| LNC_004978 | XLOC_113423 | -- | intronic_lncRNA | 0.466478 | 0.176893 | 0.212688 | 0.248644 |
| LNC_004979 | XLOC_113429 | -- | intronic_lncRNA | 0.557478 | 0.119241 | 0.267559 | 0.625454 |
| LNC_004972 | XLOC_113238 | -- | intronic_lncRNA | 0.427754 | 0.150414 | 0.72291 | 0.413269 |
| LNC_004973 | XLOC_113240 | -- | intronic_lncRNA | 0.805341 | 0.343863 | 0.827286 | 0.593034 |
| LNC_004970 | XLOC_113018 | -- | intronic_lncRNA | 1.192394 | 0.344243 | 0.824728 | 0.309707 |
| LNC_004971 | XLOC_113080 | -- | intronic_lncRNA | 2.233992 | 1.250651 | 2.026884 | 1.474297 |
| LNC_004976 | XLOC_113417 | -- | lincRNA | 0.484414 | 0.096666 | 0.203109 | 0.214956 |
| LNC_004977 | XLOC_113422 | -- | intronic_lncRNA | 0.470969 | 0 | 0.575158 | 0.123719 |
| LNC_004974 | XLOC_113405 | -- | intronic_lncRNA | 0.909591 | 0.618642 | 1.036044 | 0.808596 |
| LNC_004975 | XLOC_113405 | -- | intronic_lncRNA | 0.513006 | 0.19731 | 0.640118 | 1.016246 |
| LNC_001221 | XLOC_026302 | -- | intronic_lncRNA | 0.236008 | 0.12175 | 0.111306 | 0.445149 |
| LNC_001220 | XLOC_026287 | -- | intronic_lncRNA | 61.330791 | 46.426285 | 85.960892 | 85.944656 |
| LNC_001223 | XLOC_026352 | -- | lincRNA | 0.247244 | 0.17353 | 0.423165 | 0.308686 |
| LNC_001222 | XLOC_026303 | -- | intronic_lncRNA | 0 | 0.104692 | 0.422798 | 0 |
| LNC_001225 | XLOC_026401 | -- | intronic_lncRNA | 0 | 0.020403 | 0 | 0.005972 |
| LNC_001224 | XLOC_026388 | -- | lincRNA | 0.677019 | 0.220784 | 0.201144 | 0.238881 |
| LNC_001227 | XLOC_026467 | -- | intronic_lncRNA | 0.89505 | 0.72425 | 0.39241 | 0.106983 |
| LNC_001226 | XLOC_026401 | -- | intronic_lncRNA | 0.592444 | 0.542158 | 0.476462 | 0.598745 |
| LNC_001229 | XLOC_026580 | -- | intronic_lncRNA | 0 | 0.353158 | 0 | 0.558657 |
| LNC_001228 | XLOC_026503 | -- | intronic_lncRNA | 0.69198 | 0.566961 | 0.807695 | 0.522765 |
| LNC_003999 | XLOC_086209 | -- | antisense_lncRNA | 1.17315 | 0.81648 | 0.987525 | 0.449197 |
| LNC_003998 | XLOC_086201 | -- | lincRNA | 0 | 0 | 0 | 0.451569 |
| LNC_003991 | XLOC_086046 | -- | intronic_lncRNA | 1.121411 | 1.127589 | 1.328293 | 0.779307 |
| LNC_003990 | XLOC_086044 | -- | intronic_lncRNA | 0.697907 | 0.128707 | 0.349663 | 1.123136 |
| LNC_003993 | XLOC_086114 | -- | lincRNA | 0 | 0.654128 | 0.30137 | 0 |
| LNC_003992 | XLOC_086112 | -- | intronic_lncRNA | 0.312681 | 0.226456 | 0.109502 | 0.585211 |
| LNC_003995 | XLOC_086136 | -- | intronic_lncRNA | 0.037824 | 0.072514 | 0.048987 | 0.352591 |
| LNC_003994 | XLOC_086123 | -- | lincRNA | 0 | 0.681931 | 0 | 0 |
| LNC_003997 | XLOC_086165 | -- | lincRNA | 0.308788 | 0.123403 | 0.290156 | 0.349726 |
| LNC_003996 | XLOC_086137 | -- | intronic_lncRNA | 0.824081 | 0.210759 | 0.896826 | 0.960186 |
| LNC_002118 | XLOC_048401 | -- | intronic_lncRNA | 0 | 0.712522 | 0 | 0 |
| LNC_002119 | XLOC_048402 | -- | intronic_lncRNA | 0 | 0.213758 | 0.044132 | 0.442458 |
| LNC_002116 | XLOC_048344 | -- | intronic_lncRNA | 0.687497 | 0.632638 | 0.426934 | 0.830971 |
| LNC_002117 | XLOC_048345 | -- | intronic_lncRNA | 0.390512 | 0.499293 | 0.59673 | 0.334489 |
| LNC_002114 | XLOC_048309 | -- | lincRNA | 1.752999 | 0.870032 | 0.707457 | 0.637359 |
| LNC_002115 | XLOC_048311 | -- | lincRNA | 1.087402 | 0.947973 | 0.291213 | 0.152288 |
| LNC_002112 | XLOC_048296 | -- | intronic_lncRNA | 0.599722 | 0.381574 | 0.549047 | 0.463452 |
| LNC_002113 | XLOC_048309 | -- | lincRNA | 1.123585 | 0.524065 | 0.775113 | 0.269835 |
| LNC_002110 | XLOC_048183 | -- | intronic_lncRNA | 0.144833 | 0.333363 | 0.346987 | 0.543486 |
| LNC_002111 | XLOC_048292 | -- | intronic_lncRNA | 0.447251 | 0.638204 | 0.504896 | 0.716745 |
| LNC_003508 | XLOC_075258 | -- | lincRNA | 0 | 1.213174 | 0 | 0.569743 |
| LNC_003509 | XLOC_075258 | -- | lincRNA | 1.723168 | 0.432618 | 0.343293 | 0.318428 |
| LNC_003506 | XLOC_075256 | -- | lincRNA | 2.055905 | 0 | 0.398899 | 0.092291 |

| LNC_003507 | XLOC_075257 | -- | lincRNA | 5.628378 | 2.77822 | 1.149949 | 2.037134 |
| --- | --- | --- | --- | --- | --- | --- | --- |
| LNC_003504 | XLOC_075255 | -- | lincRNA | 3.012809 | 0.972263 | 0.353746 | 1.112582 |
| LNC_003505 | XLOC_075256 | -- | lincRNA | 3.000582 | 1.562913 | 0.552452 | 0.837232 |
| LNC_003502 | XLOC_075253 | -- | lincRNA | 3.253219 | 1.374667 | 0.609458 | 2.313441 |
| LNC_003503 | XLOC_075254 | -- | lincRNA | 0.416835 | 0.040917 | 0.310657 | 0.617065 |
| LNC_003500 | XLOC_075253 | -- | lincRNA | 2.026171 | 1.355736 | 0.675303 | 0.034269 |
| LNC_003501 | XLOC_075253 | -- | lincRNA | 2.414801 | 0.60993 | 0.822274 | 0.817759 |
| LNC_005768 | XLOC_135283 | -- | intronic_lncRNA | 0.47375 | 0.347378 | 0.527598 | 0.541525 |
| LNC_005769 | XLOC_135355 | -- | lincRNA | 0.243728 | 0 | 0.156053 | 0.531732 |
| LNC_005760 | XLOC_135036 | -- | intronic_lncRNA | 0.617885 | 0.521891 | 0 | 0 |
| LNC_005761 | XLOC_135103 | -- | intronic_lncRNA | 0.41939 | 0.278535 | 0.460139 | 0.631779 |
| LNC_005762 | XLOC_135104 | -- | lincRNA | 0.378139 | 0.343174 | 0.51052 | 0.43275 |
| LNC_005763 | XLOC_135152 | -- | intronic_lncRNA | 0.325994 | 0 | 0 | 0.68393 |
| LNC_005764 | XLOC_135168 | -- | intronic_lncRNA | 0.417238 | 0.240329 | 0 | 0.324331 |
| LNC_005765 | XLOC_135217 | -- | intronic_lncRNA | 0.181362 | 0.271358 | 0.456757 | 0.183307 |
| LNC_005766 | XLOC_135221 | -- | intronic_lncRNA | 0.613813 | 0.332567 | 0.770434 | 0.708964 |
| LNC_005767 | XLOC_135224 | -- | intronic_lncRNA | 0 | 0.421756 | 0.494835 | 0 |
| LNC_006111 | XLOC_146053 | -- | intronic_lncRNA | 0.521811 | 0.130924 | 0.63618 | 0.053006 |
| LNC_004419 | XLOC_096529 | -- | intronic_lncRNA | 0.330208 | 0.214655 | 0.207008 | 0.635895 |
| LNC_004418 | XLOC_096520 | -- | intronic_lncRNA | 0 | 0.028547 | 0.923406 | 1.262899 |
| LNC_005832 | XLOC_137467 | -- | intronic_lncRNA | 0 | 0.178005 | 0.66424 | 0.569251 |
| LNC_005833 | XLOC_137470 | -- | intronic_lncRNA | 0.182146 | 0.100567 | 0.707568 | 0.136635 |
| LNC_005834 | XLOC_137470 | -- | intronic_lncRNA | 1.004618 | 0.823891 | 1.030354 | 1.312081 |
| LNC_005835 | XLOC_137470 | -- | intronic_lncRNA | 0.145238 | 0.141077 | 0.430918 | 0.164634 |
| LNC_005836 | XLOC_137483 | -- | intronic_lncRNA | 0.672167 | 0.518231 | 0.921538 | 1.041381 |
| LNC_005837 | XLOC_137484 | -- | intronic_lncRNA | 0.28799 | 0.369736 | 1.26311 | 1.207164 |
| LNC_004411 | XLOC_096322 | -- | intronic_lncRNA | 0.264677 | 0 | 0.436136 | 0.371108 |
| LNC_004410 | XLOC_096315 | -- | intronic_lncRNA | 0 | 0 | 0.953267 | 0.069029 |
| LNC_004413 | XLOC_096345 | -- | intronic_lncRNA | 0.193948 | 0.928349 | 0.678645 | 0.35678 |
| LNC_004412 | XLOC_096345 | -- | intronic_lncRNA | 1.767008 | 1.697222 | 1.36735 | 1.166702 |
| LNC_004415 | XLOC_096494 | -- | lincRNA | 0.112695 | 0.182813 | 1.135139 | 0.212916 |
| LNC_004414 | XLOC_096420 | -- | lincRNA | 0.339281 | 0.573116 | 0.138697 | 0.092813 |
| LNC_004417 | XLOC_096520 | -- | intronic_lncRNA | 2.703619 | 2.403957 | 1.377703 | 1.765011 |
| LNC_004416 | XLOC_096499 | -- | intronic_lncRNA | 0.308094 | 0.076983 | 0.389371 | 0.530681 |
| LNC_006123 | XLOC_146669 | -- | intronic_lncRNA | 0 | 0.322167 | 0 | 0.613371 |
| LNC_006122 | XLOC_146627 | -- | lincRNA | 0.574691 | 1.383232 | 0.03709 | 0.374352 |
| LNC_006121 | XLOC_146601 | -- | intronic_lncRNA | 2.37955 | 0.629545 | 0.322579 | 0.682705 |
| LNC_006120 | XLOC_146578 | -- | intronic_lncRNA | 0.149273 | 0.151303 | 0.465356 | 0.57016 |
| LNC_006127 | XLOC_146715 | -- | intronic_lncRNA | 0.047679 | 0 | 0.130556 | 0.462236 |
| LNC_006126 | XLOC_146714 | -- | intronic_lncRNA | 0 | 0 | 0 | 0.495792 |
| LNC_006125 | XLOC_146713 | -- | intronic_lncRNA | 0 | 0 | 0.476124 | 0.163867 |
| LNC_006124 | XLOC_146671 | -- | intronic_lncRNA | 0.415597 | 0.161558 | 0.09777 | 0.39245 |
| LNC_006129 | XLOC_146810 | -- | intronic_lncRNA | 0.845248 | 0.030098 | 0.341255 | 0.495953 |
| LNC_006128 | XLOC_146757 | -- | intronic_lncRNA | 0.512155 | 0.128603 | 0 | 0 |
| LNC_004861 | XLOC_109516 | -- | intronic_lncRNA | 1.062138 | 0.596838 | 0.893965 | 0.805981 |
| LNC_005166 | XLOC_118018 | -- | intronic_lncRNA | 0.499169 | 1.082904 | 0.580766 | 0.742009 |
| LNC_005167 | XLOC_118032 | -- | intronic_lncRNA | 0.422761 | 0.736767 | 0 | 0.512326 |
| LNC_005164 | XLOC_117994 | -- | intronic_lncRNA | 0.202972 | 0.715957 | 0.781326 | 0.625641 |
| LNC_000405 | XLOC_007092 | -- | intronic_lncRNA | 0.128238 | 2.090192 | 0.198582 | 0.374129 |
| LNC_005162 | XLOC_117962 | -- | intronic_lncRNA | 0 | 0.587933 | 0.204836 | 0.541383 |
| LNC_005163 | XLOC_117963 | -- | intronic_lncRNA | 0.31966 | 0.628857 | 0.768445 | 0.289004 |
| LNC_005160 | XLOC_117887 | -- | intronic_lncRNA | 0.573059 | 0.098527 | 1.165714 | 1.01564 |
| LNC_005161 | XLOC_117962 | -- | intronic_lncRNA | 0.657664 | 0 | 0.030365 | 0.074166 |
| LNC_005168 | XLOC_118040 | -- | intronic_lncRNA | 0.666665 | 0.207341 | 0 | 0.135218 |
| LNC_005169 | XLOC_118057 | -- | intronic_lncRNA | 0.290735 | 0.337249 | 0.32037 | 0.449823 |
| LNC_003698 | XLOC_079185 | -- | lincRNA | 0.629874 | 0.490135 | 0.103895 | 0.6738 |
| LNC_003699 | XLOC_079249 | -- | intronic_lncRNA | 0 | 0 | 0.837206 | 0 |
| LNC_003696 | XLOC_079178 | -- | intronic_lncRNA | 0.145499 | 0.319568 | 0.410132 | 0.31362 |
| LNC_003697 | XLOC_079178 | -- | intronic_lncRNA | 0.497307 | 0.031275 | 0.061498 | 0.241757 |
| LNC_003694 | XLOC_079090 | -- | lincRNA | 0.516752 | 0 | 0 | 0 |
| LNC_003695 | XLOC_079111 | -- | intronic_lncRNA | 1.045949 | 0.285042 | 1.224698 | 0.956864 |
| LNC_003692 | XLOC_079073 | -- | intronic_lncRNA | 7.602951 | 16.018099 | 3.222367 | 1.394569 |
| LNC_003693 | XLOC_079073 | -- | intronic_lncRNA | 0.146406 | 0.401729 | 1.129606 | 0.956836 |
| LNC_003690 | XLOC_079073 | -- | intronic_lncRNA | 1.445599 | 2.893074 | 0.990796 | 0.932386 |
| LNC_003691 | XLOC_079073 | -- | intronic_lncRNA | 0.363542 | 0.609851 | 1.148306 | 1.988389 |
| LNC_005229 | XLOC_119619 | -- | intronic_lncRNA | 0.363758 | 0.779079 | 0.158674 | 0.254454 |
| LNC_005228 | XLOC_119618 | -- | intronic_lncRNA | 0.096836 | 0 | 0.711942 | 0.232996 |
| LNC_005225 | XLOC_119604 | -- | intronic_lncRNA | 0 | 0.337014 | 0.248339 | 0.46137 |
| LNC_005224 | XLOC_119570 | -- | intronic_lncRNA | 0.897062 | 0 | 0.154638 | 0 |
| LNC_005227 | XLOC_119614 | -- | intronic_lncRNA | 0.179574 | 0 | 0.394249 | 0.675392 |
| LNC_005226 | XLOC_119613 | -- | intronic_lncRNA | 0.075797 | 0 | 0.509281 | 0.379449 |
| LNC_005221 | XLOC_119547 | -- | intronic_lncRNA | 0.482793 | 0.269514 | 0.48019 | 0.437334 |
| LNC_005220 | XLOC_119546 | -- | intronic_lncRNA | 0.372978 | 0.454825 | 0.419246 | 0.727962 |
| LNC_005223 | XLOC_119563 | -- | intronic_lncRNA | 0 | 0 | 0.153969 | 0.549736 |
| LNC_005222 | XLOC_119548 | -- | intronic_lncRNA | 0.879624 | 0.50268 | 0.513072 | 0.45078 |
| LNC_002929 | XLOC_067291 | -- | intronic_lncRNA | 0.643301 | 0.409407 | 0.442082 | 0.442006 |
| LNC_002928 | XLOC_067290 | -- | intronic_lncRNA | 0.089577 | 0.566244 | 0.128173 | 0.51382 |

| LNC_002927 | XLOC_067285 | -- | intronic_lncRNA | 0.623462 | 0.240847 | 0.078927 | 0.031782 |
| --- | --- | --- | --- | --- | --- | --- | --- |
| LNC_002926 | XLOC_067255 | -- | intronic_lncRNA | 0.48563 | 0 | 0.129711 | 0.254793 |
| LNC_002925 | XLOC_067244 | -- | intronic_lncRNA | 0.845676 | 0.928891 | 0.68631 | 1.265815 |
| LNC_002924 | XLOC_067235 | -- | intronic_lncRNA | 0.614543 | 0.466483 | 0 | 0.260207 |
| LNC_002923 | XLOC_067234 | -- | intronic_lncRNA | 0.741735 | 0.108173 | 0.317193 | 0.061405 |
| LNC_002922 | XLOC_067233 | -- | intronic_lncRNA | 0 | 0.212452 | 0.636033 | 0 |
| LNC_002921 | XLOC_067216 | -- | intronic_lncRNA | 0.913618 | 0.213233 | 0.207802 | 0.172659 |
| LNC_002920 | XLOC_067216 | -- | intronic_lncRNA | 0.857077 | 0.237477 | 0.62696 | 0.50453 |
| LNC_000077 | XLOC_000619 | -- | lincRNA | 2.288213 | 1.856625 | 1.789652 | 0 |
| LNC_000076 | XLOC_000611 | -- | lincRNA | 5.186471 | 43.423119 | 43.759605 | 42.105145 |
| LNC_000075 | XLOC_000611 | -- | lincRNA | 14.868859 | 19.159157 | 21.330067 | 53.87318 |
| LNC_000074 | XLOC_000611 | -- | lincRNA | 56.798016 | 70.518379 | 47.132374 | 18.499096 |
| LNC_000073 | XLOC_000611 | -- | lincRNA | 4.539845 | 0.222156 | 0.001342 | 2.855596 |
| LNC_000072 | XLOC_000611 | -- | lincRNA | 0.447236 | 3.827149 | 14.296312 | 0.029548 |
| LNC_000071 | XLOC_000611 | -- | lincRNA | 0 | 9.755832 | 0 | 0 |
| LNC_000070 | XLOC_000611 | -- | lincRNA | 0 | 4.347805 | 0 | 0 |
| LNC_000079 | XLOC_000619 | -- | lincRNA | 7.943348 | 0 | 5.158789 | 4.236408 |
| LNC_000078 | XLOC_000619 | -- | lincRNA | 3.2437 | 7.775236 | 0 | 1.415021 |
| LNC_006306 | XLOC_151240 | -- | intronic_lncRNA | 0.815393 | 0.571888 | 0.647839 | 0.514495 |
| LNC_004172 | XLOC_089918 | -- | intronic_lncRNA | 0 | 0.748994 | 0 | 0.159739 |
| LNC_004173 | XLOC_089920 | -- | intronic_lncRNA | 0.517421 | 0.274474 | 0.478892 | 0.058255 |
| LNC_004170 | XLOC_089897 | -- | intronic_lncRNA | 1.779135 | 0.38513 | 0.092111 | 0.054491 |
| LNC_004171 | XLOC_089897 | -- | intronic_lncRNA | 0.898456 | 0.219046 | 0.054067 | 0 |
| LNC_004176 | XLOC_089986 | -- | intronic_lncRNA | 0.102785 | 0 | 0.379687 | 0.513259 |
| LNC_004177 | XLOC_090003 | -- | intronic_lncRNA | 0.240178 | 0 | 0.537839 | 0.063897 |
| LNC_004174 | XLOC_089923 | -- | intronic_lncRNA | 0.161023 | 0 | 0.030746 | 0.497529 |
| LNC_004175 | XLOC_089940 | -- | intronic_lncRNA | 0.569233 | 0 | 0 | 0 |
| LNC_004178 | XLOC_090056 | -- | intronic_lncRNA | 0.317964 | 0.178832 | 0.433035 | 0.42867 |
| LNC_004179 | XLOC_090069 | -- | intronic_lncRNA | 0.14905 | 0.089081 | 0.412705 | 0.481674 |
| LNC_000202 | XLOC_002555 | -- | intronic_lncRNA | 0 | 0.686975 | 0.514889 | 0 |
| LNC_000203 | XLOC_002691 | -- | intronic_lncRNA | 0.131992 | 0 | 0.056136 | 0.550043 |
| LNC_000204 | XLOC_002709 | -- | intronic_lncRNA | 0.552847 | 0.305206 | 1.357231 | 1.437873 |
| LNC_000205 | XLOC_002806 | -- | intronic_lncRNA | 0 | 0 | 0.472865 | 0 |
| LNC_000206 | XLOC_002807 | -- | lincRNA | 0.079684 | 0.129727 | 0.822775 | 1.255248 |
| LNC_000207 | XLOC_002813 | -- | intronic_lncRNA | 0.813765 | 0.371963 | 0 | 0.519949 |
| LNC_000208 | XLOC_002939 | -- | intronic_lncRNA | 0.304264 | 0.322996 | 0.405991 | 0.419273 |
| LNC_000209 | XLOC_002941 | -- | intronic_lncRNA | 0.361346 | 0.587178 | 0 | 0.427323 |
| LNC_006304 | XLOC_151148 | -- | intronic_lncRNA | 1.033917 | 0.681588 | 0.389518 | 0.292778 |
| LNC_002325 | XLOC_053845 | -- | intronic_lncRNA | 0.663088 | 0.640447 | 0.916713 | 0.983581 |
| LNC_002324 | XLOC_053763 | -- | antisense_lncRNA | 0.52408 | 0.033296 | 0.540497 | 0.83794 |
| LNC_002327 | XLOC_053847 | -- | intronic_lncRNA | 0.721144 | 0.672456 | 0.11178 | 0 |
| LNC_002326 | XLOC_053846 | -- | intronic_lncRNA | 0.368118 | 1.916094 | 1.268834 | 1.469886 |
| LNC_002321 | XLOC_053702 | -- | intronic_lncRNA | 0 | 0.51533 | 0.198041 | 0.215736 |
| LNC_002320 | XLOC_053660 | -- | lincRNA | 0.155462 | 0.137872 | 0.692887 | 0.47368 |
| LNC_002323 | XLOC_053740 | -- | intronic_lncRNA | 0.127239 | 0.156332 | 0.506429 | 0.366254 |
| LNC_002322 | XLOC_053711 | -- | lincRNA | 0.354806 | 0.580208 | 0.104207 | 0 |
| LNC_002329 | XLOC_053901 | -- | intronic_lncRNA | 0.632127 | 0.14877 | 0.4118 | 0.334569 |
| LNC_002328 | XLOC_053870 | -- | antisense_lncRNA | 0.353562 | 0 | 0 | 0.819931 |
| LNC_003335 | XLOC_075175 | -- | lincRNA | 1.071303 | 0 | 0 | 0 |
| LNC_003334 | XLOC_075175 | -- | lincRNA | 5.275603 | 2.616156 | 1.904772 | 2.452069 |
| LNC_003337 | XLOC_075175 | -- | lincRNA | 0.888164 | 0 | 0 | 0 |
| LNC_003336 | XLOC_075175 | -- | lincRNA | 1.27028 | 0.575265 | 0.178346 | 0.395422 |
| LNC_003331 | XLOC_075175 | -- | lincRNA | 1.142893 | 1.084779 | 0.602301 | 0 |
| LNC_003330 | XLOC_075175 | -- | lincRNA | 1.297523 | 0.959292 | 0.446684 | 0.476529 |
| LNC_003333 | XLOC_075175 | -- | lincRNA | 1.974411 | 0.767717 | 0.509766 | 0.460984 |
| LNC_003332 | XLOC_075175 | -- | lincRNA | 0.912727 | 0.216992 | 0.19267 | 0.235845 |
| LNC_003339 | XLOC_075175 | -- | lincRNA | 5.41687 | 2.091813 | 0.757256 | 1.087748 |
| LNC_003338 | XLOC_075175 | -- | lincRNA | 1.420968 | 0.139881 | 0.091572 | 0.045743 |
| LNC_000679 | XLOC_013576 | -- | intronic_lncRNA | 0.515584 | 0.826826 | 0.18511 | 0.561708 |
| LNC_000678 | XLOC_013575 | -- | intronic_lncRNA | 0.241895 | 0.257911 | 0 | 1.078964 |
| LNC_000675 | XLOC_013418 | -- | intronic_lncRNA | 3.710608 | 2.078895 | 2.377743 | 2.146668 |
| LNC_000674 | XLOC_013413 | -- | intronic_lncRNA | 0.904868 | 0.773002 | 0.747485 | 0.818998 |
| LNC_000677 | XLOC_013471 | -- | intronic_lncRNA | 1.836801 | 2.848266 | 1.549025 | 1.866979 |
| LNC_000676 | XLOC_013433 | -- | intronic_lncRNA | 0.323316 | 0.633634 | 0.291731 | 0.142669 |
| LNC_000671 | XLOC_013265 | -- | intronic_lncRNA | 0.356017 | 0 | 0.626103 | 1.162982 |
| LNC_000670 | XLOC_013265 | -- | intronic_lncRNA | 1.316628 | 1.108819 | 2.642787 | 2.263191 |
| LNC_000673 | XLOC_013379 | -- | intronic_lncRNA | 0 | 0 | 0 | 0.46885 |
| LNC_000672 | XLOC_013367 | -- | intronic_lncRNA | 0.191307 | 0.193376 | 0.712632 | 0.089731 |
| LNC_005587 | XLOC_130131 | -- | intronic_lncRNA | 0.545459 | 0.181816 | 0.294491 | 0 |
| LNC_001678 | XLOC_036669 | -- | intronic_lncRNA | 0.230479 | 0.367304 | 0.429996 | 0.220135 |
| LNC_001679 | XLOC_036670 | -- | intronic_lncRNA | 0.419648 | 0.416289 | 0.539245 | 0.599493 |
| LNC_001672 | XLOC_036431 | -- | intronic_lncRNA | 0.050617 | 0 | 0.19374 | 0.43046 |
| LNC_001673 | XLOC_036477 | -- | lincRNA | 0.348658 | 0.402773 | 0.351318 | 0.265738 |
| LNC_001670 | XLOC_036420 | -- | intronic_lncRNA | 0 | 0 | 0.165019 | 0.506234 |
| LNC_001671 | XLOC_036429 | -- | intronic_lncRNA | 0.190525 | 0 | 0.467095 | 0.235601 |
| LNC_001676 | XLOC_036543 | -- | lincRNA | 0.201365 | 0.914711 | 0 | 0 |
| LNC_001677 | XLOC_036609 | -- | intronic_lncRNA | 0.40455 | 1.309285 | 0.510076 | 0.721531 |

| LNC_001674 | XLOC_036488 | -- | intronic_lncRNA | 0.485329 | 0.289666 | 0.449603 | 0.280071 |
| --- | --- | --- | --- | --- | --- | --- | --- |
| LNC_001675 | XLOC_036542 | -- | lincRNA | 0.217172 | 0.716023 | 0 | 0 |
| LNC_004958 | XLOC_112451 | -- | intronic_lncRNA | 0.804562 | 0.602069 | 0.406055 | 0.121877 |
| LNC_004959 | XLOC_112458 | -- | intronic_lncRNA | 0.326017 | 0.429737 | 0.476825 | 0.5078 |
| LNC_004954 | XLOC_112391 | -- | lincRNA | 0.581465 | 0.187129 | 0.124853 | 0.040873 |
| LNC_004955 | XLOC_112435 | -- | intronic_lncRNA | 0.288419 | 0.204307 | 0.664023 | 0.747638 |
| LNC_004956 | XLOC_112436 | -- | intronic_lncRNA | 0.315639 | 1.15844 | 1.014222 | 1.009395 |
| LNC_004957 | XLOC_112445 | -- | intronic_lncRNA | 1.172537 | 0.704439 | 1.001411 | 0.860762 |
| LNC_004950 | XLOC_112292 | -- | intronic_lncRNA | 0.022325 | 0.112289 | 0.759308 | 1.144042 |
| LNC_004951 | XLOC_112330 | -- | intronic_lncRNA | 1.170283 | 0.832535 | 0.58963 | 0.558194 |
| LNC_004952 | XLOC_112342 | -- | intronic_lncRNA | 0.536783 | 0.685803 | 0.674286 | 0.616127 |
| LNC_004953 | XLOC_112343 | -- | intronic_lncRNA | 0.630848 | 0.612819 | 0.536745 | 0.39136 |
| LNC_001209 | XLOC_026040 | -- | intronic_lncRNA | 0.427351 | 0.598425 | 0.445497 | 0.440446 |
| LNC_001208 | XLOC_026032 | -- | intronic_lncRNA | 0.601619 | 0.776658 | 0.67465 | 0.842098 |
| LNC_001203 | XLOC_025982 | -- | intronic_lncRNA | 0.418602 | 2.230142 | 1.304362 | 0.369241 |
| LNC_001202 | XLOC_025982 | -- | intronic_lncRNA | 0.832811 | 2.471817 | 1.11587 | 1.4213 |
| LNC_001201 | XLOC_025925 | -- | intronic_lncRNA | 1.015346 | 0.342932 | 0.382729 | 0.360921 |
| LNC_001200 | XLOC_025919 | -- | intronic_lncRNA | 0.309464 | 0.898467 | 0.046381 | 0.640686 |
| LNC_001207 | XLOC_026023 | -- | intronic_lncRNA | 0.095884 | 0.096996 | 0.342934 | 1.05155 |
| LNC_001206 | XLOC_026023 | -- | intronic_lncRNA | 0.672468 | 0 | 0 | 0 |
| LNC_001205 | XLOC_026023 | -- | intronic_lncRNA | 0.566824 | 1.097611 | 0.634924 | 0 |
| LNC_001204 | XLOC_026013 | -- | lincRNA | 0.343627 | 0.26244 | 0.478037 | 0.257499 |
| LNC_004791 | XLOC_107016 | -- | intronic_lncRNA | 0.250645 | 0.103714 | 0.560943 | 0.137484 |
| LNC_005589 | XLOC_130137 | -- | intronic_lncRNA | 0.556444 | 0.094564 | 0.306334 | 0.253873 |
| LNC_002130 | XLOC_048594 | -- | intronic_lncRNA | 1.035201 | 1.129314 | 0.821621 | 0.706705 |
| LNC_000536 | XLOC_009970 | -- | intronic_lncRNA | 0.798488 | 0 | 0.061775 | 0.126398 |
| LNC_002132 | XLOC_048643 | -- | intronic_lncRNA | 5.749767 | 3.399158 | 12.336109 | 9.506069 |
| LNC_002133 | XLOC_048676 | -- | intronic_lncRNA | 0.518255 | 0.197432 | 0.536551 | 0.603076 |
| LNC_002134 | XLOC_048676 | -- | intronic_lncRNA | 0.126747 | 0.298209 | 0 | 0 |
| LNC_002135 | XLOC_048676 | -- | intronic_lncRNA | 1.054058 | 0.043657 | 0.285998 | 0.42321 |
| LNC_002136 | XLOC_048678 | -- | intronic_lncRNA | 0.239976 | 0.468383 | 0.786975 | 0.821341 |
| LNC_005017 | XLOC_114571 | -- | intronic_lncRNA | 0 | 0.586569 | 0 | 0 |
| LNC_002138 | XLOC_048711 | -- | lincRNA | 1.968047 | 2.557915 | 13.693279 | 10.99347 |
| LNC_002139 | XLOC_048722 | -- | lincRNA | 0.144 | 0.553032 | 0.479299 | 0.14057 |
| LNC_005014 | XLOC_114560 | -- | intronic_lncRNA | 0.659853 | 0.871836 | 0.874405 | 0.886337 |
| LNC_003520 | XLOC_075263 | -- | lincRNA | 0.263013 | 2.063105 | 0.837424 | 1.077536 |
| LNC_003521 | XLOC_075263 | -- | lincRNA | 1.420635 | 0.311367 | 0.267726 | 0.05397 |
| LNC_003522 | XLOC_075263 | -- | lincRNA | 2.601578 | 0.926284 | 0.49467 | 0.27284 |
| LNC_005015 | XLOC_114560 | -- | intronic_lncRNA | 0 | 0 | 0 | 0.738263 |
| LNC_003524 | XLOC_075263 | -- | lincRNA | 7.467575 | 1.302449 | 2.104482 | 1.882493 |
| LNC_003525 | XLOC_075263 | -- | lincRNA | 2.308236 | 0 | 0.606704 | 1.032202 |
| LNC_003526 | XLOC_075264 | -- | lincRNA | 2.455305 | 2.283128 | 0.811807 | 0.872418 |
| LNC_003527 | XLOC_075265 | -- | lincRNA | 4.151591 | 0.266795 | 1.295794 | 0.955405 |
| LNC_003528 | XLOC_075265 | -- | lincRNA | 2.084608 | 2.719136 | 0.241304 | 0.217316 |
| LNC_005012 | XLOC_114381 | -- | intronic_lncRNA | 0.669746 | 0 | 0 | 0 |
| LNC_005013 | XLOC_114559 | -- | intronic_lncRNA | 0 | 0.147995 | 0.497988 | 0 |
| LNC_005782 | XLOC_135688 | -- | intronic_lncRNA | 0.803768 | 0.092365 | 0.149805 | 0.247378 |
| LNC_005783 | XLOC_135690 | -- | intronic_lncRNA | 0.926173 | 0.597494 | 1.542636 | 0.904172 |
| LNC_005780 | XLOC_135662 | -- | intronic_lncRNA | 3.469401 | 2.253368 | 2.803427 | 2.362224 |
| LNC_005781 | XLOC_135662 | -- | intronic_lncRNA | 0 | 0.883445 | 0 | 0 |
| LNC_005786 | XLOC_136121 | -- | intronic_lncRNA | 0.111008 | 0.845975 | 0.489652 | 0.089905 |
| LNC_005010 | XLOC_114349 | -- | intronic_lncRNA | 0.175989 | 0.454862 | 0.458018 | 0.903835 |
| LNC_005784 | XLOC_135700 | -- | intronic_lncRNA | 0.821565 | 0.588671 | 0.46178 | 0.970155 |
| LNC_005785 | XLOC_135947 | -- | intronic_lncRNA | 0 | 0 | 0.499774 | 0 |
| LNC_005788 | XLOC_136143 | -- | intronic_lncRNA | 0 | 0.155023 | 0.447097 | 0.220901 |
| LNC_005011 | XLOC_114349 | -- | intronic_lncRNA | 0.541764 | 0.043612 | 0.827171 | 0.010873 |
| LNC_006330 | XLOC_152028 | -- | antisense_lncRNA | 0.412838 | 1.080602 | 0.129211 | 0 |
| LNC_006331 | XLOC_152049 | -- | intronic_lncRNA | 0.406142 | 0.394042 | 0.576097 | 0.432592 |
| LNC_006336 | XLOC_152114 | -- | intronic_lncRNA | 1.195267 | 0.227448 | 0.0921 | 0 |
| LNC_006337 | XLOC_152115 | -- | intronic_lncRNA | 0.777876 | 0.598444 | 0.727632 | 0.427589 |
| LNC_006334 | XLOC_152105 | -- | intronic_lncRNA | 0.784136 | 0 | 0.163128 | 0 |
| LNC_004852 | XLOC_109089 | -- | intronic_lncRNA | 0.553776 | 0.055076 | 0.175655 | 0.672005 |
| LNC_006335 | XLOC_152106 | -- | intronic_lncRNA | 0.529142 | 0.417661 | 0.244877 | 0.340797 |
| LNC_005018 | XLOC_114626 | -- | intronic_lncRNA | 0.638169 | 0.510876 | 0.857667 | 0.741847 |
| LNC_005019 | XLOC_114627 | -- | intronic_lncRNA | 0.616143 | 0.628935 | 0.516523 | 0.620348 |
| LNC_006338 | XLOC_152118 | -- | intronic_lncRNA | 0.887291 | 0.15952 | 0 | 0.037527 |
| LNC_006339 | XLOC_152138 | -- | intronic_lncRNA | 0.412097 | 0.560538 | 0.264015 | 0.49646 |
| LNC_005706 | XLOC_133593 | -- | lincRNA | 1.327263 | 0.422404 | 0.589724 | 0.583229 |
| LNC_000576 | XLOC_010756 | -- | intronic_lncRNA | 0.074803 | 0.140775 | 0.612983 | 0.210498 |
| LNC_004789 | XLOC_106924 | -- | intronic_lncRNA | 0.126322 | 0.040732 | 0.410361 | 0.856954 |
| LNC_004788 | XLOC_106922 | -- | intronic_lncRNA | 0.83146 | 0.068771 | 0.834904 | 0.660446 |
| LNC_004781 | XLOC_106792 | -- | intronic_lncRNA | 2.03858 | 0.548378 | 0.303459 | 0.231503 |
| LNC_004780 | XLOC_106788 | -- | lincRNA | 1.828683 | 0.350623 | 0.149531 | 0.073556 |
| LNC_004783 | XLOC_106804 | -- | intronic_lncRNA | 0 | 0 | 0.592075 | 0.355841 |
| LNC_004782 | XLOC_106792 | -- | intronic_lncRNA | 0.506375 | 0.179804 | 0.241303 | 0.405863 |
| LNC_004785 | XLOC_106912 | -- | intronic_lncRNA | 0.402436 | 0.313012 | 0.515594 | 0.602092 |
| LNC_004784 | XLOC_106911 | -- | intronic_lncRNA | 0.639568 | 0 | 0.229306 | 0.158458 |

| LNC_004787 | XLOC_106920 | -- | intronic_lncRNA | 0.027947 | 1.902948 | 0.150549 | 0 |
| --- | --- | --- | --- | --- | --- | --- | --- |
| LNC_004786 | XLOC_106919 | -- | intronic_lncRNA | 0.190668 | 0.26583 | 0.547771 | 0.092273 |
| LNC_005707 | XLOC_133596 | -- | lincRNA | 0.651441 | 0.448983 | 0.272295 | 0.45522 |
| LNC_000577 | XLOC_010761 | -- | intronic_lncRNA | 0.680817 | 0.267266 | 0.170211 | 0.173981 |
| LNC_001441 | XLOC_031349 | -- | intronic_lncRNA | 0.702433 | 0.315564 | 0.321972 | 0.361752 |
| LNC_001440 | XLOC_031326 | -- | intronic_lncRNA | 0.772932 | 0.066999 | 0.55462 | 0.41517 |
| LNC_001443 | XLOC_031366 | -- | lincRNA | 0.259221 | 0 | 0.443125 | 0.286833 |
| LNC_001442 | XLOC_031356 | -- | intronic_lncRNA | 0.119855 | 0 | 0.48424 | 0 |
| LNC_001445 | XLOC_031458 | -- | intronic_lncRNA | 0.249094 | 0.382453 | 0.583671 | 0.230799 |
| LNC_001444 | XLOC_031368 | -- | lincRNA | 0.475859 | 0.357285 | 0.519545 | 0.433951 |
| LNC_001447 | XLOC_031505 | -- | intronic_lncRNA | 0.464842 | 0.1296 | 0.25964 | 0.28288 |
| LNC_001446 | XLOC_031489 | -- | intronic_lncRNA | 1.158797 | 0.63271 | 0.783404 | 1.049119 |
| LNC_001449 | XLOC_031512 | -- | intronic_lncRNA | 0.272051 | 0.602833 | 0.637687 | 0.167288 |
| LNC_001448 | XLOC_031511 | -- | intronic_lncRNA | 0 | 0.151831 | 0.035421 | 0.545035 |
| LNC_004433 | XLOC_096928 | -- | intronic_lncRNA | 0.356339 | 0.245831 | 0.535143 | 0.491256 |
| LNC_004432 | XLOC_096922 | -- | lincRNA | 0.907703 | 0.245729 | 0.200839 | 0.129665 |
| LNC_004431 | XLOC_096867 | -- | lincRNA | 0.248478 | 0.132836 | 0.228736 | 0.622391 |
| LNC_004430 | XLOC_096738 | -- | intronic_lncRNA | 0.696166 | 0.791664 | 0.832658 | 1.096842 |
| LNC_004437 | XLOC_096974 | -- | lincRNA | 0.856882 | 1.233593 | 0.935254 | 0.899905 |
| LNC_004436 | XLOC_096973 | -- | lincRNA | 1.015003 | 1.131828 | 0.599771 | 0.60152 |
| LNC_004435 | XLOC_096955 | -- | lincRNA | 0.721887 | 0 | 0.036132 | 0 |
| LNC_004434 | XLOC_096949 | -- | lincRNA | 0.750463 | 0.195972 | 0.629571 | 0.812695 |
| LNC_004439 | XLOC_096983 | -- | intronic_lncRNA | 2.111188 | 12.619226 | 8.201643 | 10.129663 |
| LNC_004438 | XLOC_096983 | -- | intronic_lncRNA | 2.557963 | 2.818084 | 0 | 0.052838 |
| LNC_006145 | XLOC_147144 | -- | intronic_lncRNA | 0.312483 | 0.469108 | 0.850258 | 0.783987 |
| LNC_006144 | XLOC_147135 | -- | intronic_lncRNA | 0.848892 | 0.688036 | 0.858454 | 0.796083 |
| LNC_006147 | XLOC_147196 | -- | intronic_lncRNA | 0 | 0 | 0.675846 | 0 |
| LNC_006146 | XLOC_147196 | -- | intronic_lncRNA | 0.196618 | 0.145592 | 0 | 0.473045 |
| LNC_006141 | XLOC_147081 | -- | intronic_lncRNA | 0.802173 | 0.401778 | 0.306629 | 0.249311 |
| LNC_006140 | XLOC_147080 | -- | intronic_lncRNA | 0.207936 | 0.046461 | 0.189155 | 0.762749 |
| LNC_006143 | XLOC_147135 | -- | intronic_lncRNA | 0.351876 | 0 | 0.493806 | 0.566721 |
| LNC_006142 | XLOC_147094 | -- | intronic_lncRNA | 0.077477 | 0.037325 | 0.746585 | 0.646282 |
| LNC_006149 | XLOC_147213 | -- | intronic_lncRNA | 0 | 0 | 0 | 0.666761 |
| LNC_006148 | XLOC_147197 | -- | intronic_lncRNA | 0 | 0.641045 | 0.38297 | 0.04293 |
| LNC_005823 | XLOC_137262 | -- | intronic_lncRNA | 1.088745 | 0.77053 | 1.003578 | 0.789138 |
| LNC_005822 | XLOC_137261 | -- | intronic_lncRNA | 0.475047 | 0.550608 | 0.700992 | 0.532731 |
| LNC_000796 | XLOC_016957 | -- | intronic_lncRNA | 0.785806 | 0.328945 | 0.784179 | 1.015917 |
| LNC_000797 | XLOC_016981 | -- | intronic_lncRNA | 0 | 0.088956 | 0.432251 | 0 |
| LNC_000794 | XLOC_016918 | -- | intronic_lncRNA | 0.104362 | 0.572357 | 0 | 0.362552 |
| LNC_000795 | XLOC_016954 | -- | intronic_lncRNA | 0.300587 | 0.273468 | 0.980556 | 0.583998 |
| LNC_000792 | XLOC_016916 | -- | intronic_lncRNA | 0.696482 | 0.146094 | 0.618396 | 0.678226 |
| LNC_000793 | XLOC_016917 | -- | intronic_lncRNA | 0.078973 | 0.391398 | 0.474514 | 0.954731 |
| LNC_000790 | XLOC_016873 | -- | intronic_lncRNA | 0.183833 | 0.304956 | 0.645164 | 0.013114 |
| LNC_000791 | XLOC_016873 | -- | intronic_lncRNA | 0.653174 | 0.485247 | 0.034037 | 0.37516 |
| LNC_000798 | XLOC_016989 | -- | intronic_lncRNA | 0.330625 | 0.465081 | 0 | 0.251863 |
| LNC_000799 | XLOC_016989 | -- | intronic_lncRNA | 0.506558 | 0.250278 | 0 | 0.24515 |
| LNC_005829 | XLOC_137312 | -- | intronic_lncRNA | 0.484414 | 0.549382 | 0.195714 | 0.661398 |
| LNC_005828 | XLOC_137307 | -- | intronic_lncRNA | 1.325414 | 0 | 0.14645 | 0 |
| LNC_005148 | XLOC_117580 | -- | intronic_lncRNA | 1.320083 | 0.608087 | 1.991131 | 1.740485 |
| LNC_000429 | XLOC_007697 | -- | intronic_lncRNA | 0 | 0.174172 | 0 | 0.559891 |
| LNC_000420 | XLOC_007390 | -- | intronic_lncRNA | 0.038407 | 0.123049 | 0.153367 | 0.680064 |
| LNC_005141 | XLOC_117371 | -- | intronic_lncRNA | 0.290248 | 1.147171 | 0.797681 | 0.627955 |
| LNC_005142 | XLOC_117480 | -- | lincRNA | 0 | 0 | 0.286279 | 0.730538 |
| LNC_000423 | XLOC_007405 | -- | lincRNA | 0.152224 | 0.513457 | 0.148073 | 0.060136 |
| LNC_005144 | XLOC_117488 | -- | intronic_lncRNA | 0.799949 | 0.239225 | 0 | 0.161571 |
| LNC_005145 | XLOC_117522 | -- | intronic_lncRNA | 0.216596 | 0 | 0 | 0.567531 |
| LNC_005146 | XLOC_117563 | -- | lincRNA | 0.712689 | 0.36446 | 0.039263 | 0.0131 |
| LNC_000427 | XLOC_007619 | -- | intronic_lncRNA | 0.442233 | 0 | 0.479299 | 0 |
| LNC_002499 | XLOC_057943 | -- | intronic_lncRNA | 0.569638 | 1.838744 | 0.73752 | 0.946585 |
| LNC_002498 | XLOC_057926 | -- | intronic_lncRNA | 1.013701 | 1.558906 | 0.888424 | 1.0945 |
| LNC_002497 | XLOC_057920 | -- | intronic_lncRNA | 0.667015 | 0.485278 | 1.594058 | 1.67031 |
| LNC_002496 | XLOC_057909 | -- | intronic_lncRNA | 0.061065 | 0.675689 | 0.03138 | 0.101025 |
| LNC_002495 | XLOC_057909 | -- | intronic_lncRNA | 0.887673 | 0.800007 | 0.741208 | 1.020456 |
| LNC_002494 | XLOC_057898 | -- | intronic_lncRNA | 0.312808 | 0 | 0.344382 | 0.669538 |
| LNC_002493 | XLOC_057896 | -- | intronic_lncRNA | 0 | 0 | 0.089912 | 0.464982 |
| LNC_002492 | XLOC_057784 | -- | lincRNA | 0 | 0 | 0.423241 | 0.313033 |
| LNC_002491 | XLOC_057773 | -- | intronic_lncRNA | 0.502656 | 0.630932 | 0.39325 | 0.548095 |
| LNC_002490 | XLOC_057771 | -- | intronic_lncRNA | 0.543267 | 0.21411 | 0 | 0 |
| LNC_004899 | XLOC_111349 | -- | intronic_lncRNA | 0 | 0 | 0.415675 | 0 |
| LNC_004898 | XLOC_111314 | -- | intronic_lncRNA | 0.998291 | 0.035726 | 0.294991 | 0.25943 |
| LNC_004895 | XLOC_111235 | -- | intronic_lncRNA | 0 | 0 | 0.41348 | 0 |
| LNC_004894 | XLOC_111231 | -- | intronic_lncRNA | 0.32934 | 0.084485 | 0.550906 | 0.460282 |
| LNC_004897 | XLOC_111244 | -- | intronic_lncRNA | 0.643834 | 0.830224 | 0.737291 | 0.557525 |
| LNC_004896 | XLOC_111243 | -- | intronic_lncRNA | 0.098443 | 0.221627 | 0.34496 | 0.534034 |
| LNC_004891 | XLOC_111145 | -- | lincRNA | 0 | 0 | 0.258397 | 0.48441 |
| LNC_004890 | XLOC_111128 | -- | lincRNA | 0 | 0.140673 | 0.115191 | 0.706213 |
| LNC_004893 | XLOC_111230 | -- | intronic_lncRNA | 0.652254 | 0 | 0.420033 | 0.332349 |

| LNC_004892 | XLOC_111230 | -- | intronic_lncRNA | 0.084521 | 0.67016 | 0.126954 | 0.102345 |
| --- | --- | --- | --- | --- | --- | --- | --- |
| LNC_005207 | XLOC_118984 | -- | intronic_lncRNA | 0.144639 | 0 | 0.370826 | 0.432556 |
| LNC_005206 | XLOC_118980 | -- | intronic_lncRNA | 0.638844 | 0.489477 | 0.499147 | 0.410082 |
| LNC_005205 | XLOC_118979 | -- | intronic_lncRNA | 0.654478 | 0.108233 | 0.051926 | 0.079185 |
| LNC_005204 | XLOC_118979 | -- | intronic_lncRNA | 0.965424 | 0.064419 | 0.268042 | 0.113751 |
| LNC_005203 | XLOC_118979 | -- | intronic_lncRNA | 0.055259 | 0.551281 | 0.826092 | 0.736727 |
| LNC_005202 | XLOC_118958 | -- | lincRNA | 0 | 0 | 0.401464 | 0 |
| LNC_005201 | XLOC_118903 | -- | intronic_lncRNA | 0.658382 | 0.838351 | 0.541628 | 0.940396 |
| LNC_005200 | XLOC_118875 | -- | lincRNA | 0.307287 | 0.217634 | 0.088126 | 0.484611 |
| LNC_005209 | XLOC_119011 | -- | antisense_lncRNA | 0.329601 | 0 | 0 | 0.540193 |
| LNC_005208 | XLOC_119011 | -- | intronic_lncRNA | 0.652055 | 0.874398 | 0.459515 | 0.252614 |
| LNC_004239 | XLOC_091856 | -- | intronic_lncRNA | 0.510367 | 0.656041 | 0.652634 | 0.4448 |
| LNC_004238 | XLOC_091856 | -- | intronic_lncRNA | 0.071069 | 0.158636 | 1.028585 | 0.617454 |
| LNC_004231 | XLOC_091797 | -- | intronic_lncRNA | 0.416023 | 0.84738 | 0.483159 | 0.643547 |
| LNC_004230 | XLOC_091786 | -- | antisense_lncRNA | 3.269087 | 1.341933 | 0.790287 | 0.359116 |
| LNC_004233 | XLOC_091813 | -- | intronic_lncRNA | 0.775424 | 0.756257 | 0.473904 | 1.008305 |
| LNC_004232 | XLOC_091800 | -- | intronic_lncRNA | 0.346616 | 0.302962 | 0.243663 | 0.71453 |
| LNC_004235 | XLOC_091845 | -- | intronic_lncRNA | 0.748898 | 0.241409 | 0.52468 | 0.460731 |
| LNC_004234 | XLOC_091813 | -- | intronic_lncRNA | 0.20593 | 0.23911 | 0.586485 | 0 |
| LNC_004237 | XLOC_091856 | -- | intronic_lncRNA | 1.142697 | 0.570189 | 0.970898 | 0.799636 |
| LNC_004236 | XLOC_091849 | -- | intronic_lncRNA | 0.652607 | 0.185778 | 0.203566 | 0.531258 |
| LNC_000200 | XLOC_002534 | -- | intronic_lncRNA | 0.666436 | 0.341276 | 0.290461 | 0.116747 |
| LNC_004154 | XLOC_089545 | -- | intronic_lncRNA | 0 | 0.174367 | 0.403187 | 0.150497 |
| LNC_004155 | XLOC_089546 | -- | intronic_lncRNA | 0.163009 | 0 | 0.235647 | 0.810504 |
| LNC_004156 | XLOC_089547 | -- | intronic_lncRNA | 0.229761 | 0.258284 | 0.652894 | 0.701663 |
| LNC_004157 | XLOC_089619 | -- | intronic_lncRNA | 0.122915 | 0 | 0.031792 | 0.65407 |
| LNC_004150 | XLOC_089412 | -- | intronic_lncRNA | 0.353906 | 0 | 0.144968 | 0.430071 |
| LNC_004151 | XLOC_089439 | -- | antisense_lncRNA | 1.162411 | 1.112608 | 0.570985 | 0.536826 |
| LNC_004152 | XLOC_089468 | -- | intronic_lncRNA | 0.666012 | 0 | 0 | 0 |
| LNC_004153 | XLOC_089488 | -- | intronic_lncRNA | 0.077433 | 0.10646 | 0.23123 | 0.45359 |
| LNC_004158 | XLOC_089619 | -- | intronic_lncRNA | 0.593886 | 0.408396 | 0.908745 | 0.201869 |
| LNC_004159 | XLOC_089620 | -- | intronic_lncRNA | 0.222173 | 0.490731 | 0.313484 | 0.450106 |
| LNC_000201 | XLOC_002552 | -- | intronic_lncRNA | 0.543267 | 0 | 0.043898 | 0.087782 |
| LNC_005467 | XLOC_126175 | -- | lincRNA | 0.075726 | 0.361745 | 0.897542 | 0.765789 |
| LNC_005466 | XLOC_126175 | -- | lincRNA | 1.06219 | 0 | 1.25016 | 0.578315 |
| LNC_005465 | XLOC_126175 | -- | lincRNA | 0.337527 | 0.247839 | 1.228725 | 0.453932 |
| LNC_005464 | XLOC_126172 | -- | intronic_lncRNA | 0.591149 | 0 | 0 | 0 |
| LNC_005463 | XLOC_126128 | -- | intronic_lncRNA | 0.250319 | 1.778576 | 0.051437 | 0.308993 |
| LNC_005462 | XLOC_126128 | -- | intronic_lncRNA | 1.0501 | 0.882801 | 1.090174 | 1.01461 |
| LNC_005461 | XLOC_126088 | -- | intronic_lncRNA | 0.470812 | 0.030748 | 0.246523 | 0.253243 |
| LNC_005460 | XLOC_126050 | -- | intronic_lncRNA | 0.804206 | 0.851475 | 0.772735 | 0.618266 |
| LNC_002309 | XLOC_053013 | -- | intronic_lncRNA | 0.48644 | 0.237822 | 0 | 0.438847 |
| LNC_002308 | XLOC_052990 | -- | intronic_lncRNA | 0.579597 | 0.12304 | 0.088488 | 0.117702 |
| LNC_002307 | XLOC_052963 | -- | intronic_lncRNA | 0 | 0.861398 | 0 | 0 |
| LNC_002306 | XLOC_052963 | -- | intronic_lncRNA | 0.642526 | 0.32454 | 1.15559 | 0.633101 |
| LNC_004287 | XLOC_093368 | -- | intronic_lncRNA | 0.933205 | 0.473854 | 1.031334 | 1.252998 |
| LNC_002304 | XLOC_052949 | -- | intronic_lncRNA | 0.165712 | 0.010149 | 0.100708 | 0.366882 |
| LNC_002303 | XLOC_052939 | -- | intronic_lncRNA | 0.572489 | 0.199062 | 0.547379 | 0.921629 |
| LNC_002302 | XLOC_052932 | -- | intronic_lncRNA | 0.271814 | 0.738648 | 0.784778 | 0.746565 |
| LNC_002301 | XLOC_052924 | -- | intronic_lncRNA | 0.830853 | 0.928278 | 0.890703 | 0.996299 |
| LNC_002300 | XLOC_052870 | -- | intronic_lncRNA | 0.599274 | 0 | 0 | 0.232933 |
| LNC_003319 | XLOC_075165 | -- | lincRNA | 3.164426 | 1.404893 | 0.195437 | 0.75021 |
| LNC_003318 | XLOC_075163 | -- | lincRNA | 4.512629 | 1.487106 | 1.502896 | 1.571653 |
| LNC_003317 | XLOC_075162 | -- | lincRNA | 1.767444 | 0.242302 | 0.090196 | 0.294891 |
| LNC_003316 | XLOC_075162 | -- | lincRNA | 2.584392 | 2.234366 | 0.330912 | 1.512186 |
| LNC_003315 | XLOC_075162 | -- | lincRNA | 6.39775 | 2.848856 | 1.543825 | 3.629721 |
| LNC_003314 | XLOC_075162 | -- | lincRNA | 4.804619 | 2.050765 | 0.221612 | 0.935918 |
| LNC_003313 | XLOC_075161 | -- | lincRNA | 3.252758 | 1.841301 | 0.601736 | 0.789542 |
| LNC_003312 | XLOC_075160 | -- | lincRNA | 1.697086 | 0.987751 | 0.613177 | 1.030153 |
| LNC_003311 | XLOC_075159 | -- | lincRNA | 2.136932 | 1.421644 | 1.005847 | 0.667071 |
| LNC_003310 | XLOC_075158 | -- | lincRNA | 3.574842 | 2.054755 | 0 | 0.010382 |
| LNC_005469 | XLOC_126189 | -- | lincRNA | 0.1629 | 0 | 0 | 0.590849 |
| LNC_005468 | XLOC_126182 | -- | intronic_lncRNA | 0.652587 | 0 | 0 | 0 |
| LNC_000657 | XLOC_012769 | -- | intronic_lncRNA | 0.044944 | 0.152792 | 0.482904 | 0.23544 |
| LNC_000656 | XLOC_012768 | -- | intronic_lncRNA | 0.124314 | 0.619797 | 0.492656 | 0.202991 |
| LNC_000655 | XLOC_012644 | -- | intronic_lncRNA | 0.51478 | 0.171978 | 0 | 0.280377 |
| LNC_000654 | XLOC_012624 | -- | intronic_lncRNA | 0.517719 | 0.017401 | 0.05362 | 0.18607 |
| LNC_000653 | XLOC_012624 | -- | intronic_lncRNA | 0.36645 | 0.450314 | 0.594351 | 0.631898 |
| LNC_000652 | XLOC_012612 | -- | intronic_lncRNA | 0.586649 | 0.430867 | 0.712056 | 0.690486 |
| LNC_000651 | XLOC_012611 | -- | intronic_lncRNA | 0.677697 | 0.411831 | 1.355779 | 1.020781 |
| LNC_000650 | XLOC_012598 | -- | intronic_lncRNA | 0.522869 | 0.245012 | 0 | 0.216753 |
| LNC_000659 | XLOC_012789 | -- | intronic_lncRNA | 0.646317 | 0.336218 | 0.896837 | 1.217747 |
| LNC_000658 | XLOC_012769 | -- | intronic_lncRNA | 1.425408 | 0 | 0.763673 | 0.946453 |
| LNC_001658 | XLOC_036151 | -- | intronic_lncRNA | 0.568207 | 0.730076 | 0.484104 | 0.269921 |
| LNC_001659 | XLOC_036178 | -- | intronic_lncRNA | 0 | 0 | 0.566729 | 0 |
| LNC_001654 | XLOC_036143 | -- | intronic_lncRNA | 0.293284 | 0.403486 | 0.559182 | 0.704448 |
| LNC_001655 | XLOC_036148 | -- | intronic_lncRNA | 0.894206 | 0.223361 | 0.760947 | 0.334031 |

| LNC_001656 | XLOC_036149 | -- | intronic_lncRNA | 0.284949 | 0.502379 | 0.514996 | 0.4892 |
| --- | --- | --- | --- | --- | --- | --- | --- |
| LNC_001657 | XLOC_036150 | -- | intronic_lncRNA | 0.108085 | 0.271268 | 0.546775 | 0.445416 |
| LNC_001650 | XLOC_036107 | -- | intronic_lncRNA | 0.454138 | 0.175352 | 0.522835 | 0.252811 |
| LNC_001651 | XLOC_036108 | -- | intronic_lncRNA | 0.342782 | 0.352451 | 0.440123 | 0.431294 |
| LNC_001652 | XLOC_036111 | -- | intronic_lncRNA | 0.668057 | 0 | 0.698727 | 0.16881 |
| LNC_001653 | XLOC_036141 | -- | intronic_lncRNA | 0.570381 | 0.320935 | 0.78905 | 0.586967 |
| LNC_004936 | XLOC_111995 | -- | intronic_lncRNA | 0.019385 | 0.44104 | 0.007086 | 0.21939 |
| LNC_004937 | XLOC_111998 | -- | intronic_lncRNA | 0.350993 | 0.163518 | 0.803898 | 0.557246 |
| LNC_004934 | XLOC_111949 | -- | intronic_lncRNA | 0.476277 | 0.492534 | 0.406407 | 0.32244 |
| LNC_004935 | XLOC_111995 | -- | intronic_lncRNA | 0.560186 | 0.322596 | 0.551726 | 0.675841 |
| LNC_004932 | XLOC_111941 | -- | intronic_lncRNA | 0.122378 | 0 | 0.33544 | 0.581587 |
| LNC_004933 | XLOC_111948 | -- | intronic_lncRNA | 0.111788 | 0.557314 | 0.507952 | 0.345902 |
| LNC_004930 | XLOC_111937 | -- | intronic_lncRNA | 0 | 0.674106 | 0.117383 | 0.572616 |
| LNC_004931 | XLOC_111940 | -- | intronic_lncRNA | 0.584724 | 0.082985 | 0.358918 | 0.576618 |
| LNC_004938 | XLOC_111999 | -- | intronic_lncRNA | 0.859675 | 1.091621 | 1.252825 | 0.868201 |
| LNC_004939 | XLOC_112003 | -- | intronic_lncRNA | 0.472059 | 0.413218 | 0.494921 | 0.637044 |
| LNC_001269 | XLOC_027306 | -- | lincRNA | 1.455892 | 0 | 0.120149 | 0.09887 |
| LNC_001268 | XLOC_027285 | -- | intronic_lncRNA | 0.199687 | 0.492354 | 0 | 0.155747 |
| LNC_001265 | XLOC_027178 | -- | intronic_lncRNA | 0.503287 | 0 | 1.163098 | 1.700975 |
| LNC_001264 | XLOC_027175 | -- | intronic_lncRNA | 0.29331 | 0.465269 | 0.902433 | 0.557365 |
| LNC_001267 | XLOC_027208 | -- | intronic_lncRNA | 0.833041 | 0.585235 | 0.595808 | 0.278859 |
| LNC_001266 | XLOC_027185 | -- | intronic_lncRNA | 0 | 0 | 0.428961 | 0 |
| LNC_001261 | XLOC_027089 | -- | intronic_lncRNA | 0.452607 | 0.424189 | 0.17456 | 0.460807 |
| LNC_001260 | XLOC_027075 | -- | intronic_lncRNA | 0 | 0.56038 | 0 | 0 |
| LNC_001263 | XLOC_027090 | -- | intronic_lncRNA | 0.639639 | 0.249644 | 0.412283 | 0.448556 |
| LNC_001262 | XLOC_027089 | -- | intronic_lncRNA | 0.695408 | 0.441857 | 0.410341 | 0 |
| LNC_006202 | XLOC_148662 | -- | intronic_lncRNA | 4.363024 | 2.402802 | 1.809789 | 1.823196 |
| LNC_006203 | XLOC_148670 | -- | intronic_lncRNA | 0.490132 | 0.097807 | 0.205828 | 0.553843 |
| LNC_002688 | XLOC_062607 | -- | intronic_lncRNA | 0 | 0.831601 | 0 | 0.060349 |
| LNC_002689 | XLOC_062623 | -- | intronic_lncRNA | 0.511404 | 0.18386 | 0.585878 | 0.388306 |
| LNC_002682 | XLOC_062506 | -- | lincRNA | 0 | 0.100415 | 0.627958 | 0.418567 |
| LNC_002683 | XLOC_062540 | -- | intronic_lncRNA | 1.031693 | 0.044915 | 0 | 0.208725 |
| LNC_002680 | XLOC_062427 | -- | intronic_lncRNA | 0.528614 | 0.223623 | 0 | 0 |
| LNC_002681 | XLOC_062499 | -- | intronic_lncRNA | 0.076715 | 0 | 0.427077 | 0 |
| LNC_002686 | XLOC_062599 | -- | intronic_lncRNA | 0 | 0.464497 | 0.829622 | 0.603304 |
| LNC_002687 | XLOC_062606 | -- | intronic_lncRNA | 0.287771 | 0.573294 | 0.199977 | 0.214667 |
| LNC_002684 | XLOC_062555 | -- | intronic_lncRNA | 0 | 1.078922 | 0.015152 | 0.005157 |
| LNC_002685 | XLOC_062556 | -- | intronic_lncRNA | 0 | 0 | 0.401464 | 0 |
| LNC_001889 | XLOC_042526 | -- | intronic_lncRNA | 0.124009 | 0.526339 | 0 | 0.208246 |
| LNC_001888 | XLOC_042513 | -- | lincRNA | 0 | 0.506987 | 0 | 0 |
| LNC_001881 | XLOC_041963 | -- | lincRNA | 0.160144 | 0.094648 | 0.726188 | 0.20783 |
| LNC_001880 | XLOC_041896 | -- | intronic_lncRNA | 0.415171 | 0.152959 | 0.603028 | 0.382842 |
| LNC_001883 | XLOC_042296 | -- | lincRNA | 0.898218 | 0.726658 | 0.488627 | 0.40186 |
| LNC_001882 | XLOC_041966 | -- | lincRNA | 0.193226 | 0.146743 | 0.538515 | 0.881814 |
| LNC_001885 | XLOC_042496 | -- | intronic_lncRNA | 0.755294 | 0.701998 | 1.051669 | 1.12224 |
| LNC_001884 | XLOC_042350 | -- | intronic_lncRNA | 0.679796 | 0.496345 | 1.008907 | 0.827325 |
| LNC_001887 | XLOC_042500 | -- | intronic_lncRNA | 4.36368 | 5.019048 | 7.46334 | 6.269061 |
| LNC_001886 | XLOC_042497 | -- | intronic_lncRNA | 0.347028 | 0 | 1.990225 | 0.675025 |
| LNC_000132 | XLOC_001326 | -- | intronic_lncRNA | 0.187515 | 0 | 0.589582 | 0.298948 |
| LNC_005413 | XLOC_124824 | -- | lincRNA | 0.787172 | 0.371268 | 0.138112 | 0 |
| LNC_000130 | XLOC_001308 | -- | intronic_lncRNA | 0.58161 | 0 | 0.364909 | 0.151723 |
| LNC_000131 | XLOC_001315 | -- | lincRNA | 0.109135 | 0.063198 | 0.690424 | 0.609351 |
| LNC_000136 | XLOC_001332 | -- | lincRNA | 0.680546 | 0.069629 | 0.262296 | 0.724404 |
| LNC_005417 | XLOC_124931 | -- | intronic_lncRNA | 0 | 0.54488 | 0.661746 | 0.250872 |
| LNC_000134 | XLOC_001328 | -- | intronic_lncRNA | 0 | 0 | 0.053785 | 0.434333 |
| LNC_000135 | XLOC_001328 | -- | intronic_lncRNA | 0.163269 | 0.124448 | 0.728714 | 0.008873 |
| LNC_005418 | XLOC_125021 | -- | antisense_lncRNA | 0.120601 | 0.055301 | 0.466464 | 0.48717 |
| LNC_005419 | XLOC_125072 | -- | antisense_lncRNA | 0.847683 | 0 | 0.078872 | 0.112958 |
| LNC_006206 | XLOC_148704 | -- | intronic_lncRNA | 0.477568 | 0.038146 | 0 | 0.026237 |
| LNC_006207 | XLOC_148719 | -- | intronic_lncRNA | 0.912782 | 0.144275 | 0.082794 | 0.320354 |
| LNC_000415 | XLOC_007326 | -- | intronic_lncRNA | 0.887406 | 0.803488 | 0.972902 | 0.817261 |
| LNC_001469 | XLOC_031940 | -- | intronic_lncRNA | 0.721535 | 0.8043 | 0.602495 | 0.407792 |
| LNC_001468 | XLOC_031813 | -- | lincRNA | 1.012586 | 0 | 0 | 0 |
| LNC_000414 | XLOC_007236 | -- | lincRNA | 0.548393 | 0 | 0 | 0 |
| LNC_001463 | XLOC_031769 | -- | intronic_lncRNA | 0 | 0.144877 | 0.199862 | 0.523382 |
| LNC_001462 | XLOC_031738 | -- | intronic_lncRNA | 0.670138 | 0.249599 | 1.404199 | 3.251867 |
| LNC_001461 | XLOC_031681 | -- | intronic_lncRNA | 0.179637 | 0.088448 | 0.61585 | 0.586043 |
| LNC_001460 | XLOC_031657 | -- | intronic_lncRNA | 0.504812 | 0.0598 | 0.776085 | 0.526307 |
| LNC_001467 | XLOC_031791 | -- | intronic_lncRNA | 0.752893 | 1.533143 | 1.435061 | 1.058264 |
| LNC_000417 | XLOC_007389 | -- | intronic_lncRNA | 1.711501 | 0 | 0 | 0 |
| LNC_001465 | XLOC_031782 | -- | intronic_lncRNA | 0.510136 | 0.242588 | 0.548254 | 0.335703 |
| LNC_001464 | XLOC_031773 | -- | intronic_lncRNA | 0.556614 | 0 | 0.134685 | 0 |
| LNC_005176 | XLOC_118200 | -- | intronic_lncRNA | 0.27122 | 0.065032 | 0.048861 | 0.450702 |
| LNC_000411 | XLOC_007114 | -- | intronic_lncRNA | 1.079468 | 0.765167 | 0.908834 | 1.024107 |
| LNC_000410 | XLOC_007103 | -- | lincRNA | 0.796516 | 0.422105 | 0.280812 | 0.093614 |
| LNC_006211 | XLOC_148840 | -- | lincRNA | 0.656805 | 0 | 0 | 0 |
| LNC_005173 | XLOC_118097 | -- | intronic_lncRNA | 0.896088 | 0.798649 | 0.578262 | 0.896752 |

| LNC_006210 | XLOC_148800 | -- | intronic_lncRNA | 2.457555 | 0.359587 | 0.352076 | 0.567169 |
| --- | --- | --- | --- | --- | --- | --- | --- |
| LNC_005172 | XLOC_118063 | -- | intronic_lncRNA | 0.984652 | 0.291159 | 0.928343 | 0.266715 |
| LNC_006213 | XLOC_148971 | -- | intronic_lncRNA | 0.768025 | 0.051303 | 0.244478 | 0.80502 |
| LNC_004455 | XLOC_097804 | -- | intronic_lncRNA | 0.352381 | 0.324977 | 0.480724 | 0.426756 |
| LNC_004454 | XLOC_097782 | -- | intronic_lncRNA | 0 | 0.585852 | 0 | 0 |
| LNC_004457 | XLOC_097856 | -- | intronic_lncRNA | 0.543487 | 0.29647 | 0 | 0.209818 |
| LNC_004456 | XLOC_097804 | -- | intronic_lncRNA | 0.39912 | 0.194078 | 0.167465 | 0.349804 |
| LNC_004451 | XLOC_097734 | -- | intronic_lncRNA | 0.561639 | 0.360044 | 0.753356 | 0.472326 |
| LNC_004450 | XLOC_097733 | -- | intronic_lncRNA | 1.22268 | 0.557049 | 0.783756 | 0.643965 |
| LNC_004453 | XLOC_097782 | -- | antisense_lncRNA | 0.446436 | 0.175883 | 0.078363 | 0 |
| LNC_004452 | XLOC_097770 | -- | intronic_lncRNA | 2.296706 | 1.216442 | 0 | 0.673859 |
| LNC_006208 | XLOC_148797 | -- | intronic_lncRNA | 0.890289 | 1.314423 | 1.389683 | 0.40016 |
| LNC_006215 | XLOC_149015 | -- | antisense_lncRNA | 0.362436 | 0.21697 | 0 | 0.408108 |
| LNC_004459 | XLOC_097882 | -- | intronic_lncRNA | 0.650211 | 0.437731 | 0.443125 | 0.344397 |
| LNC_004458 | XLOC_097857 | -- | intronic_lncRNA | 0.359235 | 0.12554 | 0.406678 | 0.101652 |
| LNC_006214 | XLOC_148979 | -- | intronic_lncRNA | 0.754301 | 0.02466 | 1.081458 | 0.480814 |
| LNC_001799 | XLOC_040245 | -- | intronic_lncRNA | 0 | 0 | 0.984769 | 0.174153 |
| LNC_001798 | XLOC_040237 | -- | intronic_lncRNA | 0.475973 | 0.768427 | 0 | 0.41274 |
| LNC_006169 | XLOC_147810 | -- | lincRNA | 0.687344 | 0.243623 | 0.367095 | 0.27501 |
| LNC_006168 | XLOC_147775 | -- | intronic_lncRNA | 0.156285 | 0.099408 | 0.281031 | 0.507427 |
| LNC_001795 | XLOC_040224 | -- | intronic_lncRNA | 0.246257 | 0.484944 | 0 | 0.328271 |
| LNC_005179 | XLOC_118263 | -- | intronic_lncRNA | 0 | 0 | 0.149575 | 0.499608 |
| LNC_001797 | XLOC_040227 | -- | intronic_lncRNA | 0.283378 | 0 | 0.447004 | 0 |
| LNC_001796 | XLOC_040226 | -- | intronic_lncRNA | 0.1552 | 0.904245 | 0.112045 | 0.320687 |
| LNC_001791 | XLOC_039920 | -- | intronic_lncRNA | 0.282913 | 0.501586 | 0.912771 | 0.335324 |
| LNC_001790 | XLOC_039920 | -- | intronic_lncRNA | 0.397505 | 0 | 0.045299 | 0.594859 |
| LNC_001793 | XLOC_040002 | -- | intronic_lncRNA | 0 | 0.170586 | 0.414452 | 0 |
| LNC_005178 | XLOC_118253 | -- | intronic_lncRNA | 0 | 0 | 0 | 0.561386 |
| LNC_006219 | XLOC_149173 | -- | lincRNA | 0 | 0.63272 | 0.173082 | 0.055015 |
| LNC_006218 | XLOC_149045 | -- | antisense_lncRNA | 0.90299 | 0.977346 | 0.25919 | 0.425166 |
| LNC_002518 | XLOC_058452 | -- | intronic_lncRNA | 0.507904 | 0.053917 | 0.395363 | 0.682234 |
| LNC_002519 | XLOC_058456 | -- | intronic_lncRNA | 0.497261 | 0.090483 | 0.436249 | 0.805432 |
| LNC_002512 | XLOC_058209 | -- | intronic_lncRNA | 0 | 0.263624 | 0.562677 | 0.977549 |
| LNC_002513 | XLOC_058230 | -- | intronic_lncRNA | 3.239238 | 2.43884 | 1.761284 | 2.219549 |
| LNC_002510 | XLOC_058162 | -- | intronic_lncRNA | 0.457504 | 0.836656 | 0.335247 | 0.445296 |
| LNC_002511 | XLOC_058165 | -- | intronic_lncRNA | 2.356082 | 2.539476 | 4.213778 | 4.413652 |
| LNC_002516 | XLOC_058376 | -- | intronic_lncRNA | 0 | 0 | 0.466602 | 0 |
| LNC_002517 | XLOC_058427 | -- | intronic_lncRNA | 0.804328 | 0.557162 | 0.758016 | 1.009244 |
| LNC_002514 | XLOC_058268 | -- | intronic_lncRNA | 0.740991 | 0.365213 | 0.686153 | 0.452842 |
| LNC_002515 | XLOC_058316 | -- | intronic_lncRNA | 0.539829 | 0.692264 | 0.837712 | 0.818887 |
| LNC_003108 | XLOC_071488 | -- | intronic_lncRNA | 0.257644 | 0 | 0.020865 | 0.266812 |
| LNC_003109 | XLOC_071489 | -- | intronic_lncRNA | 0.618195 | 0.345756 | 0.682844 | 0.551424 |
| LNC_003102 | XLOC_071424 | -- | antisense_lncRNA | 0.484517 | 0.481722 | 0.089691 | 0 |
| LNC_003103 | XLOC_071425 | -- | intronic_lncRNA | 2.521141 | 0.198252 | 1.441983 | 0.821334 |
| LNC_003100 | XLOC_071389 | -- | intronic_lncRNA | 0.558052 | 0.226519 | 0.531726 | 0.314869 |
| LNC_003101 | XLOC_071396 | -- | intronic_lncRNA | 0.577451 | 0.27645 | 0.51675 | 0.540972 |
| LNC_003106 | XLOC_071487 | -- | intronic_lncRNA | 0.650441 | 0.09508 | 0.737707 | 0.455313 |
| LNC_003107 | XLOC_071488 | -- | intronic_lncRNA | 0.477682 | 0.229146 | 0.605622 | 0.331465 |
| LNC_003104 | XLOC_071468 | -- | intronic_lncRNA | 0.155987 | 0 | 0.633177 | 0.497199 |
| LNC_003105 | XLOC_071478 | -- | intronic_lncRNA | 1.04925 | 1.16687 | 0.936081 | 1.02924 |
| LNC_002471 | XLOC_057433 | -- | lincRNA | 4.821759 | 0.785281 | 1.2098 | 1.089137 |
| LNC_002470 | XLOC_057395 | -- | intronic_lncRNA | 0.341939 | 0.909035 | 0.424891 | 0.092085 |
| LNC_002473 | XLOC_057509 | -- | intronic_lncRNA | 0.85446 | 0.151584 | 0.845141 | 0.408966 |
| LNC_002472 | XLOC_057503 | -- | intronic_lncRNA | 0.665904 | 0.595109 | 0.603748 | 1.174787 |
| LNC_002475 | XLOC_057520 | -- | intronic_lncRNA | 0 | 0.796567 | 0.543776 | 0.290029 |
| LNC_002474 | XLOC_057510 | -- | intronic_lncRNA | 0.730903 | 0.302953 | 0.684737 | 0.778615 |
| LNC_002477 | XLOC_057523 | -- | intronic_lncRNA | 0.893286 | 0.309249 | 0.778475 | 0.737973 |
| LNC_002476 | XLOC_057520 | -- | intronic_lncRNA | 0.490239 | 0.067707 | 0.001817 | 0.118239 |
| LNC_002479 | XLOC_057536 | -- | intronic_lncRNA | 0 | 0 | 0.450849 | 0.282911 |
| LNC_002478 | XLOC_057532 | -- | intronic_lncRNA | 0.122724 | 0 | 0.249923 | 0.531659 |
| LNC_000591 | XLOC_011048 | -- | lincRNA | 0.48832 | 0.41956 | 0 | 0 |
| LNC_004213 | XLOC_090932 | -- | intronic_lncRNA | 0.2932 | 0.210772 | 0.503089 | 0.546224 |
| LNC_004212 | XLOC_090931 | -- | intronic_lncRNA | 0 | 0.51555 | 0.153154 | 0.72706 |
| LNC_004211 | XLOC_090930 | -- | intronic_lncRNA | 0 | 0 | 0.498682 | 0.279296 |
| LNC_004210 | XLOC_090922 | -- | intronic_lncRNA | 0.780977 | 0.437252 | 0.450739 | 0.699379 |
| LNC_004217 | XLOC_091247 | -- | intronic_lncRNA | 0.623592 | 0.553882 | 0.661305 | 0.472907 |
| LNC_004216 | XLOC_091116 | -- | lincRNA | 0.731447 | 0.568182 | 0.535258 | 0.891369 |
| LNC_004215 | XLOC_091049 | -- | intronic_lncRNA | 0.506576 | 0 | 0.183519 | 0 |
| LNC_004214 | XLOC_090934 | -- | lincRNA | 0 | 0.199698 | 0.230715 | 0.415263 |
| LNC_004219 | XLOC_091302 | -- | intronic_lncRNA | 0 | 1.498683 | 0.839166 | 0.777452 |
| LNC_004218 | XLOC_091247 | -- | intronic_lncRNA | 0 | 0 | 0.409633 | 0 |
| LNC_004138 | XLOC_089191 | -- | intronic_lncRNA | 0.402643 | 0.109761 | 0.241891 | 0.527912 |
| LNC_004139 | XLOC_089202 | -- | intronic_lncRNA | 1.210207 | 0.51311 | 0.243003 | 0.243997 |
| LNC_004136 | XLOC_089065 | -- | intronic_lncRNA | 1.90355 | 0.779725 | 0.656903 | 0.979322 |
| LNC_004137 | XLOC_089078 | -- | intronic_lncRNA | 0.144516 | 0.481146 | 0.066838 | 0 |
| LNC_004134 | XLOC_088955 | -- | intronic_lncRNA | 0.309921 | 0.147691 | 0.519882 | 0.845421 |
| LNC_004135 | XLOC_089050 | -- | intronic_lncRNA | 0.317529 | 0.661589 | 0.404524 | 0.62439 |

| LNC_004132 | XLOC_088933 | -- | intronic_lncRNA | 0 | 0.527267 | 0 | 0 |
| --- | --- | --- | --- | --- | --- | --- | --- |
| LNC_004133 | XLOC_088934 | -- | intronic_lncRNA | 0 | 0 | 0.391427 | 0 |
| LNC_004130 | XLOC_088925 | -- | intronic_lncRNA | 0.625066 | 0.91006 | 0.219689 | 0.832469 |
| LNC_004131 | XLOC_088926 | -- | intronic_lncRNA | 0.23049 | 0.706632 | 0.613619 | 0.424777 |
| LNC_003788 | XLOC_081105 | -- | intronic_lncRNA | 0 | 0.934722 | 0 | 0 |
| LNC_004680 | XLOC_104083 | -- | intronic_lncRNA | 1.968817 | 6.235752 | 0.013025 | 1.063404 |
| LNC_004681 | XLOC_104083 | -- | intronic_lncRNA | 1.138779 | 2.643887 | 5.647566 | 4.90436 |
| LNC_004682 | XLOC_104083 | -- | intronic_lncRNA | 3.337969 | 0 | 0 | 0 |
| LNC_004683 | XLOC_104085 | -- | intronic_lncRNA | 1.418703 | 1.273363 | 2.425489 | 1.308288 |
| LNC_004684 | XLOC_104100 | -- | intronic_lncRNA | 0.09941 | 0 | 0.95304 | 0 |
| LNC_004685 | XLOC_104105 | -- | intronic_lncRNA | 0 | 0.294413 | 0.689601 | 0.339708 |
| LNC_004686 | XLOC_104106 | -- | intronic_lncRNA | 0.295967 | 0.16538 | 0.607294 | 0.607779 |
| LNC_004687 | XLOC_104113 | -- | intronic_lncRNA | 0.84681 | 0.241193 | 0.752863 | 0.770161 |
| LNC_004688 | XLOC_104127 | -- | intronic_lncRNA | 0 | 0 | 0.598416 | 0.219968 |
| LNC_004689 | XLOC_104187 | -- | lincRNA | 0.715856 | 0 | 0 | 0 |
| LNC_003789 | XLOC_081125 | -- | antisense_lncRNA | 0.944685 | 0.463342 | 1.097825 | 0.777708 |
| LNC_003489 | XLOC_075247 | -- | lincRNA | 1.551404 | 0.725587 | 0.661693 | 0.312977 |
| LNC_003488 | XLOC_075246 | -- | lincRNA | 4.367844 | 2.175447 | 0.861317 | 1.457381 |
| LNC_003487 | XLOC_075245 | -- | lincRNA | 0.686395 | 0.360369 | 0.452784 | 0.480349 |
| LNC_003486 | XLOC_075245 | -- | lincRNA | 1.142934 | 0.604787 | 0.418883 | 1.251769 |
| LNC_003485 | XLOC_075245 | -- | lincRNA | 1.233293 | 0.586209 | 0.101697 | 0.106199 |
| LNC_003484 | XLOC_075245 | -- | lincRNA | 0.782094 | 0.683513 | 0.739017 | 1.066983 |
| LNC_003483 | XLOC_075245 | -- | lincRNA | 3.218267 | 2.447083 | 0.515417 | 0.713308 |
| LNC_003482 | XLOC_075245 | -- | lincRNA | 2.761164 | 0.133943 | 0.530181 | 1.202698 |
| LNC_003481 | XLOC_075245 | -- | lincRNA | 4.641195 | 2.425198 | 0 | 0.136974 |
| LNC_003480 | XLOC_075245 | -- | lincRNA | 1.258137 | 0.427363 | 0.662331 | 0.710323 |
| LNC_005629 | XLOC_131260 | -- | lincRNA | 0 | 0.529674 | 0.138133 | 0 |
| LNC_005628 | XLOC_131256 | -- | intronic_lncRNA | 0.7371 | 0.092063 | 0.286819 | 0.376662 |
| LNC_005621 | XLOC_131172 | -- | intronic_lncRNA | 0 | 0.251693 | 0.397496 | 0.66394 |
| LNC_005620 | XLOC_131165 | -- | intronic_lncRNA | 0.534935 | 0 | 0.230512 | 0.288118 |
| LNC_005623 | XLOC_131176 | -- | intronic_lncRNA | 0.372066 | 0.560025 | 0.652746 | 0 |
| LNC_005622 | XLOC_131172 | -- | intronic_lncRNA | 0.139668 | 0.807431 | 0.502634 | 0.032592 |
| LNC_005625 | XLOC_131182 | -- | intronic_lncRNA | 0.346671 | 1.220755 | 0.190507 | 0.193723 |
| LNC_005624 | XLOC_131178 | -- | intronic_lncRNA | 0.560468 | 0 | 0 | 0 |
| LNC_005627 | XLOC_131256 | -- | intronic_lncRNA | 0.472054 | 0.265849 | 0.845583 | 0.195456 |
| LNC_005626 | XLOC_131183 | -- | intronic_lncRNA | 0.710661 | 0.431872 | 0.645611 | 0.899209 |
| LNC_006271 | XLOC_150410 | -- | intronic_lncRNA | 0.14596 | 0.46282 | 0.305035 | 0.306349 |
| LNC_006348 | XLOC_152226 | -- | intronic_lncRNA | 1.255255 | 0 | 4.018024 | 4.96354 |
| LNC_001636 | XLOC_035663 | -- | lincRNA | 0.550506 | 0.490436 | 0.547774 | 0.475452 |
| LNC_001637 | XLOC_035786 | -- | intronic_lncRNA | 0 | 0.457202 | 0.528956 | 0.184397 |
| LNC_001634 | XLOC_035633 | -- | intronic_lncRNA | 0.296042 | 0.598729 | 0.971017 | 0.424949 |
| LNC_001635 | XLOC_035634 | -- | intronic_lncRNA | 0.471107 | 0.558039 | 0.698229 | 0.512056 |
| LNC_001632 | XLOC_035630 | -- | intronic_lncRNA | 0.431811 | 0.48806 | 0.217436 | 0.274937 |
| LNC_001633 | XLOC_035633 | -- | intronic_lncRNA | 0.052311 | 0.391064 | 0 | 0.433226 |
| LNC_001630 | XLOC_035585 | -- | intronic_lncRNA | 1.144384 | 0 | 0.033675 | 0.335334 |
| LNC_001631 | XLOC_035604 | -- | intronic_lncRNA | 0.757138 | 0.438257 | 0.421583 | 0.374338 |
| LNC_001638 | XLOC_035788 | -- | intronic_lncRNA | 0 | 0.61921 | 0 | 0 |
| LNC_001639 | XLOC_035789 | -- | intronic_lncRNA | 0 | 0.499994 | 0 | 0 |
| LNC_004918 | XLOC_111538 | -- | lincRNA | 0.947326 | 1.57859 | 0 | 0 |
| LNC_004919 | XLOC_111538 | -- | lincRNA | 0.772166 | 2.706064 | 0 | 0 |
| LNC_004910 | XLOC_111502 | -- | lincRNA | 5.021211 | 0 | 0.546178 | 0 |
| LNC_004911 | XLOC_111502 | -- | lincRNA | 0 | 5.07385 | 0 | 1.815851 |
| LNC_004912 | XLOC_111511 | -- | lincRNA | 2.42877 | 0.335511 | 0.94516 | 2.318536 |
| LNC_004913 | XLOC_111512 | -- | lincRNA | 3.543254 | 2.464653 | 0.380953 | 0.908763 |
| LNC_004914 | XLOC_111519 | -- | lincRNA | 1.077466 | 0.216508 | 0.708689 | 1.313802 |
| LNC_004915 | XLOC_111528 | -- | lincRNA | 1.503361 | 0.63822 | 0.20667 | 0.569282 |
| LNC_004916 | XLOC_111528 | -- | lincRNA | 2.31553 | 2.14921 | 0.400484 | 0.772905 |
| LNC_004917 | XLOC_111530 | -- | lincRNA | 0.275351 | 0 | 0.224743 | 0.555394 |
| LNC_001247 | XLOC_026911 | -- | intronic_lncRNA | 0.216902 | 0.401754 | 0.560617 | 0.435986 |
| LNC_001246 | XLOC_026891 | -- | intronic_lncRNA | 0.683604 | 0.426036 | 0.508185 | 0.370425 |
| LNC_001245 | XLOC_026869 | -- | intronic_lncRNA | 0.636176 | 0.188671 | 0.171399 | 0.391189 |
| LNC_001244 | XLOC_026865 | -- | intronic_lncRNA | 0.74881 | 0.178987 | 0 | 0 |
| LNC_001243 | XLOC_026829 | -- | lincRNA | 0 | 0.269916 | 0.453829 | 0 |
| LNC_001242 | XLOC_026824 | -- | intronic_lncRNA | 0.669001 | 0.829854 | 0.950413 | 0.825014 |
| LNC_001241 | XLOC_026821 | -- | intronic_lncRNA | 0 | 0.12568 | 0.419138 | 0.472115 |
| LNC_001240 | XLOC_026821 | -- | intronic_lncRNA | 0.141904 | 0.770267 | 0.347995 | 0.121822 |
| LNC_001249 | XLOC_026916 | -- | antisense_lncRNA | 0.4137 | 0.023229 | 0.379689 | 0.585631 |
| LNC_001248 | XLOC_026911 | -- | intronic_lncRNA | 0 | 0.374685 | 0 | 0 |
| LNC_006270 | XLOC_150410 | -- | intronic_lncRNA | 0.3813 | 0.162328 | 1.047677 | 1.078911 |
| LNC_001758 | XLOC_038788 | -- | intronic_lncRNA | 0.453848 | 0.377602 | 0.163909 | 0 |
| LNC_006453 | XLOC_155276 | -- | intronic_lncRNA | 0.077969 | 0 | 0 | 0 |
| LNC_005434 | XLOC_125410 | -- | antisense_lncRNA | 0.089907 | 0.072177 | 0.499357 | 0.054156 |
| LNC_000115 | XLOC_000965 | -- | lincRNA | 0 | 0.265227 | 0.597802 | 0 |
| LNC_005436 | XLOC_125465 | -- | intronic_lncRNA | 0.908433 | 2.948747 | 0.863319 | 0.862589 |
| LNC_005437 | XLOC_125479 | -- | intronic_lncRNA | 0.640676 | 0.370879 | 0.734388 | 0.653658 |
| LNC_000110 | XLOC_000947 | -- | intronic_lncRNA | 0.201132 | 0.95421 | 0.453004 | 0.577197 |
| LNC_000111 | XLOC_000947 | -- | intronic_lncRNA | 0.086919 | 0 | 0.467022 | 0.13246 |

| LNC_000112 | XLOC_000948 | -- | intronic_lncRNA | 0.19508 | 0.943426 | 0.596699 | 0.368151 |
| --- | --- | --- | --- | --- | --- | --- | --- |
| LNC_005433 | XLOC_125409 | -- | intronic_lncRNA | 0 | 0.530992 | 0.067606 | 0.159681 |
| LNC_005438 | XLOC_125499 | -- | intronic_lncRNA | 0.933053 | 0.545494 | 0.594472 | 0.690065 |
| LNC_005439 | XLOC_125526 | -- | intronic_lncRNA | 0.358947 | 0.557778 | 0.42668 | 0 |
| LNC_004708 | XLOC_104586 | -- | intronic_lncRNA | 0.310648 | 0 | 1.000711 | 0.587145 |
| LNC_006200 | XLOC_148630 | -- | intronic_lncRNA | 0.31756 | 0.201387 | 0.314773 | 0.4892 |
| LNC_000808 | XLOC_017072 | -- | intronic_lncRNA | 1.448009 | 0.596741 | 0 | 1.096988 |
| LNC_000809 | XLOC_017072 | -- | intronic_lncRNA | 0 | 0 | 1.436786 | 0.019895 |
| LNC_001409 | XLOC_030663 | -- | intronic_lncRNA | 0.229659 | 0.258961 | 0.56803 | 0.587838 |
| LNC_001408 | XLOC_030640 | -- | intronic_lncRNA | 0.787543 | 0.635344 | 0.953727 | 1.034556 |
| LNC_001405 | XLOC_030595 | -- | antisense_lncRNA | 0 | 0 | 0 | 0.43851 |
| LNC_001404 | XLOC_030595 | -- | intronic_lncRNA | 0.062171 | 0.124063 | 0.424332 | 0.657707 |
| LNC_001407 | XLOC_030627 | -- | intronic_lncRNA | 0.293332 | 0.930652 | 0.993243 | 1.347305 |
| LNC_001406 | XLOC_030599 | -- | intronic_lncRNA | 0.311687 | 0.232723 | 0.502874 | 0.062953 |
| LNC_001401 | XLOC_030553 | -- | intronic_lncRNA | 0.63717 | 0.397123 | 0.580277 | 0.660379 |
| LNC_001400 | XLOC_030552 | -- | intronic_lncRNA | 0.372035 | 0.236117 | 0.629278 | 0.114324 |
| LNC_001403 | XLOC_030581 | -- | intronic_lncRNA | 0.279058 | 0.297241 | 0.634357 | 0.437981 |
| LNC_001402 | XLOC_030561 | -- | intronic_lncRNA | 0.937145 | 1.066887 | 0.880941 | 0.983494 |
| LNC_004479 | XLOC_098374 | -- | antisense_lncRNA | 0 | 0 | 0.522619 | 0 |
| LNC_004478 | XLOC_098374 | -- | intronic_lncRNA | 0.090257 | 0.240048 | 0.612247 | 0.827915 |
| LNC_004477 | XLOC_098359 | -- | lincRNA | 0.708746 | 0.35668 | 0.114707 | 0.408102 |
| LNC_004476 | XLOC_098346 | -- | lincRNA | 2.307778 | 0.62626 | 0.469154 | 1.061231 |
| LNC_004475 | XLOC_098312 | -- | intronic_lncRNA | 0 | 0 | 0.550645 | 0 |
| LNC_004474 | XLOC_098312 | -- | intronic_lncRNA | 0.647811 | 0.133756 | 0.100182 | 0.430522 |
| LNC_004473 | XLOC_098311 | -- | intronic_lncRNA | 0 | 0 | 0 | 0.585407 |
| LNC_004472 | XLOC_098311 | -- | intronic_lncRNA | 0.66713 | 0.139254 | 0.456743 | 0.367304 |
| LNC_004471 | XLOC_098285 | -- | lincRNA | 0.632328 | 0.409677 | 0.157032 | 0.278738 |
| LNC_004470 | XLOC_098273 | -- | intronic_lncRNA | 0.520564 | 0 | 0.185303 | 0 |
| LNC_006181 | XLOC_148087 | -- | intronic_lncRNA | 0.212654 | 0.664554 | 0.579894 | 0.393178 |
| LNC_006180 | XLOC_147999 | -- | lincRNA | 0 | 0 | 0.521323 | 0 |
| LNC_006183 | XLOC_148113 | -- | intronic_lncRNA | 0.627492 | 0 | 0 | 0.057133 |
| LNC_006182 | XLOC_148107 | -- | intronic_lncRNA | 1.712868 | 2.953733 | 1.604025 | 3.36575 |
| LNC_006185 | XLOC_148155 | -- | lincRNA | 0 | 0.118715 | 0.157705 | 0.497016 |
| LNC_006184 | XLOC_148114 | -- | intronic_lncRNA | 0.488484 | 0 | 0 | 0 |
| LNC_006187 | XLOC_148158 | -- | lincRNA | 0.151266 | 1.701132 | 0.577338 | 0.479517 |
| LNC_006186 | XLOC_148156 | -- | lincRNA | 0.317687 | 0.771038 | 0.504407 | 0.154194 |
| LNC_006189 | XLOC_148220 | -- | lincRNA | 1.045259 | 1.118581 | 0.977365 | 0.890197 |
| LNC_006188 | XLOC_148198 | -- | lincRNA | 0.378831 | 0 | 0 | 0.923243 |
| LNC_003298 | XLOC_075156 | -- | lincRNA | 0 | 0.382097 | 0.500214 | 0 |
| LNC_003299 | XLOC_075157 | -- | lincRNA | 1.086581 | 0.210898 | 0.499516 | 0.246147 |
| LNC_003292 | XLOC_075154 | -- | lincRNA | 2.731899 | 0.21637 | 0.430616 | 0.524074 |
| LNC_003293 | XLOC_075154 | -- | lincRNA | 0.020627 | 0.881839 | 0 | 0.028085 |
| LNC_003290 | XLOC_075151 | -- | lincRNA | 3.457377 | 1.096076 | 0.78816 | 1.801905 |
| LNC_003291 | XLOC_075153 | -- | lincRNA | 15.742246 | 7.038282 | 3.644518 | 4.059993 |
| LNC_003296 | XLOC_075156 | -- | lincRNA | 1.955342 | 0.692156 | 0.202589 | 0.458156 |
| LNC_003297 | XLOC_075156 | -- | lincRNA | 1.410926 | 1.12777 | 0.619112 | 0.822508 |
| LNC_003294 | XLOC_075155 | -- | lincRNA | 2.97087 | 1.025349 | 1.030923 | 0.893509 |
| LNC_003295 | XLOC_075155 | -- | lincRNA | 2.484445 | 1.123623 | 0 | 0 |
| LNC_000752 | XLOC_015459 | -- | intronic_lncRNA | 0.476887 | 0 | 0.706017 | 0.585254 |
| LNC_000753 | XLOC_015526 | -- | intronic_lncRNA | 0.387013 | 0.466524 | 0.508465 | 0.819424 |
| LNC_000750 | XLOC_015391 | -- | intronic_lncRNA | 0.55088 | 0.084301 | 0.589872 | 0.395455 |
| LNC_000751 | XLOC_015392 | -- | intronic_lncRNA | 0.090123 | 0.897116 | 0.218471 | 0.495116 |
| LNC_000756 | XLOC_015654 | -- | lincRNA | 0.212966 | 0.574115 | 0.105677 | 0.093343 |
| LNC_000757 | XLOC_015690 | -- | intronic_lncRNA | 0.347191 | 0.196135 | 0.120234 | 0.44038 |
| LNC_000754 | XLOC_015530 | -- | intronic_lncRNA | 0.377798 | 0.662093 | 0.536202 | 0.535752 |
| LNC_000755 | XLOC_015576 | -- | intronic_lncRNA | 0.517882 | 0 | 0 | 0.105009 |
| LNC_005984 | XLOC_142274 | -- | intronic_lncRNA | 1.144583 | 0.047249 | 0.0302 | 0 |
| LNC_005985 | XLOC_142328 | -- | intronic_lncRNA | 0.84111 | 1.085894 | 0.594291 | 0.651824 |
| LNC_000758 | XLOC_015729 | -- | lincRNA | 0 | 0.347098 | 0.357805 | 0.518275 |
| LNC_000759 | XLOC_015741 | -- | lincRNA | 0.07819 | 1.121052 | 0 | 0.323437 |
| LNC_005980 | XLOC_142245 | -- | intronic_lncRNA | 0.488484 | 0 | 0 | 0 |
| LNC_005981 | XLOC_142249 | -- | intronic_lncRNA | 0.757632 | 0.387531 | 0.895973 | 1.10425 |
| LNC_005982 | XLOC_142273 | -- | intronic_lncRNA | 0.28319 | 0.347351 | 0.424809 | 0.178161 |
| LNC_005983 | XLOC_142274 | -- | intronic_lncRNA | 0.687771 | 0.207968 | 0.610097 | 0.60667 |
| LNC_002538 | XLOC_058717 | -- | intronic_lncRNA | 0.128427 | 0.017094 | 0 | 0.695386 |
| LNC_002539 | XLOC_058850 | -- | intronic_lncRNA | 0.51389 | 1.167555 | 0.581799 | 1.54933 |
| LNC_002534 | XLOC_058688 | -- | intronic_lncRNA | 0.29658 | 0.034462 | 1.408339 | 0.337847 |
| LNC_002535 | XLOC_058691 | -- | intronic_lncRNA | 0.201034 | 0.136507 | 1.286433 | 0.618542 |
| LNC_002536 | XLOC_058691 | -- | intronic_lncRNA | 0.288882 | 0.31995 | 0.749587 | 0.770538 |
| LNC_002537 | XLOC_058717 | -- | intronic_lncRNA | 0 | 1.329382 | 0 | 0 |
| LNC_002530 | XLOC_058660 | -- | intronic_lncRNA | 0.518194 | 0.226468 | 0.454578 | 0.295254 |
| LNC_002531 | XLOC_058661 | -- | intronic_lncRNA | 0.867627 | 0.251645 | 0.479208 | 0.786435 |
| LNC_002532 | XLOC_058671 | -- | intronic_lncRNA | 2.736623 | 3.261166 | 1.941399 | 2.033226 |
| LNC_002533 | XLOC_058686 | -- | intronic_lncRNA | 1.734347 | 1.152809 | 1.778552 | 2.423092 |
| LNC_003128 | XLOC_072221 | -- | lincRNA | 0.261147 | 0.182532 | 0.422883 | 0.056624 |
| LNC_003129 | XLOC_072229 | -- | intronic_lncRNA | 0.648636 | 0.331172 | 0.557373 | 0.345266 |
| LNC_003124 | XLOC_072038 | -- | intronic_lncRNA | 0.34272 | 0 | 0.067195 | 0.30102 |

| LNC_003125 | XLOC_072041 | -- | intronic_lncRNA | 1.205247 | 0.371045 | 0.419536 | 1.227906 |
| --- | --- | --- | --- | --- | --- | --- | --- |
| LNC_003126 | XLOC_072100 | -- | intronic_lncRNA | 0.122149 | 0.664973 | 0.261665 | 1.996465 |
| LNC_003127 | XLOC_072100 | -- | intronic_lncRNA | 1.927174 | 0.929505 | 1.458965 | 0 |
| LNC_003120 | XLOC_071775 | -- | intronic_lncRNA | 0.838304 | 0.288119 | 0.641117 | 0.328322 |
| LNC_003121 | XLOC_071795 | -- | intronic_lncRNA | 0.746107 | 0.354298 | 0.343446 | 0.331802 |
| LNC_003122 | XLOC_071799 | -- | intronic_lncRNA | 10.9182 | 10.585018 | 11.852163 | 13.378634 |
| LNC_003123 | XLOC_071992 | -- | intronic_lncRNA | 0.327491 | 0.735203 | 0.151346 | 0.246061 |
| LNC_002131 | XLOC_048612 | -- | intronic_lncRNA | 3.233752 | 2.084068 | 3.899559 | 3.340714 |
| LNC_005184 | XLOC_118322 | -- | intronic_lncRNA | 0 | 0 | 0.11603 | 0.745447 |
| LNC_005185 | XLOC_118341 | -- | intronic_lncRNA | 0.747869 | 0.66153 | 0.383202 | 0.486425 |
| LNC_005186 | XLOC_118346 | -- | intronic_lncRNA | 0.135028 | 0.712473 | 1.499153 | 0.668175 |
| LNC_005187 | XLOC_118395 | -- | intronic_lncRNA | 0.280143 | 0.465625 | 0.732299 | 1.044695 |
| LNC_005180 | XLOC_118276 | -- | intronic_lncRNA | 0 | 0 | 0.733926 | 0 |
| LNC_005181 | XLOC_118318 | -- | antisense_lncRNA | 0.682641 | 0.22076 | 0.092902 | 0.235618 |
| LNC_005182 | XLOC_118320 | -- | intronic_lncRNA | 0.39794 | 0.446379 | 0.234209 | 0.573617 |
| LNC_005183 | XLOC_118321 | -- | intronic_lncRNA | 0.078326 | 0.043939 | 0.129198 | 0.55075 |
| LNC_002453 | XLOC_056907 | -- | intronic_lncRNA | 0 | 0.596577 | 0.152605 | 0 |
| LNC_002452 | XLOC_056907 | -- | intronic_lncRNA | 0.731952 | 0.53074 | 0.455912 | 0.465778 |
| LNC_002451 | XLOC_056890 | -- | intronic_lncRNA | 0.989745 | 0.39545 | 0.649057 | 0.421779 |
| LNC_002450 | XLOC_056887 | -- | lincRNA | 0.701001 | 1.032044 | 0.792757 | 0.578682 |
| LNC_005188 | XLOC_118397 | -- | intronic_lncRNA | 0.55729 | 0.001212 | 0.035015 | 0.036936 |
| LNC_005189 | XLOC_118422 | -- | intronic_lncRNA | 0.391766 | 0 | 0.137788 | 0.898203 |
| LNC_002455 | XLOC_056979 | -- | intronic_lncRNA | 1.698702 | 0.920634 | 1.701007 | 2.655915 |
| LNC_002454 | XLOC_056941 | -- | intronic_lncRNA | 0.139323 | 0.0832 | 0.570146 | 0.100725 |
| LNC_002137 | XLOC_048707 | -- | lincRNA | 0.512155 | 0 | 0 | 0.206886 |
| LNC_002989 | XLOC_068330 | -- | intronic_lncRNA | 0.286849 | 0 | 0.205521 | 0.530766 |
| LNC_002988 | XLOC_068326 | -- | intronic_lncRNA | 0.579798 | 0.224058 | 0.564812 | 0.60802 |
| LNC_002985 | XLOC_068185 | -- | intronic_lncRNA | 0.771858 | 0.40622 | 1.027952 | 0.801454 |
| LNC_002984 | XLOC_068165 | -- | lincRNA | 0 | 0 | 0.535794 | 0 |
| LNC_002987 | XLOC_068210 | -- | intronic_lncRNA | 2.640343 | 1.819357 | 1.829572 | 1.628665 |
| LNC_002986 | XLOC_068204 | -- | lincRNA | 0 | 0 | 0.138604 | 0.735671 |
| LNC_002981 | XLOC_068086 | -- | intronic_lncRNA | 0.011163 | 0.485659 | 0.397261 | 5.744034 |
| LNC_002980 | XLOC_068086 | -- | intronic_lncRNA | 0 | 0 | 0.638979 | 0 |
| LNC_002983 | XLOC_068123 | -- | intronic_lncRNA | 0.954175 | 0.045051 | 0.036243 | 0.289896 |
| LNC_002982 | XLOC_068086 | -- | intronic_lncRNA | 0 | 0.206037 | 11.543465 | 2.230933 |
| LNC_005143 | XLOC_117487 | -- | intronic_lncRNA | 0.849367 | 0 | 0.349672 | 0.172988 |
| LNC_003523 | XLOC_075263 | -- | lincRNA | 2.087442 | 0.327183 | 0.134852 | 0.086444 |
| LNC_003176 | XLOC_073636 | -- | antisense_lncRNA | 0.47019 | 0.6021 | 0 | 0.390546 |
| LNC_003529 | XLOC_075266 | -- | lincRNA | 13.971118 | 0.135288 | 3.145628 | 4.089098 |
| LNC_001188 | XLOC_025824 | -- | intronic_lncRNA | 0.335904 | 0 | 0.321727 | 0.76556 |
| LNC_001189 | XLOC_025834 | -- | lincRNA | 0 | 0 | 0.475885 | 0.000102 |
| LNC_004277 | XLOC_092939 | -- | intronic_lncRNA | 1.159173 | 0.130103 | 0.345305 | 0.266485 |
| LNC_004276 | XLOC_092938 | -- | intronic_lncRNA | 0.079619 | 0 | 0.483602 | 0.499089 |
| LNC_004271 | XLOC_092842 | -- | intronic_lncRNA | 0.165314 | 0.170461 | 0.567268 | 0.4311 |
| LNC_004270 | XLOC_092828 | -- | intronic_lncRNA | 0.061683 | 0 | 0.486676 | 0.122866 |
| LNC_004273 | XLOC_092884 | -- | intronic_lncRNA | 0.337743 | 0.255399 | 0.177799 | 1.048561 |
| LNC_004272 | XLOC_092879 | -- | intronic_lncRNA | 0 | 0.728025 | 0 | 0.284268 |
| LNC_001180 | XLOC_025651 | -- | intronic_lncRNA | 0 | 0.254719 | 0 | 0 |
| LNC_001181 | XLOC_025651 | -- | intronic_lncRNA | 0.849955 | 0.370378 | 0.884097 | 0.6933 |
| LNC_001182 | XLOC_025651 | -- | intronic_lncRNA | 0.880102 | 0.3776 | 0.723672 | 0.208075 |
| LNC_001183 | XLOC_025704 | -- | intronic_lncRNA | 0.379256 | 0.618522 | 0.720078 | 0.647902 |
| LNC_001184 | XLOC_025742 | -- | intronic_lncRNA | 0 | 3.53132 | 0 | 0 |
| LNC_001185 | XLOC_025749 | -- | intronic_lncRNA | 0.631685 | 0.489434 | 0.981713 | 0.621727 |
| LNC_001186 | XLOC_025792 | -- | intronic_lncRNA | 0.623932 | 0.570655 | 0.144246 | 0.32597 |
| LNC_001187 | XLOC_025805 | -- | intronic_lncRNA | 3.106733 | 3.74034 | 4.38571 | 4.451941 |
| LNC_004110 | XLOC_088665 | -- | intronic_lncRNA | 0.094061 | 0.37102 | 0.568266 | 0.565382 |
| LNC_004111 | XLOC_088671 | -- | intronic_lncRNA | 2.090823 | 1.57694 | 2.340604 | 2.221628 |
| LNC_004112 | XLOC_088679 | -- | lincRNA | 0.09097 | 0.174985 | 0.543955 | 0 |
| LNC_004113 | XLOC_088685 | -- | intronic_lncRNA | 0.555216 | 0.325476 | 0.665017 | 0.615024 |
| LNC_004114 | XLOC_088702 | -- | lincRNA | 0.958275 | 0.108558 | 0.155796 | 0.387227 |
| LNC_004115 | XLOC_088715 | -- | intronic_lncRNA | 0.501117 | 0.120228 | 0 | 0 |
| LNC_004116 | XLOC_088716 | -- | intronic_lncRNA | 0 | 0.919712 | 0 | 0.058423 |
| LNC_004117 | XLOC_088721 | -- | intronic_lncRNA | 0.448543 | 0.04988 | 0.618515 | 0.920741 |
| LNC_004118 | XLOC_088742 | -- | intronic_lncRNA | 0 | 0.786063 | 0 | 0 |
| LNC_004119 | XLOC_088767 | -- | intronic_lncRNA | 0.36801 | 0.054104 | 0.657247 | 0.5693 |
| LNC_005787 | XLOC_136122 | -- | intronic_lncRNA | 0.662648 | 0.110686 | 0.426387 | 0 |
| LNC_001980 | XLOC_044828 | -- | intronic_lncRNA | 0.203888 | 0.649246 | 0 | 0 |
| LNC_001981 | XLOC_044835 | -- | intronic_lncRNA | 8.616655 | 4.18922 | 7.966026 | 8.777158 |
| LNC_001982 | XLOC_044838 | -- | intronic_lncRNA | 0.576628 | 0.440179 | 1.104598 | 0.47011 |
| LNC_001983 | XLOC_044839 | -- | intronic_lncRNA | 0.442775 | 0.394066 | 0.399755 | 0.08127 |
| LNC_001984 | XLOC_044841 | -- | intronic_lncRNA | 0.382989 | 0.544842 | 0.785905 | 0.296146 |
| LNC_001985 | XLOC_044844 | -- | intronic_lncRNA | 0.718332 | 0.573929 | 0.832862 | 0.267851 |
| LNC_001986 | XLOC_044845 | -- | intronic_lncRNA | 0.950182 | 0.054716 | 0.86631 | 0.24393 |
| LNC_001987 | XLOC_044849 | -- | lincRNA | 0.52716 | 0.357761 | 0 | 1.145414 |
| LNC_001988 | XLOC_044854 | -- | intronic_lncRNA | 0.540138 | 0.209805 | 0.504194 | 0.711185 |
| LNC_001989 | XLOC_044854 | -- | intronic_lncRNA | 0.655485 | 0.025082 | 0.579023 | 0.916347 |
| LNC_005335 | XLOC_122121 | -- | lincRNA | 0.993351 | 0.479883 | 0.146621 | 0.178567 |

| LNC_005334 | XLOC_122085 | -- | intronic_lncRNA | 0 | 0.191744 | 0.693385 | 0.149861 |
| --- | --- | --- | --- | --- | --- | --- | --- |
| LNC_005333 | XLOC_122081 | -- | intronic_lncRNA | 0.553483 | 0 | 0 | 0.092878 |
| LNC_005332 | XLOC_122080 | -- | intronic_lncRNA | 1.114306 | 0 | 0 | 0.119033 |
| LNC_005789 | XLOC_136145 | -- | intronic_lncRNA | 0 | 0.738745 | 0 | 0 |
| LNC_005331 | XLOC_122064 | -- | intronic_lncRNA | 0.20453 | 0.225514 | 0.456665 | 0 |
| LNC_005330 | XLOC_121933 | -- | intronic_lncRNA | 0.367239 | 0 | 0.28931 | 0.169887 |
| LNC_003728 | XLOC_079950 | -- | intronic_lncRNA | 0.503283 | 0.870025 | 0.302941 | 0.085641 |
| LNC_003729 | XLOC_080028 | -- | intronic_lncRNA | 0.781125 | 0.306982 | 0.266816 | 0.225133 |
| LNC_003722 | XLOC_079904 | -- | intronic_lncRNA | 0 | 0.705975 | 0 | 0 |
| LNC_003723 | XLOC_079915 | -- | intronic_lncRNA | 0.293091 | 0.487389 | 0.515763 | 0.328874 |
| LNC_003720 | XLOC_079750 | -- | intronic_lncRNA | 0.438714 | 0.656596 | 0.460259 | 0.128766 |
| LNC_003721 | XLOC_079842 | -- | intronic_lncRNA | 0.539641 | 0.420155 | 0.314929 | 0.403398 |
| LNC_003726 | XLOC_079927 | -- | intronic_lncRNA | 0.556264 | 0.134129 | 0.180543 | 0 |
| LNC_003727 | XLOC_079950 | -- | intronic_lncRNA | 0.380382 | 0.212713 | 0.320624 | 0.617292 |
| LNC_003724 | XLOC_079916 | -- | intronic_lncRNA | 0.635922 | 0.477605 | 0.364892 | 0.575854 |
| LNC_003725 | XLOC_079917 | -- | intronic_lncRNA | 0.597119 | 0.500589 | 0.532448 | 0.486369 |
| LNC_005339 | XLOC_122158 | -- | intronic_lncRNA | 0.343158 | 0.176924 | 0.497371 | 0.561441 |
| LNC_005338 | XLOC_122157 | -- | intronic_lncRNA | 0.16507 | 0.527833 | 0.147388 | 0.06932 |
| LNC_003461 | XLOC_075234 | -- | lincRNA | 0.17732 | 0.104369 | 1.91305 | 3.538401 |
| LNC_003460 | XLOC_075234 | -- | lincRNA | 6.494428 | 5.848358 | 1.031133 | 0 |
| LNC_003463 | XLOC_075235 | -- | lincRNA | 4.260773 | 2.558174 | 1.760705 | 1.013452 |
| LNC_003462 | XLOC_075234 | -- | lincRNA | 4.506225 | 0.00972 | 0.055106 | 0.04378 |
| LNC_003465 | XLOC_075236 | -- | lincRNA | 10.54071 | 3.970328 | 2.744715 | 3.113763 |
| LNC_003464 | XLOC_075235 | -- | lincRNA | 5.32887 | 0 | 0 | 0.994838 |
| LNC_003467 | XLOC_075239 | -- | lincRNA | 5.213204 | 3.240871 | 1.100873 | 1.185534 |
| LNC_003466 | XLOC_075237 | -- | lincRNA | 1.806442 | 2.033056 | 0.86186 | 0.639523 |
| LNC_003469 | XLOC_075241 | -- | lincRNA | 2.030237 | 0.283444 | 0.551241 | 0 |
| LNC_003468 | XLOC_075240 | -- | lincRNA | 1.889213 | 0.679934 | 0.243526 | 0.042573 |
| LNC_005603 | XLOC_130660 | -- | lincRNA | 0.295627 | 0.300675 | 0.258003 | 0.409563 |
| LNC_005602 | XLOC_130630 | -- | lincRNA | 0.830006 | 0.395043 | 0.618884 | 0.571161 |
| LNC_005601 | XLOC_130550 | -- | intronic_lncRNA | 0.56124 | 0.097636 | 0.329371 | 0.028954 |
| LNC_005600 | XLOC_130491 | -- | intronic_lncRNA | 0.040706 | 0.652648 | 1.093161 | 0.617642 |
| LNC_005607 | XLOC_130770 | -- | lincRNA | 0.870968 | 0.58844 | 5.504631 | 7.138852 |
| LNC_005606 | XLOC_130761 | -- | lincRNA | 0.084002 | 0.241664 | 0.461782 | 0.992651 |
| LNC_005605 | XLOC_130714 | -- | lincRNA | 0.290239 | 0.935657 | 0.185521 | 0.396314 |
| LNC_005604 | XLOC_130665 | -- | intronic_lncRNA | 0.550673 | 0.660142 | 0.387699 | 0.759479 |
| LNC_005609 | XLOC_130849 | -- | intronic_lncRNA | 0.482234 | 0.131055 | 0.037536 | 0.139668 |
| LNC_005608 | XLOC_130839 | -- | intronic_lncRNA | 0.800802 | 0 | 0.493401 | 0.876877 |
| LNC_006281 | XLOC_150612 | -- | intronic_lncRNA | 2.518393 | 2.556492 | 2.150861 | 2.650685 |
| LNC_001618 | XLOC_035452 | -- | lincRNA | 0.530864 | 0 | 0.106525 | 0 |
| LNC_001619 | XLOC_035460 | -- | intronic_lncRNA | 0.718918 | 0.40991 | 0.321146 | 0.480984 |
| LNC_001610 | XLOC_035075 | -- | intronic_lncRNA | 0 | 0.676259 | 0 | 0.135354 |
| LNC_001611 | XLOC_035139 | -- | antisense_lncRNA | 0.516708 | 0.143495 | 0 | 0 |
| LNC_001612 | XLOC_035165 | -- | intronic_lncRNA | 0.767139 | 0 | 0 | 0.06155 |
| LNC_001613 | XLOC_035184 | -- | intronic_lncRNA | 0.361727 | 0.236624 | 0.840047 | 0.477224 |
| LNC_001614 | XLOC_035194 | -- | intronic_lncRNA | 1.166183 | 0.85591 | 0.690648 | 0.748136 |
| LNC_001615 | XLOC_035229 | -- | lincRNA | 0.102373 | 0.80372 | 0 | 0.128784 |
| LNC_001616 | XLOC_035344 | -- | intronic_lncRNA | 0.789375 | 1.016229 | 1.387357 | 0.912092 |
| LNC_001617 | XLOC_035390 | -- | intronic_lncRNA | 0.857884 | 0.566242 | 0.564398 | 0.613599 |
| LNC_006279 | XLOC_150585 | -- | intronic_lncRNA | 0.246902 | 0.259621 | 0.716193 | 0.805667 |
| LNC_000569 | XLOC_010591 | -- | intronic_lncRNA | 0.438706 | 0.232839 | 0.641415 | 0.7095 |
| LNC_000568 | XLOC_010571 | -- | intronic_lncRNA | 0.899574 | 0.402172 | 1.163228 | 0.485696 |
| LNC_000561 | XLOC_010478 | -- | intronic_lncRNA | 0.947625 | 0 | 0.255956 | 0.36092 |
| LNC_000560 | XLOC_010475 | -- | intronic_lncRNA | 0.262802 | 0.082867 | 0.510214 | 0.148149 |
| LNC_000563 | XLOC_010491 | -- | intronic_lncRNA | 0 | 0.592221 | 0.501189 | 0.496719 |
| LNC_000562 | XLOC_010491 | -- | intronic_lncRNA | 0.536212 | 0 | 0.218786 | 0 |
| LNC_000565 | XLOC_010524 | -- | intronic_lncRNA | 0.353587 | 0.17647 | 0.503236 | 0 |
| LNC_000564 | XLOC_010492 | -- | intronic_lncRNA | 0.504735 | 0.131203 | 0.349052 | 0.637439 |
| LNC_000567 | XLOC_010568 | -- | intronic_lncRNA | 0.225881 | 0.158531 | 0.371964 | 0.621505 |
| LNC_000566 | XLOC_010564 | -- | intronic_lncRNA | 0.456472 | 0.208862 | 1.221248 | 0.46744 |
| LNC_003911 | XLOC_084029 | -- | lincRNA | 0.461496 | 0.903011 | 0.404136 | 0.62847 |
| LNC_003910 | XLOC_084008 | -- | lincRNA | 0.7591 | 0.476795 | 0.646601 | 0.631488 |
| LNC_003913 | XLOC_084178 | -- | intronic_lncRNA | 0.67704 | 0.234054 | 0.559922 | 0.351679 |
| LNC_003912 | XLOC_084030 | -- | lincRNA | 0.521997 | 0.612415 | 0.372926 | 0.405646 |
| LNC_003915 | XLOC_084383 | -- | intronic_lncRNA | 0.376326 | 0.653843 | 0.89542 | 0.433899 |
| LNC_003914 | XLOC_084179 | -- | intronic_lncRNA | 0.670767 | 0.657791 | 0.665138 | 0.614086 |
| LNC_003917 | XLOC_084505 | -- | intronic_lncRNA | 0 | 0.483874 | 0.132687 | 0.106993 |
| LNC_003916 | XLOC_084499 | -- | lincRNA | 0.636167 | 0.602105 | 0.961738 | 0.871093 |
| LNC_003919 | XLOC_084509 | -- | intronic_lncRNA | 0.92208 | 0.523323 | 0.402098 | 0.733811 |
| LNC_003918 | XLOC_084506 | -- | intronic_lncRNA | 0.352728 | 0.422325 | 1.062174 | 0.589319 |
| LNC_000178 | XLOC_002191 | -- | intronic_lncRNA | 0.005822 | 0.030186 | 0.583362 | 0.960917 |
| LNC_000179 | XLOC_002242 | -- | intronic_lncRNA | 1.27768 | 0 | 0.006728 | 0 |
| LNC_000176 | XLOC_002115 | -- | intronic_lncRNA | 0 | 0 | 0.443125 | 0.12135 |
| LNC_000177 | XLOC_002191 | -- | intronic_lncRNA | 0.549887 | 0.484531 | 1.663144 | 1.450222 |
| LNC_000174 | XLOC_002101 | -- | intronic_lncRNA | 0.37344 | 0.265941 | 0.8462 | 0.589848 |
| LNC_000175 | XLOC_002102 | -- | intronic_lncRNA | 0.55712 | 0.247954 | 0.572088 | 0.563271 |
| LNC_000172 | XLOC_002031 | -- | intronic_lncRNA | 0 | 0 | 0.655087 | 0 |

| LNC_000173 | XLOC_002056 | -- | intronic_lncRNA | 1.585343 | 1.190528 | 0.998874 | 1.055752 |
| --- | --- | --- | --- | --- | --- | --- | --- |
| LNC_000170 | XLOC_002021 | -- | intronic_lncRNA | 0.770239 | 0.605539 | 0.497254 | 0.636913 |
| LNC_000171 | XLOC_002028 | -- | intronic_lncRNA | 0 | 0.139576 | 0.453674 | 0 |
| LNC_000525 | XLOC_009824 | -- | intronic_lncRNA | 0.350006 | 0.179874 | 0.330007 | 0.6566 |
| LNC_000524 | XLOC_009787 | -- | intronic_lncRNA | 0.051921 | 0.463108 | 0.31779 | 0.55646 |
| LNC_005007 | XLOC_114292 | -- | intronic_lncRNA | 0.118793 | 1.265277 | 0.45162 | 0.735424 |
| LNC_000526 | XLOC_009846 | -- | intronic_lncRNA | 0.358825 | 0 | 0 | 0.712178 |
| LNC_005001 | XLOC_113988 | -- | intronic_lncRNA | 0.514329 | 0.432276 | 0.649015 | 0.941691 |
| LNC_005000 | XLOC_113988 | -- | intronic_lncRNA | 0.376123 | 1.033248 | 0.126215 | 0.07067 |
| LNC_006321 | XLOC_151799 | -- | intronic_lncRNA | 2.287946 | 2.878354 | 6.998999 | 8.338118 |
| LNC_000523 | XLOC_009244 | -- | intronic_lncRNA | 0.317071 | 0 | 0.610017 | 0 |
| LNC_006320 | XLOC_151763 | -- | lincRNA | 0.081187 | 0.648037 | 0.098404 | 0.253181 |
| LNC_000522 | XLOC_009217 | -- | intronic_lncRNA | 0.461852 | 0 | 0 | 0.428887 |
| LNC_006323 | XLOC_151799 | -- | intronic_lncRNA | 0.596251 | 0.848427 | 1.919387 | 1.248821 |
| LNC_006322 | XLOC_151799 | -- | intronic_lncRNA | 2.920643 | 0.945946 | 1.052467 | 1.236321 |
| LNC_006325 | XLOC_151885 | -- | intronic_lncRNA | 0.643132 | 0.494301 | 0.132318 | 0.636271 |
| LNC_006324 | XLOC_151885 | -- | intronic_lncRNA | 0.222937 | 0.016047 | 0.363646 | 0.051836 |
| LNC_006327 | XLOC_151979 | -- | intronic_lncRNA | 2.860219 | 3.45387 | 12.394024 | 11.104055 |
| LNC_000529 | XLOC_009897 | -- | intronic_lncRNA | 0.405002 | 0.578726 | 0.644984 | 0.640474 |
| LNC_006326 | XLOC_151974 | -- | intronic_lncRNA | 0.33755 | 0.429476 | 0.793491 | 0.612551 |
| LNC_000528 | XLOC_009854 | -- | intronic_lncRNA | 1.328573 | 0.326693 | 1.091152 | 1.118355 |
| LNC_006329 | XLOC_152014 | -- | intronic_lncRNA | 0.337368 | 0.187903 | 0.621042 | 0.253581 |
| LNC_006328 | XLOC_152012 | -- | intronic_lncRNA | 0.195276 | 0.854763 | 0.124006 | 0.16541 |
| LNC_001427 | XLOC_031124 | -- | intronic_lncRNA | 0.227318 | 0 | 0 | 0.406608 |
| LNC_001426 | XLOC_031085 | -- | intronic_lncRNA | 0 | 0.486183 | 0.355092 | 0.38492 |
| LNC_001425 | XLOC_031013 | -- | intronic_lncRNA | 1.050732 | 0.786858 | 1.126024 | 1.495439 |
| LNC_001424 | XLOC_031004 | -- | intronic_lncRNA | 0 | 0 | 0.234856 | 1.251829 |
| LNC_000828 | XLOC_017577 | -- | lincRNA | 0.490651 | 0 | 0 | 0 |
| LNC_000829 | XLOC_017592 | -- | intronic_lncRNA | 0.670124 | 0.520763 | 0.870347 | 0.8084 |
| LNC_001421 | XLOC_030955 | -- | intronic_lncRNA | 0.440701 | 0.497356 | 0.388122 | 0.459719 |
| LNC_001420 | XLOC_030790 | -- | lincRNA | 0 | 0.164481 | 0.49923 | 0 |
| LNC_000824 | XLOC_017426 | -- | intronic_lncRNA | 2.67109 | 0.147447 | 0.425828 | 0 |
| LNC_000825 | XLOC_017553 | -- | intronic_lncRNA | 0.01337 | 0.110269 | 0.771069 | 0.388391 |
| LNC_000826 | XLOC_017553 | -- | antisense_lncRNA | 0 | 0.543401 | 0.275287 | 0.347219 |
| LNC_000827 | XLOC_017560 | -- | intronic_lncRNA | 0.210731 | 0.400917 | 0.66717 | 0.357093 |
| LNC_000820 | XLOC_017315 | -- | intronic_lncRNA | 0.515847 | 0.490631 | 0.702829 | 0.420543 |
| LNC_000821 | XLOC_017319 | -- | intronic_lncRNA | 0.298957 | 0.063108 | 0.666625 | 0.677644 |
| LNC_000822 | XLOC_017404 | -- | intronic_lncRNA | 0 | 0.115046 | 0.438984 | 0.181415 |
| LNC_000823 | XLOC_017423 | -- | intronic_lncRNA | 0 | 0.527655 | 0.086503 | 0 |
| LNC_004491 | XLOC_098490 | -- | intronic_lncRNA | 0 | 0 | 0 | 0.452082 |
| LNC_004490 | XLOC_098474 | -- | intronic_lncRNA | 0.335407 | 0.678355 | 0.274686 | 0.563009 |
| LNC_004493 | XLOC_098496 | -- | lincRNA | 0.274993 | 1.095386 | 0.680417 | 0.783119 |
| LNC_004492 | XLOC_098492 | -- | intronic_lncRNA | 0.326986 | 0.341337 | 0.527773 | 0.673263 |
| LNC_004495 | XLOC_098601 | -- | intronic_lncRNA | 0.533299 | 0 | 0 | 0 |
| LNC_004494 | XLOC_098548 | -- | intronic_lncRNA | 0.388353 | 0.596633 | 0.462198 | 1.016333 |
| LNC_004497 | XLOC_098639 | -- | lincRNA | 0.984718 | 0.727634 | 0.165683 | 0.103534 |
| LNC_004496 | XLOC_098605 | -- | intronic_lncRNA | 2.192735 | 1.088462 | 2.434305 | 1.358112 |
| LNC_004499 | XLOC_098693 | -- | lincRNA | 0.661079 | 0.350957 | 0.108544 | 0.131778 |
| LNC_004498 | XLOC_098672 | -- | lincRNA | 1.612677 | 0.686781 | 0.646212 | 0.589898 |
| LNC_002088 | XLOC_047515 | -- | intronic_lncRNA | 0.655597 | 0.433178 | 0.168343 | 0.526129 |
| LNC_002089 | XLOC_047517 | -- | lincRNA | 0 | 0 | 0 | 0.413166 |
| LNC_002084 | XLOC_047026 | -- | intronic_lncRNA | 0.656323 | 0.047006 | 0.214989 | 0.605343 |
| LNC_002085 | XLOC_047026 | -- | intronic_lncRNA | 0.128836 | 0.267513 | 0.140781 | 0.151129 |
| LNC_002086 | XLOC_047031 | -- | intronic_lncRNA | 0.847193 | 0.343685 | 0.597274 | 0.691749 |
| LNC_002087 | XLOC_047514 | -- | intronic_lncRNA | 1.330479 | 1.050049 | 1.130999 | 1.470061 |
| LNC_002080 | XLOC_046969 | -- | lincRNA | 1.016974 | 0 | 0.550194 | 0.259003 |
| LNC_002081 | XLOC_046971 | -- | lincRNA | 0.378574 | 0 | 0.217624 | 0.705596 |
| LNC_002082 | XLOC_046971 | -- | lincRNA | 0.33441 | 0.410829 | 0.096003 | 0.461775 |
| LNC_002083 | XLOC_046979 | -- | lincRNA | 0 | 0 | 0 | 0.426938 |
| LNC_000774 | XLOC_016305 | -- | intronic_lncRNA | 0.639426 | 0.211423 | 0.325208 | 0.298151 |
| LNC_000775 | XLOC_016306 | -- | intronic_lncRNA | 0.470984 | 0.317273 | 0.248504 | 0.599052 |
| LNC_000776 | XLOC_016307 | -- | intronic_lncRNA | 0.720104 | 0.418514 | 0.593794 | 0.773578 |
| LNC_000777 | XLOC_016320 | -- | intronic_lncRNA | 0.550428 | 0.270076 | 0.493503 | 0.248877 |
| LNC_000770 | XLOC_016114 | -- | intronic_lncRNA | 1.011601 | 0.498557 | 0.988593 | 1.177081 |
| LNC_000771 | XLOC_016122 | -- | intronic_lncRNA | 0.574926 | 0.437419 | 0.888211 | 0.672215 |
| LNC_000772 | XLOC_016123 | -- | intronic_lncRNA | 0.065708 | 0.309617 | 0.425532 | 0.52233 |
| LNC_000773 | XLOC_016124 | -- | intronic_lncRNA | 0.924074 | 0.22081 | 0.325063 | 0.321431 |
| LNC_000778 | XLOC_016326 | -- | lincRNA | 0.080068 | 0.116559 | 0.700903 | 0.25336 |
| LNC_000779 | XLOC_016341 | -- | intronic_lncRNA | 1.312129 | 0.291136 | 0.441915 | 0.514591 |
| LNC_002556 | XLOC_059128 | -- | intronic_lncRNA | 0.372432 | 0.499229 | 0.105819 | 0.197679 |
| LNC_002557 | XLOC_059135 | -- | intronic_lncRNA | 0 | 0 | 0.42089 | 0.36583 |
| LNC_002554 | XLOC_059023 | -- | lincRNA | 0 | 0 | 0.132022 | 0.435516 |
| LNC_002555 | XLOC_059057 | -- | intronic_lncRNA | 0.713613 | 0 | 0.115754 | 0.026146 |
| LNC_002552 | XLOC_059000 | -- | intronic_lncRNA | 0 | 0 | 0 | 0.996664 |
| LNC_002553 | XLOC_059012 | -- | lincRNA | 0.699121 | 0.763951 | 1.024037 | 0.988599 |
| LNC_002550 | XLOC_058999 | -- | intronic_lncRNA | 0.657033 | 0.109068 | 0.782914 | 0.555006 |
| LNC_002551 | XLOC_059000 | -- | intronic_lncRNA | 0.348286 | 0.635568 | 0.928985 | 0.582923 |

| LNC_004286 | XLOC_093360 | -- | intronic_lncRNA | 0.69652 | 0.263894 | 1.341829 | 0.871454 |
| --- | --- | --- | --- | --- | --- | --- | --- |
| LNC_002558 | XLOC_059150 | -- | intronic_lncRNA | 1.555706 | 0 | 0 | 0 |
| LNC_002559 | XLOC_059150 | -- | intronic_lncRNA | 1.720249 | 0.120641 | 0.066648 | 0.069748 |
| LNC_003146 | XLOC_072555 | -- | intronic_lncRNA | 0.401584 | 1.100308 | 0.279804 | 0.79027 |
| LNC_003147 | XLOC_072659 | -- | lincRNA | 0.161516 | 0.162236 | 1.167165 | 0.978415 |
| LNC_003144 | XLOC_072550 | -- | intronic_lncRNA | 0.241845 | 0.461748 | 0.004698 | 0.590451 |
| LNC_003145 | XLOC_072550 | -- | intronic_lncRNA | 0.72279 | 0 | 0.477724 | 0.282195 |
| LNC_003142 | XLOC_072500 | -- | lincRNA | 0 | 0.667755 | 0 | 0 |
| LNC_003143 | XLOC_072523 | -- | lincRNA | 1.115851 | 0 | 0 | 0 |
| LNC_003140 | XLOC_072431 | -- | intronic_lncRNA | 0.43182 | 2.634826 | 0 | 0.305932 |
| LNC_003141 | XLOC_072432 | -- | intronic_lncRNA | 0.449536 | 0.63272 | 0 | 0.154267 |
| LNC_003148 | XLOC_072683 | -- | intronic_lncRNA | 0.816147 | 0.396446 | 0.55399 | 0.742226 |
| LNC_003149 | XLOC_072689 | -- | intronic_lncRNA | 0.562107 | 0 | 0.108919 | 0.453372 |
| LNC_000488 | XLOC_008675 | -- | intronic_lncRNA | 0.165612 | 0.328828 | 0.615132 | 0.267596 |
| LNC_000489 | XLOC_008676 | -- | intronic_lncRNA | 0.396447 | 0.192873 | 0.741905 | 0.776977 |
| LNC_000486 | XLOC_008670 | -- | intronic_lncRNA | 0.544401 | 0.29132 | 0.394076 | 0.517939 |
| LNC_000487 | XLOC_008671 | -- | intronic_lncRNA | 0.338091 | 0.340369 | 0.644875 | 0.327221 |
| LNC_000484 | XLOC_008654 | -- | intronic_lncRNA | 0.332971 | 0.151541 | 0.306988 | 0.708207 |
| LNC_000485 | XLOC_008655 | -- | intronic_lncRNA | 0.561585 | 0.22018 | 0.618551 | 0.414983 |
| LNC_000482 | XLOC_008639 | -- | intronic_lncRNA | 0.764691 | 0.334586 | 0.194021 | 0.198436 |
| LNC_000483 | XLOC_008653 | -- | intronic_lncRNA | 0.228857 | 0.685031 | 0.176913 | 0.184894 |
| LNC_000480 | XLOC_008433 | -- | intronic_lncRNA | 0.622089 | 0.302865 | 0.155641 | 0.428436 |
| LNC_000481 | XLOC_008586 | -- | intronic_lncRNA | 0.072121 | 0.050898 | 0.071462 | 0.524403 |
| LNC_002439 | XLOC_056474 | -- | lincRNA | 0.066026 | 0.174587 | 1.054453 | 1.043602 |
| LNC_002438 | XLOC_056345 | -- | antisense_lncRNA | 0 | 0.114094 | 0.528717 | 0.687281 |
| LNC_002435 | XLOC_056331 | -- | lincRNA | 0 | 0.069901 | 1.168819 | 1.09428 |
| LNC_002434 | XLOC_056331 | -- | lincRNA | 0 | 0 | 0.74333 | 0.104508 |
| LNC_002437 | XLOC_056337 | -- | intronic_lncRNA | 0.104839 | 0.188901 | 0.267803 | 0.780243 |
| LNC_002436 | XLOC_056331 | -- | lincRNA | 0 | 0 | 0.574956 | 0.582925 |
| LNC_002431 | XLOC_056217 | -- | intronic_lncRNA | 1.077701 | 0.154565 | 0.559519 | 0.102136 |
| LNC_002430 | XLOC_056217 | -- | intronic_lncRNA | 0.634376 | 1.70318 | 0.63382 | 1.06279 |
| LNC_002433 | XLOC_056228 | -- | intronic_lncRNA | 0.175353 | 0 | 0 | 0.462163 |
| LNC_002432 | XLOC_056228 | -- | intronic_lncRNA | 0.172667 | 0.347831 | 0 | 0.736096 |
| LNC_000121 | XLOC_001017 | -- | intronic_lncRNA | 0.535143 | 0.201069 | 0.313172 | 0.454635 |
| LNC_001157 | XLOC_024991 | -- | intronic_lncRNA | 0.621846 | 1.520129 | 0.818919 | 0.446788 |
| LNC_001156 | XLOC_024969 | -- | intronic_lncRNA | 0 | 0 | 0.437961 | 0 |
| LNC_000120 | XLOC_001016 | -- | intronic_lncRNA | 0.369077 | 0.594545 | 0.54933 | 0.475538 |
| LNC_004259 | XLOC_092799 | -- | intronic_lncRNA | 1.475263 | 0.628547 | 1.404737 | 1.350831 |
| LNC_004258 | XLOC_092472 | -- | intronic_lncRNA | 2.505398 | 1.33011 | 2.153812 | 1.167199 |
| LNC_004257 | XLOC_092232 | -- | intronic_lncRNA | 1.34018 | 0.824904 | 0.908171 | 1.186699 |
| LNC_004256 | XLOC_092230 | -- | intronic_lncRNA | 0.523816 | 0.257042 | 0.12417 | 0.60511 |
| LNC_004255 | XLOC_092205 | -- | intronic_lncRNA | 0.189247 | 0.149483 | 0.50174 | 0.368522 |
| LNC_004254 | XLOC_092199 | -- | intronic_lncRNA | 0 | 0.572362 | 0.103007 | 0.205635 |
| LNC_004253 | XLOC_092198 | -- | intronic_lncRNA | 0 | 0.653101 | 0 | 0.106602 |
| LNC_004252 | XLOC_092198 | -- | intronic_lncRNA | 0.041299 | 0.02068 | 1.005731 | 0.004778 |
| LNC_004251 | XLOC_092198 | -- | intronic_lncRNA | 0.562383 | 0.281783 | 0.398992 | 1.138555 |
| LNC_004250 | XLOC_092161 | -- | intronic_lncRNA | 0.893185 | 0.303085 | 0.129131 | 0.26833 |
| LNC_001162 | XLOC_025380 | -- | intronic_lncRNA | 0 | 0.566769 | 0 | 0 |
| LNC_001163 | XLOC_025382 | -- | intronic_lncRNA | 0.125279 | 0.499994 | 0 | 0 |
| LNC_001160 | XLOC_025224 | -- | lincRNA | 0.908365 | 0.355738 | 0.239913 | 0.34746 |
| LNC_001161 | XLOC_025377 | -- | intronic_lncRNA | 0.469087 | 0.32768 | 0.446935 | 0.297168 |
| LNC_001166 | XLOC_025419 | -- | intronic_lncRNA | 0.712801 | 0.677159 | 0.791157 | 0.507793 |
| LNC_001167 | XLOC_025420 | -- | intronic_lncRNA | 0 | 0.490611 | 0.732312 | 0.003248 |
| LNC_001164 | XLOC_025397 | -- | intronic_lncRNA | 0 | 0.678146 | 0.119235 | 0.543087 |
| LNC_001165 | XLOC_025403 | -- | intronic_lncRNA | 0.313929 | 0.414726 | 0.687433 | 0.763594 |
| LNC_001168 | XLOC_025420 | -- | intronic_lncRNA | 0.577972 | 0.165304 | 0 | 0.530957 |
| LNC_001169 | XLOC_025482 | -- | intronic_lncRNA | 0.777019 | 0.809343 | 1.269788 | 1.02307 |
| LNC_000127 | XLOC_001160 | -- | intronic_lncRNA | 2.622716 | 5.004498 | 3.213024 | 3.579185 |
| LNC_003708 | XLOC_079378 | -- | intronic_lncRNA | 0.753821 | 0.213533 | 0.354822 | 0.603092 |
| LNC_003709 | XLOC_079389 | -- | intronic_lncRNA | 1.582446 | 0.277045 | 1.21022 | 0.628448 |
| LNC_003704 | XLOC_079276 | -- | intronic_lncRNA | 0.368427 | 0 | 0.441045 | 0 |
| LNC_003705 | XLOC_079281 | -- | antisense_lncRNA | 0 | 0 | 0.903294 | 0.451569 |
| LNC_003706 | XLOC_079370 | -- | intronic_lncRNA | 0.857571 | 0.450225 | 0.236284 | 0.292297 |
| LNC_003707 | XLOC_079375 | -- | intronic_lncRNA | 0.191678 | 0 | 0.569625 | 1.024102 |
| LNC_003700 | XLOC_079249 | -- | intronic_lncRNA | 0.384841 | 0.668302 | 0.789326 | 0.786193 |
| LNC_003701 | XLOC_079249 | -- | intronic_lncRNA | 0.834983 | 1.103369 | 1.527528 | 3.420819 |
| LNC_003702 | XLOC_079262 | -- | intronic_lncRNA | 0.824892 | 0.778603 | 0.209493 | 0.525614 |
| LNC_003703 | XLOC_079262 | -- | intronic_lncRNA | 0.013369 | 0.219889 | 0.012713 | 0.46059 |
| LNC_003449 | XLOC_075227 | -- | lincRNA | 11.920263 | 3.679197 | 2.679223 | 3.396951 |
| LNC_003448 | XLOC_075226 | -- | lincRNA | 1.163861 | 0.378286 | 0.512138 | 1.429627 |
| LNC_003443 | XLOC_075222 | -- | lincRNA | 1.635706 | 0.730948 | 0.502523 | 0.337812 |
| LNC_003442 | XLOC_075221 | -- | lincRNA | 3.390599 | 2.161915 | 1.044921 | 1.44164 |
| LNC_003441 | XLOC_075220 | -- | lincRNA | 1.76545 | 0.819145 | 0.628279 | 0.466602 |
| LNC_003440 | XLOC_075220 | -- | lincRNA | 1.743353 | 0.473859 | 0.019286 | 0.145453 |
| LNC_003447 | XLOC_075226 | -- | lincRNA | 2.658624 | 1.035322 | 0.910081 | 0.87932 |
| LNC_003446 | XLOC_075225 | -- | lincRNA | 2.469344 | 0.908956 | 0.406683 | 0.680373 |
| LNC_003445 | XLOC_075224 | -- | lincRNA | 11.008135 | 5.653915 | 2.297007 | 3.906781 |

| LNC_003444 | XLOC_075223 | -- | lincRNA | 2.01841 | 0.725052 | 0.437685 | 0.489273 |
| --- | --- | --- | --- | --- | --- | --- | --- |
| LNC_005665 | XLOC_132384 | -- | intronic_lncRNA | 0 | 0.241647 | 0.931652 | 0.144956 |
| LNC_005664 | XLOC_132369 | -- | lincRNA | 0.491641 | 0.158983 | 0.827468 | 0.154113 |
| LNC_005667 | XLOC_132392 | -- | intronic_lncRNA | 0.186279 | 0.263633 | 0 | 0.637561 |
| LNC_005666 | XLOC_132388 | -- | intronic_lncRNA | 0.410976 | 0.294125 | 0.501473 | 0.250693 |
| LNC_005661 | XLOC_132218 | -- | intronic_lncRNA | 0.271721 | 0.36397 | 0.855276 | 0.882086 |
| LNC_005660 | XLOC_132216 | -- | intronic_lncRNA | 0.150206 | 0.660423 | 0 | 0.472463 |
| LNC_005663 | XLOC_132369 | -- | lincRNA | 0.368104 | 0.590342 | 0 | 0.684584 |
| LNC_005662 | XLOC_132234 | -- | lincRNA | 0 | 0 | 0 | 0.572722 |
| LNC_005669 | XLOC_132423 | -- | lincRNA | 0.524563 | 0 | 0 | 0 |
| LNC_005668 | XLOC_132393 | -- | intronic_lncRNA | 0.340719 | 0.532104 | 0.263346 | 0 |
| LNC_006088 | XLOC_145711 | -- | lincRNA | 0.967477 | 0 | 2.117254 | 2.2198 |
| LNC_006089 | XLOC_145711 | -- | lincRNA | 0.054083 | 0.926412 | 0.084489 | 0.293247 |
| LNC_006080 | XLOC_145314 | -- | intronic_lncRNA | 0.495021 | 0.188157 | 0 | 0.054467 |
| LNC_006081 | XLOC_145351 | -- | lincRNA | 2.780641 | 3.268506 | 2.76845 | 3.26244 |
| LNC_006082 | XLOC_145502 | -- | lincRNA | 0.214603 | 0.162009 | 0.343335 | 1.135636 |
| LNC_006083 | XLOC_145502 | -- | lincRNA | 0.043753 | 0 | 0.580466 | 0.106326 |
| LNC_006084 | XLOC_145503 | -- | lincRNA | 0.048917 | 0.552748 | 0.192401 | 0.388977 |
| LNC_006085 | XLOC_145545 | -- | lincRNA | 0.527117 | 0.675455 | 0.586347 | 0.5325 |
| LNC_006086 | XLOC_145548 | -- | lincRNA | 0.068942 | 0.677844 | 0.061331 | 0.780186 |
| LNC_006087 | XLOC_145585 | -- | lincRNA | 0 | 0.185104 | 0.437066 | 0.669975 |
| LNC_004538 | XLOC_099403 | -- | intronic_lncRNA | 0 | 0.493191 | 0 | 0 |
| LNC_004539 | XLOC_099407 | -- | intronic_lncRNA | 0 | 0 | 0 | 0.464982 |
| LNC_004532 | XLOC_099354 | -- | intronic_lncRNA | 0.324186 | 0.240648 | 0.527023 | 0.327803 |
| LNC_004533 | XLOC_099382 | -- | intronic_lncRNA | 2.562206 | 2.044163 | 13.990126 | 4.999042 |
| LNC_004530 | XLOC_099339 | -- | intronic_lncRNA | 1.405442 | 1.551122 | 1.354363 | 1.69576 |
| LNC_004531 | XLOC_099353 | -- | intronic_lncRNA | 0.363393 | 0.211203 | 0.679639 | 0.626553 |
| LNC_004536 | XLOC_099401 | -- | intronic_lncRNA | 0.305895 | 0.526312 | 0.520573 | 0.498555 |
| LNC_004537 | XLOC_099401 | -- | intronic_lncRNA | 0 | 0 | 0.558707 | 0.306098 |
| LNC_004534 | XLOC_099382 | -- | antisense_lncRNA | 1.287115 | 0.619331 | 0.944558 | 0.714636 |
| LNC_004535 | XLOC_099385 | -- | lincRNA | 0.674682 | 0.603836 | 0.282041 | 0.315103 |
| LNC_000543 | XLOC_010014 | -- | intronic_lncRNA | 0.747774 | 0.372425 | 0.424805 | 0.391562 |
| LNC_000542 | XLOC_010005 | -- | lincRNA | 0 | 0.484337 | 0.485398 | 0.098044 |
| LNC_000541 | XLOC_009994 | -- | intronic_lncRNA | 0.559975 | 0.375979 | 1.117766 | 1.074441 |
| LNC_000540 | XLOC_009994 | -- | intronic_lncRNA | 0.6765 | 0.308216 | 0.925515 | 0.784537 |
| LNC_000547 | XLOC_010030 | -- | intronic_lncRNA | 0.182225 | 0 | 0.368113 | 0.485335 |
| LNC_000546 | XLOC_010029 | -- | intronic_lncRNA | 0.435125 | 0.368934 | 0.23182 | 0.322552 |
| LNC_000545 | XLOC_010021 | -- | intronic_lncRNA | 0.385603 | 0.464251 | 0.233688 | 0.036937 |
| LNC_005064 | XLOC_115469 | -- | lincRNA | 0.120915 | 0 | 0.335939 | 0 |
| LNC_000549 | XLOC_010155 | -- | intronic_lncRNA | 0.86629 | 0.503513 | 0.71848 | 0.941294 |
| LNC_000548 | XLOC_010055 | -- | lincRNA | 0 | 0.267118 | 0.595658 | 0.021837 |
| LNC_003939 | XLOC_084852 | -- | antisense_lncRNA | 1.393462 | 2.100198 | 0.5742 | 0.904053 |
| LNC_003938 | XLOC_084789 | -- | intronic_lncRNA | 0.763388 | 1.159317 | 0.337776 | 0.551048 |
| LNC_003933 | XLOC_084657 | -- | intronic_lncRNA | 0.387344 | 0.441075 | 0.409732 | 0.725149 |
| LNC_003932 | XLOC_084656 | -- | intronic_lncRNA | 0.810573 | 0 | 0.086707 | 0 |
| LNC_003931 | XLOC_084644 | -- | intronic_lncRNA | 0.873438 | 0.340748 | 0.53324 | 0.436519 |
| LNC_003930 | XLOC_084644 | -- | intronic_lncRNA | 1.004873 | 0.549788 | 1.067481 | 0.814806 |
| LNC_003937 | XLOC_084773 | -- | intronic_lncRNA | 0.075005 | 0.062378 | 0.398062 | 0 |
| LNC_003936 | XLOC_084765 | -- | antisense_lncRNA | 0.990112 | 0 | 0 | 0 |
| LNC_003935 | XLOC_084749 | -- | antisense_lncRNA | 0.685634 | 1.019776 | 1.357777 | 2.548228 |
| LNC_003934 | XLOC_084657 | -- | intronic_lncRNA | 0.514841 | 0 | 0.025595 | 0.043046 |
| LNC_002488 | XLOC_057754 | -- | intronic_lncRNA | 3.212994 | 2.978707 | 3.129359 | 3.488373 |
| LNC_002489 | XLOC_057769 | -- | intronic_lncRNA | 0 | 0.236515 | 0.201883 | 0.433334 |
| LNC_000150 | XLOC_001522 | -- | intronic_lncRNA | 0.119992 | 0.089576 | 0.860925 | 0.484353 |
| LNC_000151 | XLOC_001524 | -- | intronic_lncRNA | 0.027047 | 0 | 0.774853 | 0.007932 |
| LNC_000152 | XLOC_001530 | -- | intronic_lncRNA | 0.509909 | 0.127192 | 0.120175 | 0.102989 |
| LNC_000153 | XLOC_001531 | -- | intronic_lncRNA | 0.994312 | 0 | 0.326347 | 0.565821 |
| LNC_000154 | XLOC_001532 | -- | intronic_lncRNA | 0.227959 | 0 | 0.64102 | 0 |
| LNC_000155 | XLOC_001582 | -- | intronic_lncRNA | 0.124204 | 0.73132 | 0.675733 | 0.361966 |
| LNC_000156 | XLOC_001677 | -- | intronic_lncRNA | 0.351669 | 0.300078 | 0.084814 | 0.701807 |
| LNC_000157 | XLOC_001703 | -- | intronic_lncRNA | 0.727014 | 0.70947 | 0.119338 | 0.402655 |
| LNC_000158 | XLOC_001722 | -- | intronic_lncRNA | 0.891994 | 0.63853 | 0.456448 | 0.412667 |
| LNC_000159 | XLOC_001726 | -- | intronic_lncRNA | 0.709793 | 0.339856 | 0.467515 | 0.324623 |
| LNC_003775 | XLOC_080744 | -- | intronic_lncRNA | 0.111477 | 0.108269 | 0.412053 | 0.46616 |
| LNC_000289 | XLOC_004261 | -- | antisense_lncRNA | 0.492721 | 0.146841 | 0.105356 | 0 |
| LNC_000428 | XLOC_007668 | -- | intronic_lncRNA | 0 | 0 | 0.01729 | 0.440391 |
| LNC_005149 | XLOC_117651 | -- | intronic_lncRNA | 0 | 0 | 0.393065 | 0 |
| LNC_001399 | XLOC_030526 | -- | intronic_lncRNA | 0.039952 | 0.101 | 9.544208 | 4.539011 |
| LNC_001398 | XLOC_030526 | -- | intronic_lncRNA | 10.61707 | 19.309669 | 0 | 6.869486 |
| LNC_001391 | XLOC_030497 | -- | intronic_lncRNA | 0 | 0.659641 | 0.75728 | 0.677315 |
| LNC_001390 | XLOC_030496 | -- | antisense_lncRNA | 0.399637 | 0.947639 | 0.7697 | 0.19151 |
| LNC_001393 | XLOC_030502 | -- | lincRNA | 0 | 0.268376 | 0.183685 | 0.80682 |
| LNC_001392 | XLOC_030498 | -- | intronic_lncRNA | 0.22419 | 0.338868 | 0.599956 | 0.490112 |
| LNC_001395 | XLOC_030514 | -- | intronic_lncRNA | 0.516825 | 0.17818 | 0.515359 | 0.230154 |
| LNC_001394 | XLOC_030502 | -- | lincRNA | 0.166085 | 0.35423 | 0.502514 | 0.230273 |
| LNC_001397 | XLOC_030522 | -- | intronic_lncRNA | 0.923622 | 0.13482 | 0.869972 | 0.57388 |
| LNC_001396 | XLOC_030516 | -- | intronic_lncRNA | 1.072734 | 0.45892 | 0.192191 | 0.44615 |

| LNC_000284 | XLOC_004115 | -- | intronic_lncRNA | 0.994035 | 0.382542 | 0.467011 | 0.437992 |
| --- | --- | --- | --- | --- | --- | --- | --- |
| LNC_000285 | XLOC_004117 | -- | intronic_lncRNA | 0.27859 | 0 | 0.475089 | 0.221088 |
| LNC_005140 | XLOC_117364 | -- | intronic_lncRNA | 0.820176 | 0.704052 | 1.133335 | 1.652625 |
| LNC_000421 | XLOC_007390 | -- | intronic_lncRNA | 0.934263 | 1.572415 | 0.470416 | 0.813692 |
| LNC_000422 | XLOC_007391 | -- | intronic_lncRNA | 0.045891 | 0.275661 | 0.573235 | 0.346835 |
| LNC_004701 | XLOC_104447 | -- | intronic_lncRNA | 0.257807 | 0.197372 | 0.511642 | 0.587703 |
| LNC_004700 | XLOC_104446 | -- | intronic_lncRNA | 0.548763 | 0.41535 | 0.435416 | 0.554102 |
| LNC_004703 | XLOC_104506 | -- | lincRNA | 0.572788 | 0.196073 | 0.038916 | 0.308808 |
| LNC_004702 | XLOC_104462 | -- | intronic_lncRNA | 0 | 0.843013 | 0 | 0 |
| LNC_004705 | XLOC_104566 | -- | intronic_lncRNA | 0 | 0.717811 | 0.052075 | 0 |
| LNC_004704 | XLOC_104559 | -- | intronic_lncRNA | 0 | 0 | 0.124096 | 0.538025 |
| LNC_004707 | XLOC_104584 | -- | intronic_lncRNA | 0 | 0.229956 | 0.145874 | 0.583393 |
| LNC_004706 | XLOC_104566 | -- | intronic_lncRNA | 0.424806 | 0.225358 | 0.257243 | 0.519281 |
| LNC_004709 | XLOC_104587 | -- | intronic_lncRNA | 0.134823 | 0 | 0 | 0.716387 |
| LNC_000424 | XLOC_007405 | -- | lincRNA | 0.395911 | 0.040546 | 1.005136 | 0.519317 |
| LNC_000425 | XLOC_007405 | -- | lincRNA | 1.251408 | 0 | 0 | 1.076336 |
| LNC_000426 | XLOC_007455 | -- | intronic_lncRNA | 0.565683 | 0.576408 | 0.696284 | 0.935252 |
| LNC_005147 | XLOC_117579 | -- | intronic_lncRNA | 4.036568 | 4.550105 | 3.389285 | 0 |
| LNC_000846 | XLOC_018060 | -- | intronic_lncRNA | 0.384979 | 0.491057 | 0.265125 | 0.484506 |
| LNC_000847 | XLOC_018105 | -- | intronic_lncRNA | 0.583587 | 0.203615 | 0.055522 | 0.16006 |
| LNC_000844 | XLOC_018043 | -- | intronic_lncRNA | 0.019421 | 0 | 1.130691 | 0.017179 |
| LNC_000845 | XLOC_018043 | -- | intronic_lncRNA | 2.050881 | 2.411344 | 1.376732 | 1.833056 |
| LNC_000842 | XLOC_018015 | -- | intronic_lncRNA | 0.884755 | 0 | 0 | 0 |
| LNC_000843 | XLOC_018020 | -- | intronic_lncRNA | 0.683497 | 0.745063 | 0.404015 | 0.621038 |
| LNC_000840 | XLOC_018003 | -- | intronic_lncRNA | 0.272894 | 0.415845 | 0.60147 | 0.596819 |
| LNC_000841 | XLOC_018015 | -- | antisense_lncRNA | 0 | 0 | 0 | 0.613377 |
| LNC_000848 | XLOC_018167 | -- | intronic_lncRNA | 0.500075 | 0 | 0.525042 | 0.242474 |
| LNC_000849 | XLOC_018169 | -- | intronic_lncRNA | 0.925011 | 0 | 0 | 0.078665 |
| LNC_006224 | XLOC_149301 | -- | intronic_lncRNA | 0.812677 | 0.044776 | 1.838586 | 1.30943 |
| LNC_006225 | XLOC_149304 | -- | intronic_lncRNA | 0.985278 | 0.995097 | 1.422634 | 1.966589 |
| LNC_006226 | XLOC_149305 | -- | intronic_lncRNA | 0.733272 | 1.095502 | 0.951729 | 1.713248 |
| LNC_005930 | XLOC_140151 | -- | intronic_lncRNA | 0.423043 | 0.535287 | 0.575634 | 0.422457 |
| LNC_006227 | XLOC_149403 | -- | intronic_lncRNA | 0.798876 | 0.157149 | 0 | 0 |
| LNC_006220 | XLOC_149192 | -- | intronic_lncRNA | 0.440044 | 0.36289 | 0.638856 | 0.530324 |
| LNC_006221 | XLOC_149212 | -- | intronic_lncRNA | 2.548229 | 6.443275 | 5.170561 | 6.329531 |
| LNC_006222 | XLOC_149232 | -- | intronic_lncRNA | 0.253867 | 0 | 0.410186 | 0.640407 |
| LNC_006223 | XLOC_149301 | -- | intronic_lncRNA | 0.814103 | 1.200742 | 0.856864 | 1.091153 |
| LNC_006228 | XLOC_149405 | -- | intronic_lncRNA | 4.420338 | 5.491381 | 3.440278 | 4.725534 |
| LNC_006229 | XLOC_149423 | -- | intronic_lncRNA | 0.857856 | 0.79014 | 0.04993 | 0.531695 |
| LNC_006419 | XLOC_153987 | -- | intronic_lncRNA | 3.712465 | 1.526394 | 4.110418 | 6.293297 |
| LNC_006418 | XLOC_153987 | -- | intronic_lncRNA | 0.918361 | 0.619372 | 1.902008 | 2.362293 |
| LNC_006413 | XLOC_153869 | -- | intronic_lncRNA | 0.150706 | 0.568621 | 0.383752 | 0.306949 |
| LNC_006412 | XLOC_153866 | -- | intronic_lncRNA | 0 | 1.15185 | 0.097928 | 0 |
| LNC_006411 | XLOC_153813 | -- | lincRNA | 0.350755 | 0 | 0.287579 | 0 |
| LNC_006410 | XLOC_153752 | -- | intronic_lncRNA | 0.460687 | 0 | 0.35028 | 0.011703 |
| LNC_006417 | XLOC_153920 | -- | intronic_lncRNA | 0.335026 | 1.111042 | 0.254817 | 0.383043 |
| LNC_006416 | XLOC_153909 | -- | intronic_lncRNA | 0.357499 | 0.567307 | 0.039017 | 1.058019 |
| LNC_006415 | XLOC_153907 | -- | intronic_lncRNA | 0.507713 | 0.386421 | 0.396528 | 0 |
| LNC_006414 | XLOC_153870 | -- | intronic_lncRNA | 0 | 0 | 0.036073 | 0.460423 |
| LNC_000266 | XLOC_003896 | -- | lincRNA | 0 | 0 | 0 | 0.517577 |
| LNC_000718 | XLOC_014383 | -- | intronic_lncRNA | 0 | 0 | 0 | 0.341065 |
| LNC_000719 | XLOC_014386 | -- | intronic_lncRNA | 0 | 0.946139 | 0.09586 | 0 |
| LNC_000716 | XLOC_014364 | -- | intronic_lncRNA | 0 | 0 | 0.516085 | 0 |
| LNC_000717 | XLOC_014364 | -- | intronic_lncRNA | 0.918217 | 0.322065 | 0.559327 | 0.985595 |
| LNC_000714 | XLOC_014339 | -- | intronic_lncRNA | 1.449736 | 0.96931 | 0.768735 | 0.686729 |
| LNC_000715 | XLOC_014339 | -- | intronic_lncRNA | 0.081826 | 0.108865 | 0.048387 | 0.71807 |
| LNC_000712 | XLOC_014089 | -- | intronic_lncRNA | 0.362153 | 0.171968 | 0 | 0.538376 |
| LNC_000713 | XLOC_014262 | -- | intronic_lncRNA | 0.994553 | 0.232978 | 0.465542 | 0.178718 |
| LNC_000710 | XLOC_014087 | -- | intronic_lncRNA | 0.449103 | 0.446475 | 0.586907 | 0.606241 |
| LNC_000711 | XLOC_014088 | -- | intronic_lncRNA | 0.330259 | 0.521098 | 0.306748 | 0.137365 |
| LNC_002578 | XLOC_059786 | -- | intronic_lncRNA | 0.229319 | 0.133026 | 0.260712 | 0.753996 |
| LNC_002579 | XLOC_059816 | -- | intronic_lncRNA | 0.121795 | 0 | 0 | 0.479459 |
| LNC_002570 | XLOC_059693 | -- | intronic_lncRNA | 0.097037 | 0.173905 | 0 | 0.596215 |
| LNC_002571 | XLOC_059694 | -- | intronic_lncRNA | 0.313419 | 0.385805 | 0.256901 | 0.305104 |
| LNC_002572 | XLOC_059748 | -- | lincRNA | 0.962918 | 0.326089 | 0.169571 | 0 |
| LNC_002573 | XLOC_059749 | -- | lincRNA | 1.134263 | 0.203835 | 0.14451 | 0.154382 |
| LNC_002574 | XLOC_059749 | -- | lincRNA | 0.741087 | 0 | 0 | 0.004689 |
| LNC_002575 | XLOC_059750 | -- | lincRNA | 0.854874 | 0.170357 | 0.169561 | 0.144731 |
| LNC_002576 | XLOC_059750 | -- | lincRNA | 0.730117 | 0.139751 | 0 | 0 |
| LNC_002577 | XLOC_059779 | -- | intronic_lncRNA | 0.135395 | 0.090061 | 0.424784 | 0.364378 |
| LNC_003168 | XLOC_073433 | -- | intronic_lncRNA | 0.642015 | 0.008623 | 0.27812 | 0.507405 |
| LNC_003169 | XLOC_073438 | -- | intronic_lncRNA | 0.250966 | 0 | 0 | 0.606535 |
| LNC_003160 | XLOC_072984 | -- | intronic_lncRNA | 0.102599 | 0.320951 | 0.337303 | 0.565469 |
| LNC_003161 | XLOC_073055 | -- | intronic_lncRNA | 0.507682 | 0 | 0.410229 | 0.165393 |
| LNC_003162 | XLOC_073062 | -- | antisense_lncRNA | 0.802062 | 0 | 0 | 0 |
| LNC_003163 | XLOC_073088 | -- | intronic_lncRNA | 0.789122 | 0 | 0 | 0.053246 |
| LNC_003164 | XLOC_073279 | -- | intronic_lncRNA | 0.40992 | 0.228201 | 0.362823 | 0.828722 |

| LNC_003165 | XLOC_073291 | -- | intronic_lncRNA | 0.720361 | 0.309206 | 0.407555 | 0.30247 |
| --- | --- | --- | --- | --- | --- | --- | --- |
| LNC_003166 | XLOC_073292 | -- | intronic_lncRNA | 1.091449 | 0.298333 | 0.070106 | 0 |
| LNC_003167 | XLOC_073402 | -- | intronic_lncRNA | 0.352757 | 0.199739 | 0.802595 | 1.210672 |
| LNC_002417 | XLOC_055905 | -- | lincRNA | 1.202804 | 0.442411 | 0.557755 | 0.686686 |
| LNC_002416 | XLOC_055893 | -- | intronic_lncRNA | 0.89269 | 0.584282 | 1.072564 | 1.118699 |
| LNC_002415 | XLOC_055891 | -- | intronic_lncRNA | 0 | 0.67161 | 0.147486 | 0 |
| LNC_002414 | XLOC_055856 | -- | intronic_lncRNA | 0.05051 | 0.413362 | 0.773997 | 0.526415 |
| LNC_002413 | XLOC_055855 | -- | intronic_lncRNA | 0.419558 | 0.763802 | 0.590099 | 0.662002 |
| LNC_002412 | XLOC_055831 | -- | intronic_lncRNA | 0.882047 | 0.594865 | 0.256228 | 1.826078 |
| LNC_002411 | XLOC_055783 | -- | intronic_lncRNA | 0.482986 | 0.937757 | 1.091382 | 0.29661 |
| LNC_002410 | XLOC_055781 | -- | intronic_lncRNA | 0.891938 | 0.384738 | 0.651634 | 0.845462 |
| LNC_002419 | XLOC_055931 | -- | intronic_lncRNA | 0.287906 | 0.07496 | 0.465345 | 0.337323 |
| LNC_002418 | XLOC_055930 | -- | intronic_lncRNA | 0.137667 | 0.214631 | 0.422121 | 0.75479 |
| LNC_001009 | XLOC_021713 | -- | antisense_lncRNA | 0.185441 | 0.306065 | 0.23731 | 0.634318 |
| LNC_001008 | XLOC_021635 | -- | intronic_lncRNA | 0.071964 | 0.532236 | 0 | 0 |
| LNC_001001 | XLOC_021236 | -- | intronic_lncRNA | 0 | 0.104799 | 0.393065 | 0 |
| LNC_001000 | XLOC_021235 | -- | intronic_lncRNA | 0 | 0.593453 | 0.44593 | 0.148618 |
| LNC_001003 | XLOC_021282 | -- | intronic_lncRNA | 0.73494 | 0.210305 | 0.485759 | 0.627521 |
| LNC_001002 | XLOC_021242 | -- | lincRNA | 0.818727 | 0.159566 | 0.595411 | 0 |
| LNC_001005 | XLOC_021309 | -- | intronic_lncRNA | 0.25724 | 0.079234 | 0.737402 | 0.478614 |
| LNC_001004 | XLOC_021308 | -- | intronic_lncRNA | 0.311874 | 0.691547 | 0.297519 | 0.580576 |
| LNC_001007 | XLOC_021336 | -- | intronic_lncRNA | 0 | 0.111029 | 0.814531 | 0.542927 |
| LNC_001006 | XLOC_021324 | -- | intronic_lncRNA | 0.450617 | 0.505808 | 0.271875 | 0.273042 |
| LNC_001144 | XLOC_024887 | -- | intronic_lncRNA | 0.403495 | 0 | 0.535132 | 0.50838 |
| LNC_001145 | XLOC_024904 | -- | intronic_lncRNA | 0.201447 | 0.444911 | 0.608319 | 1.059003 |
| LNC_001146 | XLOC_024904 | -- | intronic_lncRNA | 0.903982 | 0.566205 | 1.019113 | 1.512212 |
| LNC_001147 | XLOC_024905 | -- | intronic_lncRNA | 0 | 0 | 0.412029 | 0.154484 |
| LNC_001140 | XLOC_024821 | -- | intronic_lncRNA | 0.789173 | 0.144062 | 0.262925 | 0.269303 |
| LNC_001141 | XLOC_024829 | -- | intronic_lncRNA | 0.69527 | 0.299849 | 0.320015 | 0.60705 |
| LNC_001142 | XLOC_024861 | -- | intronic_lncRNA | 0.993596 | 0.352514 | 0.752959 | 0.724459 |
| LNC_001143 | XLOC_024873 | -- | lincRNA | 0.911675 | 0.729108 | 1.196755 | 1.330623 |
| LNC_001148 | XLOC_024910 | -- | intronic_lncRNA | 0.519014 | 0 | 0 | 0 |
| LNC_001149 | XLOC_024929 | -- | intronic_lncRNA | 0.300107 | 0 | 0.207526 | 0.432811 |
| LNC_005060 | XLOC_115398 | -- | intronic_lncRNA | 0.656724 | 0.663765 | 1.173203 | 1.239379 |
| LNC_003898 | XLOC_083821 | -- | lincRNA | 0.791949 | 0 | 0 | 0.538569 |
| LNC_003899 | XLOC_083892 | -- | intronic_lncRNA | 0.108421 | 0.132993 | 0.532203 | 0.080745 |
| LNC_003894 | XLOC_083665 | -- | intronic_lncRNA | 0.061709 | 0.492563 | 0.132304 | 0.09971 |
| LNC_003895 | XLOC_083667 | -- | intronic_lncRNA | 0 | 0 | 0.728075 | 0.032407 |
| LNC_003896 | XLOC_083670 | -- | intronic_lncRNA | 0.530758 | 0.302895 | 0.593961 | 0.350471 |
| LNC_003897 | XLOC_083814 | -- | intronic_lncRNA | 0.585944 | 0.193794 | 0.37285 | 0.540153 |
| LNC_003890 | XLOC_083630 | -- | intronic_lncRNA | 0.183548 | 0.28519 | 0.776664 | 0.408826 |
| LNC_003891 | XLOC_083661 | -- | intronic_lncRNA | 0 | 0 | 0.691923 | 0.20395 |
| LNC_003892 | XLOC_083663 | -- | intronic_lncRNA | 0.282499 | 0.211506 | 0.229735 | 0.548635 |
| LNC_003893 | XLOC_083664 | -- | intronic_lncRNA | 0.434237 | 0.680743 | 0.037121 | 0.110242 |
| LNC_003766 | XLOC_080575 | -- | intronic_lncRNA | 0.557598 | 0.795578 | 0.555601 | 0.649404 |
| LNC_003767 | XLOC_080614 | -- | intronic_lncRNA | 1.116404 | 0.71327 | 0.312102 | 0 |
| LNC_003764 | XLOC_080526 | -- | intronic_lncRNA | 0.39265 | 0.845403 | 0.42225 | 0.14149 |
| LNC_003765 | XLOC_080527 | -- | intronic_lncRNA | 0.703234 | 1.068216 | 0.409058 | 0.411813 |
| LNC_003762 | XLOC_080521 | -- | intronic_lncRNA | 0.49543 | 0.039522 | 0 | 0.057369 |
| LNC_003763 | XLOC_080525 | -- | intronic_lncRNA | 0.495337 | 0.515017 | 0.387835 | 0.436433 |
| LNC_003760 | XLOC_080447 | -- | antisense_lncRNA | 0 | 0 | 0 | 0.561766 |
| LNC_003761 | XLOC_080521 | -- | intronic_lncRNA | 0.707057 | 0.436436 | 0.278126 | 0.228735 |
| LNC_003768 | XLOC_080616 | -- | intronic_lncRNA | 4.486349 | 1.171084 | 0.5299 | 0.34953 |
| LNC_003769 | XLOC_080647 | -- | lincRNA | 0 | 1.108698 | 0.121687 | 0.048667 |
| LNC_005395 | XLOC_124373 | -- | intronic_lncRNA | 0.091208 | 0.207229 | 0.748789 | 0.901601 |
| LNC_005394 | XLOC_124370 | -- | intronic_lncRNA | 0.254753 | 0.319545 | 0.770503 | 0.564167 |
| LNC_005397 | XLOC_124422 | -- | antisense_lncRNA | 0.607743 | 0 | 0 | 0 |
| LNC_005396 | XLOC_124374 | -- | intronic_lncRNA | 0 | 0.190975 | 0.476358 | 0.047309 |
| LNC_005391 | XLOC_124320 | -- | intronic_lncRNA | 0 | 0 | 0.44153 | 0.419743 |
| LNC_005390 | XLOC_124318 | -- | intronic_lncRNA | 0.498083 | 0.420504 | 0.542924 | 0.227499 |
| LNC_005393 | XLOC_124357 | -- | intronic_lncRNA | 0.820055 | 0.972226 | 0.35799 | 0 |
| LNC_005392 | XLOC_124351 | -- | intronic_lncRNA | 0.515211 | 0.672177 | 0 | 0 |
| LNC_005399 | XLOC_124544 | -- | intronic_lncRNA | 0.714151 | 0.305631 | 0.190705 | 0 |
| LNC_005398 | XLOC_124486 | -- | intronic_lncRNA | 0.281032 | 0.177329 | 0.491733 | 0.554256 |
| LNC_003429 | XLOC_075209 | -- | lincRNA | 3.219152 | 1.189347 | 0.945306 | 1.118938 |
| LNC_003428 | XLOC_075209 | -- | lincRNA | 1.239509 | 0.421524 | 0.158338 | 0.484905 |
| LNC_003425 | XLOC_075207 | -- | lincRNA | 1.587953 | 0.400179 | 0.554243 | 0.650927 |
| LNC_003424 | XLOC_075207 | -- | lincRNA | 0.038789 | 1.039485 | 0.063753 | 0.129106 |
| LNC_003427 | XLOC_075208 | -- | lincRNA | 1.566524 | 0.829144 | 0.549961 | 0.520832 |
| LNC_003605 | XLOC_076625 | -- | intronic_lncRNA | 0.910748 | 0.677213 | 0.760591 | 0.88772 |
| LNC_003421 | XLOC_075207 | -- | lincRNA | 3.569412 | 1.053534 | 0.440353 | 1.263993 |
| LNC_003420 | XLOC_075207 | -- | lincRNA | 1.995919 | 0.979594 | 0.568456 | 0.661005 |
| LNC_003423 | XLOC_075207 | -- | lincRNA | 2.261237 | 0.545556 | 0.621644 | 0.22782 |
| LNC_003422 | XLOC_075207 | -- | lincRNA | 1.749423 | 0.901181 | 0.408849 | 0.419209 |
| LNC_005649 | XLOC_131580 | -- | intronic_lncRNA | 0.998404 | 1.124139 | 0.886325 | 0.74501 |
| LNC_005648 | XLOC_131579 | -- | intronic_lncRNA | 1.521402 | 1.021404 | 1.02741 | 1.590548 |
| LNC_005647 | XLOC_131568 | -- | intronic_lncRNA | 0.574748 | 1.290667 | 0.877482 | 1.093318 |

| LNC_005646 | XLOC_131523 | -- | lincRNA | 0.494324 | 0 | 0.078305 | 0.674216 |
| --- | --- | --- | --- | --- | --- | --- | --- |
| LNC_005645 | XLOC_131515 | -- | intronic_lncRNA | 2.155476 | 1.715868 | 2.234228 | 1.886124 |
| LNC_005644 | XLOC_131511 | -- | intronic_lncRNA | 1.09014 | 1.69557 | 1.242021 | 1.779842 |
| LNC_005643 | XLOC_131510 | -- | intronic_lncRNA | 0.289397 | 0.868807 | 1.084661 | 1.324345 |
| LNC_005642 | XLOC_131479 | -- | intronic_lncRNA | 0.343274 | 0.824117 | 1.159551 | 0.775309 |
| LNC_005641 | XLOC_131475 | -- | intronic_lncRNA | 0 | 0.234032 | 0.412029 | 0.031845 |
| LNC_005640 | XLOC_131472 | -- | antisense_lncRNA | 0.205876 | 0.1159 | 0.561761 | 0 |
| LNC_002387 | XLOC_055163 | -- | lincRNA | 0.816288 | 0.416282 | 0.575481 | 0.860952 |
| LNC_002386 | XLOC_055146 | -- | intronic_lncRNA | 0.038049 | 0.170938 | 0 | 0.412914 |
| LNC_002385 | XLOC_055144 | -- | intronic_lncRNA | 0.119889 | 0.781976 | 0.123269 | 0.198941 |
| LNC_002384 | XLOC_055142 | -- | intronic_lncRNA | 0.392883 | 0.6334 | 0.132381 | 0.057355 |
| LNC_002383 | XLOC_055118 | -- | intronic_lncRNA | 0.108678 | 0.749991 | 0.099207 | 0.173875 |
| LNC_002382 | XLOC_055097 | -- | intronic_lncRNA | 0.801611 | 0.924026 | 0.499353 | 0.670084 |
| LNC_002381 | XLOC_055096 | -- | lincRNA | 0 | 0 | 0.771702 | 0 |
| LNC_002380 | XLOC_055092 | -- | intronic_lncRNA | 0.16296 | 0.533736 | 0.072042 | 0.134694 |
| LNC_002389 | XLOC_055256 | -- | intronic_lncRNA | 1.229582 | 0.199081 | 0.375243 | 0.765843 |
| LNC_002388 | XLOC_055196 | -- | intronic_lncRNA | 0 | 0.439119 | 0.29397 | 0.649817 |
| LNC_003397 | XLOC_075189 | -- | lincRNA | 4.346806 | 2.415569 | 0.685198 | 1.073743 |
| LNC_003396 | XLOC_075188 | -- | lincRNA | 1.779301 | 0.698381 | 0.281238 | 0.294842 |
| LNC_003395 | XLOC_075187 | -- | lincRNA | 1.317268 | 0.374979 | 0.373302 | 0.224525 |
| LNC_003394 | XLOC_075187 | -- | lincRNA | 1.356128 | 0.388913 | 0.148676 | 0.379094 |
| LNC_003393 | XLOC_075186 | -- | lincRNA | 1.25713 | 0.640581 | 0.392631 | 0.448126 |
| LNC_003392 | XLOC_075186 | -- | lincRNA | 0.884985 | 0 | 0 | 0 |
| LNC_003391 | XLOC_075185 | -- | lincRNA | 2.411828 | 0.785899 | 0.44192 | 0.408966 |
| LNC_003390 | XLOC_075184 | -- | lincRNA | 5.890934 | 2.332165 | 1.390715 | 1.067711 |
| LNC_003399 | XLOC_075191 | -- | lincRNA | 1.746716 | 0.580336 | 0.349178 | 0.198572 |
| LNC_003398 | XLOC_075190 | -- | lincRNA | 3.43647 | 1.489166 | 0.609697 | 0.576547 |
| LNC_006062 | XLOC_144648 | -- | intronic_lncRNA | 0 | 0 | 0 | 0.440969 |
| LNC_006063 | XLOC_144670 | -- | intronic_lncRNA | 0 | 0 | 0.393065 | 0.286233 |
| LNC_006060 | XLOC_144635 | -- | intronic_lncRNA | 0 | 0 | 0 | 0.439087 |
| LNC_006061 | XLOC_144644 | -- | lincRNA | 0 | 0.493611 | 0 | 0 |
| LNC_006066 | XLOC_144686 | -- | intronic_lncRNA | 0.529088 | 0.104881 | 0.311726 | 0.254773 |
| LNC_006067 | XLOC_144687 | -- | intronic_lncRNA | 0.151659 | 0.263598 | 0.393065 | 0.085633 |
| LNC_006064 | XLOC_144684 | -- | intronic_lncRNA | 0.265384 | 0.699893 | 0.339304 | 0.228423 |
| LNC_006065 | XLOC_144685 | -- | intronic_lncRNA | 0.832121 | 0.341888 | 0.108063 | 0.432176 |
| LNC_006068 | XLOC_144761 | -- | intronic_lncRNA | 0 | 0 | 0 | 0.604676 |
| LNC_006069 | XLOC_144998 | -- | lincRNA | 0.670094 | 0.698155 | 1.188771 | 1.659497 |
| LNC_004518 | XLOC_098957 | -- | lincRNA | 0.312678 | 0.11022 | 0.903058 | 0.271239 |
| LNC_004519 | XLOC_099007 | -- | lincRNA | 0.324972 | 0 | 0.599639 | 0 |
| LNC_004514 | XLOC_098909 | -- | antisense_lncRNA | 0.411547 | 0.439319 | 0.334953 | 0.244795 |
| LNC_004515 | XLOC_098923 | -- | intronic_lncRNA | 0.185775 | 0.096998 | 0.622517 | 0.238313 |
| LNC_004516 | XLOC_098926 | -- | intronic_lncRNA | 0.290206 | 0.250712 | 0.750682 | 0 |
| LNC_004517 | XLOC_098932 | -- | lincRNA | 0.41928 | 1.011005 | 0.68146 | 0.349148 |
| LNC_004510 | XLOC_098894 | -- | intronic_lncRNA | 0.192169 | 0.158252 | 0.359206 | 1.044109 |
| LNC_004511 | XLOC_098908 | -- | intronic_lncRNA | 0.886997 | 0.989182 | 0.008433 | 0.202777 |
| LNC_004512 | XLOC_098908 | -- | intronic_lncRNA | 0.050264 | 0 | 0.914757 | 0.254964 |
| LNC_004513 | XLOC_098908 | -- | intronic_lncRNA | 0 | 0 | 1.014853 | 0.696544 |
| LNC_006367 | XLOC_152556 | -- | intronic_lncRNA | 0.498172 | 0 | 0.386332 | 0.416833 |
| LNC_005005 | XLOC_114211 | -- | intronic_lncRNA | 0.174758 | 0.965671 | 0.488124 | 0.168356 |
| LNC_005004 | XLOC_114095 | -- | intronic_lncRNA | 0.517689 | 0.527106 | 0.307748 | 0.547983 |
| LNC_000527 | XLOC_009851 | -- | intronic_lncRNA | 0.074121 | 0 | 0 | 0.522975 |
| LNC_005006 | XLOC_114236 | -- | intronic_lncRNA | 2.638046 | 2.132126 | 1.902924 | 2.099558 |
| LNC_000521 | XLOC_009198 | -- | intronic_lncRNA | 0.276064 | 0.155912 | 1.141452 | 0.50498 |
| LNC_000520 | XLOC_009195 | -- | intronic_lncRNA | 0.733597 | 0.25401 | 0.767296 | 0.802859 |
| LNC_005003 | XLOC_114083 | -- | intronic_lncRNA | 0.167727 | 0 | 0 | 0.220801 |
| LNC_005002 | XLOC_113989 | -- | intronic_lncRNA | 0.071379 | 0.786332 | 0.162227 | 0.185551 |
| LNC_005009 | XLOC_114318 | -- | intronic_lncRNA | 0.979937 | 0.000335 | 0.326896 | 0.047885 |
| LNC_005008 | XLOC_114296 | -- | intronic_lncRNA | 1.498538 | 0.999661 | 1.019212 | 1.348055 |
| LNC_003959 | XLOC_085212 | -- | intronic_lncRNA | 0.30738 | 0.540393 | 0.032039 | 0.807224 |
| LNC_003958 | XLOC_085175 | -- | intronic_lncRNA | 0.634675 | 0.944819 | 1.513519 | 0.792638 |
| LNC_003955 | XLOC_085088 | -- | lincRNA | 0 | 0 | 0.436942 | 0 |
| LNC_003954 | XLOC_085084 | -- | intronic_lncRNA | 0.518172 | 0.378701 | 0.532958 | 0.971674 |
| LNC_003957 | XLOC_085129 | -- | intronic_lncRNA | 0.023112 | 0 | 0.418142 | 0 |
| LNC_003956 | XLOC_085120 | -- | intronic_lncRNA | 0.552505 | 0.445513 | 0.663754 | 0.775721 |
| LNC_003951 | XLOC_084980 | -- | intronic_lncRNA | 0.71664 | 0.573193 | 0.907954 | 0.470418 |
| LNC_003950 | XLOC_084975 | -- | intronic_lncRNA | 0.800833 | 0.708563 | 0.176726 | 0.152688 |
| LNC_003953 | XLOC_085052 | -- | lincRNA | 0.632319 | 0.324249 | 0.309952 | 0 |
| LNC_003952 | XLOC_085012 | -- | lincRNA | 1.347497 | 0.251431 | 0.09708 | 0.144671 |
| LNC_002602 | XLOC_060128 | -- | intronic_lncRNA | 0.775474 | 0.718106 | 1.11131 | 0.696071 |
| LNC_002603 | XLOC_060194 | -- | intronic_lncRNA | 1.182403 | 0 | 0 | 0 |
| LNC_002600 | XLOC_060118 | -- | intronic_lncRNA | 0.154425 | 0.063318 | 0.307672 | 0.438158 |
| LNC_002601 | XLOC_060119 | -- | intronic_lncRNA | 0.290648 | 0.905484 | 0 | 0 |
| LNC_002606 | XLOC_060254 | -- | intronic_lncRNA | 1.071167 | 0.499634 | 0.173354 | 0.285815 |
| LNC_002607 | XLOC_060257 | -- | intronic_lncRNA | 0.420826 | 0.850817 | 0.337712 | 0.319064 |
| LNC_002604 | XLOC_060194 | -- | intronic_lncRNA | 0.033926 | 0.928966 | 1.391333 | 0.98891 |
| LNC_002605 | XLOC_060202 | -- | intronic_lncRNA | 2.078772 | 1.127582 | 1.637613 | 1.373283 |
| LNC_002608 | XLOC_060268 | -- | intronic_lncRNA | 0.746455 | 0.315037 | 0.407023 | 0.671255 |

| LNC_002609 | XLOC_060272 | -- | intronic_lncRNA | 1.400446 | 2.141703 | 2.831103 | 2.00897 |
| --- | --- | --- | --- | --- | --- | --- | --- |
| LNC_004358 | XLOC_094993 | -- | intronic_lncRNA | 0.394985 | 0.974774 | 0.734232 | 0.63017 |
| LNC_004359 | XLOC_094997 | -- | intronic_lncRNA | 0.071071 | 0 | 0.501528 | 0.221975 |
| LNC_004352 | XLOC_094725 | -- | antisense_lncRNA | 0.775062 | 0 | 0.127631 | 0 |
| LNC_004353 | XLOC_094733 | -- | intronic_lncRNA | 0.299484 | 0.250518 | 0.557993 | 0.479563 |
| LNC_004350 | XLOC_094665 | -- | intronic_lncRNA | 0.348706 | 0.307417 | 0 | 0 |
| LNC_004351 | XLOC_094666 | -- | intronic_lncRNA | 0.283255 | 0.105086 | 0.603288 | 0.448526 |
| LNC_004356 | XLOC_094961 | -- | intronic_lncRNA | 0.681413 | 0.886122 | 0.109746 | 0.295636 |
| LNC_004357 | XLOC_094968 | -- | intronic_lncRNA | 0.190615 | 1.115976 | 0.540361 | 0.35339 |
| LNC_004354 | XLOC_094924 | -- | intronic_lncRNA | 0.378685 | 0.630427 | 0.23809 | 0.53276 |
| LNC_004355 | XLOC_094926 | -- | intronic_lncRNA | 1.138062 | 0.671239 | 0.523973 | 0.436445 |
| LNC_001801 | XLOC_040408 | -- | intronic_lncRNA | 1.135556 | 0.789905 | 0.965754 | 1.458183 |
| LNC_001800 | XLOC_040337 | -- | lincRNA | 0 | 0.721983 | 0 | 0 |
| LNC_001803 | XLOC_040521 | -- | lincRNA | 0 | 0.486414 | 0 | 0 |
| LNC_001802 | XLOC_040513 | -- | intronic_lncRNA | 0.794316 | 0.589446 | 0.531946 | 0.491607 |
| LNC_001805 | XLOC_040627 | -- | intronic_lncRNA | 0 | 0 | 0.445225 | 0 |
| LNC_001804 | XLOC_040587 | -- | lincRNA | 0 | 0.115018 | 0.447694 | 0.383895 |
| LNC_001807 | XLOC_040633 | -- | intronic_lncRNA | 0 | 0 | 0.705725 | 0 |
| LNC_001806 | XLOC_040633 | -- | intronic_lncRNA | 1.876953 | 1.962581 | 2.18636 | 2.050052 |
| LNC_001809 | XLOC_040705 | -- | intronic_lncRNA | 0.362806 | 0.357108 | 0.40318 | 0.441048 |
| LNC_001808 | XLOC_040704 | -- | intronic_lncRNA | 0.569469 | 0.574434 | 0.523742 | 0.581476 |
| LNC_004729 | XLOC_105511 | -- | intronic_lncRNA | 0.188063 | 0 | 0.308703 | 0.420724 |
| LNC_004728 | XLOC_105482 | -- | intronic_lncRNA | 1.356148 | 0.996189 | 1.128396 | 1.477585 |
| LNC_004822 | XLOC_107759 | -- | intronic_lncRNA | 2.950883 | 1.475083 | 1.082303 | 0.942713 |
| LNC_004722 | XLOC_105239 | -- | intronic_lncRNA | 0.513513 | 0 | 0 | 0 |
| LNC_004721 | XLOC_105238 | -- | intronic_lncRNA | 0.14178 | 0.565847 | 0.229128 | 0 |
| LNC_004720 | XLOC_105224 | -- | lincRNA | 0.124735 | 0 | 0.547029 | 0.193796 |
| LNC_004727 | XLOC_105328 | -- | intronic_lncRNA | 0.460352 | 0.229722 | 0.682752 | 0.382572 |
| LNC_004726 | XLOC_105296 | -- | intronic_lncRNA | 0.683367 | 0.426098 | 0.883316 | 1.155768 |
| LNC_004725 | XLOC_105288 | -- | intronic_lncRNA | 0 | 0 | 0.053629 | 0.661453 |
| LNC_004724 | XLOC_105267 | -- | intronic_lncRNA | 0.509993 | 0.043611 | 0.317925 | 0.684214 |
| LNC_006475 | XLOC_155778 | -- | lincRNA | 0.5414 | 0.396388 | 0.766971 | 0.511173 |
| LNC_006474 | XLOC_155773 | -- | intronic_lncRNA | 0.914766 | 0.735992 | 0.788373 | 1.278902 |
| LNC_006477 | XLOC_155931 | -- | intronic_lncRNA | 0 | 0.091568 | 0.540553 | 0.549032 |
| LNC_006476 | XLOC_155928 | -- | lincRNA | 0.145764 | 0.904701 | 0.266882 | 0.960255 |
| LNC_006471 | XLOC_155701 | -- | lincRNA | 0.824651 | 0 | 0 | 0 |
| LNC_006470 | XLOC_155650 | -- | intronic_lncRNA | 1.11146 | 0.836694 | 1.193635 | 1.305837 |
| LNC_006473 | XLOC_155717 | -- | lincRNA | 1.109027 | 0.101778 | 0.069629 | 0 |
| LNC_006472 | XLOC_155703 | -- | lincRNA | 0.950115 | 0.376716 | 0.130817 | 0.476837 |
| LNC_005922 | XLOC_139898 | -- | intronic_lncRNA | 0 | 0 | 0.119711 | 0.841512 |
| LNC_005923 | XLOC_139979 | -- | intronic_lncRNA | 0.526444 | 0.128643 | 0 | 0 |
| LNC_005920 | XLOC_139529 | -- | antisense_lncRNA | 1.094745 | 0.338169 | 0 | 0.410154 |
| LNC_006259 | XLOC_150220 | -- | lincRNA | 0.163945 | 0.863957 | 0.291969 | 0.391471 |
| LNC_005921 | XLOC_139605 | -- | lincRNA | 0.694574 | 0.222063 | 0.046971 | 0 |
| LNC_005926 | XLOC_140059 | -- | intronic_lncRNA | 0 | 0.421129 | 0.688288 | 0.765204 |
| LNC_005927 | XLOC_140067 | -- | antisense_lncRNA | 0.28207 | 0.524109 | 0.516195 | 0.586224 |
| LNC_005924 | XLOC_140030 | -- | lincRNA | 0.535397 | 0.041134 | 0 | 0 |
| LNC_005925 | XLOC_140059 | -- | intronic_lncRNA | 1.465995 | 0.793251 | 1.237054 | 0.955899 |
| LNC_000868 | XLOC_018609 | -- | intronic_lncRNA | 0.85112 | 0.216378 | 0.418383 | 0.173092 |
| LNC_000869 | XLOC_018613 | -- | intronic_lncRNA | 0.314214 | 0.184553 | 0.473437 | 0.373167 |
| LNC_006479 | XLOC_155933 | -- | intronic_lncRNA | 1.028259 | 0 | 0.197029 | 0.493639 |
| LNC_006478 | XLOC_155932 | -- | intronic_lncRNA | 0.389744 | 0.344168 | 0.637445 | 0.195292 |
| LNC_000860 | XLOC_018403 | -- | intronic_lncRNA | 1.72275 | 1.2656 | 1.407599 | 1.836896 |
| LNC_000861 | XLOC_018403 | -- | intronic_lncRNA | 0.248881 | 0.991449 | 0.444085 | 0.294317 |
| LNC_000862 | XLOC_018481 | -- | intronic_lncRNA | 0.096295 | 0.348693 | 0.561647 | 0.094115 |
| LNC_000863 | XLOC_018524 | -- | intronic_lncRNA | 0 | 0 | 0.515497 | 0 |
| LNC_000864 | XLOC_018549 | -- | intronic_lncRNA | 1.775685 | 0.563385 | 2.386579 | 2.103277 |
| LNC_000865 | XLOC_018566 | -- | lincRNA | 0.27288 | 0 | 0.494831 | 0.121384 |
| LNC_000866 | XLOC_018581 | -- | intronic_lncRNA | 0 | 0.082682 | 0.418507 | 0.196911 |
| LNC_000867 | XLOC_018595 | -- | lincRNA | 0.677799 | 0 | 0.535616 | 0 |
| LNC_000730 | XLOC_014858 | -- | lincRNA | 0.299867 | 0.952454 | 0.156571 | 0.283257 |
| LNC_000731 | XLOC_014875 | -- | intronic_lncRNA | 1.606452 | 0.485866 | 0.290507 | 0.624194 |
| LNC_000732 | XLOC_014928 | -- | intronic_lncRNA | 0 | 0.571592 | 0.223372 | 0 |
| LNC_000733 | XLOC_014933 | -- | intronic_lncRNA | 0.521342 | 0.048985 | 0.105317 | 0.052649 |
| LNC_000734 | XLOC_015038 | -- | intronic_lncRNA | 0.246806 | 0 | 0.690754 | 0.138574 |
| LNC_000735 | XLOC_015041 | -- | intronic_lncRNA | 0.152172 | 0.087868 | 0.205141 | 1.147852 |
| LNC_000736 | XLOC_015052 | -- | intronic_lncRNA | 0 | 0 | 0 | 0.538522 |
| LNC_000737 | XLOC_015059 | -- | intronic_lncRNA | 0.331807 | 0.122569 | 0.454363 | 0.087438 |
| LNC_000738 | XLOC_015060 | -- | intronic_lncRNA | 0.193581 | 0.315474 | 0.485653 | 0.111711 |
| LNC_000739 | XLOC_015088 | -- | intronic_lncRNA | 0 | 0 | 0.235965 | 0.480914 |
| LNC_005928 | XLOC_140079 | -- | intronic_lncRNA | 0.173377 | 0.085123 | 0.226327 | 0.648022 |
| LNC_005929 | XLOC_140143 | -- | intronic_lncRNA | 0.678788 | 1.195074 | 0.515151 | 0.506341 |
| LNC_002592 | XLOC_059984 | -- | lincRNA | 0.661621 | 0.586008 | 0.72007 | 0.800268 |
| LNC_002593 | XLOC_060030 | -- | lincRNA | 0 | 0 | 0 | 0.533672 |
| LNC_002590 | XLOC_059955 | -- | intronic_lncRNA | 0.526043 | 0.233911 | 0.48446 | 1.126643 |
| LNC_002591 | XLOC_059984 | -- | lincRNA | 1.076383 | 0.333062 | 0.511986 | 0.337334 |
| LNC_002596 | XLOC_060102 | -- | intronic_lncRNA | 0.508422 | 0 | 0.131008 | 0.167954 |

| LNC_002597 | XLOC_060104 | -- | intronic_lncRNA | 0 | 0.236568 | 0.450204 | 0.372605 |
| --- | --- | --- | --- | --- | --- | --- | --- |
| LNC_002594 | XLOC_060100 | -- | intronic_lncRNA | 0.14784 | 0.449432 | 0.146912 | 0.788819 |
| LNC_002595 | XLOC_060101 | -- | intronic_lncRNA | 0.309618 | 0.608384 | 0.242521 | 0 |
| LNC_002598 | XLOC_060109 | -- | lincRNA | 0 | 0 | 0.653382 | 0 |
| LNC_002599 | XLOC_060117 | -- | intronic_lncRNA | 0.025287 | 0.497848 | 0 | 0 |
| LNC_003182 | XLOC_073650 | -- | intronic_lncRNA | 0.697045 | 0.351024 | 0.487941 | 0.388159 |
| LNC_003183 | XLOC_073653 | -- | intronic_lncRNA | 1.188461 | 0.552177 | 0.848202 | 0.853109 |
| LNC_003180 | XLOC_073647 | -- | intronic_lncRNA | 1.604037 | 0.303343 | 0.075087 | 0.365055 |
| LNC_003181 | XLOC_073648 | -- | intronic_lncRNA | 1.585102 | 0.600791 | 0.944641 | 0.761433 |
| LNC_003186 | XLOC_073655 | -- | lincRNA | 0 | 0 | 28.180424 | 0 |
| LNC_003187 | XLOC_073655 | -- | lincRNA | 0 | 0 | 8.656172 | 0 |
| LNC_003184 | XLOC_073654 | -- | intronic_lncRNA | 0.516223 | 0.401645 | 0.740711 | 0.400391 |
| LNC_003185 | XLOC_073655 | -- | lincRNA | 0 | 0.00525 | 5.732812 | 43.9692 |
| LNC_003188 | XLOC_073655 | -- | lincRNA | 0 | 0 | 14.273937 | 0 |
| LNC_003189 | XLOC_073655 | -- | lincRNA | 0 | 0 | 7.806875 | 0 |
| LNC_006299 | XLOC_151008 | -- | intronic_lncRNA | 0.323812 | 0.501917 | 0.11538 | 0.809814 |
| LNC_006298 | XLOC_150995 | -- | lincRNA | 0.00922 | 0.005652 | 0.401537 | 0.643829 |
| LNC_006258 | XLOC_150219 | -- | intronic_lncRNA | 0.679513 | 0.458419 | 0.301927 | 0.59428 |
| LNC_006291 | XLOC_150813 | -- | antisense_lncRNA | 0.209783 | 0.279012 | 0.403237 | 0.353864 |
| LNC_006290 | XLOC_150812 | -- | intronic_lncRNA | 0.764081 | 0.634268 | 0.899218 | 1.362589 |
| LNC_006293 | XLOC_150832 | -- | intronic_lncRNA | 0.79872 | 0.306869 | 0.685385 | 0.539491 |
| LNC_006292 | XLOC_150814 | -- | intronic_lncRNA | 0.704916 | 0.500381 | 1.232537 | 0.434271 |
| LNC_006295 | XLOC_150903 | -- | intronic_lncRNA | 0.374424 | 0.85397 | 0.684446 | 0.778602 |
| LNC_006294 | XLOC_150902 | -- | intronic_lncRNA | 0.173546 | 0.70292 | 0.294949 | 0.142868 |
| LNC_006297 | XLOC_150995 | -- | lincRNA | 0.442606 | 0.510247 | 0 | 0 |
| LNC_006296 | XLOC_150984 | -- | intronic_lncRNA | 0.643027 | 0.383686 | 1.638775 | 1.022257 |
| LNC_001023 | XLOC_022152 | -- | intronic_lncRNA | 0.017201 | 0 | 0 | 0.607887 |
| LNC_001022 | XLOC_022152 | -- | intronic_lncRNA | 0.677776 | 0.429551 | 0.808102 | 0.878275 |
| LNC_001021 | XLOC_022139 | -- | intronic_lncRNA | 0.835496 | 0.602756 | 0.490146 | 0.854367 |
| LNC_001020 | XLOC_022132 | -- | intronic_lncRNA | 0 | 0 | 0 | 0.464396 |
| LNC_001027 | XLOC_022299 | -- | intronic_lncRNA | 1.300125 | 0.524911 | 0.8175 | 0.675337 |
| LNC_001026 | XLOC_022223 | -- | intronic_lncRNA | 0.617194 | 0.26583 | 1.860715 | 1.056149 |
| LNC_001025 | XLOC_022221 | -- | intronic_lncRNA | 2.18085 | 1.992494 | 1.534897 | 1.535317 |
| LNC_001024 | XLOC_022157 | -- | intronic_lncRNA | 0.64778 | 0.433939 | 0.66551 | 0.739673 |
| LNC_001029 | XLOC_022375 | -- | intronic_lncRNA | 0.486286 | 0.123533 | 0.707565 | 0.445191 |
| LNC_001028 | XLOC_022364 | -- | intronic_lncRNA | 0 | 0.381845 | 0 | 0.312048 |
| LNC_004299 | XLOC_093534 | -- | intronic_lncRNA | 0.181021 | 0 | 0.328398 | 0.590519 |
| LNC_004298 | XLOC_093533 | -- | intronic_lncRNA | 0.439166 | 0 | 0.520254 | 0 |
| LNC_004293 | XLOC_093395 | -- | antisense_lncRNA | 0.232119 | 0.547327 | 0.53864 | 0.753203 |
| LNC_004292 | XLOC_093384 | -- | intronic_lncRNA | 0.370548 | 0.250969 | 0.659602 | 0.514431 |
| LNC_004291 | XLOC_093383 | -- | intronic_lncRNA | 0.403562 | 0 | 0.245557 | 1.183089 |
| LNC_004290 | XLOC_093375 | -- | intronic_lncRNA | 0.232519 | 1.269431 | 0.131095 | 0 |
| LNC_004297 | XLOC_093491 | -- | intronic_lncRNA | 0.248321 | 0.140775 | 0 | 0.58134 |
| LNC_004296 | XLOC_093487 | -- | intronic_lncRNA | 0.805287 | 0.423798 | 0.580122 | 0.529712 |
| LNC_004295 | XLOC_093458 | -- | intronic_lncRNA | 5.400447 | 3.375855 | 2.949595 | 3.912163 |
| LNC_004294 | XLOC_093458 | -- | intronic_lncRNA | 3.692311 | 24.068024 | 9.301426 | 11.151831 |
| LNC_001128 | XLOC_024742 | -- | intronic_lncRNA | 0.321602 | 0.383721 | 0.18075 | 0.840097 |
| LNC_001129 | XLOC_024744 | -- | intronic_lncRNA | 0.464725 | 0 | 0.220522 | 0.188987 |
| LNC_001126 | XLOC_024711 | -- | intronic_lncRNA | 0.580389 | 0 | 0.584027 | 0.840111 |
| LNC_001127 | XLOC_024741 | -- | intronic_lncRNA | 0.207093 | 0.098006 | 0 | 0.952916 |
| LNC_001124 | XLOC_024684 | -- | intronic_lncRNA | 0.158717 | 0.248073 | 0.52514 | 0.084946 |
| LNC_001125 | XLOC_024711 | -- | intronic_lncRNA | 0 | 0.896766 | 0 | 0.221006 |
| LNC_001122 | XLOC_024561 | -- | intronic_lncRNA | 0 | 0 | 0 | 0.451569 |
| LNC_001123 | XLOC_024682 | -- | intronic_lncRNA | 1.28078 | 2.201391 | 1.625962 | 2.044808 |
| LNC_001120 | XLOC_024288 | -- | lincRNA | 0.932576 | 0.127537 | 0.026168 | 0.045076 |
| LNC_001121 | XLOC_024312 | -- | antisense_lncRNA | 0.607817 | 0.849131 | 1.099085 | 0.302278 |
| LNC_003748 | XLOC_080332 | -- | intronic_lncRNA | 0.811204 | 0.566945 | 0.755912 | 1.122657 |
| LNC_003749 | XLOC_080334 | -- | intronic_lncRNA | 1.197966 | 2.551349 | 2.139142 | 1.829269 |
| LNC_003740 | XLOC_080289 | -- | intronic_lncRNA | 0.233685 | 0.746037 | 0.276449 | 0.203236 |
| LNC_003741 | XLOC_080290 | -- | intronic_lncRNA | 0.509909 | 0.084794 | 0.137343 | 0.141467 |
| LNC_003742 | XLOC_080302 | -- | lincRNA | 0.67675 | 0.119244 | 0 | 0.104994 |
| LNC_003743 | XLOC_080304 | -- | intronic_lncRNA | 1.852203 | 2.702022 | 2.136996 | 2.319421 |
| LNC_003744 | XLOC_080324 | -- | intronic_lncRNA | 0.159744 | 0.741358 | 0.479246 | 0.566637 |
| LNC_003745 | XLOC_080324 | -- | intronic_lncRNA | 0.47721 | 0.277493 | 0.395508 | 0.632447 |
| LNC_003746 | XLOC_080328 | -- | intronic_lncRNA | 0.677403 | 0.752153 | 0.162897 | 0.13534 |
| LNC_003747 | XLOC_080329 | -- | intronic_lncRNA | 1.207914 | 0 | 0.398062 | 1.11438 |
| LNC_000299 | XLOC_004300 | -- | intronic_lncRNA | 0.36745 | 0.996239 | 1.049813 | 0 |
| LNC_000298 | XLOC_004300 | -- | intronic_lncRNA | 1.646177 | 0.046367 | 0.444938 | 1.152642 |
| LNC_000297 | XLOC_004298 | -- | intronic_lncRNA | 0.689198 | 0.479117 | 0.505232 | 0.379995 |
| LNC_000296 | XLOC_004296 | -- | intronic_lncRNA | 0.741083 | 1.223891 | 1.593968 | 1.323942 |
| LNC_000295 | XLOC_004285 | -- | intronic_lncRNA | 0.73456 | 1.165656 | 1.443219 | 1.304552 |
| LNC_000294 | XLOC_004284 | -- | intronic_lncRNA | 0.66236 | 0.382589 | 0.830215 | 0.665636 |
| LNC_000293 | XLOC_004276 | -- | lincRNA | 0.393205 | 0.491486 | 0.040044 | 0.058587 |
| LNC_000292 | XLOC_004271 | -- | intronic_lncRNA | 0 | 0 | 0 | 0.81138 |
| LNC_000291 | XLOC_004271 | -- | intronic_lncRNA | 0.611616 | 0.385184 | 0.715976 | 0.06072 |
| LNC_000290 | XLOC_004266 | -- | intronic_lncRNA | 0.411578 | 0.414523 | 0.931204 | 0.245755 |
| LNC_002839 | XLOC_065458 | -- | lincRNA | 1.016514 | 0.39931 | 0.240547 | 0.485237 |

| LNC_002838 | XLOC_065454 | -- | lincRNA | 2.414631 | 1.658561 | 1.224121 | 1.294342 |
| --- | --- | --- | --- | --- | --- | --- | --- |
| LNC_002831 | XLOC_065428 | -- | lincRNA | 0 | 1.735482 | 0.830878 | 1.109514 |
| LNC_002830 | XLOC_065422 | -- | lincRNA | 2.955715 | 3.569207 | 0.411407 | 1.05997 |
| LNC_002833 | XLOC_065439 | -- | lincRNA | 0 | 3.848019 | 0 | 1.566784 |
| LNC_002832 | XLOC_065436 | -- | lincRNA | 0.708529 | 0 | 0 | 0.392669 |
| LNC_002835 | XLOC_065448 | -- | lincRNA | 0.126568 | 0.603265 | 0 | 0 |
| LNC_002834 | XLOC_065446 | -- | lincRNA | 1.867708 | 1.248432 | 2.20126 | 1.641992 |
| LNC_002837 | XLOC_065450 | -- | lincRNA | 3.53964 | 1.317864 | 1.881533 | 2.639247 |
| LNC_002836 | XLOC_065450 | -- | lincRNA | 0 | 1.422124 | 0 | 0.015895 |
| LNC_003407 | XLOC_075198 | -- | lincRNA | 1.47442 | 1.293158 | 0.375492 | 0.52709 |
| LNC_003406 | XLOC_075195 | -- | lincRNA | 1.467728 | 0.973154 | 0.328506 | 1.209192 |
| LNC_003405 | XLOC_075194 | -- | lincRNA | 3.137242 | 0.995818 | 0.234046 | 0.591498 |
| LNC_003404 | XLOC_075193 | -- | lincRNA | 0 | 0.705107 | 0.112168 | 0 |
| LNC_003403 | XLOC_075193 | -- | lincRNA | 4.518352 | 1.456397 | 0.922433 | 0.900019 |
| LNC_003402 | XLOC_075192 | -- | lincRNA | 3.406984 | 1.346033 | 0.356686 | 1.118184 |
| LNC_003401 | XLOC_075192 | -- | lincRNA | 0.725578 | 0 | 0.164586 | 0.101722 |
| LNC_003400 | XLOC_075192 | -- | lincRNA | 1.625172 | 0.626609 | 0.548215 | 0.603806 |
| LNC_003409 | XLOC_075199 | -- | lincRNA | 2.930239 | 1.193904 | 0.455502 | 0.900228 |
| LNC_003408 | XLOC_075199 | -- | lincRNA | 2.865765 | 2.177747 | 1.089594 | 1.310604 |
| LNC_006044 | XLOC_143930 | -- | intronic_lncRNA | 0.566759 | 0.562562 | 0.73745 | 0.798087 |
| LNC_006045 | XLOC_143930 | -- | intronic_lncRNA | 0.198582 | 0.400994 | 0.269055 | 0.412751 |
| LNC_006046 | XLOC_143934 | -- | intronic_lncRNA | 0.099169 | 0.192366 | 0.490172 | 0.390792 |
| LNC_006047 | XLOC_143944 | -- | intronic_lncRNA | 0.671009 | 0.110056 | 0.714522 | 0.55934 |
| LNC_006040 | XLOC_143790 | -- | lincRNA | 0 | 0 | 0.395061 | 0 |
| LNC_006041 | XLOC_143915 | -- | lincRNA | 0.568813 | 0 | 0 | 0 |
| LNC_006042 | XLOC_143924 | -- | intronic_lncRNA | 0.636078 | 0.566054 | 0.753588 | 0.793768 |
| LNC_006043 | XLOC_143929 | -- | intronic_lncRNA | 0.696164 | 0.860821 | 0.534841 | 0.610373 |
| LNC_006048 | XLOC_143949 | -- | intronic_lncRNA | 0.189424 | 0.305631 | 0.174543 | 0.679264 |
| LNC_006049 | XLOC_144035 | -- | antisense_lncRNA | 0.578809 | 0.22371 | 0.670028 | 0.434416 |
| LNC_004576 | XLOC_100522 | -- | intronic_lncRNA | 0.177683 | 0.184818 | 0.59461 | 0.284716 |
| LNC_004577 | XLOC_100526 | -- | intronic_lncRNA | 0.680042 | 0.239008 | 0.294031 | 0.611123 |
| LNC_004574 | XLOC_100513 | -- | intronic_lncRNA | 0 | 0.094645 | 0.47732 | 0.067924 |
| LNC_004575 | XLOC_100514 | -- | intronic_lncRNA | 0.158878 | 0.526604 | 0.228571 | 0 |
| LNC_004572 | XLOC_100483 | -- | intronic_lncRNA | 0 | 0.43008 | 0.547335 | 0.198996 |
| LNC_004573 | XLOC_100512 | -- | intronic_lncRNA | 0 | 0.484075 | 0 | 0.217758 |
| LNC_004570 | XLOC_100409 | -- | intronic_lncRNA | 0.344323 | 0.727147 | 0.706974 | 0.671169 |
| LNC_004571 | XLOC_100410 | -- | lincRNA | 0 | 0.31742 | 0.179641 | 0.723144 |
| LNC_004578 | XLOC_100527 | -- | intronic_lncRNA | 0 | 0.637749 | 0 | 0 |
| LNC_004579 | XLOC_100527 | -- | intronic_lncRNA | 0.121805 | 0.182963 | 0.420815 | 0.518521 |
| LNC_000509 | XLOC_008963 | -- | intronic_lncRNA | 0.633256 | 0.120754 | 0.376557 | 0.266955 |
| LNC_000508 | XLOC_008956 | -- | antisense_lncRNA | 0.672809 | 0.586021 | 0.565552 | 0.471324 |
| LNC_005027 | XLOC_114723 | -- | intronic_lncRNA | 1.564135 | 1.393324 | 1.471729 | 1.300731 |
| LNC_000506 | XLOC_008931 | -- | intronic_lncRNA | 0.407937 | 0.362423 | 0.847242 | 0.95732 |
| LNC_005025 | XLOC_114698 | -- | intronic_lncRNA | 0.519559 | 0.630152 | 0 | 0.555572 |
| LNC_000504 | XLOC_008915 | -- | intronic_lncRNA | 0.699392 | 0.343783 | 0.443322 | 1.129985 |
| LNC_005023 | XLOC_114697 | -- | intronic_lncRNA | 0.707605 | 0 | 0.913577 | 1.016702 |
| LNC_000502 | XLOC_008798 | -- | intronic_lncRNA | 0.515646 | 0.176443 | 0.159168 | 0.621481 |
| LNC_000501 | XLOC_008795 | -- | intronic_lncRNA | 0.595285 | 0.589529 | 0.447125 | 0.417708 |
| LNC_000500 | XLOC_008789 | -- | intronic_lncRNA | 0.398831 | 0.127008 | 0.161137 | 0.16084 |
| LNC_004990 | XLOC_113677 | -- | intronic_lncRNA | 0.405707 | 0.201056 | 1.068008 | 1.083616 |
| LNC_004991 | XLOC_113697 | -- | intronic_lncRNA | 0.600079 | 0.804736 | 1.076195 | 0.425155 |
| LNC_004992 | XLOC_113697 | -- | intronic_lncRNA | 0.18901 | 0.023153 | 0.209913 | 1.067203 |
| LNC_004993 | XLOC_113707 | -- | intronic_lncRNA | 1.120429 | 1.402913 | 1.248058 | 0.387978 |
| LNC_004994 | XLOC_113719 | -- | intronic_lncRNA | 0.92211 | 0.455491 | 0.067509 | 0.500153 |
| LNC_004995 | XLOC_113719 | -- | intronic_lncRNA | 0 | 0 | 0.936715 | 0.557861 |
| LNC_004996 | XLOC_113736 | -- | intronic_lncRNA | 0.552838 | 0.351191 | 0.415336 | 0.622191 |
| LNC_004997 | XLOC_113739 | -- | antisense_lncRNA | 0.214298 | 0.072413 | 0.875161 | 0.350082 |
| LNC_004998 | XLOC_113880 | -- | lincRNA | 1.351954 | 0.737545 | 0 | 0.664295 |
| LNC_004999 | XLOC_113978 | -- | intronic_lncRNA | 0.226613 | 0 | 0.57251 | 0.500454 |
| LNC_003977 | XLOC_085839 | -- | intronic_lncRNA | 0 | 0.002581 | 0.597153 | 0.00752 |
| LNC_003976 | XLOC_085835 | -- | antisense_lncRNA | 0.642479 | 0 | 0 | 0 |
| LNC_003975 | XLOC_085775 | -- | intronic_lncRNA | 0.649832 | 1.079602 | 1.436037 | 1.337328 |
| LNC_003974 | XLOC_085755 | -- | lincRNA | 0.817631 | 0.242266 | 0.346749 | 0.618554 |
| LNC_003973 | XLOC_085721 | -- | intronic_lncRNA | 0 | 0.391707 | 0 | 0 |
| LNC_003972 | XLOC_085718 | -- | intronic_lncRNA | 0 | 0.269935 | 0.27993 | 0 |
| LNC_003971 | XLOC_085649 | -- | intronic_lncRNA | 0.466029 | 0.427251 | 0.619924 | 0.650392 |
| LNC_003604 | XLOC_076606 | -- | intronic_lncRNA | 0.563875 | 0.337354 | 0.499666 | 1.064786 |
| LNC_003979 | XLOC_085867 | -- | intronic_lncRNA | 0 | 0.879159 | 0 | 0 |
| LNC_003978 | XLOC_085867 | -- | intronic_lncRNA | 0.69688 | 0.065819 | 0.816278 | 0.695364 |
| LNC_002624 | XLOC_060788 | -- | intronic_lncRNA | 0.831774 | 0.585351 | 0.555993 | 0.608815 |
| LNC_005038 | XLOC_115079 | -- | intronic_lncRNA | 0.987713 | 1.514565 | 1.248902 | 0.808224 |
| LNC_002626 | XLOC_060804 | -- | intronic_lncRNA | 6.515679 | 2.778588 | 7.82929 | 8.240614 |
| LNC_002627 | XLOC_060875 | -- | lincRNA | 0.386243 | 0.09909 | 0 | 0.497283 |
| LNC_002620 | XLOC_060518 | -- | intronic_lncRNA | 0.080682 | 0.004496 | 0.311234 | 0.283617 |
| LNC_002621 | XLOC_060519 | -- | intronic_lncRNA | 0 | 0.577108 | 0 | 0.138612 |
| LNC_002622 | XLOC_060647 | -- | intronic_lncRNA | 1.103272 | 0.085471 | 0.415208 | 0.76657 |
| LNC_005039 | XLOC_115090 | -- | intronic_lncRNA | 0.036211 | 0.777563 | 0.146802 | 0.079196 |

| LNC_002628 | XLOC_060881 | -- | intronic_lncRNA | 1.154094 | 0.680693 | 0.934411 | 0.765714 |
| --- | --- | --- | --- | --- | --- | --- | --- |
| LNC_002629 | XLOC_060881 | -- | intronic_lncRNA | 0.554642 | 0.797229 | 1.425849 | 1.93708 |
| LNC_005294 | XLOC_121247 | -- | intronic_lncRNA | 0.822817 | 0.429929 | 0.204501 | 0.515309 |
| LNC_005295 | XLOC_121248 | -- | intronic_lncRNA | 0.912338 | 0.727264 | 0 | 0.436753 |
| LNC_005030 | XLOC_114782 | -- | intronic_lncRNA | 0.546015 | 0.628605 | 0.505462 | 0.847871 |
| LNC_005031 | XLOC_114839 | -- | intronic_lncRNA | 0.584518 | 0.593803 | 0.63834 | 0.190327 |
| LNC_000512 | XLOC_009029 | -- | intronic_lncRNA | 0.552741 | 0.339075 | 0.419888 | 0.537483 |
| LNC_000513 | XLOC_009034 | -- | intronic_lncRNA | 1.050549 | 0.145613 | 0.546631 | 0.301535 |
| LNC_004378 | XLOC_095318 | -- | intronic_lncRNA | 0.528178 | 0.117324 | 0.495931 | 0.415399 |
| LNC_004379 | XLOC_095334 | -- | intronic_lncRNA | 0.818409 | 0.55547 | 0.479412 | 0.766608 |
| LNC_004374 | XLOC_095315 | -- | intronic_lncRNA | 0.08618 | 0 | 0.610157 | 0 |
| LNC_000514 | XLOC_009042 | -- | intronic_lncRNA | 0.621857 | 1.053365 | 1.996375 | 1.634094 |
| LNC_004376 | XLOC_095316 | -- | intronic_lncRNA | 0.657818 | 0.611251 | 0.282598 | 0.626529 |
| LNC_004377 | XLOC_095317 | -- | intronic_lncRNA | 0.513462 | 0.384071 | 0.638348 | 0.73552 |
| LNC_004370 | XLOC_095313 | -- | intronic_lncRNA | 0.085991 | 0 | 0.317642 | 0.555777 |
| LNC_004371 | XLOC_095314 | -- | intronic_lncRNA | 0.177204 | 0.35385 | 0.572564 | 0.708939 |
| LNC_004372 | XLOC_095315 | -- | intronic_lncRNA | 0.261575 | 0.249304 | 0.098703 | 0.559179 |
| LNC_004373 | XLOC_095315 | -- | intronic_lncRNA | 0 | 0.508766 | 0 | 0 |
| LNC_000516 | XLOC_009046 | -- | intronic_lncRNA | 0.665961 | 0.867914 | 1.042805 | 0.896166 |
| LNC_005037 | XLOC_114967 | -- | lincRNA | 0 | 0 | 0.622864 | 1.200024 |
| LNC_006314 | XLOC_151524 | -- | intronic_lncRNA | 0 | 0.981768 | 1.055254 | 1.444281 |
| LNC_001829 | XLOC_040969 | -- | lincRNA | 0.699647 | 0.229246 | 0.127062 | 0.385266 |
| LNC_001828 | XLOC_040968 | -- | lincRNA | 0 | 0.709332 | 0.065878 | 0.392108 |
| LNC_006315 | XLOC_151549 | -- | intronic_lncRNA | 0.798956 | 0.26285 | 0.915737 | 0.667218 |
| LNC_001823 | XLOC_040926 | -- | intronic_lncRNA | 0.622783 | 0.272465 | 0.733359 | 0.286244 |
| LNC_001822 | XLOC_040924 | -- | intronic_lncRNA | 0.339258 | 0.553748 | 0 | 0.598452 |
| LNC_001821 | XLOC_040924 | -- | intronic_lncRNA | 0.410794 | 0.141107 | 0.882027 | 0.453619 |
| LNC_001820 | XLOC_040923 | -- | intronic_lncRNA | 0 | 0.248845 | 0.574477 | 0.219085 |
| LNC_001827 | XLOC_040955 | -- | intronic_lncRNA | 0.752693 | 0.510069 | 1.921848 | 2.043808 |
| LNC_001826 | XLOC_040943 | -- | intronic_lncRNA | 0 | 0 | 0.418952 | 0 |
| LNC_001825 | XLOC_040928 | -- | intronic_lncRNA | 0.304844 | 0.062031 | 0.300918 | 0.476831 |
| LNC_001824 | XLOC_040927 | -- | intronic_lncRNA | 0.657043 | 0.213496 | 0.468152 | 0.147692 |
| LNC_006317 | XLOC_151554 | -- | intronic_lncRNA | 1.280502 | 0.535508 | 0.26535 | 0.526508 |
| LNC_006310 | XLOC_151462 | -- | lincRNA | 0.601341 | 0 | 0 | 0 |
| LNC_006311 | XLOC_151497 | -- | intronic_lncRNA | 3.285195 | 6.412509 | 4.020706 | 5.37617 |
| LNC_006312 | XLOC_151524 | -- | intronic_lncRNA | 1.407726 | 1.893389 | 0.973152 | 1.356103 |
| LNC_006313 | XLOC_151524 | -- | intronic_lncRNA | 0 | 0 | 0.956039 | 0 |
| LNC_004749 | XLOC_106046 | -- | intronic_lncRNA | 0.579526 | 0.395847 | 0.740378 | 0.610472 |
| LNC_004748 | XLOC_106036 | -- | intronic_lncRNA | 0.804637 | 0.907638 | 2.543453 | 1.169754 |
| LNC_004745 | XLOC_106016 | -- | intronic_lncRNA | 0.509909 | 0 | 0 | 0 |
| LNC_004744 | XLOC_105942 | -- | intronic_lncRNA | 2.214181 | 1.740947 | 2.316612 | 2.676956 |
| LNC_004747 | XLOC_106035 | -- | intronic_lncRNA | 0.605188 | 1.117695 | 0 | 0.681747 |
| LNC_004746 | XLOC_106017 | -- | intronic_lncRNA | 0.521342 | 0.157034 | 0 | 0 |
| LNC_004741 | XLOC_105918 | -- | intronic_lncRNA | 0.243099 | 0.718596 | 0.543671 | 0.314923 |
| LNC_002305 | XLOC_052949 | -- | intronic_lncRNA | 0.722456 | 0.33307 | 0.785067 | 1.000675 |
| LNC_004743 | XLOC_105937 | -- | intronic_lncRNA | 0.675169 | 0.484266 | 1.38436 | 0.864031 |
| LNC_004742 | XLOC_105936 | -- | lincRNA | 0.615578 | 0 | 0 | 0 |
| LNC_006318 | XLOC_151709 | -- | lincRNA | 0.294786 | 1.024411 | 0.307713 | 0.198576 |
| LNC_006319 | XLOC_151710 | -- | lincRNA | 0.608584 | 1.91029 | 0.308255 | 0.268568 |
| LNC_005337 | XLOC_122147 | -- | intronic_lncRNA | 1.146628 | 0.750021 | 1.733709 | 2.167815 |
| LNC_000088 | XLOC_000619 | -- | lincRNA | 0 | 0 | 4.257102 | 0 |
| LNC_000089 | XLOC_000619 | -- | lincRNA | 2.946868 | 12.64339 | 9.683927 | 1.271119 |
| LNC_000082 | XLOC_000619 | -- | lincRNA | 1.34633 | 0.621405 | 0 | 0.007029 |
| LNC_000083 | XLOC_000619 | -- | lincRNA | 17.263727 | 7.471503 | 10.426149 | 7.643737 |
| LNC_000080 | XLOC_000619 | -- | lincRNA | 18.174623 | 8.140789 | 9.006227 | 8.279202 |
| LNC_000081 | XLOC_000619 | -- | lincRNA | 0 | 13.977008 | 6.054397 | 13.063881 |
| LNC_000086 | XLOC_000619 | -- | lincRNA | 43.407307 | 9.811454 | 0.009625 | 16.902472 |
| LNC_000087 | XLOC_000619 | -- | lincRNA | 19.905333 | 3.789702 | 29.054279 | 25.549448 |
| LNC_000084 | XLOC_000619 | -- | lincRNA | 0 | 8.899432 | 3.131568 | 0 |
| LNC_000085 | XLOC_000619 | -- | lincRNA | 0 | 0 | 5.368405 | 0 |
| LNC_005336 | XLOC_122141 | -- | intronic_lncRNA | 0.337676 | 0.764983 | 0.422066 | 0.725071 |
| LNC_002022 | XLOC_045406 | -- | antisense_lncRNA | 0.094674 | 0.17192 | 0.063358 | 0.507196 |
| LNC_002023 | XLOC_045415 | -- | intronic_lncRNA | 1.005246 | 3.245381 | 5.175816 | 3.623231 |
| LNC_002020 | XLOC_045406 | -- | intronic_lncRNA | 0.013088 | 0.093312 | 0.364452 | 0 |
| LNC_002021 | XLOC_045406 | -- | antisense_lncRNA | 0.452623 | 0.494855 | 0.751772 | 0.820666 |
| LNC_002026 | XLOC_045426 | -- | intronic_lncRNA | 0 | 0.387886 | 0.689705 | 0.517784 |
| LNC_002027 | XLOC_045429 | -- | intronic_lncRNA | 0.696297 | 0.520111 | 0.723175 | 0.805685 |
| LNC_002024 | XLOC_045423 | -- | intronic_lncRNA | 0 | 0.418264 | 0.092123 | 0.331577 |
| LNC_002025 | XLOC_045424 | -- | intronic_lncRNA | 0 | 0 | 0.457512 | 0.457434 |
| LNC_002028 | XLOC_045469 | -- | intronic_lncRNA | 0.302106 | 0.367797 | 0.118897 | 0.608737 |
| LNC_002029 | XLOC_045470 | -- | intronic_lncRNA | 0.424558 | 0 | 0.276157 | 0.415704 |
| LNC_003212 | XLOC_074356 | -- | intronic_lncRNA | 0.773452 | 0.376844 | 0.685813 | 0.53887 |
| LNC_003213 | XLOC_074390 | -- | lincRNA | 0.984971 | 0.480119 | 0.107126 | 0.29822 |
| LNC_003210 | XLOC_074227 | -- | intronic_lncRNA | 0 | 0 | 0.434919 | 0 |
| LNC_003211 | XLOC_074238 | -- | lincRNA | 1.299243 | 0.309099 | 0.978049 | 1.229409 |
| LNC_003216 | XLOC_074537 | -- | lincRNA | 1.009221 | 0.205285 | 0.075034 | 0.17229 |
| LNC_003217 | XLOC_074565 | -- | intronic_lncRNA | 0 | 0.961315 | 0.173006 | 0 |

| LNC_003214 | XLOC_074467 | -- | intronic_lncRNA | 5.783774 | 3.586497 | 4.365574 | 4.598819 |
| --- | --- | --- | --- | --- | --- | --- | --- |
| LNC_003215 | XLOC_074536 | -- | lincRNA | 0.521195 | 0.161966 | 0.228187 | 0.021842 |
| LNC_003218 | XLOC_074649 | -- | intronic_lncRNA | 1.599121 | 1.266652 | 0.43192 | 0.64165 |
| LNC_003219 | XLOC_074729 | -- | intronic_lncRNA | 0.141684 | 0.102813 | 0.288098 | 0.774558 |
| LNC_000882 | XLOC_019168 | -- | intronic_lncRNA | 0 | 0 | 0 | 0.869688 |
| LNC_000883 | XLOC_019272 | -- | intronic_lncRNA | 0.508659 | 0.059732 | 0.734158 | 0.352022 |
| LNC_000880 | XLOC_019127 | -- | intronic_lncRNA | 0.796433 | 0.902723 | 0.862468 | 1.007182 |
| LNC_000881 | XLOC_019151 | -- | intronic_lncRNA | 0.57178 | 0.635149 | 0.497225 | 0.488143 |
| LNC_000886 | XLOC_019302 | -- | intronic_lncRNA | 0.708933 | 0.757381 | 0.402518 | 0.136558 |
| LNC_000887 | XLOC_019303 | -- | intronic_lncRNA | 0.574814 | 0.519245 | 0.221737 | 0.168327 |
| LNC_000884 | XLOC_019279 | -- | intronic_lncRNA | 0 | 0 | 0.456032 | 0.174022 |
| LNC_000885 | XLOC_019301 | -- | intronic_lncRNA | 0.557357 | 0.481923 | 0.409178 | 0.136293 |
| LNC_000888 | XLOC_019305 | -- | intronic_lncRNA | 0.550114 | 0.41365 | 0.676326 | 0.90458 |
| LNC_000889 | XLOC_019313 | -- | intronic_lncRNA | 1.645572 | 1.510911 | 1.937507 | 0.640625 |
| LNC_006459 | XLOC_155314 | -- | intronic_lncRNA | 1.755109 | 0.996089 | 0.914773 | 1.445867 |
| LNC_006458 | XLOC_155302 | -- | intronic_lncRNA | 0.711875 | 0.239178 | 0.568716 | 0.215941 |
| LNC_005904 | XLOC_139127 | -- | lincRNA | 4.798926 | 0 | 1.207073 | 0 |
| LNC_005905 | XLOC_139132 | -- | intronic_lncRNA | 0.492269 | 0 | 0.380848 | 0 |
| LNC_005906 | XLOC_139166 | -- | intronic_lncRNA | 0.304764 | 0.460388 | 0.442391 | 0.475437 |
| LNC_005907 | XLOC_139228 | -- | intronic_lncRNA | 0.121732 | 0.683585 | 0.568403 | 0.681933 |
| LNC_005900 | XLOC_138903 | -- | intronic_lncRNA | 0.169545 | 0 | 0 | 0.429541 |
| LNC_005901 | XLOC_138911 | -- | intronic_lncRNA | 1.477138 | 1.095543 | 1.06503 | 1.754857 |
| LNC_005902 | XLOC_139071 | -- | intronic_lncRNA | 0.810982 | 0.1536 | 0.373017 | 0.561668 |
| LNC_005903 | XLOC_139112 | -- | intronic_lncRNA | 1.006604 | 0.522729 | 1.372868 | 1.662881 |
| LNC_005908 | XLOC_139335 | -- | lincRNA | 0.396271 | 0.333969 | 0.115495 | 0.356728 |
| LNC_005909 | XLOC_139452 | -- | intronic_lncRNA | 0.556349 | 0 | 0.872646 | 0 |
| LNC_001593 | XLOC_034655 | -- | lincRNA | 0.99832 | 0.317973 | 0.746429 | 0.114484 |
| LNC_001592 | XLOC_034623 | -- | intronic_lncRNA | 0.766584 | 0.383171 | 2.272999 | 2.629395 |
| LNC_001591 | XLOC_034476 | -- | intronic_lncRNA | 0.530278 | 0.512391 | 0.370038 | 0.426972 |
| LNC_001590 | XLOC_034476 | -- | intronic_lncRNA | 0.041807 | 0.346978 | 0.214299 | 0.363966 |
| LNC_001597 | XLOC_034725 | -- | lincRNA | 0.519014 | 0 | 0 | 0 |
| LNC_001596 | XLOC_034712 | -- | intronic_lncRNA | 0.823503 | 1.209607 | 1.154106 | 1.376545 |
| LNC_001595 | XLOC_034662 | -- | lincRNA | 3.26609 | 0 | 1.45071 | 2.014676 |
| LNC_001594 | XLOC_034655 | -- | lincRNA | 0 | 0.656845 | 0.488663 | 1.095197 |
| LNC_001599 | XLOC_034765 | -- | antisense_lncRNA | 0.068072 | 0.403296 | 0.811647 | 1.080888 |
| LNC_001598 | XLOC_034726 | -- | lincRNA | 0.311875 | 0.487129 | 0 | 0.148629 |
| LNC_006170 | XLOC_147840 | -- | intronic_lncRNA | 0.901752 | 0 | 0 | 0 |
| LNC_006171 | XLOC_147840 | -- | antisense_lncRNA | 0 | 0 | 0.925118 | 0 |
| LNC_006172 | XLOC_147863 | -- | intronic_lncRNA | 0 | 0.170586 | 0.324654 | 0.414381 |
| LNC_006173 | XLOC_147867 | -- | intronic_lncRNA | 0.391795 | 0.29293 | 0.242513 | 0.52647 |
| LNC_006174 | XLOC_147913 | -- | lincRNA | 0.891747 | 0.339389 | 0.100376 | 0.257339 |
| LNC_006175 | XLOC_147914 | -- | intronic_lncRNA | 1.399571 | 1.328703 | 1.152904 | 1.542043 |
| LNC_006176 | XLOC_147919 | -- | lincRNA | 0.152973 | 0 | 0.658971 | 0.164508 |
| LNC_006177 | XLOC_147935 | -- | intronic_lncRNA | 0.828206 | 0 | 0 | 0.26771 |
| LNC_001045 | XLOC_022583 | -- | lincRNA | 0 | 0 | 0 | 0.425006 |
| LNC_001044 | XLOC_022576 | -- | intronic_lncRNA | 0.555918 | 0.27956 | 0.623235 | 0.680758 |
| LNC_001047 | XLOC_022648 | -- | intronic_lncRNA | 0.590918 | 0.107331 | 0.913885 | 1.112419 |
| LNC_001046 | XLOC_022648 | -- | intronic_lncRNA | 0 | 1.710663 | 0 | 0 |
| LNC_001041 | XLOC_022556 | -- | intronic_lncRNA | 1.015401 | 0.714777 | 0.547011 | 0.609423 |
| LNC_001040 | XLOC_022495 | -- | intronic_lncRNA | 0.556264 | 0.384504 | 0.224743 | 0.142572 |
| LNC_001043 | XLOC_022574 | -- | intronic_lncRNA | 0.329837 | 0.387696 | 0.435397 | 0.482302 |
| LNC_001042 | XLOC_022556 | -- | intronic_lncRNA | 0.6262 | 0.090108 | 0.136977 | 0.034767 |
| LNC_001049 | XLOC_022697 | -- | intronic_lncRNA | 0.616381 | 0.26414 | 0.318178 | 0.395332 |
| LNC_001048 | XLOC_022648 | -- | intronic_lncRNA | 1.822241 | 0.846836 | 1.987977 | 2.333132 |
| LNC_001100 | XLOC_023631 | -- | intronic_lncRNA | 0.256922 | 0.505573 | 0.729044 | 0.675464 |
| LNC_001101 | XLOC_023729 | -- | intronic_lncRNA | 0.434885 | 0 | 0 | 0.673802 |
| LNC_001102 | XLOC_023730 | -- | intronic_lncRNA | 0.207606 | 0 | 0.48276 | 0.55834 |
| LNC_001103 | XLOC_023734 | -- | intronic_lncRNA | 0.536721 | 0 | 0 | 0.440969 |
| LNC_001104 | XLOC_023735 | -- | intronic_lncRNA | 0.273165 | 0.109021 | 0.529751 | 0.33398 |
| LNC_001105 | XLOC_023763 | -- | intronic_lncRNA | 0.478652 | 0.212257 | 0.171899 | 0.178308 |
| LNC_001106 | XLOC_023769 | -- | intronic_lncRNA | 0 | 0.133026 | 0.749817 | 0.860632 |
| LNC_001107 | XLOC_023770 | -- | intronic_lncRNA | 0.744537 | 0 | 0.302954 | 0.402082 |
| LNC_001108 | XLOC_023775 | -- | intronic_lncRNA | 0.8687 | 0.555017 | 0 | 1.033639 |
| LNC_001109 | XLOC_023803 | -- | intronic_lncRNA | 1.791833 | 1.02805 | 1.531253 | 1.723024 |
| LNC_000318 | XLOC_004581 | -- | intronic_lncRNA | 0.757893 | 0.184008 | 1.050847 | 0.911002 |
| LNC_000319 | XLOC_004589 | -- | intronic_lncRNA | 0 | 0.641415 | 0 | 0 |
| LNC_000312 | XLOC_004481 | -- | intronic_lncRNA | 0.618901 | 0.137781 | 0.50381 | 0.78643 |
| LNC_000313 | XLOC_004504 | -- | lincRNA | 0.668889 | 0.080249 | 0 | 0 |
| LNC_000310 | XLOC_004461 | -- | intronic_lncRNA | 0.92682 | 1.218996 | 1.326053 | 0.756564 |
| LNC_000311 | XLOC_004476 | -- | intronic_lncRNA | 0.829868 | 0.769446 | 0.800551 | 0.732938 |
| LNC_000316 | XLOC_004526 | -- | intronic_lncRNA | 1.068885 | 0.012642 | 0.752264 | 1.27956 |
| LNC_000317 | XLOC_004573 | -- | intronic_lncRNA | 0.710387 | 0.904954 | 0.497404 | 0.49149 |
| LNC_000314 | XLOC_004506 | -- | intronic_lncRNA | 2.845238 | 0.631343 | 0.198957 | 1.464538 |
| LNC_000315 | XLOC_004526 | -- | intronic_lncRNA | 2.384293 | 3.334428 | 2.229351 | 1.139077 |
| LNC_000271 | XLOC_003944 | -- | intronic_lncRNA | 0.991993 | 0.685677 | 1.163042 | 1.436103 |
| LNC_005350 | XLOC_122813 | -- | intronic_lncRNA | 0.726883 | 0.756855 | 0.900236 | 0 |
| LNC_005353 | XLOC_123142 | -- | lincRNA | 0 | 0 | 0 | 0.441115 |

| LNC_005352 | XLOC_123079 | -- | intronic_lncRNA | 0.553616 | 0 | 0.261449 | 0 |
| --- | --- | --- | --- | --- | --- | --- | --- |
| LNC_005355 | XLOC_123171 | -- | intronic_lncRNA | 0.379976 | 0.333361 | 0.504277 | 0.559598 |
| LNC_005354 | XLOC_123158 | -- | intronic_lncRNA | 0.793944 | 0.413426 | 0.637691 | 0.512118 |
| LNC_000277 | XLOC_004009 | -- | antisense_lncRNA | 0.091422 | 0.140597 | 0.381536 | 0.084771 |
| LNC_000276 | XLOC_004008 | -- | intronic_lncRNA | 0 | 0.07614 | 0.451688 | 0.245902 |
| LNC_000279 | XLOC_004010 | -- | intronic_lncRNA | 0.683928 | 0.23888 | 0.200632 | 0.057372 |
| LNC_005358 | XLOC_123197 | -- | intronic_lncRNA | 1.135621 | 2.258379 | 0.424236 | 0.660631 |
| LNC_002813 | XLOC_065373 | -- | lincRNA | 2.128808 | 2.207279 | 0.076464 | 1.300408 |
| LNC_002812 | XLOC_065372 | -- | lincRNA | 0.968483 | 0.372494 | 0.737975 | 1.439871 |
| LNC_002811 | XLOC_065370 | -- | lincRNA | 0.589747 | 0.249997 | 0.14175 | 0.650943 |
| LNC_002810 | XLOC_065367 | -- | lincRNA | 1.322609 | 1.732722 | 0.224591 | 0.513665 |
| LNC_002817 | XLOC_065378 | -- | lincRNA | 0.848078 | 0.403351 | 0.063314 | 0.232321 |
| LNC_002816 | XLOC_065377 | -- | lincRNA | 1.59046 | 1.491464 | 1.096896 | 1.793058 |
| LNC_002815 | XLOC_065376 | -- | lincRNA | 0.411721 | 0.244007 | 0.302624 | 0.795338 |
| LNC_002814 | XLOC_065375 | -- | lincRNA | 0.56586 | 1.227208 | 0.23405 | 1.013637 |
| LNC_002819 | XLOC_065381 | -- | lincRNA | 0.503189 | 0.337205 | 0.25549 | 0.882228 |
| LNC_002818 | XLOC_065380 | -- | lincRNA | 1.434937 | 0.706396 | 0 | 1.810962 |
| LNC_005689 | XLOC_133168 | -- | intronic_lncRNA | 0.330069 | 0.199998 | 0.175737 | 0.476109 |
| LNC_005688 | XLOC_133122 | -- | intronic_lncRNA | 0.070638 | 0.135101 | 0.443432 | 0.407088 |
| LNC_005683 | XLOC_132804 | -- | intronic_lncRNA | 1.064677 | 0.266569 | 1.033902 | 0.527562 |
| LNC_005682 | XLOC_132802 | -- | intronic_lncRNA | 0.761292 | 0.658503 | 0.098545 | 0 |
| LNC_005681 | XLOC_132798 | -- | intronic_lncRNA | 0.202159 | 0.573294 | 0 | 0 |
| LNC_005680 | XLOC_132785 | -- | intronic_lncRNA | 0.292013 | 0.266073 | 0 | 0.56696 |
| LNC_005687 | XLOC_132985 | -- | intronic_lncRNA | 0.6278 | 1.14212 | 0.776592 | 0.428175 |
| LNC_005686 | XLOC_132880 | -- | intronic_lncRNA | 1.024552 | 2.649935 | 0.669813 | 1.041122 |
| LNC_005685 | XLOC_132880 | -- | intronic_lncRNA | 0.007096 | 0 | 0 | 0.60469 |
| LNC_005684 | XLOC_132880 | -- | intronic_lncRNA | 0.771574 | 1.134196 | 1.576527 | 1.133642 |
| LNC_004086 | XLOC_088500 | -- | intronic_lncRNA | 0.082601 | 0.430608 | 0.454389 | 0.384688 |
| LNC_004087 | XLOC_088551 | -- | intronic_lncRNA | 0.125822 | 0.236014 | 0.406678 | 0 |
| LNC_004084 | XLOC_088445 | -- | intronic_lncRNA | 0.242648 | 0.36133 | 0.426593 | 0.542912 |
| LNC_004085 | XLOC_088488 | -- | lincRNA | 0.355981 | 0.089093 | 0.428586 | 0.071419 |
| LNC_004082 | XLOC_088421 | -- | intronic_lncRNA | 0.691865 | 0.322577 | 0.928802 | 0.378735 |
| LNC_004083 | XLOC_088437 | -- | intronic_lncRNA | 0.980053 | 0.457798 | 0.90043 | 1.051341 |
| LNC_004080 | XLOC_088332 | -- | intronic_lncRNA | 0.454878 | 0.507149 | 0.497042 | 0.640741 |
| LNC_004081 | XLOC_088416 | -- | intronic_lncRNA | 0 | 0 | 0 | 0.716199 |
| LNC_004088 | XLOC_088552 | -- | intronic_lncRNA | 0 | 0 | 0 | 0.562739 |
| LNC_004089 | XLOC_088569 | -- | intronic_lncRNA | 0.300955 | 0 | 0.331464 | 0.902071 |
| LNC_006028 | XLOC_143442 | -- | intronic_lncRNA | 0.330523 | 0.599514 | 0.253189 | 0.471655 |
| LNC_006029 | XLOC_143459 | -- | intronic_lncRNA | 0.116402 | 0 | 0.620581 | 0.09243 |
| LNC_006026 | XLOC_143441 | -- | intronic_lncRNA | 0.443689 | 0.565374 | 0.681311 | 0 |
| LNC_006027 | XLOC_143441 | -- | intronic_lncRNA | 0.321111 | 0.702849 | 0 | 0.364654 |
| LNC_006024 | XLOC_143414 | -- | intronic_lncRNA | 0.082383 | 0.317615 | 0.065826 | 0.479216 |
| LNC_006025 | XLOC_143440 | -- | intronic_lncRNA | 8.957341 | 0.514386 | 0.124329 | 0.086568 |
| LNC_006022 | XLOC_143376 | -- | intronic_lncRNA | 0.627043 | 0 | 0.272013 | 0.510572 |
| LNC_006023 | XLOC_143377 | -- | intronic_lncRNA | 0.806789 | 0.338612 | 0.688858 | 0.327735 |
| LNC_006020 | XLOC_143373 | -- | intronic_lncRNA | 0.050991 | 0.037416 | 0.054776 | 0.612993 |
| LNC_006021 | XLOC_143376 | -- | intronic_lncRNA | 0 | 0.206178 | 0.432411 | 0.294432 |
| LNC_004558 | XLOC_100094 | -- | intronic_lncRNA | 0.125279 | 0 | 0.394127 | 0.708496 |
| LNC_004559 | XLOC_100249 | -- | intronic_lncRNA | 0.14178 | 0.495116 | 0 | 0.114544 |
| LNC_004550 | XLOC_100044 | -- | intronic_lncRNA | 3.418604 | 1.574718 | 2.332994 | 4.147326 |
| LNC_004551 | XLOC_100044 | -- | intronic_lncRNA | 1.950555 | 1.084988 | 0.950209 | 0.937763 |
| LNC_004552 | XLOC_100044 | -- | intronic_lncRNA | 0.326372 | 0.458606 | 0.084822 | 0.799196 |
| LNC_004553 | XLOC_100044 | -- | intronic_lncRNA | 1.135873 | 0.93521 | 1.398952 | 1.071663 |
| LNC_004554 | XLOC_100044 | -- | intronic_lncRNA | 1.810123 | 0.584616 | 0.885165 | 1.120715 |
| LNC_004555 | XLOC_100067 | -- | intronic_lncRNA | 1.406957 | 1.13103 | 1.64382 | 1.535542 |
| LNC_004556 | XLOC_100092 | -- | intronic_lncRNA | 0.812185 | 0 | 0.103893 | 0 |
| LNC_004557 | XLOC_100093 | -- | intronic_lncRNA | 0 | 0 | 0.713373 | 0 |
| LNC_001690 | XLOC_037016 | -- | intronic_lncRNA | 0.417261 | 0.113003 | 0.332548 | 0.829572 |
| LNC_001691 | XLOC_037028 | -- | intronic_lncRNA | 0.876053 | 0.310857 | 0.428696 | 1.117222 |
| LNC_001692 | XLOC_037029 | -- | intronic_lncRNA | 0.880415 | 0.237098 | 0.385045 | 0.749114 |
| LNC_001693 | XLOC_037029 | -- | intronic_lncRNA | 0.177033 | 0.2966 | 0.124824 | 0.301216 |
| LNC_001694 | XLOC_037031 | -- | intronic_lncRNA | 0.448035 | 0.215788 | 0.521236 | 0.44858 |
| LNC_001695 | XLOC_037033 | -- | intronic_lncRNA | 0.251705 | 0.568621 | 0 | 0 |
| LNC_001696 | XLOC_037038 | -- | intronic_lncRNA | 0.422313 | 0.121675 | 0.204589 | 0.542898 |
| LNC_001697 | XLOC_037039 | -- | intronic_lncRNA | 0.554641 | 0.430955 | 0 | 0.277519 |
| LNC_001698 | XLOC_037245 | -- | intronic_lncRNA | 0 | 0.130924 | 0.10603 | 0.954105 |
| LNC_001699 | XLOC_037246 | -- | intronic_lncRNA | 0.285848 | 0 | 0.105791 | 0.580341 |
| LNC_002259 | XLOC_052074 | -- | intronic_lncRNA | 0.359713 | 0.074653 | 0.197046 | 0.43592 |
| LNC_002258 | XLOC_052073 | -- | intronic_lncRNA | 0 | 0.974778 | 0 | 0.303879 |
| LNC_002251 | XLOC_051667 | -- | lincRNA | 0.803526 | 0.102312 | 0 | 0 |
| LNC_002250 | XLOC_051603 | -- | intronic_lncRNA | 0.139066 | 0.043631 | 0.112371 | 0.52381 |
| LNC_002253 | XLOC_051670 | -- | intronic_lncRNA | 0.427901 | 0.638108 | 0.484926 | 0.776425 |
| LNC_002252 | XLOC_051670 | -- | intronic_lncRNA | 0.120439 | 0.044399 | 0.28562 | 0.601763 |
| LNC_002255 | XLOC_051750 | -- | lincRNA | 0.121244 | 0 | 0.474457 | 0 |
| LNC_002254 | XLOC_051679 | -- | intronic_lncRNA | 0.146978 | 0.481806 | 0.53345 | 0.412833 |
| LNC_002257 | XLOC_052011 | -- | intronic_lncRNA | 1.129633 | 1.355723 | 2.305172 | 3.085043 |
| LNC_002256 | XLOC_051964 | -- | intronic_lncRNA | 0.455668 | 0.178611 | 0.184223 | 0.434121 |

| LNC_003029 | XLOC_069258 | -- | lincRNA | 0.385196 | 0 | 0.113184 | 0.474913 |
| --- | --- | --- | --- | --- | --- | --- | --- |
| LNC_003028 | XLOC_069170 | -- | lincRNA | 0.249936 | 0.540472 | 0.571787 | 0.515182 |
| LNC_003021 | XLOC_069074 | -- | intronic_lncRNA | 0.846338 | 0.503406 | 0.695229 | 0.346007 |
| LNC_003020 | XLOC_069066 | -- | intronic_lncRNA | 0.467531 | 0.599689 | 0.300969 | 0.629533 |
| LNC_003023 | XLOC_069083 | -- | intronic_lncRNA | 0.33025 | 0.317515 | 0 | 1.268349 |
| LNC_003022 | XLOC_069082 | -- | intronic_lncRNA | 0 | 0 | 0 | 0.632197 |
| LNC_003025 | XLOC_069117 | -- | intronic_lncRNA | 0.162661 | 0.19453 | 0.251437 | 0.492715 |
| LNC_003024 | XLOC_069098 | -- | intronic_lncRNA | 0.600856 | 0.262474 | 0.92755 | 0.537985 |
| LNC_003027 | XLOC_069144 | -- | intronic_lncRNA | 0.075091 | 0.579993 | 0.12873 | 0.448591 |
| LNC_003026 | XLOC_069144 | -- | intronic_lncRNA | 0.039995 | 0 | 0.509685 | 0.195466 |
| LNC_002648 | XLOC_061242 | -- | intronic_lncRNA | 0.802928 | 0.158705 | 0.288897 | 0.476933 |
| LNC_002649 | XLOC_061252 | -- | intronic_lncRNA | 0.125085 | 0.138094 | 0 | 0.39136 |
| LNC_002646 | XLOC_061225 | -- | lincRNA | 0.727574 | 1.155491 | 0.144609 | 0.373017 |
| LNC_002647 | XLOC_061237 | -- | intronic_lncRNA | 0.97806 | 0.798104 | 0.976806 | 0.960045 |
| LNC_002644 | XLOC_061119 | -- | intronic_lncRNA | 0.142758 | 0.093097 | 0.412664 | 0.135186 |
| LNC_002645 | XLOC_061198 | -- | lincRNA | 0.572142 | 0 | 0.441974 | 0.638502 |
| LNC_002642 | XLOC_061085 | -- | intronic_lncRNA | 0.074851 | 0 | 0.575037 | 0.401861 |
| LNC_002643 | XLOC_061103 | -- | intronic_lncRNA | 0.416805 | 0.416983 | 0.517923 | 0.651403 |
| LNC_002640 | XLOC_061043 | -- | intronic_lncRNA | 0.661261 | 1.004456 | 1.376656 | 0.608185 |
| LNC_002641 | XLOC_061054 | -- | intronic_lncRNA | 0.627719 | 0.433138 | 0.5137 | 0.367415 |
| LNC_005065 | XLOC_115469 | -- | lincRNA | 1.523739 | 0.14625 | 0.361623 | 0.378498 |
| LNC_004316 | XLOC_093787 | -- | intronic_lncRNA | 0 | 0 | 0 | 0.75227 |
| LNC_004317 | XLOC_093788 | -- | intronic_lncRNA | 1.48084 | 1.742402 | 1.899648 | 0.983206 |
| LNC_004314 | XLOC_093765 | -- | intronic_lncRNA | 0.432292 | 0 | 0.549112 | 0.503074 |
| LNC_004315 | XLOC_093776 | -- | intronic_lncRNA | 0.525013 | 0.294546 | 0.323029 | 0.418167 |
| LNC_004312 | XLOC_093755 | -- | intronic_lncRNA | 0.346599 | 0.76542 | 0.717505 | 0.539903 |
| LNC_004313 | XLOC_093764 | -- | lincRNA | 0.279909 | 0 | 0.396521 | 0.056582 |
| LNC_004310 | XLOC_093753 | -- | intronic_lncRNA | 0.34949 | 0.71098 | 0.505422 | 0.898852 |
| LNC_004311 | XLOC_093754 | -- | intronic_lncRNA | 0.622135 | 0.473859 | 0.958639 | 0.671222 |
| LNC_004318 | XLOC_093801 | -- | lincRNA | 0.626172 | 0.190916 | 0.062685 | 0 |
| LNC_004319 | XLOC_093816 | -- | intronic_lncRNA | 1.718367 | 1.342419 | 0 | 1.343506 |
| LNC_000267 | XLOC_003905 | -- | lincRNA | 0.356069 | 0.654506 | 1.285431 | 0.764565 |
| LNC_005702 | XLOC_133480 | -- | intronic_lncRNA | 0 | 0.524881 | 0 | 0 |
| LNC_005703 | XLOC_133487 | -- | antisense_lncRNA | 1.433531 | 1.098481 | 0.775215 | 0.63083 |
| LNC_005700 | XLOC_133409 | -- | intronic_lncRNA | 0.096518 | 0.663576 | 0.476482 | 0.66796 |
| LNC_005701 | XLOC_133420 | -- | lincRNA | 0.242019 | 0.449173 | 0.314314 | 0.386494 |
| LNC_001849 | XLOC_041230 | -- | intronic_lncRNA | 1.124673 | 0.285902 | 0.544314 | 1.140008 |
| LNC_001848 | XLOC_041196 | -- | intronic_lncRNA | 1.206606 | 1.766757 | 1.582479 | 0.568068 |
| LNC_001845 | XLOC_041185 | -- | intronic_lncRNA | 0.096329 | 0.551923 | 0 | 1.139274 |
| LNC_001844 | XLOC_041185 | -- | intronic_lncRNA | 0.77618 | 0.442744 | 0.585353 | 0.345016 |
| LNC_001847 | XLOC_041195 | -- | intronic_lncRNA | 0.324904 | 0.462433 | 0.239978 | 0.6074 |
| LNC_001846 | XLOC_041194 | -- | intronic_lncRNA | 0.155082 | 0.09925 | 0.835045 | 0.313387 |
| LNC_001841 | XLOC_041183 | -- | intronic_lncRNA | 1.399539 | 1.020235 | 0.419783 | 0.782702 |
| LNC_001840 | XLOC_041183 | -- | intronic_lncRNA | 0.055274 | 0.002787 | 0.003563 | 8.20E-05 |
| LNC_001843 | XLOC_041183 | -- | intronic_lncRNA | 0.03273 | 0.25758 | 0.483899 | 0.194796 |
| LNC_001842 | XLOC_041183 | -- | intronic_lncRNA | 1.206155 | 1.097461 | 0.381721 | 0.252104 |
| LNC_005704 | XLOC_133520 | -- | intronic_lncRNA | 0 | 0.130043 | 0.375981 | 0.725156 |
| LNC_005705 | XLOC_133574 | -- | lincRNA | 2.386477 | 1.475131 | 1.790542 | 2.284712 |
| LNC_000439 | XLOC_007861 | -- | intronic_lncRNA | 0.597169 | 0.144731 | 0.165616 | 0.162474 |
| LNC_005158 | XLOC_117857 | -- | lincRNA | 1.328697 | 6.406915 | 4.380015 | 4.888943 |
| LNC_006239 | XLOC_149584 | -- | lincRNA | 0.823503 | 0.295563 | 0.156571 | 0.078272 |
| LNC_004767 | XLOC_106582 | -- | lincRNA | 2.601651 | 1.301361 | 0 | 3.354046 |
| LNC_004766 | XLOC_106582 | -- | lincRNA | 2.44491 | 4.892962 | 0.798436 | 2.448037 |
| LNC_004765 | XLOC_106582 | -- | lincRNA | 8.8867 | 3.591534 | 3.133244 | 3.580586 |
| LNC_004764 | XLOC_106582 | -- | lincRNA | 4.906684 | 2.724968 | 0 | 2.575126 |
| LNC_004763 | XLOC_106582 | -- | lincRNA | 15.977617 | 9.495317 | 7.756124 | 11.505884 |
| LNC_004762 | XLOC_106506 | -- | intronic_lncRNA | 0.599099 | 0.288955 | 0.125052 | 0.321113 |
| LNC_004761 | XLOC_106505 | -- | intronic_lncRNA | 0.143548 | 0.338168 | 0.352285 | 0.233381 |
| LNC_000436 | XLOC_007835 | -- | intronic_lncRNA | 0.351474 | 0.302605 | 0.245067 | 0.285282 |
| LNC_000435 | XLOC_007829 | -- | intronic_lncRNA | 0.641472 | 0.531001 | 0.954698 | 0.427209 |
| LNC_004769 | XLOC_106582 | -- | lincRNA | 2.41012 | 0.228611 | 2.873155 | 2.503126 |
| LNC_004768 | XLOC_106582 | -- | lincRNA | 0.163108 | 0 | 0 | 1.170721 |
| LNC_005154 | XLOC_117817 | -- | intronic_lncRNA | 0.743892 | 0.328973 | 1.181482 | 0.252418 |
| LNC_000433 | XLOC_007824 | -- | antisense_lncRNA | 0.355149 | 0.865261 | 1.212361 | 0.797608 |
| LNC_000432 | XLOC_007823 | -- | intronic_lncRNA | 0.48644 | 0.364012 | 0.096956 | 0 |
| LNC_006233 | XLOC_149428 | -- | intronic_lncRNA | 0.147839 | 0.243369 | 0 | 0.381926 |
| LNC_005151 | XLOC_117758 | -- | intronic_lncRNA | 0.180476 | 0.324486 | 0.276122 | 0.717376 |
| LNC_006232 | XLOC_149428 | -- | intronic_lncRNA | 0.11995 | 0 | 0.4843 | 0 |
| LNC_000430 | XLOC_007702 | -- | intronic_lncRNA | 1.302604 | 0.303353 | 0.706758 | 0.757471 |
| LNC_006231 | XLOC_149426 | -- | intronic_lncRNA | 0.357503 | 0.662987 | 0.103844 | 0.796316 |
| LNC_006230 | XLOC_149426 | -- | intronic_lncRNA | 0.430185 | 0.127731 | 0.987759 | 0.153649 |
| LNC_006237 | XLOC_149566 | -- | antisense_lncRNA | 0 | 0.627546 | 0.097025 | 0.083987 |
| LNC_006236 | XLOC_149476 | -- | intronic_lncRNA | 0.613505 | 0.685076 | 0.158223 | 0.105739 |
| LNC_006235 | XLOC_149441 | -- | intronic_lncRNA | 1.06171 | 0.572012 | 0.691282 | 0.397846 |
| LNC_006234 | XLOC_149430 | -- | intronic_lncRNA | 0.490279 | 0.594342 | 0.573991 | 0.475441 |
| LNC_005548 | XLOC_128562 | -- | intronic_lncRNA | 0.892114 | 0.995831 | 0.724283 | 0.949937 |
| LNC_005549 | XLOC_128577 | -- | intronic_lncRNA | 0.513976 | 0.438061 | 0.380784 | 0.878637 |

| LNC_005544 | XLOC_128350 | -- | intronic_lncRNA | 0.465299 | 0 | 0 | 0.509335 |
| --- | --- | --- | --- | --- | --- | --- | --- |
| LNC_005545 | XLOC_128352 | -- | intronic_lncRNA | 0 | 0.213625 | 0.261444 | 0.326807 |
| LNC_005546 | XLOC_128352 | -- | intronic_lncRNA | 0.121844 | 0.506544 | 0 | 0.058729 |
| LNC_005547 | XLOC_128520 | -- | intronic_lncRNA | 0.550506 | 0.281787 | 1.145756 | 0.707235 |
| LNC_005540 | XLOC_128053 | -- | lincRNA | 1.011134 | 0 | 0.076405 | 0 |
| LNC_005541 | XLOC_128185 | -- | intronic_lncRNA | 0 | 0.607933 | 0.239156 | 0.442124 |
| LNC_005542 | XLOC_128189 | -- | lincRNA | 1.029784 | 0.604916 | 0.962849 | 0.943395 |
| LNC_005543 | XLOC_128320 | -- | intronic_lncRNA | 0.744059 | 0.560196 | 0.372597 | 0.517889 |
| LNC_002004 | XLOC_045272 | -- | intronic_lncRNA | 0 | 0.343019 | 0.279037 | 0 |
| LNC_002005 | XLOC_045278 | -- | intronic_lncRNA | 0.169408 | 0.142046 | 0.708132 | 0.249691 |
| LNC_002006 | XLOC_045278 | -- | intronic_lncRNA | 0.734127 | 0.118421 | 0.441744 | 0.708977 |
| LNC_002007 | XLOC_045279 | -- | intronic_lncRNA | 0.768161 | 0.779504 | 0.505031 | 0.640043 |
| LNC_002000 | XLOC_045233 | -- | intronic_lncRNA | 0 | 0.437731 | 0.664688 | 0.445264 |
| LNC_002001 | XLOC_045262 | -- | intronic_lncRNA | 0.140589 | 0.260145 | 0.697898 | 0.364599 |
| LNC_002002 | XLOC_045267 | -- | intronic_lncRNA | 0.08376 | 0 | 0.12837 | 0.642868 |
| LNC_002003 | XLOC_045270 | -- | intronic_lncRNA | 0 | 0.400548 | 0.389265 | 0 |
| LNC_002008 | XLOC_045324 | -- | intronic_lncRNA | 0.437815 | 0.389257 | 0.994155 | 0.947844 |
| LNC_002009 | XLOC_045327 | -- | intronic_lncRNA | 0.766801 | 0.486719 | 0.468445 | 0.70525 |
| LNC_003234 | XLOC_074982 | -- | lincRNA | 0.249127 | 0.460542 | 0.603469 | 0.301683 |
| LNC_003235 | XLOC_075052 | -- | lincRNA | 0.346024 | 0.138573 | 0.383304 | 0.589724 |
| LNC_003236 | XLOC_075082 | -- | lincRNA | 0.653873 | 0.397855 | 0.692483 | 0.3227 |
| LNC_003237 | XLOC_075117 | -- | lincRNA | 0.521753 | 0.310449 | 0.037838 | 0.642487 |
| LNC_003230 | XLOC_074940 | -- | intronic_lncRNA | 1.27109 | 1.059929 | 0.875332 | 0.464597 |
| LNC_003231 | XLOC_074961 | -- | intronic_lncRNA | 0.526145 | 0.450441 | 0.205373 | 0.62832 |
| LNC_003232 | XLOC_074962 | -- | intronic_lncRNA | 1.841003 | 1.180937 | 1.68978 | 1.655 |
| LNC_003233 | XLOC_074967 | -- | intronic_lncRNA | 0.408884 | 0.079733 | 0.521951 | 0.283665 |
| LNC_003238 | XLOC_075117 | -- | lincRNA | 1.686568 | 1.215411 | 0.649308 | 0.989267 |
| LNC_003239 | XLOC_075117 | -- | lincRNA | 3.048303 | 1.010192 | 0.87456 | 0.945854 |
| LNC_005968 | XLOC_141286 | -- | lincRNA | 0 | 0.229677 | 0.563655 | 0.196071 |
| LNC_005969 | XLOC_141307 | -- | lincRNA | 0.750599 | 0.055137 | 0.021331 | 0.063982 |
| LNC_005966 | XLOC_141277 | -- | intronic_lncRNA | 0.297381 | 0.306559 | 0.147032 | 0.457157 |
| LNC_005967 | XLOC_141277 | -- | intronic_lncRNA | 0.28434 | 0.318399 | 0.435142 | 0.767468 |
| LNC_005964 | XLOC_141217 | -- | intronic_lncRNA | 0.758286 | 0.269286 | 0.574995 | 0.153076 |
| LNC_005965 | XLOC_141274 | -- | intronic_lncRNA | 0.11469 | 0.82783 | 0.163027 | 0.146303 |
| LNC_005962 | XLOC_141138 | -- | intronic_lncRNA | 1.140584 | 0.977276 | 1.436586 | 2.144458 |
| LNC_005963 | XLOC_141165 | -- | intronic_lncRNA | 0.736047 | 0.282674 | 0.864825 | 0.555878 |
| LNC_005960 | XLOC_141137 | -- | intronic_lncRNA | 0.587971 | 0.526127 | 0.529396 | 0 |
| LNC_005961 | XLOC_141137 | -- | intronic_lncRNA | 0 | 0 | 0 | 0.823 |
| LNC_001069 | XLOC_023082 | -- | antisense_lncRNA | 0.368708 | 0.129866 | 0.763149 | 0.559966 |
| LNC_001068 | XLOC_023078 | -- | antisense_lncRNA | 0.529697 | 0.152153 | 0.433444 | 0.646569 |
| LNC_001067 | XLOC_023002 | -- | intronic_lncRNA | 1.127174 | 0.944021 | 0.930589 | 1.144505 |
| LNC_001066 | XLOC_022992 | -- | intronic_lncRNA | 0.663626 | 0 | 0 | 0 |
| LNC_001065 | XLOC_022968 | -- | intronic_lncRNA | 0.544163 | 0 | 0.196959 | 0.507302 |
| LNC_001064 | XLOC_022967 | -- | intronic_lncRNA | 0.8834 | 3.177226 | 1.651135 | 2.535641 |
| LNC_001063 | XLOC_022958 | -- | intronic_lncRNA | 0.348946 | 1.102238 | 0.489369 | 0.345726 |
| LNC_001062 | XLOC_022927 | -- | lincRNA | 0 | 0 | 0 | 0.571631 |
| LNC_001061 | XLOC_022927 | -- | lincRNA | 0.216902 | 0.649246 | 0.109335 | 0.010304 |
| LNC_001060 | XLOC_022915 | -- | intronic_lncRNA | 0.467194 | 0 | 0 | 0 |
| LNC_003832 | XLOC_082271 | -- | intronic_lncRNA | 0.114826 | 0.190139 | 0.191461 | 0.627283 |
| LNC_003833 | XLOC_082272 | -- | intronic_lncRNA | 0 | 0 | 0.48794 | 0 |
| LNC_003830 | XLOC_082269 | -- | intronic_lncRNA | 0.187113 | 0 | 0.875231 | 0.495909 |
| LNC_003831 | XLOC_082269 | -- | intronic_lncRNA | 0.642121 | 0.165372 | 0.700288 | 0.618803 |
| LNC_003836 | XLOC_082311 | -- | intronic_lncRNA | 2.60012 | 0.290493 | 0.842363 | 0.677777 |
| LNC_003837 | XLOC_082315 | -- | intronic_lncRNA | 0.776947 | 0.655027 | 1.00466 | 0.628206 |
| LNC_003834 | XLOC_082277 | -- | intronic_lncRNA | 0.113535 | 0.669107 | 0.578375 | 1.122815 |
| LNC_003835 | XLOC_082282 | -- | intronic_lncRNA | 0 | 0.717585 | 1.785244 | 0.635895 |
| LNC_003838 | XLOC_082342 | -- | intronic_lncRNA | 3.25166 | 2.365929 | 3.554456 | 2.920061 |
| LNC_003839 | XLOC_082356 | -- | intronic_lncRNA | 0.857657 | 1.724747 | 0 | 0.094875 |
| LNC_000338 | XLOC_005139 | -- | intronic_lncRNA | 1.711824 | 0.500806 | 2.839961 | 1.572289 |
| LNC_000339 | XLOC_005147 | -- | lincRNA | 0.588893 | 1.261492 | 1.997653 | 2.33819 |
| LNC_000334 | XLOC_005110 | -- | intronic_lncRNA | 0 | 0.411059 | 0 | 0.975439 |
| LNC_000335 | XLOC_005111 | -- | intronic_lncRNA | 0.522049 | 0.268748 | 0.255526 | 0.793929 |
| LNC_000336 | XLOC_005111 | -- | intronic_lncRNA | 0.241836 | 0.077585 | 0.358903 | 0.029674 |
| LNC_000337 | XLOC_005123 | -- | intronic_lncRNA | 0 | 0.51555 | 0.091855 | 0 |
| LNC_000330 | XLOC_005006 | -- | antisense_lncRNA | 0.592564 | 0.637188 | 0.465592 | 0.60797 |
| LNC_000331 | XLOC_005009 | -- | intronic_lncRNA | 0.219523 | 0.306643 | 0.141434 | 0.425648 |
| LNC_000332 | XLOC_005038 | -- | intronic_lncRNA | 0.423024 | 0.156465 | 1.176162 | 0.754278 |
| LNC_000333 | XLOC_005110 | -- | intronic_lncRNA | 0.599343 | 0.107336 | 0.282068 | 0.082227 |
| LNC_002783 | XLOC_064701 | -- | intronic_lncRNA | 0.258294 | 0.14702 | 0.545518 | 0.446815 |
| LNC_002782 | XLOC_064496 | -- | intronic_lncRNA | 1.028843 | 0.214432 | 0 | 0 |
| LNC_002781 | XLOC_064493 | -- | intronic_lncRNA | 14.963162 | 35.774223 | 28.108957 | 9.501905 |
| LNC_002780 | XLOC_064493 | -- | intronic_lncRNA | 16.431461 | 18.74003 | 8.550295 | 14.68049 |
| LNC_002787 | XLOC_064887 | -- | intronic_lncRNA | 0 | 1.033409 | 1.301635 | 0 |
| LNC_002786 | XLOC_064860 | -- | intronic_lncRNA | 0 | 0.932172 | 0.488941 | 0.16936 |
| LNC_002785 | XLOC_064761 | -- | intronic_lncRNA | 0.270186 | 0.527267 | 0 | 0 |
| LNC_002784 | XLOC_064748 | -- | intronic_lncRNA | 0 | 0 | 0.49208 | 0.025738 |
| LNC_002789 | XLOC_064888 | -- | intronic_lncRNA | 0.516472 | 1.520093 | 1.432512 | 1.215211 |

| LNC_002788 | XLOC_064887 | -- | intronic_lncRNA | 0.396596 | 0.529797 | 0 | 0.59566 |
| --- | --- | --- | --- | --- | --- | --- | --- |
| LNC_003784 | XLOC_080977 | -- | intronic_lncRNA | 9.80E-05 | 0 | 0 | 1.95358 |
| LNC_003785 | XLOC_080985 | -- | lincRNA | 0.831771 | 0.315503 | 0.115378 | 0.141557 |
| LNC_003786 | XLOC_081004 | -- | lincRNA | 0 | 0.487389 | 0 | 0 |
| LNC_005378 | XLOC_123722 | -- | intronic_lncRNA | 0 | 0.557123 | 0 | 0.395898 |
| LNC_003780 | XLOC_080920 | -- | lincRNA | 0.54079 | 0 | 0 | 0 |
| LNC_003781 | XLOC_080934 | -- | lincRNA | 0.169851 | 0 | 0.443948 | 1.005151 |
| LNC_003782 | XLOC_080957 | -- | lincRNA | 0 | 0.809293 | 0 | 0 |
| LNC_003783 | XLOC_080977 | -- | intronic_lncRNA | 1.25865 | 1.006883 | 1.935991 | 0 |
| LNC_000253 | XLOC_003559 | -- | intronic_lncRNA | 1.731843 | 1.265602 | 2.03128 | 1.053979 |
| LNC_000252 | XLOC_003556 | -- | intronic_lncRNA | 0.496172 | 0 | 0.281025 | 0.118545 |
| LNC_000251 | XLOC_003545 | -- | intronic_lncRNA | 0.999168 | 0.309605 | 0 | 0.288854 |
| LNC_005370 | XLOC_123487 | -- | antisense_lncRNA | 0 | 0 | 0 | 0.423092 |
| LNC_000257 | XLOC_003650 | -- | intronic_lncRNA | 0.168005 | 0.205455 | 0.915668 | 0.409422 |
| LNC_005376 | XLOC_123544 | -- | intronic_lncRNA | 0.860299 | 0.922115 | 0.738941 | 0.289147 |
| LNC_005375 | XLOC_123542 | -- | intronic_lncRNA | 0.817126 | 0.923797 | 0.987186 | 0.332665 |
| LNC_005374 | XLOC_123542 | -- | intronic_lncRNA | 0.013822 | 0 | 1.126978 | 1.477611 |
| LNC_002875 | XLOC_066212 | -- | intronic_lncRNA | 0.870754 | 2.432947 | 1.471651 | 1.503706 |
| LNC_002874 | XLOC_066187 | -- | intronic_lncRNA | 2.154481 | 0.574774 | 0.77152 | 0.457723 |
| LNC_002877 | XLOC_066252 | -- | intronic_lncRNA | 0.480572 | 0.732079 | 0.252538 | 0.387306 |
| LNC_002876 | XLOC_066252 | -- | intronic_lncRNA | 0.537973 | 0.924503 | 1.125451 | 2.021823 |
| LNC_002871 | XLOC_066159 | -- | intronic_lncRNA | 0.324635 | 1.383673 | 0.881 | 1.172226 |
| LNC_002870 | XLOC_066159 | -- | intronic_lncRNA | 1.187972 | 0 | 0 | 0 |
| LNC_002873 | XLOC_066183 | -- | intronic_lncRNA | 0 | 0 | 0.312788 | 0.478132 |
| LNC_002872 | XLOC_066165 | -- | intronic_lncRNA | 0.643167 | 1.276721 | 0.743526 | 0 |
| LNC_002879 | XLOC_066264 | -- | intronic_lncRNA | 0.574687 | 0.104128 | 0.213633 | 0.052195 |
| LNC_002878 | XLOC_066252 | -- | intronic_lncRNA | 0.734929 | 0.305618 | 0.323424 | 0.418025 |
| LNC_000929 | XLOC_020061 | -- | intronic_lncRNA | 0.02519 | 0.86999 | 0.469713 | 0.216572 |
| LNC_000928 | XLOC_020060 | -- | intronic_lncRNA | 0.391225 | 0.204694 | 0.255603 | 0.294685 |
| LNC_006002 | XLOC_142665 | -- | intronic_lncRNA | 0 | 0.123595 | 0.406181 | 0.536464 |
| LNC_006003 | XLOC_142666 | -- | intronic_lncRNA | 0.380017 | 0.708618 | 0.14379 | 0 |
| LNC_006004 | XLOC_142722 | -- | intronic_lncRNA | 0.467762 | 0.67968 | 1.505772 | 0.938041 |
| LNC_006005 | XLOC_142767 | -- | intronic_lncRNA | 0.302068 | 0 | 0 | 0 |
| LNC_006006 | XLOC_142767 | -- | intronic_lncRNA | 0.29852 | 0.315606 | 1.150232 | 1.045932 |
| LNC_006007 | XLOC_142786 | -- | intronic_lncRNA | 0.653699 | 1.056688 | 0.676569 | 0.401827 |
| LNC_000921 | XLOC_019945 | -- | intronic_lncRNA | 0.247947 | 0.646714 | 0.370346 | 0.188601 |
| LNC_000920 | XLOC_019944 | -- | intronic_lncRNA | 0.245791 | 0.688308 | 0.247932 | 0.694353 |
| LNC_000923 | XLOC_019963 | -- | intronic_lncRNA | 0.502567 | 0.03823 | 0.055743 | 0.396823 |
| LNC_000922 | XLOC_019963 | -- | intronic_lncRNA | 0.543308 | 0.615234 | 0.585772 | 0.382569 |
| LNC_000925 | XLOC_019991 | -- | intronic_lncRNA | 0.845554 | 0.424127 | 0.52009 | 1.030271 |
| LNC_000924 | XLOC_019990 | -- | intronic_lncRNA | 0.198802 | 0 | 0.452891 | 0.212175 |
| LNC_000927 | XLOC_020057 | -- | intronic_lncRNA | 0.784873 | 0.435199 | 0.582847 | 0.790811 |
| LNC_000926 | XLOC_020028 | -- | intronic_lncRNA | 0.17035 | 0.14423 | 0.54128 | 0.725361 |
| LNC_005455 | XLOC_125724 | -- | intronic_lncRNA | 1.01174 | 0.380075 | 0.345683 | 0.891527 |
| LNC_002273 | XLOC_052236 | -- | intronic_lncRNA | 1.506509 | 0.791311 | 0.787365 | 0.415274 |
| LNC_002272 | XLOC_052201 | -- | intronic_lncRNA | 0 | 0 | 0.400974 | 0.020525 |
| LNC_002271 | XLOC_052200 | -- | intronic_lncRNA | 0.932227 | 0.499194 | 1.244419 | 1.618486 |
| LNC_002270 | XLOC_052198 | -- | intronic_lncRNA | 0.498989 | 0 | 0.207838 | 0.207802 |
| LNC_002277 | XLOC_052379 | -- | intronic_lncRNA | 0.783271 | 0.602531 | 0.734831 | 0.890863 |
| LNC_002276 | XLOC_052373 | -- | intronic_lncRNA | 0.21961 | 0.424127 | 0.257326 | 0.600991 |
| LNC_002275 | XLOC_052313 | -- | intronic_lncRNA | 1.255138 | 0.82575 | 0.582821 | 0.357748 |
| LNC_002274 | XLOC_052276 | -- | intronic_lncRNA | 0.642615 | 0.202644 | 0.774614 | 0.152858 |
| LNC_002279 | XLOC_052443 | -- | intronic_lncRNA | 0.188066 | 0 | 0.592075 | 0.301906 |
| LNC_002278 | XLOC_052423 | -- | intronic_lncRNA | 2.400416 | 2.179735 | 2.029788 | 2.28649 |
| LNC_003003 | XLOC_068719 | -- | intronic_lncRNA | 0.670511 | 0.647821 | 0.364568 | 0.377107 |
| LNC_003002 | XLOC_068603 | -- | intronic_lncRNA | 1.564448 | 1.888927 | 1.962924 | 3.425388 |
| LNC_003001 | XLOC_068598 | -- | intronic_lncRNA | 0 | 0.054983 | 0 | 1.613396 |
| LNC_003000 | XLOC_068596 | -- | intronic_lncRNA | 0.694983 | 0.434296 | 0.673499 | 0.72361 |
| LNC_003007 | XLOC_068891 | -- | lincRNA | 0 | 0.951058 | 0 | 0 |
| LNC_003006 | XLOC_068866 | -- | lincRNA | 0.619846 | 0.124886 | 0.58639 | 0.208508 |
| LNC_003005 | XLOC_068827 | -- | intronic_lncRNA | 0 | 0 | 0.436942 | 0 |
| LNC_003004 | XLOC_068730 | -- | intronic_lncRNA | 2.279657 | 1.444014 | 1.556794 | 1.33734 |
| LNC_003009 | XLOC_068894 | -- | intronic_lncRNA | 0.368085 | 0.308601 | 0.998911 | 0.393324 |
| LNC_003008 | XLOC_068891 | -- | lincRNA | 0.823979 | 0.025591 | 0.154004 | 0.076989 |
| LNC_005453 | XLOC_125708 | -- | intronic_lncRNA | 0.04687 | 0.149834 | 0.515467 | 0.169257 |
| LNC_002660 | XLOC_061853 | -- | intronic_lncRNA | 0 | 0 | 0.399755 | 0 |
| LNC_002661 | XLOC_061898 | -- | intronic_lncRNA | 0 | 0 | 0 | 0.398459 |
| LNC_002662 | XLOC_061899 | -- | intronic_lncRNA | 0.213067 | 0.861611 | 0.060562 | 0.340427 |
| LNC_002663 | XLOC_061950 | -- | intronic_lncRNA | 0 | 0.444858 | 0.154753 | 0.061657 |
| LNC_002664 | XLOC_061951 | -- | intronic_lncRNA | 0.511499 | 0.176007 | 0.802636 | 0.496003 |
| LNC_002665 | XLOC_061966 | -- | intronic_lncRNA | 0.139066 | 0 | 0.674229 | 0 |
| LNC_002666 | XLOC_061989 | -- | intronic_lncRNA | 0.353664 | 0.034065 | 0.455978 | 0.94351 |
| LNC_002667 | XLOC_061989 | -- | intronic_lncRNA | 0.367921 | 0.558425 | 0.525618 | 0.291819 |
| LNC_002668 | XLOC_061989 | -- | intronic_lncRNA | 1.577515 | 0.600134 | 0.825909 | 0.517423 |
| LNC_002669 | XLOC_062006 | -- | intronic_lncRNA | 0.581296 | 0 | 0.611253 | 0.56168 |
| LNC_004338 | XLOC_094320 | -- | intronic_lncRNA | 0.64527 | 0 | 0 | 0 |
| LNC_004339 | XLOC_094324 | -- | intronic_lncRNA | 0.683802 | 0 | 0.318226 | 0.29317 |

| LNC_004848 | XLOC_108868 | -- | intronic_lncRNA | 6.313613 | 0.008572 | 7.527063 | 0.012538 |
| --- | --- | --- | --- | --- | --- | --- | --- |
| LNC_004849 | XLOC_108868 | -- | intronic_lncRNA | 0.012189 | 6.33936 | 0 | 7.627212 |
| LNC_004330 | XLOC_093963 | -- | intronic_lncRNA | 0.111147 | 0.345694 | 0.535724 | 0.715373 |
| LNC_004331 | XLOC_093974 | -- | intronic_lncRNA | 0.568405 | 0.310617 | 0.451647 | 0.045157 |
| LNC_004840 | XLOC_108785 | -- | lincRNA | 0 | 0 | 0.403716 | 1.172929 |
| LNC_004333 | XLOC_094011 | -- | lincRNA | 0 | 0.734169 | 0 | 0 |
| LNC_004334 | XLOC_094086 | -- | lincRNA | 0.58324 | 0 | 0 | 0 |
| LNC_004335 | XLOC_094110 | -- | intronic_lncRNA | 1.002344 | 0.718889 | 1.245252 | 1.284569 |
| LNC_004844 | XLOC_108818 | -- | intronic_lncRNA | 0.82348 | 0.517985 | 0.942149 | 0.806936 |
| LNC_004845 | XLOC_108851 | -- | intronic_lncRNA | 0.528974 | 0.454039 | 0.651878 | 0.831077 |
| LNC_001867 | XLOC_041590 | -- | lincRNA | 0 | 0.089147 | 0.339042 | 0.611218 |
| LNC_001866 | XLOC_041567 | -- | lincRNA | 0 | 0 | 1.83509 | 1.100501 |
| LNC_001865 | XLOC_041567 | -- | lincRNA | 0.216811 | 0 | 2.336162 | 2.063893 |
| LNC_001864 | XLOC_041567 | -- | lincRNA | 0 | 0.098928 | 0.515076 | 0.994384 |
| LNC_001863 | XLOC_041546 | -- | intronic_lncRNA | 1.560541 | 1.45518 | 1.694782 | 1.293591 |
| LNC_001862 | XLOC_041493 | -- | intronic_lncRNA | 1.650879 | 2.299972 | 2.498333 | 2.948528 |
| LNC_001861 | XLOC_041465 | -- | lincRNA | 0.200878 | 0.309653 | 0.211496 | 0.712515 |
| LNC_001860 | XLOC_041281 | -- | intronic_lncRNA | 0.480407 | 0.10697 | 0.242803 | 0.405279 |
| LNC_001319 | XLOC_028816 | -- | intronic_lncRNA | 0.486914 | 0.008239 | 0.036362 | 0.085442 |
| LNC_001318 | XLOC_028816 | -- | intronic_lncRNA | 0.989821 | 0.570722 | 0.766326 | 0.585208 |
| LNC_001869 | XLOC_041621 | -- | lincRNA | 0 | 0 | 0 | 0.496439 |
| LNC_001868 | XLOC_041618 | -- | intronic_lncRNA | 0.670073 | 0.768488 | 1.467199 | 1.096012 |
| LNC_003649 | XLOC_078088 | -- | intronic_lncRNA | 11.027009 | 4.084646 | 10.379421 | 9.509596 |
| LNC_003648 | XLOC_078078 | -- | intronic_lncRNA | 0.308485 | 0.126085 | 0.158613 | 0.484492 |
| LNC_003641 | XLOC_077911 | -- | intronic_lncRNA | 1.447842 | 0.627713 | 1.381015 | 1.211278 |
| LNC_003640 | XLOC_077897 | -- | lincRNA | 0.814189 | 0.127133 | 0.160282 | 0.102685 |
| LNC_003643 | XLOC_077925 | -- | intronic_lncRNA | 0.745784 | 0.354369 | 0.849362 | 0.535714 |
| LNC_003642 | XLOC_077920 | -- | intronic_lncRNA | 0.40341 | 0.498023 | 0.34098 | 0.569055 |
| LNC_003645 | XLOC_077926 | -- | antisense_lncRNA | 0.471127 | 0.144825 | 0.529176 | 0.504027 |
| LNC_003644 | XLOC_077925 | -- | intronic_lncRNA | 0.89048 | 0.397392 | 0.516303 | 0.67835 |
| LNC_003647 | XLOC_077996 | -- | intronic_lncRNA | 20.992792 | 14.492046 | 3.201297 | 5.568864 |
| LNC_003646 | XLOC_077937 | -- | intronic_lncRNA | 0.73261 | 0.261402 | 0.448299 | 0.432357 |
| LNC_005412 | XLOC_124818 | -- | intronic_lncRNA | 0.382556 | 0.447764 | 1.009806 | 1.072572 |
| LNC_006446 | XLOC_155005 | -- | intronic_lncRNA | 0.767088 | 0.39276 | 0.823396 | 0.777448 |
| LNC_000133 | XLOC_001327 | -- | intronic_lncRNA | 0.325006 | 0 | 0 | 0.558157 |
| LNC_006447 | XLOC_155023 | -- | intronic_lncRNA | 0.581759 | 0.341964 | 0.606259 | 0.541819 |
| LNC_005410 | XLOC_124788 | -- | intronic_lncRNA | 0.734067 | 0.286756 | 0.409164 | 0.345865 |
| LNC_006448 | XLOC_155024 | -- | intronic_lncRNA | 0.691938 | 0.704347 | 0.966255 | 1.068786 |
| LNC_005411 | XLOC_124789 | -- | intronic_lncRNA | 0.091247 | 0.184711 | 0.163053 | 0.582303 |
| LNC_006449 | XLOC_155080 | -- | intronic_lncRNA | 0 | 0 | 0 | 0.561922 |
| LNC_005416 | XLOC_124908 | -- | intronic_lncRNA | 0.149819 | 0 | 0.602206 | 0.261444 |
| LNC_000137 | XLOC_001359 | -- | intronic_lncRNA | 1.510307 | 1.520272 | 1.196208 | 2.433738 |
| LNC_005414 | XLOC_124887 | -- | intronic_lncRNA | 0.368135 | 0 | 0.600619 | 0 |
| LNC_005415 | XLOC_124894 | -- | intronic_lncRNA | 0.672043 | 0.608908 | 1.027629 | 1.019732 |
| LNC_005526 | XLOC_127661 | -- | intronic_lncRNA | 2.812191 | 0.837104 | 3.029404 | 2.357283 |
| LNC_005527 | XLOC_127661 | -- | intronic_lncRNA | 0.741903 | 0.21086 | 0.173796 | 0.612167 |
| LNC_005524 | XLOC_127661 | -- | intronic_lncRNA | 0.123997 | 0.064522 | 0.622434 | 0.66903 |
| LNC_005525 | XLOC_127661 | -- | intronic_lncRNA | 0.277135 | 0.073153 | 1.880343 | 1.031962 |
| LNC_005522 | XLOC_127576 | -- | intronic_lncRNA | 0.821468 | 2.20E-05 | 0.687974 | 1.099285 |
| LNC_005523 | XLOC_127576 | -- | intronic_lncRNA | 0 | 0.726449 | 0 | 0 |
| LNC_005520 | XLOC_127524 | -- | intronic_lncRNA | 0.797812 | 0.230497 | 0.513461 | 0.227895 |
| LNC_005521 | XLOC_127552 | -- | intronic_lncRNA | 0.372556 | 0.067302 | 0.505067 | 0.191238 |
| LNC_000138 | XLOC_001393 | -- | intronic_lncRNA | 0.857435 | 0.645442 | 1.518933 | 0.869905 |
| LNC_005528 | XLOC_127670 | -- | intronic_lncRNA | 1.051817 | 0.462944 | 0.39605 | 0.179831 |
| LNC_005529 | XLOC_127671 | -- | intronic_lncRNA | 0.759302 | 0.25813 | 1.035179 | 0.381225 |
| LNC_005931 | XLOC_140156 | -- | intronic_lncRNA | 0.423172 | 0.211112 | 0.28457 | 0.479687 |
| LNC_000139 | XLOC_001418 | -- | intronic_lncRNA | 0.02523 | 1.066464 | 1.254674 | 0.084657 |
| LNC_002068 | XLOC_046472 | -- | intronic_lncRNA | 0 | 0.616418 | 0.646706 | 0.720102 |
| LNC_002069 | XLOC_046507 | -- | intronic_lncRNA | 0.815051 | 1.204509 | 1.258808 | 0.942591 |
| LNC_002066 | XLOC_046342 | -- | intronic_lncRNA | 0.044659 | 0 | 0.323419 | 0.765743 |
| LNC_002067 | XLOC_046424 | -- | intronic_lncRNA | 0.816155 | 1.50764 | 1.116288 | 0.847043 |
| LNC_002064 | XLOC_046339 | -- | intronic_lncRNA | 0.179782 | 0.842286 | 0.716837 | 0.284684 |
| LNC_002065 | XLOC_046340 | -- | intronic_lncRNA | 0.731497 | 0.190412 | 0.748703 | 0.673717 |
| LNC_002062 | XLOC_046305 | -- | intronic_lncRNA | 0.239584 | 0.386342 | 0.395924 | 0.544963 |
| LNC_002063 | XLOC_046324 | -- | intronic_lncRNA | 0.927845 | 0.522817 | 0.673794 | 0.382568 |
| LNC_002060 | XLOC_046241 | -- | intronic_lncRNA | 0 | 0.470583 | 0.476382 | 0.4763 |
| LNC_002061 | XLOC_046265 | -- | intronic_lncRNA | 0.871944 | 0.078621 | 0 | 0.057921 |
| LNC_005932 | XLOC_140158 | -- | intronic_lncRNA | 0.074213 | 0 | 0 | 0.611898 |
| LNC_003258 | XLOC_075135 | -- | lincRNA | 4.59447 | 1.228409 | 0.738486 | 1.166358 |
| LNC_003259 | XLOC_075135 | -- | lincRNA | 0.897245 | 0 | 0 | 0 |
| LNC_003256 | XLOC_075133 | -- | lincRNA | 3.016522 | 1.457552 | 0.793553 | 1.2142 |
| LNC_003257 | XLOC_075134 | -- | lincRNA | 2.015566 | 1.129112 | 0.172428 | 0.567437 |
| LNC_003254 | XLOC_075128 | -- | lincRNA | 1.298676 | 0.641642 | 0.262467 | 0.511911 |
| LNC_003255 | XLOC_075131 | -- | lincRNA | 5.217077 | 1.667536 | 0.901019 | 1.047086 |
| LNC_003252 | XLOC_075127 | -- | lincRNA | 4.190183 | 1.812393 | 1.133302 | 1.480078 |
| LNC_003253 | XLOC_075127 | -- | lincRNA | 1.281664 | 0.854173 | 0.410766 | 0.160434 |
| LNC_003250 | XLOC_075119 | -- | lincRNA | 1.399382 | 0.457541 | 0.444934 | 0.378644 |

| LNC_003251 | XLOC_075120 | -- | lincRNA | 1.00014 | 0.454715 | 0.193594 | 0.167422 |
| --- | --- | --- | --- | --- | --- | --- | --- |
| LNC_005937 | XLOC_140284 | -- | intronic_lncRNA | 1.05595 | 0.199506 | 1.08409 | 0.28033 |
| LNC_005936 | XLOC_140272 | -- | intronic_lncRNA | 0.640511 | 0.692068 | 0.401289 | 0.874554 |
| LNC_005940 | XLOC_140310 | -- | intronic_lncRNA | 0.445881 | 0.175776 | 0.571816 | 0.221524 |
| LNC_005941 | XLOC_140401 | -- | intronic_lncRNA | 0.040793 | 0.448785 | 0.254022 | 0.515598 |
| LNC_005942 | XLOC_140412 | -- | intronic_lncRNA | 0.938148 | 0.096666 | 0.823041 | 0.334743 |
| LNC_005943 | XLOC_140413 | -- | intronic_lncRNA | 0.58838 | 0 | 0.808108 | 0.757134 |
| LNC_005944 | XLOC_140420 | -- | intronic_lncRNA | 0.110246 | 0 | 0 | 0.504719 |
| LNC_005945 | XLOC_140468 | -- | lincRNA | 0.541249 | 0.121084 | 0 | 0 |
| LNC_005946 | XLOC_140478 | -- | intronic_lncRNA | 0.96376 | 1.577947 | 0.520018 | 0.818005 |
| LNC_005947 | XLOC_140479 | -- | intronic_lncRNA | 0 | 0.108383 | 0.589513 | 0.355782 |
| LNC_005948 | XLOC_140480 | -- | intronic_lncRNA | 0 | 0.073697 | 0.53636 | 0.317222 |
| LNC_005949 | XLOC_140481 | -- | intronic_lncRNA | 0 | 0 | 0.701064 | 0.174457 |
| LNC_006253 | XLOC_150150 | -- | intronic_lncRNA | 0.676825 | 0.673342 | 1.151304 | 0.556954 |
| LNC_001081 | XLOC_023344 | -- | intronic_lncRNA | 0.436292 | 0.286385 | 0.105384 | 0.706684 |
| LNC_001080 | XLOC_023300 | -- | intronic_lncRNA | 0 | 0.534227 | 0 | 0.31101 |
| LNC_001083 | XLOC_023433 | -- | intronic_lncRNA | 0.738278 | 0.34986 | 0.780778 | 0.650205 |
| LNC_001082 | XLOC_023432 | -- | intronic_lncRNA | 0 | 0.192564 | 0.557318 | 0.513737 |
| LNC_001085 | XLOC_023436 | -- | intronic_lncRNA | 0.353445 | 0.194901 | 0.595538 | 0.270615 |
| LNC_001084 | XLOC_023433 | -- | intronic_lncRNA | 0 | 0.391493 | 0.704625 | 0.695923 |
| LNC_001087 | XLOC_023517 | -- | intronic_lncRNA | 0.620061 | 0.935103 | 1.096723 | 1.161445 |
| LNC_001086 | XLOC_023456 | -- | intronic_lncRNA | 0 | 0 | 0 | 0.504875 |
| LNC_001089 | XLOC_023524 | -- | intronic_lncRNA | 0.800529 | 0 | 0 | 0.005538 |
| LNC_001088 | XLOC_023524 | -- | intronic_lncRNA | 0 | 0.897041 | 0.975246 | 0.903291 |
| LNC_005379 | XLOC_123722 | -- | intronic_lncRNA | 0.433802 | 0.36444 | 0.409663 | 0.794685 |
| LNC_000688 | XLOC_013734 | -- | intronic_lncRNA | 2.351753 | 3.197736 | 3.712909 | 3.833308 |
| LNC_000689 | XLOC_013764 | -- | intronic_lncRNA | 0.389196 | 0.766873 | 0.412451 | 1.11244 |
| LNC_000684 | XLOC_013666 | -- | intronic_lncRNA | 0.464322 | 0.566147 | 0.052307 | 0.669841 |
| LNC_000685 | XLOC_013666 | -- | intronic_lncRNA | 0.258091 | 0.131634 | 0.582624 | 0.153144 |
| LNC_000686 | XLOC_013666 | -- | antisense_lncRNA | 0.031988 | 0.038519 | 0.510033 | 0.178228 |
| LNC_000687 | XLOC_013667 | -- | intronic_lncRNA | 0.284717 | 0 | 0.376338 | 0.902346 |
| LNC_000680 | XLOC_013639 | -- | intronic_lncRNA | 4.700338 | 1.385734 | 5.857921 | 3.857714 |
| LNC_000681 | XLOC_013657 | -- | intronic_lncRNA | 0.296907 | 2.427379 | 1.41905 | 0.576624 |
| LNC_000682 | XLOC_013660 | -- | intronic_lncRNA | 0.063991 | 1.077961 | 0 | 0.269879 |
| LNC_000683 | XLOC_013660 | -- | intronic_lncRNA | 0.538235 | 0.330724 | 0.813407 | 1.052546 |
| LNC_003814 | XLOC_082019 | -- | intronic_lncRNA | 0.232519 | 0.899078 | 0.225337 | 0.151566 |
| LNC_003815 | XLOC_082019 | -- | intronic_lncRNA | 0 | 0.005667 | 0.485689 | 0.034699 |
| LNC_003816 | XLOC_082044 | -- | intronic_lncRNA | 0.952453 | 0.815803 | 1.666268 | 2.45796 |
| LNC_003817 | XLOC_082057 | -- | intronic_lncRNA | 0.43913 | 0.613099 | 0.326175 | 0.482644 |
| LNC_003810 | XLOC_081855 | -- | lincRNA | 0.646422 | 0.825446 | 0.441927 | 0.619676 |
| LNC_003811 | XLOC_081866 | -- | lincRNA | 0.110921 | 0.669223 | 0.636722 | 0.780712 |
| LNC_003812 | XLOC_081872 | -- | lincRNA | 0.982594 | 1.347823 | 1.690855 | 0.705688 |
| LNC_003813 | XLOC_081934 | -- | lincRNA | 0.621443 | 0.083452 | 0 | 0.399581 |
| LNC_003818 | XLOC_082084 | -- | intronic_lncRNA | 0 | 0 | 1.038625 | 1.134534 |
| LNC_003819 | XLOC_082088 | -- | intronic_lncRNA | 0.248374 | 0.707419 | 0.669814 | 1.388675 |
| LNC_000356 | XLOC_005839 | -- | intronic_lncRNA | 0.16043 | 0.391354 | 0.685804 | 1.157417 |
| LNC_000357 | XLOC_005839 | -- | intronic_lncRNA | 0.342093 | 0.042938 | 1.149558 | 0.173537 |
| LNC_000354 | XLOC_005835 | -- | intronic_lncRNA | 0.376137 | 0.369254 | 0.135051 | 0.506734 |
| LNC_000355 | XLOC_005836 | -- | intronic_lncRNA | 0.280151 | 0.367708 | 0.577256 | 0.225705 |
| LNC_000352 | XLOC_005823 | -- | intronic_lncRNA | 0 | 0 | 0.333721 | 0.72127 |
| LNC_000353 | XLOC_005834 | -- | intronic_lncRNA | 0.598854 | 0.331286 | 0.392947 | 0.330059 |
| LNC_000350 | XLOC_005576 | -- | lincRNA | 1.445607 | 1.837588 | 1.184295 | 0.871032 |
| LNC_000351 | XLOC_005728 | -- | intronic_lncRNA | 0.672753 | 0.5631 | 1.021849 | 0.492936 |
| LNC_000358 | XLOC_005839 | -- | intronic_lncRNA | 0.172266 | 0.515908 | 0.261837 | 0.76747 |
| LNC_000359 | XLOC_005862 | -- | lincRNA | 0.465539 | 0.706855 | 0.045648 | 0 |
| LNC_005319 | XLOC_121701 | -- | intronic_lncRNA | 0.146343 | 0 | 0 | 1.168787 |
| LNC_005318 | XLOC_121677 | -- | intronic_lncRNA | 0.327923 | 0.572631 | 0.055439 | 0.064094 |
| LNC_005315 | XLOC_121611 | -- | intronic_lncRNA | 0.588649 | 0.071966 | 0.180945 | 0.471728 |
| LNC_005314 | XLOC_121610 | -- | intronic_lncRNA | 0.608703 | 0.143177 | 0.172334 | 0.53217 |
| LNC_005317 | XLOC_121663 | -- | intronic_lncRNA | 0.301111 | 0.301295 | 0.610017 | 0 |
| LNC_005316 | XLOC_121625 | -- | intronic_lncRNA | 0.40438 | 0.100532 | 0.728395 | 0.530616 |
| LNC_005311 | XLOC_121483 | -- | lincRNA | 0 | 0.557686 | 0.142437 | 0.180859 |
| LNC_005310 | XLOC_121471 | -- | intronic_lncRNA | 0.665653 | 0.586136 | 1.153844 | 0.70402 |
| LNC_005313 | XLOC_121610 | -- | intronic_lncRNA | 0 | 0.43515 | 0.633051 | 0.051279 |
| LNC_005312 | XLOC_121587 | -- | intronic_lncRNA | 0.314988 | 0.114428 | 0.56805 | 0.588645 |
| LNC_002859 | XLOC_065811 | -- | intronic_lncRNA | 0.572404 | 1.083456 | 0.583393 | 0.259501 |
| LNC_002858 | XLOC_065800 | -- | intronic_lncRNA | 0.926378 | 0 | 0 | 2.041358 |
| LNC_002857 | XLOC_065787 | -- | antisense_lncRNA | 0 | 0 | 0 | 0.506014 |
| LNC_002856 | XLOC_065784 | -- | antisense_lncRNA | 0 | 0.148418 | 0.092095 | 0.413772 |
| LNC_002855 | XLOC_065747 | -- | intronic_lncRNA | 0 | 0 | 0.311058 | 0.413408 |
| LNC_002854 | XLOC_065708 | -- | intronic_lncRNA | 0.877327 | 0.417156 | 1.303408 | 1.17233 |
| LNC_002853 | XLOC_065682 | -- | intronic_lncRNA | 0.567808 | 0.627032 | 0.120157 | 0.100009 |
| LNC_002852 | XLOC_065659 | -- | intronic_lncRNA | 0 | 0.159047 | 0.437082 | 0.348003 |
| LNC_002851 | XLOC_065640 | -- | intronic_lncRNA | 0.664385 | 0.097976 | 0.923238 | 0.76879 |
| LNC_002850 | XLOC_065629 | -- | intronic_lncRNA | 0.232054 | 1.341933 | 0.925987 | 0.749911 |
| LNC_005371 | XLOC_123532 | -- | intronic_lncRNA | 0.066358 | 0.068217 | 0.388996 | 0.126642 |
| LNC_004042 | XLOC_087296 | -- | intronic_lncRNA | 0.771199 | 0 | 0.975057 | 0.662581 |

| LNC_004043 | XLOC_087424 | -- | lincRNA | 0.413954 | 0.489046 | 0 | 0.407656 |
| --- | --- | --- | --- | --- | --- | --- | --- |
| LNC_004040 | XLOC_087233 | -- | intronic_lncRNA | 0.286071 | 0 | 0.156879 | 0.460387 |
| LNC_004041 | XLOC_087273 | -- | intronic_lncRNA | 0.351384 | 0.155317 | 0.233709 | 0.422231 |
| LNC_004046 | XLOC_087451 | -- | intronic_lncRNA | 0.664701 | 0.048079 | 0.142865 | 0 |
| LNC_004047 | XLOC_087454 | -- | intronic_lncRNA | 0 | 0.221371 | 0.259534 | 0.780728 |
| LNC_004044 | XLOC_087431 | -- | intronic_lncRNA | 0.431637 | 0.576243 | 0.598735 | 0.700633 |
| LNC_004045 | XLOC_087436 | -- | antisense_lncRNA | 0 | 0 | 0 | 0.551211 |
| LNC_004048 | XLOC_087508 | -- | intronic_lncRNA | 0 | 0.72713 | 0.277117 | 0.864917 |
| LNC_004049 | XLOC_087513 | -- | intronic_lncRNA | 0.078134 | 0.379009 | 0.907933 | 1.269379 |
| LNC_000903 | XLOC_019531 | -- | intronic_lncRNA | 9.004103 | 4.813513 | 6.83637 | 6.376358 |
| LNC_000902 | XLOC_019522 | -- | intronic_lncRNA | 0.467494 | 0.54903 | 0.544665 | 0.358922 |
| LNC_000901 | XLOC_019515 | -- | intronic_lncRNA | 0.320804 | 0.256068 | 0.103689 | 0.461338 |
| LNC_000900 | XLOC_019514 | -- | intronic_lncRNA | 0 | 0.269895 | 0.395408 | 0 |
| LNC_000907 | XLOC_019746 | -- | intronic_lncRNA | 0.153548 | 0.469112 | 0.886468 | 0.182511 |
| LNC_000906 | XLOC_019679 | -- | intronic_lncRNA | 1.251843 | 0 | 0.773795 | 1.200243 |
| LNC_000905 | XLOC_019584 | -- | intronic_lncRNA | 0.336629 | 2.728725 | 0.083227 | 2.502865 |
| LNC_000904 | XLOC_019561 | -- | lincRNA | 0.580045 | 0.124681 | 0.188432 | 0.23404 |
| LNC_000909 | XLOC_019753 | -- | intronic_lncRNA | 1.288793 | 0.115572 | 0.135587 | 0.509876 |
| LNC_000908 | XLOC_019752 | -- | intronic_lncRNA | 0.573009 | 0.435809 | 1.048274 | 1.419173 |
| LNC_004594 | XLOC_101579 | -- | intronic_lncRNA | 0.91768 | 0.311546 | 0.588727 | 0.803638 |
| LNC_004595 | XLOC_101579 | -- | intronic_lncRNA | 0.799518 | 0.381711 | 1.320528 | 0.620162 |
| LNC_004596 | XLOC_101650 | -- | lincRNA | 0.628192 | 0.249891 | 0.449725 | 0.901376 |
| LNC_004597 | XLOC_101677 | -- | intronic_lncRNA | 0.187052 | 0.600392 | 0.512878 | 0.640945 |
| LNC_004590 | XLOC_101496 | -- | intronic_lncRNA | 0.651536 | 0.466818 | 0.464617 | 0.350136 |
| LNC_004591 | XLOC_101501 | -- | intronic_lncRNA | 0.879793 | 0.153459 | 0.125399 | 0.271171 |
| LNC_004592 | XLOC_101506 | -- | intronic_lncRNA | 0.391818 | 0.491027 | 0.623512 | 0 |
| LNC_004593 | XLOC_101513 | -- | intronic_lncRNA | 0 | 0.154791 | 0.481481 | 0.34276 |
| LNC_004598 | XLOC_101677 | -- | intronic_lncRNA | 0.90797 | 0 | 0.258765 | 0.296508 |
| LNC_004599 | XLOC_101677 | -- | intronic_lncRNA | 0.345253 | 0 | 0.449104 | 0.28021 |
| LNC_005885 | XLOC_138700 | -- | intronic_lncRNA | 0.199111 | 1.186653 | 0.32394 | 0 |
| LNC_005884 | XLOC_138620 | -- | lincRNA | 1.290658 | 0.184711 | 1.460248 | 0.641003 |
| LNC_005887 | XLOC_138732 | -- | intronic_lncRNA | 0.506432 | 0 | 0.306025 | 0.436318 |
| LNC_005886 | XLOC_138701 | -- | intronic_lncRNA | 0 | 0 | 0.529751 | 0 |
| LNC_005881 | XLOC_138513 | -- | intronic_lncRNA | 0.630042 | 0.663081 | 0.189286 | 0.191331 |
| LNC_005880 | XLOC_138512 | -- | intronic_lncRNA | 0.922641 | 0.450106 | 0.263519 | 0.494623 |
| LNC_005883 | XLOC_138585 | -- | lincRNA | 0.422201 | 0.116503 | 0.255263 | 0.818976 |
| LNC_005882 | XLOC_138515 | -- | intronic_lncRNA | 0.750097 | 0.123725 | 0.153836 | 0.113982 |
| LNC_005889 | XLOC_138779 | -- | intronic_lncRNA | 0.486523 | 0.301817 | 0.094152 | 0.157331 |
| LNC_005888 | XLOC_138734 | -- | intronic_lncRNA | 0 | 0 | 0.606712 | 0 |
| LNC_002215 | XLOC_050772 | -- | intronic_lncRNA | 0.420546 | 1.589312 | 1.578032 | 0.989512 |
| LNC_002214 | XLOC_050736 | -- | lincRNA | 0.273556 | 0.027141 | 0.340844 | 0.574238 |
| LNC_002217 | XLOC_050796 | -- | intronic_lncRNA | 2.261077 | 1.536084 | 2.976273 | 3.713997 |
| LNC_002216 | XLOC_050776 | -- | intronic_lncRNA | 0.939747 | 0.43272 | 1.679747 | 2.255965 |
| LNC_002211 | XLOC_050548 | -- | intronic_lncRNA | 0 | 0.262243 | 0.340511 | 1.278824 |
| LNC_002210 | XLOC_050499 | -- | intronic_lncRNA | 0.177265 | 0.145351 | 0 | 0.570566 |
| LNC_002213 | XLOC_050654 | -- | intronic_lncRNA | 1.095143 | 0.197818 | 0.065403 | 0 |
| LNC_002212 | XLOC_050562 | -- | lincRNA | 0 | 0.318976 | 0.48424 | 0.086457 |
| LNC_002219 | XLOC_050823 | -- | intronic_lncRNA | 0.908933 | 0.086759 | 0.222839 | 0.136338 |
| LNC_002218 | XLOC_050823 | -- | intronic_lncRNA | 1.030027 | 0.474404 | 0.291151 | 1.328957 |
| LNC_003065 | XLOC_070429 | -- | intronic_lncRNA | 0.106364 | 0.429625 | 0.927055 | 0.105136 |
| LNC_003064 | XLOC_070428 | -- | intronic_lncRNA | 0.428324 | 0.595983 | 0.529163 | 0.330743 |
| LNC_003067 | XLOC_070445 | -- | intronic_lncRNA | 0.97738 | 0.691546 | 0.681371 | 0.306277 |
| LNC_003066 | XLOC_070445 | -- | intronic_lncRNA | 1.870775 | 1.477918 | 0.582859 | 0.843075 |
| LNC_003061 | XLOC_070359 | -- | intronic_lncRNA | 0.88832 | 0.368311 | 0.663829 | 0.680138 |
| LNC_003060 | XLOC_070283 | -- | intronic_lncRNA | 0 | 0 | 0.664635 | 0.250691 |
| LNC_003063 | XLOC_070426 | -- | intronic_lncRNA | 0 | 0.320661 | 0.42241 | 0.423756 |
| LNC_003062 | XLOC_070416 | -- | lincRNA | 0 | 0 | 0 | 0.477997 |
| LNC_003069 | XLOC_070447 | -- | intronic_lncRNA | 2.075953 | 2.133652 | 1.728256 | 1.28372 |
| LNC_003068 | XLOC_070446 | -- | intronic_lncRNA | 1.283951 | 0.964112 | 0.052917 | 0.274364 |
| LNC_005089 | XLOC_116372 | -- | intronic_lncRNA | 1.089548 | 0.381368 | 0.173764 | 0.083446 |
| LNC_005088 | XLOC_116371 | -- | intronic_lncRNA | 1.096748 | 0.505577 | 0.583671 | 0.828977 |
| LNC_005085 | XLOC_116272 | -- | lincRNA | 0.666394 | 0.719713 | 0.534143 | 0.476034 |
| LNC_005084 | XLOC_116213 | -- | intronic_lncRNA | 0.133816 | 0.587292 | 0.172286 | 0.086488 |
| LNC_005087 | XLOC_116370 | -- | intronic_lncRNA | 0.840825 | 0.356634 | 0.391762 | 0.769752 |
| LNC_005086 | XLOC_116324 | -- | lincRNA | 0.562587 | 0.678943 | 0.328427 | 0.221868 |
| LNC_005081 | XLOC_116116 | -- | intronic_lncRNA | 0.624443 | 0.567693 | 0.153251 | 0.229836 |
| LNC_005080 | XLOC_115947 | -- | intronic_lncRNA | 0 | 0 | 0.624836 | 0.215759 |
| LNC_005083 | XLOC_116210 | -- | intronic_lncRNA | 0.440664 | 0.303105 | 0.386607 | 0.504388 |
| LNC_005082 | XLOC_116183 | -- | intronic_lncRNA | 0.841982 | 0.628327 | 0.783138 | 0.953947 |
| LNC_001748 | XLOC_038676 | -- | intronic_lncRNA | 0.227831 | 0.361592 | 0.204987 | 0.055509 |
| LNC_001749 | XLOC_038702 | -- | intronic_lncRNA | 0.43186 | 0.631305 | 1.261822 | 1.247397 |
| LNC_001742 | XLOC_038479 | -- | lincRNA | 2.161297 | 1.730911 | 1.316993 | 1.313079 |
| LNC_001743 | XLOC_038490 | -- | intronic_lncRNA | 0 | 0.761839 | 0 | 0 |
| LNC_001740 | XLOC_038419 | -- | intronic_lncRNA | 0 | 0 | 0.548349 | 0 |
| LNC_001741 | XLOC_038420 | -- | intronic_lncRNA | 0.398005 | 0.362626 | 0.546164 | 0.694999 |
| LNC_001746 | XLOC_038655 | -- | intronic_lncRNA | 0.252742 | 0.180548 | 0.403742 | 0.420759 |
| LNC_001747 | XLOC_038658 | -- | lincRNA | 0 | 0 | 0.407513 | 0 |

| LNC_001744 | XLOC_038524 | -- | lincRNA | 0.637321 | 0 | 0 | 0 |
| --- | --- | --- | --- | --- | --- | --- | --- |
| LNC_001745 | XLOC_038654 | -- | intronic_lncRNA | 0.443299 | 0.380061 | 0.419559 | 0.40987 |
| LNC_005029 | XLOC_114758 | -- | intronic_lncRNA | 1.24959 | 0.675763 | 1.404633 | 1.160388 |
| LNC_005028 | XLOC_114742 | -- | intronic_lncRNA | 0.541347 | 0.730482 | 0.621233 | 0.985151 |
| LNC_006309 | XLOC_151354 | -- | intronic_lncRNA | 0.53582 | 0.598453 | 0.782739 | 0.944826 |
| LNC_000507 | XLOC_008948 | -- | intronic_lncRNA | 0.541471 | 0.258976 | 0.463876 | 0.206273 |
| LNC_006308 | XLOC_151347 | -- | intronic_lncRNA | 0.270588 | 0.252042 | 0.679635 | 0.406406 |
| LNC_004868 | XLOC_110127 | -- | intronic_lncRNA | 0.314153 | 0.023604 | 0.182059 | 0.379867 |
| LNC_004869 | XLOC_110127 | -- | intronic_lncRNA | 0.444152 | 0.723661 | 0 | 0.249853 |
| LNC_005026 | XLOC_114723 | -- | intronic_lncRNA | 1.373566 | 0.457671 | 1.198965 | 0.919675 |
| LNC_004864 | XLOC_109715 | -- | intronic_lncRNA | 0.418986 | 0.489859 | 0 | 0.467516 |
| LNC_004865 | XLOC_109801 | -- | lincRNA | 0.269014 | 0.448774 | 0.461153 | 0.350622 |
| LNC_004866 | XLOC_109919 | -- | lincRNA | 0 | 0 | 0.604882 | 0.533873 |
| LNC_004867 | XLOC_109987 | -- | lincRNA | 0.073585 | 0.038107 | 0.13254 | 0.43065 |
| LNC_004860 | XLOC_109514 | -- | intronic_lncRNA | 0.737789 | 0.73598 | 0.891383 | 0.65569 |
| LNC_000505 | XLOC_008929 | -- | intronic_lncRNA | 0.233863 | 0.413646 | 0.511461 | 0.440549 |
| LNC_004862 | XLOC_109603 | -- | intronic_lncRNA | 0.512306 | 0.622883 | 0.619785 | 0.609882 |
| LNC_004863 | XLOC_109651 | -- | intronic_lncRNA | 1.209608 | 0.506716 | 1.211299 | 1.051226 |
| LNC_005024 | XLOC_114698 | -- | intronic_lncRNA | 0.570279 | 0.132907 | 1.019927 | 0.446861 |
| LNC_000503 | XLOC_008810 | -- | intronic_lncRNA | 0.685192 | 0.626901 | 1.324346 | 0.814826 |
| LNC_005022 | XLOC_114678 | -- | antisense_lncRNA | 0.850883 | 0.113095 | 0 | 0 |
| LNC_006303 | XLOC_151127 | -- | intronic_lncRNA | 0.863948 | 0.460005 | 1.056481 | 0.871179 |
| LNC_005021 | XLOC_114644 | -- | intronic_lncRNA | 0.301325 | 0 | 0.590815 | 0.526818 |
| LNC_006302 | XLOC_151023 | -- | lincRNA | 0.27354 | 0.328608 | 0.619187 | 0.184926 |
| LNC_005020 | XLOC_114642 | -- | intronic_lncRNA | 0.728888 | 0.228707 | 0.188551 | 0 |
| LNC_006301 | XLOC_151010 | -- | intronic_lncRNA | 0.159282 | 0.234291 | 0.461309 | 0.172906 |
| LNC_006300 | XLOC_151009 | -- | intronic_lncRNA | 0 | 0.404744 | 0 | 0.396314 |
| LNC_006307 | XLOC_151241 | -- | intronic_lncRNA | 1.050139 | 2.344571 | 0.617896 | 0.312389 |
| LNC_001466 | XLOC_031784 | -- | intronic_lncRNA | 0.071052 | 0.132722 | 0.537429 | 0.805825 |
| LNC_001339 | XLOC_029187 | -- | intronic_lncRNA | 2.0284 | 0.001363 | 0 | 0.012648 |
| LNC_001338 | XLOC_029181 | -- | intronic_lncRNA | 1.38035 | 0.801947 | 1.478678 | 1.078109 |
| LNC_006305 | XLOC_151177 | -- | intronic_lncRNA | 0.579599 | 0.204903 | 0.342856 | 0.921895 |
| LNC_001333 | XLOC_029164 | -- | intronic_lncRNA | 0.27375 | 0.667774 | 0.341669 | 1.030621 |
| LNC_001332 | XLOC_029156 | -- | intronic_lncRNA | 1.374772 | 0.688659 | 2.620329 | 2.984052 |
| LNC_001331 | XLOC_029152 | -- | intronic_lncRNA | 4.391785 | 1.274593 | 0.97336 | 0.791973 |
| LNC_001330 | XLOC_029101 | -- | intronic_lncRNA | 2.66769 | 3.550207 | 0.787177 | 0.674372 |
| LNC_001337 | XLOC_029171 | -- | intronic_lncRNA | 0.749533 | 0.807564 | 0.545293 | 0.252736 |
| LNC_001336 | XLOC_029169 | -- | intronic_lncRNA | 0.123381 | 0 | 0.100366 | 0.501743 |
| LNC_001335 | XLOC_029168 | -- | intronic_lncRNA | 0.74385 | 0.094848 | 0.592742 | 0.277248 |
| LNC_001334 | XLOC_029164 | -- | intronic_lncRNA | 1.092262 | 0 | 0.793942 | 0 |
| LNC_006166 | XLOC_147728 | -- | intronic_lncRNA | 0.66358 | 0.914482 | 0.706499 | 1.030402 |
| LNC_006422 | XLOC_153987 | -- | intronic_lncRNA | 0.210681 | 0.198057 | 0.260574 | 0.316014 |
| LNC_003663 | XLOC_078352 | -- | intronic_lncRNA | 1.909205 | 0.408375 | 0.62556 | 0.641559 |
| LNC_003662 | XLOC_078207 | -- | intronic_lncRNA | 0.358681 | 0.224918 | 0.488253 | 0.356705 |
| LNC_003661 | XLOC_078181 | -- | intronic_lncRNA | 0.449781 | 0 | 0.734679 | 0.181336 |
| LNC_003660 | XLOC_078153 | -- | intronic_lncRNA | 0.231918 | 0.034633 | 0.811257 | 0.532092 |
| LNC_003667 | XLOC_078417 | -- | intronic_lncRNA | 0.437888 | 0 | 0.613162 | 0 |
| LNC_003666 | XLOC_078360 | -- | intronic_lncRNA | 1.368341 | 0.364907 | 0.848983 | 1.511005 |
| LNC_003665 | XLOC_078355 | -- | intronic_lncRNA | 1.465094 | 0.585323 | 0.192224 | 0.142457 |
| LNC_003664 | XLOC_078353 | -- | intronic_lncRNA | 0.780555 | 0.445983 | 0.881836 | 0.781686 |
| LNC_003669 | XLOC_078451 | -- | intronic_lncRNA | 0.028275 | 1.574917 | 1.159782 | 0.151491 |
| LNC_003668 | XLOC_078451 | -- | intronic_lncRNA | 3.661598 | 1.282294 | 1.797432 | 2.995819 |
| LNC_006423 | XLOC_153987 | -- | intronic_lncRNA | 0.201588 | 0.166978 | 0.209069 | 0.229646 |
| LNC_006420 | XLOC_153987 | -- | intronic_lncRNA | 0.47864 | 0.213714 | 0.794326 | 0.492662 |
| LNC_005508 | XLOC_127300 | -- | intronic_lncRNA | 1.060632 | 1.372045 | 0.997714 | 0.701766 |
| LNC_005509 | XLOC_127311 | -- | intronic_lncRNA | 0 | 0 | 0.537839 | 0 |
| LNC_005500 | XLOC_127095 | -- | intronic_lncRNA | 0 | 0 | 0.154712 | 0.384335 |
| LNC_005501 | XLOC_127111 | -- | intronic_lncRNA | 1.014109 | 0 | 0.026754 | 0 |
| LNC_005502 | XLOC_127131 | -- | lincRNA | 0.555375 | 0.739255 | 0.387458 | 0.17385 |
| LNC_005503 | XLOC_127209 | -- | intronic_lncRNA | 0 | 0 | 0.416093 | 0.107222 |
| LNC_005504 | XLOC_127222 | -- | intronic_lncRNA | 0.798855 | 0.4909 | 0.336235 | 0.77819 |
| LNC_005505 | XLOC_127225 | -- | intronic_lncRNA | 0.758137 | 1.357976 | 0.711643 | 0.810775 |
| LNC_005506 | XLOC_127254 | -- | lincRNA | 0.577693 | 0.485125 | 0.799676 | 0.789512 |
| LNC_005507 | XLOC_127300 | -- | intronic_lncRNA | 1.26981 | 1.038671 | 0.570471 | 0.605597 |
| LNC_002040 | XLOC_045736 | -- | intronic_lncRNA | 0.624621 | 0.398159 | 0.496647 | 0.427327 |
| LNC_002041 | XLOC_045737 | -- | intronic_lncRNA | 1.398605 | 1.435721 | 1.660165 | 1.230452 |
| LNC_002042 | XLOC_045763 | -- | intronic_lncRNA | 0.641083 | 0 | 0.141438 | 0 |
| LNC_002043 | XLOC_045805 | -- | intronic_lncRNA | 0.556264 | 0 | 0 | 0 |
| LNC_002044 | XLOC_045811 | -- | intronic_lncRNA | 0 | 0 | 0 | 0.408035 |
| LNC_002045 | XLOC_045811 | -- | intronic_lncRNA | 0.369401 | 0.191682 | 0.575676 | 0 |
| LNC_002046 | XLOC_045912 | -- | intronic_lncRNA | 0.176717 | 0 | 0.374964 | 0.403117 |
| LNC_002047 | XLOC_046001 | -- | intronic_lncRNA | 1.025264 | 0.05807 | 0.059483 | 0.341585 |
| LNC_002048 | XLOC_046002 | -- | intronic_lncRNA | 0.018289 | 0.421185 | 0.479299 | 0.347432 |
| LNC_002049 | XLOC_046020 | -- | intronic_lncRNA | 0.433306 | 0.461491 | 0.597578 | 0.65284 |
| LNC_003270 | XLOC_075144 | -- | lincRNA | 1.719296 | 1.152133 | 0.148479 | 0.630687 |
| LNC_003271 | XLOC_075145 | -- | lincRNA | 4.863759 | 1.214318 | 0.823356 | 0.766509 |
| LNC_003272 | XLOC_075145 | -- | lincRNA | 3.568063 | 1.110052 | 0.245937 | 1.009484 |

| LNC_003273 | XLOC_075145 | -- | lincRNA | 1.71392 | 1.127748 | 0.303752 | 0.363718 |
| --- | --- | --- | --- | --- | --- | --- | --- |
| LNC_003274 | XLOC_075145 | -- | lincRNA | 3.428272 | 1.212101 | 1.150494 | 1.045539 |
| LNC_003275 | XLOC_075145 | -- | lincRNA | 0.002371 | 0.72737 | 0 | 0.588793 |
| LNC_003276 | XLOC_075145 | -- | lincRNA | 2.045778 | 0.92245 | 1.756631 | 0 |
| LNC_003277 | XLOC_075145 | -- | lincRNA | 0.456449 | 0.491836 | 0.014283 | 0 |
| LNC_003278 | XLOC_075145 | -- | lincRNA | 2.64171 | 1.573646 | 0.593255 | 1.964911 |
| LNC_003279 | XLOC_075145 | -- | lincRNA | 10.169019 | 0.767761 | 1.223256 | 0.805013 |
| LNC_006160 | XLOC_147657 | -- | intronic_lncRNA | 0.130141 | 0 | 0.701064 | 0 |
| LNC_006427 | XLOC_154035 | -- | lincRNA | 1.560752 | 0.211975 | 1.31328 | 1.631689 |
| LNC_003970 | XLOC_085626 | -- | intronic_lncRNA | 0 | 0.153033 | 0.135089 | 1.226329 |
| LNC_006424 | XLOC_153987 | -- | intronic_lncRNA | 1.935518 | 0.613086 | 1.358653 | 1.366809 |
| LNC_004986 | XLOC_113643 | -- | intronic_lncRNA | 0.710904 | 0.176566 | 0.325675 | 0.265337 |
| LNC_006425 | XLOC_153987 | -- | antisense_lncRNA | 0.045169 | 0 | 0.347858 | 0.805045 |
| LNC_004679 | XLOC_103955 | -- | intronic_lncRNA | 1.476351 | 0.185774 | 0.073589 | 0.183302 |
| LNC_004678 | XLOC_103875 | -- | intronic_lncRNA | 0.693318 | 0.795719 | 0.908797 | 1.200476 |
| LNC_004671 | XLOC_103767 | -- | intronic_lncRNA | 0 | 0 | 0.16782 | 0.409142 |
| LNC_004670 | XLOC_103757 | -- | intronic_lncRNA | 0.5618 | 0.050815 | 0.567388 | 0.652589 |
| LNC_004673 | XLOC_103807 | -- | intronic_lncRNA | 1.045974 | 0.364286 | 0.910975 | 1.24909 |
| LNC_004672 | XLOC_103775 | -- | intronic_lncRNA | 0 | 0 | 0.428961 | 0 |
| LNC_004675 | XLOC_103808 | -- | intronic_lncRNA | 0.136994 | 0.512587 | 0.02727 | 0.220293 |
| LNC_004674 | XLOC_103808 | -- | intronic_lncRNA | 0 | 0.374404 | 0.460964 | 0.156516 |
| LNC_004677 | XLOC_103846 | -- | intronic_lncRNA | 0.283375 | 0.478901 | 0.279524 | 0.734828 |
| LNC_004676 | XLOC_103845 | -- | intronic_lncRNA | 0 | 0 | 0.69755 | 0.566797 |
| LNC_001531 | XLOC_033361 | -- | intronic_lncRNA | 0.299456 | 0 | 0.533764 | 0 |
| LNC_001530 | XLOC_033360 | -- | intronic_lncRNA | 0.306733 | 0.347792 | 0.58932 | 0.568636 |
| LNC_001533 | XLOC_033510 | -- | lincRNA | 0.579179 | 0.734764 | 0.43549 | 0.447391 |
| LNC_001532 | XLOC_033384 | -- | intronic_lncRNA | 2.93947 | 0.103941 | 0 | 0 |
| LNC_001535 | XLOC_033676 | -- | intronic_lncRNA | 0.546438 | 1.115795 | 1.041207 | 1.235075 |
| LNC_001534 | XLOC_033597 | -- | lincRNA | 1.590534 | 0.6623 | 0.578596 | 0.590893 |
| LNC_001537 | XLOC_033680 | -- | intronic_lncRNA | 0.570724 | 0.716925 | 0.439708 | 0.55772 |
| LNC_001536 | XLOC_033680 | -- | intronic_lncRNA | 0.698411 | 0.063274 | 0.843592 | 1.35361 |
| LNC_001539 | XLOC_033724 | -- | intronic_lncRNA | 0.693328 | 0.742732 | 0.798435 | 0.900017 |
| LNC_001538 | XLOC_033688 | -- | intronic_lncRNA | 0 | 0 | 0.540563 | 0.063057 |
| LNC_004332 | XLOC_094004 | -- | intronic_lncRNA | 0.395221 | 0.14572 | 0.620708 | 0.634671 |
| LNC_006167 | XLOC_147740 | -- | intronic_lncRNA | 0.331421 | 0.708617 | 0.532634 | 0.586765 |
| LNC_001794 | XLOC_040200 | -- | intronic_lncRNA | 0.695719 | 0.435515 | 1.167731 | 0.960012 |
| LNC_006428 | XLOC_154037 | -- | lincRNA | 1.227116 | 2.163585 | 0.686298 | 0.983591 |
| LNC_006165 | XLOC_147724 | -- | intronic_lncRNA | 1.165306 | 1.465132 | 1.7507 | 1.872813 |
| LNC_006164 | XLOC_147724 | -- | intronic_lncRNA | 0.1196 | 0.043298 | 0.142986 | 0.471097 |
| LNC_002625 | XLOC_060788 | -- | intronic_lncRNA | 0 | 0 | 0 | 0.972051 |
| LNC_006163 | XLOC_147675 | -- | intronic_lncRNA | 0.703992 | 0 | 0.326144 | 0.215586 |
| LNC_006162 | XLOC_147674 | -- | intronic_lncRNA | 0 | 0 | 0 | 0.546083 |
| LNC_006161 | XLOC_147663 | -- | intronic_lncRNA | 0.640392 | 0.165975 | 1.125841 | 0.640407 |
| LNC_001792 | XLOC_039924 | -- | intronic_lncRNA | 0.317784 | 0.215317 | 0.517832 | 0.502711 |
| LNC_002623 | XLOC_060786 | -- | intronic_lncRNA | 0.287108 | 1.036023 | 0.663216 | 0.465748 |
| LNC_003878 | XLOC_083397 | -- | intronic_lncRNA | 0.396864 | 0.91328 | 0.315233 | 0.166848 |
| LNC_003879 | XLOC_083398 | -- | intronic_lncRNA | 0 | 0.444471 | 0.354642 | 0.538731 |
| LNC_003876 | XLOC_083395 | -- | lincRNA | 0.494861 | 0.35177 | 0 | 0.185863 |
| LNC_003877 | XLOC_083397 | -- | intronic_lncRNA | 0 | 0 | 0 | 0.972653 |
| LNC_003874 | XLOC_083349 | -- | intronic_lncRNA | 0.081843 | 0.541955 | 0.508714 | 0.295003 |
| LNC_003875 | XLOC_083367 | -- | intronic_lncRNA | 0.082664 | 0 | 0.298128 | 0.598003 |
| LNC_003872 | XLOC_083279 | -- | intronic_lncRNA | 0.304452 | 0.732982 | 0.376411 | 0.405269 |
| LNC_003873 | XLOC_083342 | -- | intronic_lncRNA | 0.673867 | 0 | 0.557523 | 0.293578 |
| LNC_003870 | XLOC_083275 | -- | intronic_lncRNA | 0.562213 | 0.411412 | 0.408982 | 0.568302 |
| LNC_003871 | XLOC_083276 | -- | intronic_lncRNA | 0.528352 | 0.081689 | 0.131872 | 0.330727 |
| LNC_000378 | XLOC_006449 | -- | intronic_lncRNA | 0.601419 | 0.357279 | 0.541633 | 0.766711 |
| LNC_000379 | XLOC_006450 | -- | intronic_lncRNA | 0.646136 | 0.376708 | 0.182768 | 0.639576 |
| LNC_000370 | XLOC_006175 | -- | intronic_lncRNA | 1.734681 | 1.618129 | 1.656269 | 1.882984 |
| LNC_000371 | XLOC_006208 | -- | lincRNA | 0.764656 | 0.19355 | 0.072263 | 0 |
| LNC_000372 | XLOC_006290 | -- | intronic_lncRNA | 0.2167 | 0.542516 | 0.544569 | 0.16533 |
| LNC_000373 | XLOC_006311 | -- | intronic_lncRNA | 0.376024 | 0.401975 | 1.26676 | 0.911382 |
| LNC_000374 | XLOC_006346 | -- | intronic_lncRNA | 0.796873 | 0.622663 | 1.047171 | 1.138408 |
| LNC_000375 | XLOC_006373 | -- | intronic_lncRNA | 0 | 0.415093 | 0.43371 | 0.12557 |
| LNC_000376 | XLOC_006381 | -- | intronic_lncRNA | 0.733879 | 0.307414 | 1.016541 | 0.566249 |
| LNC_000377 | XLOC_006449 | -- | intronic_lncRNA | 0.42751 | 0.093298 | 0 | 0 |
| LNC_000217 | XLOC_003094 | -- | intronic_lncRNA | 0.767794 | 0.411436 | 0.410409 | 0.438949 |
| LNC_000216 | XLOC_003065 | -- | intronic_lncRNA | 0.446995 | 0.121084 | 0 | 0.392177 |
| LNC_000215 | XLOC_003064 | -- | intronic_lncRNA | 0 | 0 | 0 | 0.522832 |
| LNC_000214 | XLOC_003061 | -- | intronic_lncRNA | 0.160268 | 0.494255 | 0 | 0 |
| LNC_000213 | XLOC_003034 | -- | lincRNA | 0 | 0 | 0 | 0.643331 |
| LNC_000212 | XLOC_002955 | -- | intronic_lncRNA | 0.638606 | 0.242256 | 0.883211 | 0.686404 |
| LNC_000211 | XLOC_002955 | -- | intronic_lncRNA | 0.096415 | 0.140562 | 0.494382 | 0.567188 |
| LNC_000210 | XLOC_002949 | -- | lincRNA | 0.836624 | 0.017216 | 0 | 0 |
| LNC_000219 | XLOC_003101 | -- | intronic_lncRNA | 0.195204 | 0.169711 | 0.591608 | 0.021047 |
| LNC_000218 | XLOC_003094 | -- | intronic_lncRNA | 0 | 0 | 1.03716 | 0 |
| LNC_001298 | XLOC_028469 | -- | intronic_lncRNA | 0.240786 | 0.093332 | 0.441937 | 0.620223 |
| LNC_001299 | XLOC_028491 | -- | intronic_lncRNA | 0.166827 | 0.557138 | 0 | 0.136521 |

| LNC_001294 | XLOC_028461 | -- | intronic_lncRNA | 0.887752 | 0.870499 | 0 | 0.493525 |
| --- | --- | --- | --- | --- | --- | --- | --- |
| LNC_001295 | XLOC_028465 | -- | intronic_lncRNA | 0.805168 | 0.369329 | 0.384618 | 0.333225 |
| LNC_001296 | XLOC_028466 | -- | intronic_lncRNA | 0.722169 | 0.364926 | 0.669066 | 0.537073 |
| LNC_001297 | XLOC_028468 | -- | intronic_lncRNA | 0 | 0.098304 | 0.594969 | 0 |
| LNC_001290 | XLOC_028271 | -- | intronic_lncRNA | 0.6906 | 0.39127 | 0.483797 | 0.332125 |
| LNC_001291 | XLOC_028355 | -- | intronic_lncRNA | 0.986704 | 0.603009 | 0.531223 | 0.279543 |
| LNC_001292 | XLOC_028356 | -- | intronic_lncRNA | 0.144907 | 0.462911 | 0.952586 | 0.183534 |
| LNC_001293 | XLOC_028458 | -- | intronic_lncRNA | 0.867789 | 0.557337 | 0.451478 | 0.748095 |
| LNC_004064 | XLOC_087775 | -- | intronic_lncRNA | 0 | 0 | 0.3828 | 0.544389 |
| LNC_004065 | XLOC_087779 | -- | intronic_lncRNA | 0.387531 | 0.130927 | 1.030391 | 0.49609 |
| LNC_004066 | XLOC_087810 | -- | intronic_lncRNA | 0.370252 | 0.221653 | 0.149391 | 0.478606 |
| LNC_004067 | XLOC_087829 | -- | intronic_lncRNA | 0 | 0.557686 | 0 | 0 |
| LNC_004060 | XLOC_087745 | -- | intronic_lncRNA | 0.198395 | 0.410444 | 0.372306 | 0.249223 |
| LNC_004061 | XLOC_087766 | -- | intronic_lncRNA | 0.350179 | 0.628908 | 0.396143 | 0.346283 |
| LNC_004062 | XLOC_087768 | -- | lincRNA | 0.538237 | 0 | 0 | 0.101464 |
| LNC_004063 | XLOC_087775 | -- | intronic_lncRNA | 0.358754 | 0 | 0 | 0.665958 |
| LNC_004068 | XLOC_087840 | -- | intronic_lncRNA | 0 | 0.199998 | 0.24637 | 0.485826 |
| LNC_004069 | XLOC_087872 | -- | lincRNA | 0.14691 | 0.660832 | 0 | 0 |
| LNC_000965 | XLOC_020620 | -- | intronic_lncRNA | 0 | 0 | 0.185105 | 0.438908 |
| LNC_000964 | XLOC_020598 | -- | intronic_lncRNA | 0.389365 | 0.600585 | 0.744407 | 0.208416 |
| LNC_000967 | XLOC_020678 | -- | intronic_lncRNA | 0 | 0.335 | 0.641684 | 0 |
| LNC_000966 | XLOC_020659 | -- | intronic_lncRNA | 0.647192 | 0.590951 | 0.626465 | 0.605895 |
| LNC_000961 | XLOC_020504 | -- | lincRNA | 0.0203 | 0.618522 | 0.179805 | 0 |
| LNC_000960 | XLOC_020494 | -- | intronic_lncRNA | 0.909503 | 0.307314 | 0.560857 | 0.614064 |
| LNC_000963 | XLOC_020590 | -- | intronic_lncRNA | 0 | 0.385301 | 0.506099 | 0.56444 |
| LNC_000962 | XLOC_020514 | -- | intronic_lncRNA | 0.175548 | 0.175981 | 0 | 0.401395 |
| LNC_000969 | XLOC_020698 | -- | lincRNA | 1.005071 | 0 | 0.952043 | 0.000848 |
| LNC_000968 | XLOC_020698 | -- | lincRNA | 0.811587 | 1.02783 | 0.570175 | 0.978244 |
| LNC_004375 | XLOC_095315 | -- | intronic_lncRNA | 0.008787 | 0.833965 | 0 | 0 |
| LNC_002185 | XLOC_049775 | -- | intronic_lncRNA | 8.734239 | 10.243195 | 11.355677 | 14.150002 |
| LNC_002184 | XLOC_049707 | -- | intronic_lncRNA | 2.41123 | 0.982384 | 1.741122 | 0.676853 |
| LNC_002187 | XLOC_049777 | -- | antisense_lncRNA | 0 | 0 | 0 | 0.460208 |
| LNC_002186 | XLOC_049775 | -- | intronic_lncRNA | 3.60463 | 4.247245 | 7.996681 | 8.710773 |
| LNC_002181 | XLOC_049616 | -- | intronic_lncRNA | 0.166324 | 0.182531 | 0.614088 | 0.523825 |
| LNC_002180 | XLOC_049615 | -- | intronic_lncRNA | 0.69036 | 0.582237 | 0.54497 | 0.749412 |
| LNC_002183 | XLOC_049706 | -- | intronic_lncRNA | 0.972119 | 0.486325 | 1.315961 | 1.476569 |
| LNC_002182 | XLOC_049690 | -- | intronic_lncRNA | 0.971933 | 0.288291 | 0.773395 | 0.557169 |
| LNC_002189 | XLOC_050008 | -- | intronic_lncRNA | 0.765569 | 0.652419 | 0.947259 | 0.492618 |
| LNC_002188 | XLOC_049778 | -- | intronic_lncRNA | 0.278556 | 0.500509 | 1.152915 | 0.502745 |
| LNC_003595 | XLOC_076171 | -- | lincRNA | 0 | 0 | 0 | 0.788271 |
| LNC_003594 | XLOC_076117 | -- | intronic_lncRNA | 0.374694 | 0.507977 | 0.721009 | 0.775284 |
| LNC_003597 | XLOC_076259 | -- | lincRNA | 0.096412 | 0.140775 | 0.513036 | 0.226837 |
| LNC_003596 | XLOC_076247 | -- | intronic_lncRNA | 0.54949 | 0.179573 | 0.361366 | 0.264489 |
| LNC_003591 | XLOC_076079 | -- | intronic_lncRNA | 0.374085 | 0.115844 | 0.619796 | 0.462128 |
| LNC_003590 | XLOC_076042 | -- | intronic_lncRNA | 0.104428 | 0 | 0 | 0.438908 |
| LNC_003593 | XLOC_076109 | -- | lincRNA | 0.260076 | 0 | 0.624892 | 0.104363 |
| LNC_003592 | XLOC_076094 | -- | intronic_lncRNA | 0.632785 | 0.475573 | 0.668103 | 0.565385 |
| LNC_003599 | XLOC_076265 | -- | lincRNA | 0 | 0 | 0 | 0.596903 |
| LNC_003598 | XLOC_076260 | -- | lincRNA | 0.38416 | 0.096105 | 0.233494 | 0.543429 |
| LNC_002239 | XLOC_051372 | -- | intronic_lncRNA | 0.597881 | 0.437142 | 0.820004 | 0.389448 |
| LNC_002238 | XLOC_051354 | -- | intronic_lncRNA | 0 | 1.438413 | 0 | 0 |
| LNC_002237 | XLOC_051345 | -- | lincRNA | 0.000485 | 0.425991 | 0.698105 | 0.097768 |
| LNC_002236 | XLOC_051235 | -- | lincRNA | 0.174784 | 0 | 0.482995 | 0 |
| LNC_002235 | XLOC_051173 | -- | intronic_lncRNA | 0.98318 | 0.287796 | 0 | 0.158125 |
| LNC_002234 | XLOC_051161 | -- | intronic_lncRNA | 0.416042 | 0.84109 | 0.833186 | 0.197771 |
| LNC_002233 | XLOC_051097 | -- | intronic_lncRNA | 0.274196 | 0.67231 | 0.322232 | 0.175091 |
| LNC_002232 | XLOC_051092 | -- | intronic_lncRNA | 0.774217 | 0.693296 | 0.255835 | 0 |
| LNC_002231 | XLOC_051080 | -- | intronic_lncRNA | 1.015468 | 0.258954 | 0.062233 | 0 |
| LNC_002230 | XLOC_051076 | -- | intronic_lncRNA | 0.934313 | 0.616518 | 0.495101 | 0.448857 |
| LNC_003049 | XLOC_070156 | -- | intronic_lncRNA | 0.002652 | 0.001569 | 1.239301 | 0 |
| LNC_003048 | XLOC_070136 | -- | lincRNA | 0 | 0 | 0 | 0.461933 |
| LNC_003047 | XLOC_070126 | -- | intronic_lncRNA | 0.786434 | 0.445578 | 0.311728 | 0.792967 |
| LNC_003046 | XLOC_070114 | -- | intronic_lncRNA | 0.509059 | 0.056533 | 0.236347 | 0.175082 |
| LNC_003045 | XLOC_070113 | -- | intronic_lncRNA | 0.363796 | 0.293444 | 0.561717 | 0.524293 |
| LNC_003044 | XLOC_070086 | -- | intronic_lncRNA | 0.291679 | 0.179902 | 0.684923 | 0.29673 |
| LNC_003043 | XLOC_070076 | -- | intronic_lncRNA | 0.875266 | 0.486459 | 0.683634 | 0.079194 |
| LNC_003042 | XLOC_070076 | -- | intronic_lncRNA | 0.563134 | 0.809761 | 0.16915 | 0.74862 |
| LNC_003041 | XLOC_070055 | -- | intronic_lncRNA | 1.113559 | 0.603518 | 0.940381 | 1.197023 |
| LNC_003040 | XLOC_069941 | -- | intronic_lncRNA | 0.332476 | 0 | 0.480935 | 0.51184 |
| LNC_000587 | XLOC_010986 | -- | lincRNA | 0.735877 | 0.314941 | 1.138249 | 0.98025 |
| LNC_000586 | XLOC_010983 | -- | lincRNA | 0.461346 | 0.076719 | 0.528116 | 0.193347 |
| LNC_000585 | XLOC_010982 | -- | lincRNA | 1.264228 | 0.813126 | 0.935631 | 1.477823 |
| LNC_000584 | XLOC_010981 | -- | lincRNA | 0 | 0.553458 | 0 | 0 |
| LNC_000583 | XLOC_010884 | -- | intronic_lncRNA | 0.432941 | 0.572917 | 0.708027 | 0.482431 |
| LNC_000582 | XLOC_010873 | -- | intronic_lncRNA | 1.012208 | 0 | 0.122297 | 0 |
| LNC_000581 | XLOC_010865 | -- | intronic_lncRNA | 1.014082 | 0.242739 | 0.139228 | 0.085889 |
| LNC_000580 | XLOC_010865 | -- | intronic_lncRNA | 0.39223 | 0 | 0.753237 | 0.7485 |

| LNC_000589 | XLOC_011012 | -- | intronic_lncRNA | 0.050462 | 0.000253 | 0.866674 | 0.426055 |
| --- | --- | --- | --- | --- | --- | --- | --- |
| LNC_000588 | XLOC_010987 | -- | lincRNA | 0.174357 | 0.216361 | 0.532832 | 0.88154 |
| LNC_001768 | XLOC_038900 | -- | intronic_lncRNA | 0.573651 | 0.291313 | 0.78432 | 1.174812 |
| LNC_001769 | XLOC_038913 | -- | intronic_lncRNA | 0 | 0 | 0.458256 | 0.187089 |
| LNC_001764 | XLOC_038847 | -- | intronic_lncRNA | 6.345341 | 6.657331 | 7.316471 | 9.385572 |
| LNC_001765 | XLOC_038868 | -- | intronic_lncRNA | 1.86356 | 1.166309 | 1.771516 | 2.406838 |
| LNC_001766 | XLOC_038887 | -- | intronic_lncRNA | 0.618436 | 0.821808 | 1.21114 | 0.375088 |
| LNC_001767 | XLOC_038896 | -- | intronic_lncRNA | 4.687837 | 4.898035 | 5.220202 | 5.152802 |
| LNC_001760 | XLOC_038821 | -- | intronic_lncRNA | 0.054943 | 0 | 0.488359 | 0.078716 |
| LNC_001761 | XLOC_038822 | -- | intronic_lncRNA | 0.599312 | 0 | 0 | 0.048516 |
| LNC_001762 | XLOC_038847 | -- | intronic_lncRNA | 0.115864 | 0.071598 | 0.136285 | 0.083382 |
| LNC_001763 | XLOC_038847 | -- | intronic_lncRNA | 2.724688 | 1.93255 | 2.460405 | 2.32286 |
| LNC_005357 | XLOC_123187 | -- | intronic_lncRNA | 1.146382 | 0.451424 | 1.115109 | 1.169391 |
| LNC_004806 | XLOC_107266 | -- | intronic_lncRNA | 0.26755 | 0.192051 | 0 | 0.544275 |
| LNC_004807 | XLOC_107304 | -- | lincRNA | 0 | 0.480657 | 0 | 0.622716 |
| LNC_004804 | XLOC_107210 | -- | intronic_lncRNA | 0.927791 | 0.813653 | 0.523565 | 0.074782 |
| LNC_004805 | XLOC_107236 | -- | lincRNA | 0.148544 | 0.623446 | 0.154531 | 0.458924 |
| LNC_004802 | XLOC_107194 | -- | intronic_lncRNA | 0.647903 | 1.874621 | 0.693106 | 0.413583 |
| LNC_004803 | XLOC_107197 | -- | intronic_lncRNA | 0.896912 | 1.115486 | 0.824756 | 0.56273 |
| LNC_004800 | XLOC_107192 | -- | intronic_lncRNA | 0.22796 | 0.406802 | 0.841269 | 1.188036 |
| LNC_004801 | XLOC_107192 | -- | intronic_lncRNA | 0.452804 | 0 | 0 | 0 |
| LNC_004808 | XLOC_107315 | -- | intronic_lncRNA | 0.816346 | 0.386352 | 0.854099 | 0.783139 |
| LNC_004809 | XLOC_107369 | -- | lincRNA | 0.704576 | 0.449425 | 0.201273 | 0 |
| LNC_005356 | XLOC_123187 | -- | intronic_lncRNA | 0 | 0.907722 | 0.514722 | 0.219101 |
| LNC_005778 | XLOC_135638 | -- | intronic_lncRNA | 0.543267 | 0.10841 | 0 | 0 |
| LNC_000262 | XLOC_003750 | -- | intronic_lncRNA | 0.436549 | 0.128887 | 0.410067 | 0.139771 |
| LNC_000263 | XLOC_003780 | -- | lincRNA | 0.601341 | 0 | 0 | 0 |
| LNC_005340 | XLOC_122162 | -- | intronic_lncRNA | 0.186712 | 0.217825 | 0.391427 | 0.472313 |
| LNC_005711 | XLOC_133829 | -- | antisense_lncRNA | 0.387901 | 0.602315 | 0.400541 | 0.287246 |
| LNC_000261 | XLOC_003748 | -- | intronic_lncRNA | 0 | 0 | 0.179045 | 0.674113 |
| LNC_001359 | XLOC_029892 | -- | intronic_lncRNA | 0.829424 | 0.626983 | 0.99727 | 1.075804 |
| LNC_001358 | XLOC_029891 | -- | intronic_lncRNA | 1.698361 | 0.629665 | 1.818327 | 0.812651 |
| LNC_005710 | XLOC_133748 | -- | antisense_lncRNA | 0.854818 | 0.085888 | 0.180511 | 0.325359 |
| LNC_001355 | XLOC_029792 | -- | intronic_lncRNA | 0.762579 | 0.501359 | 1.287458 | 1.890583 |
| LNC_001354 | XLOC_029759 | -- | intronic_lncRNA | 0.778005 | 0.417435 | 0.682406 | 0.497973 |
| LNC_001357 | XLOC_029805 | -- | lincRNA | 1.100957 | 0.347544 | 0.09839 | 0.103041 |
| LNC_001356 | XLOC_029803 | -- | intronic_lncRNA | 0.181454 | 0 | 0.547766 | 0 |
| LNC_001351 | XLOC_029648 | -- | intronic_lncRNA | 0.584244 | 0.245987 | 0.533973 | 0.486105 |
| LNC_001350 | XLOC_029547 | -- | intronic_lncRNA | 0.307622 | 0.324136 | 0.765696 | 0.064333 |
| LNC_001353 | XLOC_029747 | -- | intronic_lncRNA | 0.087858 | 0.40511 | 0.41314 | 0.534759 |
| LNC_001352 | XLOC_029746 | -- | intronic_lncRNA | 0.548079 | 0.276187 | 0.631503 | 0.656739 |
| LNC_005712 | XLOC_133850 | -- | intronic_lncRNA | 2.463594 | 2.789279 | 3.134513 | 3.302629 |
| LNC_000264 | XLOC_003804 | -- | intronic_lncRNA | 1.112644 | 0.857804 | 1.802008 | 1.707493 |
| LNC_005715 | XLOC_133973 | -- | lincRNA | 0.615379 | 0.184482 | 0.724969 | 0.227677 |
| LNC_000265 | XLOC_003815 | -- | lincRNA | 0 | 0 | 0 | 0.525877 |
| LNC_005714 | XLOC_133934 | -- | lincRNA | 0.437332 | 0.474722 | 0 | 0.556876 |
| LNC_005717 | XLOC_134068 | -- | lincRNA | 0.189415 | 0.746215 | 0.106579 | 0 |
| LNC_005716 | XLOC_134010 | -- | intronic_lncRNA | 0.605079 | 0.487511 | 0.532661 | 0.571736 |
| LNC_000268 | XLOC_003937 | -- | lincRNA | 0.275387 | 0.684584 | 0.263871 | 0.285314 |
| LNC_000269 | XLOC_003943 | -- | intronic_lncRNA | 1.40712 | 0.419715 | 0.192044 | 0.924602 |
| LNC_005298 | XLOC_121269 | -- | intronic_lncRNA | 0 | 0.045391 | 0.455417 | 0.01485 |
| LNC_005299 | XLOC_121270 | -- | intronic_lncRNA | 0.273337 | 0.181715 | 0.171459 | 0.7855 |
| LNC_003607 | XLOC_076766 | -- | intronic_lncRNA | 0.236216 | 0.094958 | 0.663905 | 0.394955 |
| LNC_003606 | XLOC_076751 | -- | intronic_lncRNA | 0.61562 | 0.529923 | 0 | 0.137377 |
| LNC_003601 | XLOC_076323 | -- | intronic_lncRNA | 1.368859 | 0.981207 | 0.811007 | 0.697808 |
| LNC_003600 | XLOC_076309 | -- | intronic_lncRNA | 0.977499 | 0.304296 | 1.208406 | 1.558029 |
| LNC_003603 | XLOC_076570 | -- | antisense_lncRNA | 2.599854 | 0.71255 | 0.300927 | 0.080919 |
| LNC_003602 | XLOC_076511 | -- | intronic_lncRNA | 0.553413 | 0.270097 | 0.929099 | 0.624665 |
| LNC_005290 | XLOC_121213 | -- | intronic_lncRNA | 0.635725 | 0.716846 | 1.39059 | 0.839342 |
| LNC_005291 | XLOC_121223 | -- | intronic_lncRNA | 0.253227 | 0.808509 | 0.454253 | 0.363703 |
| LNC_005292 | XLOC_121242 | -- | intronic_lncRNA | 0.494841 | 0.470092 | 0.834934 | 0.801901 |
| LNC_005293 | XLOC_121242 | -- | intronic_lncRNA | 0.962976 | 0.811048 | 1.19802 | 1.09233 |
| LNC_003609 | XLOC_076943 | -- | intronic_lncRNA | 0.819777 | 0.174295 | 0.071957 | 0 |
| LNC_003608 | XLOC_076767 | -- | intronic_lncRNA | 0.058191 | 0.088945 | 0.900408 | 0.14254 |
| LNC_005296 | XLOC_121254 | -- | intronic_lncRNA | 37.32333 | 93.697548 | 56.648613 | 64.078156 |
| LNC_005297 | XLOC_121268 | -- | intronic_lncRNA | 0 | 0 | 0.538258 | 0 |
| LNC_006246 | XLOC_150040 | -- | intronic_lncRNA | 0.525192 | 0.075512 | 0.431382 | 0.567837 |
| LNC_002958 | XLOC_067788 | -- | intronic_lncRNA | 0.640547 | 0 | 0.509251 | 0.655501 |
| LNC_002959 | XLOC_067798 | -- | intronic_lncRNA | 1.006609 | 0.454911 | 0.946964 | 1.392769 |
| LNC_006247 | XLOC_150040 | -- | intronic_lncRNA | 0.661842 | 0.03903 | 0.266752 | 0.154007 |
| LNC_002952 | XLOC_067755 | -- | intronic_lncRNA | 0.435559 | 0.030847 | 0.469821 | 0.798908 |
| LNC_002953 | XLOC_067762 | -- | intronic_lncRNA | 0.692574 | 0.216415 | 0.131449 | 0.220417 |
| LNC_002950 | XLOC_067755 | -- | intronic_lncRNA | 1.06419 | 0.563366 | 0.83931 | 0.639983 |
| LNC_002951 | XLOC_067755 | -- | intronic_lncRNA | 0 | 1.357905 | 0.096672 | 0.149468 |
| LNC_002956 | XLOC_067779 | -- | intronic_lncRNA | 0.451898 | 0.388648 | 0.482582 | 0 |
| LNC_002957 | XLOC_067780 | -- | intronic_lncRNA | 0.703938 | 0.361065 | 0.653418 | 0.40742 |
| LNC_002954 | XLOC_067771 | -- | intronic_lncRNA | 0.621858 | 0.420293 | 1.30105 | 0.445395 |

| LNC_002955 | XLOC_067778 | -- | intronic_lncRNA | 0.458182 | 0.079315 | 0.42168 | 0.591627 |
| --- | --- | --- | --- | --- | --- | --- | --- |
| LNC_001979 | XLOC_044816 | -- | intronic_lncRNA | 0.619776 | 1.194872 | 0 | 0.456218 |
| LNC_001978 | XLOC_044793 | -- | intronic_lncRNA | 1.926983 | 1.278226 | 1.037906 | 0.756175 |
| LNC_006245 | XLOC_149885 | -- | lincRNA | 0.634121 | 0.235628 | 0.166663 | 0.135072 |
| LNC_001971 | XLOC_044430 | -- | intronic_lncRNA | 0.447375 | 1.442647 | 1.395318 | 0.009356 |
| LNC_001970 | XLOC_044430 | -- | intronic_lncRNA | 0 | 0 | 0 | 1.729475 |
| LNC_001973 | XLOC_044583 | -- | intronic_lncRNA | 0.041119 | 0.19465 | 0.357885 | 0 |
| LNC_001972 | XLOC_044508 | -- | lincRNA | 0.291745 | 0.218865 | 0.394086 | 1.36459 |
| LNC_001975 | XLOC_044728 | -- | intronic_lncRNA | 0.640013 | 0.354639 | 0.806256 | 0.90252 |
| LNC_001974 | XLOC_044723 | -- | antisense_lncRNA | 0.088044 | 0.635635 | 0.072419 | 0.170671 |
| LNC_001977 | XLOC_044729 | -- | intronic_lncRNA | 0.550779 | 0.275139 | 0.393655 | 0.201695 |
| LNC_001976 | XLOC_044729 | -- | intronic_lncRNA | 0.165612 | 0.771121 | 0.308978 | 0.1781 |
| LNC_006240 | XLOC_149587 | -- | lincRNA | 1.318472 | 0.980035 | 0.741441 | 0.900654 |
| LNC_006241 | XLOC_149645 | -- | lincRNA | 0.679151 | 0.281914 | 0.942567 | 0.743453 |
| LNC_000002 | XLOC_000061 | -- | lincRNA | 0.302012 | 0.634836 | 0.088324 | 0.21905 |
| LNC_000003 | XLOC_000070 | -- | lincRNA | 1.106977 | 1.43065 | 0.960461 | 1.700234 |
| LNC_000001 | XLOC_000035 | -- | lincRNA | 0.769628 | 0.313027 | 0.23742 | 0.477343 |
| LNC_000006 | XLOC_000164 | -- | lincRNA | 1.729508 | 1.005081 | 1.249209 | 2.072985 |
| LNC_000007 | XLOC_000195 | -- | lincRNA | 0 | 0 | 0 | 0.428887 |
| LNC_000004 | XLOC_000077 | -- | lincRNA | 1.265304 | 1.45201 | 0.915564 | 1.208748 |
| LNC_000005 | XLOC_000124 | -- | lincRNA | 1.00475 | 0.688297 | 0.451229 | 0.235002 |
| LNC_000008 | XLOC_000208 | -- | lincRNA | 0.169095 | 0.863053 | 0.308429 | 0.564036 |
| LNC_000009 | XLOC_000222 | -- | lincRNA | 1.331736 | 2.586246 | 1.675641 | 1.695475 |
| LNC_004653 | XLOC_103349 | -- | intronic_lncRNA | 0.247588 | 0.544594 | 0.074978 | 0.121266 |
| LNC_004652 | XLOC_103307 | -- | antisense_lncRNA | 0.751156 | 0.638609 | 0.220429 | 0.270872 |
| LNC_004651 | XLOC_103300 | -- | intronic_lncRNA | 0.341939 | 0.318386 | 0.078285 | 0 |
| LNC_004650 | XLOC_103300 | -- | intronic_lncRNA | 0.332884 | 0.267494 | 0.493962 | 0.044898 |
| LNC_004657 | XLOC_103402 | -- | intronic_lncRNA | 0.153873 | 0.462134 | 0.241952 | 0.62785 |
| LNC_004656 | XLOC_103401 | -- | intronic_lncRNA | 0 | 0.584972 | 0 | 0 |
| LNC_004655 | XLOC_103386 | -- | intronic_lncRNA | 0.248949 | 0 | 0.350021 | 0.502818 |
| LNC_004654 | XLOC_103353 | -- | lincRNA | 0.611707 | 0.455461 | 0.2788 | 0.202865 |
| LNC_004659 | XLOC_103413 | -- | intronic_lncRNA | 0.646251 | 0.550578 | 0.398247 | 0.414697 |
| LNC_004658 | XLOC_103405 | -- | intronic_lncRNA | 0 | 0 | 0.535371 | 0 |
| LNC_001519 | XLOC_032765 | -- | intronic_lncRNA | 0.830013 | 0.4071 | 1.653356 | 1.167175 |
| LNC_001518 | XLOC_032764 | -- | intronic_lncRNA | 0.314331 | 0.17185 | 0.390152 | 0.41745 |
| LNC_001513 | XLOC_032730 | -- | intronic_lncRNA | 0.355751 | 0.286865 | 0.956706 | 0 |
| LNC_001512 | XLOC_032728 | -- | intronic_lncRNA | 0.630095 | 0.106166 | 0.456441 | 0.643484 |
| LNC_001511 | XLOC_032727 | -- | antisense_lncRNA | 0.51593 | 0.085019 | 0.090214 | 0.059411 |
| LNC_001510 | XLOC_032695 | -- | intronic_lncRNA | 0 | 0 | 0 | 0.505777 |
| LNC_001517 | XLOC_032763 | -- | intronic_lncRNA | 0.503222 | 0.829518 | 0.990654 | 0.97712 |
| LNC_001516 | XLOC_032732 | -- | intronic_lncRNA | 0.550108 | 0.261631 | 0.738469 | 0.569509 |
| LNC_001515 | XLOC_032730 | -- | intronic_lncRNA | 0 | 0 | 0.000805 | 0.785778 |
| LNC_001514 | XLOC_032730 | -- | intronic_lncRNA | 0.837619 | 0 | 0.026809 | 0 |
| LNC_004740 | XLOC_105872 | -- | intronic_lncRNA | 0 | 0 | 0.56422 | 0 |
| LNC_005805 | XLOC_136686 | -- | intronic_lncRNA | 0.730612 | 0.578119 | 0.229165 | 0.2792 |
| LNC_005804 | XLOC_136685 | -- | intronic_lncRNA | 0.120843 | 0.585933 | 0.435801 | 0.022874 |
| LNC_003850 | XLOC_082531 | -- | intronic_lncRNA | 0 | 0.23267 | 0.408091 | 0.377214 |
| LNC_003851 | XLOC_082611 | -- | intronic_lncRNA | 0.094821 | 0.212 | 0.690859 | 0.681773 |
| LNC_003852 | XLOC_082664 | -- | intronic_lncRNA | 0.092416 | 0.14933 | 0.364517 | 0.952952 |
| LNC_003853 | XLOC_082696 | -- | intronic_lncRNA | 0.543585 | 0.445206 | 0.814997 | 1.082111 |
| LNC_003854 | XLOC_082697 | -- | intronic_lncRNA | 1.423783 | 0.848111 | 1.49159 | 1.337818 |
| LNC_003855 | XLOC_082725 | -- | intronic_lncRNA | 0.723885 | 1.154319 | 1.487424 | 0.831161 |
| LNC_003856 | XLOC_082739 | -- | lincRNA | 0.135185 | 0 | 0.546178 | 0 |
| LNC_003857 | XLOC_082746 | -- | intronic_lncRNA | 0.38042 | 0.75023 | 0.378246 | 0.439356 |
| LNC_003858 | XLOC_082794 | -- | lincRNA | 0.057318 | 0.244379 | 0.70171 | 0.046039 |
| LNC_003859 | XLOC_082820 | -- | intronic_lncRNA | 0.11541 | 0.634364 | 0.161019 | 0 |
| LNC_005807 | XLOC_136781 | -- | lincRNA | 0.626841 | 1.11582 | 1.546015 | 2.199524 |
| LNC_000392 | XLOC_006761 | -- | intronic_lncRNA | 0.582267 | 0 | 0 | 0.156805 |
| LNC_000393 | XLOC_006762 | -- | intronic_lncRNA | 0.398071 | 0.595883 | 0.357309 | 0 |
| LNC_000390 | XLOC_006635 | -- | lincRNA | 0.195503 | 0.520173 | 0.47217 | 0 |
| LNC_000391 | XLOC_006714 | -- | intronic_lncRNA | 0 | 1.032792 | 0 | 0 |
| LNC_000396 | XLOC_006905 | -- | intronic_lncRNA | 1.348556 | 0.888236 | 1.376263 | 1.271199 |
| LNC_000397 | XLOC_006935 | -- | intronic_lncRNA | 0.500965 | 0 | 0.785708 | 0.695176 |
| LNC_000394 | XLOC_006810 | -- | intronic_lncRNA | 0 | 0.339377 | 0.934122 | 0 |
| LNC_000395 | XLOC_006901 | -- | antisense_lncRNA | 0.956225 | 0.642656 | 0.708204 | 0.813259 |
| LNC_000398 | XLOC_007021 | -- | intronic_lncRNA | 0.768912 | 0.500451 | 0.132137 | 0.595977 |
| LNC_000399 | XLOC_007021 | -- | antisense_lncRNA | 0.369913 | 1.21268 | 0.2554 | 0.653427 |
| LNC_002721 | XLOC_063267 | -- | intronic_lncRNA | 0.161546 | 0.622263 | 0.407934 | 0.327936 |
| LNC_002720 | XLOC_063202 | -- | lincRNA | 0 | 0 | 1.089189 | 0.249222 |
| LNC_002723 | XLOC_063276 | -- | intronic_lncRNA | 0.163286 | 0.267604 | 0 | 0.395757 |
| LNC_002722 | XLOC_063267 | -- | intronic_lncRNA | 0.18199 | 0.072002 | 0.093987 | 0.437036 |
| LNC_002725 | XLOC_063358 | -- | intronic_lncRNA | 0.560736 | 0 | 0 | 0 |
| LNC_002724 | XLOC_063352 | -- | intronic_lncRNA | 0.128998 | 0.527295 | 0.300504 | 0.776912 |
| LNC_002727 | XLOC_063388 | -- | intronic_lncRNA | 0.179412 | 0 | 0.434194 | 0.241436 |
| LNC_002726 | XLOC_063359 | -- | intronic_lncRNA | 0 | 0.51555 | 0 | 0.173136 |
| LNC_002729 | XLOC_063471 | -- | intronic_lncRNA | 0.992527 | 0.845919 | 0.423009 | 0.872741 |
| LNC_002728 | XLOC_063432 | -- | lincRNA | 0.830446 | 0.42122 | 0.085965 | 0.095974 |

| LNC_002899 | XLOC_066838 | -- | intronic_lncRNA | 0.041102 | 0 | 0.438984 | 0 |
| --- | --- | --- | --- | --- | --- | --- | --- |
| LNC_002898 | XLOC_066837 | -- | intronic_lncRNA | 0.550992 | 0 | 0.396993 | 0 |
| LNC_002893 | XLOC_066652 | -- | intronic_lncRNA | 0.607263 | 0 | 0.39458 | 0.074269 |
| LNC_002892 | XLOC_066621 | -- | intronic_lncRNA | 0.105073 | 0.071428 | 0.343222 | 0.289182 |
| LNC_002891 | XLOC_066601 | -- | intronic_lncRNA | 0 | 0.08467 | 0.342856 | 0.365865 |
| LNC_002890 | XLOC_066585 | -- | intronic_lncRNA | 0.267136 | 0.251228 | 1.096454 | 0.451323 |
| LNC_002897 | XLOC_066836 | -- | intronic_lncRNA | 0.795936 | 0.464919 | 0.577935 | 0.66307 |
| LNC_002896 | XLOC_066713 | -- | lincRNA | 0.376451 | 0.45999 | 0.17725 | 0.254646 |
| LNC_002895 | XLOC_066709 | -- | intronic_lncRNA | 0.358005 | 0 | 0.678474 | 0.444286 |
| LNC_002894 | XLOC_066656 | -- | intronic_lncRNA | 0.531522 | 0.199539 | 0.416206 | 0.561907 |
| LNC_004008 | XLOC_086452 | -- | intronic_lncRNA | 0.202778 | 0.529637 | 0.327707 | 0.301802 |
| LNC_004009 | XLOC_086453 | -- | intronic_lncRNA | 0.390523 | 0.111968 | 0.032644 | 0.453312 |
| LNC_004006 | XLOC_086359 | -- | lincRNA | 0.862925 | 0.087086 | 0 | 0.774728 |
| LNC_004007 | XLOC_086403 | -- | lincRNA | 0.257864 | 0.355207 | 0.594368 | 0.341039 |
| LNC_004004 | XLOC_086304 | -- | intronic_lncRNA | 0.31769 | 0.581546 | 0.154596 | 0 |
| LNC_004005 | XLOC_086332 | -- | intronic_lncRNA | 0.655444 | 0.292663 | 0.59389 | 0.683755 |
| LNC_004002 | XLOC_086301 | -- | intronic_lncRNA | 0.738445 | 0.777891 | 0.257673 | 0.62371 |
| LNC_004003 | XLOC_086302 | -- | intronic_lncRNA | 0.571272 | 0.134622 | 0.404932 | 0.516198 |
| LNC_004000 | XLOC_086253 | -- | lincRNA | 0 | 0 | 0.463254 | 0.458007 |
| LNC_004001 | XLOC_086282 | -- | intronic_lncRNA | 0.728146 | 0.234225 | 0.613318 | 0.576517 |
| LNC_005568 | XLOC_129133 | -- | intronic_lncRNA | 0 | 0.365781 | 0.583937 | 0.668515 |
| LNC_005719 | XLOC_134096 | -- | intronic_lncRNA | 1.502931 | 0.807215 | 1.097332 | 1.016796 |
| LNC_005718 | XLOC_134096 | -- | intronic_lncRNA | 1.762451 | 0.619181 | 0.682123 | 0.822116 |
| LNC_005569 | XLOC_129133 | -- | intronic_lncRNA | 0 | 0.327493 | 0.259651 | 0 |
| LNC_000949 | XLOC_020297 | -- | intronic_lncRNA | 0.491954 | 0.851324 | 0.310624 | 0.497206 |
| LNC_000948 | XLOC_020297 | -- | intronic_lncRNA | 1.918354 | 1.240882 | 1.178352 | 0.816257 |
| LNC_000947 | XLOC_020248 | -- | intronic_lncRNA | 0.846584 | 0.621375 | 1.113371 | 1.132465 |
| LNC_000946 | XLOC_020248 | -- | intronic_lncRNA | 0 | 1.065556 | 0 | 0 |
| LNC_000945 | XLOC_020246 | -- | lincRNA | 0.527857 | 0.691235 | 0.890342 | 0.757561 |
| LNC_000944 | XLOC_020211 | -- | intronic_lncRNA | 0.57598 | 0.531071 | 0.548316 | 1.000831 |
| LNC_000943 | XLOC_020189 | -- | intronic_lncRNA | 0.663117 | 0.309784 | 0.370582 | 0.179333 |
| LNC_000942 | XLOC_020189 | -- | intronic_lncRNA | 0.855867 | 0.322598 | 0.364872 | 1.847721 |
| LNC_000941 | XLOC_020188 | -- | intronic_lncRNA | 0.564178 | 0.866161 | 0.624575 | 0.667721 |
| LNC_000940 | XLOC_020167 | -- | intronic_lncRNA | 0.556908 | 0.21142 | 0.57435 | 0.59326 |
| LNC_005841 | XLOC_137506 | -- | intronic_lncRNA | 0.394936 | 0.539731 | 0.461553 | 0.505019 |
| LNC_005840 | XLOC_137493 | -- | intronic_lncRNA | 0.060005 | 0.496424 | 0 | 0.125235 |
| LNC_005843 | XLOC_137650 | -- | intronic_lncRNA | 0.237749 | 1.069054 | 0.411439 | 0.299322 |
| LNC_005842 | XLOC_137574 | -- | lincRNA | 0.745371 | 0 | 0.097571 | 0 |
| LNC_005845 | XLOC_137654 | -- | lincRNA | 0 | 0.305205 | 0.406249 | 0.500523 |
| LNC_005844 | XLOC_137651 | -- | intronic_lncRNA | 0.485173 | 0.257185 | 0.253495 | 0.766651 |
| LNC_005847 | XLOC_137701 | -- | intronic_lncRNA | 0.293404 | 0.346682 | 0.417655 | 0.458797 |
| LNC_005846 | XLOC_137683 | -- | intronic_lncRNA | 0.438724 | 0.065457 | 0.258745 | 0.059959 |
| LNC_005849 | XLOC_137719 | -- | intronic_lncRNA | 0 | 0 | 0.555216 | 0 |
| LNC_005848 | XLOC_137707 | -- | intronic_lncRNA | 2.888717 | 7.645089 | 9.012365 | 11.846541 |
| LNC_005562 | XLOC_128962 | -- | intronic_lncRNA | 1.549569 | 0.724996 | 0.926123 | 0.664561 |
| LNC_005563 | XLOC_128970 | -- | intronic_lncRNA | 0.197558 | 0 | 0.056818 | 0.482498 |
| LNC_005560 | XLOC_128949 | -- | intronic_lncRNA | 0 | 0.766511 | 0 | 0 |
| LNC_005561 | XLOC_128961 | -- | intronic_lncRNA | 1.03296 | 0.617953 | 0.41464 | 0.159906 |
| LNC_005566 | XLOC_129069 | -- | lincRNA | 0.180847 | 0 | 0.408545 | 0 |
| LNC_005567 | XLOC_129069 | -- | lincRNA | 0.490339 | 0.103566 | 0 | 0.550988 |
| LNC_005564 | XLOC_129041 | -- | lincRNA | 1.134465 | 0.524488 | 0.299848 | 0.423174 |
| LNC_005565 | XLOC_129042 | -- | intronic_lncRNA | 0.610935 | 0.110897 | 0.202075 | 0 |
| LNC_006398 | XLOC_153385 | -- | intronic_lncRNA | 0.208724 | 0.397075 | 0.116093 | 0.295101 |
| LNC_006399 | XLOC_153404 | -- | intronic_lncRNA | 0.634379 | 0.521434 | 0.845291 | 0.843726 |
| LNC_006394 | XLOC_153376 | -- | antisense_lncRNA | 0 | 0 | 0 | 0.543835 |
| LNC_006395 | XLOC_153378 | -- | intronic_lncRNA | 4.500595 | 5.510449 | 1.675558 | 1.59147 |
| LNC_006396 | XLOC_153383 | -- | intronic_lncRNA | 0.096542 | 0 | 0.150961 | 0.611898 |
| LNC_006397 | XLOC_153384 | -- | intronic_lncRNA | 0.162373 | 0.540751 | 0.179114 | 0.300844 |
| LNC_006390 | XLOC_153283 | -- | intronic_lncRNA | 0.736473 | 0 | 0.398062 | 0.187941 |
| LNC_006391 | XLOC_153337 | -- | intronic_lncRNA | 0.126639 | 0.006897 | 0.166335 | 0.494139 |
| LNC_006392 | XLOC_153337 | -- | intronic_lncRNA | 0 | 0.865267 | 0.363395 | 0.352831 |
| LNC_006393 | XLOC_153337 | -- | intronic_lncRNA | 0.379961 | 0.145513 | 0.395412 | 0.249395 |
| LNC_001706 | XLOC_037333 | -- | intronic_lncRNA | 0.746203 | 0.256575 | 0.49206 | 0.416618 |
| LNC_001707 | XLOC_037492 | -- | intronic_lncRNA | 0.563553 | 0.496144 | 0.769177 | 0.768079 |
| LNC_001704 | XLOC_037324 | -- | intronic_lncRNA | 0 | 0 | 0.407289 | 0 |
| LNC_001705 | XLOC_037326 | -- | intronic_lncRNA | 0.068468 | 0 | 0.691568 | 0.071654 |
| LNC_001702 | XLOC_037306 | -- | antisense_lncRNA | 0.263835 | 0.128744 | 0.533054 | 0.131525 |
| LNC_001703 | XLOC_037323 | -- | intronic_lncRNA | 0.159208 | 0.084395 | 0.595253 | 0.352401 |
| LNC_001700 | XLOC_037248 | -- | intronic_lncRNA | 0.211078 | 0 | 0.462013 | 0.188879 |
| LNC_001701 | XLOC_037253 | -- | intronic_lncRNA | 0.970996 | 0 | 0 | 0 |
| LNC_001708 | XLOC_037493 | -- | antisense_lncRNA | 0 | 0.014893 | 0.650821 | 0.169408 |
| LNC_001709 | XLOC_037552 | -- | intronic_lncRNA | 0.31785 | 0 | 0.421267 | 0 |
| LNC_004828 | XLOC_108236 | -- | intronic_lncRNA | 1.068401 | 0.440962 | 0.579892 | 0.79649 |
| LNC_004829 | XLOC_108237 | -- | intronic_lncRNA | 0.723605 | 0.172474 | 0.287805 | 0 |
| LNC_004398 | XLOC_095983 | -- | intronic_lncRNA | 0.178608 | 0 | 0.39722 | 0.099288 |
| LNC_004399 | XLOC_095986 | -- | intronic_lncRNA | 0.213892 | 0.63688 | 0.086474 | 0.325783 |
| LNC_004396 | XLOC_095912 | -- | intronic_lncRNA | 0.744548 | 0 | 0.554779 | 0 |

| LNC_004397 | XLOC_095942 | -- | intronic_lncRNA | 0 | 0 | 0.427012 | 0 |
| --- | --- | --- | --- | --- | --- | --- | --- |
| LNC_004394 | XLOC_095810 | -- | intronic_lncRNA | 0.461449 | 0.433205 | 0 | 0.521813 |
| LNC_004395 | XLOC_095887 | -- | intronic_lncRNA | 0.595369 | 0 | 0 | 0 |
| LNC_004392 | XLOC_095730 | -- | intronic_lncRNA | 0 | 0 | 0.406092 | 0.101054 |
| LNC_004393 | XLOC_095731 | -- | intronic_lncRNA | 0 | 0 | 0 | 0.396314 |
| LNC_004826 | XLOC_108194 | -- | intronic_lncRNA | 0.608106 | 0.216979 | 0.368583 | 0.092284 |
| LNC_004391 | XLOC_095628 | -- | intronic_lncRNA | 0.69139 | 0.188309 | 0.762012 | 1.676748 |
| LNC_001377 | XLOC_030281 | -- | intronic_lncRNA | 0 | 0.150025 | 0.069683 | 0.462326 |
| LNC_001376 | XLOC_030256 | -- | intronic_lncRNA | 0 | 0 | 0.735813 | 0 |
| LNC_001375 | XLOC_030242 | -- | intronic_lncRNA | 0.560703 | 0.177792 | 0 | 0.170156 |
| LNC_001374 | XLOC_030229 | -- | intronic_lncRNA | 0.026604 | 0.596805 | 0.342163 | 0.583547 |
| LNC_001373 | XLOC_030226 | -- | antisense_lncRNA | 0.875963 | 0 | 0 | 0.748814 |
| LNC_001372 | XLOC_030224 | -- | intronic_lncRNA | 1.05316 | 0.243918 | 0.253515 | 0.76812 |
| LNC_001371 | XLOC_030223 | -- | intronic_lncRNA | 0.089514 | 0 | 0.323423 | 0.290042 |
| LNC_001370 | XLOC_030185 | -- | intronic_lncRNA | 0.564365 | 0.674031 | 0.561375 | 0.705512 |
| LNC_001379 | XLOC_030292 | -- | intronic_lncRNA | 0.426989 | 0.550836 | 1.291362 | 1.581995 |
| LNC_001378 | XLOC_030283 | -- | intronic_lncRNA | 0.549637 | 1.149457 | 1.230064 | 1.023101 |
| LNC_003629 | XLOC_077427 | -- | intronic_lncRNA | 0.505911 | 0.741297 | 0.981241 | 1.13015 |
| LNC_003628 | XLOC_077423 | -- | lincRNA | 0.511404 | 0.384193 | 0.310704 | 0.669063 |
| LNC_003627 | XLOC_077419 | -- | intronic_lncRNA | 0.253833 | 0.434995 | 0.146785 | 0.337156 |
| LNC_003626 | XLOC_077413 | -- | intronic_lncRNA | 2.71843 | 1.548199 | 1.864149 | 1.80722 |
| LNC_003625 | XLOC_077379 | -- | intronic_lncRNA | 0.272009 | 2.061244 | 0.802716 | 0.600361 |
| LNC_003624 | XLOC_077187 | -- | intronic_lncRNA | 0.903531 | 0 | 0 | 0.037522 |
| LNC_003623 | XLOC_077187 | -- | intronic_lncRNA | 0.984597 | 0.596152 | 0.833721 | 0.821211 |
| LNC_003622 | XLOC_077183 | -- | intronic_lncRNA | 0.640358 | 0.174509 | 0.518535 | 0.318831 |
| LNC_003621 | XLOC_077177 | -- | intronic_lncRNA | 0.11345 | 0.212425 | 0.693982 | 0.534166 |
| LNC_003620 | XLOC_077136 | -- | lincRNA | 0 | 0 | 0.393065 | 0 |
| LNC_005272 | XLOC_120782 | -- | intronic_lncRNA | 0.970498 | 0 | 0.014708 | 0 |
| LNC_005273 | XLOC_120785 | -- | antisense_lncRNA | 2.033985 | 3.915652 | 4.51021 | 4.995156 |
| LNC_005270 | XLOC_120782 | -- | antisense_lncRNA | 0.888659 | 0.055999 | 0.896233 | 0 |
| LNC_005271 | XLOC_120782 | -- | intronic_lncRNA | 0.004429 | 1.951356 | 1.136433 | 2.27991 |
| LNC_005276 | XLOC_120820 | -- | intronic_lncRNA | 0.013042 | 0 | 0 | 0.861744 |
| LNC_005277 | XLOC_120831 | -- | intronic_lncRNA | 0.30221 | 0.234435 | 0.811329 | 0.634089 |
| LNC_005274 | XLOC_120796 | -- | antisense_lncRNA | 0.347617 | 1.167916 | 0.682313 | 0.263711 |
| LNC_005275 | XLOC_120820 | -- | intronic_lncRNA | 1.026699 | 0.796546 | 0.65443 | 0.025365 |
| LNC_005278 | XLOC_120832 | -- | intronic_lncRNA | 1.942309 | 0.904668 | 2.0539 | 1.808961 |
| LNC_005279 | XLOC_120834 | -- | intronic_lncRNA | 0.523203 | 0.573393 | 0.466408 | 0.608393 |
| LNC_006455 | XLOC_155278 | -- | intronic_lncRNA | 0.058748 | 0.775821 | 0.562532 | 0.806542 |
| LNC_004820 | XLOC_107731 | -- | intronic_lncRNA | 0.867706 | 1.292933 | 0.851978 | 0.940771 |
| LNC_006454 | XLOC_155276 | -- | intronic_lncRNA | 0.495479 | 0.581098 | 0.644229 | 0.244801 |
| LNC_002978 | XLOC_068086 | -- | intronic_lncRNA | 0 | 0 | 0.004871 | 1.934568 |
| LNC_002979 | XLOC_068086 | -- | intronic_lncRNA | 0.415019 | 0.216132 | 0.341693 | 2.100278 |
| LNC_004821 | XLOC_107755 | -- | intronic_lncRNA | 2.445447 | 0.966431 | 1.771399 | 1.68681 |
| LNC_002974 | XLOC_068070 | -- | intronic_lncRNA | 0.712251 | 0.356482 | 0.308665 | 1.300379 |
| LNC_002975 | XLOC_068073 | -- | intronic_lncRNA | 0.805173 | 0.75837 | 0.754923 | 1.325332 |
| LNC_002976 | XLOC_068078 | -- | intronic_lncRNA | 0 | 0.006826 | 0 | 0.005866 |
| LNC_002977 | XLOC_068086 | -- | intronic_lncRNA | 0 | 0 | 0.738171 | 4.128465 |
| LNC_002970 | XLOC_068061 | -- | intronic_lncRNA | 0.918296 | 0.7067 | 1.013551 | 0.864325 |
| LNC_002971 | XLOC_068068 | -- | intronic_lncRNA | 0.550959 | 0.371548 | 0.246917 | 0.545133 |
| LNC_002972 | XLOC_068068 | -- | intronic_lncRNA | 0.770276 | 0.374752 | 0.441572 | 0.189965 |
| LNC_002973 | XLOC_068069 | -- | intronic_lncRNA | 0.272909 | 0.084412 | 0.151058 | 0.435457 |
| LNC_001953 | XLOC_043972 | -- | intronic_lncRNA | 0 | 0.138704 | 0.060452 | 0.540147 |
| LNC_001952 | XLOC_043965 | -- | intronic_lncRNA | 0.611265 | 0.453297 | 0.333481 | 0.291798 |
| LNC_001951 | XLOC_043961 | -- | intronic_lncRNA | 0.641968 | 0.200548 | 0.648384 | 0.134136 |
| LNC_001950 | XLOC_043960 | -- | intronic_lncRNA | 0.814148 | 0.857153 | 1.310612 | 1.125454 |
| LNC_001957 | XLOC_044115 | -- | lincRNA | 0.27248 | 0.106904 | 1.036907 | 0.700108 |
| LNC_001956 | XLOC_044068 | -- | intronic_lncRNA | 0.667 | 0.341878 | 1.232722 | 1.339518 |
| LNC_001955 | XLOC_044042 | -- | intronic_lncRNA | 0.36152 | 0.134819 | 0.842988 | 0.3187 |
| LNC_001954 | XLOC_044016 | -- | lincRNA | 0.039745 | 0 | 0 | 1.198289 |
| LNC_004824 | XLOC_107996 | -- | lincRNA | 0.622787 | 0 | 0 | 0.08907 |
| LNC_001959 | XLOC_044129 | -- | intronic_lncRNA | 1.044245 | 0.086825 | 0 | 0 |
| LNC_001958 | XLOC_044124 | -- | intronic_lncRNA | 0.757089 | 0.989631 | 0.453072 | 0.317625 |
| LNC_000123 | XLOC_001021 | -- | intronic_lncRNA | 0.642316 | 0.443273 | 0.63688 | 0.123201 |
| LNC_004825 | XLOC_108153 | -- | intronic_lncRNA | 0.737518 | 0.081244 | 0.59197 | 0.465201 |
| LNC_000122 | XLOC_001018 | -- | intronic_lncRNA | 0.260529 | 0.629031 | 0.629447 | 0.981276 |
| LNC_004390 | XLOC_095620 | -- | intronic_lncRNA | 0.113454 | 0.347926 | 0.595762 | 0.612015 |
| LNC_005405 | XLOC_124664 | -- | intronic_lncRNA | 1.118146 | 0.52319 | 0.78689 | 0.963673 |
| LNC_004827 | XLOC_108235 | -- | intronic_lncRNA | 0 | 0.511759 | 0.552603 | 0.388137 |
| LNC_000124 | XLOC_001045 | -- | intronic_lncRNA | 0.151126 | 0 | 0.209618 | 0.39245 |
| LNC_000024 | XLOC_000302 | -- | lincRNA | 0 | 0 | 0 | 11.518198 |
| LNC_000025 | XLOC_000302 | -- | lincRNA | 12.852495 | 0.414899 | 7.120664 | 5.520719 |
| LNC_000026 | XLOC_000302 | -- | lincRNA | 0 | 0.029388 | 20.261267 | 7.970026 |
| LNC_000027 | XLOC_000303 | -- | lincRNA | 0.189929 | 2.139877 | 8.3458 | 13.68312 |
| LNC_000020 | XLOC_000289 | -- | lincRNA | 32.779865 | 65.492271 | 1.285951 | 26.836733 |
| LNC_000021 | XLOC_000289 | -- | lincRNA | 16.765375 | 0 | 0.242524 | 13.935709 |
| LNC_000022 | XLOC_000291 | -- | lincRNA | 11.95527 | 9.187792 | 27.436438 | 21.842533 |
| LNC_000023 | XLOC_000297 | -- | lincRNA | 5.924429 | 2.578945 | 2.705775 | 2.503715 |

| LNC_000126 | XLOC_001139 | -- | intronic_lncRNA | 0.349149 | 0.346102 | 0.48672 | 0.598418 |
| --- | --- | --- | --- | --- | --- | --- | --- |
| LNC_000028 | XLOC_000303 | -- | lincRNA | 26.127609 | 15.073671 | 20.190176 | 10.215998 |
| LNC_000029 | XLOC_000306 | -- | lincRNA | 10.833764 | 4.446321 | 7.914211 | 6.749975 |
| LNC_005409 | XLOC_124788 | -- | intronic_lncRNA | 0.40947 | 0.280427 | 0.340789 | 0.276255 |
| LNC_000128 | XLOC_001287 | -- | intronic_lncRNA | 0.213883 | 0.881761 | 0.270065 | 0.042868 |
| LNC_004823 | XLOC_107962 | -- | intronic_lncRNA | 0.286472 | 0.165047 | 0.607387 | 0.954783 |
| LNC_004635 | XLOC_103091 | -- | intronic_lncRNA | 0.696546 | 0 | 0 | 0 |
| LNC_004634 | XLOC_103069 | -- | lincRNA | 0.187616 | 0.37514 | 0.381672 | 0.54548 |
| LNC_004637 | XLOC_103122 | -- | intronic_lncRNA | 0.49773 | 0.300464 | 0.572568 | 0.784537 |
| LNC_004636 | XLOC_103092 | -- | intronic_lncRNA | 0.490819 | 0.564814 | 0.714274 | 0.280083 |
| LNC_004631 | XLOC_103006 | -- | intronic_lncRNA | 0.39532 | 0.36973 | 0.281333 | 0.530541 |
| LNC_004630 | XLOC_102992 | -- | intronic_lncRNA | 0.216298 | 0.100172 | 0.16225 | 0.405013 |
| LNC_004633 | XLOC_103050 | -- | intronic_lncRNA | 0.129039 | 0.305547 | 0.436298 | 0.596007 |
| LNC_004632 | XLOC_103035 | -- | lincRNA | 0.295574 | 0 | 0.636368 | 0.159197 |
| LNC_004639 | XLOC_103123 | -- | intronic_lncRNA | 0.245273 | 0.221882 | 0.214492 | 0.792627 |
| LNC_004638 | XLOC_103122 | -- | intronic_lncRNA | 0.53442 | 0.095924 | 0.833291 | 0.037973 |
| LNC_001579 | XLOC_034279 | -- | intronic_lncRNA | 0 | 0 | 0.425853 | 0.534391 |
| LNC_001578 | XLOC_034234 | -- | intronic_lncRNA | 0.117791 | 0.974882 | 0.028078 | 0.189467 |
| LNC_001575 | XLOC_034193 | -- | intronic_lncRNA | 1.334213 | 1.09119 | 0.789056 | 0.764931 |
| LNC_001574 | XLOC_034167 | -- | intronic_lncRNA | 0.513595 | 0 | 0.990358 | 0.607685 |
| LNC_001577 | XLOC_034222 | -- | intronic_lncRNA | 0.382917 | 0.533125 | 0.101669 | 0.508259 |
| LNC_001576 | XLOC_034206 | -- | intronic_lncRNA | 0.877341 | 0.499479 | 0.542425 | 0.464771 |
| LNC_001571 | XLOC_034120 | -- | intronic_lncRNA | 0.470204 | 0 | 0.539191 | 0 |
| LNC_001570 | XLOC_034111 | -- | intronic_lncRNA | 1.343384 | 0.925867 | 0.759573 | 0.285863 |
| LNC_001573 | XLOC_034145 | -- | intronic_lncRNA | 2.806143 | 2.216655 | 2.613563 | 2.785614 |
| LNC_001572 | XLOC_034127 | -- | intronic_lncRNA | 0.193291 | 0.264051 | 0.407032 | 0.538071 |
| LNC_006278 | XLOC_150558 | -- | antisense_lncRNA | 0 | 0.492912 | 0.214098 | 0 |
| LNC_002378 | XLOC_055011 | -- | intronic_lncRNA | 0.536084 | 0.532735 | 0.875927 | 0.647918 |
| LNC_002379 | XLOC_055057 | -- | intronic_lncRNA | 0.7751 | 0.652483 | 0.593579 | 0.496015 |
| LNC_002372 | XLOC_054937 | -- | intronic_lncRNA | 0.206209 | 0 | 0.432293 | 0 |
| LNC_002373 | XLOC_054942 | -- | intronic_lncRNA | 0.316484 | 0.681009 | 0.18919 | 0.079837 |
| LNC_002370 | XLOC_054859 | -- | intronic_lncRNA | 0.703181 | 0 | 0 | 0 |
| LNC_002371 | XLOC_054937 | -- | intronic_lncRNA | 0 | 1.542128 | 0.2546 | 0.1655 |
| LNC_002376 | XLOC_054992 | -- | intronic_lncRNA | 19.590021 | 30.203794 | 23.003477 | 25.233042 |
| LNC_002377 | XLOC_055010 | -- | antisense_lncRNA | 0 | 0.495721 | 0 | 0 |
| LNC_002374 | XLOC_054987 | -- | intronic_lncRNA | 0.530891 | 0.00503 | 0 | 0 |
| LNC_002375 | XLOC_054988 | -- | intronic_lncRNA | 0.243832 | 0.255937 | 0.512271 | 0.432334 |
| LNC_003368 | XLOC_075175 | -- | lincRNA | 0.889884 | 1.431167 | 0.216509 | 0.331736 |
| LNC_003369 | XLOC_075175 | -- | lincRNA | 3.587881 | 0.496579 | 0.272133 | 0.301217 |
| LNC_003362 | XLOC_075175 | -- | lincRNA | 5.812919 | 1.393487 | 1.451946 | 0.847519 |
| LNC_003363 | XLOC_075175 | -- | lincRNA | 5.2424 | 2.065685 | 1.334112 | 1.237721 |
| LNC_003360 | XLOC_075175 | -- | lincRNA | 2.520218 | 0 | 0 | 0.968439 |
| LNC_003361 | XLOC_075175 | -- | lincRNA | 3.069888 | 2.017106 | 0.688842 | 0.676673 |
| LNC_003366 | XLOC_075175 | -- | lincRNA | 0 | 1.58618 | 0 | 0.754651 |
| LNC_003367 | XLOC_075175 | -- | lincRNA | 3.089289 | 0.127987 | 0.417132 | 0.496366 |
| LNC_003364 | XLOC_075175 | -- | lincRNA | 2.882452 | 1.460979 | 0.369853 | 0.391382 |
| LNC_003365 | XLOC_075175 | -- | lincRNA | 3.359506 | 1.898944 | 0.874095 | 0.767448 |
| LNC_000622 | XLOC_011684 | -- | intronic_lncRNA | 1.179281 | 1.461187 | 4.004652 | 4.290369 |
| LNC_000623 | XLOC_011710 | -- | intronic_lncRNA | 0.57714 | 0 | 0.151534 | 0.205647 |
| LNC_000620 | XLOC_011684 | -- | intronic_lncRNA | 2.667795 | 2.893794 | 5.441649 | 4.989371 |
| LNC_000621 | XLOC_011684 | -- | intronic_lncRNA | 0.991432 | 0.791813 | 0.738432 | 0.705793 |
| LNC_000626 | XLOC_011804 | -- | intronic_lncRNA | 0.249604 | 0.063038 | 0.440507 | 0.802669 |
| LNC_000627 | XLOC_011843 | -- | intronic_lncRNA | 0.689022 | 0.819498 | 0.467235 | 0.014415 |
| LNC_000624 | XLOC_011739 | -- | intronic_lncRNA | 0.98579 | 1.619758 | 1.423552 | 2.0273 |
| LNC_000625 | XLOC_011748 | -- | antisense_lncRNA | 0 | 0.024929 | 0.515982 | 0.029865 |
| LNC_000628 | XLOC_011843 | -- | intronic_lncRNA | 0 | 0.344806 | 0.540649 | 0.964985 |
| LNC_000629 | XLOC_011870 | -- | antisense_lncRNA | 0.656671 | 0 | 0.168514 | 0 |
| LNC_002709 | XLOC_062923 | -- | intronic_lncRNA | 1.076597 | 1.799972 | 1.121661 | 0.960728 |
| LNC_002708 | XLOC_062864 | -- | intronic_lncRNA | 0.408922 | 0.634928 | 0.696461 | 1.137547 |
| LNC_002703 | XLOC_062752 | -- | intronic_lncRNA | 0.162071 | 0.784464 | 0 | 0.696591 |
| LNC_002702 | XLOC_062750 | -- | intronic_lncRNA | 1.127829 | 0.702247 | 0.753288 | 1.405154 |
| LNC_002701 | XLOC_062743 | -- | intronic_lncRNA | 0.621806 | 0 | 0.040703 | 0 |
| LNC_002700 | XLOC_062741 | -- | intronic_lncRNA | 0.544534 | 1.276161 | 1.140425 | 1.347845 |
| LNC_002707 | XLOC_062835 | -- | intronic_lncRNA | 0.700357 | 0.403744 | 0 | 0.077158 |
| LNC_002706 | XLOC_062829 | -- | intronic_lncRNA | 0.459704 | 0 | 0.08139 | 0.532783 |
| LNC_002705 | XLOC_062819 | -- | intronic_lncRNA | 0 | 0.67968 | 0 | 0.18345 |
| LNC_002704 | XLOC_062782 | -- | intronic_lncRNA | 0.199355 | 0.556942 | 0.722635 | 0.250169 |
| LNC_004020 | XLOC_086831 | -- | intronic_lncRNA | 0 | 0 | 0 | 0.826261 |
| LNC_004021 | XLOC_086831 | -- | intronic_lncRNA | 1.880803 | 1.883922 | 0.938636 | 0.805899 |
| LNC_004022 | XLOC_086831 | -- | intronic_lncRNA | 1.643979 | 1.617086 | 2.057403 | 1.702344 |
| LNC_004023 | XLOC_086860 | -- | intronic_lncRNA | 0.748742 | 1.322529 | 0.98196 | 0.439936 |
| LNC_004024 | XLOC_086915 | -- | lincRNA | 1.134946 | 0.378609 | 0.891736 | 1.119909 |
| LNC_004025 | XLOC_086919 | -- | intronic_lncRNA | 0.43818 | 0 | 0.216457 | 0.092616 |
| LNC_004026 | XLOC_086930 | -- | intronic_lncRNA | 0.361371 | 0.145378 | 0.732958 | 0.67508 |
| LNC_004027 | XLOC_086966 | -- | intronic_lncRNA | 1.050382 | 0.171244 | 0.453451 | 0.05653 |
| LNC_004028 | XLOC_087018 | -- | intronic_lncRNA | 0.706223 | 0.558648 | 0.530637 | 0.488079 |
| LNC_004029 | XLOC_087108 | -- | intronic_lncRNA | 0.391724 | 0.526706 | 0.689768 | 0.946395 |

| LNC_005489 | XLOC_126848 | -- | lincRNA | 2.263602 | 2.365186 | 1.886621 | 2.932325 |
| --- | --- | --- | --- | --- | --- | --- | --- |
| LNC_005488 | XLOC_126791 | -- | lincRNA | 0 | 0 | 0.523703 | 0.153354 |
| LNC_005935 | XLOC_140243 | -- | lincRNA | 0.520678 | 0.109876 | 0.175053 | 0.178806 |
| LNC_005481 | XLOC_126474 | -- | lincRNA | 0.024275 | 0.518577 | 0 | 0 |
| LNC_005480 | XLOC_126392 | -- | lincRNA | 0.712018 | 0.338425 | 0.171233 | 0.449408 |
| LNC_005483 | XLOC_126628 | -- | intronic_lncRNA | 0.454681 | 0.080915 | 0.306313 | 0.262475 |
| LNC_005482 | XLOC_126628 | -- | intronic_lncRNA | 0.979018 | 1.085462 | 1.725783 | 1.619542 |
| LNC_005485 | XLOC_126735 | -- | lincRNA | 0.920529 | 1.255084 | 0.363023 | 0.73631 |
| LNC_005484 | XLOC_126644 | -- | intronic_lncRNA | 0.916505 | 0.546914 | 2.66873 | 1.791267 |
| LNC_005487 | XLOC_126780 | -- | intronic_lncRNA | 0.182232 | 0.111397 | 0.681198 | 0.098044 |
| LNC_005486 | XLOC_126776 | -- | lincRNA | 0.403643 | 0.366333 | 0.31499 | 0.679757 |
| LNC_000400 | XLOC_007035 | -- | intronic_lncRNA | 0.576503 | 0 | 1.11922 | 0.229198 |
| LNC_006350 | XLOC_152275 | -- | intronic_lncRNA | 0.131139 | 0.87236 | 0.108358 | 0 |
| LNC_002141 | XLOC_048797 | -- | intronic_lncRNA | 0.494701 | 0.155666 | 1.267946 | 0.609541 |
| LNC_002140 | XLOC_048796 | -- | intronic_lncRNA | 0.600158 | 0 | 0 | 0 |
| LNC_002143 | XLOC_048857 | -- | intronic_lncRNA | 0.676776 | 0.999478 | 1.326416 | 0.470919 |
| LNC_002142 | XLOC_048798 | -- | intronic_lncRNA | 0.195943 | 0.283154 | 0.793104 | 0.720512 |
| LNC_002145 | XLOC_048894 | -- | intronic_lncRNA | 0.349001 | 0.353189 | 0.09273 | 0.254578 |
| LNC_002144 | XLOC_048874 | -- | intronic_lncRNA | 0.156654 | 0.207251 | 0.420325 | 0.1674 |
| LNC_002147 | XLOC_048894 | -- | intronic_lncRNA | 1.69261 | 1.678949 | 3.641475 | 3.138871 |
| LNC_002146 | XLOC_048894 | -- | intronic_lncRNA | 0.211283 | 0.915152 | 0.245154 | 0.281752 |
| LNC_002149 | XLOC_048928 | -- | intronic_lncRNA | 0 | 1.585315 | 0.092638 | 0 |
| LNC_002148 | XLOC_048895 | -- | intronic_lncRNA | 0.689463 | 0 | 0 | 0 |
| LNC_003551 | XLOC_075281 | -- | lincRNA | 3.066394 | 1.652727 | 0.822499 | 0.738197 |
| LNC_003550 | XLOC_075281 | -- | lincRNA | 1.426606 | 1.138535 | 0.181228 | 0.34928 |
| LNC_003553 | XLOC_075283 | -- | lincRNA | 1.704142 | 0.252297 | 0 | 0 |
| LNC_003552 | XLOC_075281 | -- | lincRNA | 0.649608 | 0 | 0 | 0 |
| LNC_003555 | XLOC_075289 | -- | lincRNA | 6.567712 | 3.432692 | 1.573144 | 0 |
| LNC_003554 | XLOC_075284 | -- | lincRNA | 1.438418 | 0.473362 | 0.119388 | 0.207717 |
| LNC_003557 | XLOC_075388 | -- | intronic_lncRNA | 0.41928 | 0.419272 | 0.276168 | 0 |
| LNC_003556 | XLOC_075289 | -- | lincRNA | 1.252647 | 0.374981 | 0.188802 | 1.599434 |
| LNC_003559 | XLOC_075420 | -- | intronic_lncRNA | 1.425669 | 0.19677 | 0.43877 | 1.111895 |
| LNC_003558 | XLOC_075389 | -- | intronic_lncRNA | 0 | 0.4447 | 0.061015 | 0.372231 |
| LNC_006351 | XLOC_152275 | -- | intronic_lncRNA | 0.465954 | 0.061174 | 0.122961 | 0.409664 |
| LNC_005733 | XLOC_134477 | -- | intronic_lncRNA | 0.387466 | 0.33453 | 0.625612 | 0.570735 |
| LNC_005732 | XLOC_134449 | -- | lincRNA | 0.412755 | 0 | 0.250884 | 0.376247 |
| LNC_005731 | XLOC_134413 | -- | intronic_lncRNA | 0 | 0 | 0.299258 | 0.649297 |
| LNC_005730 | XLOC_134413 | -- | intronic_lncRNA | 0.766568 | 0.12602 | 0.068003 | 0.030607 |
| LNC_005737 | XLOC_134586 | -- | intronic_lncRNA | 0 | 0 | 0 | 0.433428 |
| LNC_005736 | XLOC_134586 | -- | intronic_lncRNA | 0.372274 | 0.345125 | 0.225962 | 0.555424 |
| LNC_005735 | XLOC_134553 | -- | intronic_lncRNA | 0.998075 | 0.701311 | 1.067077 | 1.566838 |
| LNC_005734 | XLOC_134524 | -- | intronic_lncRNA | 0.385412 | 0 | 0.624888 | 0 |
| LNC_005739 | XLOC_134588 | -- | intronic_lncRNA | 0.591272 | 0.457869 | 0.957408 | 1.615347 |
| LNC_005738 | XLOC_134587 | -- | intronic_lncRNA | 0.587837 | 0.081205 | 0.539298 | 0.520838 |
| LNC_006352 | XLOC_152277 | -- | intronic_lncRNA | 0.636761 | 0.144925 | 0.231577 | 0.114378 |
| LNC_003089 | XLOC_070734 | -- | intronic_lncRNA | 1.140902 | 0.443843 | 0.432409 | 0.403903 |
| LNC_003088 | XLOC_070690 | -- | intronic_lncRNA | 0.799919 | 0 | 0.323548 | 0.568107 |
| LNC_003083 | XLOC_070652 | -- | intronic_lncRNA | 0.134024 | 0.712959 | 0 | 0 |
| LNC_003082 | XLOC_070648 | -- | intronic_lncRNA | 0 | 0 | 0.415675 | 0 |
| LNC_003081 | XLOC_070643 | -- | intronic_lncRNA | 0.283589 | 0 | 0.075285 | 0.421087 |
| LNC_003080 | XLOC_070642 | -- | intronic_lncRNA | 0.251281 | 0 | 0.338184 | 0.201883 |
| LNC_003087 | XLOC_070677 | -- | intronic_lncRNA | 0.155256 | 0.605474 | 0 | 0 |
| LNC_003086 | XLOC_070669 | -- | intronic_lncRNA | 1.210469 | 0 | 0.108535 | 0 |
| LNC_003085 | XLOC_070654 | -- | intronic_lncRNA | 0.319406 | 0.254693 | 0.441045 | 0 |
| LNC_003084 | XLOC_070653 | -- | intronic_lncRNA | 0 | 0.721983 | 0.191087 | 0 |
| LNC_006019 | XLOC_143367 | -- | intronic_lncRNA | 0.257994 | 0.975252 | 0.569551 | 0.946224 |
| LNC_006018 | XLOC_143366 | -- | intronic_lncRNA | 0.648079 | 0.627861 | 0.683504 | 0.730589 |
| LNC_001496 | XLOC_032567 | -- | intronic_lncRNA | 1.672644 | 1.225462 | 2.533997 | 3.178491 |
| LNC_001497 | XLOC_032586 | -- | intronic_lncRNA | 0.57547 | 0.577462 | 0.587483 | 0.515492 |
| LNC_001494 | XLOC_032563 | -- | intronic_lncRNA | 0.102799 | 0.138932 | 0.914303 | 0.09218 |
| LNC_001495 | XLOC_032563 | -- | intronic_lncRNA | 0.697501 | 0.129077 | 1.175388 | 1.365379 |
| LNC_001492 | XLOC_032504 | -- | intronic_lncRNA | 0.411035 | 0.166562 | 0.387443 | 0.97048 |
| LNC_001493 | XLOC_032512 | -- | intronic_lncRNA | 0.56993 | 0.897231 | 1.285868 | 0.643098 |
| LNC_001490 | XLOC_032430 | -- | intronic_lncRNA | 11.174572 | 5.292788 | 5.618469 | 4.101561 |
| LNC_001491 | XLOC_032499 | -- | intronic_lncRNA | 0 | 0 | 0 | 0.721717 |
| LNC_001498 | XLOC_032601 | -- | intronic_lncRNA | 11.733192 | 13.389799 | 13.833227 | 11.985251 |
| LNC_001499 | XLOC_032603 | -- | intronic_lncRNA | 0 | 0 | 0.494435 | 0 |
| LNC_005869 | XLOC_138055 | -- | intronic_lncRNA | 0.247552 | 0.248404 | 0.501739 | 0.26685 |
| LNC_005868 | XLOC_138052 | -- | intronic_lncRNA | 0 | 0.504933 | 0.049348 | 0.32463 |
| LNC_005863 | XLOC_137856 | -- | intronic_lncRNA | 0.593174 | 0.766353 | 0.307396 | 0.254008 |
| LNC_005862 | XLOC_137855 | -- | intronic_lncRNA | 0.468263 | 0.373086 | 0.197088 | 0.584546 |
| LNC_005861 | XLOC_137846 | -- | intronic_lncRNA | 0.247462 | 0 | 0.932282 | 0.225989 |
| LNC_005860 | XLOC_137846 | -- | intronic_lncRNA | 0.394669 | 0.354131 | 0.597862 | 0.956586 |
| LNC_005867 | XLOC_137972 | -- | intronic_lncRNA | 0 | 0.197277 | 0.223673 | 0.798693 |
| LNC_005866 | XLOC_137947 | -- | intronic_lncRNA | 0.627294 | 0.221442 | 0 | 0.557426 |
| LNC_005865 | XLOC_137933 | -- | intronic_lncRNA | 0.386032 | 0.63851 | 0.948454 | 0.692228 |
| LNC_005864 | XLOC_137870 | -- | intronic_lncRNA | 0.780381 | 0.63496 | 0.871271 | 1.22829 |

| LNC_006356 | XLOC_152323 | -- | intronic_lncRNA | 1.135429 | 0 | 0.175301 | 0.218576 |
| --- | --- | --- | --- | --- | --- | --- | --- |
| LNC_001728 | XLOC_038055 | -- | intronic_lncRNA | 0 | 0.539529 | 0 | 0.211152 |
| LNC_001729 | XLOC_038130 | -- | intronic_lncRNA | 0.247845 | 0 | 0.429347 | 0.244192 |
| LNC_006118 | XLOC_146521 | -- | lincRNA | 0.496834 | 0.065873 | 0 | 0 |
| LNC_006119 | XLOC_146559 | -- | intronic_lncRNA | 0.777654 | 0.415759 | 0.283817 | 0.449737 |
| LNC_001720 | XLOC_037828 | -- | intronic_lncRNA | 0.45143 | 0.061504 | 0.136013 | 0.319316 |
| LNC_001721 | XLOC_037832 | -- | intronic_lncRNA | 0.198993 | 0 | 0.49403 | 0 |
| LNC_001722 | XLOC_037834 | -- | intronic_lncRNA | 0.315292 | 0.143225 | 0.513492 | 0.405798 |
| LNC_001723 | XLOC_037875 | -- | lincRNA | 0.957111 | 0.739919 | 1.1024 | 0.747177 |
| LNC_001724 | XLOC_038021 | -- | intronic_lncRNA | 0.241671 | 0.520277 | 0.249667 | 0.55284 |
| LNC_001725 | XLOC_038038 | -- | intronic_lncRNA | 0.379054 | 0.867196 | 0.497769 | 0.12965 |
| LNC_001726 | XLOC_038038 | -- | intronic_lncRNA | 0.80468 | 0.555645 | 0.575032 | 1.149324 |
| LNC_001727 | XLOC_038054 | -- | intronic_lncRNA | 0 | 0.402798 | 0.181496 | 0.470842 |
| LNC_006358 | XLOC_152378 | -- | intronic_lncRNA | 2.258421 | 1.519389 | 2.557425 | 3.235668 |
| LNC_000409 | XLOC_007098 | -- | intronic_lncRNA | 0.653611 | 0.854953 | 0.11621 | 0.468936 |
| LNC_006359 | XLOC_152406 | -- | antisense_lncRNA | 0.331666 | 0.699754 | 0.462164 | 0.480186 |
| LNC_006378 | XLOC_152797 | -- | intronic_lncRNA | 0.230239 | 0.105641 | 0.37357 | 0.056008 |
| LNC_006379 | XLOC_153066 | -- | lincRNA | 0 | 0 | 0 | 0.455953 |
| LNC_006376 | XLOC_152788 | -- | intronic_lncRNA | 0.439179 | 0.412595 | 0.370829 | 0.373121 |
| LNC_006377 | XLOC_152797 | -- | intronic_lncRNA | 0.164654 | 0.626152 | 0.000659 | 0.020854 |
| LNC_006374 | XLOC_152677 | -- | intronic_lncRNA | 0.471411 | 0.46951 | 0.344101 | 0.45518 |
| LNC_006375 | XLOC_152698 | -- | intronic_lncRNA | 2.152258 | 1.824647 | 2.784296 | 2.032725 |
| LNC_006372 | XLOC_152619 | -- | intronic_lncRNA | 0.569499 | 0.47271 | 0.827411 | 0.853919 |
| LNC_006373 | XLOC_152668 | -- | intronic_lncRNA | 0.11223 | 0.717813 | 0 | 0 |
| LNC_006370 | XLOC_152559 | -- | intronic_lncRNA | 0.652474 | 0.007224 | 0.234905 | 0.373354 |
| LNC_006371 | XLOC_152573 | -- | lincRNA | 0.526741 | 0 | 0 | 0 |
| LNC_000459 | XLOC_008146 | -- | intronic_lncRNA | 0.277541 | 0.295038 | 0.509719 | 0.537266 |
| LNC_005138 | XLOC_117311 | -- | intronic_lncRNA | 0.654613 | 0 | 0 | 0 |
| LNC_005131 | XLOC_117207 | -- | intronic_lncRNA | 2.095902 | 1.220792 | 1.807378 | 1.778509 |
| LNC_005130 | XLOC_117189 | -- | lincRNA | 0 | 0 | 0 | 1.057017 |
| LNC_000453 | XLOC_007959 | -- | intronic_lncRNA | 0.351513 | 0.204704 | 0.180885 | 0.732441 |
| LNC_000452 | XLOC_007940 | -- | intronic_lncRNA | 0.34034 | 0.451706 | 0.543498 | 0.416267 |
| LNC_005135 | XLOC_117290 | -- | intronic_lncRNA | 0.909747 | 0.668784 | 0.039517 | 0.276457 |
| LNC_005134 | XLOC_117256 | -- | intronic_lncRNA | 0.500666 | 0.377817 | 0.597037 | 0.260565 |
| LNC_000457 | XLOC_008134 | -- | antisense_lncRNA | 0 | 0 | 0.846329 | 0 |
| LNC_000456 | XLOC_008038 | -- | intronic_lncRNA | 0.133187 | 0 | 0.364175 | 0.623746 |
| LNC_005254 | XLOC_120519 | -- | antisense_lncRNA | 0.627261 | 0.358196 | 0.262503 | 0.184109 |
| LNC_005255 | XLOC_120529 | -- | intronic_lncRNA | 0.401006 | 0.379044 | 0.209731 | 0.811363 |
| LNC_005256 | XLOC_120529 | -- | intronic_lncRNA | 0.391878 | 1.516784 | 1.077347 | 1.203188 |
| LNC_005257 | XLOC_120575 | -- | lincRNA | 0.551997 | 0.598703 | 0 | 0 |
| LNC_005250 | XLOC_120481 | -- | intronic_lncRNA | 0.268753 | 0.536973 | 0.279385 | 0.667597 |
| LNC_005251 | XLOC_120483 | -- | intronic_lncRNA | 0.014813 | 0.013507 | 1.305362 | 0.002761 |
| LNC_005252 | XLOC_120483 | -- | intronic_lncRNA | 0.89442 | 0.902302 | 0.41553 | 0.971645 |
| LNC_005253 | XLOC_120507 | -- | intronic_lncRNA | 0.202054 | 0.507653 | 0.411127 | 0.222165 |
| LNC_005258 | XLOC_120657 | -- | lincRNA | 0 | 1.09957 | 0 | 0 |
| LNC_005259 | XLOC_120663 | -- | intronic_lncRNA | 0.671348 | 0.185895 | 0.296581 | 0.445548 |
| LNC_002916 | XLOC_067187 | -- | intronic_lncRNA | 2.021163 | 0.825553 | 1.117332 | 0.473962 |
| LNC_002917 | XLOC_067187 | -- | intronic_lncRNA | 0.003026 | 0.625721 | 0.003459 | 0.000986 |
| LNC_002914 | XLOC_067187 | -- | intronic_lncRNA | 1.346162 | 0.621029 | 1.524955 | 1.1777 |
| LNC_002915 | XLOC_067187 | -- | intronic_lncRNA | 0 | 0 | 0 | 1.074699 |
| LNC_002912 | XLOC_067174 | -- | intronic_lncRNA | 0.610497 | 0.371982 | 0.137182 | 0.221844 |
| LNC_002913 | XLOC_067176 | -- | intronic_lncRNA | 0 | 0.120081 | 0.464202 | 0.528295 |
| LNC_002910 | XLOC_067173 | -- | intronic_lncRNA | 0 | 0.085168 | 0.545558 | 0.061223 |
| LNC_002911 | XLOC_067174 | -- | intronic_lncRNA | 0 | 0.06522 | 0.622322 | 0.630689 |
| LNC_002918 | XLOC_067200 | -- | lincRNA | 0.564173 | 0.874988 | 0.05888 | 0.135702 |
| LNC_002919 | XLOC_067216 | -- | intronic_lncRNA | 0.847688 | 0 | 0 | 0.379356 |
| LNC_001935 | XLOC_043577 | -- | intronic_lncRNA | 0.58651 | 0 | 0.31595 | 0.300452 |
| LNC_001934 | XLOC_043547 | -- | intronic_lncRNA | 0 | 0 | 1.182824 | 0.382751 |
| LNC_001937 | XLOC_043663 | -- | intronic_lncRNA | 0.188782 | 0.373555 | 0.318449 | 0 |
| LNC_001936 | XLOC_043611 | -- | intronic_lncRNA | 0.635296 | 0.409024 | 0.385723 | 0.380382 |
| LNC_001931 | XLOC_043514 | -- | antisense_lncRNA | 0.041941 | 0 | 0.366462 | 0.512928 |
| LNC_001930 | XLOC_043459 | -- | intronic_lncRNA | 0.384652 | 0.034543 | 0.174485 | 0.732477 |
| LNC_001933 | XLOC_043543 | -- | intronic_lncRNA | 0.699304 | 0 | 0.438811 | 0.535839 |
| LNC_001932 | XLOC_043516 | -- | antisense_lncRNA | 0.561419 | 0.312903 | 0.230675 | 0.20155 |
| LNC_004980 | XLOC_113429 | -- | intronic_lncRNA | 0 | 0 | 0.587244 | 0.157468 |
| LNC_001939 | XLOC_043666 | -- | intronic_lncRNA | 0 | 0 | 0 | 0.747415 |
| LNC_001938 | XLOC_043666 | -- | intronic_lncRNA | 0.578571 | 0.859249 | 0.602611 | 0.742879 |
| LNC_000048 | XLOC_000406 | -- | lincRNA | 2.902363 | 4.428241 | 0.390727 | 0.790885 |
| LNC_000049 | XLOC_000431 | -- | lincRNA | 0.462386 | 0.534332 | 0.134016 | 0.460323 |
| LNC_000046 | XLOC_000406 | -- | lincRNA | 2.966847 | 3.942261 | 1.081263 | 0.649099 |
| LNC_000047 | XLOC_000406 | -- | lincRNA | 0 | 4.625154 | 0 | 0 |
| LNC_000044 | XLOC_000402 | -- | lincRNA | 0.590344 | 0 | 0 | 0 |
| LNC_000045 | XLOC_000404 | -- | lincRNA | 1.282845 | 3.763779 | 0.256616 | 0.203807 |
| LNC_000042 | XLOC_000396 | -- | lincRNA | 0.997606 | 0.732622 | 0.426288 | 1.121191 |
| LNC_000043 | XLOC_000401 | -- | lincRNA | 0 | 0.915649 | 0 | 0 |
| LNC_000040 | XLOC_000396 | -- | lincRNA | 2.334558 | 1.276735 | 0.436307 | 0.815326 |
| LNC_000041 | XLOC_000396 | -- | lincRNA | 26.238489 | 15.199239 | 13.257084 | 16.546635 |

| LNC_004619 | XLOC_102795 | -- | intronic_lncRNA | 0.760157 | 0.234815 | 0.095083 | 0.84483 |
| --- | --- | --- | --- | --- | --- | --- | --- |
| LNC_004618 | XLOC_102791 | -- | intronic_lncRNA | 0.10666 | 0.219936 | 0.387836 | 0.727283 |
| LNC_004617 | XLOC_102754 | -- | intronic_lncRNA | 1.134912 | 0.552893 | 0.516168 | 0.367448 |
| LNC_004616 | XLOC_102743 | -- | intronic_lncRNA | 0 | 0 | 0.2487 | 0.324718 |
| LNC_004615 | XLOC_102743 | -- | intronic_lncRNA | 0.524747 | 0.005808 | 0.775417 | 0.27149 |
| LNC_004614 | XLOC_102743 | -- | antisense_lncRNA | 0 | 0.335649 | 0.191503 | 0.844854 |
| LNC_004613 | XLOC_102737 | -- | intronic_lncRNA | 1.383971 | 0.422763 | 0.402776 | 0.461555 |
| LNC_004612 | XLOC_102736 | -- | intronic_lncRNA | 1.077175 | 0.513643 | 1.157914 | 0.671564 |
| LNC_004611 | XLOC_102702 | -- | intronic_lncRNA | 0.539347 | 0 | 0.554939 | 0 |
| LNC_004610 | XLOC_102667 | -- | lincRNA | 0.062277 | 0 | 0.615927 | 0.554686 |
| LNC_001557 | XLOC_034021 | -- | antisense_lncRNA | 1.374021 | 0.193273 | 0 | 0.042246 |
| LNC_001556 | XLOC_034015 | -- | lincRNA | 3.265725 | 0.705588 | 0 | 0 |
| LNC_001555 | XLOC_033981 | -- | intronic_lncRNA | 0.089293 | 0 | 0.432915 | 0.432359 |
| LNC_001554 | XLOC_033974 | -- | intronic_lncRNA | 0 | 1.127229 | 0.022172 | 0 |
| LNC_001553 | XLOC_033974 | -- | intronic_lncRNA | 1.101092 | 0.395726 | 1.124917 | 1.362603 |
| LNC_001552 | XLOC_033970 | -- | intronic_lncRNA | 0.476918 | 0.506277 | 0.792992 | 0.723835 |
| LNC_001551 | XLOC_033932 | -- | intronic_lncRNA | 0.533299 | 0 | 0 | 0 |
| LNC_001550 | XLOC_033929 | -- | intronic_lncRNA | 1.619339 | 0.612158 | 1.027805 | 1.634536 |
| LNC_001559 | XLOC_034021 | -- | intronic_lncRNA | 1.611338 | 0.634011 | 0.105056 | 0 |
| LNC_001558 | XLOC_034021 | -- | intronic_lncRNA | 2.463681 | 0.331416 | 0.318215 | 0.141739 |
| LNC_002630 | XLOC_060905 | -- | intronic_lncRNA | 0.174777 | 0.041577 | 0.652847 | 0.08761 |
| LNC_002358 | XLOC_054613 | -- | intronic_lncRNA | 0.447035 | 0.241162 | 0.426371 | 0.374839 |
| LNC_002359 | XLOC_054614 | -- | intronic_lncRNA | 0.159259 | 0.703937 | 0.386065 | 0.058456 |
| LNC_002354 | XLOC_054389 | -- | intronic_lncRNA | 0.643056 | 0.421001 | 0.253333 | 0.417831 |
| LNC_002355 | XLOC_054390 | -- | intronic_lncRNA | 0.275645 | 0.608917 | 0.097369 | 0.19418 |
| LNC_002356 | XLOC_054406 | -- | intronic_lncRNA | 0.868902 | 0.937291 | 0.251216 | 0.287077 |
| LNC_002357 | XLOC_054601 | -- | intronic_lncRNA | 0.057941 | 0.523696 | 0.476781 | 0.128981 |
| LNC_002350 | XLOC_054168 | -- | antisense_lncRNA | 1.105291 | 0.497947 | 0.152761 | 0.096435 |
| LNC_002351 | XLOC_054225 | -- | lincRNA | 0 | 0.172105 | 0.505487 | 0.523052 |
| LNC_002352 | XLOC_054288 | -- | intronic_lncRNA | 0 | 0.205671 | 0 | 0.41412 |
| LNC_002353 | XLOC_054342 | -- | lincRNA | 0.191216 | 0.126571 | 0.772296 | 1.079072 |
| LNC_003348 | XLOC_075175 | -- | lincRNA | 0.526048 | 1.151418 | 0.075614 | 0 |
| LNC_003349 | XLOC_075175 | -- | lincRNA | 2.410982 | 1.369573 | 0.459057 | 0.23161 |
| LNC_003344 | XLOC_075175 | -- | lincRNA | 1.494429 | 0.451547 | 0.095627 | 0.272213 |
| LNC_003345 | XLOC_075175 | -- | lincRNA | 1.940027 | 1.661039 | 0.182896 | 1.198143 |
| LNC_003346 | XLOC_075175 | -- | lincRNA | 1.794491 | 0 | 0 | 0 |
| LNC_003347 | XLOC_075175 | -- | lincRNA | 2.202751 | 0.400325 | 0.430374 | 0.176854 |
| LNC_003340 | XLOC_075175 | -- | lincRNA | 1.363943 | 0.573618 | 0 | 0.293551 |
| LNC_003341 | XLOC_075175 | -- | lincRNA | 0.755557 | 0.761906 | 1.636195 | 1.518633 |
| LNC_003342 | XLOC_075175 | -- | lincRNA | 1.785613 | 0.255649 | 0.336169 | 0.227654 |
| LNC_003343 | XLOC_075175 | -- | lincRNA | 1.891154 | 1.539321 | 0.539594 | 0.969249 |
| LNC_000604 | XLOC_011504 | -- | intronic_lncRNA | 0.977603 | 0 | 0.044468 | 0.233549 |
| LNC_000605 | XLOC_011572 | -- | intronic_lncRNA | 174.798584 | 316.312378 | 1.921344 | 2.235099 |
| LNC_000606 | XLOC_011617 | -- | intronic_lncRNA | 0 | 0 | 0.044848 | 0.52818 |
| LNC_000607 | XLOC_011617 | -- | intronic_lncRNA | 0.583068 | 0.530685 | 0.228444 | 0 |
| LNC_000600 | XLOC_011415 | -- | intronic_lncRNA | 1.174771 | 0.69232 | 1.279464 | 1.294548 |
| LNC_000601 | XLOC_011455 | -- | lincRNA | 0.393333 | 0 | 0.04118 | 0.496468 |
| LNC_000602 | XLOC_011478 | -- | intronic_lncRNA | 1.03323 | 0.343101 | 1.223703 | 1.225881 |
| LNC_000603 | XLOC_011504 | -- | intronic_lncRNA | 1.002872 | 0.353558 | 0.666798 | 0.55767 |
| LNC_000608 | XLOC_011625 | -- | antisense_lncRNA | 1.213226 | 0.816408 | 1.300566 | 0.982118 |
| LNC_000609 | XLOC_011637 | -- | intronic_lncRNA | 0.242967 | 0 | 1.355865 | 0.412471 |
| LNC_002769 | XLOC_064110 | -- | intronic_lncRNA | 0 | 0 | 0.334462 | 0.399686 |
| LNC_002768 | XLOC_064108 | -- | intronic_lncRNA | 0.351599 | 0.239008 | 0.412934 | 0.203636 |
| LNC_002765 | XLOC_064063 | -- | intronic_lncRNA | 0 | 0.394553 | 0.874454 | 0.559085 |
| LNC_002764 | XLOC_064048 | -- | intronic_lncRNA | 0 | 0.511007 | 0 | 0 |
| LNC_002767 | XLOC_064106 | -- | intronic_lncRNA | 0 | 0 | 0.113457 | 0.453751 |
| LNC_002766 | XLOC_064073 | -- | intronic_lncRNA | 0 | 0 | 0.792764 | 0.579279 |
| LNC_002761 | XLOC_064022 | -- | intronic_lncRNA | 0.610276 | 0.404775 | 0.464619 | 1.246477 |
| LNC_002760 | XLOC_064006 | -- | intronic_lncRNA | 0.60954 | 0.583925 | 1.730241 | 0.633605 |
| LNC_002763 | XLOC_064047 | -- | antisense_lncRNA | 0.872414 | 0.036975 | 0.65131 | 0.120302 |
| LNC_002762 | XLOC_064036 | -- | intronic_lncRNA | 0.55684 | 0.227743 | 0 | 0 |
| LNC_001232 | XLOC_026659 | -- | intronic_lncRNA | 0.607241 | 0 | 0.037952 | 0.496637 |
| LNC_001233 | XLOC_026659 | -- | intronic_lncRNA | 0.105957 | 0.074935 | 0.599411 | 0.162845 |
| LNC_001230 | XLOC_026623 | -- | intronic_lncRNA | 0.559287 | 0 | 0.578859 | 0.942053 |
| LNC_001231 | XLOC_026624 | -- | intronic_lncRNA | 1.239246 | 0.905666 | 1.146272 | 0.95225 |
| LNC_001236 | XLOC_026767 | -- | intronic_lncRNA | 0.371008 | 0.109376 | 0.7234 | 0.430571 |
| LNC_001237 | XLOC_026786 | -- | antisense_lncRNA | 0.626943 | 2.422215 | 2.8179 | 3.40483 |
| LNC_001234 | XLOC_026699 | -- | intronic_lncRNA | 0 | 0 | 0 | 0.55035 |
| LNC_001235 | XLOC_026760 | -- | intronic_lncRNA | 0.437083 | 0.41256 | 0.193583 | 0.470361 |
| LNC_001238 | XLOC_026793 | -- | intronic_lncRNA | 0.437846 | 0.213625 | 0.57986 | 0.865604 |
| LNC_001239 | XLOC_026817 | -- | intronic_lncRNA | 0 | 0.510258 | 0.068873 | 0 |
| LNC_003154 | XLOC_072770 | -- | intronic_lncRNA | 0.388872 | 0.446332 | 0.573127 | 0.586557 |
| LNC_003988 | XLOC_086008 | -- | intronic_lncRNA | 0 | 0 | 0.828062 | 0.333022 |
| LNC_003989 | XLOC_086042 | -- | intronic_lncRNA | 0.534812 | 0.436403 | 0.454518 | 0.518332 |
| LNC_003982 | XLOC_085884 | -- | intronic_lncRNA | 0.11896 | 0.123666 | 0.540175 | 0.79073 |
| LNC_003983 | XLOC_085885 | -- | intronic_lncRNA | 0 | 0 | 0.268874 | 0.534388 |
| LNC_003980 | XLOC_085871 | -- | intronic_lncRNA | 3.212681 | 5.063691 | 6.251206 | 5.498932 |

| LNC_003981 | XLOC_085871 | -- | intronic_lncRNA | 0 | 0 | 0.005296 | 1.135514 |
| --- | --- | --- | --- | --- | --- | --- | --- |
| LNC_003986 | XLOC_085984 | -- | intronic_lncRNA | 0.125765 | 0.685031 | 0 | 0.114356 |
| LNC_003987 | XLOC_086008 | -- | intronic_lncRNA | 1.654743 | 2.174436 | 1.293485 | 1.570562 |
| LNC_003984 | XLOC_085927 | -- | intronic_lncRNA | 0.609751 | 0 | 0.164235 | 0 |
| LNC_003985 | XLOC_085937 | -- | intronic_lncRNA | 0.738936 | 0 | 0 | 0 |
| LNC_000183 | XLOC_002328 | -- | intronic_lncRNA | 0.848162 | 0.128247 | 0.100654 | 0 |
| LNC_000182 | XLOC_002327 | -- | intronic_lncRNA | 0.484414 | 0 | 0 | 0 |
| LNC_000181 | XLOC_002325 | -- | intronic_lncRNA | 0.389859 | 1.214454 | 0.753375 | 0.40304 |
| LNC_000180 | XLOC_002324 | -- | intronic_lncRNA | 0.566821 | 0.355577 | 0.796357 | 0.967824 |
| LNC_000187 | XLOC_002409 | -- | intronic_lncRNA | 0.127477 | 0.10724 | 0.412029 | 0 |
| LNC_000186 | XLOC_002402 | -- | intronic_lncRNA | 0 | 0.076558 | 0.211232 | 0.416833 |
| LNC_000185 | XLOC_002332 | -- | intronic_lncRNA | 0.97759 | 0.535304 | 0 | 0.427152 |
| LNC_000184 | XLOC_002331 | -- | intronic_lncRNA | 1.045466 | 0 | 0.070106 | 0.106076 |
| LNC_000189 | XLOC_002459 | -- | intronic_lncRNA | 0.191496 | 0.403363 | 0.483467 | 0.409467 |
| LNC_000188 | XLOC_002457 | -- | intronic_lncRNA | 0.697556 | 0 | 0.306242 | 0.477592 |
| LNC_002169 | XLOC_049480 | -- | intronic_lncRNA | 0.032153 | 0.103589 | 0.927119 | 0.594047 |
| LNC_002168 | XLOC_049479 | -- | intronic_lncRNA | 0.444685 | 0.211783 | 0.645808 | 0.521731 |
| LNC_002163 | XLOC_049311 | -- | intronic_lncRNA | 0.26444 | 0 | 0.353593 | 0.638785 |
| LNC_002162 | XLOC_049029 | -- | intronic_lncRNA | 0.359443 | 0.131369 | 0.744022 | 0.89933 |
| LNC_002161 | XLOC_048959 | -- | intronic_lncRNA | 0.77094 | 0.399192 | 0.703012 | 0.880226 |
| LNC_002160 | XLOC_048958 | -- | intronic_lncRNA | 0.466763 | 0.992485 | 0.666536 | 1.014683 |
| LNC_002167 | XLOC_049457 | -- | intronic_lncRNA | 0.142133 | 0 | 0.42096 | 0 |
| LNC_002166 | XLOC_049443 | -- | intronic_lncRNA | 0.547711 | 0.484936 | 0.474794 | 0.63375 |
| LNC_002165 | XLOC_049395 | -- | lincRNA | 0.132739 | 0 | 0.123876 | 0.563431 |
| LNC_002164 | XLOC_049317 | -- | antisense_lncRNA | 0.393298 | 1.148479 | 0.794038 | 0 |
| LNC_003579 | XLOC_075755 | -- | intronic_lncRNA | 1.071514 | 0.004455 | 0 | 0 |
| LNC_003578 | XLOC_075754 | -- | intronic_lncRNA | 1.014719 | 0.27626 | 0.362173 | 0.524422 |
| LNC_003573 | XLOC_075571 | -- | intronic_lncRNA | 0.475365 | 0.527719 | 0.892835 | 1.044746 |
| LNC_003572 | XLOC_075570 | -- | intronic_lncRNA | 0.282594 | 0.2783 | 1.486031 | 0.855421 |
| LNC_003571 | XLOC_075562 | -- | intronic_lncRNA | 0.669267 | 0.551883 | 0 | 0 |
| LNC_003570 | XLOC_075561 | -- | intronic_lncRNA | 0.26024 | 0.740162 | 0 | 0 |
| LNC_003577 | XLOC_075753 | -- | intronic_lncRNA | 1.654608 | 1.371666 | 1.036161 | 1.128007 |
| LNC_003576 | XLOC_075753 | -- | intronic_lncRNA | 0.254142 | 0.274552 | 0.134781 | 0.397686 |
| LNC_003575 | XLOC_075743 | -- | lincRNA | 0 | 0.311224 | 0.716381 | 0.557024 |
| LNC_003574 | XLOC_075608 | -- | lincRNA | 0.888631 | 0 | 0 | 0 |
| LNC_000989 | XLOC_021068 | -- | intronic_lncRNA | 0.235003 | 0 | 0.322471 | 0.757315 |
| LNC_000988 | XLOC_021064 | -- | intronic_lncRNA | 1.380486 | 0.264224 | 1.347658 | 1.028358 |
| LNC_000983 | XLOC_021003 | -- | intronic_lncRNA | 0.705347 | 0.504912 | 0.878993 | 1.1905 |
| LNC_000982 | XLOC_021002 | -- | intronic_lncRNA | 0.511769 | 0.284832 | 0.180903 | 0 |
| LNC_000981 | XLOC_021000 | -- | intronic_lncRNA | 0.149433 | 0.239055 | 0.664118 | 0.140332 |
| LNC_000980 | XLOC_020999 | -- | intronic_lncRNA | 0 | 0 | 0 | 0.396314 |
| LNC_000987 | XLOC_021046 | -- | intronic_lncRNA | 0.855308 | 0 | 0 | 0 |
| LNC_000986 | XLOC_021019 | -- | intronic_lncRNA | 0.793906 | 1.2582 | 1.125913 | 0.865828 |
| LNC_000985 | XLOC_021015 | -- | intronic_lncRNA | 0 | 0.295162 | 0.358559 | 0.101176 |
| LNC_000984 | XLOC_021003 | -- | intronic_lncRNA | 0.204462 | 0 | 0.585441 | 0 |
| LNC_005755 | XLOC_135012 | -- | intronic_lncRNA | 0 | 0 | 12.927636 | 0.053728 |
| LNC_005754 | XLOC_135007 | -- | lincRNA | 0.726367 | 0 | 0 | 0.062725 |
| LNC_005757 | XLOC_135024 | -- | intronic_lncRNA | 0 | 1.072583 | 0 | 0.006127 |
| LNC_005756 | XLOC_135024 | -- | intronic_lncRNA | 0.336579 | 0 | 0.221602 | 0.471191 |
| LNC_005751 | XLOC_134884 | -- | intronic_lncRNA | 0.49653 | 0.075246 | 0.379768 | 0.398712 |
| LNC_005750 | XLOC_134883 | -- | intronic_lncRNA | 0.770807 | 0.42622 | 0.249405 | 0.609274 |
| LNC_005753 | XLOC_134890 | -- | intronic_lncRNA | 0.203964 | 0 | 0.594969 | 0.411958 |
| LNC_005752 | XLOC_134885 | -- | intronic_lncRNA | 0.42178 | 0 | 0.454928 | 0 |
| LNC_005759 | XLOC_135024 | -- | intronic_lncRNA | 1.146751 | 0 | 1.184317 | 0.904425 |
| LNC_005758 | XLOC_135024 | -- | intronic_lncRNA | 0 | 0 | 0 | 0.495649 |
| LNC_002299 | XLOC_052863 | -- | intronic_lncRNA | 0.451079 | 0.484635 | 0.558215 | 0.426106 |
| LNC_002298 | XLOC_052781 | -- | lincRNA | 0.702591 | 0.139819 | 0.498099 | 0.680592 |
| LNC_002295 | XLOC_052675 | -- | intronic_lncRNA | 0 | 0 | 0 | 1.610226 |
| LNC_002294 | XLOC_052675 | -- | intronic_lncRNA | 0.53576 | 0.383964 | 0.840764 | 0.42064 |
| LNC_002297 | XLOC_052690 | -- | antisense_lncRNA | 0.186623 | 1.117523 | 0.61573 | 0.827654 |
| LNC_002296 | XLOC_052686 | -- | intronic_lncRNA | 1.325776 | 0.62442 | 6.606422 | 6.999609 |
| LNC_002291 | XLOC_052665 | -- | intronic_lncRNA | 0.301387 | 0.096661 | 0.414097 | 0.262584 |
| LNC_002290 | XLOC_052661 | -- | intronic_lncRNA | 0.037968 | 0.018189 | 1.902635 | 1.44684 |
| LNC_002293 | XLOC_052673 | -- | intronic_lncRNA | 0.57512 | 0.256754 | 0.361696 | 0.222631 |
| LNC_002292 | XLOC_052665 | -- | intronic_lncRNA | 0.463337 | 0.699414 | 0.81137 | 2.293261 |
| LNC_004408 | XLOC_096315 | -- | intronic_lncRNA | 0 | 0.076469 | 0.724325 | 0.525802 |
| LNC_004409 | XLOC_096315 | -- | intronic_lncRNA | 1.051718 | 0.4558 | 0.352849 | 0.728062 |
| LNC_005809 | XLOC_136827 | -- | intronic_lncRNA | 0.338825 | 0.506041 | 0.255989 | 0.037565 |
| LNC_005808 | XLOC_136811 | -- | intronic_lncRNA | 0.637273 | 0 | 0 | 0 |
| LNC_004402 | XLOC_096131 | -- | intronic_lncRNA | 0.375065 | 0.06318 | 0.613748 | 0.206432 |
| LNC_004403 | XLOC_096258 | -- | lincRNA | 0 | 0.633344 | 0.053168 | 0 |
| LNC_004400 | XLOC_095986 | -- | intronic_lncRNA | 0.281279 | 0.009803 | 0.542787 | 0 |
| LNC_004401 | XLOC_096124 | -- | intronic_lncRNA | 0.359894 | 0.450327 | 0.360745 | 0.348174 |
| LNC_004406 | XLOC_096311 | -- | intronic_lncRNA | 0.739119 | 0.425502 | 0.511217 | 0.433158 |
| LNC_004407 | XLOC_096313 | -- | intronic_lncRNA | 0.017767 | 0.498562 | 0.107075 | 0.596792 |
| LNC_004404 | XLOC_096279 | -- | lincRNA | 0.147196 | 0.594533 | 0.138299 | 0.264664 |
| LNC_004405 | XLOC_096279 | -- | lincRNA | 0.47014 | 0.875313 | 0.524155 | 0.35577 |

| LNC_006138 | XLOC_146928 | -- | intronic_lncRNA | 0.72662 | 0 | 0.244642 | 0.51529 |
| --- | --- | --- | --- | --- | --- | --- | --- |
| LNC_006139 | XLOC_146929 | -- | intronic_lncRNA | 0.053516 | 0 | 0.710906 | 0.329115 |
| LNC_006134 | XLOC_146859 | -- | intronic_lncRNA | 0.263757 | 0.899203 | 0.121642 | 0.150047 |
| LNC_006135 | XLOC_146903 | -- | intronic_lncRNA | 1.067108 | 0.192939 | 0.230636 | 0.266255 |
| LNC_006136 | XLOC_146917 | -- | intronic_lncRNA | 3.154529 | 1.892038 | 6.937016 | 7.522277 |
| LNC_006137 | XLOC_146919 | -- | intronic_lncRNA | 0.319273 | 0.804814 | 0.165788 | 0.348073 |
| LNC_006130 | XLOC_146832 | -- | intronic_lncRNA | 0.933803 | 0.606036 | 0.853786 | 0.89794 |
| LNC_006131 | XLOC_146837 | -- | intronic_lncRNA | 0.515934 | 0.806801 | 0.658649 | 1.210919 |
| LNC_006132 | XLOC_146844 | -- | intronic_lncRNA | 0.095633 | 0.062956 | 0.529816 | 0 |
| LNC_006133 | XLOC_146851 | -- | intronic_lncRNA | 0.603937 | 0.592765 | 0.610293 | 0.696673 |
| LNC_003549 | XLOC_075280 | -- | lincRNA | 3.238446 | 1.24321 | 0.720089 | 0.557867 |
| LNC_006486 | XLOC_156093 | -- | lincRNA | 0 | 0.671807 | 0 | 0 |
| LNC_005351 | XLOC_122813 | -- | intronic_lncRNA | 0.435236 | 0.285577 | 0.47128 | 0.414646 |
| LNC_000270 | XLOC_003943 | -- | intronic_lncRNA | 0 | 0 | 0.992267 | 0 |
| LNC_006487 | XLOC_156167 | -- | intronic_lncRNA | 1.008254 | 0.62702 | 0.59372 | 0.469329 |
| LNC_000273 | XLOC_003966 | -- | intronic_lncRNA | 1.014762 | 0.25324 | 0.394027 | 0.417679 |
| LNC_000272 | XLOC_003957 | -- | lincRNA | 1.275845 | 2.107165 | 2.752317 | 2.189264 |
| LNC_000275 | XLOC_003998 | -- | intronic_lncRNA | 0.419952 | 0.228044 | 10.028395 | 9.077814 |
| LNC_000274 | XLOC_003988 | -- | intronic_lncRNA | 0.996737 | 0.29548 | 1.129633 | 0.731037 |
| LNC_005113 | XLOC_116905 | -- | intronic_lncRNA | 0.492027 | 0 | 0.630396 | 0.495204 |
| LNC_000472 | XLOC_008376 | -- | intronic_lncRNA | 0.488484 | 0.240445 | 0.124994 | 0 |
| LNC_005111 | XLOC_116900 | -- | intronic_lncRNA | 0.443405 | 0.743481 | 0.366582 | 0.328493 |
| LNC_005110 | XLOC_116899 | -- | intronic_lncRNA | 0.736194 | 0.541099 | 0.397221 | 0.241164 |
| LNC_000477 | XLOC_008404 | -- | intronic_lncRNA | 0 | 0.725784 | 0 | 0.280368 |
| LNC_000476 | XLOC_008403 | -- | intronic_lncRNA | 0.421724 | 0.246222 | 0.506992 | 0.708514 |
| LNC_000475 | XLOC_008393 | -- | intronic_lncRNA | 0.947125 | 0.302259 | 0.107363 | 0.566063 |
| LNC_000474 | XLOC_008379 | -- | intronic_lncRNA | 0.392572 | 0.706636 | 0.805862 | 0.912356 |
| LNC_005119 | XLOC_117072 | -- | intronic_lncRNA | 0 | 0 | 0.172386 | 0.575059 |
| LNC_000478 | XLOC_008405 | -- | intronic_lncRNA | 0 | 0.442674 | 0.651159 | 0.459391 |
| LNC_005721 | XLOC_134198 | -- | lincRNA | 0.953454 | 0.267486 | 0.186215 | 0.059198 |
| LNC_005359 | XLOC_123204 | -- | intronic_lncRNA | 0.547564 | 0.964102 | 0.71537 | 0.086502 |
| LNC_000278 | XLOC_004009 | -- | intronic_lncRNA | 0.892341 | 0 | 0 | 0 |
| LNC_005877 | XLOC_138364 | -- | intronic_lncRNA | 0.333266 | 0.158468 | 0.063313 | 0.6713 |
| LNC_005238 | XLOC_119832 | -- | intronic_lncRNA | 0.943779 | 0.17236 | 0.069794 | 0.170733 |
| LNC_005239 | XLOC_119839 | -- | intronic_lncRNA | 0.847161 | 0.93703 | 0.875575 | 1.013375 |
| LNC_005236 | XLOC_119795 | -- | intronic_lncRNA | 0.630724 | 0.23609 | 0.73293 | 0.445736 |
| LNC_005237 | XLOC_119827 | -- | lincRNA | 1.024827 | 0.308507 | 0.403364 | 0.41634 |
| LNC_005234 | XLOC_119788 | -- | intronic_lncRNA | 0.376268 | 0 | 0.623236 | 0.228531 |
| LNC_005235 | XLOC_119789 | -- | intronic_lncRNA | 0.530864 | 0 | 0.286689 | 0.424599 |
| LNC_005232 | XLOC_119665 | -- | intronic_lncRNA | 0.103376 | 0 | 0.523195 | 0.174368 |
| LNC_005233 | XLOC_119730 | -- | lincRNA | 1.149008 | 0.224167 | 0.108605 | 0 |
| LNC_005230 | XLOC_119625 | -- | lincRNA | 0.534885 | 0 | 0.101837 | 0 |
| LNC_005231 | XLOC_119649 | -- | intronic_lncRNA | 0.463058 | 0.467736 | 0.635333 | 0.183266 |
| LNC_002938 | XLOC_067372 | -- | intronic_lncRNA | 0.568328 | 0.460407 | 0.682276 | 0.864033 |
| LNC_002939 | XLOC_067382 | -- | lincRNA | 0.505475 | 0.321571 | 0 | 0 |
| LNC_002930 | XLOC_067315 | -- | intronic_lncRNA | 0.884117 | 0.314362 | 1.375749 | 0.729159 |
| LNC_002931 | XLOC_067315 | -- | intronic_lncRNA | 0.700631 | 0.322903 | 0.45571 | 0.962772 |
| LNC_002932 | XLOC_067315 | -- | intronic_lncRNA | 0.777066 | 0.413567 | 0.792557 | 1.52486 |
| LNC_002933 | XLOC_067315 | -- | intronic_lncRNA | 3.214965 | 2.106933 | 2.792715 | 2.588569 |
| LNC_002934 | XLOC_067354 | -- | intronic_lncRNA | 0.941419 | 0.484533 | 0.836464 | 0.71355 |
| LNC_002935 | XLOC_067364 | -- | intronic_lncRNA | 0.663611 | 0.404973 | 0.386531 | 0.066285 |
| LNC_002936 | XLOC_067364 | -- | intronic_lncRNA | 1.240919 | 0.171608 | 0.294251 | 1.14078 |
| LNC_002937 | XLOC_067365 | -- | intronic_lncRNA | 1.30864 | 0.565594 | 0.735027 | 0.403422 |
| LNC_006255 | XLOC_150190 | -- | intronic_lncRNA | 0.754836 | 0.416845 | 0.568273 | 0.760618 |
| LNC_006254 | XLOC_150151 | -- | intronic_lncRNA | 0 | 0.638759 | 0.103461 | 0.205507 |
| LNC_006257 | XLOC_150191 | -- | intronic_lncRNA | 0.811943 | 0.759595 | 0.837692 | 1.156383 |
| LNC_006256 | XLOC_150190 | -- | intronic_lncRNA | 0.402367 | 0.177929 | 0.864637 | 0.284119 |
| LNC_006251 | XLOC_150144 | -- | intronic_lncRNA | 0.005119 | 1.200441 | 0.021063 | 0.032955 |
| LNC_006250 | XLOC_150144 | -- | intronic_lncRNA | 1.215985 | 0.300249 | 1.59601 | 1.220183 |
| LNC_000060 | XLOC_000611 | -- | lincRNA | 3.259694 | 10.77147 | 4.595742 | 5.585824 |
| LNC_005581 | XLOC_129902 | -- | intronic_lncRNA | 3.687547 | 3.258336 | 0.103105 | 0 |
| LNC_000062 | XLOC_000611 | -- | lincRNA | 4.792693 | 0 | 3.795669 | 0 |
| LNC_000063 | XLOC_000611 | -- | lincRNA | 3.809974 | 5.882802 | 6.991924 | 5.047494 |
| LNC_000064 | XLOC_000611 | -- | lincRNA | 16.849791 | 22.674017 | 0.929442 | 9.640604 |
| LNC_000065 | XLOC_000611 | -- | lincRNA | 3.016007 | 3.943378 | 27.531044 | 0.937068 |
| LNC_000066 | XLOC_000611 | -- | lincRNA | 4.499077 | 0 | 15.267925 | 0 |
| LNC_000067 | XLOC_000611 | -- | lincRNA | 0 | 0 | 0.167987 | 4.507502 |
| LNC_000068 | XLOC_000611 | -- | lincRNA | 0 | 0 | 18.790493 | 0 |
| LNC_000069 | XLOC_000611 | -- | lincRNA | 0.317711 | 95.895012 | 1.982339 | 0.108207 |
| LNC_006252 | XLOC_150144 | -- | intronic_lncRNA | 0.150892 | 0.736826 | 0.552282 | 0.228819 |
| LNC_006238 | XLOC_149581 | -- | intronic_lncRNA | 0.815515 | 0.980335 | 0.506964 | 0.508372 |
| LNC_004189 | XLOC_090370 | -- | intronic_lncRNA | 1.263351 | 1.003775 | 0.310143 | 0.680862 |
| LNC_004188 | XLOC_090313 | -- | intronic_lncRNA | 0.686832 | 0.4063 | 0.504537 | 0.325699 |
| LNC_004187 | XLOC_090312 | -- | intronic_lncRNA | 0.816015 | 0.590666 | 0.370436 | 0.153581 |
| LNC_004186 | XLOC_090300 | -- | intronic_lncRNA | 0.315066 | 0.460537 | 0.445951 | 0.129678 |
| LNC_004185 | XLOC_090282 | -- | intronic_lncRNA | 0.146171 | 0.229749 | 0.498627 | 0.354319 |
| LNC_004184 | XLOC_090280 | -- | intronic_lncRNA | 0.498592 | 0.375867 | 0.460544 | 0.343644 |

| LNC_004183 | XLOC_090274 | -- | intronic_lncRNA | 1.712887 | 0.028359 | 0.394778 | 0 |
| --- | --- | --- | --- | --- | --- | --- | --- |
| LNC_004182 | XLOC_090250 | -- | antisense_lncRNA | 0.061562 | 0 | 0 | 0.453193 |
| LNC_004181 | XLOC_090250 | -- | intronic_lncRNA | 0.398322 | 0.360682 | 0.403748 | 0.157207 |
| LNC_004180 | XLOC_090098 | -- | intronic_lncRNA | 0 | 0.505441 | 0 | 0.370726 |
| LNC_001919 | XLOC_043251 | -- | intronic_lncRNA | 0.110111 | 0.124378 | 0.354829 | 0.450003 |
| LNC_001918 | XLOC_043236 | -- | intronic_lncRNA | 1.659247 | 1.165585 | 1.501861 | 2.062743 |
| LNC_001917 | XLOC_043236 | -- | intronic_lncRNA | 0.550711 | 0.530312 | 0.851749 | 0.805392 |
| LNC_001916 | XLOC_043236 | -- | intronic_lncRNA | 0 | 0.496347 | 0.805657 | 0 |
| LNC_001915 | XLOC_043227 | -- | antisense_lncRNA | 0.903863 | 1.413736 | 0.831963 | 0.880155 |
| LNC_001914 | XLOC_043197 | -- | intronic_lncRNA | 1.302863 | 0.325839 | 0.150665 | 0 |
| LNC_001913 | XLOC_043194 | -- | intronic_lncRNA | 1.193264 | 0.356609 | 0.089045 | 0.044515 |
| LNC_001912 | XLOC_043186 | -- | intronic_lncRNA | 1.33872 | 0.415309 | 0.078081 | 0.192427 |
| LNC_001911 | XLOC_043181 | -- | intronic_lncRNA | 0.673316 | 0.145343 | 0 | 0 |
| LNC_001910 | XLOC_043126 | -- | intronic_lncRNA | 0.311483 | 0.411924 | 0 | 0 |
| LNC_002336 | XLOC_053991 | -- | intronic_lncRNA | 1.803274 | 1.210164 | 1.061035 | 1.263668 |
| LNC_002337 | XLOC_053996 | -- | intronic_lncRNA | 0.410459 | 0 | 0.063301 | 0 |
| LNC_002334 | XLOC_053954 | -- | intronic_lncRNA | 0.485305 | 1.240278 | 1.140421 | 1.904057 |
| LNC_002335 | XLOC_053968 | -- | antisense_lncRNA | 0 | 0.354553 | 0.32847 | 0.66276 |
| LNC_002332 | XLOC_053918 | -- | intronic_lncRNA | 0.924534 | 0.3526 | 0.504473 | 0.655275 |
| LNC_002333 | XLOC_053919 | -- | intronic_lncRNA | 0.911595 | 0.503855 | 0.233647 | 0.359326 |
| LNC_002330 | XLOC_053910 | -- | intronic_lncRNA | 0.245915 | 0.403921 | 0.622708 | 0.549186 |
| LNC_002331 | XLOC_053910 | -- | intronic_lncRNA | 0.487932 | 0.171603 | 0.097043 | 0.106703 |
| LNC_002338 | XLOC_053996 | -- | intronic_lncRNA | 0.154988 | 0.581975 | 0.293328 | 1.247683 |
| LNC_002339 | XLOC_053998 | -- | intronic_lncRNA | 0 | 0 | 0 | 0.545458 |
| LNC_003326 | XLOC_075175 | -- | lincRNA | 2.69702 | 1.565762 | 0.420954 | 0.847943 |
| LNC_003327 | XLOC_075175 | -- | lincRNA | 3.091093 | 1.115426 | 1.101429 | 0.884288 |
| LNC_003324 | XLOC_075172 | -- | lincRNA | 1.714403 | 0.974072 | 0.446888 | 0.323256 |
| LNC_003325 | XLOC_075173 | -- | lincRNA | 3.410271 | 2.81016 | 1.57379 | 1.619977 |
| LNC_003322 | XLOC_075168 | -- | lincRNA | 2.28948 | 1.041301 | 0.912236 | 0.576116 |
| LNC_003323 | XLOC_075170 | -- | lincRNA | 1.597347 | 0.246385 | 0.624494 | 0.107824 |
| LNC_003320 | XLOC_075166 | -- | lincRNA | 0.801924 | 0.398026 | 0.130908 | 0.702834 |
| LNC_003321 | XLOC_075167 | -- | lincRNA | 2.371468 | 1.55127 | 1.429378 | 0.461264 |
| LNC_003328 | XLOC_075175 | -- | lincRNA | 6.651161 | 3.928299 | 2.111284 | 3.030245 |
| LNC_003329 | XLOC_075175 | -- | lincRNA | 2.447615 | 1.822263 | 0.772218 | 1.191612 |
| LNC_000668 | XLOC_012938 | -- | lincRNA | 0.068676 | 0 | 0.410586 | 0 |
| LNC_000669 | XLOC_013210 | -- | intronic_lncRNA | 8.758283 | 4.056282 | 6.824141 | 7.002109 |
| LNC_000666 | XLOC_012938 | -- | lincRNA | 0 | 0.099023 | 0.526511 | 0 |
| LNC_000667 | XLOC_012938 | -- | lincRNA | 0.561197 | 0.606573 | 0.651921 | 0.305159 |
| LNC_000664 | XLOC_012905 | -- | lincRNA | 1.529498 | 0.724012 | 0.384779 | 0.555907 |
| LNC_000665 | XLOC_012935 | -- | lincRNA | 0.785663 | 0.352579 | 0.254202 | 0.215069 |
| LNC_000662 | XLOC_012829 | -- | intronic_lncRNA | 0.519014 | 0 | 0 | 0 |
| LNC_000663 | XLOC_012847 | -- | intronic_lncRNA | 1.559462 | 0.712508 | 1.128629 | 0.381828 |
| LNC_000660 | XLOC_012789 | -- | intronic_lncRNA | 0.004132 | 0.445958 | 0.160272 | 0 |
| LNC_000661 | XLOC_012789 | -- | intronic_lncRNA | 0.151399 | 0.359895 | 0.547139 | 0.731788 |
| LNC_002747 | XLOC_063810 | -- | intronic_lncRNA | 0.653958 | 0.585172 | 0.102238 | 0 |
| LNC_002746 | XLOC_063804 | -- | intronic_lncRNA | 6.009638 | 5.130653 | 5.011053 | 3.073472 |
| LNC_002745 | XLOC_063737 | -- | intronic_lncRNA | 0.484414 | 0 | 0 | 0 |
| LNC_002744 | XLOC_063735 | -- | intronic_lncRNA | 0.165346 | 0 | 0.154743 | 0.626176 |
| LNC_002743 | XLOC_063725 | -- | lincRNA | 0.257077 | 0.519 | 0.28653 | 0.640816 |
| LNC_002742 | XLOC_063690 | -- | intronic_lncRNA | 0.409684 | 0.433925 | 0.833534 | 0.396402 |
| LNC_002741 | XLOC_063669 | -- | intronic_lncRNA | 0 | 0 | 0.112279 | 0.781066 |
| LNC_002740 | XLOC_063637 | -- | lincRNA | 0 | 0.061065 | 0.536814 | 0.112063 |
| LNC_002749 | XLOC_063858 | -- | intronic_lncRNA | 0.85072 | 0.971958 | 1.582545 | 2.437474 |
| LNC_002748 | XLOC_063830 | -- | lincRNA | 0.290775 | 0.389025 | 0.545484 | 0.466422 |
| LNC_004969 | XLOC_112895 | -- | intronic_lncRNA | 0.148336 | 0.258255 | 0.067157 | 0.563887 |
| LNC_004968 | XLOC_112894 | -- | intronic_lncRNA | 1.268464 | 0 | 0 | 0.363117 |
| LNC_004961 | XLOC_112515 | -- | intronic_lncRNA | 0.490113 | 0.311458 | 0 | 0.354675 |
| LNC_004960 | XLOC_112471 | -- | lincRNA | 0.59284 | 0.282343 | 0.55359 | 0.309155 |
| LNC_004963 | XLOC_112667 | -- | lincRNA | 4.044996 | 2.207019 | 0.28855 | 0.346796 |
| LNC_004962 | XLOC_112667 | -- | lincRNA | 5.49381 | 2.256111 | 0.13978 | 0.04458 |
| LNC_004965 | XLOC_112891 | -- | intronic_lncRNA | 0.680798 | 0.207613 | 0 | 0 |
| LNC_004964 | XLOC_112669 | -- | intronic_lncRNA | 10.95834 | 7.350705 | 8.305669 | 7.974973 |
| LNC_004967 | XLOC_112893 | -- | intronic_lncRNA | 0.594429 | 0.052105 | 0.065395 | 0.042872 |
| LNC_004966 | XLOC_112892 | -- | intronic_lncRNA | 0.566226 | 0 | 0.260952 | 0.245252 |
| LNC_001214 | XLOC_026112 | -- | intronic_lncRNA | 1.363966 | 5.898115 | 1.915124 | 2.045726 |
| LNC_001215 | XLOC_026199 | -- | lincRNA | 0.13184 | 0.493322 | 0.679146 | 0.126486 |
| LNC_001216 | XLOC_026217 | -- | intronic_lncRNA | 0 | 0.701605 | 0 | 0 |
| LNC_001217 | XLOC_026264 | -- | intronic_lncRNA | 0.259161 | 0.723783 | 0.394852 | 0.206442 |
| LNC_001210 | XLOC_026041 | -- | lincRNA | 0 | 0 | 0.665868 | 0.526061 |
| LNC_001211 | XLOC_026041 | -- | lincRNA | 0.994204 | 0.095709 | 0 | 0.279844 |
| LNC_001212 | XLOC_026052 | -- | intronic_lncRNA | 0.453571 | 1.673726 | 0.534804 | 0.029532 |
| LNC_001213 | XLOC_026055 | -- | intronic_lncRNA | 0 | 0.168867 | 0.065531 | 0.593636 |
| LNC_001218 | XLOC_026268 | -- | intronic_lncRNA | 0.570859 | 0.451551 | 0.38494 | 0.289736 |
| LNC_001219 | XLOC_026271 | -- | intronic_lncRNA | 0 | 0 | 0.335365 | 0.693338 |
| LNC_005713 | XLOC_133863 | -- | lincRNA | 0.052619 | 0.270663 | 0.725254 | 0.318402 |
| LNC_002109 | XLOC_048153 | -- | intronic_lncRNA | 0.643501 | 0 | 0.243234 | 0 |
| LNC_002108 | XLOC_048058 | -- | intronic_lncRNA | 0 | 0.039207 | 0.564969 | 0 |

| LNC_002105 | XLOC_047839 | -- | intronic_lncRNA | 0.619964 | 0.796956 | 0.927571 | 1.182672 |
| --- | --- | --- | --- | --- | --- | --- | --- |
| LNC_002104 | XLOC_047799 | -- | lincRNA | 0.592306 | 0.577282 | 0.084807 | 0 |
| LNC_002107 | XLOC_047885 | -- | intronic_lncRNA | 0.060936 | 0.146564 | 0.614602 | 0.2939 |
| LNC_002106 | XLOC_047871 | -- | intronic_lncRNA | 0.533222 | 0.321973 | 0.568904 | 0.408818 |
| LNC_002101 | XLOC_047793 | -- | intronic_lncRNA | 0.712605 | 0.129057 | 0 | 0.475071 |
| LNC_002100 | XLOC_047709 | -- | intronic_lncRNA | 3.273108 | 2.303697 | 1.15611 | 0.681897 |
| LNC_002103 | XLOC_047793 | -- | intronic_lncRNA | 0.771793 | 0.826923 | 2.082434 | 1.937655 |
| LNC_002102 | XLOC_047793 | -- | intronic_lncRNA | 0.604415 | 0 | 0.705068 | 0.347059 |
| LNC_003519 | XLOC_075263 | -- | lincRNA | 0.766773 | 0 | 0 | 1.44319 |
| LNC_003518 | XLOC_075263 | -- | lincRNA | 3.330327 | 0.909877 | 0.437144 | 1.811723 |
| LNC_003515 | XLOC_075262 | -- | lincRNA | 0.872635 | 1.172692 | 0.180855 | 0.215294 |
| LNC_003514 | XLOC_075262 | -- | lincRNA | 2.887312 | 0.900918 | 0.570658 | 0.5896 |
| LNC_003517 | XLOC_075263 | -- | lincRNA | 1.185112 | 0.736101 | 0.285097 | 0.119481 |
| LNC_003516 | XLOC_075263 | -- | lincRNA | 7.208256 | 3.286488 | 1.785849 | 2.476723 |
| LNC_003511 | XLOC_075260 | -- | lincRNA | 8.755759 | 6.660738 | 1.665922 | 3.646824 |
| LNC_003510 | XLOC_075258 | -- | lincRNA | 1.39588 | 0.598777 | 0.382281 | 0.395121 |
| LNC_003513 | XLOC_075261 | -- | lincRNA | 1.777852 | 0.044426 | 0 | 0.03507 |
| LNC_003512 | XLOC_075261 | -- | lincRNA | 4.352192 | 2.331967 | 1.611459 | 1.3838 |
| LNC_005579 | XLOC_129718 | -- | intronic_lncRNA | 0 | 0.399569 | 0.201499 | 0.403944 |
| LNC_005779 | XLOC_135639 | -- | intronic_lncRNA | 0.203739 | 0.426466 | 0.518066 | 0 |
| LNC_000098 | XLOC_000741 | -- | lincRNA | 1.179972 | 0.551377 | 0.81248 | 0.629414 |
| LNC_005777 | XLOC_135624 | -- | intronic_lncRNA | 0.792659 | 0.667981 | 0.489979 | 0.63696 |
| LNC_005776 | XLOC_135601 | -- | lincRNA | 0 | 0.487389 | 0 | 0 |
| LNC_005775 | XLOC_135592 | -- | intronic_lncRNA | 0.332169 | 1.255396 | 0.140304 | 0.384922 |
| LNC_005774 | XLOC_135591 | -- | intronic_lncRNA | 0 | 0.32383 | 0.419107 | 0.125794 |
| LNC_005773 | XLOC_135493 | -- | intronic_lncRNA | 0.724465 | 0.063055 | 0.443062 | 0.178042 |
| LNC_005772 | XLOC_135492 | -- | intronic_lncRNA | 0.512909 | 0 | 0.462002 | 0 |
| LNC_005771 | XLOC_135491 | -- | intronic_lncRNA | 0.492879 | 0.71326 | 0.186973 | 0 |
| LNC_005770 | XLOC_135396 | -- | lincRNA | 0.714121 | 0.901593 | 0.443395 | 0.392538 |
| LNC_005571 | XLOC_129336 | -- | lincRNA | 0 | 0.153083 | 0 | 0.926439 |
| LNC_000090 | XLOC_000619 | -- | lincRNA | 0 | 38.250778 | 0 | 0 |
| LNC_005573 | XLOC_129426 | -- | intronic_lncRNA | 0.374513 | 1.166241 | 0.348515 | 0 |
| LNC_000092 | XLOC_000620 | -- | lincRNA | 0.448939 | 3.019348 | 10.313478 | 25.995415 |
| LNC_005575 | XLOC_129462 | -- | intronic_lncRNA | 0.30668 | 0.200506 | 0.671554 | 0.732192 |
| LNC_005574 | XLOC_129462 | -- | intronic_lncRNA | 1.455004 | 0.565693 | 1.328868 | 0.316643 |
| LNC_004798 | XLOC_107159 | -- | intronic_lncRNA | 0.886837 | 0.304679 | 0.569607 | 0.589951 |
| LNC_004799 | XLOC_107192 | -- | intronic_lncRNA | 0.254019 | 0.195082 | 0.739976 | 0.337919 |
| LNC_005577 | XLOC_129560 | -- | intronic_lncRNA | 1.737865 | 0.475039 | 0.075769 | 0.10925 |
| LNC_004792 | XLOC_107017 | -- | intronic_lncRNA | 0.391709 | 0.624808 | 0.167544 | 0.316629 |
| LNC_004793 | XLOC_107021 | -- | intronic_lncRNA | 2.167782 | 1.616512 | 1.656082 | 1.993527 |
| LNC_004790 | XLOC_106959 | -- | lincRNA | 1.309963 | 0.594236 | 0.11979 | 0.173022 |
| LNC_005576 | XLOC_129489 | -- | intronic_lncRNA | 0.404432 | 0.423537 | 0.721563 | 0.648427 |
| LNC_004796 | XLOC_107152 | -- | intronic_lncRNA | 1.246585 | 0.651692 | 0.73478 | 1.017677 |
| LNC_004797 | XLOC_107152 | -- | intronic_lncRNA | 0.89373 | 0.946453 | 0.456458 | 0.562191 |
| LNC_004794 | XLOC_107031 | -- | lincRNA | 0.577728 | 2.215634 | 0.752842 | 0.817815 |
| LNC_004795 | XLOC_107142 | -- | intronic_lncRNA | 0.281716 | 0.62067 | 0.250588 | 0.443726 |
| LNC_001452 | XLOC_031551 | -- | intronic_lncRNA | 0.978037 | 0.029736 | 0.738814 | 0.199913 |
| LNC_001453 | XLOC_031566 | -- | intronic_lncRNA | 7.57806 | 6.038662 | 5.050333 | 5.024937 |
| LNC_001450 | XLOC_031532 | -- | antisense_lncRNA | 0.61141 | 1.250257 | 0.717119 | 0.896044 |
| LNC_001451 | XLOC_031551 | -- | intronic_lncRNA | 5.375501 | 3.623094 | 4.775823 | 4.518432 |
| LNC_001456 | XLOC_031587 | -- | intronic_lncRNA | 0.863871 | 0.425812 | 0.399778 | 0.917087 |
| LNC_001457 | XLOC_031589 | -- | intronic_lncRNA | 0.248677 | 0.379453 | 0.299873 | 0.436996 |
| LNC_001454 | XLOC_031566 | -- | intronic_lncRNA | 0.04054 | 0.019472 | 1.138934 | 4.90053 |
| LNC_001455 | XLOC_031586 | -- | intronic_lncRNA | 0.201392 | 0 | 0.78841 | 0.083237 |
| LNC_006484 | XLOC_156080 | -- | lincRNA | 0.741113 | 0.334188 | 0.875431 | 0.807404 |
| LNC_006485 | XLOC_156081 | -- | lincRNA | 0.266259 | 0.192377 | 0.563538 | 0.683536 |
| LNC_001458 | XLOC_031592 | -- | intronic_lncRNA | 0.31843 | 0.811878 | 0.341402 | 0.247462 |
| LNC_001459 | XLOC_031601 | -- | lincRNA | 0.077247 | 0.716462 | 0.062324 | 0.09876 |
| LNC_006480 | XLOC_155934 | -- | intronic_lncRNA | 0.17391 | 0.867007 | 0.276374 | 0.331617 |
| LNC_006481 | XLOC_155934 | -- | intronic_lncRNA | 0.12196 | 0.48885 | 0.538686 | 0.022762 |
| LNC_006482 | XLOC_155934 | -- | intronic_lncRNA | 0.083045 | 0.023824 | 0.49407 | 0.630377 |
| LNC_006483 | XLOC_156074 | -- | lincRNA | 0.391736 | 0.486189 | 0.568125 | 0.733456 |
| LNC_005827 | XLOC_137284 | -- | intronic_lncRNA | 0.195734 | 0 | 0.768541 | 0.977958 |
| LNC_005826 | XLOC_137281 | -- | intronic_lncRNA | 0.723539 | 0.168416 | 0 | 0 |
| LNC_005825 | XLOC_137265 | -- | intronic_lncRNA | 0.238386 | 0.410353 | 0.072171 | 0.461816 |
| LNC_005824 | XLOC_137263 | -- | intronic_lncRNA | 1.247967 | 0.782168 | 0.075395 | 0.075382 |
| LNC_004428 | XLOC_096698 | -- | intronic_lncRNA | 0 | 0.506544 | 0 | 0.459378 |
| LNC_004429 | XLOC_096735 | -- | intronic_lncRNA | 0 | 0.092503 | 0.372305 | 0.649894 |
| LNC_005821 | XLOC_137250 | -- | intronic_lncRNA | 0.523298 | 0.623057 | 0.704369 | 1.786102 |
| LNC_005820 | XLOC_137248 | -- | intronic_lncRNA | 0 | 0.456579 | 0.831171 | 0.599788 |
| LNC_004424 | XLOC_096619 | -- | intronic_lncRNA | 0 | 0 | 0.763044 | 0.44176 |
| LNC_004425 | XLOC_096619 | -- | antisense_lncRNA | 0 | 0.421103 | 0.498215 | 0 |
| LNC_004426 | XLOC_096620 | -- | intronic_lncRNA | 0.348703 | 0.241305 | 0.669562 | 0.413047 |
| LNC_004427 | XLOC_096690 | -- | antisense_lncRNA | 0.457588 | 0.185301 | 0.611902 | 0.549164 |
| LNC_004420 | XLOC_096536 | -- | intronic_lncRNA | 0 | 0.239784 | 0.092997 | 1.107056 |
| LNC_004421 | XLOC_096543 | -- | antisense_lncRNA | 0.668224 | 0.3165 | 0.437398 | 0.43682 |
| LNC_004422 | XLOC_096550 | -- | intronic_lncRNA | 0.329564 | 0.238353 | 0 | 0.646333 |

| LNC_004423 | XLOC_096550 | -- | intronic_lncRNA | 0.625698 | 0.247151 | 0.564645 | 0.383298 |
| --- | --- | --- | --- | --- | --- | --- | --- |
| LNC_006156 | XLOC_147579 | -- | intronic_lncRNA | 0 | 0 | 0.769394 | 0 |
| LNC_006157 | XLOC_147609 | -- | intronic_lncRNA | 0.099537 | 0.328398 | 0.399649 | 0.428887 |
| LNC_006154 | XLOC_147468 | -- | lincRNA | 0.313209 | 0 | 0.393167 | 0.280605 |
| LNC_006155 | XLOC_147566 | -- | intronic_lncRNA | 0.167863 | 0.543744 | 0 | 0 |
| LNC_006152 | XLOC_147236 | -- | intronic_lncRNA | 0.309989 | 0.74909 | 0.707404 | 1.203222 |
| LNC_006153 | XLOC_147251 | -- | intronic_lncRNA | 0.402793 | 0.211077 | 0.503268 | 0.559899 |
| LNC_006150 | XLOC_147214 | -- | intronic_lncRNA | 0.655892 | 0.520093 | 0.048682 | 0.123328 |
| LNC_006151 | XLOC_147223 | -- | antisense_lncRNA | 0.159983 | 0.042255 | 0.854093 | 1.427749 |
| LNC_006158 | XLOC_147635 | -- | intronic_lncRNA | 0.241863 | 0.081093 | 0.571154 | 0.53687 |
| LNC_006159 | XLOC_147656 | -- | intronic_lncRNA | 0 | 0.525163 | 0.241298 | 0.101191 |
| LNC_005175 | XLOC_118127 | -- | intronic_lncRNA | 0.716084 | 0.534028 | 0.701245 | 1.090695 |
| LNC_005174 | XLOC_118112 | -- | intronic_lncRNA | 0 | 0 | 0 | 0.428966 |
| LNC_005177 | XLOC_118251 | -- | intronic_lncRNA | 0.188471 | 0.09906 | 0.441233 | 0.299318 |
| LNC_000416 | XLOC_007354 | -- | intronic_lncRNA | 0.236919 | 0.609028 | 0.482101 | 0.76999 |
| LNC_005171 | XLOC_118061 | -- | intronic_lncRNA | 0.596332 | 0 | 0.440141 | 0.584232 |
| LNC_005170 | XLOC_118060 | -- | intronic_lncRNA | 0.055361 | 0 | 0 | 0.447268 |
| LNC_000413 | XLOC_007120 | -- | intronic_lncRNA | 0.292524 | 0.182319 | 0.310343 | 0.864337 |
| LNC_000412 | XLOC_007120 | -- | intronic_lncRNA | 1.912641 | 1.415887 | 1.681517 | 1.139998 |
| LNC_002480 | XLOC_057537 | -- | intronic_lncRNA | 0 | 0 | 0.616017 | 0.46142 |
| LNC_002481 | XLOC_057558 | -- | intronic_lncRNA | 2.285643 | 0.800757 | 1.202579 | 0.951615 |
| LNC_002482 | XLOC_057579 | -- | intronic_lncRNA | 0.826038 | 0.876299 | 0.183691 | 0.185992 |
| LNC_002483 | XLOC_057620 | -- | intronic_lncRNA | 0.614225 | 1.553418 | 0.482456 | 0.880977 |
| LNC_002484 | XLOC_057632 | -- | intronic_lncRNA | 0.3325 | 0.146334 | 0.373678 | 0.746105 |
| LNC_002485 | XLOC_057648 | -- | lincRNA | 0 | 0.176209 | 0 | 0.682971 |
| LNC_002486 | XLOC_057684 | -- | intronic_lncRNA | 0.760176 | 1.791277 | 1.496571 | 1.316373 |
| LNC_002487 | XLOC_057747 | -- | intronic_lncRNA | 0.143246 | 0 | 0.415515 | 0.203785 |
| LNC_004888 | XLOC_111119 | -- | intronic_lncRNA | 0.665741 | 0.0297 | 0.209191 | 0.2946 |
| LNC_004889 | XLOC_111119 | -- | intronic_lncRNA | 0.555846 | 0.00372 | 0.026428 | 0 |
| LNC_004886 | XLOC_111118 | -- | intronic_lncRNA | 0.743454 | 0.864339 | 0.807687 | 0.668796 |
| LNC_004887 | XLOC_111118 | -- | intronic_lncRNA | 0.337753 | 0.151526 | 0.922188 | 0.196157 |
| LNC_004884 | XLOC_111089 | -- | intronic_lncRNA | 0 | 0.75261 | 0.305008 | 0.253621 |
| LNC_004885 | XLOC_111090 | -- | intronic_lncRNA | 0 | 0.277067 | 0.373975 | 0.447945 |
| LNC_004882 | XLOC_110906 | -- | intronic_lncRNA | 0.578757 | 0.318719 | 0.40138 | 0.711665 |
| LNC_004883 | XLOC_111061 | -- | lincRNA | 0.174138 | 0.27447 | 0.428359 | 0.495467 |
| LNC_004880 | XLOC_110663 | -- | intronic_lncRNA | 0.574349 | 0.115537 | 0.380511 | 0.16091 |
| LNC_004881 | XLOC_110664 | -- | intronic_lncRNA | 0.377139 | 0.46819 | 0.558893 | 0.780063 |
| LNC_003689 | XLOC_079019 | -- | intronic_lncRNA | 2.06925 | 2.117956 | 1.701699 | 0.810069 |
| LNC_003688 | XLOC_079010 | -- | intronic_lncRNA | 0.897383 | 1.293051 | 0.978789 | 0.646343 |
| LNC_003685 | XLOC_078870 | -- | intronic_lncRNA | 0.227022 | 0.144368 | 0.347617 | 0.880863 |
| LNC_003684 | XLOC_078817 | -- | intronic_lncRNA | 0.777406 | 0.943658 | 0.224698 | 0.245547 |
| LNC_003687 | XLOC_079008 | -- | intronic_lncRNA | 0.477726 | 0.04524 | 0.089488 | 0.093909 |
| LNC_003686 | XLOC_079007 | -- | intronic_lncRNA | 1.130298 | 0.65117 | 0.573651 | 0.265329 |
| LNC_003681 | XLOC_078640 | -- | intronic_lncRNA | 0.728695 | 0 | 0.231867 | 0.343056 |
| LNC_003680 | XLOC_078638 | -- | intronic_lncRNA | 0.965607 | 0 | 0.176522 | 0 |
| LNC_003683 | XLOC_078816 | -- | intronic_lncRNA | 0.766463 | 0.333909 | 0.455802 | 0.499388 |
| LNC_003682 | XLOC_078727 | -- | lincRNA | 0.378615 | 0.269154 | 0.622725 | 0.140042 |
| LNC_005210 | XLOC_119021 | -- | intronic_lncRNA | 1.029138 | 0 | 0.155534 | 0.534945 |
| LNC_005211 | XLOC_119022 | -- | intronic_lncRNA | 0.193173 | 0.161996 | 0.273526 | 0.356064 |
| LNC_005212 | XLOC_119023 | -- | intronic_lncRNA | 0.916377 | 0.068557 | 0.499324 | 0.291993 |
| LNC_005213 | XLOC_119101 | -- | intronic_lncRNA | 0.632352 | 0.67279 | 0.700664 | 0.672652 |
| LNC_005214 | XLOC_119101 | -- | intronic_lncRNA | 0.575157 | 0.001329 | 0.062328 | 0.038672 |
| LNC_005215 | XLOC_119206 | -- | intronic_lncRNA | 0.589083 | 0 | 0.324986 | 0.434104 |
| LNC_005216 | XLOC_119207 | -- | intronic_lncRNA | 0.49096 | 0 | 0 | 0.236402 |
| LNC_005217 | XLOC_119285 | -- | lincRNA | 0.46448 | 0 | 0 | 0.199475 |
| LNC_005218 | XLOC_119297 | -- | intronic_lncRNA | 0.984248 | 0 | 0 | 0.155461 |
| LNC_005219 | XLOC_119540 | -- | intronic_lncRNA | 0.343226 | 0.730983 | 0.413425 | 0.194063 |
| LNC_004385 | XLOC_095540 | -- | intronic_lncRNA | 0.840585 | 0.727002 | 1.549288 | 1.546048 |
| LNC_004836 | XLOC_108648 | -- | intronic_lncRNA | 0.429718 | 0.602997 | 0.113828 | 0.236996 |
| LNC_004835 | XLOC_108563 | -- | lincRNA | 2.958714 | 1.891628 | 0.536191 | 0.992592 |
| LNC_000114 | XLOC_000964 | -- | intronic_lncRNA | 0.136132 | 0.840296 | 0.665769 | 1.133978 |
| LNC_004834 | XLOC_108563 | -- | lincRNA | 2.992421 | 2.223103 | 1.171767 | 1.168179 |
| LNC_005435 | XLOC_125456 | -- | lincRNA | 0.136987 | 0.288863 | 0.967422 | 1.665193 |
| LNC_004381 | XLOC_095460 | -- | antisense_lncRNA | 0.72811 | 0.445861 | 0.602817 | 0.85558 |
| LNC_000116 | XLOC_000979 | -- | intronic_lncRNA | 0 | 0.290933 | 0.542568 | 0.198388 |
| LNC_004832 | XLOC_108541 | -- | intronic_lncRNA | 0.256047 | 0 | 0.419386 | 0 |
| LNC_000117 | XLOC_001003 | -- | intronic_lncRNA | 0 | 0.496079 | 0.26184 | 0.399686 |
| LNC_004831 | XLOC_108417 | -- | intronic_lncRNA | 0.69057 | 0.313686 | 0.712532 | 0.673878 |
| LNC_005430 | XLOC_125339 | -- | intronic_lncRNA | 0.18759 | 0.133184 | 0.524337 | 0.302743 |
| LNC_004830 | XLOC_108411 | -- | intronic_lncRNA | 0.394464 | 0.201441 | 0.505161 | 0.343991 |
| LNC_005431 | XLOC_125365 | -- | lincRNA | 0.645373 | 0.099374 | 0 | 0 |
| LNC_004228 | XLOC_091780 | -- | intronic_lncRNA | 0.219638 | 0.219694 | 0.482167 | 0.396993 |
| LNC_004229 | XLOC_091782 | -- | intronic_lncRNA | 0 | 0.535751 | 0 | 0.074632 |
| LNC_005432 | XLOC_125409 | -- | intronic_lncRNA | 0.783652 | 0.071428 | 0.580621 | 0.318405 |
| LNC_004222 | XLOC_091534 | -- | intronic_lncRNA | 0 | 0 | 0.396722 | 0.20845 |
| LNC_004223 | XLOC_091541 | -- | antisense_lncRNA | 0 | 0 | 0.455197 | 0 |
| LNC_004220 | XLOC_091302 | -- | intronic_lncRNA | 1.824685 | 0 | 1.079416 | 0.465669 |

| LNC_004221 | XLOC_091305 | -- | intronic_lncRNA | 0.257504 | 0.677507 | 0.134856 | 0.75434 |
| --- | --- | --- | --- | --- | --- | --- | --- |
| LNC_004226 | XLOC_091703 | -- | intronic_lncRNA | 0.501566 | 0.394566 | 0.869358 | 1.297152 |
| LNC_004227 | XLOC_091718 | -- | intronic_lncRNA | 0.689023 | 0.387726 | 0.665259 | 0.826092 |
| LNC_004224 | XLOC_091559 | -- | intronic_lncRNA | 2.037076 | 2.244058 | 0.798731 | 2.061336 |
| LNC_004225 | XLOC_091571 | -- | intronic_lncRNA | 0.691879 | 0.475517 | 0.629195 | 0.688679 |
| LNC_000118 | XLOC_001004 | -- | intronic_lncRNA | 0.221728 | 0.491041 | 0 | 0.079468 |
| LNC_000119 | XLOC_001011 | -- | lincRNA | 0.160737 | 0.329542 | 0.802426 | 0.592821 |
| LNC_004161 | XLOC_089660 | -- | intronic_lncRNA | 0.182647 | 0.191417 | 0.388585 | 0.42055 |
| LNC_004160 | XLOC_089621 | -- | intronic_lncRNA | 0.530864 | 0.108238 | 0.3303 | 0.334532 |
| LNC_004163 | XLOC_089722 | -- | intronic_lncRNA | 0.428685 | 1.019693 | 0.83135 | 0 |
| LNC_004162 | XLOC_089690 | -- | intronic_lncRNA | 0.501061 | 0.171003 | 1.097761 | 0.967486 |
| LNC_004165 | XLOC_089802 | -- | intronic_lncRNA | 1.006428 | 0.432122 | 0.450005 | 0.492621 |
| LNC_004164 | XLOC_089766 | -- | intronic_lncRNA | 0.110724 | 0.119833 | 0.441892 | 0 |
| LNC_004167 | XLOC_089813 | -- | intronic_lncRNA | 0.632649 | 1.029514 | 0.221167 | 0.082549 |
| LNC_004166 | XLOC_089808 | -- | intronic_lncRNA | 0.463833 | 0.203322 | 0.77253 | 1.14431 |
| LNC_004169 | XLOC_089848 | -- | lincRNA | 0 | 0 | 0.31499 | 0.493971 |
| LNC_004168 | XLOC_089845 | -- | lincRNA | 0.17969 | 0 | 1.028722 | 0.684728 |
| LNC_002318 | XLOC_053598 | -- | lincRNA | 0.235888 | 0.39728 | 0.878159 | 1.022909 |
| LNC_002319 | XLOC_053648 | -- | lincRNA | 2.863589 | 0.269347 | 0.356163 | 0.362038 |
| LNC_002310 | XLOC_053041 | -- | intronic_lncRNA | 0 | 0 | 0.890405 | 0.380883 |
| LNC_002311 | XLOC_053041 | -- | intronic_lncRNA | 0.335363 | 0.341118 | 0.072452 | 0.457191 |
| LNC_002312 | XLOC_053327 | -- | lincRNA | 0.882351 | 0.467668 | 0.757092 | 0.491453 |
| LNC_002313 | XLOC_053434 | -- | intronic_lncRNA | 0.492575 | 0.570216 | 0.750423 | 0.490422 |
| LNC_002314 | XLOC_053455 | -- | intronic_lncRNA | 0.379036 | 0.104573 | 0.098061 | 0.538916 |
| LNC_002315 | XLOC_053456 | -- | intronic_lncRNA | 0.200969 | 0.235372 | 0.622925 | 0.574535 |
| LNC_002316 | XLOC_053508 | -- | intronic_lncRNA | 0.924342 | 1.058298 | 1.173889 | 1.367744 |
| LNC_002317 | XLOC_053532 | -- | lincRNA | 0.051597 | 0.02982 | 0.931376 | 0.936044 |
| LNC_003308 | XLOC_075158 | -- | lincRNA | 0 | 0.828984 | 0.237704 | 0 |
| LNC_003309 | XLOC_075158 | -- | lincRNA | 2.438938 | 0.449645 | 1.689671 | 0.369875 |
| LNC_006316 | XLOC_151553 | -- | intronic_lncRNA | 1.276924 | 0.462602 | 0.172148 | 0.649536 |
| LNC_003300 | XLOC_075157 | -- | lincRNA | 9.551091 | 5.007202 | 2.791498 | 2.725694 |
| LNC_003301 | XLOC_075158 | -- | lincRNA | 3.119004 | 2.079047 | 0.990484 | 1.377041 |
| LNC_003302 | XLOC_075158 | -- | lincRNA | 2.810392 | 1.077676 | 0.589856 | 0.554051 |
| LNC_003303 | XLOC_075158 | -- | lincRNA | 1.525683 | 0.452696 | 0.231581 | 0.190471 |
| LNC_003304 | XLOC_075158 | -- | lincRNA | 1.901156 | 1.080089 | 0.709794 | 1.227219 |
| LNC_003305 | XLOC_075158 | -- | lincRNA | 14.850782 | 7.495161 | 3.355397 | 0 |
| LNC_003306 | XLOC_075158 | -- | lincRNA | 1.536219 | 0.131494 | 0.087445 | 3.085948 |
| LNC_003307 | XLOC_075158 | -- | lincRNA | 3.128136 | 1.782509 | 0.57186 | 1.088588 |
| LNC_000640 | XLOC_012296 | -- | antisense_lncRNA | 0.921893 | 0.239898 | 0.112489 | 0.124195 |
| LNC_000641 | XLOC_012313 | -- | intronic_lncRNA | 0.97192 | 0.403624 | 1.403597 | 0.738864 |
| LNC_000642 | XLOC_012314 | -- | intronic_lncRNA | 0.669563 | 0.332773 | 1.377813 | 0.500316 |
| LNC_000643 | XLOC_012456 | -- | lincRNA | 0.048735 | 0 | 0.125069 | 0.423092 |
| LNC_000644 | XLOC_012460 | -- | intronic_lncRNA | 1.068825 | 0.629436 | 0.733701 | 0.916489 |
| LNC_000645 | XLOC_012461 | -- | intronic_lncRNA | 0.620285 | 0.511449 | 0.397832 | 0.222871 |
| LNC_000646 | XLOC_012475 | -- | intronic_lncRNA | 0.421725 | 0.365542 | 0.757325 | 0.341042 |
| LNC_000647 | XLOC_012505 | -- | intronic_lncRNA | 0 | 0.676377 | 0.728534 | 0.790022 |
| LNC_000648 | XLOC_012594 | -- | intronic_lncRNA | 0 | 0.560744 | 0 | 0.101652 |
| LNC_000649 | XLOC_012595 | -- | intronic_lncRNA | 0.512578 | 0.057941 | 0.469243 | 0.160347 |
| LNC_001669 | XLOC_036417 | -- | intronic_lncRNA | 0.16474 | 0.507983 | 0.095083 | 0.352594 |
| LNC_001668 | XLOC_036331 | -- | intronic_lncRNA | 0 | 0.059263 | 0.693751 | 0.005897 |
| LNC_001661 | XLOC_036304 | -- | intronic_lncRNA | 0.402298 | 0.401843 | 0 | 0.574115 |
| LNC_001660 | XLOC_036181 | -- | intronic_lncRNA | 0.739373 | 0.669016 | 0.421039 | 0.291146 |
| LNC_001663 | XLOC_036317 | -- | intronic_lncRNA | 0.527495 | 0.363679 | 0.549447 | 0.44133 |
| LNC_001662 | XLOC_036316 | -- | intronic_lncRNA | 0.688107 | 0.222355 | 0.551318 | 0.600315 |
| LNC_001665 | XLOC_036329 | -- | intronic_lncRNA | 0.217644 | 0.260334 | 0.621894 | 0.435627 |
| LNC_001664 | XLOC_036325 | -- | antisense_lncRNA | 0.310867 | 0.544689 | 0 | 0 |
| LNC_001667 | XLOC_036331 | -- | intronic_lncRNA | 0.700176 | 0.551892 | 0.236505 | 0.725897 |
| LNC_001666 | XLOC_036330 | -- | intronic_lncRNA | 0.20139 | 0.137838 | 0.271824 | 0.813883 |
| LNC_004943 | XLOC_112133 | -- | intronic_lncRNA | 1.042171 | 0 | 0 | 0.329375 |
| LNC_004942 | XLOC_112120 | -- | intronic_lncRNA | 0.533233 | 0.588438 | 0.332683 | 0.63741 |
| LNC_004941 | XLOC_112101 | -- | intronic_lncRNA | 0.677789 | 0.474962 | 1.127108 | 0.810289 |
| LNC_004940 | XLOC_112009 | -- | intronic_lncRNA | 0.158463 | 0.013677 | 0 | 0.648027 |
| LNC_004947 | XLOC_112280 | -- | intronic_lncRNA | 0.380638 | 0.523446 | 0 | 1.140976 |
| LNC_004946 | XLOC_112254 | -- | intronic_lncRNA | 0.546786 | 0.086734 | 0.95705 | 0.238965 |
| LNC_004945 | XLOC_112252 | -- | intronic_lncRNA | 0.572701 | 0.74453 | 0.533852 | 0.47108 |
| LNC_004944 | XLOC_112250 | -- | intronic_lncRNA | 0.294136 | 0.639497 | 0.528359 | 0.052363 |
| LNC_004949 | XLOC_112282 | -- | intronic_lncRNA | 0.271961 | 0.155644 | 0.495522 | 0.579369 |
| LNC_004948 | XLOC_112281 | -- | intronic_lncRNA | 0 | 0.2098 | 0 | 0.521813 |
| LNC_006426 | XLOC_153997 | -- | intronic_lncRNA | 0.854848 | 0.074053 | 0.369937 | 0.277021 |
| LNC_005128 | XLOC_117156 | -- | intronic_lncRNA | 0.887482 | 1.485093 | 2.14768 | 1.25752 |
| LNC_000449 | XLOC_007921 | -- | intronic_lncRNA | 1.11216 | 0.124718 | 0.443759 | 0.865667 |
| LNC_001278 | XLOC_027683 | -- | lincRNA | 0 | 0 | 0 | 0.407695 |
| LNC_001279 | XLOC_027685 | -- | intronic_lncRNA | 0 | 0.345999 | 0 | 0.630989 |
| LNC_001276 | XLOC_027627 | -- | lincRNA | 0.972965 | 0.133315 | 0.058616 | 0 |
| LNC_001277 | XLOC_027639 | -- | lincRNA | 0.06182 | 0.083487 | 0.75154 | 0.1866 |
| LNC_001274 | XLOC_027462 | -- | intronic_lncRNA | 0.073027 | 0.291454 | 0 | 0.354584 |
| LNC_001275 | XLOC_027463 | -- | intronic_lncRNA | 0.357721 | 0.267689 | 0 | 0 |

| LNC_001272 | XLOC_027409 | -- | intronic_lncRNA | 0 | 0.55876 | 0 | 0.180976 |
| --- | --- | --- | --- | --- | --- | --- | --- |
| LNC_001273 | XLOC_027413 | -- | intronic_lncRNA | 0 | 0.356984 | 0.618043 | 0.617936 |
| LNC_001270 | XLOC_027391 | -- | intronic_lncRNA | 0 | 0.513268 | 0 | 0 |
| LNC_001271 | XLOC_027396 | -- | intronic_lncRNA | 2.227318 | 2.656829 | 3.189239 | 4.385086 |
| LNC_005122 | XLOC_117107 | -- | intronic_lncRNA | 0 | 0 | 0.498641 | 0.334859 |
| LNC_005123 | XLOC_117116 | -- | intronic_lncRNA | 0 | 0 | 0.475659 | 0.118894 |
| LNC_005120 | XLOC_117097 | -- | intronic_lncRNA | 0.546674 | 0.558781 | 0 | 0 |
| LNC_005121 | XLOC_117107 | -- | intronic_lncRNA | 0.375042 | 0.031435 | 0.525993 | 1.060665 |
| LNC_005126 | XLOC_117120 | -- | intronic_lncRNA | 0 | 0.071959 | 0.142296 | 0.58267 |
| LNC_005127 | XLOC_117148 | -- | intronic_lncRNA | 0.775956 | 0.011685 | 0.0274 | 0 |
| LNC_005124 | XLOC_117118 | -- | intronic_lncRNA | 0.243509 | 0.044369 | 0.48711 | 1.029868 |
| LNC_005125 | XLOC_117119 | -- | intronic_lncRNA | 0.664028 | 0.926905 | 1.09746 | 0.611733 |
| LNC_006212 | XLOC_148928 | -- | lincRNA | 0.966952 | 0.253472 | 0.107552 | 0.227447 |
| LNC_001898 | XLOC_042900 | -- | intronic_lncRNA | 0 | 0.056227 | 0.305237 | 0.382436 |
| LNC_001899 | XLOC_042925 | -- | intronic_lncRNA | 0 | 0 | 0.626666 | 0.09627 |
| LNC_001892 | XLOC_042633 | -- | intronic_lncRNA | 0.114428 | 0.208554 | 0.184926 | 0.924472 |
| LNC_001893 | XLOC_042634 | -- | intronic_lncRNA | 0.541023 | 0.377817 | 0.846711 | 0.37018 |
| LNC_001890 | XLOC_042544 | -- | intronic_lncRNA | 0.553616 | 0.138094 | 0 | 0 |
| LNC_001891 | XLOC_042545 | -- | intronic_lncRNA | 0.611398 | 0.322417 | 0.666393 | 0.506674 |
| LNC_001896 | XLOC_042728 | -- | intronic_lncRNA | 1.196844 | 0.796387 | 0.824822 | 1.248432 |
| LNC_001897 | XLOC_042894 | -- | intronic_lncRNA | 0.37543 | 0.132519 | 0.572265 | 0.339795 |
| LNC_001894 | XLOC_042637 | -- | intronic_lncRNA | 0 | 0.51555 | 0.343064 | 0.057434 |
| LNC_001895 | XLOC_042638 | -- | intronic_lncRNA | 0.180527 | 0.540366 | 0.169213 | 0 |
| LNC_002127 | XLOC_048516 | -- | intronic_lncRNA | 0.416375 | 0.09389 | 0.438215 | 0.603655 |
| LNC_002126 | XLOC_048489 | -- | intronic_lncRNA | 0 | 0 | 0.436942 | 0.685881 |
| LNC_002125 | XLOC_048442 | -- | intronic_lncRNA | 0.396287 | 0.603979 | 0.064606 | 0.070199 |
| LNC_002124 | XLOC_048441 | -- | intronic_lncRNA | 0.848782 | 0.149098 | 0 | 0.181092 |
| LNC_002123 | XLOC_048438 | -- | intronic_lncRNA | 0.469146 | 0.24116 | 0.521803 | 0.70525 |
| LNC_002122 | XLOC_048438 | -- | intronic_lncRNA | 0.744312 | 0 | 0.073079 | 0.151236 |
| LNC_002121 | XLOC_048438 | -- | intronic_lncRNA | 0 | 0 | 0.645115 | 0.095127 |
| LNC_002120 | XLOC_048404 | -- | intronic_lncRNA | 0.053195 | 0.24721 | 0 | 0.612917 |
| LNC_002129 | XLOC_048586 | -- | intronic_lncRNA | 1.709675 | 1.446889 | 1.80401 | 2.115848 |
| LNC_002128 | XLOC_048571 | -- | intronic_lncRNA | 0.396494 | 0.236732 | 0.708936 | 1.058748 |
| LNC_003537 | XLOC_075271 | -- | lincRNA | 1.798757 | 0 | 0 | 0 |
| LNC_003536 | XLOC_075271 | -- | lincRNA | 1.816073 | 0.69531 | 0.351002 | 0.571772 |
| LNC_003535 | XLOC_075270 | -- | lincRNA | 3.326951 | 2.540467 | 0.888414 | 1.93663 |
| LNC_003534 | XLOC_075269 | -- | lincRNA | 1.31986 | 0.363512 | 0.555521 | 0.744273 |
| LNC_003533 | XLOC_075268 | -- | lincRNA | 5.059391 | 1.053529 | 0.70942 | 1.449749 |
| LNC_003532 | XLOC_075267 | -- | lincRNA | 4.874351 | 2.576154 | 1.230294 | 1.503304 |
| LNC_003531 | XLOC_075266 | -- | lincRNA | 2.186966 | 0.799715 | 0.366497 | 0.066833 |
| LNC_003530 | XLOC_075266 | -- | lincRNA | 1.129098 | 0.262054 | 0.231957 | 0.267842 |
| LNC_003539 | XLOC_075271 | -- | lincRNA | 1.510421 | 0.690827 | 0.227928 | 0.183041 |
| LNC_003538 | XLOC_075271 | -- | lincRNA | 1.191862 | 1.952392 | 0.574504 | 0.912772 |
| LNC_005791 | XLOC_136164 | -- | lincRNA | 0.539347 | 0.119586 | 0.213671 | 0 |
| LNC_005790 | XLOC_136155 | -- | intronic_lncRNA | 0.821703 | 0.336801 | 0.748215 | 0.456887 |
| LNC_005793 | XLOC_136265 | -- | intronic_lncRNA | 0.246456 | 0.566426 | 0.404475 | 0 |
| LNC_005792 | XLOC_136248 | -- | lincRNA | 0.69391 | 0.252023 | 0.397088 | 0.675044 |
| LNC_005795 | XLOC_136430 | -- | lincRNA | 6.213078 | 3.835278 | 2.587549 | 3.162557 |
| LNC_005794 | XLOC_136301 | -- | intronic_lncRNA | 0.720844 | 0.223796 | 0.437337 | 1.1197 |
| LNC_005797 | XLOC_136542 | -- | intronic_lncRNA | 0.462449 | 0.507731 | 0.540047 | 0.613216 |
| LNC_005796 | XLOC_136500 | -- | intronic_lncRNA | 0.667071 | 0 | 0 | 0.089662 |
| LNC_005799 | XLOC_136571 | -- | intronic_lncRNA | 0.515666 | 0 | 0.15152 | 0.206284 |
| LNC_005798 | XLOC_136551 | -- | intronic_lncRNA | 0.719186 | 0.551859 | 0.867333 | 0.871853 |
| LNC_006216 | XLOC_149015 | -- | intronic_lncRNA | 1.03509 | 0.517541 | 1.461901 | 1.658616 |
| LNC_001474 | XLOC_032053 | -- | intronic_lncRNA | 0.249506 | 0.595883 | 0.241291 | 0.209619 |
| LNC_001475 | XLOC_032228 | -- | intronic_lncRNA | 0.382422 | 0.473284 | 2.543337 | 2.579427 |
| LNC_001476 | XLOC_032274 | -- | intronic_lncRNA | 0 | 0 | 0.503397 | 1.231146 |
| LNC_001477 | XLOC_032325 | -- | intronic_lncRNA | 0.262938 | 0.013088 | 0.401464 | 0.008428 |
| LNC_001470 | XLOC_031942 | -- | intronic_lncRNA | 0.131291 | 0.34615 | 0.404925 | 0.385754 |
| LNC_001471 | XLOC_032011 | -- | intronic_lncRNA | 0 | 0 | 0.423165 | 0 |
| LNC_001472 | XLOC_032014 | -- | antisense_lncRNA | 0.045963 | 0.551668 | 0.45559 | 0.064301 |
| LNC_001473 | XLOC_032014 | -- | intronic_lncRNA | 0.482454 | 0.570551 | 2.63652 | 2.159178 |
| LNC_001478 | XLOC_032327 | -- | intronic_lncRNA | 0 | 0 | 0 | 0.548208 |
| LNC_001479 | XLOC_032337 | -- | intronic_lncRNA | 0.653309 | 0.765547 | 0.650279 | 0.371492 |
| LNC_004446 | XLOC_097427 | -- | lincRNA | 0.578932 | 0.70624 | 0.595097 | 0.601665 |
| LNC_004447 | XLOC_097427 | -- | lincRNA | 0.38291 | 0.346107 | 0.715334 | 0.570497 |
| LNC_004444 | XLOC_097392 | -- | lincRNA | 0 | 0 | 0 | 0.434844 |
| LNC_004445 | XLOC_097427 | -- | lincRNA | 0.263866 | 0 | 0.639519 | 0.411388 |
| LNC_004442 | XLOC_097322 | -- | intronic_lncRNA | 0.095037 | 0.526495 | 0.194319 | 0.07704 |
| LNC_004443 | XLOC_097361 | -- | lincRNA | 0.145618 | 0.346824 | 0.412493 | 0 |
| LNC_004440 | XLOC_097050 | -- | intronic_lncRNA | 0.756047 | 0.540243 | 0.53556 | 0.578627 |
| LNC_004441 | XLOC_097089 | -- | intronic_lncRNA | 0.452857 | 1.046149 | 0.439217 | 0.68699 |
| LNC_004448 | XLOC_097716 | -- | intronic_lncRNA | 0.126921 | 0 | 0.158622 | 0.512015 |
| LNC_004449 | XLOC_097718 | -- | intronic_lncRNA | 0 | 0.759816 | 0 | 0 |
| LNC_006178 | XLOC_147952 | -- | intronic_lncRNA | 0.107574 | 0.32591 | 0.603023 | 0.271347 |
| LNC_006179 | XLOC_147991 | -- | intronic_lncRNA | 0.670353 | 0.242685 | 2.516514 | 1.656245 |
| LNC_001788 | XLOC_039910 | -- | intronic_lncRNA | 0.090861 | 0.290546 | 0.400323 | 0.400254 |

| LNC_001789 | XLOC_039920 | -- | intronic_lncRNA | 0.053193 | 0.278733 | 0.458935 | 0.312025 |
| --- | --- | --- | --- | --- | --- | --- | --- |
| LNC_001786 | XLOC_039907 | -- | intronic_lncRNA | 0.2947 | 0.551324 | 0.486977 | 0.201118 |
| LNC_001787 | XLOC_039908 | -- | intronic_lncRNA | 0.066334 | 0.653145 | 0.752528 | 0.202379 |
| LNC_001784 | XLOC_039810 | -- | intronic_lncRNA | 0.647813 | 0.346265 | 0.770469 | 0.556782 |
| LNC_001785 | XLOC_039906 | -- | intronic_lncRNA | 0.175254 | 0.55654 | 0.231006 | 1.535157 |
| LNC_001782 | XLOC_039591 | -- | lincRNA | 0.777165 | 0.964837 | 0.596488 | 0.441653 |
| LNC_001783 | XLOC_039674 | -- | lincRNA | 0.58336 | 0.605763 | 0.323689 | 0.468708 |
| LNC_001780 | XLOC_039497 | -- | intronic_lncRNA | 1.650633 | 0.539967 | 0.18477 | 0.210928 |
| LNC_001781 | XLOC_039515 | -- | intronic_lncRNA | 0.553208 | 0.398656 | 0.233102 | 0.853646 |
| LNC_006217 | XLOC_149044 | -- | intronic_lncRNA | 0.480605 | 0.578589 | 0.540497 | 0.39551 |
| LNC_006369 | XLOC_152557 | -- | intronic_lncRNA | 3.23842 | 3.960974 | 1.844508 | 1.143879 |
| LNC_006368 | XLOC_152557 | -- | intronic_lncRNA | 1.077051 | 0 | 0 | 0 |
| LNC_000785 | XLOC_016532 | -- | lincRNA | 0.616421 | 0.487279 | 0.298126 | 0.478025 |
| LNC_000784 | XLOC_016524 | -- | intronic_lncRNA | 0.106888 | 1.279872 | 1.634162 | 0 |
| LNC_000787 | XLOC_016668 | -- | lincRNA | 0.468062 | 0.418419 | 0.393547 | 0.8191 |
| LNC_000786 | XLOC_016573 | -- | intronic_lncRNA | 0.772361 | 0.516335 | 1.074448 | 0.556085 |
| LNC_000781 | XLOC_016362 | -- | lincRNA | 0.631366 | 1.019382 | 0.394986 | 0.177891 |
| LNC_000780 | XLOC_016341 | -- | intronic_lncRNA | 3.445877 | 0.163368 | 0.364045 | 0.464216 |
| LNC_000783 | XLOC_016511 | -- | lincRNA | 0.653425 | 0.686399 | 0.510802 | 0.528412 |
| LNC_000782 | XLOC_016412 | -- | lincRNA | 0.047713 | 0.048898 | 0.398062 | 0 |
| LNC_000789 | XLOC_016826 | -- | intronic_lncRNA | 2.696773 | 3.212923 | 4.905869 | 6.2786 |
| LNC_000788 | XLOC_016759 | -- | intronic_lncRNA | 2.044551 | 1.13142 | 2.314166 | 1.766753 |
| LNC_000802 | XLOC_017036 | -- | intronic_lncRNA | 0.393429 | 0.558479 | 0.279064 | 0.843993 |
| LNC_000803 | XLOC_017042 | -- | intronic_lncRNA | 2.105511 | 1.782019 | 0.84823 | 2.127446 |
| LNC_000800 | XLOC_016993 | -- | intronic_lncRNA | 1.394747 | 0.843723 | 2.023406 | 1.644569 |
| LNC_000801 | XLOC_016994 | -- | intronic_lncRNA | 0 | 0.479333 | 0.309907 | 0.421116 |
| LNC_000806 | XLOC_017053 | -- | intronic_lncRNA | 0.137341 | 0.112101 | 0.416166 | 0.287824 |
| LNC_000807 | XLOC_017068 | -- | intronic_lncRNA | 1.403017 | 0.343883 | 0.852238 | 0.812301 |
| LNC_000804 | XLOC_017042 | -- | intronic_lncRNA | 0 | 0 | 0.801076 | 0.118571 |
| LNC_005159 | XLOC_117883 | -- | intronic_lncRNA | 0.486704 | 0.200569 | 0.484059 | 0.552916 |
| LNC_000438 | XLOC_007861 | -- | intronic_lncRNA | 0.174006 | 0.563822 | 0.473883 | 0.717259 |
| LNC_000437 | XLOC_007860 | -- | intronic_lncRNA | 0.398261 | 0.66123 | 0.161671 | 0.493598 |
| LNC_005156 | XLOC_117854 | -- | intronic_lncRNA | 0.388228 | 0.402708 | 0.690827 | 1.095225 |
| LNC_005155 | XLOC_117849 | -- | intronic_lncRNA | 0 | 0.466303 | 0 | 0.659813 |
| LNC_000434 | XLOC_007825 | -- | intronic_lncRNA | 0.1998 | 0.165904 | 0.812652 | 0.288712 |
| LNC_005153 | XLOC_117817 | -- | intronic_lncRNA | 0.337052 | 0.532536 | 0.005342 | 0.276466 |
| LNC_005152 | XLOC_117766 | -- | lincRNA | 0 | 0 | 0.606542 | 0.152002 |
| LNC_000431 | XLOC_007815 | -- | intronic_lncRNA | 0.20555 | 0.142126 | 0.199091 | 0.33213 |
| LNC_005150 | XLOC_117688 | -- | intronic_lncRNA | 0.116994 | 0.171728 | 0.971715 | 1.428251 |
| LNC_002462 | XLOC_057274 | -- | intronic_lncRNA | 1.177786 | 0.219858 | 1.779243 | 1.960673 |
| LNC_002463 | XLOC_057274 | -- | intronic_lncRNA | 0.030478 | 1.404129 | 0.009627 | 0.814351 |
| LNC_002460 | XLOC_057259 | -- | intronic_lncRNA | 0 | 0 | 0.648253 | 0.576426 |
| LNC_002461 | XLOC_057259 | -- | intronic_lncRNA | 1.216301 | 0.544215 | 0.953414 | 0.963646 |
| LNC_002466 | XLOC_057347 | -- | intronic_lncRNA | 0.697959 | 0.608883 | 0.434996 | 0.848771 |
| LNC_002467 | XLOC_057374 | -- | intronic_lncRNA | 2.137085 | 0.74618 | 2.346042 | 1.61572 |
| LNC_002464 | XLOC_057325 | -- | lincRNA | 0.524149 | 0 | 0.150152 | 0 |
| LNC_002465 | XLOC_057327 | -- | lincRNA | 0 | 0 | 0.501473 | 0 |
| LNC_002468 | XLOC_057380 | -- | intronic_lncRNA | 0.503958 | 0 | 0.71761 | 0.423971 |
| LNC_002469 | XLOC_057381 | -- | intronic_lncRNA | 0 | 0 | 0.653948 | 0.325447 |
| LNC_002459 | XLOC_057259 | -- | intronic_lncRNA | 0.383782 | 0.274205 | 0.806604 | 0.4912 |
| LNC_004208 | XLOC_090902 | -- | lincRNA | 0 | 0 | 0 | 0.552508 |
| LNC_004209 | XLOC_090921 | -- | intronic_lncRNA | 0.61704 | 0.319864 | 0.325993 | 0.834298 |
| LNC_004204 | XLOC_090698 | -- | intronic_lncRNA | 0.570877 | 0.231372 | 0.607142 | 0.159005 |
| LNC_004205 | XLOC_090702 | -- | intronic_lncRNA | 0.231784 | 0.682241 | 0.293082 | 0.221662 |
| LNC_004206 | XLOC_090708 | -- | intronic_lncRNA | 0 | 0 | 0.385405 | 0.626176 |
| LNC_004207 | XLOC_090709 | -- | intronic_lncRNA | 0.064876 | 0 | 0.155021 | 0.619976 |
| LNC_004200 | XLOC_090521 | -- | intronic_lncRNA | 0.646959 | 0 | 0 | 0.044913 |
| LNC_004201 | XLOC_090523 | -- | intronic_lncRNA | 0.368354 | 0.724693 | 0.979456 | 0.451879 |
| LNC_004202 | XLOC_090524 | -- | intronic_lncRNA | 0.116944 | 0.292926 | 0.494161 | 0.039531 |
| LNC_004203 | XLOC_090684 | -- | intronic_lncRNA | 0.199074 | 0.055616 | 0.120645 | 0.596422 |
| LNC_004149 | XLOC_089327 | -- | intronic_lncRNA | 0.317519 | 0.399036 | 0.743217 | 0.704349 |
| LNC_004148 | XLOC_089281 | -- | lincRNA | 0.641021 | 0.221927 | 0.955232 | 2.360688 |
| LNC_004143 | XLOC_089221 | -- | intronic_lncRNA | 0 | 0.195284 | 0 | 0.474375 |
| LNC_004142 | XLOC_089220 | -- | intronic_lncRNA | 0.135185 | 0 | 0.436942 | 0.573752 |
| LNC_004141 | XLOC_089203 | -- | intronic_lncRNA | 0.135158 | 0.214709 | 0.059586 | 0.514108 |
| LNC_004140 | XLOC_089203 | -- | antisense_lncRNA | 1.074147 | 0.519365 | 0 | 0 |
| LNC_004147 | XLOC_089281 | -- | lincRNA | 0 | 0.149047 | 0.904119 | 0.040126 |
| LNC_004146 | XLOC_089281 | -- | lincRNA | 0.079852 | 0.619415 | 0.010321 | 0.425123 |
| LNC_004145 | XLOC_089224 | -- | intronic_lncRNA | 0.462223 | 0 | 0.594432 | 0 |
| LNC_004144 | XLOC_089223 | -- | intronic_lncRNA | 0.365447 | 0.36676 | 0.086344 | 0.474236 |
| LNC_003498 | XLOC_075252 | -- | lincRNA | 2.007489 | 0.70954 | 0.64647 | 0.50511 |
| LNC_003499 | XLOC_075253 | -- | lincRNA | 1.19088 | 0.758615 | 0.411551 | 0.694632 |
| LNC_003490 | XLOC_075248 | -- | lincRNA | 1.223284 | 0.41263 | 0.234814 | 0.19844 |
| LNC_003491 | XLOC_075249 | -- | lincRNA | 4.855008 | 2.040604 | 0.886411 | 0.493107 |
| LNC_003492 | XLOC_075249 | -- | lincRNA | 5.258046 | 3.562917 | 2.013283 | 1.581745 |
| LNC_003493 | XLOC_075250 | -- | lincRNA | 3.266057 | 0.576088 | 0.412382 | 1.099526 |
| LNC_003494 | XLOC_075251 | -- | lincRNA | 1.731601 | 1.294472 | 0.831608 | 0.555158 |

| LNC_003495 | XLOC_075252 | -- | lincRNA | 7.483986 | 2.834622 | 2.591038 | 2.622469 |
| --- | --- | --- | --- | --- | --- | --- | --- |
| LNC_003496 | XLOC_075252 | -- | lincRNA | 8.08937 | 2.58479 | 1.878106 | 2.418247 |
| LNC_003497 | XLOC_075252 | -- | lincRNA | 1.21305 | 0.583597 | 0.44236 | 0.222282 |
| LNC_005638 | XLOC_131448 | -- | intronic_lncRNA | 1.333896 | 1.716218 | 1.316202 | 1.742097 |
| LNC_005639 | XLOC_131457 | -- | lincRNA | 0.784236 | 1.016601 | 0.859326 | 1.109396 |
| LNC_005632 | XLOC_131290 | -- | intronic_lncRNA | 0.367705 | 0.576526 | 1.460007 | 0.984509 |
| LNC_005633 | XLOC_131300 | -- | lincRNA | 0 | 0.034021 | 0 | 0.589496 |
| LNC_005630 | XLOC_131263 | -- | intronic_lncRNA | 0.448238 | 0.497848 | 0 | 0.201559 |
| LNC_005631 | XLOC_131286 | -- | intronic_lncRNA | 0.988628 | 0 | 0.114285 | 0.228531 |
| LNC_005636 | XLOC_131413 | -- | intronic_lncRNA | 1.841012 | 2.810907 | 1.588215 | 1.789441 |
| LNC_005637 | XLOC_131426 | -- | intronic_lncRNA | 0.705255 | 0.496539 | 0.9928 | 0.827527 |
| LNC_005634 | XLOC_131303 | -- | lincRNA | 0.214897 | 0.119893 | 0.184101 | 0.503776 |
| LNC_005635 | XLOC_131412 | -- | intronic_lncRNA | 3.51525 | 2.280368 | 2.904057 | 3.153181 |
| LNC_006332 | XLOC_152096 | -- | intronic_lncRNA | 1.155626 | 0.299221 | 0.363456 | 0.263408 |
| LNC_006333 | XLOC_152099 | -- | intronic_lncRNA | 1.061728 | 0 | 0 | 0 |
| LNC_001643 | XLOC_035831 | -- | lincRNA | 0.294299 | 0.705742 | 0.326152 | 0.570555 |
| LNC_001642 | XLOC_035798 | -- | lincRNA | 0.57657 | 0.053118 | 0 | 0 |
| LNC_001641 | XLOC_035795 | -- | intronic_lncRNA | 0 | 0 | 0.166416 | 0.558722 |
| LNC_001640 | XLOC_035794 | -- | intronic_lncRNA | 0 | 0.121497 | 0 | 0.86171 |
| LNC_001647 | XLOC_036080 | -- | intronic_lncRNA | 0.379342 | 0.109242 | 0.477519 | 0.286849 |
| LNC_001646 | XLOC_036006 | -- | lincRNA | 0.375501 | 0.60844 | 0.324279 | 0.284104 |
| LNC_001645 | XLOC_035955 | -- | lincRNA | 0.437537 | 0.496516 | 0.270704 | 0.397326 |
| LNC_001644 | XLOC_035934 | -- | intronic_lncRNA | 0 | 0.308121 | 0.48276 | 0.151652 |
| LNC_001649 | XLOC_036083 | -- | intronic_lncRNA | 0.489907 | 0.547248 | 0.673513 | 0.441441 |
| LNC_001648 | XLOC_036082 | -- | intronic_lncRNA | 0.521831 | 0.105961 | 0.519086 | 0.179837 |
| LNC_004925 | XLOC_111711 | -- | intronic_lncRNA | 0.903277 | 1.162803 | 0.9611 | 0.86869 |
| LNC_004924 | XLOC_111710 | -- | intronic_lncRNA | 0.071446 | 1.756868 | 0.785897 | 0.493995 |
| LNC_004927 | XLOC_111770 | -- | intronic_lncRNA | 0.666915 | 0.802628 | 0.34345 | 0.949683 |
| LNC_004926 | XLOC_111770 | -- | intronic_lncRNA | 0 | 0 | 0.516063 | 0.005629 |
| LNC_004921 | XLOC_111648 | -- | intronic_lncRNA | 0.275403 | 0.110764 | 0.373532 | 0.936318 |
| LNC_004920 | XLOC_111542 | -- | lincRNA | 0.290002 | 0 | 0.149525 | 1.096329 |
| LNC_004923 | XLOC_111710 | -- | intronic_lncRNA | 0.898409 | 0.152151 | 0.03836 | 0.088715 |
| LNC_004922 | XLOC_111662 | -- | intronic_lncRNA | 1.674516 | 1.019206 | 2.513108 | 2.127838 |
| LNC_004929 | XLOC_111869 | -- | lincRNA | 0.460909 | 0.195935 | 1.017618 | 0.946303 |
| LNC_004928 | XLOC_111775 | -- | lincRNA | 0.556004 | 0.496373 | 0.607059 | 0.446081 |
| LNC_001250 | XLOC_026916 | -- | intronic_lncRNA | 0 | 0.75874 | 0.893844 | 0 |
| LNC_001251 | XLOC_026963 | -- | intronic_lncRNA | 0.088685 | 0.126421 | 0.054581 | 0.710305 |
| LNC_001252 | XLOC_026965 | -- | intronic_lncRNA | 0.418749 | 0.440499 | 0.587141 | 0.19568 |
| LNC_001253 | XLOC_026967 | -- | intronic_lncRNA | 0.100232 | 0.150387 | 0.486358 | 0.608451 |
| LNC_001254 | XLOC_026968 | -- | intronic_lncRNA | 0.45517 | 0.25855 | 0.545403 | 0.288842 |
| LNC_001255 | XLOC_027043 | -- | intronic_lncRNA | 1.078813 | 0.565382 | 0.656837 | 0.57846 |
| LNC_001256 | XLOC_027043 | -- | intronic_lncRNA | 0.587472 | 1.026771 | 0.200593 | 0 |
| LNC_001257 | XLOC_027064 | -- | intronic_lncRNA | 0.544413 | 1.309261 | 0.176719 | 0.726901 |
| LNC_001258 | XLOC_027065 | -- | intronic_lncRNA | 0.027827 | 0.576134 | 0.364395 | 0.756515 |
| LNC_001259 | XLOC_027065 | -- | intronic_lncRNA | 0.672641 | 0 | 0 | 0.01077 |
| LNC_000233 | XLOC_003247 | -- | intronic_lncRNA | 0.440253 | 0.809293 | 0 | 0.207512 |
| LNC_002699 | XLOC_062717 | -- | intronic_lncRNA | 0.347043 | 0.192754 | 0.284164 | 0.420566 |
| LNC_002698 | XLOC_062713 | -- | intronic_lncRNA | 0.526536 | 0.306139 | 0.321651 | 0.20169 |
| LNC_002691 | XLOC_062672 | -- | intronic_lncRNA | 0.450413 | 0.049546 | 0.40332 | 0.266972 |
| LNC_002690 | XLOC_062665 | -- | intronic_lncRNA | 0.104999 | 0 | 0.416905 | 0.301141 |
| LNC_002693 | XLOC_062690 | -- | intronic_lncRNA | 0 | 0 | 0 | 0.423589 |
| LNC_002692 | XLOC_062673 | -- | intronic_lncRNA | 0.128297 | 0.107208 | 0.409422 | 0.368356 |
| LNC_002695 | XLOC_062701 | -- | intronic_lncRNA | 0.367868 | 0.120985 | 0.188656 | 0.578517 |
| LNC_002694 | XLOC_062699 | -- | intronic_lncRNA | 0.970986 | 0.646232 | 0.209201 | 0.7849 |
| LNC_002697 | XLOC_062703 | -- | intronic_lncRNA | 1.231173 | 0.362786 | 1.333109 | 0.562458 |
| LNC_002696 | XLOC_062703 | -- | intronic_lncRNA | 0.014292 | 0 | 0.238838 | 0.890535 |
| LNC_005401 | XLOC_124577 | -- | intronic_lncRNA | 0.416795 | 1.036963 | 1.421721 | 2.107672 |
| LNC_005400 | XLOC_124551 | -- | intronic_lncRNA | 0.40735 | 0.182086 | 0.725826 | 0.413306 |
| LNC_005403 | XLOC_124613 | -- | intronic_lncRNA | 0.835032 | 0.558915 | 0.515197 | 0.648039 |
| LNC_005402 | XLOC_124586 | -- | intronic_lncRNA | 0.260427 | 0 | 0.257932 | 0.438908 |
| LNC_000125 | XLOC_001046 | -- | intronic_lncRNA | 0.158422 | 0.124462 | 0.603772 | 0.100779 |
| LNC_005404 | XLOC_124655 | -- | intronic_lncRNA | 0.281387 | 0 | 0.588292 | 0.204313 |
| LNC_005407 | XLOC_124702 | -- | lincRNA | 0 | 0 | 0.605509 | 0 |
| LNC_005406 | XLOC_124671 | -- | intronic_lncRNA | 0.577049 | 0.446383 | 0.869934 | 0.61213 |
| LNC_000129 | XLOC_001305 | -- | intronic_lncRNA | 0.127958 | 0 | 0.257818 | 0.664573 |
| LNC_005408 | XLOC_124764 | -- | intronic_lncRNA | 0.461395 | 1.424577 | 1.400607 | 0.999138 |
| LNC_005988 | XLOC_142463 | -- | lincRNA | 0.686479 | 0.332488 | 0.418706 | 0.391642 |
| LNC_005989 | XLOC_142499 | -- | intronic_lncRNA | 0 | 0.427033 | 0.560661 | 0.138766 |
| LNC_005986 | XLOC_142445 | -- | intronic_lncRNA | 0.497777 | 0 | 0.362633 | 0.041385 |
| LNC_005987 | XLOC_142446 | -- | intronic_lncRNA | 0.652138 | 0 | 0.073902 | 0.467295 |
| LNC_001418 | XLOC_030751 | -- | intronic_lncRNA | 0.81668 | 0.169505 | 0.400799 | 0.273952 |
| LNC_001419 | XLOC_030767 | -- | intronic_lncRNA | 1.692686 | 1.21737 | 1.81059 | 2.127189 |
| LNC_001416 | XLOC_030735 | -- | intronic_lncRNA | 0.350118 | 0.314212 | 0.583697 | 0.651513 |
| LNC_001417 | XLOC_030750 | -- | intronic_lncRNA | 0.616329 | 0.45432 | 0.429455 | 0.591662 |
| LNC_001414 | XLOC_030723 | -- | antisense_lncRNA | 1.109998 | 0.243009 | 0.239872 | 0.179589 |
| LNC_001415 | XLOC_030732 | -- | intronic_lncRNA | 0 | 0.890525 | 0.240928 | 0.216971 |
| LNC_001412 | XLOC_030721 | -- | intronic_lncRNA | 0.987323 | 0.482182 | 0.660108 | 0.674249 |

| LNC_001413 | XLOC_030723 | -- | intronic_lncRNA | 2.368599 | 2.888924 | 1.878345 | 2.33619 |
| --- | --- | --- | --- | --- | --- | --- | --- |
| LNC_001410 | XLOC_030672 | -- | intronic_lncRNA | 1.372875 | 1.075809 | 1.372104 | 1.140324 |
| LNC_001411 | XLOC_030672 | -- | intronic_lncRNA | 0.909153 | 0.945169 | 1.102723 | 1.158145 |
| LNC_004468 | XLOC_098229 | -- | intronic_lncRNA | 0.145324 | 0 | 0.870339 | 0 |
| LNC_004469 | XLOC_098236 | -- | intronic_lncRNA | 0.78325 | 0.767931 | 0.984271 | 0.893164 |
| LNC_004460 | XLOC_098027 | -- | intronic_lncRNA | 0 | 0 | 0.50647 | 0 |
| LNC_004461 | XLOC_098028 | -- | intronic_lncRNA | 0.521342 | 0.211379 | 0 | 0 |
| LNC_004462 | XLOC_098029 | -- | intronic_lncRNA | 0.859443 | 0.198009 | 0.199122 | 0.126245 |
| LNC_004463 | XLOC_098109 | -- | intronic_lncRNA | 0.599735 | 0.671746 | 0.753816 | 0.602605 |
| LNC_004464 | XLOC_098109 | -- | intronic_lncRNA | 0.677682 | 0.117808 | 0.042157 | 0.041601 |
| LNC_004465 | XLOC_098110 | -- | intronic_lncRNA | 0.509301 | 0.318005 | 0.430929 | 0 |
| LNC_004466 | XLOC_098227 | -- | intronic_lncRNA | 0.748323 | 0 | 0.603958 | 0.129613 |
| LNC_004467 | XLOC_098228 | -- | intronic_lncRNA | 0.754448 | 0.353755 | 0.374771 | 0.373457 |
| LNC_006192 | XLOC_148306 | -- | antisense_lncRNA | 0 | 0.314713 | 0.305 | 0.203524 |
| LNC_006193 | XLOC_148308 | -- | lincRNA | 0.339749 | 0.317222 | 0.621761 | 0.251951 |
| LNC_006190 | XLOC_148229 | -- | lincRNA | 1.277152 | 1.080037 | 0 | 0.526521 |
| LNC_006191 | XLOC_148240 | -- | lincRNA | 0.028872 | 0 | 0.346216 | 0 |
| LNC_006196 | XLOC_148354 | -- | antisense_lncRNA | 2.06518 | 1.343738 | 0.738523 | 0.801546 |
| LNC_006197 | XLOC_148582 | -- | lincRNA | 0 | 0 | 0.675846 | 0.237884 |
| LNC_006194 | XLOC_148316 | -- | lincRNA | 0 | 0 | 0.555184 | 0 |
| LNC_006195 | XLOC_148353 | -- | intronic_lncRNA | 0.310076 | 0.55868 | 0.477824 | 0.611087 |
| LNC_006198 | XLOC_148613 | -- | intronic_lncRNA | 0.636131 | 0.180533 | 0.968823 | 0.330828 |
| LNC_006199 | XLOC_148618 | -- | intronic_lncRNA | 0.087194 | 0.724992 | 0 | 0.353691 |
| LNC_005157 | XLOC_117857 | -- | lincRNA | 0.973843 | 2.364121 | 1.298118 | 2.376632 |
| LNC_002509 | XLOC_058130 | -- | intronic_lncRNA | 0.623736 | 0.37373 | 1.285272 | 0.427815 |
| LNC_002508 | XLOC_058115 | -- | lincRNA | 0.677533 | 0 | 0.070979 | 0.078272 |
| LNC_002501 | XLOC_057971 | -- | intronic_lncRNA | 5.172326 | 6.158075 | 5.11161 | 4.694644 |
| LNC_002500 | XLOC_057943 | -- | intronic_lncRNA | 0.356409 | 6.082651 | 1.756769 | 1.054932 |
| LNC_002503 | XLOC_058019 | -- | intronic_lncRNA | 0.028581 | 1.304714 | 1.598808 | 2.068791 |
| LNC_002502 | XLOC_058008 | -- | intronic_lncRNA | 0.742937 | 0.420757 | 1.07884 | 0.928754 |
| LNC_002505 | XLOC_058030 | -- | intronic_lncRNA | 0.859308 | 0.969888 | 0.137341 | 0.235601 |
| LNC_002504 | XLOC_058019 | -- | intronic_lncRNA | 6.581144 | 5.219961 | 3.583615 | 3.049559 |
| LNC_002507 | XLOC_058105 | -- | intronic_lncRNA | 0.135817 | 0 | 0.246563 | 0.548635 |
| LNC_002506 | XLOC_058033 | -- | intronic_lncRNA | 0 | 0 | 0.206922 | 0.413772 |
| LNC_003119 | XLOC_071604 | -- | intronic_lncRNA | 0.479264 | 0.198148 | 0.43018 | 0.685465 |
| LNC_003118 | XLOC_071603 | -- | intronic_lncRNA | 0.608189 | 0.137602 | 0.250736 | 0.389967 |
| LNC_003111 | XLOC_071533 | -- | intronic_lncRNA | 0.717382 | 1.082927 | 0.647296 | 0.496168 |
| LNC_003110 | XLOC_071493 | -- | intronic_lncRNA | 3.733017 | 1.415331 | 4.135672 | 2.068298 |
| LNC_003113 | XLOC_071538 | -- | intronic_lncRNA | 0.584259 | 0.193791 | 1.094561 | 0.723739 |
| LNC_003112 | XLOC_071535 | -- | intronic_lncRNA | 0.604581 | 0 | 0 | 0.806235 |
| LNC_003115 | XLOC_071557 | -- | intronic_lncRNA | 0.793329 | 0.254187 | 0.621967 | 0.657141 |
| LNC_003114 | XLOC_071538 | -- | intronic_lncRNA | 0.761399 | 0.420809 | 0.615562 | 0.669468 |
| LNC_003117 | XLOC_071594 | -- | intronic_lncRNA | 0.895137 | 0.492354 | 0.627878 | 1.406237 |
| LNC_003116 | XLOC_071577 | -- | intronic_lncRNA | 1.332538 | 0.741999 | 0.882917 | 0.555199 |
| LNC_000244 | XLOC_003399 | -- | antisense_lncRNA | 0.402857 | 0.44162 | 0.643162 | 0.531415 |
| LNC_005365 | XLOC_123272 | -- | intronic_lncRNA | 0.285304 | 0.747624 | 0 | 0.465594 |
| LNC_000246 | XLOC_003465 | -- | intronic_lncRNA | 0.728741 | 0.569578 | 0.17344 | 0.474375 |
| LNC_000247 | XLOC_003493 | -- | intronic_lncRNA | 0.450449 | 0.296941 | 0.134863 | 1.059708 |
| LNC_005360 | XLOC_123204 | -- | intronic_lncRNA | 0.617908 | 0 | 0.761716 | 0.317774 |
| LNC_000241 | XLOC_003348 | -- | intronic_lncRNA | 1.118098 | 0 | 0 | 0 |
| LNC_002444 | XLOC_056754 | -- | intronic_lncRNA | 2.985407 | 4.067036 | 2.724638 | 4.092072 |
| LNC_002445 | XLOC_056773 | -- | intronic_lncRNA | 0 | 0.180065 | 0.340166 | 0 |
| LNC_002446 | XLOC_056778 | -- | intronic_lncRNA | 0.432891 | 0.275484 | 0.463338 | 0.476005 |
| LNC_002447 | XLOC_056781 | -- | intronic_lncRNA | 0.378466 | 0 | 0 | 0.411958 |
| LNC_002440 | XLOC_056499 | -- | intronic_lncRNA | 0.359471 | 0.777196 | 1.086292 | 0.394 |
| LNC_002441 | XLOC_056509 | -- | intronic_lncRNA | 0.534435 | 0.406611 | 0.531985 | 0.549505 |
| LNC_002442 | XLOC_056551 | -- | lincRNA | 0 | 0.645863 | 0 | 0 |
| LNC_002443 | XLOC_056724 | -- | intronic_lncRNA | 1.206945 | 1.005034 | 0.695082 | 0.587302 |
| LNC_005363 | XLOC_123254 | -- | lincRNA | 0.342505 | 0 | 0.684384 | 0.375205 |
| LNC_002448 | XLOC_056784 | -- | intronic_lncRNA | 0.492028 | 0.441833 | 0.326205 | 0.374229 |
| LNC_002449 | XLOC_056847 | -- | intronic_lncRNA | 0.543267 | 0.903416 | 0.146328 | 0.138988 |
| LNC_002458 | XLOC_057255 | -- | lincRNA | 0.223634 | 0.229246 | 0.506844 | 0.740645 |
| LNC_006260 | XLOC_150228 | -- | intronic_lncRNA | 0.793133 | 1.432024 | 0.173375 | 0.052143 |
| LNC_006261 | XLOC_150284 | -- | intronic_lncRNA | 0 | 0.571422 | 0 | 0 |
| LNC_000248 | XLOC_003506 | -- | intronic_lncRNA | 0.133441 | 0 | 0.501418 | 0.286555 |
| LNC_006262 | XLOC_150287 | -- | intronic_lncRNA | 0.722926 | 0.465489 | 0.42403 | 0.585576 |
| LNC_005369 | XLOC_123467 | -- | lincRNA | 0.110327 | 0.601474 | 0.069587 | 0 |
| LNC_006263 | XLOC_150288 | -- | intronic_lncRNA | 0.518078 | 0.021689 | 0.401875 | 0.604072 |
| LNC_002998 | XLOC_068534 | -- | intronic_lncRNA | 0.912526 | 0 | 0.417315 | 0.232496 |
| LNC_002999 | XLOC_068572 | -- | intronic_lncRNA | 0.315916 | 0.589842 | 0.792793 | 0.512816 |
| LNC_002996 | XLOC_068526 | -- | intronic_lncRNA | 0.625708 | 0.474066 | 0.614096 | 1.157206 |
| LNC_002997 | XLOC_068528 | -- | intronic_lncRNA | 0 | 0.473529 | 0.165469 | 0.238281 |
| LNC_002994 | XLOC_068461 | -- | intronic_lncRNA | 1.874022 | 2.019198 | 0.829226 | 1.610438 |
| LNC_002995 | XLOC_068486 | -- | lincRNA | 0.29776 | 0.659083 | 0 | 0 |
| LNC_002992 | XLOC_068456 | -- | intronic_lncRNA | 0 | 0.247732 | 0.610017 | 0 |
| LNC_002993 | XLOC_068458 | -- | intronic_lncRNA | 0.664498 | 0.42232 | 0.559053 | 0.381031 |
| LNC_002990 | XLOC_068331 | -- | intronic_lncRNA | 0.533682 | 0 | 0 | 0.149865 |

| LNC_002991 | XLOC_068405 | -- | intronic_lncRNA | 0.371346 | 0.185566 | 0.653892 | 0.272408 |
| --- | --- | --- | --- | --- | --- | --- | --- |
| LNC_006266 | XLOC_150362 | -- | intronic_lncRNA | 0.307581 | 0 | 0.827688 | 0.870991 |
| LNC_002457 | XLOC_057227 | -- | intronic_lncRNA | 0.206082 | 0.207697 | 0.195172 | 0.502566 |
| LNC_006267 | XLOC_150366 | -- | intronic_lncRNA | 0 | 0 | 0 | 0.590394 |
| LNC_002456 | XLOC_057201 | -- | lincRNA | 0.318132 | 0.3298 | 0.587587 | 0.100031 |
| LNC_006268 | XLOC_150373 | -- | intronic_lncRNA | 0.175067 | 0.971973 | 0.930641 | 0.700016 |
| LNC_006269 | XLOC_150404 | -- | intronic_lncRNA | 0.495916 | 0.315672 | 0.544549 | 0.545221 |
| LNC_004266 | XLOC_092823 | -- | intronic_lncRNA | 0.134145 | 0.223074 | 0.289054 | 0.137518 |
| LNC_004267 | XLOC_092826 | -- | intronic_lncRNA | 0.754502 | 0.792433 | 0.271496 | 0.136521 |
| LNC_004264 | XLOC_092813 | -- | intronic_lncRNA | 0.697142 | 0.352262 | 0.961156 | 0.802357 |
| LNC_004265 | XLOC_092815 | -- | intronic_lncRNA | 0 | 1.326548 | 0.116898 | 0 |
| LNC_004262 | XLOC_092805 | -- | intronic_lncRNA | 0.234079 | 0.155703 | 0.490098 | 0.313161 |
| LNC_004263 | XLOC_092806 | -- | intronic_lncRNA | 0 | 0.472235 | 0.465046 | 0.210903 |
| LNC_004260 | XLOC_092803 | -- | intronic_lncRNA | 0.765323 | 0.394051 | 0.735391 | 0.645682 |
| LNC_004261 | XLOC_092804 | -- | intronic_lncRNA | 0.177224 | 0.429985 | 0.57282 | 0.184403 |
| LNC_004268 | XLOC_092827 | -- | intronic_lncRNA | 0.504321 | 0.247737 | 0.428961 | 0 |
| LNC_004269 | XLOC_092828 | -- | intronic_lncRNA | 0.722458 | 0.430849 | 0.522313 | 0.632913 |
| LNC_004129 | XLOC_088895 | -- | lincRNA | 0.112985 | 0 | 0.467044 | 0.15432 |
| LNC_004128 | XLOC_088845 | -- | intronic_lncRNA | 0.654142 | 0.345399 | 0.508898 | 0.762876 |
| LNC_004125 | XLOC_088822 | -- | intronic_lncRNA | 0 | 0 | 0.345139 | 0 |
| LNC_004124 | XLOC_088822 | -- | intronic_lncRNA | 0.560235 | 0.182159 | 0.15042 | 0.648595 |
| LNC_004127 | XLOC_088844 | -- | intronic_lncRNA | 0.845078 | 0.121847 | 0.394717 | 0.199854 |
| LNC_004126 | XLOC_088842 | -- | intronic_lncRNA | 0.292176 | 0.130629 | 0.725551 | 0.583514 |
| LNC_004121 | XLOC_088782 | -- | intronic_lncRNA | 0.163517 | 0.524881 | 0.45469 | 0.169901 |
| LNC_004120 | XLOC_088780 | -- | intronic_lncRNA | 0.255383 | 0 | 0.436266 | 0.363492 |
| LNC_004123 | XLOC_088819 | -- | intronic_lncRNA | 0.275496 | 0 | 0.222613 | 0.445149 |
| LNC_004122 | XLOC_088782 | -- | intronic_lncRNA | 0.455868 | 0 | 0.239336 | 0.399946 |
| LNC_004697 | XLOC_104378 | -- | intronic_lncRNA | 0.125564 | 0.393148 | 0.539015 | 0.513579 |
| LNC_004696 | XLOC_104359 | -- | lincRNA | 0.819099 | 0.362695 | 0.406416 | 0.583482 |
| LNC_004695 | XLOC_104358 | -- | lincRNA | 1.143987 | 1.432823 | 3.375625 | 2.419099 |
| LNC_004694 | XLOC_104297 | -- | intronic_lncRNA | 0.593194 | 0 | 0.584852 | 0.332587 |
| LNC_004693 | XLOC_104297 | -- | intronic_lncRNA | 0 | 0 | 0.592337 | 0.308655 |
| LNC_004692 | XLOC_104294 | -- | intronic_lncRNA | 0.15335 | 0.093658 | 0.761522 | 0.519531 |
| LNC_004691 | XLOC_104287 | -- | intronic_lncRNA | 0.275837 | 0.208654 | 0.469135 | 0.409537 |
| LNC_004690 | XLOC_104281 | -- | antisense_lncRNA | 0.444416 | 0 | 0.525414 | 0.454791 |
| LNC_004699 | XLOC_104445 | -- | intronic_lncRNA | 0.170136 | 0.198046 | 0.246542 | 0.366542 |
| LNC_004698 | XLOC_104378 | -- | intronic_lncRNA | 0.19591 | 0.240399 | 0.048076 | 0.548873 |
| LNC_004760 | XLOC_106492 | -- | intronic_lncRNA | 0.207691 | 0.237793 | 0.482091 | 0.530224 |
| LNC_003472 | XLOC_075243 | -- | lincRNA | 1.726374 | 1.372156 | 0.515138 | 0.643695 |
| LNC_003473 | XLOC_075244 | -- | lincRNA | 1.131462 | 1.242801 | 0.589401 | 0.527766 |
| LNC_003470 | XLOC_075241 | -- | lincRNA | 0.004946 | 0.546511 | 0 | 0.735239 |
| LNC_003471 | XLOC_075242 | -- | lincRNA | 0.806541 | 0.201259 | 0.1136 | 0.702225 |
| LNC_003476 | XLOC_075244 | -- | lincRNA | 0.484239 | 0.164177 | 0.290895 | 0.649716 |
| LNC_003477 | XLOC_075245 | -- | lincRNA | 5.974998 | 2.984548 | 2.681794 | 0 |
| LNC_003474 | XLOC_075244 | -- | lincRNA | 1.463351 | 0.084629 | 0 | 0.15878 |
| LNC_003475 | XLOC_075244 | -- | lincRNA | 2.201981 | 0.681742 | 0.717347 | 0.542918 |
| LNC_003478 | XLOC_075245 | -- | lincRNA | 2.414725 | 0.741046 | 0.465067 | 1.332627 |
| LNC_003479 | XLOC_075245 | -- | lincRNA | 5.556517 | 2.336977 | 2.071454 | 1.628288 |
| LNC_005618 | XLOC_131152 | -- | lincRNA | 1.604548 | 3.826558 | 2.388489 | 3.576978 |
| LNC_005619 | XLOC_131162 | -- | intronic_lncRNA | 0.901664 | 0.128459 | 0.463471 | 0.691185 |
| LNC_005614 | XLOC_131034 | -- | lincRNA | 0.20635 | 0.543065 | 0.197766 | 0 |
| LNC_005615 | XLOC_131091 | -- | intronic_lncRNA | 0.210819 | 0.083653 | 0.632456 | 0.237745 |
| LNC_005616 | XLOC_131093 | -- | intronic_lncRNA | 0.19796 | 0.086502 | 0.443909 | 0.465896 |
| LNC_005617 | XLOC_131098 | -- | intronic_lncRNA | 0.338511 | 0.572464 | 0.58186 | 0.295997 |
| LNC_005610 | XLOC_130869 | -- | lincRNA | 0.44628 | 0.666103 | 2.587919 | 1.727831 |
| LNC_005611 | XLOC_130926 | -- | intronic_lncRNA | 0.67099 | 0.162588 | 0.342188 | 1.370027 |
| LNC_005612 | XLOC_130986 | -- | lincRNA | 0.932329 | 0.365392 | 0.424969 | 0.308421 |
| LNC_005613 | XLOC_130991 | -- | lincRNA | 0.713522 | 0.488411 | 0.430963 | 0.303195 |
| LNC_000258 | XLOC_003710 | -- | intronic_lncRNA | 0.955374 | 0.503422 | 0.411127 | 0.8715 |
| LNC_000578 | XLOC_010817 | -- | intronic_lncRNA | 0.723626 | 0 | 0 | 0 |
| LNC_000579 | XLOC_010865 | -- | intronic_lncRNA | 0.709177 | 0.60387 | 0.134539 | 0.167677 |
| LNC_001625 | XLOC_035518 | -- | intronic_lncRNA | 0.564978 | 0.63018 | 1.169677 | 0.442206 |
| LNC_001624 | XLOC_035515 | -- | intronic_lncRNA | 7.735806 | 5.416904 | 8.912549 | 8.512494 |
| LNC_001627 | XLOC_035563 | -- | intronic_lncRNA | 0.449086 | 0.671318 | 0.327127 | 0.273286 |
| LNC_001626 | XLOC_035544 | -- | intronic_lncRNA | 0.831448 | 0.110868 | 0.150254 | 0.349118 |
| LNC_001621 | XLOC_035462 | -- | intronic_lncRNA | 0.713193 | 0.118034 | 0.200191 | 0.395205 |
| LNC_001620 | XLOC_035461 | -- | intronic_lncRNA | 0.654447 | 0.64908 | 0.25674 | 0 |
| LNC_001623 | XLOC_035507 | -- | lincRNA | 0.433032 | 0.898811 | 0 | 0 |
| LNC_001622 | XLOC_035463 | -- | intronic_lncRNA | 1.109709 | 0.565641 | 0.408862 | 0.371536 |
| LNC_001629 | XLOC_035585 | -- | intronic_lncRNA | 0.611082 | 1.348854 | 1.815985 | 1.195782 |
| LNC_001628 | XLOC_035568 | -- | intronic_lncRNA | 0.467417 | 0.274325 | 0.416045 | 1.205484 |
| LNC_000572 | XLOC_010597 | -- | intronic_lncRNA | 0 | 0.23205 | 0.931276 | 0.93329 |
| LNC_000573 | XLOC_010624 | -- | intronic_lncRNA | 0.50485 | 0 | 0.352285 | 0.644521 |
| LNC_000570 | XLOC_010595 | -- | intronic_lncRNA | 0.023056 | 0.574719 | 0.85496 | 1.547799 |
| LNC_000571 | XLOC_010595 | -- | intronic_lncRNA | 1.348882 | 2.347955 | 3.180716 | 3.467062 |
| LNC_004909 | XLOC_111496 | -- | lincRNA | 1.561416 | 0 | 1.218551 | 1.455954 |
| LNC_004908 | XLOC_111486 | -- | lincRNA | 0 | 0.515237 | 0.213132 | 0.257804 |

| LNC_004907 | XLOC_111485 | -- | lincRNA | 0 | 0.193331 | 0 | 0.397381 |
| --- | --- | --- | --- | --- | --- | --- | --- |
| LNC_004906 | XLOC_111482 | -- | lincRNA | 0.585211 | 0.317493 | 0 | 0.282886 |
| LNC_004905 | XLOC_111481 | -- | lincRNA | 1.235223 | 1.539842 | 0.193529 | 0 |
| LNC_004904 | XLOC_111473 | -- | lincRNA | 1.0704 | 0.330484 | 0 | 0.985432 |
| LNC_004903 | XLOC_111472 | -- | lincRNA | 0.576077 | 0.060104 | 0 | 0.246412 |
| LNC_004902 | XLOC_111469 | -- | lincRNA | 0.908382 | 0.85321 | 0.265875 | 2.037729 |
| LNC_004901 | XLOC_111454 | -- | intronic_lncRNA | 1.334251 | 1.080741 | 1.989922 | 1.3509 |
| LNC_004900 | XLOC_111355 | -- | intronic_lncRNA | 0.580484 | 0.485152 | 0.666329 | 0.923073 |
| LNC_000574 | XLOC_010641 | -- | intronic_lncRNA | 0 | 0 | 0.553125 | 0 |
| LNC_000575 | XLOC_010755 | -- | intronic_lncRNA | 0.158552 | 0.229436 | 0.407628 | 0.249551 |
| LNC_005058 | XLOC_115380 | -- | intronic_lncRNA | 1.453528 | 0.498209 | 0.517757 | 0.15308 |
| LNC_005059 | XLOC_115380 | -- | intronic_lncRNA | 0.74206 | 1.058575 | 2.765232 | 2.685968 |
| LNC_005052 | XLOC_115313 | -- | lincRNA | 1.089327 | 0.29123 | 0.008623 | 0.011864 |
| LNC_005053 | XLOC_115338 | -- | intronic_lncRNA | 0.727897 | 0 | 0.139915 | 0.383426 |
| LNC_005050 | XLOC_115279 | -- | intronic_lncRNA | 0.091015 | 0.218547 | 0.312381 | 0.574645 |
| LNC_005051 | XLOC_115279 | -- | intronic_lncRNA | 0.497709 | 0.228893 | 0.938468 | 0.609373 |
| LNC_005056 | XLOC_115355 | -- | intronic_lncRNA | 0.583842 | 0.1619 | 0.344685 | 0.379133 |
| LNC_005057 | XLOC_115356 | -- | intronic_lncRNA | 0.545123 | 0 | 0.127095 | 0.097729 |
| LNC_005054 | XLOC_115343 | -- | intronic_lncRNA | 0.910024 | 0.620801 | 0.996766 | 1.411109 |
| LNC_005055 | XLOC_115350 | -- | intronic_lncRNA | 0.830132 | 0.49591 | 0.410391 | 0.451891 |
