# Supplementary material for: Identification of Novel Multi-Omics Expression Landscapes and Meta-Analysis of Landscape-Based Competitive Endogenous RNA Networks in ALDH+ Lung Adenocarcinoma Stem Cells
Source: Biomed Res Int. 2022 Aug 31;2022:9545609. doi: 10.1155/2022/9545609 (PMC9453044; doi:10.1155/2022/9545609)
Supplement: Supplementary Materials — The supporting information includes the following: Figure S1. First base bias and position base bias of novel miRNAs; Table S1. Detailed information of miRNAs; Table S2. Novel lncRNAs; Table S3. Novel miRNAs; Table S4. Novel circRNAs; Table S5. Information on ceRNA-based molecular interactions and pairs. [file 9545609.f1.zip › Table_S3_Novel_miRNAs.doc]

sRNA/

LSC1-m	LSC2-m	LTC1-m	LTC2-m	LSC1-m.tpm	LSC2-m.tpm	LTC1-m.tpm	LTC2-m.tpm

readcount

| novel_142 | 0 | 96 | 0 | 0 | 0 | 24.94202927 | 0 | 0 |
| --- | --- | --- | --- | --- | --- | --- | --- | --- |
| novel_180 | 0 | 2 | 0 | 0 | 0 | 0.51962561 | 0 | 0 |
| novel_189 | 39 | 76 | 6 | 3 | 7.189692856 | 19.74577317 | 0.617135 | 0.301103937 |
| novel_190 | 52 | 23 | 18 | 20 | 9.586257142 | 5.975694512 | 1.851405 | 2.007359582 |
| novel_192 | 67 | 38 | 3 | 3 | 12.35152362 | 9.872886585 | 0.3085675 | 0.301103937 |
| novel_217 | 0 | 9 | 1 | 3 | 0 | 2.338315244 | 0.102855833 | 0.301103937 |
| novel_225 | 0 | 1 | 0 | 0 | 0 | 0.259812805 | 0 | 0 |
| novel_234 | 8 | 3 | 7 | 11 | 1.474808791 | 0.779438415 | 0.719990833 | 1.10404777 |
| novel_245 | 1 | 1 | 5 | 7 | 0.184351099 | 0.259812805 | 0.514279167 | 0.702575854 |
| novel_248 | 4 | 5 | 2 | 3 | 0.737404396 | 1.299064024 | 0.205711667 | 0.301103937 |
| novel_249 | 2 | 2 | 4 | 6 | 0.368702198 | 0.51962561 | 0.411423333 | 0.602207875 |
| novel_262 | 0 | 0 | 0 | 1 | 0 | 0 | 0 | 0.100367979 |
| novel_275 | 4 | 4 | 3 | 1 | 0.737404396 | 1.039251219 | 0.3085675 | 0.100367979 |
| novel_276 | 8 | 0 | 0 | 0 | 1.474808791 | 0 | 0 | 0 |
| novel_294 | 0 | 0 | 5 | 0 | 0 | 0 | 0.514279167 | 0 |
| novel_295 | 0 | 0 | 0 | 1 | 0 | 0 | 0 | 0.100367979 |
| novel_304 | 0 | 1 | 0 | 0 | 0 | 0.259812805 | 0 | 0 |
| novel_312 | 0 | 0 | 1 | 0 | 0 | 0 | 0.102855833 | 0 |
| novel_326 | 0 | 0 | 0 | 1 | 0 | 0 | 0 | 0.100367979 |
| novel_342 | 2 | 0 | 1 | 0 | 0.368702198 | 0 | 0.102855833 | 0 |
| novel_343 | 0 | 3 | 0 | 0 | 0 | 0.779438415 | 0 | 0 |
| novel_346 | 0 | 0 | 0 | 3 | 0 | 0 | 0 | 0.301103937 |
| novel_352 | 0 | 0 | 0 | 3 | 0 | 0 | 0 | 0.301103937 |
| novel_355 | 0 | 4 | 7 | 1 | 0 | 1.039251219 | 0.719990833 | 0.100367979 |
| novel_361 | 0 | 11 | 0 | 0 | 0 | 2.857940854 | 0 | 0 |
| novel_383 | 116 | 11 | 33 | 29 | 21.38472747 | 2.857940854 | 3.394242501 | 2.910671395 |
| novel_384 | 812 | 155 | 123 | 139 | 149.6930923 | 40.27098476 | 12.6512675 | 13.9511491 |
| novel_405 | 0 | 1 | 0 | 0 | 0 | 0.259812805 | 0 | 0 |
| novel_417 | 0 | 1 | 0 | 0 | 0 | 0.259812805 | 0 | 0 |
| novel_423 | 2 | 4 | 4 | 1 | 0.368702198 | 1.039251219 | 0.411423333 | 0.100367979 |
| novel_446 | 0 | 0 | 6 | 0 | 0 | 0 | 0.617135 | 0 |
| novel_452 | 1 | 0 | 2 | 3 | 0.184351099 | 0 | 0.205711667 | 0.301103937 |
| novel_455 | 0 | 0 | 2 | 0 | 0 | 0 | 0.205711667 | 0 |
| novel_468 | 0 | 5 | 8 | 0 | 0 | 1.299064024 | 0.822846667 | 0 |
| novel_475 | 0 | 4 | 0 | 0 | 0 | 1.039251219 | 0 | 0 |
| novel_482 | 6 | 6 | 13 | 8 | 1.106106593 | 1.558876829 | 1.337125834 | 0.802943833 |
| novel_488 | 5 | 0 | 1 | 0 | 0.921755494 | 0 | 0.102855833 | 0 |
| novel_490 | 7 | 0 | 0 | 0 | 1.290457692 | 0 | 0 | 0 |
| novel_491 | 0 | 0 | 0 | 5 | 0 | 0 | 0 | 0.501839896 |
| novel_494 | 5 | 0 | 6 | 4 | 0.921755494 | 0 | 0.617135 | 0.401471916 |
| novel_498 | 2 | 14 | 0 | 0 | 0.368702198 | 3.637379268 | 0 | 0 |
| novel_502 | 5 | 6 | 7 | 3 | 0.921755494 | 1.558876829 | 0.719990833 | 0.301103937 |
| novel_503 | 4 | 1 | 4 | 7 | 0.737404396 | 0.259812805 | 0.411423333 | 0.702575854 |
| novel_508 | 0 | 13 | 0 | 0 | 0 | 3.377566463 | 0 | 0 |
| novel_510 | 0 | 1 | 0 | 1 | 0 | 0.259812805 | 0 | 0.100367979 |
| novel_519 | 4 | 7 | 0 | 2 | 0.737404396 | 1.818689634 | 0 | 0.200735958 |
| novel_527 | 0 | 8 | 0 | 0 | 0 | 2.078502439 | 0 | 0 |
| novel_532 | 3 | 17 | 2 | 5 | 0.553053297 | 4.416817683 | 0.205711667 | 0.501839896 |
| novel_560 | 39 | 46 | 9 | 16 | 7.189692856 | 11.95138902 | 0.9257025 | 1.605887666 |
| novel_565 | 0 | 1 | 0 | 0 | 0 | 0.259812805 | 0 | 0 |
| novel_566 | 11 | 1 | 5 | 5 | 2.027862088 | 0.259812805 | 0.514279167 | 0.501839896 |
| novel_567 | 3 | 0 | 0 | 2 | 0.553053297 | 0 | 0 | 0.200735958 |
| novel_601 | 0 | 0 | 1 | 2 | 0 | 0 | 0.102855833 | 0.200735958 |
| novel_602 | 8 | 0 | 2 | 0 | 1.474808791 | 0 | 0.205711667 | 0 |
| novel_606 | 0 | 7 | 0 | 1 | 0 | 1.818689634 | 0 | 0.100367979 |
| novel_621 | 2 | 0 | 4 | 1 | 0.368702198 | 0 | 0.411423333 | 0.100367979 |
| novel_626 | 0 | 9 | 0 | 0 | 0 | 2.338315244 | 0 | 0 |
| novel_633 | 43 | 11 | 6 | 7 | 7.927097252 | 2.857940854 | 0.617135 | 0.702575854 |
| novel_641 | 12 | 1 | 1 | 0 | 2.212213187 | 0.259812805 | 0.102855833 | 0 |
| novel_652 | 12 | 0 | 0 | 0 | 2.212213187 | 0 | 0 | 0 |
| novel_653 | 0 | 1 | 1 | 3 | 0 | 0.259812805 | 0.102855833 | 0.301103937 |
| novel_658 | 0 | 0 | 0 | 6 | 0 | 0 | 0 | 0.602207875 |
| novel_66 | 7 | 4 | 0 | 0 | 1.290457692 | 1.039251219 | 0 | 0 |
| novel_661 | 10 | 2 | 0 | 0 | 1.843510989 | 0.51962561 | 0 | 0 |
| novel_668 | 0 | 2 | 0 | 0 | 0 | 0.51962561 | 0 | 0 |
| novel_671 | 0 | 1 | 2 | 1 | 0 | 0.259812805 | 0.205711667 | 0.100367979 |
| novel_677 | 0 | 0 | 1 | 2 | 0 | 0 | 0.102855833 | 0.200735958 |
| novel_680 | 6 | 0 | 0 | 0 | 1.106106593 | 0 | 0 | 0 |
| novel_684 | 23 | 10 | 28 | 22 | 4.240075274 | 2.598128049 | 2.879963334 | 2.208095541 |
| novel_696 | 0 | 1 | 4 | 6 | 0 | 0.259812805 | 0.411423333 | 0.602207875 |
| novel_697 | 11 | 0 | 0 | 0 | 2.027862088 | 0 | 0 | 0 |
| novel_698 | 2 | 0 | 4 | 5 | 0.368702198 | 0 | 0.411423333 | 0.501839896 |
| novel_701 | 5 | 0 | 0 | 0 | 0.921755494 | 0 | 0 | 0 |
| novel_710 | 0 | 4 | 0 | 0 | 0 | 1.039251219 | 0 | 0 |
| novel_714 | 6 | 0 | 1 | 0 | 1.106106593 | 0 | 0.102855833 | 0 |

| novel_735 | 6 | 2 | 0 | 0 | 1.106106593 | 0.51962561 | 0 | 0 |
| --- | --- | --- | --- | --- | --- | --- | --- | --- |
| novel_740 | 4 | 0 | 0 | 0 | 0.737404396 | 0 | 0 | 0 |
| novel_741 | 0 | 0 | 1 | 0 | 0 | 0 | 0.102855833 | 0 |
| novel_748 | 14 | 5 | 3 | 3 | 2.580915384 | 1.299064024 | 0.3085675 | 0.301103937 |
| novel_749 | 0 | 0 | 2 | 7 | 0 | 0 | 0.205711667 | 0.702575854 |
| novel_762 | 11 | 12 | 7 | 6 | 2.027862088 | 3.117753658 | 0.719990833 | 0.602207875 |
| novel_765 | 0 | 7 | 0 | 0 | 0 | 1.818689634 | 0 | 0 |
| novel_766 | 0 | 10 | 0 | 3 | 0 | 2.598128049 | 0 | 0.301103937 |
| novel_773 | 1 | 0 | 0 | 0 | 0.184351099 | 0 | 0 | 0 |
| novel_778 | 9 | 4 | 0 | 0 | 1.65915989 | 1.039251219 | 0 | 0 |
| novel_795 | 0 | 4 | 0 | 0 | 0 | 1.039251219 | 0 | 0 |
| novel_796 | 3 | 0 | 0 | 0 | 0.553053297 | 0 | 0 | 0 |
| novel_797 | 0 | 0 | 6 | 1 | 0 | 0 | 0.617135 | 0.100367979 |
| novel_799 | 0 | 0 | 4 | 0 | 0 | 0 | 0.411423333 | 0 |
| novel_805 | 0 | 3 | 0 | 2 | 0 | 0.779438415 | 0 | 0.200735958 |
| novel_808 | 0 | 3 | 0 | 0 | 0 | 0.779438415 | 0 | 0 |
| novel_811 | 4 | 2 | 0 | 0 | 0.737404396 | 0.51962561 | 0 | 0 |
| novel_819 | 0 | 0 | 0 | 5 | 0 | 0 | 0 | 0.501839896 |
| novel_823 | 0 | 0 | 7 | 0 | 0 | 0 | 0.719990833 | 0 |
| novel_828 | 2 | 0 | 0 | 0 | 0.368702198 | 0 | 0 | 0 |
| novel_833 | 5 | 0 | 0 | 0 | 0.921755494 | 0 | 0 | 0 |
| novel_834 | 1 | 3 | 0 | 0 | 0.184351099 | 0.779438415 | 0 | 0 |
| novel_842 | 5 | 0 | 0 | 0 | 0.921755494 | 0 | 0 | 0 |
| novel_848 | 1 | 0 | 2 | 2 | 0.184351099 | 0 | 0.205711667 | 0.200735958 |
| novel_92 | 426 | 945 | 631 | 698 | 78.53356812 | 245.5231006 | 64.90203085 | 70.05684943 |
