# Supplementary material for: Identification of Novel Multi-Omics Expression Landscapes and Meta-Analysis of Landscape-Based Competitive Endogenous RNA Networks in ALDH+ Lung Adenocarcinoma Stem Cells
Source: Biomed Res Int. 2022 Aug 31;2022:9545609. doi: 10.1155/2022/9545609 (PMC9453044; doi:10.1155/2022/9545609)
Supplement: Supplementary Materials — The supporting information includes the following: Figure S1. First base bias and position base bias of novel miRNAs; Table S1. Detailed information of miRNAs; Table S2. Novel lncRNAs; Table S3. Novel miRNAs; Table S4. Novel circRNAs; Table S5. Information on ceRNA-based molecular interactions and pairs. [file 9545609.f1.zip › Table_S4_Novel_circRNAs.doc]

circRNA

LTC1	LTC2	LSC1	LSC2	LTC1.tpm	LTC2.tpm	LSC1.tpm	LSC2.tpm

readcount

| hsa_circ_0000014 | 0 | 7 | 2 | 11 | 0 | 1204.40468 | 421.9409283 2553.98189 |
| --- | --- | --- | --- | --- | --- | --- | --- |
| hsa_circ_0000033 | 4 | 2 | 4 | 4 | 693.962526 | 344.1156228 | 843.8818565 928.7206873 |
| hsa_circ_0000069 | 12 | 6 | 10 | 2 | 2081.887578 | 1032.346869 | 2109.704641 464.3603436 |
| hsa_circ_0000091 | 3 | 11 | 6 | 6 | 520.4718945 | 1892.635926 | 1265.822785 1393.081031 |
| hsa_circ_0000095 | 10 | 6 | 0 | 2 | 1734.906315 | 1032.346869 | 0 464.3603436 |
| hsa_circ_0000098 | 3 | 3 | 0 | 0 | 520.4718945 | 516.1734343 | 0 0 |
| hsa_circ_0000117 | 2 | 2 | 2 | 6 | 346.981263 | 344.1156228 | 421.9409283 1393.081031 |
| hsa_circ_0000118 | 12 | 19 | 14 | 37 | 2081.887578 | 3269.098417 | 2953.586498 8590.666357 |
| hsa_circ_0000119 | 6 | 12 | 5 | 10 | 1040.943789 | 2064.693737 | 1054.852321 2321.801718 |
| hsa_circ_0000153 | 6 | 4 | 3 | 0 | 1040.943789 | 688.2312457 | 632.9113924 0 |
| hsa_circ_0000160 | 5 | 3 | 9 | 0 | 867.4531575 | 516.1734343 | 1898.734177 0 |
| hsa_circ_0000175 | 6 | 15 | 7 | 17 | 1040.943789 | 2580.867171 | 1476.793249 3947.062921 |
| hsa_circ_0000195 | 2 | 3 | 4 | 0 | 346.981263 | 516.1734343 | 843.8818565 0 |
| hsa_circ_0000198 | 4 | 3 | 0 | 0 | 693.962526 | 516.1734343 | 0 0 |
| hsa_circ_0000211 | 4 | 4 | 3 | 0 | 693.962526 | 688.2312457 | 632.9113924 0 |
| hsa_circ_0000219 | 9 | 8 | 7 | 2 | 1561.415684 | 1376.462491 | 1476.793249 464.3603436 |
| hsa_circ_0000231 | 98 | 87 | 64 | 26 | 17002.08189 | 14969.02959 | 13502.1097 6036.684467 |
| hsa_circ_0000264 | 11 | 0 | 3 | 10 | 1908.396947 | 0 | 632.9113924 2321.801718 |
| hsa_circ_0000277 | 11 | 14 | 8 | 7 | 1908.396947 | 2408.80936 | 1687.763713 1625.261203 |
| hsa_circ_0000280 | 3 | 7 | 5 | 2 | 520.4718945 | 1204.40468 | 1054.852321 464.3603436 |
| hsa_circ_0000296 | 2 | 5 | 0 | 0 | 346.981263 | 860.2890571 | 0 0 |
| hsa_circ_0000337 | 2 | 2 | 0 | 2 | 346.981263 | 344.1156228 | 0 464.3603436 |
| hsa_circ_0000344 | 4 | 3 | 2 | 3 | 693.962526 | 516.1734343 | 421.9409283 696.5405154 |
| hsa_circ_0000347 | 0 | 0 | 2 | 3 | 0 | 0 | 421.9409283 696.5405154 |
| hsa_circ_0000378 | 3 | 2 | 0 | 0 | 520.4718945 | 344.1156228 | 0 0 |
| hsa_circ_0000386 | 0 | 2 | 5 | 4 | 0 | 344.1156228 | 1054.852321 928.7206873 |
| hsa_circ_0000396 | 7 | 8 | 7 | 11 | 1214.434421 | 1376.462491 | 1476.793249 2553.98189 |
| hsa_circ_0000397 | 8 | 13 | 5 | 20 | 1387.925052 | 2236.751549 | 1054.852321 4643.603436 |
| hsa_circ_0000398 | 3 | 3 | 0 | 0 | 520.4718945 | 516.1734343 | 0 0 |
| hsa_circ_0000399 | 2 | 4 | 4 | 0 | 346.981263 | 688.2312457 | 843.8818565 0 |
| hsa_circ_0000417 | 4 | 6 | 0 | 8 | 693.962526 | 1032.346869 | 0 1857.441375 |
| hsa_circ_0000418 | 0 | 5 | 4 | 7 | 0 | 860.2890571 | 843.8818565 1625.261203 |
| hsa_circ_0000437 | 3 | 4 | 0 | 8 | 520.4718945 | 688.2312457 | 0 1857.441375 |
| hsa_circ_0000439 | 0 | 0 | 4 | 2 | 0 | 0 | 843.8818565 464.3603436 |
| hsa_circ_0000443 | 6 | 3 | 4 | 3 | 1040.943789 | 516.1734343 | 843.8818565 696.5405154 |
| hsa_circ_0000448 | 11 | 7 | 7 | 9 | 1908.396947 | 1204.40468 | 1476.793249 2089.621546 |
| hsa_circ_0000467 | 5 | 0 | 6 | 3 | 867.4531575 | 0 | 1265.822785 696.5405154 |
| hsa_circ_0000471 | 10 | 4 | 6 | 14 | 1734.906315 | 688.2312457 | 1265.822785 3250.522405 |
| hsa_circ_0000478 | 12 | 13 | 10 | 9 | 2081.887578 | 2236.751549 | 2109.704641 2089.621546 |
| hsa_circ_0000489 | 10 | 10 | 4 | 0 | 1734.906315 | 1720.578114 | 843.8818565 0 |
| hsa_circ_0000504 | 2 | 4 | 0 | 0 | 346.981263 | 688.2312457 | 0 0 |
| hsa_circ_0000523 | 3 | 4 | 0 | 5 | 520.4718945 | 688.2312457 | 0 1160.900859 |
| hsa_circ_0000544 | 8 | 10 | 6 | 3 | 1387.925052 | 1720.578114 | 1265.822785 696.5405154 |
| hsa_circ_0000545 | 4 | 0 | 3 | 3 | 693.962526 | 0 | 632.9113924 696.5405154 |
| hsa_circ_0000566 | 5 | 5 | 9 | 11 | 867.4531575 | 860.2890571 | 1898.734177 2553.98189 |
| hsa_circ_0000567 | 31 | 17 | 8 | 12 | 5378.209577 | 2924.982794 | 1687.763713 2786.162062 |
| hsa_circ_0000571 | 0 | 0 | 2 | 2 | 0 | 0 | 421.9409283 464.3603436 |
| hsa_circ_0000586 | 15 | 22 | 16 | 13 | 2602.359473 | 3785.271851 | 3375.527426 3018.342234 |
| hsa_circ_0000592 | 6 | 3 | 9 | 4 | 1040.943789 | 516.1734343 | 1898.734177 928.7206873 |
| hsa_circ_0000607 | 43 | 19 | 29 | 20 | 7460.097155 | 3269.098417 | 6118.14346 4643.603436 |
| hsa_circ_0000609 | 3 | 3 | 13 | 0 | 520.4718945 | 516.1734343 | 2742.616034 0 |
| hsa_circ_0000620 | 11 | 9 | 3 | 0 | 1908.396947 | 1548.520303 | 632.9113924 0 |
| hsa_circ_0000629 | 4 | 4 | 0 | 0 | 693.962526 | 688.2312457 | 0 0 |
| hsa_circ_0000638 | 7 | 13 | 8 | 7 | 1214.434421 | 2236.751549 | 1687.763713 1625.261203 |
| hsa_circ_0000642 | 5 | 0 | 6 | 5 | 867.4531575 | 0 | 1265.822785 1160.900859 |
| hsa_circ_0000643 | 4 | 9 | 7 | 12 | 693.962526 | 1548.520303 | 1476.793249 2786.162062 |
| hsa_circ_0000647 | 2 | 4 | 0 | 0 | 346.981263 | 688.2312457 | 0 0 |
| hsa_circ_0000672 | 3 | 5 | 3 | 4 | 520.4718945 | 860.2890571 | 632.9113924 928.7206873 |
| hsa_circ_0000698 | 24 | 15 | 16 | 4 | 4163.775156 | 2580.867171 | 3375.527426 928.7206873 |
| hsa_circ_0000711 | 10 | 19 | 17 | 19 | 1734.906315 | 3269.098417 | 3586.49789 4411.423264 |
| hsa_circ_0000780 | 3 | 0 | 3 | 2 | 520.4718945 | 0 | 632.9113924 464.3603436 |
| hsa_circ_0000799 | 8 | 10 | 11 | 14 | 1387.925052 | 1720.578114 | 2320.675105 3250.522405 |
| hsa_circ_0000817 | 5 | 5 | 2 | 0 | 867.4531575 | 860.2890571 | 421.9409283 0 |
| hsa_circ_0000818 | 8 | 2 | 4 | 5 | 1387.925052 | 344.1156228 | 843.8818565 1160.900859 |
| hsa_circ_0000826 | 9 | 7 | 3 | 5 | 1561.415684 | 1204.40468 | 632.9113924 1160.900859 |
| hsa_circ_0000831 | 6 | 13 | 6 | 11 | 1040.943789 | 2236.751549 | 1265.822785 2553.98189 |
| hsa_circ_0000835 | 36 | 28 | 30 | 12 | 6245.662734 | 4817.61872 | 6329.113924 2786.162062 |
| hsa_circ_0000836 | 14 | 8 | 14 | 9 | 2428.868841 | 1376.462491 | 2953.586498 2089.621546 |
| hsa_circ_0000839 | 8 | 5 | 10 | 6 | 1387.925052 | 860.2890571 | 2109.704641 1393.081031 |
| hsa_circ_0000842 | 3 | 0 | 3 | 2 | 520.4718945 | 0 | 632.9113924 464.3603436 |
| hsa_circ_0000847 | 23 | 15 | 10 | 9 | 3990.284525 | 2580.867171 | 2109.704641 2089.621546 |
| hsa_circ_0000849 | 2 | 3 | 0 | 2 | 346.981263 | 516.1734343 | 0 464.3603436 |
| hsa_circ_0000854 | 0 | 2 | 2 | 4 | 0 | 344.1156228 | 421.9409283 928.7206873 |
| hsa_circ_0000894 | 3 | 2 | 0 | 0 | 520.4718945 | 344.1156228 | 0 0 |
| hsa_circ_0000943 | 0 | 7 | 3 | 3 | 0 | 1204.40468 | 632.9113924 696.5405154 |

| hsa_circ_0000987 | 0 | 11 | 5 | 2 | 0 | 1892.635926 | 1054.852321 464.3603436 |
| --- | --- | --- | --- | --- | --- | --- | --- |
| hsa_circ_0000997 | 7 | 5 | 6 | 8 | 1214.434421 | 860.2890571 | 1265.822785 1857.441375 |
| hsa_circ_0001006 | 24 | 23 | 24 | 15 | 4163.775156 | 3957.329663 | 5063.291139 3482.702577 |
| hsa_circ_0001009 | 7 | 6 | 9 | 8 | 1214.434421 | 1032.346869 | 1898.734177 1857.441375 |
| hsa_circ_0001016 | 3 | 0 | 4 | 8 | 520.4718945 | 0 | 843.8818565 1857.441375 |
| hsa_circ_0001017 | 39 | 30 | 25 | 27 | 6766.134629 | 5161.734343 | 5274.261603 6268.864639 |
| hsa_circ_0001020 | 5 | 0 | 3 | 2 | 867.4531575 | 0 | 632.9113924 464.3603436 |
| hsa_circ_0001023 | 9 | 10 | 6 | 6 | 1561.415684 | 1720.578114 | 1265.822785 1393.081031 |
| hsa_circ_0001051 | 0 | 0 | 2 | 3 | 0 | 0 | 421.9409283 696.5405154 |
| hsa_circ_0001061 | 0 | 3 | 2 | 2 | 0 | 516.1734343 | 421.9409283 464.3603436 |
| hsa_circ_0001064 | 5 | 3 | 3 | 4 | 867.4531575 | 516.1734343 | 632.9113924 928.7206873 |
| hsa_circ_0001068 | 2 | 2 | 5 | 2 | 346.981263 | 344.1156228 | 1054.852321 464.3603436 |
| hsa_circ_0001074 | 2 | 0 | 3 | 2 | 346.981263 | 0 | 632.9113924 464.3603436 |
| hsa_circ_0001081 | 2 | 3 | 0 | 0 | 346.981263 | 516.1734343 | 0 0 |
| hsa_circ_0001083 | 23 | 18 | 16 | 13 | 3990.284525 | 3097.040606 | 3375.527426 3018.342234 |
| hsa_circ_0001085 | 5 | 16 | 16 | 8 | 867.4531575 | 2752.924983 | 3375.527426 1857.441375 |
| hsa_circ_0001098 | 6 | 12 | 0 | 0 | 1040.943789 | 2064.693737 | 0 0 |
| hsa_circ_0001103 | 17 | 11 | 10 | 0 | 2949.340736 | 1892.635926 | 2109.704641 0 |
| hsa_circ_0001112 | 18 | 16 | 6 | 9 | 3122.831367 | 2752.924983 | 1265.822785 2089.621546 |
| hsa_circ_0001116 | 0 | 5 | 8 | 3 | 0 | 860.2890571 | 1687.763713 696.5405154 |
| hsa_circ_0001118 | 14 | 6 | 25 | 6 | 2428.868841 | 1032.346869 | 5274.261603 1393.081031 |
| hsa_circ_0001130 | 20 | 10 | 11 | 3 | 3469.81263 | 1720.578114 | 2320.675105 696.5405154 |
| hsa_circ_0001165 | 3 | 3 | 0 | 0 | 520.4718945 | 516.1734343 | 0 0 |
| hsa_circ_0001181 | 12 | 12 | 18 | 7 | 2081.887578 | 2064.693737 | 3797.468354 1625.261203 |
| hsa_circ_0001189 | 3 | 6 | 2 | 5 | 520.4718945 | 1032.346869 | 421.9409283 1160.900859 |
| hsa_circ_0001246 | 11 | 3 | 2 | 0 | 1908.396947 | 516.1734343 | 421.9409283 0 |
| hsa_circ_0001247 | 20 | 18 | 15 | 8 | 3469.81263 | 3097.040606 | 3164.556962 1857.441375 |
| hsa_circ_0001258 | 10 | 4 | 4 | 6 | 1734.906315 | 688.2312457 | 843.8818565 1393.081031 |
| hsa_circ_0001278 | 9 | 13 | 5 | 6 | 1561.415684 | 2236.751549 | 1054.852321 1393.081031 |
| hsa_circ_0001313 | 3 | 3 | 2 | 7 | 520.4718945 | 516.1734343 | 421.9409283 1625.261203 |
| hsa_circ_0001315 | 7 | 7 | 0 | 0 | 1214.434421 | 1204.40468 | 0 0 |
| hsa_circ_0001345 | 12 | 7 | 6 | 4 | 2081.887578 | 1204.40468 | 1265.822785 928.7206873 |
| hsa_circ_0001346 | 43 | 34 | 39 | 21 | 7460.097155 | 5849.965588 | 8227.848101 4875.783608 |
| hsa_circ_0001355 | 2 | 7 | 0 | 5 | 346.981263 | 1204.40468 | 0 1160.900859 |
| hsa_circ_0001358 | 0 | 0 | 3 | 4 | 0 | 0 | 632.9113924 928.7206873 |
| hsa_circ_0001380 | 2 | 6 | 6 | 2 | 346.981263 | 1032.346869 | 1265.822785 464.3603436 |
| hsa_circ_0001383 | 2 | 4 | 0 | 3 | 346.981263 | 688.2312457 | 0 696.5405154 |
| hsa_circ_0001386 | 14 | 8 | 6 | 4 | 2428.868841 | 1376.462491 | 1265.822785 928.7206873 |
| hsa_circ_0001402 | 3 | 4 | 0 | 0 | 520.4718945 | 688.2312457 | 0 0 |
| hsa_circ_0001414 | 2 | 6 | 0 | 5 | 346.981263 | 1032.346869 | 0 1160.900859 |
| hsa_circ_0001417 | 0 | 0 | 4 | 7 | 0 | 0 | 843.8818565 1625.261203 |
| hsa_circ_0001434 | 11 | 8 | 4 | 2 | 1908.396947 | 1376.462491 | 843.8818565 464.3603436 |
| hsa_circ_0001439 | 10 | 11 | 6 | 5 | 1734.906315 | 1892.635926 | 1265.822785 1160.900859 |
| hsa_circ_0001451 | 12 | 16 | 12 | 19 | 2081.887578 | 2752.924983 | 2531.64557 4411.423264 |
| hsa_circ_0001460 | 10 | 10 | 0 | 8 | 1734.906315 | 1720.578114 | 0 1857.441375 |
| hsa_circ_0001467 | 0 | 0 | 3 | 3 | 0 | 0 | 632.9113924 696.5405154 |
| hsa_circ_0001472 | 4 | 5 | 2 | 4 | 693.962526 | 860.2890571 | 421.9409283 928.7206873 |
| hsa_circ_0001478 | 2 | 2 | 2 | 0 | 346.981263 | 344.1156228 | 421.9409283 0 |
| hsa_circ_0001481 | 18 | 30 | 7 | 3 | 3122.831367 | 5161.734343 | 1476.793249 696.5405154 |
| hsa_circ_0001487 | 4 | 9 | 2 | 0 | 693.962526 | 1548.520303 | 421.9409283 0 |
| hsa_circ_0001492 | 13 | 9 | 12 | 3 | 2255.37821 | 1548.520303 | 2531.64557 696.5405154 |
| hsa_circ_0001495 | 5 | 11 | 10 | 5 | 867.4531575 | 1892.635926 | 2109.704641 1160.900859 |
| hsa_circ_0001518 | 17 | 12 | 8 | 5 | 2949.340736 | 2064.693737 | 1687.763713 1160.900859 |
| hsa_circ_0001519 | 16 | 11 | 11 | 0 | 2775.850104 | 1892.635926 | 2320.675105 0 |
| hsa_circ_0001522 | 28 | 23 | 14 | 19 | 4857.737682 | 3957.329663 | 2953.586498 4411.423264 |
| hsa_circ_0001535 | 36 | 44 | 22 | 43 | 6245.662734 | 7570.543703 | 4641.350211 9983.747388 |
| hsa_circ_0001550 | 65 | 61 | 46 | 31 | 11276.89105 | 10495.5265 | 9704.64135 7197.585326 |
| hsa_circ_0001551 | 6 | 4 | 2 | 8 | 1040.943789 | 688.2312457 | 421.9409283 1857.441375 |
| hsa_circ_0001554 | 13 | 6 | 5 | 0 | 2255.37821 | 1032.346869 | 1054.852321 0 |
| hsa_circ_0001558 | 22 | 26 | 20 | 8 | 3816.793893 | 4473.503097 | 4219.409283 1857.441375 |
| hsa_circ_0001564 | 0 | 8 | 5 | 3 | 0 | 1376.462491 | 1054.852321 696.5405154 |
| hsa_circ_0001566 | 27 | 37 | 28 | 8 | 4684.247051 | 6366.139023 | 5907.172996 1857.441375 |
| hsa_circ_0001573 | 21 | 11 | 24 | 12 | 3643.303262 | 1892.635926 | 5063.291139 2786.162062 |
| hsa_circ_0001575 | 0 | 0 | 4 | 5 | 0 | 0 | 843.8818565 1160.900859 |
| hsa_circ_0001613 | 8 | 5 | 6 | 5 | 1387.925052 | 860.2890571 | 1265.822785 1160.900859 |
| hsa_circ_0001615 | 2 | 5 | 0 | 2 | 346.981263 | 860.2890571 | 0 464.3603436 |
| hsa_circ_0001632 | 8 | 2 | 3 | 0 | 1387.925052 | 344.1156228 | 632.9113924 0 |
| hsa_circ_0001649 | 3 | 2 | 0 | 6 | 520.4718945 | 344.1156228 | 0 1393.081031 |
| hsa_circ_0001661 | 6 | 2 | 5 | 6 | 1040.943789 | 344.1156228 | 1054.852321 1393.081031 |
| hsa_circ_0001663 | 3 | 4 | 7 | 7 | 520.4718945 | 688.2312457 | 1476.793249 1625.261203 |
| hsa_circ_0001667 | 6 | 3 | 5 | 2 | 1040.943789 | 516.1734343 | 1054.852321 464.3603436 |
| hsa_circ_0001685 | 10 | 8 | 6 | 5 | 1734.906315 | 1376.462491 | 1265.822785 1160.900859 |
| hsa_circ_0001686 | 18 | 16 | 23 | 10 | 3122.831367 | 2752.924983 | 4852.320675 2321.801718 |
| hsa_circ_0001687 | 6 | 2 | 0 | 4 | 1040.943789 | 344.1156228 | 0 928.7206873 |
| hsa_circ_0001696 | 3 | 4 | 2 | 0 | 520.4718945 | 688.2312457 | 421.9409283 0 |
| hsa_circ_0001699 | 10 | 5 | 7 | 9 | 1734.906315 | 860.2890571 | 1476.793249 2089.621546 |
| hsa_circ_0001700 | 2 | 4 | 4 | 2 | 346.981263 | 688.2312457 | 843.8818565 464.3603436 |

| hsa_circ_0001725 | 9 | 9 | 11 | 12 | 1561.415684 | 1548.520303 | 2320.675105 2786.162062 |
| --- | --- | --- | --- | --- | --- | --- | --- |
| hsa_circ_0001727 | 12 | 23 | 17 | 14 | 2081.887578 | 3957.329663 | 3586.49789 3250.522405 |
| hsa_circ_0001741 | 15 | 11 | 7 | 7 | 2602.359473 | 1892.635926 | 1476.793249 1625.261203 |
| hsa_circ_0001746 | 12 | 0 | 6 | 8 | 2081.887578 | 0 | 1265.822785 1857.441375 |
| hsa_circ_0001747 | 5 | 2 | 2 | 3 | 867.4531575 | 344.1156228 | 421.9409283 696.5405154 |
| hsa_circ_0001750 | 4 | 4 | 5 | 2 | 693.962526 | 688.2312457 | 1054.852321 464.3603436 |
| hsa_circ_0001769 | 2 | 9 | 4 | 3 | 346.981263 | 1548.520303 | 843.8818565 696.5405154 |
| hsa_circ_0001772 | 20 | 11 | 14 | 11 | 3469.81263 | 1892.635926 | 2953.586498 2553.98189 |
| hsa_circ_0001776 | 2 | 4 | 0 | 0 | 346.981263 | 688.2312457 | 0 0 |
| hsa_circ_0001777 | 11 | 9 | 3 | 5 | 1908.396947 | 1548.520303 | 632.9113924 1160.900859 |
| hsa_circ_0001788 | 6 | 3 | 3 | 0 | 1040.943789 | 516.1734343 | 632.9113924 0 |
| hsa_circ_0001798 | 0 | 2 | 3 | 8 | 0 | 344.1156228 | 632.9113924 1857.441375 |
| hsa_circ_0001811 | 0 | 0 | 2 | 2 | 0 | 0 | 421.9409283 464.3603436 |
| hsa_circ_0001821 | 17 | 14 | 14 | 11 | 2949.340736 | 2408.80936 | 2953.586498 2553.98189 |
| hsa_circ_0001847 | 18 | 7 | 13 | 7 | 3122.831367 | 1204.40468 | 2742.616034 1625.261203 |
| hsa_circ_0001861 | 0 | 9 | 2 | 4 | 0 | 1548.520303 | 421.9409283 928.7206873 |
| hsa_circ_0001865 | 3 | 2 | 0 | 0 | 520.4718945 | 344.1156228 | 0 0 |
| hsa_circ_0001868 | 3 | 15 | 4 | 4 | 520.4718945 | 2580.867171 | 843.8818565 928.7206873 |
| hsa_circ_0001869 | 0 | 3 | 7 | 3 | 0 | 516.1734343 | 1476.793249 696.5405154 |
| hsa_circ_0001875 | 20 | 15 | 15 | 9 | 3469.81263 | 2580.867171 | 3164.556962 2089.621546 |
| hsa_circ_0001882 | 8 | 13 | 4 | 12 | 1387.925052 | 2236.751549 | 843.8818565 2786.162062 |
| hsa_circ_0001900 | 108 | 117 | 67 | 23 | 18736.9882 | 20130.76394 | 14135.0211 5340.143952 |
| hsa_circ_0001906 | 4 | 3 | 0 | 0 | 693.962526 | 516.1734343 | 0 0 |
| hsa_circ_0001922 | 3 | 6 | 3 | 0 | 520.4718945 | 1032.346869 | 632.9113924 0 |
| hsa_circ_0001944 | 17 | 20 | 31 | 20 | 2949.340736 | 3441.156228 | 6540.084388 4643.603436 |
| hsa_circ_0001946 | 418 | 446 | 655 | 922 | 72519.08397 | 76737.7839 | 138185.654 214070.1184 |
| hsa_circ_0001958 | 4 | 10 | 3 | 6 | 693.962526 | 1720.578114 | 632.9113924 1393.081031 |
| hsa_circ_0001977 | 9 | 4 | 4 | 3 | 1561.415684 | 688.2312457 | 843.8818565 696.5405154 |
| hsa_circ_0002024 | 16 | 17 | 14 | 0 | 2775.850104 | 2924.982794 | 2953.586498 0 |
| hsa_circ_0002058 | 3 | 5 | 3 | 5 | 520.4718945 | 860.2890571 | 632.9113924 1160.900859 |
| hsa_circ_0002059 | 2 | 5 | 6 | 4 | 346.981263 | 860.2890571 | 1265.822785 928.7206873 |
| hsa_circ_0002076 | 36 | 29 | 42 | 14 | 6245.662734 | 4989.676531 | 8860.759494 3250.522405 |
| hsa_circ_0002085 | 5 | 6 | 9 | 0 | 867.4531575 | 1032.346869 | 1898.734177 0 |
| hsa_circ_0002111 | 42 | 68 | 25 | 20 | 7286.606523 | 11699.93118 | 5274.261603 4643.603436 |
| hsa_circ_0002138 | 9 | 12 | 3 | 3 | 1561.415684 | 2064.693737 | 632.9113924 696.5405154 |
| hsa_circ_0002141 | 2 | 10 | 6 | 0 | 346.981263 | 1720.578114 | 1265.822785 0 |
| hsa_circ_0002158 | 9 | 2 | 3 | 0 | 1561.415684 | 344.1156228 | 632.9113924 0 |
| hsa_circ_0002172 | 3 | 4 | 3 | 3 | 520.4718945 | 688.2312457 | 632.9113924 696.5405154 |
| hsa_circ_0002220 | 6 | 4 | 4 | 2 | 1040.943789 | 688.2312457 | 843.8818565 464.3603436 |
| hsa_circ_0002274 | 4 | 4 | 0 | 4 | 693.962526 | 688.2312457 | 0 928.7206873 |
| hsa_circ_0002301 | 0 | 3 | 6 | 3 | 0 | 516.1734343 | 1265.822785 696.5405154 |
| hsa_circ_0002317 | 4 | 2 | 4 | 0 | 693.962526 | 344.1156228 | 843.8818565 0 |
| hsa_circ_0002346 | 3 | 11 | 0 | 2 | 520.4718945 | 1892.635926 | 0 464.3603436 |
| hsa_circ_0002348 | 6 | 11 | 0 | 0 | 1040.943789 | 1892.635926 | 0 0 |
| hsa_circ_0002374 | 10 | 3 | 4 | 3 | 1734.906315 | 516.1734343 | 843.8818565 696.5405154 |
| hsa_circ_0002392 | 2 | 2 | 2 | 0 | 346.981263 | 344.1156228 | 421.9409283 0 |
| hsa_circ_0002398 | 7 | 7 | 2 | 2 | 1214.434421 | 1204.40468 | 421.9409283 464.3603436 |
| hsa_circ_0002431 | 2 | 2 | 2 | 0 | 346.981263 | 344.1156228 | 421.9409283 0 |
| hsa_circ_0002457 | 3 | 6 | 3 | 9 | 520.4718945 | 1032.346869 | 632.9113924 2089.621546 |
| hsa_circ_0002465 | 6 | 6 | 5 | 6 | 1040.943789 | 1032.346869 | 1054.852321 1393.081031 |
| hsa_circ_0002483 | 3 | 8 | 5 | 0 | 520.4718945 | 1376.462491 | 1054.852321 0 |
| hsa_circ_0002490 | 0 | 0 | 2 | 3 | 0 | 0 | 421.9409283 696.5405154 |
| hsa_circ_0002544 | 0 | 5 | 2 | 8 | 0 | 860.2890571 | 421.9409283 1857.441375 |
| hsa_circ_0002556 | 4 | 8 | 5 | 0 | 693.962526 | 1376.462491 | 1054.852321 0 |
| hsa_circ_0002564 | 5 | 5 | 0 | 8 | 867.4531575 | 860.2890571 | 0 1857.441375 |
| hsa_circ_0002603 | 2 | 2 | 0 | 0 | 346.981263 | 344.1156228 | 0 0 |
| hsa_circ_0002607 | 8 | 3 | 0 | 4 | 1387.925052 | 516.1734343 | 0 928.7206873 |
| hsa_circ_0002665 | 5 | 0 | 5 | 5 | 867.4531575 | 0 | 1054.852321 1160.900859 |
| hsa_circ_0002669 | 6 | 3 | 4 | 3 | 1040.943789 | 516.1734343 | 843.8818565 696.5405154 |
| hsa_circ_0002693 | 2 | 5 | 0 | 0 | 346.981263 | 860.2890571 | 0 0 |
| hsa_circ_0002744 | 5 | 3 | 2 | 0 | 867.4531575 | 516.1734343 | 421.9409283 0 |
| hsa_circ_0002750 | 7 | 6 | 0 | 0 | 1214.434421 | 1032.346869 | 0 0 |
| hsa_circ_0002762 | 6 | 3 | 0 | 0 | 1040.943789 | 516.1734343 | 0 0 |
| hsa_circ_0002782 | 165 | 130 | 107 | 47 | 28625.9542 | 22367.51549 | 22573.83966 10912.46808 |
| hsa_circ_0002789 | 5 | 0 | 2 | 2 | 867.4531575 | 0 | 421.9409283 464.3603436 |
| hsa_circ_0002802 | 4 | 3 | 0 | 6 | 693.962526 | 516.1734343 | 0 1393.081031 |
| hsa_circ_0002876 | 4 | 2 | 3 | 0 | 693.962526 | 344.1156228 | 632.9113924 0 |
| hsa_circ_0002922 | 71 | 74 | 0 | 52 | 12317.83484 | 12732.27805 | 0 12073.36893 |
| hsa_circ_0002926 | 3 | 0 | 4 | 3 | 520.4718945 | 0 | 843.8818565 696.5405154 |
| hsa_circ_0002928 | 8 | 3 | 4 | 3 | 1387.925052 | 516.1734343 | 843.8818565 696.5405154 |
| hsa_circ_0002985 | 0 | 0 | 2 | 2 | 0 | 0 | 421.9409283 464.3603436 |
| hsa_circ_0002999 | 0 | 3 | 2 | 2 | 0 | 516.1734343 | 421.9409283 464.3603436 |
| hsa_circ_0003008 | 4 | 3 | 0 | 0 | 693.962526 | 516.1734343 | 0 0 |
| hsa_circ_0003045 | 0 | 0 | 3 | 5 | 0 | 0 | 632.9113924 1160.900859 |
| hsa_circ_0003047 | 2 | 2 | 0 | 0 | 346.981263 | 344.1156228 | 0 0 |
| hsa_circ_0003054 | 4 | 0 | 3 | 2 | 693.962526 | 0 | 632.9113924 464.3603436 |
| hsa_circ_0003063 | 12 | 22 | 19 | 9 | 2081.887578 | 3785.271851 | 4008.438819 2089.621546 |

| hsa_circ_0003102 | 8 | 2 | 0 | 0 | 1387.925052 | 344.1156228 | 0 0 |
| --- | --- | --- | --- | --- | --- | --- | --- |
| hsa_circ_0003141 | 0 | 0 | 2 | 2 | 0 | 0 | 421.9409283 464.3603436 |
| hsa_circ_0003171 | 5 | 5 | 4 | 3 | 867.4531575 | 860.2890571 | 843.8818565 696.5405154 |
| hsa_circ_0003177 | 4 | 0 | 3 | 3 | 693.962526 | 0 | 632.9113924 696.5405154 |
| hsa_circ_0003220 | 2 | 5 | 4 | 0 | 346.981263 | 860.2890571 | 843.8818565 0 |
| hsa_circ_0003221 | 3 | 5 | 0 | 0 | 520.4718945 | 860.2890571 | 0 0 |
| hsa_circ_0003239 | 3 | 6 | 9 | 6 | 520.4718945 | 1032.346869 | 1898.734177 1393.081031 |
| hsa_circ_0003250 | 3 | 3 | 0 | 6 | 520.4718945 | 516.1734343 | 0 1393.081031 |
| hsa_circ_0003267 | 22 | 17 | 10 | 18 | 3816.793893 | 2924.982794 | 2109.704641 4179.243093 |
| hsa_circ_0003270 | 4 | 0 | 6 | 3 | 693.962526 | 0 | 1265.822785 696.5405154 |
| hsa_circ_0003273 | 2 | 2 | 0 | 2 | 346.981263 | 344.1156228 | 0 464.3603436 |
| hsa_circ_0003295 | 7 | 2 | 2 | 0 | 1214.434421 | 344.1156228 | 421.9409283 0 |
| hsa_circ_0003302 | 4 | 4 | 3 | 4 | 693.962526 | 688.2312457 | 632.9113924 928.7206873 |
| hsa_circ_0003322 | 6 | 4 | 0 | 0 | 1040.943789 | 688.2312457 | 0 0 |
| hsa_circ_0003327 | 2 | 3 | 0 | 0 | 346.981263 | 516.1734343 | 0 0 |
| hsa_circ_0003353 | 3 | 9 | 0 | 2 | 520.4718945 | 1548.520303 | 0 464.3603436 |
| hsa_circ_0003401 | 0 | 0 | 2 | 5 | 0 | 0 | 421.9409283 1160.900859 |
| hsa_circ_0003441 | 16 | 20 | 8 | 9 | 2775.850104 | 3441.156228 | 1687.763713 2089.621546 |
| hsa_circ_0003458 | 2 | 3 | 5 | 0 | 346.981263 | 516.1734343 | 1054.852321 0 |
| hsa_circ_0003472 | 4 | 2 | 3 | 2 | 693.962526 | 344.1156228 | 632.9113924 464.3603436 |
| hsa_circ_0003500 | 2 | 2 | 6 | 3 | 346.981263 | 344.1156228 | 1265.822785 696.5405154 |
| hsa_circ_0003502 | 4 | 6 | 5 | 0 | 693.962526 | 1032.346869 | 1054.852321 0 |
| hsa_circ_0003507 | 4 | 0 | 2 | 2 | 693.962526 | 0 | 421.9409283 464.3603436 |
| hsa_circ_0003511 | 2 | 2 | 5 | 2 | 346.981263 | 344.1156228 | 1054.852321 464.3603436 |
| hsa_circ_0003513 | 4 | 4 | 3 | 7 | 693.962526 | 688.2312457 | 632.9113924 1625.261203 |
| hsa_circ_0003608 | 2 | 3 | 0 | 0 | 346.981263 | 516.1734343 | 0 0 |
| hsa_circ_0003632 | 5 | 2 | 3 | 2 | 867.4531575 | 344.1156228 | 632.9113924 464.3603436 |
| hsa_circ_0003633 | 5 | 3 | 0 | 6 | 867.4531575 | 516.1734343 | 0 1393.081031 |
| hsa_circ_0003643 | 4 | 9 | 0 | 9 | 693.962526 | 1548.520303 | 0 2089.621546 |
| hsa_circ_0003659 | 3 | 8 | 2 | 3 | 520.4718945 | 1376.462491 | 421.9409283 696.5405154 |
| hsa_circ_0003664 | 0 | 3 | 2 | 3 | 0 | 516.1734343 | 421.9409283 696.5405154 |
| hsa_circ_0003694 | 14 | 11 | 0 | 2 | 2428.868841 | 1892.635926 | 0 464.3603436 |
| hsa_circ_0003698 | 9 | 11 | 4 | 4 | 1561.415684 | 1892.635926 | 843.8818565 928.7206873 |
| hsa_circ_0003718 | 4 | 4 | 2 | 3 | 693.962526 | 688.2312457 | 421.9409283 696.5405154 |
| hsa_circ_0003731 | 12 | 9 | 11 | 25 | 2081.887578 | 1548.520303 | 2320.675105 5804.504295 |
| hsa_circ_0003764 | 4 | 4 | 0 | 0 | 693.962526 | 688.2312457 | 0 0 |
| hsa_circ_0003823 | 23 | 22 | 11 | 0 | 3990.284525 | 3785.271851 | 2320.675105 0 |
| hsa_circ_0003836 | 0 | 2 | 4 | 2 | 0 | 344.1156228 | 843.8818565 464.3603436 |
| hsa_circ_0003877 | 6 | 3 | 0 | 0 | 1040.943789 | 516.1734343 | 0 0 |
| hsa_circ_0003885 | 2 | 2 | 3 | 0 | 346.981263 | 344.1156228 | 632.9113924 0 |
| hsa_circ_0003898 | 4 | 4 | 0 | 0 | 693.962526 | 688.2312457 | 0 0 |
| hsa_circ_0003909 | 3 | 5 | 0 | 0 | 520.4718945 | 860.2890571 | 0 0 |
| hsa_circ_0003915 | 6 | 12 | 5 | 3 | 1040.943789 | 2064.693737 | 1054.852321 696.5405154 |
| hsa_circ_0003922 | 5 | 2 | 4 | 9 | 867.4531575 | 344.1156228 | 843.8818565 2089.621546 |
| hsa_circ_0003961 | 7 | 3 | 3 | 0 | 1214.434421 | 516.1734343 | 632.9113924 0 |
| hsa_circ_0003965 | 3 | 3 | 0 | 0 | 520.4718945 | 516.1734343 | 0 0 |
| hsa_circ_0004058 | 12 | 8 | 15 | 6 | 2081.887578 | 1376.462491 | 3164.556962 1393.081031 |
| hsa_circ_0004089 | 2 | 0 | 3 | 4 | 346.981263 | 0 | 632.9113924 928.7206873 |
| hsa_circ_0004127 | 3 | 2 | 0 | 0 | 520.4718945 | 344.1156228 | 0 0 |
| hsa_circ_0004166 | 2 | 3 | 0 | 2 | 346.981263 | 516.1734343 | 0 464.3603436 |
| hsa_circ_0004191 | 5 | 6 | 3 | 6 | 867.4531575 | 1032.346869 | 632.9113924 1393.081031 |
| hsa_circ_0004210 | 0 | 0 | 3 | 3 | 0 | 0 | 632.9113924 696.5405154 |
| hsa_circ_0004220 | 4 | 4 | 3 | 3 | 693.962526 | 688.2312457 | 632.9113924 696.5405154 |
| hsa_circ_0004243 | 5 | 7 | 2 | 0 | 867.4531575 | 1204.40468 | 421.9409283 0 |
| hsa_circ_0004269 | 12 | 14 | 10 | 3 | 2081.887578 | 2408.80936 | 2109.704641 696.5405154 |
| hsa_circ_0004270 | 5 | 3 | 3 | 0 | 867.4531575 | 516.1734343 | 632.9113924 0 |
| hsa_circ_0004273 | 2 | 3 | 0 | 0 | 346.981263 | 516.1734343 | 0 0 |
| hsa_circ_0004314 | 12 | 11 | 11 | 10 | 2081.887578 | 1892.635926 | 2320.675105 2321.801718 |
| hsa_circ_0004338 | 0 | 6 | 6 | 6 | 0 | 1032.346869 | 1265.822785 1393.081031 |
| hsa_circ_0004364 | 4 | 4 | 0 | 0 | 693.962526 | 688.2312457 | 0 0 |
| hsa_circ_0004366 | 0 | 2 | 2 | 2 | 0 | 344.1156228 | 421.9409283 464.3603436 |
| hsa_circ_0004368 | 2 | 2 | 5 | 2 | 346.981263 | 344.1156228 | 1054.852321 464.3603436 |
| hsa_circ_0004382 | 5 | 4 | 2 | 0 | 867.4531575 | 688.2312457 | 421.9409283 0 |
| hsa_circ_0004383 | 6 | 2 | 11 | 0 | 1040.943789 | 344.1156228 | 2320.675105 0 |
| hsa_circ_0004416 | 7 | 3 | 4 | 2 | 1214.434421 | 516.1734343 | 843.8818565 464.3603436 |
| hsa_circ_0004417 | 22 | 25 | 18 | 10 | 3816.793893 | 4301.445286 | 3797.468354 2321.801718 |
| hsa_circ_0004422 | 3 | 3 | 0 | 0 | 520.4718945 | 516.1734343 | 0 0 |
| hsa_circ_0004435 | 6 | 3 | 6 | 0 | 1040.943789 | 516.1734343 | 1265.822785 0 |
| hsa_circ_0004488 | 7 | 5 | 3 | 0 | 1214.434421 | 860.2890571 | 632.9113924 0 |
| hsa_circ_0004527 | 0 | 0 | 2 | 2 | 0 | 0 | 421.9409283 464.3603436 |
| hsa_circ_0004565 | 2 | 3 | 4 | 3 | 346.981263 | 516.1734343 | 843.8818565 696.5405154 |
| hsa_circ_0004566 | 2 | 2 | 0 | 4 | 346.981263 | 344.1156228 | 0 928.7206873 |
| hsa_circ_0004587 | 2 | 7 | 2 | 2 | 346.981263 | 1204.40468 | 421.9409283 464.3603436 |
| hsa_circ_0004628 | 2 | 2 | 3 | 0 | 346.981263 | 344.1156228 | 632.9113924 0 |
| hsa_circ_0004652 | 13 | 7 | 5 | 2 | 2255.37821 | 1204.40468 | 1054.852321 464.3603436 |
| hsa_circ_0004664 | 2 | 4 | 6 | 3 | 346.981263 | 688.2312457 | 1265.822785 696.5405154 |
| hsa_circ_0004671 | 6 | 5 | 7 | 2 | 1040.943789 | 860.2890571 | 1476.793249 464.3603436 |

| hsa_circ_0004673 | 0 | 3 | 4 | 2 | 0 | 516.1734343 | 843.8818565 464.3603436 |
| --- | --- | --- | --- | --- | --- | --- | --- |
| hsa_circ_0004689 | 2 | 5 | 0 | 0 | 346.981263 | 860.2890571 | 0 0 |
| hsa_circ_0004771 | 5 | 4 | 5 | 5 | 867.4531575 | 688.2312457 | 1054.852321 1160.900859 |
| hsa_circ_0004791 | 10 | 10 | 7 | 2 | 1734.906315 | 1720.578114 | 1476.793249 464.3603436 |
| hsa_circ_0004805 | 3 | 2 | 6 | 5 | 520.4718945 | 344.1156228 | 1265.822785 1160.900859 |
| hsa_circ_0004849 | 7 | 10 | 5 | 6 | 1214.434421 | 1720.578114 | 1054.852321 1393.081031 |
| hsa_circ_0004870 | 3 | 3 | 0 | 0 | 520.4718945 | 516.1734343 | 0 0 |
| hsa_circ_0004874 | 0 | 3 | 9 | 5 | 0 | 516.1734343 | 1898.734177 1160.900859 |
| hsa_circ_0004887 | 0 | 12 | 6 | 3 | 0 | 2064.693737 | 1265.822785 696.5405154 |
| hsa_circ_0004915 | 10 | 6 | 8 | 0 | 1734.906315 | 1032.346869 | 1687.763713 0 |
| hsa_circ_0004924 | 5 | 7 | 9 | 3 | 867.4531575 | 1204.40468 | 1898.734177 696.5405154 |
| hsa_circ_0004983 | 2 | 0 | 2 | 2 | 346.981263 | 0 | 421.9409283 464.3603436 |
| hsa_circ_0004994 | 3 | 2 | 2 | 10 | 520.4718945 | 344.1156228 | 421.9409283 2321.801718 |
| hsa_circ_0005005 | 2 | 3 | 0 | 0 | 346.981263 | 516.1734343 | 0 0 |
| hsa_circ_0005023 | 2 | 4 | 0 | 0 | 346.981263 | 688.2312457 | 0 0 |
| hsa_circ_0005044 | 3 | 4 | 0 | 0 | 520.4718945 | 688.2312457 | 0 0 |
| hsa_circ_0005077 | 4 | 0 | 5 | 4 | 693.962526 | 0 | 1054.852321 928.7206873 |
| hsa_circ_0005090 | 11 | 11 | 8 | 9 | 1908.396947 | 1892.635926 | 1687.763713 2089.621546 |
| hsa_circ_0005131 | 0 | 0 | 2 | 2 | 0 | 0 | 421.9409283 464.3603436 |
| hsa_circ_0005142 | 2 | 6 | 0 | 2 | 346.981263 | 1032.346869 | 0 464.3603436 |
| hsa_circ_0005147 | 4 | 8 | 4 | 5 | 693.962526 | 1376.462491 | 843.8818565 1160.900859 |
| hsa_circ_0005181 | 7 | 4 | 9 | 5 | 1214.434421 | 688.2312457 | 1898.734177 1160.900859 |
| hsa_circ_0005214 | 5 | 2 | 0 | 0 | 867.4531575 | 344.1156228 | 0 0 |
| hsa_circ_0005227 | 2 | 2 | 5 | 0 | 346.981263 | 344.1156228 | 1054.852321 0 |
| hsa_circ_0005231 | 2 | 2 | 0 | 0 | 346.981263 | 344.1156228 | 0 0 |
| hsa_circ_0005245 | 16 | 16 | 12 | 4 | 2775.850104 | 2752.924983 | 2531.64557 928.7206873 |
| hsa_circ_0005251 | 3 | 4 | 2 | 2 | 520.4718945 | 688.2312457 | 421.9409283 464.3603436 |
| hsa_circ_0005252 | 4 | 6 | 0 | 0 | 693.962526 | 1032.346869 | 0 0 |
| hsa_circ_0005260 | 10 | 9 | 4 | 4 | 1734.906315 | 1548.520303 | 843.8818565 928.7206873 |
| hsa_circ_0005272 | 2 | 2 | 0 | 0 | 346.981263 | 344.1156228 | 0 0 |
| hsa_circ_0005281 | 4 | 2 | 0 | 0 | 693.962526 | 344.1156228 | 0 0 |
| hsa_circ_0005309 | 2 | 6 | 0 | 0 | 346.981263 | 1032.346869 | 0 0 |
| hsa_circ_0005311 | 2 | 2 | 10 | 0 | 346.981263 | 344.1156228 | 2109.704641 0 |
| hsa_circ_0005325 | 2 | 3 | 0 | 0 | 346.981263 | 516.1734343 | 0 0 |
| hsa_circ_0005337 | 2 | 2 | 0 | 0 | 346.981263 | 344.1156228 | 0 0 |
| hsa_circ_0005379 | 15 | 2 | 8 | 3 | 2602.359473 | 344.1156228 | 1687.763713 696.5405154 |
| hsa_circ_0005405 | 4 | 7 | 3 | 2 | 693.962526 | 1204.40468 | 632.9113924 464.3603436 |
| hsa_circ_0005418 | 4 | 8 | 4 | 2 | 693.962526 | 1376.462491 | 843.8818565 464.3603436 |
| hsa_circ_0005435 | 3 | 2 | 2 | 4 | 520.4718945 | 344.1156228 | 421.9409283 928.7206873 |
| hsa_circ_0005465 | 10 | 0 | 4 | 5 | 1734.906315 | 0 | 843.8818565 1160.900859 |
| hsa_circ_0005476 | 2 | 4 | 3 | 3 | 346.981263 | 688.2312457 | 632.9113924 696.5405154 |
| hsa_circ_0005483 | 4 | 0 | 3 | 6 | 693.962526 | 0 | 632.9113924 1393.081031 |
| hsa_circ_0005542 | 6 | 0 | 2 | 2 | 1040.943789 | 0 | 421.9409283 464.3603436 |
| hsa_circ_0005567 | 10 | 13 | 5 | 0 | 1734.906315 | 2236.751549 | 1054.852321 0 |
| hsa_circ_0005581 | 0 | 0 | 4 | 2 | 0 | 0 | 843.8818565 464.3603436 |
| hsa_circ_0005594 | 0 | 2 | 2 | 2 | 0 | 344.1156228 | 421.9409283 464.3603436 |
| hsa_circ_0005623 | 6 | 7 | 3 | 0 | 1040.943789 | 1204.40468 | 632.9113924 0 |
| hsa_circ_0005630 | 0 | 3 | 2 | 2 | 0 | 516.1734343 | 421.9409283 464.3603436 |
| hsa_circ_0005638 | 3 | 4 | 0 | 2 | 520.4718945 | 688.2312457 | 0 464.3603436 |
| hsa_circ_0005646 | 5 | 5 | 8 | 0 | 867.4531575 | 860.2890571 | 1687.763713 0 |
| hsa_circ_0005653 | 3 | 0 | 6 | 2 | 520.4718945 | 0 | 1265.822785 464.3603436 |
| hsa_circ_0005667 | 4 | 4 | 3 | 3 | 693.962526 | 688.2312457 | 632.9113924 696.5405154 |
| hsa_circ_0005683 | 4 | 5 | 0 | 4 | 693.962526 | 860.2890571 | 0 928.7206873 |
| hsa_circ_0005695 | 10 | 3 | 3 | 0 | 1734.906315 | 516.1734343 | 632.9113924 0 |
| hsa_circ_0005704 | 8 | 2 | 0 | 0 | 1387.925052 | 344.1156228 | 0 0 |
| hsa_circ_0005720 | 4 | 6 | 2 | 0 | 693.962526 | 1032.346869 | 421.9409283 0 |
| hsa_circ_0005772 | 2 | 5 | 4 | 0 | 346.981263 | 860.2890571 | 843.8818565 0 |
| hsa_circ_0005775 | 4 | 2 | 0 | 0 | 693.962526 | 344.1156228 | 0 0 |
| hsa_circ_0005841 | 4 | 2 | 3 | 0 | 693.962526 | 344.1156228 | 632.9113924 0 |
| hsa_circ_0005881 | 4 | 4 | 4 | 3 | 693.962526 | 688.2312457 | 843.8818565 696.5405154 |
| hsa_circ_0005898 | 6 | 13 | 0 | 0 | 1040.943789 | 2236.751549 | 0 0 |
| hsa_circ_0005939 | 5 | 10 | 12 | 4 | 867.4531575 | 1720.578114 | 2531.64557 928.7206873 |
| hsa_circ_0005941 | 8 | 6 | 4 | 0 | 1387.925052 | 1032.346869 | 843.8818565 0 |
| hsa_circ_0005954 | 6 | 4 | 6 | 7 | 1040.943789 | 688.2312457 | 1265.822785 1625.261203 |
| hsa_circ_0005970 | 2 | 2 | 0 | 0 | 346.981263 | 344.1156228 | 0 0 |
| hsa_circ_0005982 | 2 | 9 | 5 | 4 | 346.981263 | 1548.520303 | 1054.852321 928.7206873 |
| hsa_circ_0005988 | 2 | 2 | 0 | 0 | 346.981263 | 344.1156228 | 0 0 |
| hsa_circ_0006006 | 8 | 2 | 0 | 4 | 1387.925052 | 344.1156228 | 0 928.7206873 |
| hsa_circ_0006010 | 0 | 0 | 4 | 2 | 0 | 0 | 843.8818565 464.3603436 |
| hsa_circ_0006049 | 2 | 3 | 0 | 0 | 346.981263 | 516.1734343 | 0 0 |
| hsa_circ_0006062 | 0 | 5 | 4 | 2 | 0 | 860.2890571 | 843.8818565 464.3603436 |
| hsa_circ_0006087 | 0 | 0 | 2 | 5 | 0 | 0 | 421.9409283 1160.900859 |
| hsa_circ_0006114 | 8 | 7 | 2 | 6 | 1387.925052 | 1204.40468 | 421.9409283 1393.081031 |
| hsa_circ_0006117 | 0 | 0 | 2 | 5 | 0 | 0 | 421.9409283 1160.900859 |
| hsa_circ_0006151 | 8 | 7 | 7 | 2 | 1387.925052 | 1204.40468 | 1476.793249 464.3603436 |
| hsa_circ_0006157 | 18 | 11 | 8 | 2 | 3122.831367 | 1892.635926 | 1687.763713 464.3603436 |
| hsa_circ_0006168 | 2 | 3 | 0 | 0 | 346.981263 | 516.1734343 | 0 0 |

| hsa_circ_0006189 | 2 | 3 | 0 | 0 | 346.981263 | 516.1734343 | 0 0 |
| --- | --- | --- | --- | --- | --- | --- | --- |
| hsa_circ_0006258 | 22 | 16 | 7 | 5 | 3816.793893 | 2752.924983 | 1476.793249 1160.900859 |
| hsa_circ_0006308 | 0 | 5 | 5 | 5 | 0 | 860.2890571 | 1054.852321 1160.900859 |
| hsa_circ_0006324 | 2 | 4 | 4 | 2 | 346.981263 | 688.2312457 | 843.8818565 464.3603436 |
| hsa_circ_0006346 | 2 | 4 | 0 | 3 | 346.981263 | 688.2312457 | 0 696.5405154 |
| hsa_circ_0006360 | 6 | 4 | 0 | 0 | 1040.943789 | 688.2312457 | 0 0 |
| hsa_circ_0006364 | 4 | 7 | 2 | 3 | 693.962526 | 1204.40468 | 421.9409283 696.5405154 |
| hsa_circ_0006373 | 0 | 4 | 4 | 5 | 0 | 688.2312457 | 843.8818565 1160.900859 |
| hsa_circ_0006381 | 0 | 0 | 2 | 2 | 0 | 0 | 421.9409283 464.3603436 |
| hsa_circ_0006393 | 0 | 0 | 2 | 3 | 0 | 0 | 421.9409283 696.5405154 |
| hsa_circ_0006508 | 4 | 7 | 13 | 4 | 693.962526 | 1204.40468 | 2742.616034 928.7206873 |
| hsa_circ_0006528 | 5 | 6 | 4 | 0 | 867.4531575 | 1032.346869 | 843.8818565 0 |
| hsa_circ_0006539 | 3 | 2 | 0 | 0 | 520.4718945 | 344.1156228 | 0 0 |
| hsa_circ_0006609 | 12 | 5 | 4 | 0 | 2081.887578 | 860.2890571 | 843.8818565 0 |
| hsa_circ_0006675 | 2 | 5 | 3 | 2 | 346.981263 | 860.2890571 | 632.9113924 464.3603436 |
| hsa_circ_0006702 | 27 | 17 | 8 | 10 | 4684.247051 | 2924.982794 | 1687.763713 2321.801718 |
| hsa_circ_0006727 | 0 | 0 | 2 | 3 | 0 | 0 | 421.9409283 696.5405154 |
| hsa_circ_0006801 | 3 | 5 | 0 | 0 | 520.4718945 | 860.2890571 | 0 0 |
| hsa_circ_0006848 | 4 | 5 | 3 | 0 | 693.962526 | 860.2890571 | 632.9113924 0 |
| hsa_circ_0006849 | 0 | 3 | 4 | 2 | 0 | 516.1734343 | 843.8818565 464.3603436 |
| hsa_circ_0006856 | 4 | 3 | 0 | 0 | 693.962526 | 516.1734343 | 0 0 |
| hsa_circ_0006881 | 3 | 2 | 3 | 0 | 520.4718945 | 344.1156228 | 632.9113924 0 |
| hsa_circ_0006916 | 2 | 2 | 5 | 0 | 346.981263 | 344.1156228 | 1054.852321 0 |
| hsa_circ_0006922 | 3 | 6 | 9 | 6 | 520.4718945 | 1032.346869 | 1898.734177 1393.081031 |
| hsa_circ_0006948 | 2 | 4 | 0 | 0 | 346.981263 | 688.2312457 | 0 0 |
| hsa_circ_0006956 | 3 | 2 | 5 | 0 | 520.4718945 | 344.1156228 | 1054.852321 0 |
| hsa_circ_0006990 | 5 | 8 | 5 | 4 | 867.4531575 | 1376.462491 | 1054.852321 928.7206873 |
| hsa_circ_0007001 | 4 | 4 | 0 | 0 | 693.962526 | 688.2312457 | 0 0 |
| hsa_circ_0007099 | 6 | 5 | 0 | 2 | 1040.943789 | 860.2890571 | 0 464.3603436 |
| hsa_circ_0007108 | 2 | 3 | 2 | 0 | 346.981263 | 516.1734343 | 421.9409283 0 |
| hsa_circ_0007127 | 16 | 8 | 23 | 5 | 2775.850104 | 1376.462491 | 4852.320675 1160.900859 |
| hsa_circ_0007130 | 6 | 7 | 0 | 0 | 1040.943789 | 1204.40468 | 0 0 |
| hsa_circ_0007144 | 33 | 31 | 16 | 7 | 5725.19084 | 5333.792154 | 3375.527426 1625.261203 |
| hsa_circ_0007162 | 5 | 4 | 5 | 0 | 867.4531575 | 688.2312457 | 1054.852321 0 |
| hsa_circ_0007178 | 3 | 5 | 0 | 0 | 520.4718945 | 860.2890571 | 0 0 |
| hsa_circ_0007259 | 6 | 2 | 4 | 6 | 1040.943789 | 344.1156228 | 843.8818565 1393.081031 |
| hsa_circ_0007271 | 4 | 3 | 0 | 5 | 693.962526 | 516.1734343 | 0 1160.900859 |
| hsa_circ_0007292 | 13 | 15 | 13 | 16 | 2255.37821 | 2580.867171 | 2742.616034 3714.882749 |
| hsa_circ_0007308 | 6 | 2 | 0 | 4 | 1040.943789 | 344.1156228 | 0 928.7206873 |
| hsa_circ_0007418 | 3 | 2 | 0 | 2 | 520.4718945 | 344.1156228 | 0 464.3603436 |
| hsa_circ_0007430 | 0 | 2 | 6 | 4 | 0 | 344.1156228 | 1265.822785 928.7206873 |
| hsa_circ_0007440 | 2 | 5 | 2 | 0 | 346.981263 | 860.2890571 | 421.9409283 0 |
| hsa_circ_0007444 | 5 | 12 | 3 | 9 | 867.4531575 | 2064.693737 | 632.9113924 2089.621546 |
| hsa_circ_0007466 | 4 | 7 | 0 | 4 | 693.962526 | 1204.40468 | 0 928.7206873 |
| hsa_circ_0007477 | 2 | 4 | 0 | 0 | 346.981263 | 688.2312457 | 0 0 |
| hsa_circ_0007487 | 6 | 7 | 5 | 0 | 1040.943789 | 1204.40468 | 1054.852321 0 |
| hsa_circ_0007509 | 6 | 9 | 7 | 0 | 1040.943789 | 1548.520303 | 1476.793249 0 |
| hsa_circ_0007542 | 6 | 7 | 4 | 0 | 1040.943789 | 1204.40468 | 843.8818565 0 |
| hsa_circ_0007582 | 6 | 3 | 5 | 0 | 1040.943789 | 516.1734343 | 1054.852321 0 |
| hsa_circ_0007621 | 2 | 4 | 2 | 0 | 346.981263 | 688.2312457 | 421.9409283 0 |
| hsa_circ_0007641 | 2 | 2 | 0 | 4 | 346.981263 | 344.1156228 | 0 928.7206873 |
| hsa_circ_0007646 | 6 | 4 | 5 | 8 | 1040.943789 | 688.2312457 | 1054.852321 1857.441375 |
| hsa_circ_0007701 | 2 | 7 | 0 | 0 | 346.981263 | 1204.40468 | 0 0 |
| hsa_circ_0007772 | 2 | 4 | 0 | 0 | 346.981263 | 688.2312457 | 0 0 |
| hsa_circ_0007788 | 7 | 7 | 0 | 0 | 1214.434421 | 1204.40468 | 0 0 |
| hsa_circ_0007804 | 0 | 0 | 2 | 3 | 0 | 0 | 421.9409283 696.5405154 |
| hsa_circ_0007846 | 5 | 5 | 4 | 10 | 867.4531575 | 860.2890571 | 843.8818565 2321.801718 |
| hsa_circ_0007908 | 7 | 9 | 8 | 4 | 1214.434421 | 1548.520303 | 1687.763713 928.7206873 |
| hsa_circ_0007918 | 6 | 15 | 6 | 5 | 1040.943789 | 2580.867171 | 1265.822785 1160.900859 |
| hsa_circ_0007930 | 6 | 0 | 3 | 2 | 1040.943789 | 0 | 632.9113924 464.3603436 |
| hsa_circ_0007941 | 3 | 11 | 3 | 0 | 520.4718945 | 1892.635926 | 632.9113924 0 |
| hsa_circ_0007948 | 0 | 0 | 2 | 2 | 0 | 0 | 421.9409283 464.3603436 |
| hsa_circ_0007976 | 4 | 4 | 0 | 0 | 693.962526 | 688.2312457 | 0 0 |
| hsa_circ_0008011 | 3 | 0 | 7 | 3 | 520.4718945 | 0 | 1476.793249 696.5405154 |
| hsa_circ_0008032 | 0 | 3 | 3 | 3 | 0 | 516.1734343 | 632.9113924 696.5405154 |
| hsa_circ_0008039 | 6 | 3 | 0 | 9 | 1040.943789 | 516.1734343 | 0 2089.621546 |
| hsa_circ_0008043 | 24 | 21 | 23 | 20 | 4163.775156 | 3613.21404 | 4852.320675 4643.603436 |
| hsa_circ_0008054 | 2 | 2 | 0 | 2 | 346.981263 | 344.1156228 | 0 464.3603436 |
| hsa_circ_0008058 | 7 | 7 | 6 | 3 | 1214.434421 | 1204.40468 | 1265.822785 696.5405154 |
| hsa_circ_0008075 | 0 | 0 | 3 | 4 | 0 | 0 | 632.9113924 928.7206873 |
| hsa_circ_0008083 | 4 | 6 | 0 | 2 | 693.962526 | 1032.346869 | 0 464.3603436 |
| hsa_circ_0008131 | 3 | 2 | 0 | 8 | 520.4718945 | 344.1156228 | 0 1857.441375 |
| hsa_circ_0008177 | 2 | 2 | 0 | 0 | 346.981263 | 344.1156228 | 0 0 |
| hsa_circ_0008193 | 6 | 12 | 2 | 9 | 1040.943789 | 2064.693737 | 421.9409283 2089.621546 |
| hsa_circ_0008197 | 2 | 2 | 4 | 2 | 346.981263 | 344.1156228 | 843.8818565 464.3603436 |
| hsa_circ_0008199 | 4 | 8 | 6 | 3 | 693.962526 | 1376.462491 | 1265.822785 696.5405154 |
| hsa_circ_0008202 | 2 | 4 | 0 | 3 | 346.981263 | 688.2312457 | 0 696.5405154 |

| hsa_circ_0008210 | 7 | 5 | 4 | 2 | 1214.434421 | 860.2890571 | 843.8818565 | 464.3603436 |
| --- | --- | --- | --- | --- | --- | --- | --- | --- |
| hsa_circ_0008230 | 4 | 4 | 2 | 0 | 693.962526 | 688.2312457 | 421.9409283 | 0 |
| hsa_circ_0008261 | 2 | 2 | 0 | 0 | 346.981263 | 344.1156228 | 0 | 0 |
| hsa_circ_0008265 | 5 | 5 | 0 | 2 | 867.4531575 | 860.2890571 | 0 | 464.3603436 |
| hsa_circ_0008278 | 3 | 8 | 12 | 0 | 520.4718945 | 1376.462491 | 2531.64557 | 0 |
| hsa_circ_0008303 | 6 | 4 | 10 | 0 | 1040.943789 | 688.2312457 | 2109.704641 | 0 |
| hsa_circ_0008309 | 7 | 13 | 6 | 3 | 1214.434421 | 2236.751549 | 1265.822785 | 696.5405154 |
| hsa_circ_0008319 | 3 | 4 | 2 | 2 | 520.4718945 | 688.2312457 | 421.9409283 | 464.3603436 |
| hsa_circ_0008342 | 4 | 6 | 7 | 0 | 693.962526 | 1032.346869 | 1476.793249 | 0 |
| hsa_circ_0008345 | 5 | 3 | 5 | 0 | 867.4531575 | 516.1734343 | 1054.852321 | 0 |
| hsa_circ_0008351 | 4 | 4 | 0 | 0 | 693.962526 | 688.2312457 | 0 | 0 |
| hsa_circ_0008382 | 0 | 3 | 8 | 9 | 0 | 516.1734343 | 1687.763713 | 2089.621546 |
| hsa_circ_0008417 | 3 | 2 | 0 | 0 | 520.4718945 | 344.1156228 | 0 | 0 |
| hsa_circ_0008433 | 0 | 0 | 10 | 4 | 0 | 0 | 2109.704641 | 928.7206873 |
| hsa_circ_0008460 | 6 | 4 | 5 | 0 | 1040.943789 | 688.2312457 | 1054.852321 | 0 |
| hsa_circ_0008494 | 11 | 8 | 3 | 12 | 1908.396947 | 1376.462491 | 632.9113924 | 2786.162062 |
| hsa_circ_0008500 | 9 | 14 | 5 | 7 | 1561.415684 | 2408.80936 | 1054.852321 | 1625.261203 |
| hsa_circ_0008599 | 2 | 4 | 4 | 0 | 346.981263 | 688.2312457 | 843.8818565 | 0 |
| hsa_circ_0008621 | 2 | 0 | 5 | 6 | 346.981263 | 0 | 1054.852321 | 1393.081031 |
| hsa_circ_0008629 | 2 | 2 | 3 | 0 | 346.981263 | 344.1156228 | 632.9113924 | 0 |
| hsa_circ_0008640 | 2 | 0 | 4 | 2 | 346.981263 | 0 | 843.8818565 | 464.3603436 |
| hsa_circ_0008647 | 6 | 6 | 0 | 2 | 1040.943789 | 1032.346869 | 0 | 464.3603436 |
| hsa_circ_0008678 | 8 | 5 | 3 | 3 | 1387.925052 | 860.2890571 | 632.9113924 | 696.5405154 |
| hsa_circ_0008683 | 7 | 8 | 7 | 0 | 1214.434421 | 1376.462491 | 1476.793249 | 0 |
| hsa_circ_0008694 | 6 | 2 | 0 | 0 | 1040.943789 | 344.1156228 | 0 | 0 |
| hsa_circ_0008719 | 0 | 0 | 3 | 3 | 0 | 0 | 632.9113924 | 696.5405154 |
| hsa_circ_0008720 | 8 | 6 | 0 | 5 | 1387.925052 | 1032.346869 | 0 | 1160.900859 |
| hsa_circ_0008777 | 21 | 11 | 21 | 5 | 3643.303262 | 1892.635926 | 4430.379747 | 1160.900859 |
| hsa_circ_0008797 | 4 | 2 | 0 | 3 | 693.962526 | 344.1156228 | 0 | 696.5405154 |
| hsa_circ_0008798 | 0 | 0 | 2 | 3 | 0 | 0 | 421.9409283 | 696.5405154 |
| hsa_circ_0008812 | 2 | 0 | 2 | 7 | 346.981263 | 0 | 421.9409283 | 1625.261203 |
| hsa_circ_0008861 | 8 | 3 | 4 | 3 | 1387.925052 | 516.1734343 | 843.8818565 | 696.5405154 |
| hsa_circ_0008881 | 2 | 3 | 0 | 0 | 346.981263 | 516.1734343 | 0 | 0 |
| hsa_circ_0008886 | 5 | 2 | 0 | 0 | 867.4531575 | 344.1156228 | 0 | 0 |
| hsa_circ_0008922 | 13 | 19 | 8 | 16 | 2255.37821 | 3269.098417 | 1687.763713 | 3714.882749 |
| hsa_circ_0008927 | 5 | 3 | 2 | 0 | 867.4531575 | 516.1734343 | 421.9409283 | 0 |
| hsa_circ_0008928 | 0 | 4 | 9 | 3 | 0 | 688.2312457 | 1898.734177 | 696.5405154 |
| hsa_circ_0008938 | 3 | 4 | 7 | 0 | 520.4718945 | 688.2312457 | 1476.793249 | 0 |
| hsa_circ_0008951 | 2 | 0 | 2 | 4 | 346.981263 | 0 | 421.9409283 | 928.7206873 |
| hsa_circ_0008953 | 10 | 4 | 3 | 6 | 1734.906315 | 688.2312457 | 632.9113924 | 1393.081031 |
| hsa_circ_0008967 | 5 | 4 | 6 | 0 | 867.4531575 | 688.2312457 | 1265.822785 | 0 |
| hsa_circ_0009008 | 3 | 12 | 0 | 0 | 520.4718945 | 2064.693737 | 0 | 0 |
| hsa_circ_0009022 | 12 | 9 | 7 | 2 | 2081.887578 | 1548.520303 | 1476.793249 | 464.3603436 |
| hsa_circ_0009035 | 7 | 4 | 2 | 2 | 1214.434421 | 688.2312457 | 421.9409283 | 464.3603436 |
| hsa_circ_0009037 | 6 | 3 | 2 | 0 | 1040.943789 | 516.1734343 | 421.9409283 | 0 |
| hsa_circ_0009043 | 17 | 12 | 14 | 8 | 2949.340736 | 2064.693737 | 2953.586498 | 1857.441375 |
| hsa_circ_0009064 | 3 | 5 | 0 | 0 | 520.4718945 | 860.2890571 | 0 | 0 |
| hsa_circ_0009084 | 3 | 2 | 0 | 0 | 520.4718945 | 344.1156228 | 0 | 0 |
| hsa_circ_0009110 | 0 | 0 | 2 | 3 | 0 | 0 | 421.9409283 | 696.5405154 |
| hsa_circ_0009158 | 4 | 5 | 0 | 3 | 693.962526 | 860.2890571 | 0 | 696.5405154 |
| hsa_circ_0010458 | 6 | 0 | 5 | 3 | 1040.943789 | 0 | 1054.852321 | 696.5405154 |
| hsa_circ_0012553 | 2 | 4 | 2 | 0 | 346.981263 | 688.2312457 | 421.9409283 | 0 |
| hsa_circ_0012823 | 0 | 0 | 2 | 3 | 0 | 0 | 421.9409283 | 696.5405154 |
| hsa_circ_0013167 | 0 | 0 | 6 | 2 | 0 | 0 | 1265.822785 | 464.3603436 |
| hsa_circ_0013222 | 5 | 5 | 5 | 2 | 867.4531575 | 860.2890571 | 1054.852321 | 464.3603436 |
| hsa_circ_0014292 | 0 | 0 | 2 | 4 | 0 | 0 | 421.9409283 | 928.7206873 |
| hsa_circ_0014577 | 0 | 0 | 4 | 2 | 0 | 0 | 843.8818565 | 464.3603436 |
| hsa_circ_0014606 | 7 | 4 | 2 | 18 | 1214.434421 | 688.2312457 | 421.9409283 | 4179.243093 |
| hsa_circ_0015027 | 3 | 2 | 0 | 2 | 520.4718945 | 344.1156228 | 0 | 464.3603436 |
| hsa_circ_0015259 | 10 | 2 | 5 | 0 | 1734.906315 | 344.1156228 | 1054.852321 | 0 |
| hsa_circ_0015260 | 2 | 6 | 0 | 0 | 346.981263 | 1032.346869 | 0 | 0 |
| hsa_circ_0015261 | 3 | 4 | 3 | 0 | 520.4718945 | 688.2312457 | 632.9113924 | 0 |
| hsa_circ_0016599 | 11 | 12 | 11 | 16 | 1908.396947 | 2064.693737 | 2320.675105 | 3714.882749 |
| hsa_circ_0016600 | 4 | 3 | 15 | 0 | 693.962526 | 516.1734343 | 3164.556962 | 0 |
| hsa_circ_0016601 | 12 | 25 | 39 | 24 | 2081.887578 | 4301.445286 | 8227.848101 | 5572.324124 |
| hsa_circ_0016867 | 4 | 0 | 5 | 5 | 693.962526 | 0 | 1054.852321 | 1160.900859 |
| hsa_circ_0016956 | 5 | 0 | 5 | 2 | 867.4531575 | 0 | 1054.852321 | 464.3603436 |
| hsa_circ_0016957 | 3 | 3 | 0 | 0 | 520.4718945 | 516.1734343 | 0 | 0 |
| hsa_circ_0017289 | 8 | 19 | 6 | 9 | 1387.925052 | 3269.098417 | 1265.822785 | 2089.621546 |
| hsa_circ_0017586 | 5 | 3 | 2 | 0 | 867.4531575 | 516.1734343 | 421.9409283 | 0 |
| hsa_circ_0017636 | 5 | 2 | 7 | 0 | 867.4531575 | 344.1156228 | 1476.793249 | 0 |
| hsa_circ_0018401 | 0 | 4 | 2 | 7 | 0 | 688.2312457 | 421.9409283 | 1625.261203 |
| hsa_circ_0018478 | 8 | 5 | 5 | 2 | 1387.925052 | 860.2890571 | 1054.852321 | 464.3603436 |
| hsa_circ_0018484 | 2 | 3 | 2 | 2 | 346.981263 | 516.1734343 | 421.9409283 | 464.3603436 |
| hsa_circ_0018524 | 4 | 4 | 0 | 0 | 693.962526 | 688.2312457 | 0 | 0 |
| hsa_circ_0018992 | 12 | 5 | 0 | 0 | 2081.887578 | 860.2890571 | 0 | 0 |
| hsa_circ_0018998 | 4 | 7 | 3 | 2 | 693.962526 | 1204.40468 | 632.9113924 | 464.3603436 |

| hsa_circ_0019170 | 5 | 7 | 2 | 7 | 867.4531575 | 1204.40468 | 421.9409283 1625.261203 |
| --- | --- | --- | --- | --- | --- | --- | --- |
| hsa_circ_0019446 | 3 | 3 | 4 | 0 | 520.4718945 | 516.1734343 | 843.8818565 0 |
| hsa_circ_0021089 | 2 | 4 | 0 | 0 | 346.981263 | 688.2312457 | 0 0 |
| hsa_circ_0021570 | 16 | 13 | 22 | 15 | 2775.850104 | 2236.751549 | 4641.350211 3482.702577 |
| hsa_circ_0021712 | 7 | 5 | 3 | 6 | 1214.434421 | 860.2890571 | 632.9113924 1393.081031 |
| hsa_circ_0023694 | 2 | 2 | 0 | 5 | 346.981263 | 344.1156228 | 0 1160.900859 |
| hsa_circ_0023891 | 0 | 5 | 2 | 3 | 0 | 860.2890571 | 421.9409283 696.5405154 |
| hsa_circ_0023919 | 5 | 7 | 3 | 2 | 867.4531575 | 1204.40468 | 632.9113924 464.3603436 |
| hsa_circ_0023923 | 7 | 5 | 7 | 4 | 1214.434421 | 860.2890571 | 1476.793249 928.7206873 |
| hsa_circ_0023936 | 6 | 4 | 2 | 0 | 1040.943789 | 688.2312457 | 421.9409283 0 |
| hsa_circ_0025349 | 6 | 8 | 0 | 7 | 1040.943789 | 1376.462491 | 0 1625.261203 |
| hsa_circ_0025570 | 3 | 3 | 0 | 2 | 520.4718945 | 516.1734343 | 0 464.3603436 |
| hsa_circ_0025619 | 3 | 4 | 0 | 0 | 520.4718945 | 688.2312457 | 0 0 |
| hsa_circ_0025882 | 4 | 2 | 0 | 0 | 693.962526 | 344.1156228 | 0 0 |
| hsa_circ_0025953 | 2 | 3 | 0 | 0 | 346.981263 | 516.1734343 | 0 0 |
| hsa_circ_0027491 | 3 | 2 | 0 | 5 | 520.4718945 | 344.1156228 | 0 1160.900859 |
| hsa_circ_0027702 | 11 | 7 | 3 | 2 | 1908.396947 | 1204.40468 | 632.9113924 464.3603436 |
| hsa_circ_0028088 | 2 | 3 | 2 | 0 | 346.981263 | 516.1734343 | 421.9409283 0 |
| hsa_circ_0029434 | 8 | 9 | 0 | 3 | 1387.925052 | 1548.520303 | 0 696.5405154 |
| hsa_circ_0029775 | 2 | 4 | 0 | 2 | 346.981263 | 688.2312457 | 0 464.3603436 |
| hsa_circ_0029976 | 2 | 4 | 4 | 0 | 346.981263 | 688.2312457 | 843.8818565 0 |
| hsa_circ_0030389 | 6 | 4 | 4 | 5 | 1040.943789 | 688.2312457 | 843.8818565 1160.900859 |
| hsa_circ_0030632 | 2 | 3 | 2 | 4 | 346.981263 | 516.1734343 | 421.9409283 928.7206873 |
| hsa_circ_0030720 | 6 | 6 | 5 | 6 | 1040.943789 | 1032.346869 | 1054.852321 1393.081031 |
| hsa_circ_0031482 | 3 | 9 | 3 | 3 | 520.4718945 | 1548.520303 | 632.9113924 696.5405154 |
| hsa_circ_0031584 | 9 | 18 | 0 | 9 | 1561.415684 | 3097.040606 | 0 2089.621546 |
| hsa_circ_0031607 | 19 | 19 | 14 | 9 | 3296.321999 | 3269.098417 | 2953.586498 2089.621546 |
| hsa_circ_0031608 | 9 | 9 | 10 | 0 | 1561.415684 | 1548.520303 | 2109.704641 0 |
| hsa_circ_0031884 | 0 | 0 | 2 | 2 | 0 | 0 | 421.9409283 464.3603436 |
| hsa_circ_0032029 | 5 | 0 | 6 | 7 | 867.4531575 | 0 | 1265.822785 1625.261203 |
| hsa_circ_0032965 | 6 | 7 | 7 | 2 | 1040.943789 | 1204.40468 | 1476.793249 464.3603436 |
| hsa_circ_0032969 | 4 | 7 | 13 | 7 | 693.962526 | 1204.40468 | 2742.616034 1625.261203 |
| hsa_circ_0034293 | 4 | 4 | 0 | 4 | 693.962526 | 688.2312457 | 0 928.7206873 |
| hsa_circ_0034803 | 2 | 2 | 0 | 0 | 346.981263 | 344.1156228 | 0 0 |
| hsa_circ_0035189 | 10 | 11 | 7 | 3 | 1734.906315 | 1892.635926 | 1476.793249 696.5405154 |
| hsa_circ_0035285 | 0 | 6 | 7 | 2 | 0 | 1032.346869 | 1476.793249 464.3603436 |
| hsa_circ_0036372 | 10 | 7 | 8 | 3 | 1734.906315 | 1204.40468 | 1687.763713 696.5405154 |
| hsa_circ_0036610 | 3 | 2 | 0 | 0 | 520.4718945 | 344.1156228 | 0 0 |
| hsa_circ_0036629 | 0 | 4 | 3 | 7 | 0 | 688.2312457 | 632.9113924 1625.261203 |
| hsa_circ_0038111 | 4 | 4 | 3 | 0 | 693.962526 | 688.2312457 | 632.9113924 0 |
| hsa_circ_0038816 | 0 | 0 | 2 | 3 | 0 | 0 | 421.9409283 696.5405154 |
| hsa_circ_0039264 | 5 | 5 | 3 | 5 | 867.4531575 | 860.2890571 | 632.9113924 1160.900859 |
| hsa_circ_0040809 | 2 | 3 | 3 | 11 | 346.981263 | 516.1734343 | 632.9113924 2553.98189 |
| hsa_circ_0040823 | 9 | 8 | 2 | 6 | 1561.415684 | 1376.462491 | 421.9409283 1393.081031 |
| hsa_circ_0041150 | 33 | 33 | 22 | 8 | 5725.19084 | 5677.907777 | 4641.350211 1857.441375 |
| hsa_circ_0041152 | 9 | 5 | 0 | 0 | 1561.415684 | 860.2890571 | 0 0 |
| hsa_circ_0041153 | 21 | 18 | 9 | 11 | 3643.303262 | 3097.040606 | 1898.734177 2553.98189 |
| hsa_circ_0041154 | 3 | 3 | 2 | 2 | 520.4718945 | 516.1734343 | 421.9409283 464.3603436 |
| hsa_circ_0042091 | 0 | 3 | 5 | 4 | 0 | 516.1734343 | 1054.852321 928.7206873 |
| hsa_circ_0042498 | 4 | 4 | 0 | 0 | 693.962526 | 688.2312457 | 0 0 |
| hsa_circ_0044686 | 6 | 3 | 3 | 0 | 1040.943789 | 516.1734343 | 632.9113924 0 |
| hsa_circ_0045006 | 2 | 2 | 0 | 0 | 346.981263 | 344.1156228 | 0 0 |
| hsa_circ_0047270 | 8 | 8 | 6 | 13 | 1387.925052 | 1376.462491 | 1265.822785 3018.342234 |
| hsa_circ_0048000 | 2 | 7 | 0 | 0 | 346.981263 | 1204.40468 | 0 0 |
| hsa_circ_0052767 | 3 | 2 | 2 | 0 | 520.4718945 | 344.1156228 | 421.9409283 0 |
| hsa_circ_0052852 | 2 | 0 | 3 | 3 | 346.981263 | 0 | 632.9113924 696.5405154 |
| hsa_circ_0052943 | 0 | 0 | 2 | 3 | 0 | 0 | 421.9409283 696.5405154 |
| hsa_circ_0054886 | 4 | 2 | 2 | 4 | 693.962526 | 344.1156228 | 421.9409283 928.7206873 |
| hsa_circ_0055904 | 0 | 0 | 2 | 2 | 0 | 0 | 421.9409283 464.3603436 |
| hsa_circ_0055906 | 0 | 2 | 2 | 2 | 0 | 344.1156228 | 421.9409283 464.3603436 |
| hsa_circ_0055945 | 2 | 3 | 4 | 10 | 346.981263 | 516.1734343 | 843.8818565 2321.801718 |
| hsa_circ_0056037 | 3 | 2 | 0 | 0 | 520.4718945 | 344.1156228 | 0 0 |
| hsa_circ_0057105 | 6 | 8 | 7 | 2 | 1040.943789 | 1376.462491 | 1476.793249 464.3603436 |
| hsa_circ_0057123 | 2 | 0 | 6 | 2 | 346.981263 | 0 | 1265.822785 464.3603436 |
| hsa_circ_0057523 | 3 | 4 | 0 | 0 | 520.4718945 | 688.2312457 | 0 0 |
| hsa_circ_0058039 | 0 | 6 | 2 | 3 | 0 | 1032.346869 | 421.9409283 696.5405154 |
| hsa_circ_0058042 | 2 | 3 | 2 | 0 | 346.981263 | 516.1734343 | 421.9409283 0 |
| hsa_circ_0058051 | 5 | 3 | 0 | 0 | 867.4531575 | 516.1734343 | 0 0 |
| hsa_circ_0058493 | 11 | 9 | 11 | 4 | 1908.396947 | 1548.520303 | 2320.675105 928.7206873 |
| hsa_circ_0058495 | 18 | 14 | 11 | 4 | 3122.831367 | 2408.80936 | 2320.675105 928.7206873 |
| hsa_circ_0058806 | 4 | 3 | 4 | 0 | 693.962526 | 516.1734343 | 843.8818565 0 |
| hsa_circ_0058912 | 4 | 3 | 6 | 0 | 693.962526 | 516.1734343 | 1265.822785 0 |
| hsa_circ_0061774 | 6 | 8 | 0 | 0 | 1040.943789 | 1376.462491 | 0 0 |
| hsa_circ_0061776 | 7 | 6 | 0 | 0 | 1214.434421 | 1032.346869 | 0 0 |
| hsa_circ_0062603 | 2 | 4 | 2 | 0 | 346.981263 | 688.2312457 | 421.9409283 0 |
| hsa_circ_0062642 | 8 | 2 | 0 | 0 | 1387.925052 | 344.1156228 | 0 0 |
| hsa_circ_0062688 | 2 | 10 | 0 | 2 | 346.981263 | 1720.578114 | 0 464.3603436 |

| hsa_circ_0065244 | 3 | 3 | 0 | 0 | 520.4718945 | 516.1734343 | 0 0 |
| --- | --- | --- | --- | --- | --- | --- | --- |
| hsa_circ_0065301 | 4 | 2 | 0 | 3 | 693.962526 | 344.1156228 | 0 696.5405154 |
| hsa_circ_0065307 | 3 | 2 | 0 | 0 | 520.4718945 | 344.1156228 | 0 0 |
| hsa_circ_0067985 | 3 | 6 | 0 | 5 | 520.4718945 | 1032.346869 | 0 1160.900859 |
| hsa_circ_0069101 | 4 | 4 | 0 | 0 | 693.962526 | 688.2312457 | 0 0 |
| hsa_circ_0069725 | 3 | 3 | 0 | 0 | 520.4718945 | 516.1734343 | 0 0 |
| hsa_circ_0069981 | 4 | 2 | 0 | 10 | 693.962526 | 344.1156228 | 0 2321.801718 |
| hsa_circ_0069982 | 9 | 4 | 6 | 9 | 1561.415684 | 688.2312457 | 1265.822785 2089.621546 |
| hsa_circ_0070022 | 5 | 3 | 7 | 4 | 867.4531575 | 516.1734343 | 1476.793249 928.7206873 |
| hsa_circ_0070039 | 6 | 6 | 0 | 4 | 1040.943789 | 1032.346869 | 0 928.7206873 |
| hsa_circ_0071185 | 3 | 8 | 2 | 0 | 520.4718945 | 1376.462491 | 421.9409283 0 |
| hsa_circ_0071375 | 13 | 0 | 8 | 3 | 2255.37821 | 0 | 1687.763713 696.5405154 |
| hsa_circ_0071653 | 18 | 13 | 0 | 10 | 3122.831367 | 2236.751549 | 0 2321.801718 |
| hsa_circ_0072309 | 12 | 20 | 13 | 10 | 2081.887578 | 3441.156228 | 2742.616034 2321.801718 |
| hsa_circ_0072568 | 3 | 3 | 3 | 2 | 520.4718945 | 516.1734343 | 632.9113924 464.3603436 |
| hsa_circ_0072688 | 3 | 5 | 0 | 15 | 520.4718945 | 860.2890571 | 0 3482.702577 |
| hsa_circ_0073371 | 2 | 5 | 3 | 3 | 346.981263 | 860.2890571 | 632.9113924 696.5405154 |
| hsa_circ_0073517 | 2 | 0 | 3 | 5 | 346.981263 | 0 | 632.9113924 1160.900859 |
| hsa_circ_0073608 | 6 | 3 | 2 | 0 | 1040.943789 | 516.1734343 | 421.9409283 0 |
| hsa_circ_0073706 | 0 | 0 | 7 | 2 | 0 | 0 | 1476.793249 464.3603436 |
| hsa_circ_0073904 | 0 | 0 | 2 | 3 | 0 | 0 | 421.9409283 696.5405154 |
| hsa_circ_0074061 | 5 | 7 | 0 | 3 | 867.4531575 | 1204.40468 | 0 696.5405154 |
| hsa_circ_0074104 | 2 | 2 | 0 | 0 | 346.981263 | 344.1156228 | 0 0 |
| hsa_circ_0074159 | 3 | 3 | 0 | 0 | 520.4718945 | 516.1734343 | 0 0 |
| hsa_circ_0075158 | 4 | 6 | 12 | 6 | 693.962526 | 1032.346869 | 2531.64557 1393.081031 |
| hsa_circ_0075346 | 0 | 0 | 2 | 3 | 0 | 0 | 421.9409283 696.5405154 |
| hsa_circ_0075357 | 3 | 2 | 0 | 0 | 520.4718945 | 344.1156228 | 0 0 |
| hsa_circ_0075835 | 6 | 5 | 0 | 2 | 1040.943789 | 860.2890571 | 0 464.3603436 |
| hsa_circ_0076961 | 3 | 2 | 0 | 0 | 520.4718945 | 344.1156228 | 0 0 |
| hsa_circ_0077216 | 5 | 4 | 2 | 3 | 867.4531575 | 688.2312457 | 421.9409283 696.5405154 |
| hsa_circ_0077495 | 2 | 6 | 10 | 0 | 346.981263 | 1032.346869 | 2109.704641 0 |
| hsa_circ_0078617 | 5 | 9 | 7 | 5 | 867.4531575 | 1548.520303 | 1476.793249 1160.900859 |
| hsa_circ_0078632 | 4 | 3 | 0 | 0 | 693.962526 | 516.1734343 | 0 0 |
| hsa_circ_0079040 | 9 | 4 | 0 | 0 | 1561.415684 | 688.2312457 | 0 0 |
| hsa_circ_0079480 | 3 | 2 | 0 | 0 | 520.4718945 | 344.1156228 | 0 0 |
| hsa_circ_0079673 | 3 | 4 | 3 | 2 | 520.4718945 | 688.2312457 | 632.9113924 464.3603436 |
| hsa_circ_0080835 | 4 | 4 | 0 | 0 | 693.962526 | 688.2312457 | 0 0 |
| hsa_circ_0081808 | 0 | 0 | 8 | 2 | 0 | 0 | 1687.763713 464.3603436 |
| hsa_circ_0083229 | 2 | 2 | 0 | 0 | 346.981263 | 344.1156228 | 0 0 |
| hsa_circ_0083390 | 3 | 2 | 4 | 2 | 520.4718945 | 344.1156228 | 843.8818565 464.3603436 |
| hsa_circ_0083470 | 2 | 2 | 0 | 2 | 346.981263 | 344.1156228 | 0 464.3603436 |
| hsa_circ_0084606 | 4 | 2 | 0 | 10 | 693.962526 | 344.1156228 | 0 2321.801718 |
| hsa_circ_0084615 | 57 | 78 | 32 | 61 | 9888.965996 | 13420.50929 | 6751.054852 14162.99048 |
| hsa_circ_0085323 | 2 | 3 | 0 | 2 | 346.981263 | 516.1734343 | 0 464.3603436 |
| hsa_circ_0085438 | 4 | 5 | 11 | 0 | 693.962526 | 860.2890571 | 2320.675105 0 |
| hsa_circ_0085439 | 2 | 11 | 6 | 0 | 346.981263 | 1892.635926 | 1265.822785 0 |
| hsa_circ_0085440 | 5 | 7 | 4 | 8 | 867.4531575 | 1204.40468 | 843.8818565 1857.441375 |
| hsa_circ_0085441 | 0 | 2 | 4 | 7 | 0 | 344.1156228 | 843.8818565 1625.261203 |
| hsa_circ_0086726 | 5 | 3 | 7 | 4 | 867.4531575 | 516.1734343 | 1476.793249 928.7206873 |
| hsa_circ_0087199 | 4 | 3 | 3 | 3 | 693.962526 | 516.1734343 | 632.9113924 696.5405154 |
| hsa_circ_0087200 | 9 | 22 | 2 | 15 | 1561.415684 | 3785.271851 | 421.9409283 3482.702577 |
| hsa_circ_0087234 | 3 | 8 | 0 | 0 | 520.4718945 | 1376.462491 | 0 0 |
| hsa_circ_0087255 | 2 | 4 | 0 | 0 | 346.981263 | 688.2312457 | 0 0 |
| hsa_circ_0087357 | 18 | 18 | 12 | 11 | 3122.831367 | 3097.040606 | 2531.64557 2553.98189 |
| hsa_circ_0087386 | 4 | 0 | 2 | 7 | 693.962526 | 0 | 421.9409283 1625.261203 |
| hsa_circ_0087391 | 7 | 9 | 0 | 5 | 1214.434421 | 1548.520303 | 0 1160.900859 |
| hsa_circ_0087605 | 0 | 6 | 5 | 4 | 0 | 1032.346869 | 1054.852321 928.7206873 |
| hsa_circ_0088030 | 10 | 19 | 9 | 16 | 1734.906315 | 3269.098417 | 1898.734177 3714.882749 |
| hsa_circ_0088300 | 0 | 0 | 6 | 2 | 0 | 0 | 1265.822785 464.3603436 |
| hsa_circ_0089254 | 2 | 2 | 0 | 0 | 346.981263 | 344.1156228 | 0 0 |
| hsa_circ_0089371 | 2 | 2 | 0 | 14 | 346.981263 | 344.1156228 | 0 3250.522405 |
| hsa_circ_0089466 | 0 | 0 | 3 | 3 | 0 | 0 | 632.9113924 696.5405154 |
| hsa_circ_0090162 | 5 | 7 | 6 | 2 | 867.4531575 | 1204.40468 | 1265.822785 464.3603436 |
| hsa_circ_0091065 | 0 | 0 | 4 | 2 | 0 | 0 | 843.8818565 464.3603436 |
| hsa_circ_0091187 | 6 | 9 | 8 | 4 | 1040.943789 | 1548.520303 | 1687.763713 928.7206873 |
| hsa_circ_0091483 | 2 | 5 | 2 | 0 | 346.981263 | 860.2890571 | 421.9409283 0 |
| hsa_circ_0091537 | 0 | 0 | 2 | 3 | 0 | 0 | 421.9409283 696.5405154 |
| novel_circ_0000067 | 3 | 11 | 0 | 0 | 520.4718945 | 1892.635926 | 0 0 |
| novel_circ_0000069 | 3 | 3 | 4 | 0 | 520.4718945 | 516.1734343 | 843.8818565 0 |
| novel_circ_0000079 | 2 | 0 | 4 | 3 | 346.981263 | 0 | 843.8818565 696.5405154 |
| novel_circ_0000089 | 10 | 4 | 6 | 4 | 1734.906315 | 688.2312457 | 1265.822785 928.7206873 |
| novel_circ_0000149 | 3 | 2 | 0 | 0 | 520.4718945 | 344.1156228 | 0 0 |
| novel_circ_0000225 | 2 | 2 | 0 | 0 | 346.981263 | 344.1156228 | 0 0 |
| novel_circ_0000267 | 2 | 2 | 0 | 0 | 346.981263 | 344.1156228 | 0 0 |
| novel_circ_0000306 | 5 | 0 | 3 | 2 | 867.4531575 | 0 | 632.9113924 464.3603436 |
| novel_circ_0000309 | 2 | 3 | 3 | 0 | 346.981263 | 516.1734343 | 632.9113924 0 |
| novel_circ_0000312 | 5 | 3 | 0 | 2 | 867.4531575 | 516.1734343 | 0 464.3603436 |

| novel_circ_0000329 | 5 | 4 | 6 | 0 | 867.4531575 | 688.2312457 | 1265.822785 0 |
| --- | --- | --- | --- | --- | --- | --- | --- |
| novel_circ_0000354 | 2 | 4 | 4 | 0 | 346.981263 | 688.2312457 | 843.8818565 0 |
| novel_circ_0000366 | 5 | 2 | 0 | 0 | 867.4531575 | 344.1156228 | 0 0 |
| novel_circ_0000369 | 4 | 2 | 2 | 0 | 693.962526 | 344.1156228 | 421.9409283 0 |
| novel_circ_0000380 | 3 | 6 | 7 | 10 | 520.4718945 | 1032.346869 | 1476.793249 2321.801718 |
| novel_circ_0000381 | 7 | 9 | 0 | 0 | 1214.434421 | 1548.520303 | 0 0 |
| novel_circ_0000426 | 2 | 2 | 0 | 4 | 346.981263 | 344.1156228 | 0 928.7206873 |
| novel_circ_0000434 | 4 | 6 | 12 | 6 | 693.962526 | 1032.346869 | 2531.64557 1393.081031 |
| novel_circ_0000491 | 2 | 3 | 0 | 0 | 346.981263 | 516.1734343 | 0 0 |
| novel_circ_0000503 | 7 | 2 | 2 | 2 | 1214.434421 | 344.1156228 | 421.9409283 464.3603436 |
| novel_circ_0000504 | 9 | 4 | 2 | 4 | 1561.415684 | 688.2312457 | 421.9409283 928.7206873 |
| novel_circ_0000515 | 2 | 5 | 0 | 0 | 346.981263 | 860.2890571 | 0 0 |
| novel_circ_0000542 | 3 | 4 | 3 | 0 | 520.4718945 | 688.2312457 | 632.9113924 0 |
| novel_circ_0000551 | 0 | 0 | 3 | 5 | 0 | 0 | 632.9113924 1160.900859 |
| novel_circ_0000555 | 8 | 0 | 2 | 2 | 1387.925052 | 0 | 421.9409283 464.3603436 |
| novel_circ_0000612 | 3 | 0 | 7 | 6 | 520.4718945 | 0 | 1476.793249 1393.081031 |
| novel_circ_0000622 | 4 | 2 | 2 | 4 | 693.962526 | 344.1156228 | 421.9409283 928.7206873 |
| novel_circ_0000626 | 14 | 6 | 5 | 4 | 2428.868841 | 1032.346869 | 1054.852321 928.7206873 |
| novel_circ_0000638 | 9 | 0 | 6 | 15 | 1561.415684 | 0 | 1265.822785 3482.702577 |
| novel_circ_0000640 | 0 | 0 | 3 | 13 | 0 | 0 | 632.9113924 3018.342234 |
| novel_circ_0000642 | 3 | 0 | 5 | 10 | 520.4718945 | 0 | 1054.852321 2321.801718 |
| novel_circ_0000644 | 0 | 0 | 2 | 2 | 0 | 0 | 421.9409283 464.3603436 |
| novel_circ_0000646 | 2 | 3 | 0 | 8 | 346.981263 | 516.1734343 | 0 1857.441375 |
| novel_circ_0000647 | 0 | 0 | 7 | 6 | 0 | 0 | 1476.793249 1393.081031 |
| novel_circ_0000648 | 0 | 4 | 7 | 9 | 0 | 688.2312457 | 1476.793249 2089.621546 |
| novel_circ_0000650 | 6 | 3 | 9 | 11 | 1040.943789 | 516.1734343 | 1898.734177 2553.98189 |
| novel_circ_0000651 | 0 | 6 | 8 | 14 | 0 | 1032.346869 | 1687.763713 3250.522405 |
| novel_circ_0000653 | 0 | 0 | 7 | 2 | 0 | 0 | 1476.793249 464.3603436 |
| novel_circ_0000656 | 4 | 6 | 10 | 2 | 693.962526 | 1032.346869 | 2109.704641 464.3603436 |
| novel_circ_0000660 | 0 | 0 | 5 | 3 | 0 | 0 | 1054.852321 696.5405154 |
| novel_circ_0000661 | 8 | 5 | 12 | 7 | 1387.925052 | 860.2890571 | 2531.64557 1625.261203 |
| novel_circ_0000687 | 6 | 2 | 0 | 0 | 1040.943789 | 344.1156228 | 0 0 |
| novel_circ_0000702 | 3 | 3 | 0 | 0 | 520.4718945 | 516.1734343 | 0 0 |
| novel_circ_0000711 | 4 | 6 | 3 | 3 | 693.962526 | 1032.346869 | 632.9113924 696.5405154 |
| novel_circ_0000725 | 0 | 0 | 2 | 2 | 0 | 0 | 421.9409283 464.3603436 |
| novel_circ_0000757 | 19 | 18 | 34 | 21 | 3296.321999 | 3097.040606 | 7172.995781 4875.783608 |
| novel_circ_0000824 | 4 | 7 | 2 | 3 | 693.962526 | 1204.40468 | 421.9409283 696.5405154 |
| novel_circ_0000846 | 5 | 2 | 0 | 0 | 867.4531575 | 344.1156228 | 0 0 |
| novel_circ_0000857 | 0 | 3 | 3 | 3 | 0 | 516.1734343 | 632.9113924 696.5405154 |
| novel_circ_0000861 | 2 | 2 | 0 | 0 | 346.981263 | 344.1156228 | 0 0 |
| novel_circ_0000863 | 3 | 5 | 7 | 3 | 520.4718945 | 860.2890571 | 1476.793249 696.5405154 |
| novel_circ_0000932 | 0 | 0 | 2 | 4 | 0 | 0 | 421.9409283 928.7206873 |
| novel_circ_0000973 | 2 | 2 | 0 | 0 | 346.981263 | 344.1156228 | 0 0 |
| novel_circ_0000982 | 13 | 5 | 0 | 0 | 2255.37821 | 860.2890571 | 0 0 |
| novel_circ_0001040 | 2 | 3 | 0 | 0 | 346.981263 | 516.1734343 | 0 0 |
| novel_circ_0001058 | 4 | 7 | 2 | 3 | 693.962526 | 1204.40468 | 421.9409283 696.5405154 |
| novel_circ_0001059 | 0 | 2 | 8 | 2 | 0 | 344.1156228 | 1687.763713 464.3603436 |
| novel_circ_0001063 | 8 | 6 | 6 | 3 | 1387.925052 | 1032.346869 | 1265.822785 696.5405154 |
| novel_circ_0001089 | 6 | 8 | 0 | 0 | 1040.943789 | 1376.462491 | 0 0 |
| novel_circ_0001101 | 0 | 0 | 5 | 7 | 0 | 0 | 1054.852321 1625.261203 |
| novel_circ_0001102 | 0 | 0 | 10 | 3 | 0 | 0 | 2109.704641 696.5405154 |
| novel_circ_0001107 | 5 | 4 | 2 | 2 | 867.4531575 | 688.2312457 | 421.9409283 464.3603436 |
| novel_circ_0001112 | 13 | 22 | 7 | 9 | 2255.37821 | 3785.271851 | 1476.793249 2089.621546 |
| novel_circ_0001179 | 2 | 3 | 0 | 0 | 346.981263 | 516.1734343 | 0 0 |
| novel_circ_0001187 | 4 | 3 | 0 | 0 | 693.962526 | 516.1734343 | 0 0 |
| novel_circ_0001228 | 6 | 0 | 3 | 2 | 1040.943789 | 0 | 632.9113924 464.3603436 |
| novel_circ_0001258 | 11 | 6 | 2 | 0 | 1908.396947 | 1032.346869 | 421.9409283 0 |
| novel_circ_0001266 | 3 | 2 | 0 | 0 | 520.4718945 | 344.1156228 | 0 0 |
| novel_circ_0001278 | 3 | 4 | 0 | 0 | 520.4718945 | 688.2312457 | 0 0 |
| novel_circ_0001283 | 0 | 0 | 3 | 2 | 0 | 0 | 632.9113924 464.3603436 |
| novel_circ_0001295 | 3 | 5 | 0 | 0 | 520.4718945 | 860.2890571 | 0 0 |
| novel_circ_0001298 | 0 | 5 | 3 | 3 | 0 | 860.2890571 | 632.9113924 696.5405154 |
| novel_circ_0001322 | 6 | 3 | 0 | 0 | 1040.943789 | 516.1734343 | 0 0 |
| novel_circ_0001329 | 4 | 5 | 4 | 2 | 693.962526 | 860.2890571 | 843.8818565 464.3603436 |
| novel_circ_0001331 | 0 | 0 | 28 | 2 | 0 | 0 | 5907.172996 464.3603436 |
| novel_circ_0001347 | 2 | 3 | 3 | 4 | 346.981263 | 516.1734343 | 632.9113924 928.7206873 |
| novel_circ_0001361 | 3 | 4 | 0 | 0 | 520.4718945 | 688.2312457 | 0 0 |
| novel_circ_0001369 | 0 | 2 | 2 | 4 | 0 | 344.1156228 | 421.9409283 928.7206873 |
| novel_circ_0001378 | 0 | 0 | 5 | 5 | 0 | 0 | 1054.852321 1160.900859 |
| novel_circ_0001382 | 0 | 3 | 2 | 2 | 0 | 516.1734343 | 421.9409283 464.3603436 |
| novel_circ_0001422 | 4 | 3 | 3 | 0 | 693.962526 | 516.1734343 | 632.9113924 0 |
| novel_circ_0001470 | 5 | 0 | 2 | 3 | 867.4531575 | 0 | 421.9409283 696.5405154 |
| novel_circ_0001476 | 13 | 23 | 21 | 0 | 2255.37821 | 3957.329663 | 4430.379747 0 |
| novel_circ_0001477 | 7 | 11 | 10 | 2 | 1214.434421 | 1892.635926 | 2109.704641 464.3603436 |
| novel_circ_0001485 | 27 | 28 | 36 | 24 | 4684.247051 | 4817.61872 | 7594.936709 5572.324124 |
| novel_circ_0001513 | 2 | 4 | 0 | 0 | 346.981263 | 688.2312457 | 0 0 |
| novel_circ_0001515 | 4 | 5 | 5 | 6 | 693.962526 | 860.2890571 | 1054.852321 1393.081031 |

| novel_circ_0001587 | 0 | 0 | 3 | 4 | 0 | 0 | 632.9113924 928.7206873 |
| --- | --- | --- | --- | --- | --- | --- | --- |
| novel_circ_0001588 | 2 | 4 | 0 | 0 | 346.981263 | 688.2312457 | 0 0 |
| novel_circ_0001651 | 6 | 5 | 7 | 6 | 1040.943789 | 860.2890571 | 1476.793249 1393.081031 |
| novel_circ_0001716 | 6 | 4 | 10 | 7 | 1040.943789 | 688.2312457 | 2109.704641 1625.261203 |
| novel_circ_0001717 | 0 | 5 | 9 | 2 | 0 | 860.2890571 | 1898.734177 464.3603436 |
| novel_circ_0001748 | 3 | 6 | 0 | 0 | 520.4718945 | 1032.346869 | 0 0 |
| novel_circ_0001763 | 7 | 2 | 0 | 2 | 1214.434421 | 344.1156228 | 0 464.3603436 |
| novel_circ_0001783 | 3 | 3 | 0 | 0 | 520.4718945 | 516.1734343 | 0 0 |
| novel_circ_0001789 | 5 | 12 | 4 | 6 | 867.4531575 | 2064.693737 | 843.8818565 1393.081031 |
| novel_circ_0001790 | 6 | 5 | 0 | 0 | 1040.943789 | 860.2890571 | 0 0 |
| novel_circ_0001827 | 3 | 5 | 6 | 0 | 520.4718945 | 860.2890571 | 1265.822785 0 |
| novel_circ_0001867 | 2 | 5 | 2 | 2 | 346.981263 | 860.2890571 | 421.9409283 464.3603436 |
| novel_circ_0001874 | 5 | 4 | 2 | 0 | 867.4531575 | 688.2312457 | 421.9409283 0 |
| novel_circ_0001881 | 5 | 2 | 4 | 3 | 867.4531575 | 344.1156228 | 843.8818565 696.5405154 |
| novel_circ_0001973 | 7 | 2 | 8 | 3 | 1214.434421 | 344.1156228 | 1687.763713 696.5405154 |
| novel_circ_0001975 | 10 | 10 | 2 | 7 | 1734.906315 | 1720.578114 | 421.9409283 1625.261203 |
| novel_circ_0002053 | 6 | 5 | 7 | 0 | 1040.943789 | 860.2890571 | 1476.793249 0 |
| novel_circ_0002066 | 7 | 7 | 5 | 4 | 1214.434421 | 1204.40468 | 1054.852321 928.7206873 |
| novel_circ_0002102 | 9 | 3 | 0 | 0 | 1561.415684 | 516.1734343 | 0 0 |
| novel_circ_0002121 | 3 | 3 | 3 | 2 | 520.4718945 | 516.1734343 | 632.9113924 464.3603436 |
| novel_circ_0002123 | 0 | 0 | 4 | 2 | 0 | 0 | 843.8818565 464.3603436 |
| novel_circ_0002129 | 4 | 6 | 8 | 5 | 693.962526 | 1032.346869 | 1687.763713 1160.900859 |
| novel_circ_0002151 | 5 | 6 | 5 | 0 | 867.4531575 | 1032.346869 | 1054.852321 0 |
| novel_circ_0002297 | 3 | 3 | 0 | 0 | 520.4718945 | 516.1734343 | 0 0 |
| novel_circ_0002298 | 25 | 17 | 6 | 6 | 4337.265788 | 2924.982794 | 1265.822785 1393.081031 |
| novel_circ_0002300 | 28 | 26 | 40 | 14 | 4857.737682 | 4473.503097 | 8438.818565 3250.522405 |
| novel_circ_0002310 | 4 | 4 | 0 | 0 | 693.962526 | 688.2312457 | 0 0 |
| novel_circ_0002319 | 4 | 3 | 11 | 0 | 693.962526 | 516.1734343 | 2320.675105 0 |
| novel_circ_0002337 | 20 | 24 | 15 | 14 | 3469.81263 | 4129.387474 | 3164.556962 3250.522405 |
| novel_circ_0002393 | 0 | 4 | 6 | 11 | 0 | 688.2312457 | 1265.822785 2553.98189 |
| novel_circ_0002405 | 4 | 7 | 2 | 0 | 693.962526 | 1204.40468 | 421.9409283 0 |
| novel_circ_0002450 | 0 | 8 | 2 | 6 | 0 | 1376.462491 | 421.9409283 1393.081031 |
| novel_circ_0002468 | 2 | 2 | 0 | 2 | 346.981263 | 344.1156228 | 0 464.3603436 |
| novel_circ_0002470 | 9 | 5 | 0 | 2 | 1561.415684 | 860.2890571 | 0 464.3603436 |
| novel_circ_0002541 | 4 | 0 | 6 | 2 | 693.962526 | 0 | 1265.822785 464.3603436 |
| novel_circ_0002546 | 83 | 83 | 0 | 59 | 14399.72241 | 14280.79835 | 0 13698.63014 |
| novel_circ_0002554 | 0 | 3 | 2 | 2 | 0 | 516.1734343 | 421.9409283 464.3603436 |
| novel_circ_0002623 | 6 | 3 | 4 | 2 | 1040.943789 | 516.1734343 | 843.8818565 464.3603436 |
| novel_circ_0002642 | 6 | 5 | 0 | 4 | 1040.943789 | 860.2890571 | 0 928.7206873 |
| novel_circ_0002645 | 8 | 18 | 7 | 4 | 1387.925052 | 3097.040606 | 1476.793249 928.7206873 |
| novel_circ_0002667 | 6 | 8 | 2 | 0 | 1040.943789 | 1376.462491 | 421.9409283 0 |
| novel_circ_0002684 | 3 | 6 | 0 | 0 | 520.4718945 | 1032.346869 | 0 0 |
| novel_circ_0002697 | 5 | 4 | 2 | 4 | 867.4531575 | 688.2312457 | 421.9409283 928.7206873 |
| novel_circ_0002698 | 22 | 22 | 16 | 12 | 3816.793893 | 3785.271851 | 3375.527426 2786.162062 |
| novel_circ_0002717 | 0 | 2 | 2 | 3 | 0 | 344.1156228 | 421.9409283 696.5405154 |
| novel_circ_0002721 | 19 | 17 | 0 | 2 | 3296.321999 | 2924.982794 | 0 464.3603436 |
| novel_circ_0002771 | 0 | 3 | 3 | 2 | 0 | 516.1734343 | 632.9113924 464.3603436 |
| novel_circ_0002795 | 8 | 5 | 5 | 0 | 1387.925052 | 860.2890571 | 1054.852321 0 |
| novel_circ_0002797 | 2 | 6 | 0 | 0 | 346.981263 | 1032.346869 | 0 0 |
| novel_circ_0002798 | 0 | 0 | 2 | 2 | 0 | 0 | 421.9409283 464.3603436 |
| novel_circ_0002880 | 18 | 12 | 12 | 10 | 3122.831367 | 2064.693737 | 2531.64557 2321.801718 |
| novel_circ_0002883 | 7 | 4 | 11 | 9 | 1214.434421 | 688.2312457 | 2320.675105 2089.621546 |
| novel_circ_0002905 | 24 | 17 | 15 | 2 | 4163.775156 | 2924.982794 | 3164.556962 464.3603436 |
| novel_circ_0002935 | 5 | 4 | 0 | 2 | 867.4531575 | 688.2312457 | 0 464.3603436 |
| novel_circ_0002937 | 8 | 3 | 4 | 0 | 1387.925052 | 516.1734343 | 843.8818565 0 |
| novel_circ_0002964 | 5 | 10 | 5 | 3 | 867.4531575 | 1720.578114 | 1054.852321 696.5405154 |
| novel_circ_0002976 | 0 | 4 | 6 | 8 | 0 | 688.2312457 | 1265.822785 1857.441375 |
| novel_circ_0002990 | 2 | 11 | 12 | 8 | 346.981263 | 1892.635926 | 2531.64557 1857.441375 |
| novel_circ_0002993 | 2 | 0 | 2 | 3 | 346.981263 | 0 | 421.9409283 696.5405154 |
| novel_circ_0002996 | 0 | 0 | 3 | 5 | 0 | 0 | 632.9113924 1160.900859 |
| novel_circ_0002997 | 2 | 3 | 13 | 8 | 346.981263 | 516.1734343 | 2742.616034 1857.441375 |
| novel_circ_0002998 | 2 | 6 | 3 | 4 | 346.981263 | 1032.346869 | 632.9113924 928.7206873 |
| novel_circ_0003002 | 3 | 4 | 3 | 2 | 520.4718945 | 688.2312457 | 632.9113924 464.3603436 |
| novel_circ_0003031 | 9 | 4 | 8 | 6 | 1561.415684 | 688.2312457 | 1687.763713 1393.081031 |
| novel_circ_0003033 | 4 | 2 | 3 | 0 | 693.962526 | 344.1156228 | 632.9113924 0 |
| novel_circ_0003052 | 0 | 0 | 4 | 2 | 0 | 0 | 843.8818565 464.3603436 |
| novel_circ_0003098 | 0 | 0 | 5 | 4 | 0 | 0 | 1054.852321 928.7206873 |
| novel_circ_0003103 | 0 | 2 | 2 | 2 | 0 | 344.1156228 | 421.9409283 464.3603436 |
| novel_circ_0003133 | 3 | 4 | 3 | 0 | 520.4718945 | 688.2312457 | 632.9113924 0 |
| novel_circ_0003148 | 3 | 2 | 0 | 2 | 520.4718945 | 344.1156228 | 0 464.3603436 |
| novel_circ_0003207 | 3 | 4 | 5 | 4 | 520.4718945 | 688.2312457 | 1054.852321 928.7206873 |
| novel_circ_0003210 | 0 | 3 | 3 | 6 | 0 | 516.1734343 | 632.9113924 1393.081031 |
| novel_circ_0003219 | 0 | 0 | 2 | 2 | 0 | 0 | 421.9409283 464.3603436 |
| novel_circ_0003222 | 14 | 9 | 13 | 3 | 2428.868841 | 1548.520303 | 2742.616034 696.5405154 |
| novel_circ_0003242 | 0 | 6 | 3 | 3 | 0 | 1032.346869 | 632.9113924 696.5405154 |
| novel_circ_0003249 | 3 | 5 | 0 | 0 | 520.4718945 | 860.2890571 | 0 0 |
| novel_circ_0003273 | 4 | 0 | 4 | 3 | 693.962526 | 0 | 843.8818565 696.5405154 |

| novel_circ_0003281 | 2 | 2 | 0 | 0 | 346.981263 | 344.1156228 | 0 0 |
| --- | --- | --- | --- | --- | --- | --- | --- |
| novel_circ_0003283 | 4 | 0 | 2 | 3 | 693.962526 | 0 | 421.9409283 696.5405154 |
| novel_circ_0003290 | 0 | 0 | 2 | 3 | 0 | 0 | 421.9409283 696.5405154 |
| novel_circ_0003292 | 3 | 4 | 7 | 14 | 520.4718945 | 688.2312457 | 1476.793249 3250.522405 |
| novel_circ_0003295 | 0 | 5 | 3 | 3 | 0 | 860.2890571 | 632.9113924 696.5405154 |
| novel_circ_0003302 | 15 | 20 | 3 | 5 | 2602.359473 | 3441.156228 | 632.9113924 1160.900859 |
| novel_circ_0003310 | 3 | 2 | 0 | 0 | 520.4718945 | 344.1156228 | 0 0 |
| novel_circ_0003318 | 5 | 2 | 0 | 0 | 867.4531575 | 344.1156228 | 0 0 |
