# Supplementary material for: Identification of Novel Multi-Omics Expression Landscapes and Meta-Analysis of Landscape-Based Competitive Endogenous RNA Networks in ALDH+ Lung Adenocarcinoma Stem Cells
Source: Biomed Res Int. 2022 Aug 31;2022:9545609. doi: 10.1155/2022/9545609 (PMC9453044; doi:10.1155/2022/9545609)
Supplement: Supplementary Materials — The supporting information includes the following: Figure S1. First base bias and position base bias of novel miRNAs; Table S1. Detailed information of miRNAs; Table S2. Novel lncRNAs; Table S3. Novel miRNAs; Table S4. Novel circRNAs; Table S5. Information on ceRNA-based molecular interactions and pairs. [file 9545609.f1.zip › Table_S5_ceRNA_based molecular information and pairs information.doc]

# Table 5.1:node information

**ID1	ID2	interaction**

| hsa-let-7g-5p | hsa_circ_0006727 | lncRNA-miRNA |
| --- | --- | --- |
| hsa-let-7g-5p | hsa_circ_0038816 | lncRNA-miRNA |
| hsa-let-7g-5p | hsa_circ_0052943 | lncRNA-miRNA |
| hsa-miR-122-5p | hsa_circ_0002762 | lncRNA-miRNA |
| hsa-miR-122-5p | hsa_circ_0007788 | lncRNA-miRNA |
| hsa-miR-122-5p | hsa_circ_0008351 | lncRNA-miRNA |
| hsa-miR-122-5p | hsa_circ_0015260 | lncRNA-miRNA |
| hsa-miR-122b-3p | hsa_circ_0001481 | lncRNA-miRNA |
| hsa-miR-122b-3p | hsa_circ_0002750 | lncRNA-miRNA |
| hsa-miR-122b-3p | hsa_circ_0018524 | lncRNA-miRNA |
| hsa-miR-122b-3p | hsa_circ_0048000 | lncRNA-miRNA |
| hsa-miR-122b-3p | novel_circ_0000381 | lncRNA-miRNA |
| hsa-miR-1268a | hsa_circ_0001575 | lncRNA-miRNA |
| hsa-miR-1268a | hsa_circ_0006727 | lncRNA-miRNA |
| hsa-miR-1287-5p | hsa_circ_0001417 | lncRNA-miRNA |
| hsa-miR-1287-5p | hsa_circ_0006727 | lncRNA-miRNA |
| hsa-miR-1287-5p | hsa_circ_0007804 | lncRNA-miRNA |
| hsa-miR-1293 | hsa_circ_0002750 | lncRNA-miRNA |
| hsa-miR-1293 | hsa_circ_0007178 | lncRNA-miRNA |
| hsa-miR-1293 | hsa_circ_0008351 | lncRNA-miRNA |
| hsa-miR-1307-3p | novel_circ_0000982 | lncRNA-miRNA |
| hsa-miR-130a-5p | hsa_circ_0001358 | lncRNA-miRNA |
| hsa-miR-130a-5p | hsa_circ_0006087 | lncRNA-miRNA |
| hsa-miR-130a-5p | hsa_circ_0006727 | lncRNA-miRNA |
| hsa-miR-130a-5p | hsa_circ_0013167 | lncRNA-miRNA |
| hsa-miR-130a-5p | hsa_circ_0014577 | lncRNA-miRNA |
| hsa-miR-130a-5p | hsa_circ_0073904 | lncRNA-miRNA |
| hsa-miR-130a-5p | hsa_circ_0091537 | lncRNA-miRNA |
| hsa-miR-130b-3p | hsa_circ_0001575 | lncRNA-miRNA |
| hsa-miR-130b-3p | hsa_circ_0003045 | lncRNA-miRNA |
| hsa-miR-130b-3p | hsa_circ_0006010 | lncRNA-miRNA |
| hsa-miR-130b-3p | hsa_circ_0006727 | lncRNA-miRNA |
| hsa-miR-130b-3p | hsa_circ_0052943 | lncRNA-miRNA |
| hsa-miR-141-3p | hsa_circ_0002750 | lncRNA-miRNA |
| hsa-miR-141-3p | hsa_circ_0002762 | lncRNA-miRNA |
| hsa-miR-141-3p | hsa_circ_0042498 | lncRNA-miRNA |
| hsa-miR-143-3p | hsa_circ_0003322 | lncRNA-miRNA |
| hsa-miR-143-3p | hsa_circ_0018992 | lncRNA-miRNA |
| hsa-miR-146b-5p | hsa_circ_0002348 | lncRNA-miRNA |
| hsa-miR-146b-5p | hsa_circ_0006360 | lncRNA-miRNA |
| hsa-miR-146b-5p | hsa_circ_0061774 | lncRNA-miRNA |
| hsa-miR-146b-5p | hsa_circ_0061776 | lncRNA-miRNA |
| hsa-miR-147b-3p | hsa_circ_0005309 | lncRNA-miRNA |
| hsa-miR-147b-3p | hsa_circ_0007788 | lncRNA-miRNA |
| hsa-miR-148a-3p | hsa_circ_0003102 | lncRNA-miRNA |
| hsa-miR-148a-3p | hsa_circ_0003322 | lncRNA-miRNA |
| hsa-miR-148a-3p | hsa_circ_0008351 | lncRNA-miRNA |
| hsa-miR-148a-3p | hsa_circ_0062642 | lncRNA-miRNA |

| hsa-miR-148a-3p | novel_circ_0003249 | lncRNA-miRNA |
| --- | --- | --- |
| hsa-miR-148b-3p | hsa_circ_0003045 | lncRNA-miRNA |
| hsa-miR-148b-3p | hsa_circ_0006727 | lncRNA-miRNA |
| hsa-miR-148b-3p | hsa_circ_0052943 | lncRNA-miRNA |
| hsa-miR-181b-3p | hsa_circ_0001575 | lncRNA-miRNA |
| hsa-miR-181b-3p | hsa_circ_0006727 | lncRNA-miRNA |
| hsa-miR-181b-3p | hsa_circ_0008798 | lncRNA-miRNA |
| hsa-miR-181b-3p | hsa_circ_0014577 | lncRNA-miRNA |
| hsa-miR-181b-3p | hsa_circ_0089466 | lncRNA-miRNA |
| hsa-miR-183-5p | hsa_circ_0018524 | lncRNA-miRNA |
| hsa-miR-183-5p | hsa_circ_0069101 | lncRNA-miRNA |
| hsa-miR-1843 | hsa_circ_0008351 | lncRNA-miRNA |
| hsa-miR-193b-3p | hsa_circ_0001481 | lncRNA-miRNA |
| hsa-miR-193b-3p | hsa_circ_0003221 | lncRNA-miRNA |
| hsa-miR-193b-3p | hsa_circ_0008351 | lncRNA-miRNA |
| hsa-miR-195-5p | hsa_circ_0001417 | lncRNA-miRNA |
| hsa-miR-195-5p | hsa_circ_0001575 | lncRNA-miRNA |
| hsa-miR-195-5p | hsa_circ_0006727 | lncRNA-miRNA |
| hsa-miR-195-5p | hsa_circ_0009110 | lncRNA-miRNA |
| hsa-miR-195-5p | hsa_circ_0052943 | lncRNA-miRNA |
| hsa-miR-195-5p | novel_circ_0000551 | lncRNA-miRNA |
| hsa-miR-195-5p | novel_circ_0000932 | lncRNA-miRNA |
| hsa-miR-196a-5p | hsa_circ_0073706 | lncRNA-miRNA |
| hsa-miR-196b-5p | hsa_circ_0073706 | lncRNA-miRNA |
| hsa-miR-19b-3p | hsa_circ_0001417 | lncRNA-miRNA |
| hsa-miR-19b-3p | hsa_circ_0001575 | lncRNA-miRNA |
| hsa-miR-19b-3p | hsa_circ_0052943 | lncRNA-miRNA |
| hsa-miR-200c-3p | hsa_circ_0001315 | lncRNA-miRNA |
| hsa-miR-200c-3p | hsa_circ_0003221 | lncRNA-miRNA |
| hsa-miR-200c-3p | hsa_circ_0003694 | lncRNA-miRNA |
| hsa-miR-200c-3p | hsa_circ_0007001 | lncRNA-miRNA |
| hsa-miR-200c-3p | hsa_circ_0008351 | lncRNA-miRNA |
| hsa-miR-212-5p | hsa_circ_0002348 | lncRNA-miRNA |
| hsa-miR-216b-5p | hsa_circ_0042498 | lncRNA-miRNA |
| hsa-miR-216b-5p | novel_circ_0000067 | lncRNA-miRNA |
| hsa-miR-221-5p | hsa_circ_0001575 | lncRNA-miRNA |
| hsa-miR-221-5p | hsa_circ_0006727 | lncRNA-miRNA |
| hsa-miR-221-5p | hsa_circ_0008075 | lncRNA-miRNA |
| hsa-miR-22-3p | hsa_circ_0003221 | lncRNA-miRNA |
| hsa-miR-22-3p | hsa_circ_0003909 | lncRNA-miRNA |
| hsa-miR-22-3p | hsa_circ_0041152 | lncRNA-miRNA |
| hsa-miR-22-3p | hsa_circ_0079040 | lncRNA-miRNA |
| hsa-miR-22-5p | hsa_circ_0001481 | lncRNA-miRNA |
| hsa-miR-22-5p | hsa_circ_0002750 | lncRNA-miRNA |
| hsa-miR-22-5p | hsa_circ_0007701 | lncRNA-miRNA |
| hsa-miR-22-5p | hsa_circ_0009008 | lncRNA-miRNA |
| hsa-miR-22-5p | novel_circ_0000067 | lncRNA-miRNA |
| hsa-miR-22-5p | novel_circ_0002102 | lncRNA-miRNA |
| hsa-miR-27b-3p | hsa_circ_0001051 | lncRNA-miRNA |
| hsa-miR-27b-3p | hsa_circ_0001575 | lncRNA-miRNA |
| hsa-miR-27b-3p | hsa_circ_0052943 | lncRNA-miRNA |

| hsa-miR-3129-3p | hsa_circ_0001575 | lncRNA-miRNA |
| --- | --- | --- |
| hsa-miR-3129-5p | hsa_circ_0001358 | lncRNA-miRNA |
| hsa-miR-3129-5p | hsa_circ_0001575 | lncRNA-miRNA |
| hsa-miR-3129-5p | hsa_circ_0006727 | lncRNA-miRNA |
| hsa-miR-3129-5p | hsa_circ_0013167 | lncRNA-miRNA |
| hsa-miR-3129-5p | hsa_circ_0014577 | lncRNA-miRNA |
| hsa-miR-3144-3p | hsa_circ_0001575 | lncRNA-miRNA |
| hsa-miR-3144-3p | hsa_circ_0006727 | lncRNA-miRNA |
| hsa-miR-330-5p | hsa_circ_0005898 | lncRNA-miRNA |
| hsa-miR-330-5p | hsa_circ_0007001 | lncRNA-miRNA |
| hsa-miR-330-5p | hsa_circ_0007178 | lncRNA-miRNA |
| hsa-miR-330-5p | novel_circ_0003249 | lncRNA-miRNA |
| hsa-miR-331-5p | hsa_circ_0003221 | lncRNA-miRNA |
| hsa-miR-331-5p | hsa_circ_0008694 | lncRNA-miRNA |
| hsa-miR-331-5p | novel_circ_0001089 | lncRNA-miRNA |
| hsa-miR-331-5p | novel_circ_0002797 | lncRNA-miRNA |
| hsa-miR-3615 | hsa_circ_0069101 | lncRNA-miRNA |
| hsa-miR-361-5p | hsa_circ_0001417 | lncRNA-miRNA |
| hsa-miR-361-5p | hsa_circ_0001575 | lncRNA-miRNA |
| hsa-miR-361-5p | hsa_circ_0006117 | lncRNA-miRNA |
| hsa-miR-361-5p | hsa_circ_0006727 | lncRNA-miRNA |
| hsa-miR-361-5p | novel_circ_0000551 | lncRNA-miRNA |
| hsa-miR-365b-5p | hsa_circ_0001481 | lncRNA-miRNA |
| hsa-miR-365b-5p | hsa_circ_0003322 | lncRNA-miRNA |
| hsa-miR-365b-5p | hsa_circ_0015260 | lncRNA-miRNA |
| hsa-miR-374a-3p | hsa_circ_0006393 | lncRNA-miRNA |
| hsa-miR-374a-5p | hsa_circ_0001575 | lncRNA-miRNA |
| hsa-miR-3909 | hsa_circ_0018524 | lncRNA-miRNA |
| hsa-miR-3909 | hsa_circ_0048000 | lncRNA-miRNA |
| hsa-miR-3909 | novel_circ_0003249 | lncRNA-miRNA |
| hsa-miR-3913-3p | hsa_circ_0001098 | lncRNA-miRNA |
| hsa-miR-3913-3p | hsa_circ_0002348 | lncRNA-miRNA |
| hsa-miR-3913-3p | hsa_circ_0002750 | lncRNA-miRNA |
| hsa-miR-3913-3p | hsa_circ_0003221 | lncRNA-miRNA |
| hsa-miR-3913-3p | hsa_circ_0048000 | lncRNA-miRNA |
| hsa-miR-3913-3p | hsa_circ_0058051 | lncRNA-miRNA |
| hsa-miR-3913-3p | hsa_circ_0069101 | lncRNA-miRNA |
| hsa-miR-3913-5p | hsa_circ_0007001 | lncRNA-miRNA |
| hsa-miR-3913-5p | hsa_circ_0008351 | lncRNA-miRNA |
| hsa-miR-3913-5p | hsa_circ_0009064 | lncRNA-miRNA |
| hsa-miR-3913-5p | hsa_circ_0015260 | lncRNA-miRNA |
| hsa-miR-3913-5p | hsa_circ_0018524 | lncRNA-miRNA |
| hsa-miR-3913-5p | novel_circ_0000687 | lncRNA-miRNA |
| hsa-miR-3940-3p | hsa_circ_0006727 | lncRNA-miRNA |
| hsa-miR-424-5p | hsa_circ_0001417 | lncRNA-miRNA |
| hsa-miR-424-5p | hsa_circ_0001575 | lncRNA-miRNA |
| hsa-miR-424-5p | hsa_circ_0006727 | lncRNA-miRNA |
| hsa-miR-424-5p | hsa_circ_0009110 | lncRNA-miRNA |
| hsa-miR-424-5p | hsa_circ_0052943 | lncRNA-miRNA |
| hsa-miR-424-5p | novel_circ_0000551 | lncRNA-miRNA |
| hsa-miR-424-5p | novel_circ_0000932 | lncRNA-miRNA |

hsa-miR-425-5p hsa_circ_0018992 lncRNA-miRNA hsa-miR-4521 hsa_circ_0001575 lncRNA-miRNA hsa-miR-4521 hsa_circ_0006727 lncRNA-miRNA hsa-miR-4529-5p hsa_circ_0005309 lncRNA-miRNA hsa-miR-455-5p hsa_circ_0018992 lncRNA-miRNA hsa-miR-532-5p hsa_circ_0000629 lncRNA-miRNA hsa-miR-532-5p hsa_circ_0001098 lncRNA-miRNA hsa-miR-532-5p hsa_circ_0008351 lncRNA-miRNA hsa-miR-532-5p hsa_circ_0058051 lncRNA-miRNA hsa-miR-548ah-3p hsa_circ_0001481 lncRNA-miRNA hsa-miR-548ah-3p novel_circ_0000687 lncRNA-miRNA hsa-miR-548d-5p novel_circ_0000640 lncRNA-miRNA hsa-miR-548f-3p hsa_circ_0018992 lncRNA-miRNA hsa-miR-548f-3p hsa_circ_0042498 lncRNA-miRNA hsa-miR-548n hsa_circ_0001575 lncRNA-miRNA hsa-miR-548n novel_circ_0000640 lncRNA-miRNA hsa-miR-548n novel_circ_0001378 lncRNA-miRNA hsa-miR-548t-3p hsa_circ_0001417 lncRNA-miRNA hsa-miR-548t-3p hsa_circ_0001575 lncRNA-miRNA hsa-miR-548t-3p hsa_circ_0006727 lncRNA-miRNA hsa-miR-548t-3p hsa_circ_0081808 lncRNA-miRNA hsa-miR-548t-3p novel_circ_0000647 lncRNA-miRNA hsa-miR-550a-3p hsa_circ_0014292 lncRNA-miRNA hsa-miR-550b-2-5phsa_circ_0001575 lncRNA-miRNA hsa-miR-550b-2-5phsa_circ_0006727 lncRNA-miRNA hsa-miR-551b-5p hsa_circ_0007178 lncRNA-miRNA hsa-miR-551b-5p hsa_circ_0007976 lncRNA-miRNA hsa-miR-551b-5p hsa_circ_0009064 lncRNA-miRNA hsa-miR-551b-5p hsa_circ_0015260 lncRNA-miRNA hsa-miR-580-3p hsa_circ_0005581 lncRNA-miRNA hsa-miR-580-3p hsa_circ_0006010 lncRNA-miRNA hsa-miR-580-3p hsa_circ_0009110 lncRNA-miRNA hsa-miR-580-3p novel_circ_0001101 lncRNA-miRNA hsa-miR-580-3p novel_circ_0001102 lncRNA-miRNA hsa-miR-628-5p hsa_circ_0001575 lncRNA-miRNA hsa-miR-628-5p hsa_circ_0006727 lncRNA-miRNA hsa-miR-628-5p hsa_circ_0008433 lncRNA-miRNA hsa-miR-769-5p hsa_circ_0003322 lncRNA-miRNA hsa-miR-769-5p hsa_circ_0003694 lncRNA-miRNA hsa-miR-769-5p hsa_circ_0079040 lncRNA-miRNA hsa-miR-933 hsa_circ_0003877 lncRNA-miRNA hsa-miR-933 hsa_circ_0015260 lncRNA-miRNA hsa-miR-942-5p hsa_circ_0001575 lncRNA-miRNA hsa-miR-942-5p hsa_circ_0006727 lncRNA-miRNA hsa-miR-942-5p hsa_circ_0013167 lncRNA-miRNA hsa-miR-942-5p hsa_circ_0014577 lncRNA-miRNA hsa-miR-942-5p novel_circ_0001283 lncRNA-miRNA hsa-miR-96-5p hsa_circ_0002750 lncRNA-miRNA hsa-miR-96-5p hsa_circ_0003102 lncRNA-miRNA hsa-miR-96-5p hsa_circ_0003877 lncRNA-miRNA hsa-miR-96-5p hsa_circ_0008351 lncRNA-miRNA

| hsa-miR-96-5p | hsa_circ_0015260 | lncRNA-miRNA |
| --- | --- | --- |
| hsa-miR-96-5p | hsa_circ_0062642 | lncRNA-miRNA |
| DNASE1 | hsa-miR-3909 | mRNA-miRNA |
| HPD | hsa-miR-1268a | mRNA-miRNA |
| CATIP | hsa-miR-3940-3p | mRNA-miRNA |
| MRC2 | hsa-miR-330-5p | mRNA-miRNA |
| ECEL1 | hsa-miR-1268a | mRNA-miRNA |
| CST2 | hsa-miR-942-5p | mRNA-miRNA |
| FHAD1 | hsa-miR-200c-3p | mRNA-miRNA |
| ZNF446 | hsa-miR-769-5p | mRNA-miRNA |
| PTP4A3 | hsa-miR-330-5p | mRNA-miRNA |
| TUFT1 | hsa-miR-330-5p | mRNA-miRNA |
| FLNA | hsa-miR-1293 | mRNA-miRNA |
| ORC1 | hsa-miR-550b-2-5p | mRNA-miRNA |
| DAB2IP | hsa-miR-330-5p | mRNA-miRNA |
| ZC4H2 | hsa-miR-4521 | mRNA-miRNA |
| ACSF3 | hsa-miR-193b-3p | mRNA-miRNA |
| ACSF3 | hsa-miR-330-5p | mRNA-miRNA |
| BMPR1B | hsa-miR-942-5p | mRNA-miRNA |
| SVEP1 | hsa-miR-942-5p | mRNA-miRNA |
| PES1 | hsa-miR-3909 | mRNA-miRNA |
| CALCRL | hsa-miR-1287-5p | mRNA-miRNA |
| CAPRIN2 | hsa-miR-1287-5p | mRNA-miRNA |
| ARL6IP4 | hsa-miR-212-5p | mRNA-miRNA |
| TCOF1 | hsa-miR-1287-5p | mRNA-miRNA |
| CLCN2 | hsa-miR-22-3p | mRNA-miRNA |
| EGR4 | hsa-miR-1268a | mRNA-miRNA |
| RASAL1 | hsa-miR-130b-3p | mRNA-miRNA |
| CLCN7 | hsa-miR-3940-3p | mRNA-miRNA |
| FAM160A2 | hsa-miR-3940-3p | mRNA-miRNA |
| IMPDH1 | hsa-miR-769-5p | mRNA-miRNA |
| TENM2 | hsa-miR-365b-5p | mRNA-miRNA |
| CD151 | hsa-miR-933 | mRNA-miRNA |
| C11orf96 | hsa-miR-3940-3p | mRNA-miRNA |
| NPEPPS | hsa-miR-195-5p | mRNA-miRNA |
| MT1X | hsa-miR-330-5p | mRNA-miRNA |
| MT1X | hsa-miR-769-5p | mRNA-miRNA |
| ADGRG1 | hsa-miR-3940-3p | mRNA-miRNA |
| PLD2 | hsa-miR-3909 | mRNA-miRNA |
| CEP131 | hsa-miR-330-5p | mRNA-miRNA |
| CEP131 | hsa-miR-3909 | mRNA-miRNA |
| TBCB | hsa-miR-221-5p | mRNA-miRNA |
| CTDP1 | hsa-miR-96-5p | mRNA-miRNA |
| KCTD17 | hsa-miR-365b-5p | mRNA-miRNA |
| DHRS3 | hsa-miR-193b-3p | mRNA-miRNA |
| ARFRP1 | hsa-miR-330-5p | mRNA-miRNA |
| ARFRP1 | hsa-miR-365b-5p | mRNA-miRNA |
| BAIAP3 | hsa-miR-1307-3p | mRNA-miRNA |
| BAIAP3 | hsa-miR-3615 | mRNA-miRNA |
| WDR45 | hsa-miR-330-5p | mRNA-miRNA |
| TSC2 | hsa-miR-148a-3p | mRNA-miRNA |

SPG7	hsa-miR-330-5p mRNA-miRNA hsa-miR-942-5p	COLCA1	lncRNA-miRNA

hsa-miR-942-5p	AC073333.8	lncRNA-miRNA

hsa-miR-942-5p	LNC_001195	lncRNA-miRNA

hsa-miR-942-5p	LNC_001960	lncRNA-miRNA

hsa-miR-942-5p	LNC_005052	lncRNA-miRNA

hsa-miR-548ad-5p LNC_004962	lncRNA-miRNA

hsa-miR-548ad-5p LNC_005052	lncRNA-miRNA

hsa-miR-548ad-5p LNC_005431	lncRNA-miRNA

novel_384	DLEU1	lncRNA-miRNA

novel_384	LNC_000026	lncRNA-miRNA

novel_384	LNC_000275	lncRNA-miRNA

novel_384	LNC_000635	lncRNA-miRNA

novel_384	LNC_001399	lncRNA-miRNA

novel_384	LNC_001866	lncRNA-miRNA

novel_384	LNC_001934	lncRNA-miRNA

novel_384	LNC_002228	lncRNA-miRNA

novel_384	LNC_002436	lncRNA-miRNA

novel_384	LNC_002977	lncRNA-miRNA

novel_384	LNC_003132	lncRNA-miRNA

novel_384	LNC_003818	lncRNA-miRNA

novel_384	LNC_004513	lncRNA-miRNA

novel_384	LNC_004840	lncRNA-miRNA

novel_384	LNC_004995	lncRNA-miRNA

novel_384	LNC_005755	lncRNA-miRNA

hsa-miR-551b-5p	LNC_002228	lncRNA-miRNA

hsa-miR-551b-5p	LNC_005755	lncRNA-miRNA

hsa-miR-548ah-3p LNC_000275	lncRNA-miRNA

hsa-miR-548ah-3p LNC_002228	lncRNA-miRNA

hsa-miR-548ah-3p LNC_003132	lncRNA-miRNA

hsa-miR-548ah-3p LNC_004840	lncRNA-miRNA

hsa-miR-548t-3p COLCA1	lncRNA-miRNA hsa-miR-548t-3p RP11-24F11.5 lncRNA-miRNA hsa-miR-548t-3p LNC_001195	lncRNA-miRNA

hsa-miR-548t-3p	LNC_004962	lncRNA-miRNA

hsa-miR-548t-3p	LNC_005431	lncRNA-miRNA

hsa-miR-143-3p	LNC_001866	lncRNA-miRNA

hsa-miR-143-3p	LNC_002228	lncRNA-miRNA hsa-miR-3144-3p	RP11-24F11.5	lncRNA-miRNA

hsa-miR-3144-3p	LNC_004962	lncRNA-miRNA

hsa-miR-550b-2-5p LNC_005052	lncRNA-miRNA

hsa-miR-4661-5p LNC_003818	lncRNA-miRNA hsa-miR-27b-3p RP11-637C24.2 lncRNA-miRNA hsa-miR-27b-3p	LNC_000605	lncRNA-miRNA

hsa-miR-27b-3p	LNC_005431	lncRNA-miRNA

hsa-miR-455-5p	LNC_001399	lncRNA-miRNA

hsa-miR-455-5p	LNC_002977	lncRNA-miRNA

hsa-miR-146b-5p	LNC_002977	lncRNA-miRNA

hsa-miR-146b-5p	LNC_005755	lncRNA-miRNA

hsa-miR-130a-5p	C20orf197	lncRNA-miRNA

hsa-miR-130a-5p	LNC_001195	lncRNA-miRNA

| hsa-miR-130a-5p | LNC_004962 | lncRNA-miRNA |
| --- | --- | --- |
| hsa-miR-130a-5p | LNC_005052 | lncRNA-miRNA |
| hsa-miR-200c-3p | LNC_000275 | lncRNA-miRNA |
| hsa-miR-200c-3p | LNC_002228 | lncRNA-miRNA |
| hsa-miR-200c-3p | LNC_004995 | lncRNA-miRNA |
| hsa-miR-141-3p | LNC_003132 | lncRNA-miRNA |
| hsa-miR-196a-5p | COLCA1 | lncRNA-miRNA |
| hsa-miR-196a-5p | C20orf197 | lncRNA-miRNA |
| hsa-miR-196a-5p | LNC_001195 | lncRNA-miRNA |
| hsa-miR-196a-5p | LNC_005052 | lncRNA-miRNA |
| hsa-miR-148b-3p | LNC_005052 | lncRNA-miRNA |
| hsa-miR-3913-5p | LNC_001866 | lncRNA-miRNA |
| hsa-miR-3913-5p | LNC_002436 | lncRNA-miRNA |
| hsa-miR-3913-5p | LNC_002977 | lncRNA-miRNA |
| hsa-miR-19b-3p | RP11-637C24.2 | lncRNA-miRNA |
| hsa-miR-19b-3p | LNC_004962 | lncRNA-miRNA |
| hsa-miR-193b-3p | LNC_004513 | lncRNA-miRNA |
| hsa-miR-193b-3p | LNC_004840 | lncRNA-miRNA |
| hsa-miR-4521 | C20orf197 | lncRNA-miRNA |
| hsa-miR-4521 | LNC_001195 | lncRNA-miRNA |
| hsa-miR-3615 | LNC_002436 | lncRNA-miRNA |
| hsa-miR-4529-5p | DLEU1 | lncRNA-miRNA |
| hsa-miR-4529-5p | LNC_001866 | lncRNA-miRNA |
| hsa-miR-4529-5p | LNC_003132 | lncRNA-miRNA |
| hsa-miR-4529-5p | LNC_003818 | lncRNA-miRNA |
| hsa-miR-4529-5p | LNC_004995 | lncRNA-miRNA |
| hsa-miR-769-5p | LNC_004995 | lncRNA-miRNA |
| hsa-miR-130b-3p | RP11-637C24.2 | lncRNA-miRNA |
| hsa-miR-130b-3p | LNC_000605 | lncRNA-miRNA |
| hsa-miR-130b-3p | LNC_001195 | lncRNA-miRNA |
| hsa-miR-532-5p | LNC_001866 | lncRNA-miRNA |
| hsa-miR-1843 | LNC_001866 | lncRNA-miRNA |
| hsa-miR-1843 | LNC_002977 | lncRNA-miRNA |
| hsa-miR-181b-3p | COLCA1 | lncRNA-miRNA |
| hsa-miR-181b-3p | LNC_005052 | lncRNA-miRNA |
| hsa-miR-181a-3p | RP11-637C24.2 | lncRNA-miRNA |
| hsa-miR-181a-3p | LNC_001195 | lncRNA-miRNA |
| hsa-miR-216b-5p | LNC_004995 | lncRNA-miRNA |
| hsa-miR-3129-5p | COLCA1 | lncRNA-miRNA |
| hsa-miR-3129-5p | RP11-219G17.4 | lncRNA-miRNA |
| hsa-miR-3129-5p | LNC_005052 | lncRNA-miRNA |
| hsa-miR-3129-5p | LNC_005431 | lncRNA-miRNA |
| hsa-miR-3129-3p | LNC_001960 | lncRNA-miRNA |
| novel_560 | DLEU1 | lncRNA-miRNA |
| novel_560 | RP11-384F7.2 | lncRNA-miRNA |
| novel_560 | LNC_000026 | lncRNA-miRNA |
| novel_560 | LNC_000275 | lncRNA-miRNA |
| novel_560 | LNC_000635 | lncRNA-miRNA |
| novel_560 | LNC_001399 | lncRNA-miRNA |
| novel_560 | LNC_001866 | lncRNA-miRNA |
| novel_560 | LNC_001934 | lncRNA-miRNA |

novel_560	LNC_002228	lncRNA-miRNA

novel_560	LNC_002436	lncRNA-miRNA

novel_560	LNC_002977	lncRNA-miRNA

novel_560	LNC_003132	lncRNA-miRNA

novel_560	LNC_003818	lncRNA-miRNA

novel_560	LNC_004513	lncRNA-miRNA

novel_560	LNC_004840	lncRNA-miRNA

novel_560	LNC_004995	lncRNA-miRNA

novel_560	LNC_005755	lncRNA-miRNA

hsa-miR-425-5p	LNC_002436	lncRNA-miRNA

hsa-miR-580-3p	LNC_005052	lncRNA-miRNA

hsa-miR-148a-3p	LNC_000275	lncRNA-miRNA

hsa-miR-148a-3p	LNC_004995	lncRNA-miRNA

hsa-miR-196b-5p	C20orf197	lncRNA-miRNA

hsa-miR-550a-3p	LNC_005052	lncRNA-miRNA

hsa-miR-550b-2-5p COLCA1	lncRNA-miRNA hsa-miR-550b-2-5p RP11-24F11.5	lncRNA-miRNA

hsa-miR-548n	RP11-24F11.5	lncRNA-miRNA hsa-miR-548n	LNC_004962	lncRNA-miRNA

hsa-miR-548n	LNC_005052	lncRNA-miRNA

hsa-miR-548n	LNC_005431	lncRNA-miRNA

hsa-miR-96-5p	LNC_001866	lncRNA-miRNA

hsa-miR-96-5p	LNC_004513	lncRNA-miRNA

hsa-miR-96-5p	LNC_004995	lncRNA-miRNA

hsa-miR-183-5p	LNC_002436	lncRNA-miRNA

hsa-miR-598-3p	LNC_005052	lncRNA-miRNA

hsa-miR-548d-5p	LNC_004962	lncRNA-miRNA

hsa-miR-548d-5p	LNC_005052	lncRNA-miRNA

hsa-miR-548d-5p	LNC_005431	lncRNA-miRNA hsa-miR-1287-5p	RP11-219G17.4	lncRNA-miRNA

hsa-miR-1287-5p	C20orf197	lncRNA-miRNA

hsa-miR-1287-5p	LNC_005431	lncRNA-miRNA

hsa-miR-1293	LNC_000275	lncRNA-miRNA

hsa-miR-1293	LNC_001866	lncRNA-miRNA

hsa-miR-1293	LNC_002436	lncRNA-miRNA

hsa-miR-1293	LNC_002977	lncRNA-miRNA

hsa-miR-1268a	COLCA1	lncRNA-miRNA

hsa-miR-1268a	LNC_000305	lncRNA-miRNA

hsa-miR-22-3p	LNC_000275	lncRNA-miRNA

hsa-miR-22-3p	LNC_000635	lncRNA-miRNA

hsa-miR-22-3p	LNC_002977	lncRNA-miRNA

hsa-miR-212-5p	LNC_000275	lncRNA-miRNA

hsa-miR-212-5p	LNC_001934	lncRNA-miRNA

hsa-miR-212-5p	LNC_004995	lncRNA-miRNA

hsa-miR-195-5p	LNC_005052	lncRNA-miRNA

hsa-miR-122b-3p	DLEU1	lncRNA-miRNA

hsa-miR-122b-3p	LNC_002977	lncRNA-miRNA

hsa-miR-122b-3p	LNC_004995	lncRNA-miRNA

hsa-miR-3940-3p	LNC_000017	lncRNA-miRNA

hsa-miR-3940-3p	LNC_000058	lncRNA-miRNA

hsa-miR-330-5p	LNC_001866	lncRNA-miRNA

| hsa-miR-330-5p | LNC_002436 | lncRNA-miRNA |
| --- | --- | --- |
| hsa-miR-221-5p | RP11-637C24.2 | lncRNA-miRNA |
| hsa-miR-221-5p | LNC_001195 | lncRNA-miRNA |
| hsa-miR-221-5p | LNC_005052 | lncRNA-miRNA |
| hsa-miR-221-5p | LNC_005431 | lncRNA-miRNA |
| hsa-miR-374a-5p | LNC_001195 | lncRNA-miRNA |
| hsa-miR-374a-3p | C20orf197 | lncRNA-miRNA |
| hsa-miR-374a-3p | LNC_005052 | lncRNA-miRNA |
| hsa-miR-374a-3p | LNC_005431 | lncRNA-miRNA |
| hsa-miR-361-5p | C20orf197 | lncRNA-miRNA |
| hsa-miR-361-5p | LNC_005052 | lncRNA-miRNA |
| hsa-miR-361-5p | LNC_005431 | lncRNA-miRNA |
| hsa-miR-424-5p | RP11-219G17.4 | lncRNA-miRNA |
| hsa-miR-424-5p | LNC_001195 | lncRNA-miRNA |

hsa-miR-424-5p	LNC_005052	lncRNA-miRNA

#  Table 5.2: node information

**node	info	group**

| hsa-let-7g-5p  hsa-let-7i-3p  hsa-let-7i-5p | down  down  down | miRNA  miRNA  miRNA |
| --- | --- | --- |
| hsa-miR-103a-2-5p | down | miRNA |
| hsa-miR-122-5p | up | miRNA |
| hsa-miR-122b-3p | up | miRNA |
| hsa-miR-1246 | up | miRNA |
| hsa-miR-1268a | down | miRNA |
| hsa-miR-1287-5p | down | miRNA |
| hsa-miR-1293 | up | miRNA |
| hsa-miR-1307-3p | up | miRNA |
| hsa-miR-130a-3p | down | miRNA |
| hsa-miR-130a-5p | down | miRNA |
| hsa-miR-130b-3p | down | miRNA |
| hsa-miR-1322 | up | miRNA |
| hsa-miR-132-5p | up | miRNA |
| hsa-miR-133a-3p | up | miRNA |
| hsa-miR-135a-2-3p | down | miRNA |
| hsa-miR-139-5p | up | miRNA |
| hsa-miR-1-3p | up | miRNA |
| hsa-miR-141-3p | up | miRNA |
| hsa-miR-143-3p | up | miRNA |
| hsa-miR-146b-5p | up | miRNA |
| hsa-miR-147b-3p | up | miRNA |
| hsa-miR-148a-3p | up | miRNA |
| hsa-miR-148b-3p | down | miRNA |
| hsa-miR-151a-3p | up | miRNA |
| hsa-miR-152-3p | down | miRNA |
| hsa-miR-15b-5p | down | miRNA |
| hsa-miR-16-5p | down | miRNA |
| hsa-miR-181a-3p | down | miRNA |

| hsa-miR-181b-3p | down | miRNA |
| --- | --- | --- |
| hsa-miR-183-5p | up | miRNA |
| hsa-miR-1843 | up | miRNA |
| hsa-miR-193b-3p | up | miRNA |
| hsa-miR-195-3p | up | miRNA |
| hsa-miR-195-5p | down | miRNA |
| hsa-miR-196a-5p | down | miRNA |
| hsa-miR-196b-5p | down | miRNA |
| hsa-miR-19b-3p | down | miRNA |
| hsa-miR-200c-3p | up | miRNA |
| hsa-miR-204-3p | up | miRNA |
| hsa-miR-204-5p | up | miRNA |
| hsa-miR-205-5p | up | miRNA |
| hsa-miR-212-5p | up | miRNA |
| hsa-miR-214-3p | up | miRNA |
| hsa-miR-216a-3p | up | miRNA |
| hsa-miR-216b-5p | up | miRNA |
| hsa-miR-219a-1-3p | up | miRNA |
| hsa-miR-221-5p | down | miRNA |
| hsa-miR-22-3p | up | miRNA |
| hsa-miR-22-5p | up | miRNA |
| hsa-miR-23a-3p | down | miRNA |
| hsa-miR-23b-3p | down | miRNA |
| hsa-miR-27b-3p | down | miRNA |
| hsa-miR-29b-1-5p | down | miRNA |
| hsa-miR-302a-5p | down | miRNA |
| hsa-miR-30b-5p | down | miRNA |
| hsa-miR-3117-3p | down | miRNA |
| hsa-miR-3129-3p | down | miRNA |
| hsa-miR-3129-5p | down | miRNA |
| hsa-miR-3144-3p | down | miRNA |
| hsa-miR-3150a-5p | up | miRNA |
| hsa-miR-3150b-3p | up | miRNA |
| hsa-miR-3158-3p | up | miRNA |
| hsa-miR-3158-5p | up | miRNA |
| hsa-miR-320a-5p | down | miRNA |
| hsa-miR-330-5p | up | miRNA |
| hsa-miR-331-5p | up | miRNA |
| hsa-miR-335-3p | down | miRNA |
| hsa-miR-335-5p | down | miRNA |
| hsa-miR-338-5p | down | miRNA |
| hsa-miR-33a-3p | down | miRNA |
| hsa-miR-3615 | up | miRNA |
| hsa-miR-361-5p | down | miRNA |
| hsa-miR-363-3p | up | miRNA |
| hsa-miR-365b-5p | up | miRNA |
| hsa-miR-3662 | up | miRNA |
| hsa-miR-374a-3p | down | miRNA |
| hsa-miR-374a-5p | down | miRNA |
| hsa-miR-375-3p | up | miRNA |
| hsa-miR-3909 | up | miRNA |

| hsa-miR-3913-3p | up | miRNA |
| --- | --- | --- |
| hsa-miR-3913-5p | up | miRNA |
| hsa-miR-3924 | up | miRNA |
| hsa-miR-3940-3p | down | miRNA |
| hsa-miR-424-5p | down | miRNA |
| hsa-miR-425-5p | up | miRNA |
| hsa-miR-4521 | down | miRNA |
| hsa-miR-4529-5p | up | miRNA |
| hsa-miR-455-5p | up | miRNA |
| hsa-miR-4635 | up | miRNA |
| hsa-miR-4637 | down | miRNA |
| hsa-miR-4661-5p | up | miRNA |
| hsa-miR-4664-3p | up | miRNA |
| hsa-miR-4775 | down | miRNA |
| hsa-miR-499a-5p | up | miRNA |
| hsa-miR-499b-3p | up | miRNA |
| hsa-miR-509-3-5p | up | miRNA |
| hsa-miR-514a-3p | up | miRNA |
| hsa-miR-532-5p | up | miRNA |
| hsa-miR-548ad-5p | down | miRNA |
| hsa-miR-548ah-3p | up | miRNA |
| hsa-miR-548ap-3p | down | miRNA |
| hsa-miR-548az-5p | up | miRNA |
| hsa-miR-548d-5p | down | miRNA |
| hsa-miR-548f-3p | up | miRNA |
| hsa-miR-548n | down | miRNA |
| hsa-miR-548o-3p | up | miRNA |
| hsa-miR-548t-3p | down | miRNA |
| hsa-miR-549a-3p | up | miRNA |
| hsa-miR-549a-5p | up | miRNA |
| hsa-miR-550a-3p | down | miRNA |
| hsa-miR-550b-2-5p | down | miRNA |
| hsa-miR-551b-5p | up | miRNA |
| hsa-miR-5586-3p | down | miRNA |
| hsa-miR-5690 | up | miRNA |
| hsa-miR-578 | up | miRNA |
| hsa-miR-580-3p | down | miRNA |
| hsa-miR-590-3p | down | miRNA |
| hsa-miR-598-3p | down | miRNA |
| hsa-miR-615-3p | up | miRNA |
| hsa-miR-627-5p | down | miRNA |
| hsa-miR-628-5p | down | miRNA |
| hsa-miR-641 | up | miRNA |
| hsa-miR-659-5p | up | miRNA |
| hsa-miR-6866-5p | up | miRNA |
| hsa-miR-769-5p | up | miRNA |
| hsa-miR-7704 | up | miRNA |
| hsa-miR-7974 | up | miRNA |
| hsa-miR-887-3p | down | miRNA |
| hsa-miR-892a | up | miRNA |
| hsa-miR-892c-3p | up | miRNA |

| hsa-miR-933 | up | miRNA |
| --- | --- | --- |
| hsa-miR-935 | down | miRNA |
| hsa-miR-942-5p | down | miRNA |
| hsa-miR-96-5p | up | miRNA |
| novel_189 | up | miRNA |
| novel_192 | up | miRNA |
| novel_384 | up | miRNA |
| novel_560 | up | miRNA |
| novel_66 | up | miRNA |
| novel_778 | up | miRNA |
| AC073333.8 | up | lncRNA |
| C20orf197 | up | lncRNA |
| COLCA1 | up | lncRNA |
| DLEU1 | down | lncRNA |
| hsa_circ_0000629 | down | lncRNA |
| hsa_circ_0001051 | up | lncRNA |
| hsa_circ_0001098 | down | lncRNA |
| hsa_circ_0001315 | down | lncRNA |
| hsa_circ_0001358 | up | lncRNA |
| hsa_circ_0001417 | up | lncRNA |
| hsa_circ_0001481 | down | lncRNA |
| hsa_circ_0001575 | up | lncRNA |
| hsa_circ_0001946 | up | lncRNA |
| hsa_circ_0002348 | down | lncRNA |
| hsa_circ_0002750 | down | lncRNA |
| hsa_circ_0002762 | down | lncRNA |
| hsa_circ_0003045 | up | lncRNA |
| hsa_circ_0003102 | down | lncRNA |
| hsa_circ_0003221 | down | lncRNA |
| hsa_circ_0003322 | down | lncRNA |
| hsa_circ_0003694 | down | lncRNA |
| hsa_circ_0003877 | down | lncRNA |
| hsa_circ_0003898 | down | lncRNA |
| hsa_circ_0003909 | down | lncRNA |
| hsa_circ_0004364 | down | lncRNA |
| hsa_circ_0005252 | down | lncRNA |
| hsa_circ_0005309 | down | lncRNA |
| hsa_circ_0005581 | up | lncRNA |
| hsa_circ_0005898 | down | lncRNA |
| hsa_circ_0006010 | up | lncRNA |
| hsa_circ_0006087 | up | lncRNA |
| hsa_circ_0006117 | up | lncRNA |
| hsa_circ_0006360 | down | lncRNA |
| hsa_circ_0006393 | up | lncRNA |
| hsa_circ_0006727 | up | lncRNA |
| hsa_circ_0006801 | down | lncRNA |
| hsa_circ_0007001 | down | lncRNA |
| hsa_circ_0007130 | down | lncRNA |
| hsa_circ_0007178 | down | lncRNA |
| hsa_circ_0007701 | down | lncRNA |
| hsa_circ_0007788 | down | lncRNA |

| hsa_circ_0007804 | up | lncRNA |
| --- | --- | --- |
| hsa_circ_0007976 | down | lncRNA |
| hsa_circ_0008075 | up | lncRNA |
| hsa_circ_0008351 | down | lncRNA |
| hsa_circ_0008433 | up | lncRNA |
| hsa_circ_0008694 | down | lncRNA |
| hsa_circ_0008798 | up | lncRNA |
| hsa_circ_0009008 | down | lncRNA |
| hsa_circ_0009064 | down | lncRNA |
| hsa_circ_0009110 | up | lncRNA |
| hsa_circ_0012823 | up | lncRNA |
| hsa_circ_0013167 | up | lncRNA |
| hsa_circ_0014292 | up | lncRNA |
| hsa_circ_0014577 | up | lncRNA |
| hsa_circ_0015260 | down | lncRNA |
| hsa_circ_0018524 | down | lncRNA |
| hsa_circ_0018992 | down | lncRNA |
| hsa_circ_0038816 | up | lncRNA |
| hsa_circ_0041152 | down | lncRNA |
| hsa_circ_0042498 | down | lncRNA |
| hsa_circ_0048000 | down | lncRNA |
| hsa_circ_0052943 | up | lncRNA |
| hsa_circ_0058051 | down | lncRNA |
| hsa_circ_0061774 | down | lncRNA |
| hsa_circ_0061776 | down | lncRNA |
| hsa_circ_0062642 | down | lncRNA |
| hsa_circ_0069101 | down | lncRNA |
| hsa_circ_0073706 | up | lncRNA |
| hsa_circ_0073904 | up | lncRNA |
| hsa_circ_0075346 | up | lncRNA |
| hsa_circ_0079040 | down | lncRNA |
| hsa_circ_0080835 | down | lncRNA |
| hsa_circ_0081808 | up | lncRNA |
| hsa_circ_0087234 | down | lncRNA |
| hsa_circ_0089466 | up | lncRNA |
| hsa_circ_0091537 | up | lncRNA |
| LNC_000017 | up | lncRNA |
| LNC_000026 | down | lncRNA |
| LNC_000058 | up | lncRNA |
| LNC_000275 | down | lncRNA |
| LNC_000305 | up | lncRNA |
| LNC_000605 | up | lncRNA |
| LNC_000635 | down | lncRNA |
| LNC_001195 | up | lncRNA |
| LNC_001399 | down | lncRNA |
| LNC_001866 | down | lncRNA |
| LNC_001934 | down | lncRNA |
| LNC_001960 | up | lncRNA |
| LNC_002228 | down | lncRNA |
| LNC_002436 | down | lncRNA |
| LNC_002977 | down | lncRNA |

| LNC_003132 | down | lncRNA |
| --- | --- | --- |
| LNC_003818 | down | lncRNA |
| LNC_004513 | down | lncRNA |
| LNC_004840 | down | lncRNA |
| LNC_004962 | up | lncRNA |
| LNC_004995 | down | lncRNA |
| LNC_005052 | up | lncRNA |
| LNC_005431 | up | lncRNA |
| LNC_005755 | down | lncRNA |
| novel_circ_0000067 | down | lncRNA |
| novel_circ_0000381 | down | lncRNA |
| novel_circ_0000551 | up | lncRNA |
| novel_circ_0000640 | up | lncRNA |
| novel_circ_0000647 | up | lncRNA |
| novel_circ_0000660 | up | lncRNA |
| novel_circ_0000687 | down | lncRNA |
| novel_circ_0000932 | up | lncRNA |
| novel_circ_0000982 | down | lncRNA |
| novel_circ_0001089 | down | lncRNA |
| novel_circ_0001101 | up | lncRNA |
| novel_circ_0001102 | up | lncRNA |
| novel_circ_0001283 | up | lncRNA |
| novel_circ_0001295 | down | lncRNA |
| novel_circ_0001322 | down | lncRNA |
| novel_circ_0001378 | up | lncRNA |
| novel_circ_0001748 | down | lncRNA |
| novel_circ_0001790 | down | lncRNA |
| novel_circ_0002102 | down | lncRNA |
| novel_circ_0002721 | down | lncRNA |
| novel_circ_0002797 | down | lncRNA |
| novel_circ_0002996 | up | lncRNA |
| novel_circ_0003249 | down | lncRNA |
| RP11-219G17.4 | up | lncRNA |
| RP11-24F11.5 | up | lncRNA |
| RP11-384F7.2 | down | lncRNA |
| RP11-637C24.2 | up | lncRNA |
| ABLIM1 | up | mRNA |
| AC093512.2 | down | mRNA |
| ACSF3 | down | mRNA |
| ACTR1A | down | mRNA |
| ADGRG1 | up | mRNA |
| ANAPC11 | down | mRNA |
| ANGPTL4 | down | mRNA |
| ANO10 | down | mRNA |
| APLP1 | down | mRNA |
| ARFRP1 | down | mRNA |
| ARHGDIA | up | mRNA |
| ARL6IP4 | down | mRNA |
| BAIAP3 | down | mRNA |
| BCAP31 | down | mRNA |
| BMPR1B | up | mRNA |

| C11orf96 | up | mRNA |
| --- | --- | --- |
| C19orf48 | up | mRNA |
| CALCRL | up | mRNA |
| CAPN1 | down | mRNA |
| CAPRIN2 | up | mRNA |
| CARHSP1 | down | mRNA |
| CATIP | up | mRNA |
| CD151 | down | mRNA |
| CEP131 | down | mRNA |
| CHD1L | down | mRNA |
| CHRDL1 | up | mRNA |
| CLCN2 | down | mRNA |
| CLCN7 | up | mRNA |
| CST2 | up | mRNA |
| CTDP1 | down | mRNA |
| CTSB | up | mRNA |
| DAB2IP | down | mRNA |
| DGKQ | down | mRNA |
| DHRS3 | down | mRNA |
| DNASE1 | down | mRNA |
| DOK1 | down | mRNA |
| ECEL1 | up | mRNA |
| EFTUD2 | up | mRNA |
| EGR4 | up | mRNA |
| FAM160A2 | up | mRNA |
| FAM19A5 | down | mRNA |
| FDFT1 | up | mRNA |
| FHAD1 | down | mRNA |
| FLNA | down | mRNA |
| FLNA | down | mRNA |
| FLYWCH1 | down | mRNA |
| FOSB | up | mRNA |
| HPD | up | mRNA |
| IKBKB | down | mRNA |
| IMPDH1 | down | mRNA |
| KCTD17 | down | mRNA |
| LRRC4B | down | mRNA |
| MPP1 | down | mRNA |
| MRC2 | down | mRNA |
| MRPL10 | up | mRNA |
| MT1X | down | mRNA |
| MT2A | down | mRNA |
| MTA1 | down | mRNA |
| NPEPPS | up | mRNA |
| OBSL1 | up | mRNA |
| ORC1 | up | mRNA |
| OS9 | up | mRNA |
| PES1 | down | mRNA |
| PHGDH | down | mRNA |
| PIK3R1 | up | mRNA |
| PLD2 | down | mRNA |

| PNPLA6 | down | mRNA |
| --- | --- | --- |
| PTP4A3 | down | mRNA |
| PTPRF | down | mRNA |
| RASAL1 | up | mRNA |
| SERPINA3 | down | mRNA |
| SHISA5 | down | mRNA |
| SLC22A3 | up | mRNA |
| SMARCC2 | down | mRNA |
| SPG7 | down | mRNA |
| SVEP1 | up | mRNA |
| TBCB | up | mRNA |
| TCOF1 | up | mRNA |
| TENM2 | down | mRNA |
| TMEM132E | down | mRNA |
| TMEM51 | down | mRNA |
| TSC2 | down | mRNA |
| TTC9C | down | mRNA |
| TUBGCP3 | down | mRNA |
| TUFT1 | down | mRNA |
| UBE2V1 | up | mRNA |
| UNKL | down | mRNA |
| WDR45 | down | mRNA |
| ZC4H2 | up | mRNA |

ZNF446	down	mRNA
